# Catalytic enantioselective synthesis of perfluoroalkyl-substituted β-lactones *via* a concerted asynchronous [2 + 2] cycloaddition: a synthetic and computational study†
†Electronic supplementary information (ESI) available: Experimental procedures, product characterisation data (mpt, NMR, IR, HRMS, [*α*]_D_, HPLC), traces (^1^H, ^13^C, ^19^F NMR, HPLC), X-ray crystallographic data (CCDC 1886142, CCDC 1886143 and CCDC 1886144), coordinates, thermal corrections and energies of all computed structures. CCDC 1886142–1886144. For ESI and crystallographic data in CIF or other electronic format see DOI: 10.1039/c9sc00390h


**DOI:** 10.1039/c9sc00390h

**Published:** 2019-04-29

**Authors:** Diego-Javier Barrios Antúnez, Mark D. Greenhalgh, Alexander C. Brueckner, Daniel M. Walden, Pilar Elías-Rodríguez, Patrick Roberts, Benjamin G. Young, Thomas H. West, Alexandra M. Z. Slawin, Paul Ha-Yeon Cheong, Andrew D. Smith

**Affiliations:** a EaStCHEM , School of Chemistry , University of St Andrews , North Haugh , St Andrews , KY16 9ST , UK . Email: ads10@st-andrews.ac.uk; b Department of Chemistry , Oregon State University , 153 Gilbert Hall , Corvallis , Oregon 97333 , USA . Email: cheongh@oregonstate.edu; c Department of Chemistry, Physics, and Engineering , Biola University , 315 Lim Center , La Mirada , California 90639 , USA

## Abstract

The enantioselective preparation of a range of perfluoroalkyl-substituted β-lactones through an isothiourea (HyperBTM) catalysed reaction using symmetric anhydrides as ammonium enolate precursors and perfluoroalkylketones (R_F_ = CF_3_, C_2_F_5_, C_4_F_9_) is reported. Following optimisation, high diastereo- and enantioselectivity was observed for β-lactone formation using C_2_F_5_- and C_4_F_9_-substituted ketones at room temperature (26 examples, up to >95 : 5 dr and >99 : 1 er), whilst –78 °C was necessary for optimal dr and er with CF_3_-substituted ketones (11 examples, up to >95 : 5 dr and >99 : 1 er). Derivatisation of the β-lactones through ring-opening, as well as a two-step conversion to give perfluoroalkyl-substituted oxetanes, is demonstrated without loss of stereochemical integrity. Density functional theory computations, alongside ^13^C natural abundance KIE studies, have been used to probe the reaction mechanism with a concerted asynchronous [2 + 2]-cycloaddition pathway favoured over a stepwise aldol–lactonisation process.

## Abstract 2

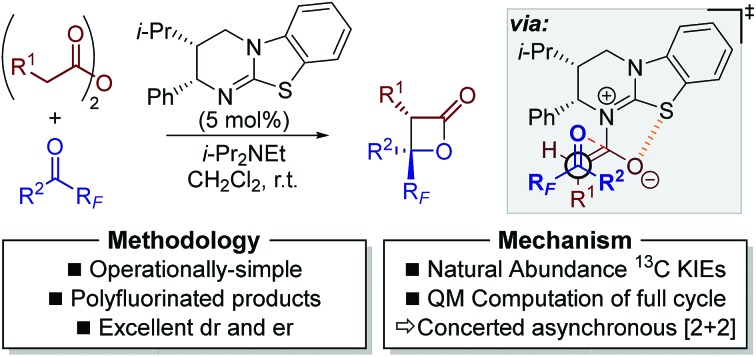
A study into the Lewis base-catalysed enantioselective synthesis of polyfluorinated β-lactones.

## 1. Introduction

The enantioselective preparation of β-lactones remains a significant goal in chemical synthesis due to the synthetic versatility of β-lactones and their presence in numerous natural products and bioactive compounds.^1^ Accordingly, a range of approaches has been reported for the synthesis of enantioenriched β-lactones, including substrate-, chiral auxiliary- and catalyst-controlled processes. Although enantioselective Lewis acid catalysis has proven a successful approach,^2^ the majority of catalytic methods involve the use of chiral Lewis basic catalysts.^3^

**Scheme 1:**
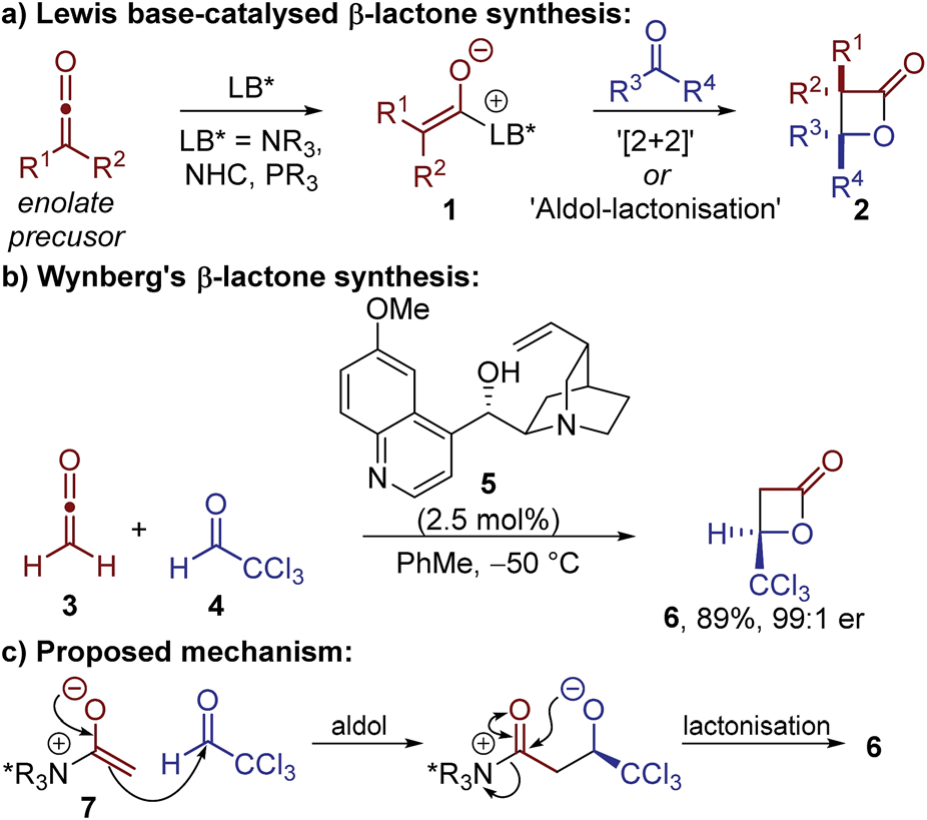
Synthesis and previously proposed mechanism for the formation of β-lactones using Lewis base catalysis.

Lewis base-catalysed β-lactone synthesis is generally proposed to proceed by generation of a chiral enolate **1** which reacts with an electrophilic carbonyl-containing substrate to give a β-lactone product **2** (Scheme 1a). In principle, this reaction could take place through either a [2 + 2] cycloaddition, or a stepwise aldol–lactonisation process. Most methods have focussed on using highly-reactive ketenes as the enolate precursor, in ketene dimerisations or through reaction with aldehydes.^4^–^9^ Although N-heterocyclic carbenes (NHCs) and phosphines have been used in these processes,^4^,^5^ the majority of examples have reported the use of tertiary amine catalysts – in particular *Cinchona* alkaloid derivatives.^7^,8a–d,f–i,^9^

Building on the work of Borrmann and Wegler,^6^ a series of seminal studies by Wynberg in the 1980s demonstrated that quinidine **5** could catalyse the highly enantioselective formal [2 + 2] cycloaddition between ketene **3** and chloral **4** (Scheme 1b).^7^ It was proposed the reaction proceeded by addition of quinidine **5** to ketene **3** to give a C(1)-ammonium enolate intermediate **7**,3*b* followed by reaction with chloral **4***via* an aldol–lactonisation process (Scheme 1c). This stepwise mechanism was preferred over the thermally-forbidden concerted [2 + 2] cycloaddition, however no experimental evidence was provided to support this assertion. The scope and applicability of this transformation has been expanded by Calter, Romo, Nelson and Fu, amongst others, to allow the use of di- or monosubstituted ketenes (isolated or generated *in situ*) with a broader range of aldehydes.^8^ The power of these synthetic methods has been exemplified through their application in a number of elegant total syntheses.^9^ Despite these significant advances, no experimental or computational studies of the reaction mechanism using tertiary amine catalysts have been published, with the reaction usually assumed to proceed by a stepwise aldol–lactonisation pathway.

**Scheme 2:**
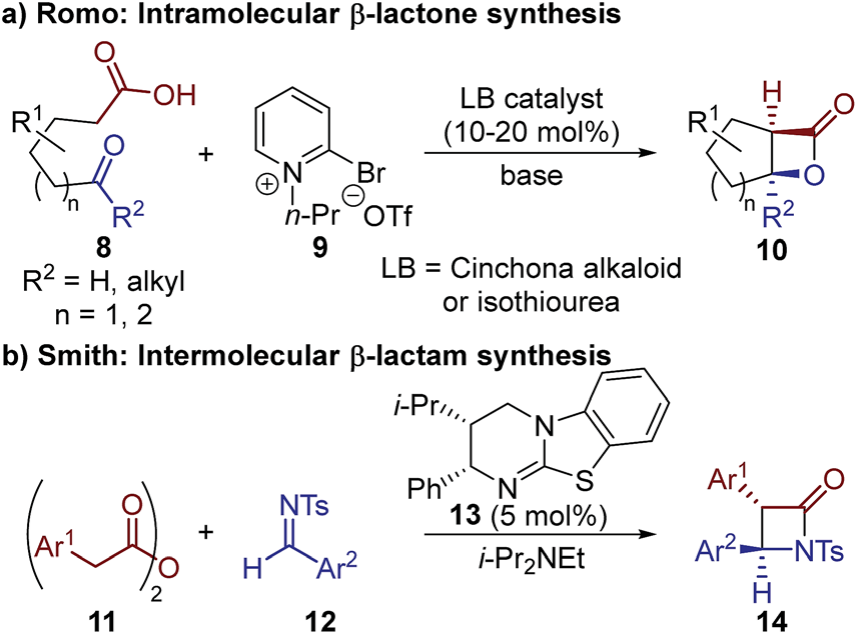
Previous applications of bench-stable carboxylic acid derivatives as ammonium enolate precursors in formal [2 + 2] cycloaddition reactions.

The limitation of using highly reactive ketenes in these reactions was originally addressed by Romo, who introduced the use of carboxylic acids as bench-stable ammonium enolate precursors (Scheme 2a).^10^ A modified Mukaiyama reagent **9** was used for *in situ* functionalisation of carboxylic acid substrates bearing pendant aldehydes or ketones **8**, to promote ammonium enolate formation and subsequent *intramolecular* formal [2 + 2] cycloaddition. We expanded this general approach to allow the use of alternative bench-stable carboxylic acid derivatives, such as anhydrides **11**, in *intermolecular* formal [2 + 2] cycloadditions (Scheme 2b).^11^ Reaction of the catalytically-generated chiral ammonium enolate intermediates with aldimines **12** was used for the diastereo- and enantioselective synthesis of β-lactams **14**; however analogous methods to generate β-lactones have not been developed.

It is widely acknowledged that the chemical and physical properties of small molecules can be significantly affected by the incorporation of fluorine and perfluorinated groups.^12^ Many drugs and drug candidates contain perfluorinated groups, making simple and robust fluorination methods an area of significant interest. Numerous methods have been reported for the enantioselective introduction of trifluoromethyl groups; however analogous methods for the introduction of longer chain perfluoroalkyl groups at stereogenic centres remains underdeveloped.^13^ To address this limitation, in previous work we reported the use of perfluoroalkyl-substituted ketones for the synthesis of β-lactones using NHC redox catalysis.^14^ Although highly enantioselective, this method was restricted to the synthesis of α-alkyl-substituted β-lactones, which proved unstable to isolation by column chromatography and therefore required *in situ* derivatisation to give acyclic products.

**Scheme 3:**
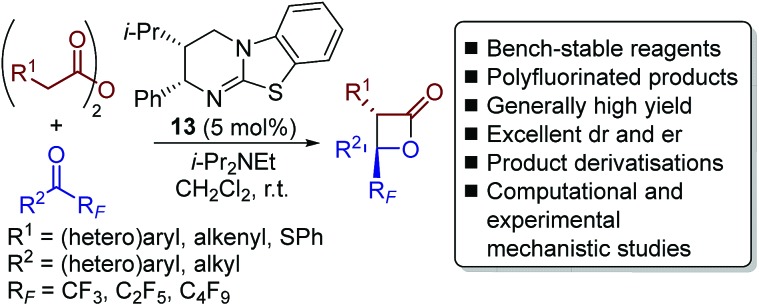
This work: enantioselective synthesis of highly-substituted perfluoroalkyl-substituted β-lactones.

Herein we report the catalytic enantioselective synthesis of perfluoroalkyl-substituted β-lactones from bench-stable anhydrides and perfluoroalkyl-substituted ketones using isothiourea catalysis (Scheme 3). This method precludes the requirement of using highly unstable ketene starting materials. Derivatisation of the enantioenriched β-lactones into a range of products, including perfluoroalkyl-substituted oxetanes, is reported in good yield and with no loss in enantiopurity. Natural abundance ^13^C kinetic isotope effect (KIE) experiments in tandem with computational analyses were utilised to investigate the reaction mechanism, with results indicating the operation of a concerted asynchronous [2 + 2] cycloaddition.

## 2. Results and discussion

### 2.1. Reaction optimisation

Initial studies for the synthesis of perfluoroalkyl-substituted β-lactones used the isothiourea catalyst HyperBTM **13**, perfluorobutyl-substituted ketone **18**, and a range of ammonium enolate precursors **15–17** in CH_2_Cl_2_ at room temperature (Table 1). First, the use of *in situ* generated mixed anhydrides was investigated. Functionalisation of phenylacetic acid with pivaloyl chloride to provide mixed anhydride **15** provided access to perfluorobutyl-substituted β-lactone **21** in 51% yield and promising stereoselectivity (92 : 8 dr, 89 : 11 er; entry 1). Co-elution of the β-lactone product **21** with pivalic anhydride complicated product isolation and therefore alternative enolate precursors were investigated. The use of *para*-methoxybenzoic mixed anhydride **16** simplified product isolation and provided β-lactone **21** with improved diastereoselectivity and enantioselectivity (entry 2). The use of bench-stable phenylacetic anhydride **17** gave β-lactone **21** in an improved yield of 61%, and with excellent diastereo- and enantioselectivity (entry 3). The use of alternative isothiourea catalysts, solvents, or reaction temperatures was not beneficial,^15^ however, increasing the equivalents of anhydride **17** provided β-lactone **21** in improved yield (entry 4). The catalyst loading could be reduced to just 1 mol% without compromising the yield or stereoselectivity of the transformation (entries 5 and 6).

**Table 1:** Reaction optimisation

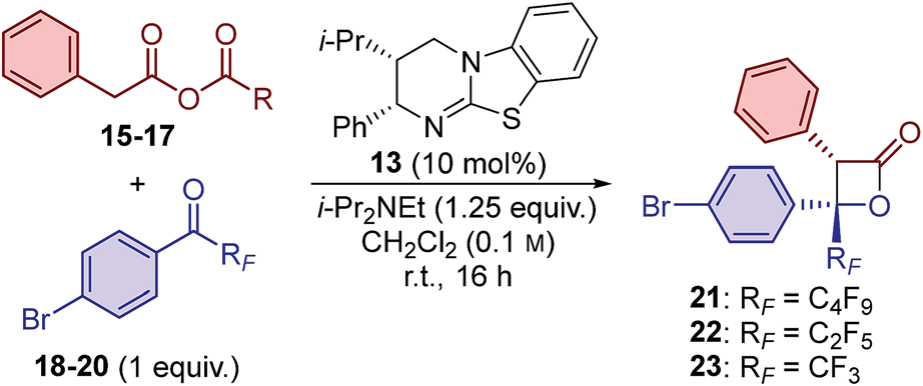					
Entry	Anhydride (equiv.)	Ketone (R_F_)	Yield (%)	dr^*a*^	er^*b*^
1	R = *t*-Bu^*c*^	**18** (C_4_F_9_)	51	92 : 8	89 : 11
	**15** (1.25)				
2	R = 4-MeO-C_6_H_4_^*c*^	**18** (C_4_F_9_)	51	95 : 5	96 : 4
	**16** (1.25)				
3	R = CH_2_Ph	**18** (C_4_F_9_)	61	>95 : 5	97 : 3
	**17** (1.25)				
4	**17** (2.5)	**18** (C_4_F_9_)	69	>95 : 5	96 : 4
5^*d*^	**17** (2.5)	**18** (C_4_F_9_)	74	>95 : 5	97 : 3
6^*e*^	**17** (2.5)	**18** (C_4_F_9_)	75	>95 : 5	97 : 3
7^*d*^	**17** (2.5)	**19** (C_2_F_5_)	75	>95 : 5	97 : 3
8^*d*^	**17** (2.5)	**20** (CF_3_)	88	88 : 12	77 : 23
9^*d*^ ^,^^*f*^	**17** (2.5)	**20** (CF_3_)	95	90 : 10	95 : 5

^*a*^Determined by ^1^H NMR spectroscopic analysis of the crude reaction product mixture.

^*b*^Determined by HPLC analysis using a chiral support.

^*c*^Mixed anhydride formed *in situ* from phenylacetic acid and the appropriate acid chloride.

^*d*^5 mol% **13** used.

^*e*^1 mol% **13** used.

^*f*^Reaction conducted at –78 °C.

Variation of the perfluoroalkyl substituent on the ketone was investigated next. The developed methodology proved equally applicable at room temperature when using perfluoroethyl-substituted ketone **19**, with β-lactone **22** obtained in 75% yield, >95 : 5 dr and 97 : 3 er (entry 7). Reducing the size of the perfluoroalkyl group led to a decrease in stereoselectivity, with trifluoroacetophenone derivative **20** providing β-lactone **23** in 88 : 12 dr and 77 : 23 er at room temperature (entry 8). Reducing the reaction temperature to –78 °C provided access to β-lactone **23** in excellent yield (95%), similar diastereoselectivity (90 : 10) but with significantly improved enantioselectivity (95 : 5; entry 9).^16^ In contrast to our previous work with α-alkyl-substituted β-lactones,^14^ all three α-aryl-substituted β-lactones (**21–23**) were easy to isolate and were found to be thermally stable up to 140 °C.^17^

### 2.2. Reaction scope and limitations

#### 2.2.1. Variation of ketone

Having demonstrated β-lactone formation with perfluorobutyl-, perfluoroethyl- and trifluoromethyl-substituted ketones, the generality of the method was investigated (Table 2).^18^*meta*-Fluorophenyl-substituted ketones bearing both perfluorobutyl and perfluoroethyl groups provided β-lactones **24** and **25** in good yield and excellent stereocontrol. The synthesis of the trifluoromethyl-substituted analogue **26** again required the reaction to be performed at –78 °C to achieve high enantioselectivity (97 : 3 er), indicating this reaction temperature to be optimal when using trifluoromethyl-substituted ketones.

**Table 2:** Scope: variation of perfluoroalkyl-substituted ketone^*a*^

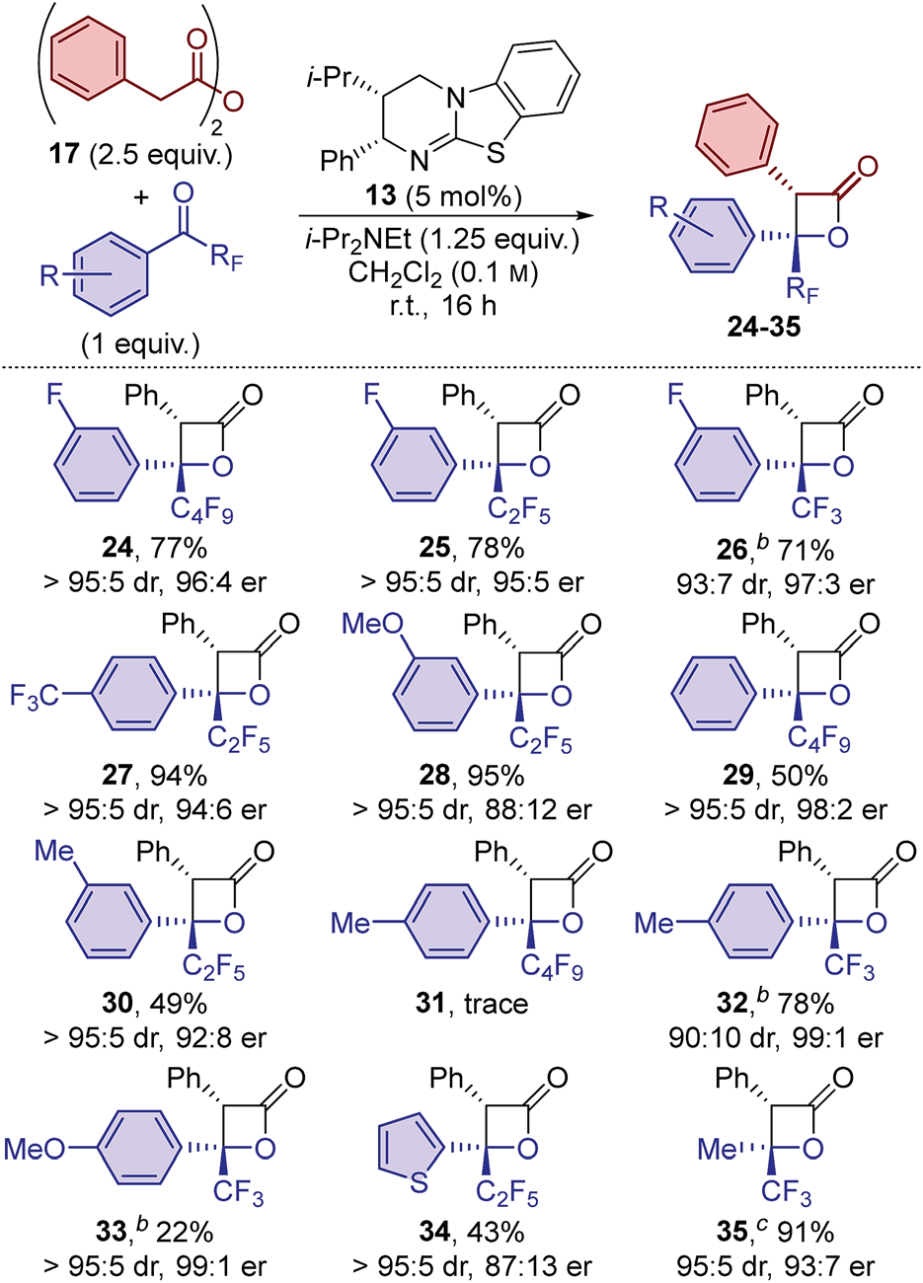

^*a*^dr determined by ^1^H NMR spectroscopic analysis of the crude reaction product mixture. er determined by HPLC analysis using a chiral support.

^*b*^Reaction conducted at –78 °C.

^*c*^Phenylacetic anhydride **17** (1 equiv.), 1,1,1-trifluoroacetone (2.5 equiv.) used, reaction conducted at 0 °C.

The electronic nature of the aromatic substituent was then investigated. The introduction of electron-withdrawing groups (positive Hammett sigma constants)^19^ was tolerated, with *p*-CF_3_ and *m*-OMe-substituted β-lactone products **27** and **28** obtained in excellent yield and with excellent diastereoselectivity and good enantioselectivity. The use of ketones bearing electron-neutral or weakly electron-donating groups (negative Hammett sigma constants)^19^ were also tolerated, with **29** and **30** obtained with good to excellent diastereo- and enantioselectivity, albeit in slightly reduced yield (∼50%). The introduction of a more strongly electron-donating *para*-methyl substituent led to only trace amounts of β-lactone **31** in the perfluorobutyl-substituted ketone series. In contrast, using the analogous trifluoromethyl-substituted ketone led to the formation of β-lactone **32** in high yield (78%) and with excellent enantioselectivity (99 : 1 er). The system was further challenged by the introduction of a highly electron-donating *para*-methoxy group. β-Lactone **33** was produced with excellent stereoselectivity (>95 : 5 dr, 99 : 1 er), albeit in a relatively low yield (22%), which was attributable to lower conversion of the ketone starting material. The use of heteroaromatic ketones was also reasonably successful, with thienyl-substituted β-lactone **34** obtained in 43% yield, with high diastereoselectivity, but reduced enantioselectivity (>95 : 5 dr, 87 : 13 er). Finally, the reaction scope was extended to include alkyl-substituted ketones, with the use of trifluoroacetone providing access to β-lactone **35** in an excellent 91% yield and with high diastereo- and enantioselectivity (95 : 5 dr, 93 : 7 er).

#### 2.2.2. Variation of anhydride

The scope of anhydrides applicable in this protocol was first probed through reactions with perfluorobutyl-substituted ketone **18** (Table 3). Anhydrides bearing electron-donating and electron-withdrawing substituents in the *para*-position were tolerated, with β-lactones **36–40** all obtained with excellent diastereo- and enantioselectivity. Anhydrides bearing electron-donating substituents provided the corresponding β-lactones in high yield. On the other hand, incorporation of electron-withdrawing groups resulted in a drop in yield. This effect was most pronounced for *para*-trifluoromethyl substitution, with β-lactone **40** obtained in only 12% yield. The substitution pattern of the anhydride was next studied, with both *meta*- and *ortho*-tolyl-substituted β-lactones **41** and **42** obtained in high yield and with excellent diastereo- and enantioselectivity. This is particularly notable for the sterically-hindered *ortho*-tolyl-substituted example **42**, as this substitution pattern has frequently been reported to provide lower levels of stereoselectivity in related isothiourea-catalysed methods.11a,^20^ Heteroaromatic anhydrides were also readily applicable, with thienyl- and indolyl-substituted β-lactones **43** and **44** obtained in good to excellent yield and with excellent diastereo- and enantioselectivity. Beyond aryl acetic acid derivatives, the use of (*E*)-pent-3-enoic anhydride provided 3-alkenyl-substituted β-lactone **45** in good yield, but with slightly lower levels of diastereo- and enantiocontrol. In addition, phenylthioacetic anhydride was tolerated, giving β-lactone **46** in relatively low yield and moderate enantioenrichment but with excellent diastereoselectivity.

The generality of the procedure was further probed by applying a subset of anhydrides for the formation of β-lactones using perfluoroethyl-substituted ketone **19** (Table 4) and trifluoromethyl-substituted ketone **20** (Table 5). The results obtained using perfluoroethyl-substituted ketone **19** feature similar trends as when using perfluorobutyl-substituted ketone **18**. β-Lactones **47–51** were obtained with excellent diastereo- and enantioselectivity when using arylacetic anhydride derivatives,^21^ and slightly reduced stereocontrol when using (*E*)-pent-3-enoic anhydride (**52**). In all cases excellent yields were obtained, with the exception of an electron-withdrawing group on the aryl acetic anhydride, which again resulted in a slight drop in yield (50 : 65%).

The same set of anhydrides was also applied in the synthesis of β-lactones with trifluoromethyl-substituted ketone **20** at –78 °C (Table 5). All 3-aryl-substituted β-lactones **53–56** were obtained with similar diastereoselectivity (∼90 : 10 dr),^22^ however alkenyl-substituted β-lactone **58** was obtained with relatively low diastereocontrol (69 : 31 dr). In this series, high to excellent enantioselectivities were obtained in each case, including for the formation of 3-alkenyl-substituted β-lactone **58**, which is in contrast to the other series in which a drop in enantioselectivity was observed when using this anhydride (see Tables 3 and 4, **45** and **52**).

**Table 3:** Scope: variation of anhydride using perfluorobutyl-substituted ketone **18**^*a*^

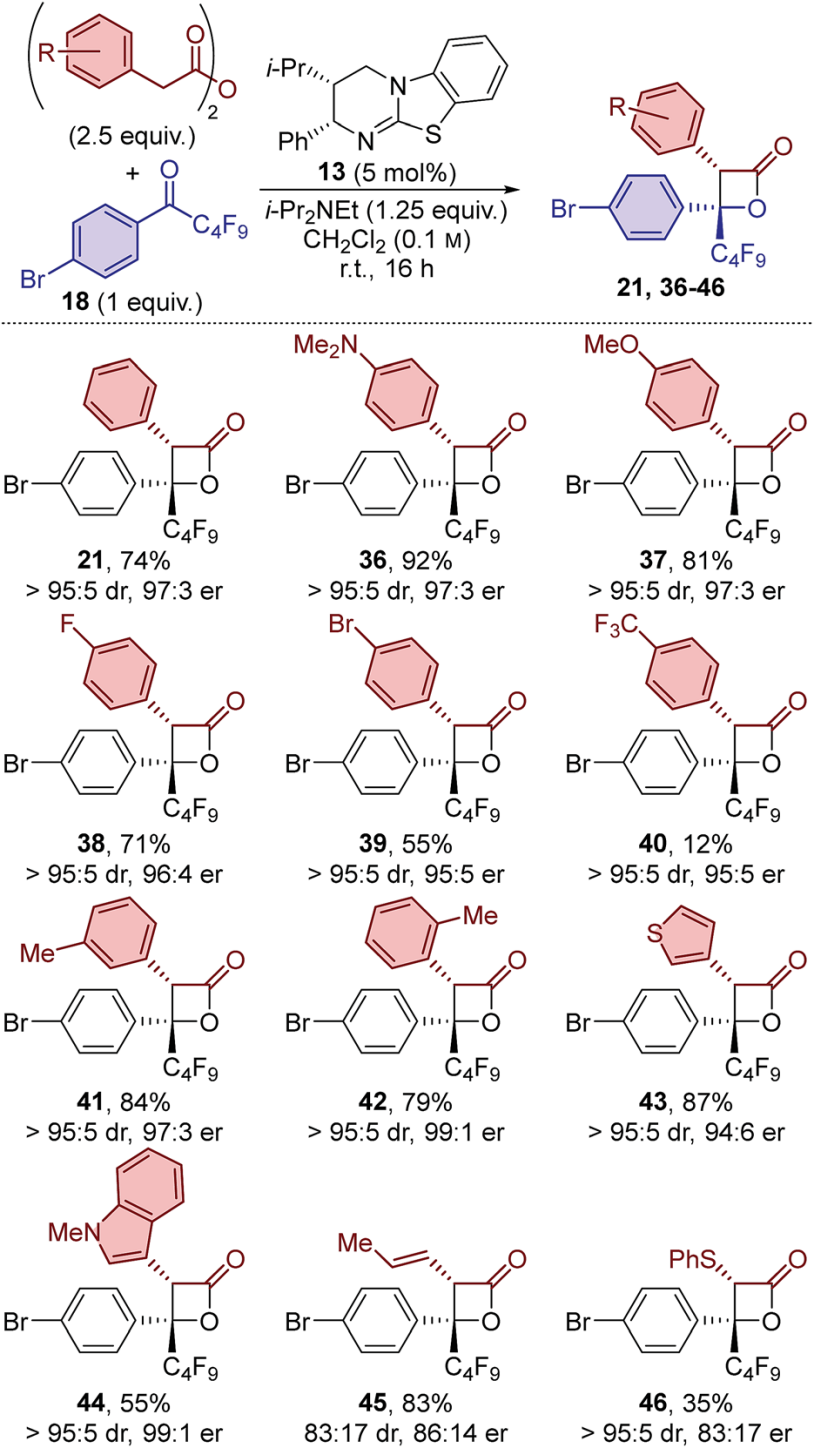

^*a*^dr determined by ^1^H NMR spectroscopic analysis of the crude reaction product mixture. er determined by HPLC analysis using a chiral support.

**Table 4:** Scope: variation of anhydride using perfluoroethyl-substituted ketone **19**^*a*^

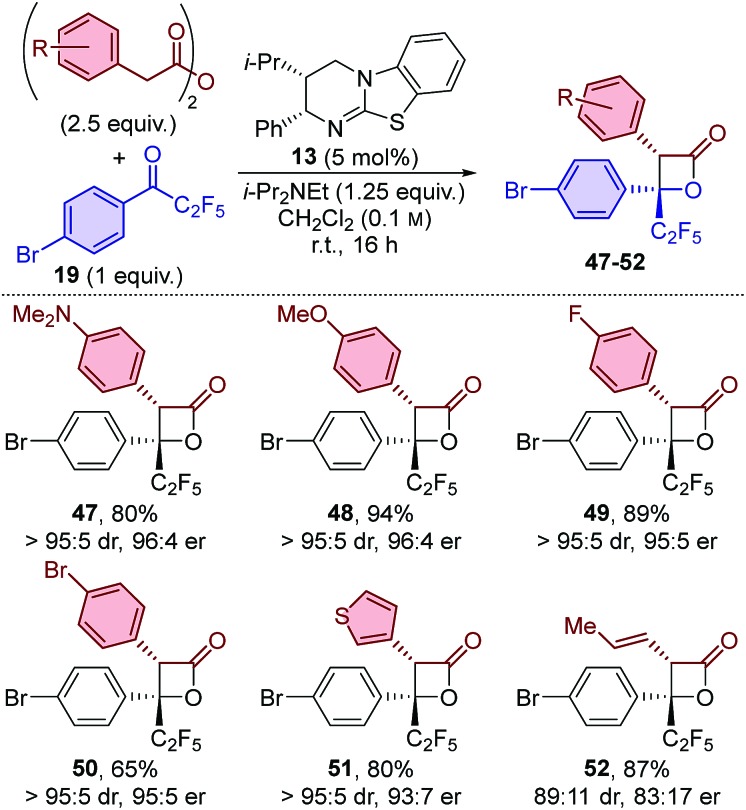

^*a*^dr determined by ^1^H NMR spectroscopic analysis of the crude reaction product mixture. er determined by HPLC analysis using a chiral support.

**Table 5:** Scope: variation of anhydride using trifluoromethyl-substituted ketone **20**^*a*^

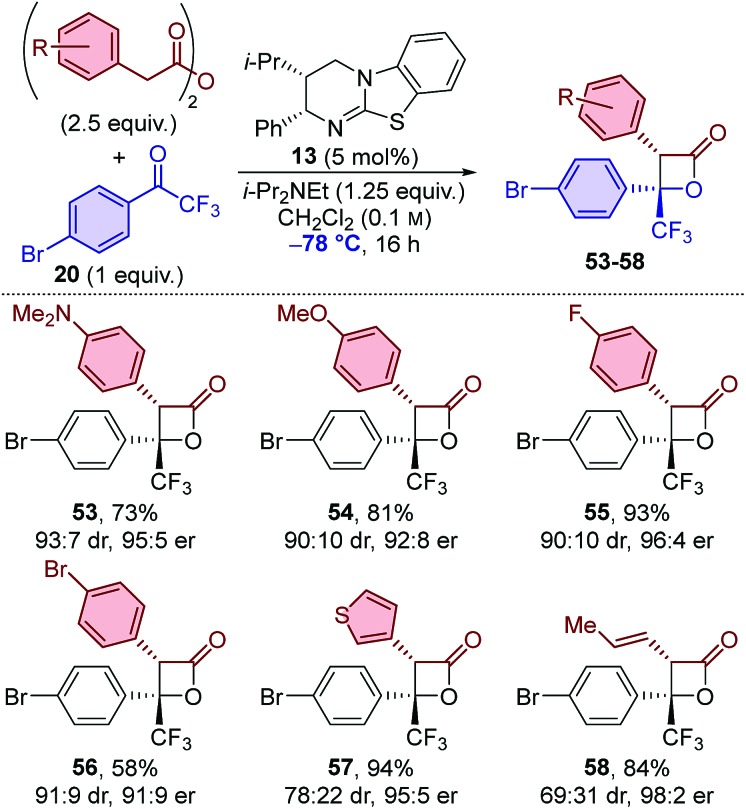

^*a*^dr determined by ^1^H NMR spectroscopic analysis of the crude reaction product mixture. er determined by HPLC analysis using a chiral support.

#### 2.2.3. Product derivatisation

**Scheme 4:**
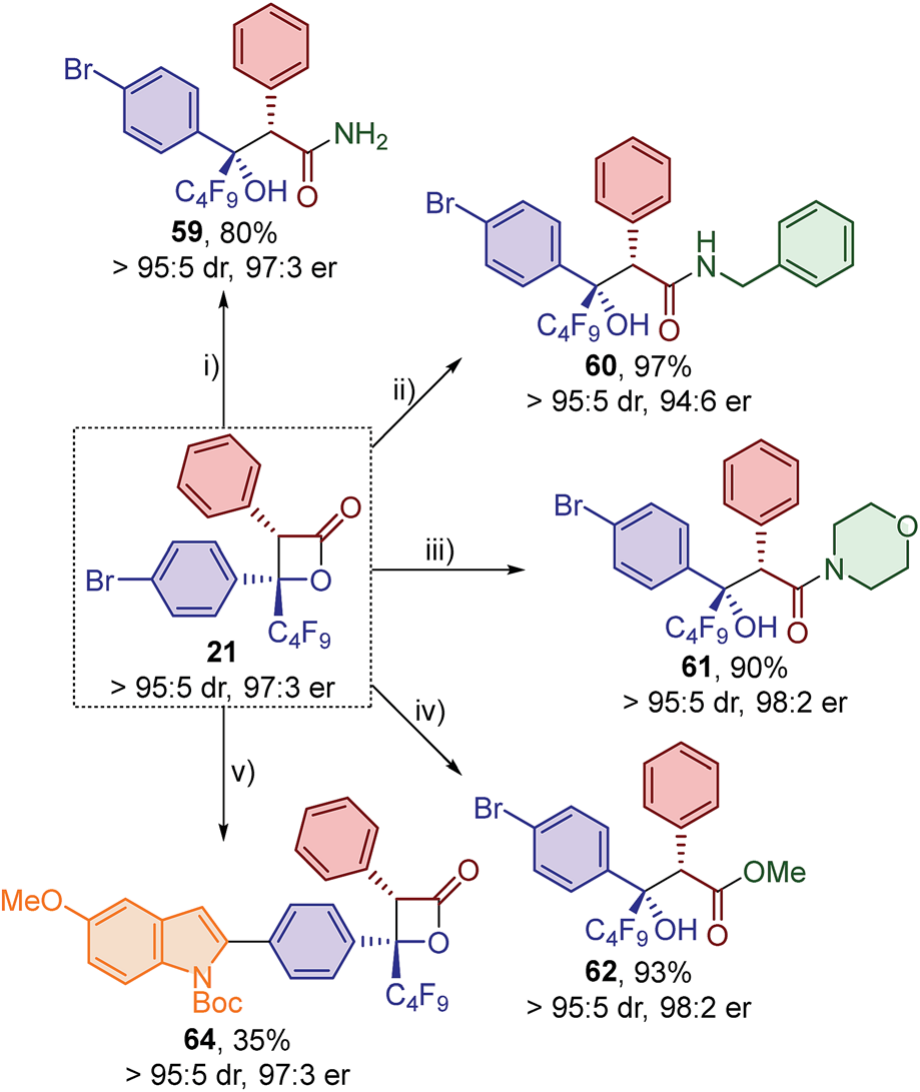
Derivatisations of β-lactone **21**. Conditions: (i) **21** (1 equiv.), NH_3_ (5 equiv.), 1,4-dioxane (0.1 M), r.t., 16 h; (ii) **21** (1 equiv.), BnNH_2_ (5 equiv.), CH_2_Cl_2_ (0.1 M), r.t., 16 h; (iii) **21** (1 equiv.), morpholine (5 equiv.), CH_2_Cl_2_ (0.1 M), r.t., 16 h; (iv) **21** (1 equiv.), NaOMe (5 equiv.), MeOH/CH_2_Cl_2_ (1 : 1, 0.1 M), –78 °C, 2 h; (v) **21** (1 equiv.), *N*-Boc-5-methoxyindole-2-boronic acid **63** (1 equiv.), Pd(PPh_3_)_4_ (0.1 equiv.), Na_2_CO_3_ (3 equiv.), DMF/H_2_O (9 : 1, 0.06 M), 85 °C, 4 h.

The synthetic utility of the β-lactone products was investigated through a series of elaborations to introduce further functionality (Scheme 4). The addition of ammonia to β-lactone **21** in either water or ethanol was unsuccessful, with only the formation of perfluorobutyl-substituted ketone **18** and phenylacetic acid observed. This is consistent with the operation of an unwanted formal retro-[2 + 2] process under these conditions. This problem was overcome by using ammonia in 1,4-dioxane, with primary amide **59** obtained in 80% yield and with no erosion of diastereo- or enantiopurity. The addition of primary and secondary amines was also successful, with secondary and tertiary amides **60** and **61** obtained in high yield and excellent diastereo- and enantiopurity.^23^ The synthesis of esters was next investigated. The room temperature addition of methanol, in the presence of substoichiometric DMAP, provided ester **62** in low yield (22%) and eroded enantiopurity (89 : 11 er). The addition of sodium methoxide in dichloromethane at –78 °C however allowed access to ester **62** in 93% yield with no loss in enantiopurity (98 : 2 er). Finally, the arylbromide functionality of β-lactone **21** was exploited for a Suzuki–Miyaura cross-coupling with 2-indolylboronic acid derivative **63** to give β-lactone **64** with no loss in diastereo- or enantiopurity. Although β-lactone **64** was only isolated in 35% yield, this derivatisation demonstrates the appreciable stability of these β-lactones under basic aqueous conditions, even at high reaction temperatures.

Finally, derivatisation of the β-lactone products to give perfluoroalkyl-substituted oxetanes was investigated (Table 6). Oxetanes are of significant interest in medicinal chemistry as bioisosteres for *gem*-dimethyl and carbonyl groups, and also in synthetic chemistry as versatile reactive intermediates.^24^ Reduction of the β-lactones using i-Bu_2_AlH (DIBAL) at –78 °C provided access to diols **65–69** in moderate yield and with no loss in diastereo- or enantiopurity. Treatment of these diols with sodium hydride, followed by 2,4,6-triisopropylbenzenesulfonyl chloride, provided perfluoroalkyl-substituted oxetanes **70–74** in excellent yield and in highly enantioenriched form. The protocol proved to be general with comparable results obtained when using β-lactones bearing perfluorobutyl, perfluoroethyl or trifluoromethyl substituents and various functionalised aromatic groups.

**Table 6:** Synthesis of perfluoroalkyl-substituted diols and oxetanes^*a*^

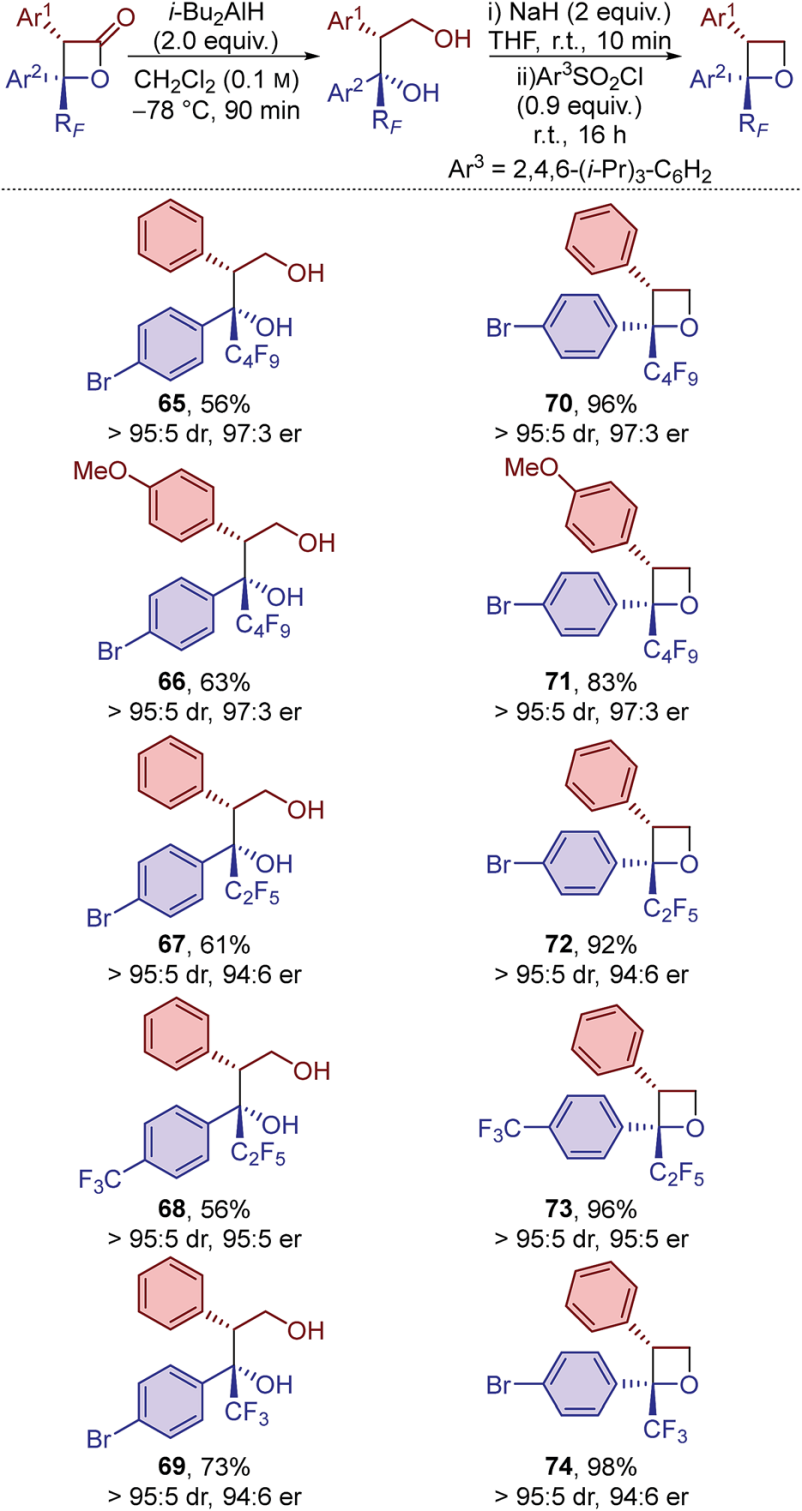

^*a*^dr determined by ^1^H NMR spectroscopic analysis of the crude reaction product mixture. er determined by HPLC analysis using a chiral support.

### 2.3. Mechanism

#### 2.3.1. Concerted *vs.* stepwise lactonisation

Considerable attention has been given to the mechanistic question of *concerted versus stepwise* reaction pathways for formal [2 + 2] cycloaddition reactions involving ketenes.^25^ However, significantly fewer mechanistic studies have been disclosed for catalytic reactions of non-ketene derived reactive intermediates.4e,^26^–^28^ Paddon-Row and Lupton have reported the synthesis of fused β-lactones using NHC catalysis, with computations suggesting β-lactone formation takes place *via* a stepwise aldol–lactonisation pathway.^26^ In a related mechanistic investigation, we explored an NHC-catalysed dynamic kinetic resolution/β-lactonisation of α-substituted-β-ketoesters. β-Lactone ring formation was found to proceed through either a concerted asynchronous [2 + 2] cycloaddition (major product) or an NHC-spiro intermediate (minor products), with an alternative stepwise aldol–lactonisation pathway not located on the potential energy surface (PES).^27^ Recent computational studies by Hare and Tantillo on a Rh(ii)-catalysed C–H insertion/β-lactonisation reaction revealed an even more complex mechanistic scenario in which a post-transition state bifurcation contributes to product selectivity.^28^

**Fig. 1:**
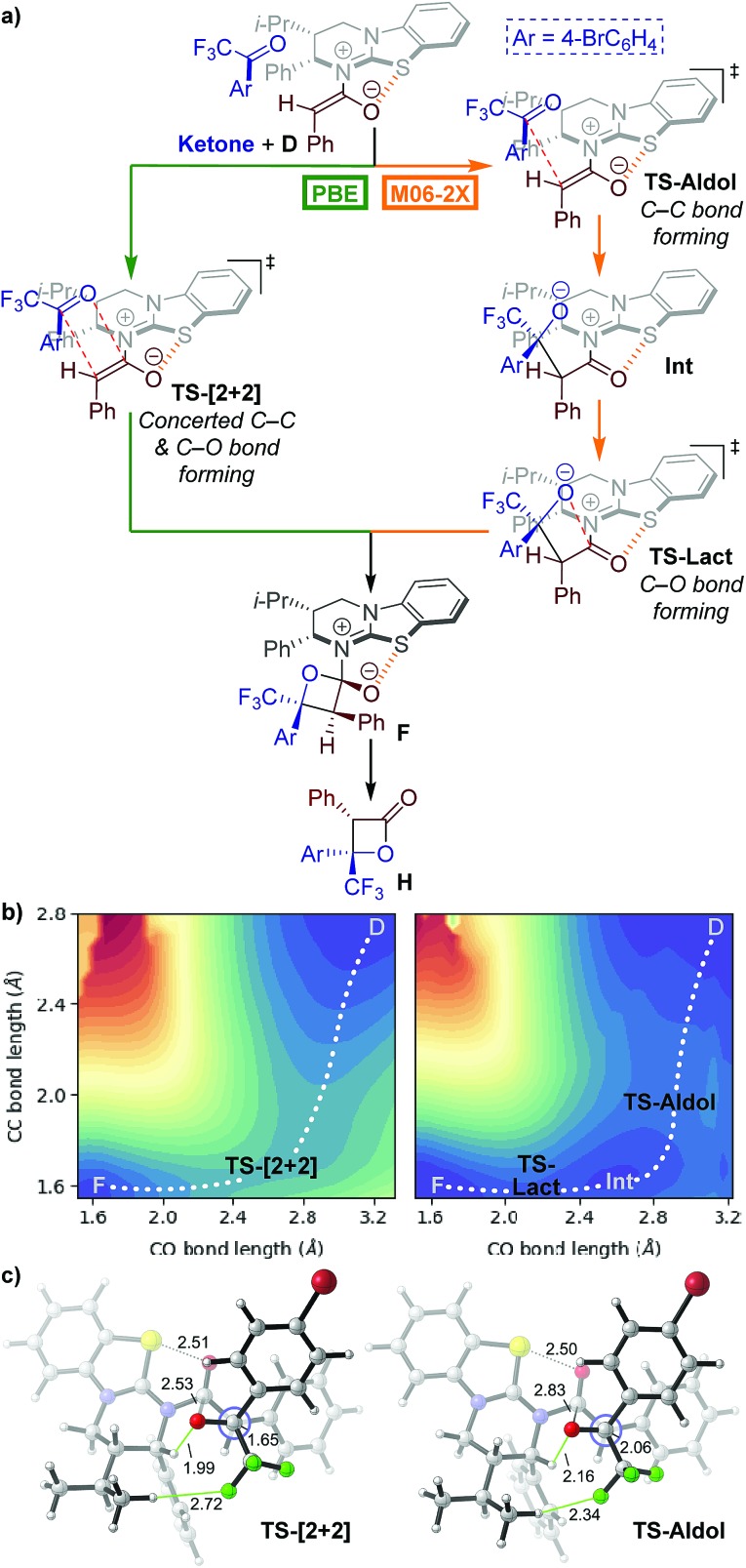
(a) Possible mechanistic pathways for the formation of intermediate **F**; Ar = 4-bromophenyl. (b) Potential energy surface (PES) contours generated with parallel distance scans in the vicinity of the β-lactone forming transition state bond lengths. Blue regions are lower in energy while red regions are higher in energy. Structure **D** is not a stationary point on the PES but represents a relatively lower energy acylated catalyst–ketone complex directly “downhill” from the first saddle point. Contour images generated and rendered with Matplotlib.^34^ (c) Computed *concerted asynchronous* [2 + 2] and *stepwise* aldol TSs using PBE and M06-2X, respectively. The Newman projection looks down the forming C–C bond. Distances are in Ångströms. Structure images made with CYLView.^35^

Our initial mechanistic objective was to identify the nature of the potential energy surface (PES) for the lactonisation step of the isothiourea-catalysed formation of perfluoroalkyl-substituted β-lactones.^15^ Phenylacetic anhydride **17**, 4′-bromo-2,2,2-trifluoroacetophenone **20** and HyperBTM **13** were used as model substrates to examine the PES. We began by computing transition states (TSs) connecting HyperBTM-enolate complex **D** to product–catalyst complex **F** using two widely applied density functional theory (DFT) methods, M06-2X^29^ and PBE^30^ (Fig. 1a).

Surprisingly, the two DFT methods revealed different reaction mechanisms. M06-2X predicted a stepwise process in which **TS-Aldol** (Fig. 1) was located where only the β-lactone C–C bond was formed. A subsequent ring-closing lactonisation TS (**TS-Lact**) was located that forms the C–O bond, ultimately giving the product–catalyst complex **F**. Attempts to locate a concerted TS with M06-2X were unsuccessful. Interestingly, PBE gave the concerted asynchronous **TS-[2 + 2]**,^31^,^32^ and no stepwise transition structures could be located.

We computed the respective PESs around the located transition structures (Fig. 1b) in order to clarify the differences between the predicted mechanisms for each method. The PESs were computed by fixing the C–C bond distance and varying the C–O bond distance of the forming β-lactone. The M06-2X surface indeed revealed a stepwise process involving two first-order saddle-points (**TS-Aldol** and **TS-Lact**) and a zwitterionic intermediate (**Int**). In contrast, the PBE surface showed a single first-order saddle point connecting the enolate–ketone complex **D** directly to the product–catalyst complex **F**. As these two popular theoretical methods revealed differing mechanistic pathways, we turned to the use of coordinated kinetic isotope effect experiments and computations to elucidate the active mechanism.^33^

#### 2.3.2. Kinetic isotope effects at natural abundance

**Scheme 5:**
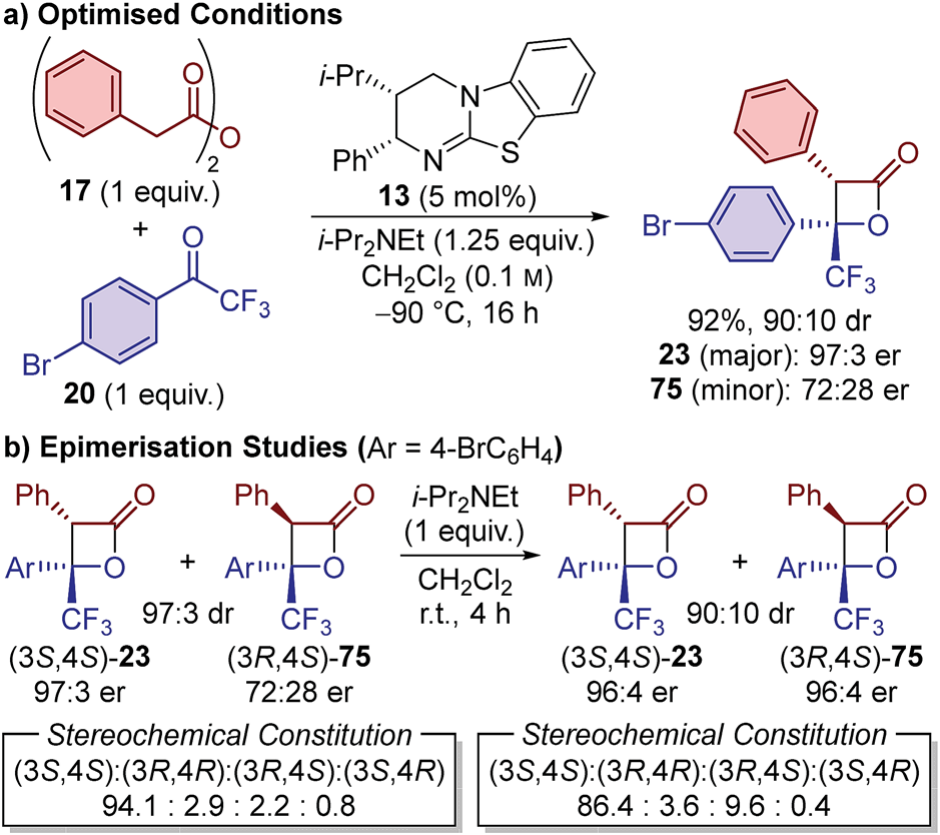
Reaction optimisation for KIE at natural abundance analysis.

With computed TS geometries for both the stepwise and concerted pathways in hand, we sought to investigate the formation of the β-lactone using ^13^C kinetic isotope effects (KIEs) at natural abundance.25d,e,^36^ This method is most conveniently applied for reactions which are conducted with a 1 : 1 ratio of reagents and give just a single product (as a single stereoisomer). The formal cycloaddition using phenylacetic anhydride **17** and 4′-bromo-2,2,2-trifluoroacetophenone **20** was therefore further optimised to fulfil these criteria. By conducting the reaction at –90 °C in anhydrous CH_2_Cl_2_ under inert conditions, the reaction stoichiometry could be reduced to 1 : 1, with β-lactone **23** isolated in 92% yield and 90 : 10 dr (Scheme 5a). Notably, the major diastereoisomer **23** was isolated in highly enantioenriched form (97 : 3 er), while the minor diastereoisomer **75** was isolated in a 72 : 28 er. To investigate the origin of this observation, a 97 : 3 sample of β-lactones **23** (97 : 3 er) and **75** (72 : 28 er) was treated with i-Pr_2_NEt in CH_2_Cl_2_ (Scheme 5b). After 2 h, the diastereomeric ratio had readjusted to 90 : 10 (96 : 4 er for both diastereoisomers), with no further change observed upon extended exposure times. This is consistent with base-mediated epimerisation at C(3), and allowed the configuration of the minor diastereoisomer **75** isolated from the catalytic reaction to be unambiguously assigned as (3*R*,4*S*).^37^ This stereoisomer is most likely generated by C(3)-epimerisation of the major (3*S*,4*S*)-stereoisomer, and indicates that the kinetically-derived diastereomeric ratio in the catalytic reaction is likely to be significantly higher than 90 : 10. The optimised system is therefore proposed to provide ∼90–95% selectivity for initial generation of the (3*S*,4*S*)-stereoisomer, which was considered to be sufficient for reliable assessment of the ^13^C KIEs at natural abundance.

**Fig. 2:**
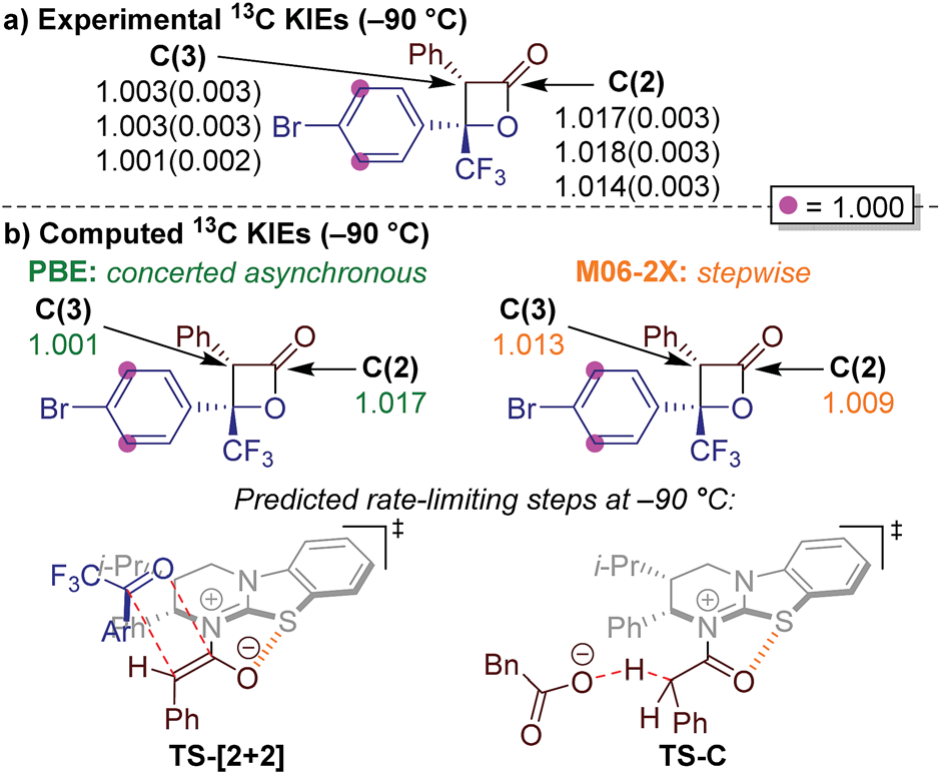
Experimentally and computationally-determined ^13^C KIEs. (a) ^13^C KIEs measured at –90 °C in three independent reactions, with standard deviations from five measurements given in parentheses; (b) computed KIEs are reported with the Bigeleisen–Mayer method with frequencies derived from M06-2X and PBE methods at –90 °C. M06-2X predicts deprotonation as rate-limiting, therefore the reported KIEs are derived from **TS-C**. PBE predicts cycloaddition as rate-limiting, and the associated KIEs are derived from **TS-[2 + 2]**.

Determination of ^13^C KIEs for each carbon of the β-lactone **23** ring was approached by performing reactions to low conversion (∼5%) and analysing the ^13^C isotopic composition of β-lactone product **23**.^38^,^39^ The isotopic composition of each sample was compared to a sample of β-lactone **23** obtained from a reaction taken to completion. In each case, the signal from the *meta*-carbons of the 4-bromophenyl (Ar) substituent was used as an internal standard, with clear baseline separation and the assumption of negligible isotope effect at this position. Unfortunately, due to incomplete chromatographic separation of the diastereoisomers, the ^13^C KIE for C(4) of β-lactone **23** could not be reliably measured due to partial overlap of the signals arising from each diastereoisomer, which appear as quartets (*J* = 33 Hz) from ^13^C–^19^F coupling with the CF_3_ substituent. ^13^C KIEs were therefore only calculated for C(2) and C(3) (Fig. 2a). Three independent experiments were performed, with each sample analysed five times by quantitative ^13^C NMR spectroscopy.^15^ Computed KIEs (Fig. 2b) are reported using the Bigeleisen–Mayer method^40^ with the *meta*-carbons of the 4-bromophenyl substituent set to unity and thermal corrections computed at –90 °C to match experimental conditions (see ESI†). Computation of all predicted KIEs were automated by use of the *Onyx* isotope effect program.^41^

The experimental KIEs at natural abundance reveal a small, normal isotope effect at C(2), and essentially no isotope effect at C(3) (Fig. 2a).^42^ For PBE/6-31G(d),^43^ the predicted KIEs at C(2) and C(3) of the rate-limiting concerted asynchronous **TS-[2 + 2]** are in excellent agreement with the experimental values. Interestingly, at –90 °C, M06-2X predicted deprotonation (**TS-C**, Fig. 2b right) as the rate-limiting step, whereas at room temperature, the **TS-Aldol** was rate-limiting (see ESI†). It is important to note that computations using the M06-2X level of theory did not match the experimental KIEs, regardless of the rate-limiting step. Based on these results, we propose a concerted asynchronous [2 + 2] cycloaddition as rate-limiting in the HyperBTM-catalysed synthesis of perfluoroalkyl-substituted β-lactones reported in this manuscript.

#### 2.3.3. Computed catalytic cycle

The complete catalytic cycle was computed with phenylacetic anhydride **17**, 4′-bromo-2,2,2-trifluoroacetophenone **20** and HyperBTM **13**. Exhaustive manual conformational searches were performed to locate all pertinent conformations. Geometries were computed using the PBE/6-31G(d) and M06-2X/6-31G(d) levels of theory as implemented in Gaussian 09.^15^,^44^,^45^ Vibrational frequencies and thermal corrections to the Gibbs free energy were calculated at both 25 °C and –90 °C to match the initial reaction optimisation and KIE experiments, respectively. All transition states were verified with intrinsic reaction coordinate (IRC) calculations and single-point energy refinements were computed using the larger 6-311++G(2df,p) basis set with the same DFT method.^46^ Implicit solvation was included using the polarisable continuum model (PCM)^47^ for dichloromethane in both the geometry optimisation and single-point energy refinement.

**Fig. 3:**
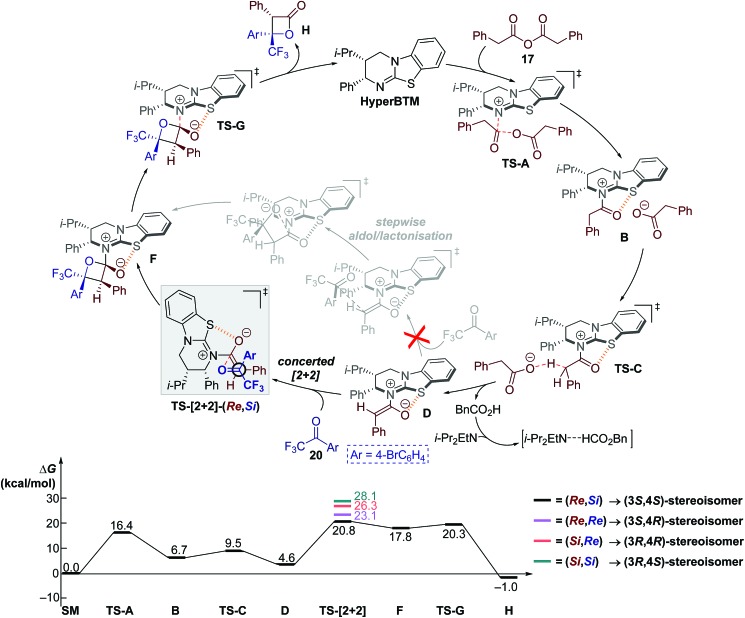
(Top) Proposed catalytic cycle; Ar = 4-bromophenyl. (Bottom) Computed reaction coordinate using PBE/6-311++G(2df,p)/PCM(DCM)//PBE/6-31G(d)/PCM(DCM) with thermal corrections computed at –90 °C.

The computed catalytic cycle with PBE at –90 °C is summarised in Fig. 3, as this method correctly predicted the experimental KIE results (*vide supra*). Addition of HyperBTM to phenylacetic anhydride **17** proceeds, *via***TS-A** (Δ*G*^‡^ = 16.4 kcal mol^–1^), to give the acylated-HyperBTM and phenylacetate ion pair **B** (Δ*G* = 6.7 kcal mol^–1^), without location of a tetrahedral intermediate.^48^ Deprotonation of the acyl group by phenylacetate (**TS-C**, Δ*G*^‡^ = 9.5 kcal mol^–1^) yields (*Z*)-enolate **D** (Δ*G* = 4.6 kcal mol^–1^).^49^ Intermediate **D** is the key reactive species, with subsequent rate-limiting concerted asynchronous [2 + 2] cycloaddition with ketone **20** (**TS-[2 + 2]-(Re,Si)**, Δ*G*^‡^ = 20.8 kcal mol^–1^) leading to tetrahedral intermediate **F** (Δ*G* = 17.8 kcal mol^–1^). Catalyst turnover occurs through cleavage of the catalyst–lactone N–C bond, releasing product and initiating a new catalytic cycle (**TS-G**, Δ*G*^‡^ = 20.3 kcal mol^–1^). All acylated HyperBTM intermediates and transition states in the catalytic cycle exhibit a stabilising and rigidifying 1,5-S···O interaction^50^,^51^ (dashed orange line, Fig. 3).

Computations overpredict both enantioselectivity 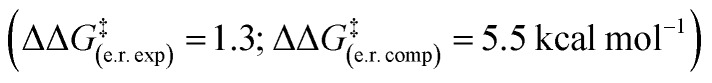 and diastereoselectivity 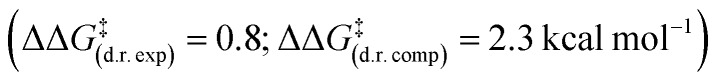 compared to experiments. These deviations may arise in part from the previously discussed epimerisation at the C(3) position. Alternative theoretical methods (*e.g.* B3LYP) and reaction mechanisms that would lead to the formation of racemic β-lactone (*e.g.* cycloaddition between *in situ* generated ketene and ketone **20**) were investigated. However, all alternative mechanisms were significantly disfavoured (Δ*G*^‡^ > 30.7 kcal mol^–1^) compared to the HyperBTM-catalysed concerted asynchronous [2 + 2] mechanism, and other quantum mechanical methods gave similar predicted selectivities.^15^

## Conclusions

In conclusion, the isothiourea-catalysed reaction between symmetric anhydrides and perfluoroalkyl ketones (R_F_ = CF_3_, C_2_F_5_, C_4_F_9_) has been demonstrated for the enantioselective generation of a range of isolable perfluoroalkyl-substituted β-lactones with excellent stereocontrol (37 examples, up to >95 : 5 dr and >99 : 1 er). Derivatisation of the β-lactones was achieved through ring-opening processes, in addition to conversion to perfluoroalkyl-substituted oxetanes through a simple two-step process. Quantum mechanical computations were used to elucidate the mechanism of this process. Interestingly, two widely applied density functional theory (DFT) methods gave different mechanistic pathways. PESs were used to verify the opposing mechanisms, wherein M06-2X predicted a stepwise aldol/lactonisation process and PBE predicted a concerted asynchronous [2 + 2] process. Experimental and computed ^13^C natural abundance KIE studies were juxtaposed to elucidate the active mechanism. The experimental and PBE-predicted KIE values were in excellent agreement, suggesting the reaction proceeds through a concerted asynchronous [2 + 2] mechanism.^52^

## Conflicts of interest

There are no conflicts to declare.

## Author contributions

D.-J. B. A., M. D. G., P. E.-R., P. R. and T. H. W. contributed to the synthetic work. A. M. Z. S. obtained X-ray crystal structures. A. C. B., D. M. W. and B. G. Y. performed the computational work. M. D. G., A. C. B. and D. M. W. wrote the manuscript. A. D. S. and P. H. Y. C. supervised the project and the writing of the manuscript.

## Supplementary Material

Supplementary information

Crystal structure data
