# Supplementary material for: Catalytic enantioselective synthesis of perfluoroalkyl-substituted β-lactones via a concerted asynchronous [2 + 2] cycloaddition: a synthetic and computational study
Source: Chem Sci. 2019 Apr 29;10(24):6162–73. doi: 10.1039/c9sc00390h (PMC6585878; doi:10.1039/c9sc00390h)
Supplement: Supplementary file 1 [file SC-010-C9SC00390H-s001.pdf]

Electronic Supplementary Material (ESI) for Chemical Science.
This journal is © The Royal Society of Chemistry 2019

Catalytic enantioselective synthesis of perfluoroalkyl-substituted β-lactones *via* a concerted asynchronous [2+2] cycloaddition: A synthetic and computational study

Diego-Javier Barrios Antúnez,[a] Mark D. Greenhalgh,[a] Alexander C. Brueckner,[b] Daniel M. Walden,[b] Pilar Elías-Rodríguez,[a] Patrick Roberts,[a] Benjamin G. Young,[c] Thomas H. West,[a] Alexandra M. Z. Slawin,[a] Paul Ha-Yeon Cheong*[b] and Andrew D. Smith*[a]

[a] EaStCHEM, School of Chemistry, University of St Andrews, North Haugh, St Andrews, KY16 9ST, U.K.
[b] Department of Chemistry, Oregon State University, 153 Gilbert Hall, Corvallis, Oregon 97333, USA.
[c] Department of Chemistry, Physics, and Engineering, Biola University, 315 Lim Center, La Mirada, California 90639, USA.
* E-mails: ads10@st-andrews.ac.uk; cheongh@oregonstate.edu

Experimental Supporting Information

Table of Contents

General Experimental S2
General Procedures S4
Synthesis of Homoanhydrides S6
Synthesis of Perfluorinated Ketones S9
Synthesis of β-lactones S13
Product Derivatisations S37
Epimerisation Studies S46
Thermal Stability of β-Lactones **21**–**23** S51
Determination of ^{13}C KIEs at Natural Abundance S52
X-Ray Crystallographic Details S56
Computational Details and Discussion S57
Computed Geometries and Energies S65
- Concerted [2+2] Mechanism Coordinate Diagram: PBE S65
- Stepwise Mechanism Coordinate Diagram: M06-2X S77
- Concerted [2+2] Mechanism Additional Structures: PBE S92
- Stepwise Mechanism Additional Structures: M06-2X S107
- Alternative Mechanisms: PBE S127

NMR Spectra (^{1}H, ^{13}C, ^{19}F) S135
HPLC Spectra S221
References S273

General Experimental

Reactions involving moisture sensitive reagents were carried out in flame-dried glassware under a nitrogen atmosphere using standard vacuum line techniques, and using anhydrous solvents. Anhydrous solvents (Et_2O, CH_2Cl_2 and toluene) were obtained from an anhydrous solvent system (purified using an alumina column, Mbraun SPS-800). All other reactions were performed in standard glassware with no precautions to exclude air or moisture. Solvents and commercial reagents were used as supplied without further purification unless otherwise stated.

Room temperature (r.t.) refers to 20-25 °C. Temperatures of 0 °C and −78 °C were obtained using ice/water and CO_2(s)/acetone baths respectively. −90 °C was obtained using an immersion cooler (HAAKE EK 90). Reactions involving heating were performed using a DrySyn block and a contact thermocouple.

In vacuo refers to the use either a Büchi Rotavapor R-200 with a Büchi V-491 heating bath and Büchi V-800 vacuum controller; a Büchi Rotavapor R-210 with a Büchi V-491 heating bath and Büchi V-850 vacuum controller; a Heidolph Laborota 4001 with vacuum controller; an IKA RV10 rotary evaporator with an IKA HB10 heating bath and ILMVAC vacuum controller; or an IKA RV10 rotary evaporator with an IKA HB10 heating bath and Vacuubrand CVC3000 vacuum controller. Rotary evaporator condensers are fitted to Julabo FL601 Recirculating Coolers filled with ethylene glycol set to –5 °C.

Analytical thin layer chromatography was performed on pre-coated aluminium plates (Kieselgel 60 F_{254} silica). TLC visualisation was carried out with ultraviolet light (254 nm), followed by staining with a 1% aqueous $KMnO_4$ solution. Flash column chromatography was performed on Kieselgel 60 silica in the solvent system stated. Automated chromatography was performed on a Biotage Isolera Four running Biotage OS578 with a UV/Vis detector using the method stated and cartridges filled with Kieselgel 60 silica.

Melting points were recorded on an Electrothermal 9100 melting point apparatus and are uncorrected.

Optical rotations were measured on a Perkin Elmer Precisly/Model-341 polarimeter operating at the sodium D line with a 100 mm path cell at 20 °C.

HPLC analyses were obtained using either a Shimadzu HPLC consisting of a DGU-20A5 degassing unit, LC-20AT liquid chromatography pump, SIL-20AHT autosampler, CMB-20A communications bus module, SPD-M20A diode array detector and a CTO-20A column oven; or a Shimadzu HPLC consisting of a DGU-20A5R degassing unit, LC-20AD liquid chromatography pump, SIL-20AHT autosampler, SPD-20A UV/Vis detector and a CTO-20A column oven. Separation was achieved using DAICEL CHIRALCEL OD-H columns or DAICEL CHIRALPAK AD-H, AS-H and IC columns using the method stated. HPLC traces of enantiomerically enriched compounds were compared with authentic racemic spectra.

^{1}H, ^{13}C{^{1}H} and ^{19}F{^{1}H} nuclear magnetic resonance (NMR) spectra were acquired on either a Bruker Avance II 400 (^{1}H 400 MHz; ^{13}C{^{1}H} 101 MHz, ^{19}F{^{1}H} 376 MHz) or a Bruker Avance II 500 (^{1}H 500 MHz; ^{13}C{^{1}H} 126 MHz, ^{19}F{^{1}H} 471 MHz) spectrometer at ambient temperature in the deuterated solvent stated. All chemical shifts are quoted in parts per million (ppm) and referenced to the residual solvent peak. All coupling constants, *J*, are quoted in Hz. Multiplicities are indicated by: s (singlet), d (doublet), t (triplet), q (quartet), dd (doublet of doublets), dt (doublet of triplets), dq (doublet of quartets), tt (triplet of triplets), ddd (doublet of doublet of doublets) and m (multiplet). The abbreviation Ar is used to denote aromatic, Ph to denote phenyl, br to denote broad and app to denote apparent. NMR peak assignments were confirmed using 2D ^{1}H–^{13}C heteronuclear single quantum coherence (HSQC) and 2D ^{1}H–^{13}C heteronuclear multiple-bond correlation spectroscopy (HMBC) where necessary.

Infrared spectra were recorded on a Shimadzu IRAffinity-1 Fourier transform IR spectrophotometer fitted with a Specac Quest ATR accessory (diamond puck). Spectra were recorded of either thin films or solids, with characteristic absorption wavenumbers (ν_{max}) reported in cm^{-1}.

Mass spectrometry (*m/z*) data were acquired by electrospray ionization (ESI), atmospheric pressure chemical ionization (APCI) or nanospray ionization (NSI) either at the University of St Andrews or the EPSRC National Mass Spectrometry Facility, Swansea.

General Procedures

General Procedure A

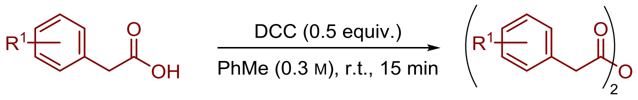

N,*N*'-Dicyclohexylcarbodiimide (DCC) (0.5 equiv.) was added to a solution of the appropriate carboxylic acid (1 equiv.) in toluene (0.3 M) at r.t., and the reaction mixture was allowed to stir for 15 min. The suspension was filtered through Celite®, which was washed with extra toluene, and the filtrate was concentrated *in vacuo* to give the crude product, which was either used without further purification, or recrystallised from hot Et_2O.

General Procedure B

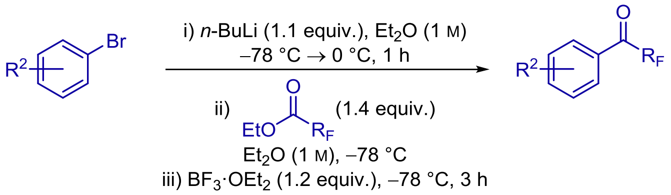

Following a literature procedure,[1] *n*-butyl lithium (2.5 M in hexane, 1.1 equiv.) was added dropwise to a solution of the suitable bromoarene (1 equiv.) in anhydrous Et_2O (1 M) at −78 °C under an inert atmosphere. The reaction mixture was then allowed to warm to 0 °C and allowed to stir for 1 h. This solution was then transferred by cannula into a flask containing the suitable polyfluoroester (1.4 equiv.) in Et_2O (1 M) at –78 °C under an inert atmosphere. $BF_3{\cdot}OEt_2$ (1.2 equiv.) was then added and the mixture was allowed to stir at –78 °C for 3 h. Saturated aqueous NH_4Cl was added and the biphasic mixture was allowed to warm to r.t. The layers were separated, the aqueous layer was extracted with Et_2O, and the combined organic layers were washed with brine, dried ($MgSO_4$) and concentrated *in vacuo*. The residue was purified as described to afford the desired ketone.

General Procedure C

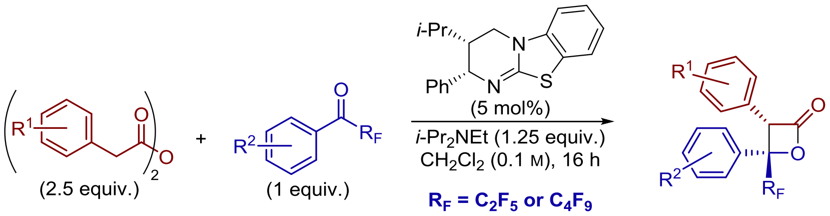

(2*S*,3*R*)-HyperBTM (5 mol%) and *i*-Pr_2NEt (1.25 equiv.) were added to a solution of the appropriate ketone (1 equiv.) and anhydride (2.5 equiv.) in CH_2Cl_2 (0.1 M) at r.t., and the mixture was allowed to stir for 16 h. The reaction mixture was diluted with CH_2Cl_2, 0.1 M HCl was added, and the layers separated. The aqueous layer was extracted with CH_2Cl_2 and the combined organic layers were washed sequentially with 0.1 M HCl and brine, dried ($MgSO_4$) and concentrated *in vacuo* to afford

the crude compound, which was purified by Biotage column chromatography in the stated solvent system.

General Procedure D

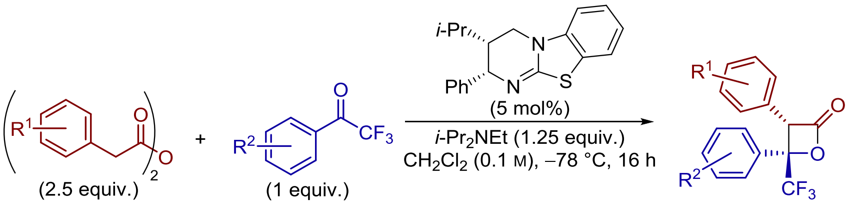

(2*S*,3*R*)-HyperBTM (5 mol%) and *i*-Pr_2NEt (1.25 equiv.) were added to a solution of the appropriate ketone (1 equiv.) and anhydride (2.5 equiv.) in CH_2Cl_2 (0.1 M) at −78 °C under an inert atmosphere, and the mixture was allowed to stir for 16 h. The reaction mixture was diluted with CH_2Cl_2, 0.1 M HCl was added, and the layers separated. The aqueous layer was extracted with CH_2Cl_2 and the combined organic layers were washed sequentially with 0.1 M HCl and brine, dried ($MgSO_4$) and concentrated *in vacuo* to afford the crude compound, which was purified by Biotage column chromatography in the stated solvent system.

General Procedure E

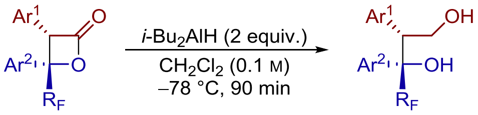

1 M *i*-Bu_2AlH (DIBAL) solution in hexane (2 equiv.) was added dropwise to a solution of a lactone (1 equiv.) in CH_2Cl_2 (0.1 M) at −78 °C under an inert atmosphere, and the reaction allowed to stir for 90 min. Aqueous NH_4Cl was added and the mixture was allowed to warm to r.t. The aqueous layer was extracted with CH_2Cl_2 (2 × 10 mL) and the combined organic layers dried ($MgSO_4$), filtered and concentrated *in vacuo* to give a residue, which was purified by column chromatography.

General Procedure F

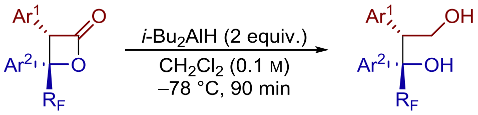

NaH 60% in mineral oil (2 equiv.) was added to a solution of a diol (1 equiv.) in THF (0.02 M) under an inert atmosphere and the mixture was stirred at r.t. for 10 min. 2,4,6-Triisopropylbenzenesulfonyl chloride (0.9 equiv.) was added and the mixture stirred for 16h. Aqueous NH_4Cl was added and the aqueous phase extracted with EtOAc (2 × 10 mL). The combined organic layers were dried ($MgSO_4$), filtered and concentrated *in vacuo* to give a residue, which was purified by column chromatography.

Synthesis of Homoanhydrides

2-Phenylacetic anhydride 17

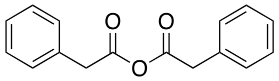

Following General Procedure A, phenylacetic acid (0.45 g, 3.33 mmol) and DCC (0.35 g, 1.70 mmol) in toluene (10 mL) gave 2-phenylacetic anhydride **17** as a colourless solid (0.43 g, quant.).

m.p. 68–70 °C {lit.[1] 72–72.5 °C}; **^{1}H NMR** (400 MHz, $CDCl_3$) δ_H: 3.76 (4H, s, 2 × C*H₂*), 7.23–7.25 (4H, m, Ar*H*), 7.32–7.38 (6H, m, Ar*H*). All data in accordance with the literature.[1]

2-(4-(Dimethylamino)phenyl)acetic anhydride S1

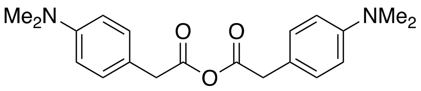

Following General Procedure A, 4-(dimethylamino)phenylacetic acid (1.5 g, 8.37 mmol) and DCC (864 g, 4.19 mmol) in toluene (28 mL) gave 2-(4-(dimethylamino)phenyl)acetic anhydride **S1** as a brown solid (1.47 g, quant.).

m.p. 80–82 °C; **^{1}H NMR** (500 MHz, $CDCl_3$) δ_H: 2.97 (12H, s, 4 × CH_3), 3.65 (4H, s, 2 × CH_2), 6.69–6.73 (4H, m, ArC(3,5)*H*), 7.09–7.13 (4H, m, ArC(2,6)*H*); **^{13}C{^{1}H} NMR** (126 MHz, $CDCl_3$) δ_C: 40.7 (*C*H_3), 41.2 (*C*H_2), 112.9 (Ar*C*(3,5)H), 119.8 (Ar*C*(1)), 130.2 (Ar*C*(2,6)H), 150.0 (Ar*C*(4)), 168.0 (*C*=O); **IR** (neat) ν_{max} cm^{-1} 2887, 1803 (C=O), 1694, 1522, 1044, 802; **HRMS** ($APCI^+$) $C_{20}H_{25}O_3N_2$ ($[M+H]^+$), found 341.1854, requires 341.1860 (−1.6 ppm).

2-(4-Methoxyphenyl)acetic anhydride S2

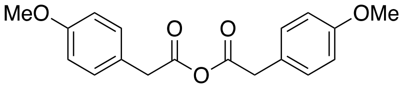

Following General Procedure A, 4-methoxyphenylacetic acid (2.5 g, 15 mmol) and DCC (1.55 g, 7.52 mmol) in toluene (50 mL) gave 2-(4-methoxyphenyl)acetic anhydride **S2** as a colourless solid (2.43 g, quant.).

m.p. 58–60 °C; {lit.[1] 60–62 °C}; **^{1}H NMR** (500 MHz, $CDCl_3$) δ_H: 3.68 (4H, s, 2 × CH_2), 3.83 (6H, s, 2 × OCH_3), 6.85–6.89 (4H, m, Ar*H*), 7.12–7.16 (4H, m, Ar*H*). All data in accordance with the literature.[1]

2-(4-Fluorophenyl)acetic anhydride S3

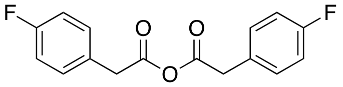

Following General Procedure A, 4-fluorophenylacetic acid (1.5 g, 9.73 mmol) and DCC (1 g, 4.86 mmol) in toluene (32 mL) gave 2-(4-fluorophenyl)acetic anhydride **S3** as a colourless solid (1.45 g, quant.).

m.p. 32–34 °C; {lit.[1] 36–38 °C}; **^{1}H NMR** (300 MHz, $CDCl_3$) δ_H: 3.69 (4H, s, 2 × C*H₂*), 6.90–7.05 (4H, m, Ar*H*), 7.08–7.22 (4H, m, Ar*H*). All data in accordance with the literature.[1]

2-(4-Bromophenyl)acetic anhydride S4

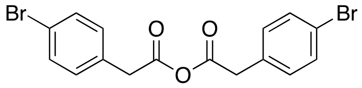

Following General Procedure A, 4-bromophenylacetic acid (1.5 g, 6.98 mmol) and DCC (720 mg, 3.49 mmol) in toluene (24 mL) gave 2-(4-bromophenyl)acetic anhydride **S4** as a colourless solid (1.47 g, quant.).

m.p. 75–77 °C; {lit.[1] 76–78 °C}; **^{1}H NMR** (500 MHz, $CDCl_3$) δ_H: 3.68 (4H, s, 2 × CH_2), 7.05–7.09 (4H, m, Ar*H*), 7.43–7.47 (4H, m, Ar*H*). All data in accordance with the literature.[1]

2-(4-(Trifluoromethylphenyl)acetic anhydride S5

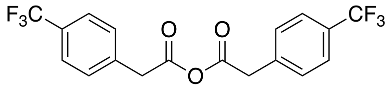

Following General Procedure A, 4-(trifluoromethyl)phenylacetic acid (2.0 g, 9.80 mmol) and DCC (1 g, 4.90 mmol) in toluene (31 mL) gave 2-(4-(trifluoromethylphenyl)acetic anhydride **S5** as a yellow solid (2.05 g, quant.)

m.p. 55–57 °C; {lit.[1] 58–60 °C}; **^{1}H NMR** (300 MHz, $CDCl_3$) δ_H: 3.80 (4H, s, 2 × CH_2), 7.33 (4H, m, Ar*H*), 7.59 (4H, m, Ar*H*). All data in accordance with the literature.[1]

2-(*m*-Tolyl)acetic anhydride S6

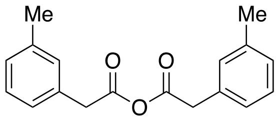

Following General Procedure A, *m*-tolylacetic acid (1.00 g, 6.7 mmol) and DCC (0.75 g, 3.7 mmol) in toluene (20 mL) gave 2-(*m*-tolyl)acetic anhydride **S6** (0.86 g, 46%) as a yellow oil.

^{1}H NMR (400 MHz, $CDCl_3$) δ_H: 2.37 (6H, s, 2 × ArCH_3), 3.71 (4H, s, 2 × CH_2), 7.01–7.10 (4H, m, Ar*H*), 7.11–7.17 (2H, m, Ar*H*), 7.20–7.30 (2H, m, Ar*H*). All data in accordance with the literature.[2]

2-(*o*-Tolyl)acetic anhydride S7

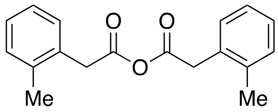

Following General Procedure A, *o*-tolylacetic acid (2.00 g, 13.4 mmol) and DCC (1.50 g, 7.4 mmol, 0.55 equiv.) in toluene (30 mL) gave 2-(*o*-tolyl)acetic anhydride **S7** (1.27 g, 34%) as a yellow oil.

^{1}H NMR (400 MHz, $CDCl_3$) δ_H: 2.26 (6H, m, 2 × ArCH_3), 3.74 (4H, m, 2 × CH_2), 7.12–7.24 (8H, m, Ar*H*). All data in accordance with the literature.[3]

2-(Thiophen-3-yl)acetic anhydride S8

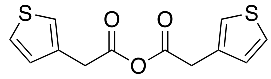

Following General Procedure A, 3-thiopheneacetic acid (2.5 g, 17.58 mmol) and DCC (1.8 g, 8.79 mmol) in toluene (59 mL) gave 2-(thiophen-3-yl)acetic anhydride **S8** as a yellow solid (2.43 g, quant.). **m.p.** 38–40 °C; {lit.[1] 40–42 °C}; **^{1}H NMR** (500 MHz, $CDCl_3$) δ_H: 3.79 (4H, s, 2 × CH_2), 6.99 (2H, dd, *J* 5.0, 1.3, Ar*H*), 7.15 (2H, dd, *J* 2.1, 1.1, Ar*H*), 7.31 (2H, dd, *J* 5.0, 3.0, Ar*H*). All data in accordance with the literature.[1]

2-(1-Methyl-1*H*-indol-3-yl)acetic anhydride S9

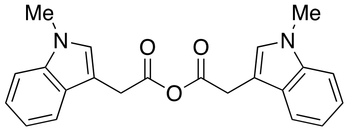

Following General Procedure A, 1-methyl-1*H*-indol-3-ylacetic acid (1.00 g, 5.3 mmol) and DCC (0.60 g, 2.9 mmol) in toluene (30 mL) gave 2-(1-methyl-1*H*-indol-3-yl)acetic anhydride **S9** as a yellow oil (1.18 g, 62%).

^{1}H NMR (500 MHz, $CDCl_3$) δ_H: 3.73 (6H, s, 2 × CH_3), 3.91 (4H, m, 2 × CH_2), 6.96 (2H, s, ArC(2)*H*), 7.15–7.20 (2H, m, Ar*H*), 7.21–7.25 (2H, m, ArC*H*), 7.32–7.36 (2H, m, ArC(6)*H*), 7.56 (2H, dd, *J* 7.8, 1.1, ArC(4)*H*). All data in accordance with the literature.[1]

(*E*)-Pent-3-enoic anhydride S10

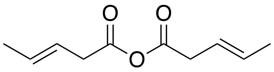

Following General Procedure A, (*E*)-3-pentenoic acid (2.5 g, 25 mmol) and DCC (2.6 g, 12.5 mmol) in toluene (83 mL) gave (*E*)-pent-3-enoic anhydride **S10** as a pale yellow oil (1.40 g, 61%).

^{1}H NMR (300 MHz, $CDCl_3$) δ_H: 1.69–1.73 (6H, m, 2 × CH_3), 3.11–3.20 (4H, m, 2 × CH_2), 5.44–5.57 (2H, m, 2 × C*H*), 5.57–5.71 (2H, m, 2 × C*H*). All data in accordance with the literature.[1]

2-(Phenylthio)acetic anhydride S11

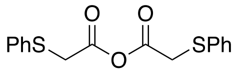

Following General Procedure A, 2-(phenylthio)acetic acid (1.11 g, 6.6 mmol) and DCC (0.70 g, 3.4 mmol) in toluene (20 mL) gave 2-(phenylthio)acetic anhydride **S11** (1.18 g, 62%) as a yellow oil.

^{1}H NMR (500 MHz, $CDCl_3$) δ_H: 3.67 (4H, s, 2 × CH_2), 7.26–7.37 (6H, m, PhH), 7.43–7.50 (4H, m, PhH). Used without further purification.

Synthesis of Perfluorinated Ketones

1-(4-Bromophenyl)-2,2,3,3,4,4,5,5,5-nonafluoropentan-1-one 18

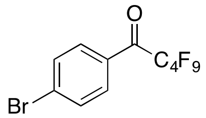

Following General Procedure B, 1,4-dibromobenzene (5 g, 21.2 mmol), *n*-BuLi (2.5 M in hexanes, 9.3 mL, 23.3 mmol), ethyl perfluoropentanoate (8.7 g, 29.7 mmol) and BF_3·OEt_2 (3.2 mL, 25.4 mmol) gave, after column chromatography (eluent Et_2O:hexane, 0% → 0.2%), 1-(4-bromophenyl)-2,2,3,3,4,4,5,5,5-nonafluoropentan-1-one **18** as a colourless oil (5.98 g, 70%).

^{1}H NMR (300 MHz, $CDCl_3$) δ_H: 7.66–7.73 (2H, m, ArC(3,5)H), 7.89–7.97 (2H, m, ArC(2,6)H); **^{19}F NMR** (376 MHz, $CDCl_3$) δ_F: −125.3 – −125.1 (m, CF_2), −122.0 – −121.8 (m, CF_2), −113.2 – −113.0 (m, CF_2), −80.9 (tt, *J* 9.8, 2.6, CF_3). All data in accordance with the literature.[4]

1-(4-Bromophenyl)-2,2,3,3,3-pentafluoropropan-1-one 19

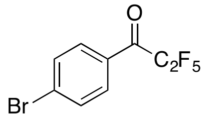

Following General Procedure B, 1,4-dibromobenzene (4.72 g, 20 mmol), *n*-BuLi (2.5 M in hexanes, 8.8 mL, 22 mmol), ethyl pentafluoropropionate (4.14 mL, 28 mmol) and BF_3·OEt_2 (3 mL, 24 mmol) gave, after distillation, 1-(4-bromophenyl)-2,2,3,3,3-pentafluoropropan-1-one **19** as a light yellow oil (3.04 g, 50%).

b.p. 45-46 °C, 1 mbar; {lit[4] 170 °C, 70 mbar}; **^{1}H NMR** (500 MHz, $CDCl_3$) δ_H: 7.70–7.75 (1H, m, ArC(3,5)H), 7.93-7.98 (1H, m, ArC(2,6)H); **^{19}F NMR** (471 MHz, $CDCl_3$) δ_F: −115.8 (CF_2), −81.7 (CF_3). All data in accordance with the literature.[4]

2,2,3,3,4,4,5,5,5-Nonafluoro-1-(3-fluorophenyl)pentan-1-one S12

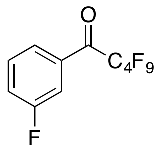

Following General Procedure B, 3-bromofluorobenzene (2.2 mL, 20 mmol), *n*-BuLi (2.5 M in hexanes, 8.8 mL, 22 mmol), ethyl perfluoropentanoate (8.18 g, 28 mmol) and $BF_3{\cdot}OEt_2$ (3 mL, 24 mmol) gave, after distillation, 2,2,3,3,4,4,5,5,5-nonafluoro-1-(3-fluorophenyl)pentan-1-one **S12** as a colourless oil (3.05 g, 32%).

b.p. 53-54 °C, 1 mbar; **^{1}H NMR** (500 MHz, $CDCl_3$) δ_H: 7.37–7.45 (1H, m, ArC(4)*H*), 7.50–7.58 (1H, m, ArC(5)*H*), 7.71–7.78 (1H, m, Ar(2)*H*), 7.83–7.91 (1H, m, ArC(6)*H*); **^{13}C NMR** (126 MHz, $CDCl_3$) δ_c: 105.0–121.1, (m, $CF_2CF_2CF_2CF_3$), 117.1 (dt, $^2J_{CF}$ 23.7, 3.3, Ar*C*(2)H), 122.8 (d, $^2J_{CF}$ 21.4, Ar*C*(4)H), 126.1–126.3 (m, Ar*C*(6)H), 131.0 (d, $^3J_{CF}$ 7.6, Ar*C*(5)H), 133.4 (app d, $^3J_{CF}$ 6.8, Ar*C*(1)), 162.8 (d, $^1J_{CF}$ 249.4, Ar*C*(3)F), 182.4 (t, $^2J_{CF}$ 26.5, *C*O); **^{19}F NMR** (471 MHz, $CDCl_3$) δ_F: −125.4 – −125.3 (m, CF_2), −122.1 – −121.9 (m, CF_2), −113.1 (t, *J* 12.6, CF_2), −110.4 – −110.3 (m, Ar*F*), −81.1 (tt, *J* 9.8, 2.8, CF_3); **IR** (neat) ν_{max} cm^{-1}: 1717 (CO); **HRMS** ($APCI^+$) $C_{11}H_5F_{10}O$ $[M+H]^+$, found 343.0186, requires 343.0175 (+3.2 ppm).

2,2,3,3,3-Pentafluoro-1-(3-fluorophenyl)propan-1-one S13

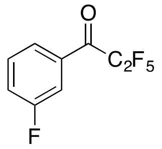

Following General Procedure B, 3-bromofluorobenzene (2.2 mL, 20 mmol), *n*-BuLi (1.65 M in hexanes, 13.3 mL, 22 mmol), ethyl pentafluoropropionate (4.14 mL, 28 mmol) and $BF_3{\cdot}OEt_2$ (3 mL, 24 mmol) gave, after distillation, 2,2,3,3,3-pentafluoro-1-(3-fluorophenyl)propan-1-one **S13** as a yellow oil (1.4 g, 29%).

b.p. 78–80 °C, 1 mbar; **^{1}H NMR** (500 MHz, $CDCl_3$) δ_H: 7.40–7.46 (1H, m, ArC(4)*H*), 7.52–7.59 (1H, m, ArC(5)*H*), 7.77–7.79 (1H, m, ArC(2)*H*), 7.87–7.92 (1H, m, ArC(6)*H*); **^{13}C NMR** (126 MHz, $CDCl_3$) δ_c: 105.8–121.7 (m, CF_2CF_3), 117.0 (dt, *J* 23.5, 2.9, Ar*C*(2)H), 122.9 (d, *J* 21.3, Ar*C*(4)H), 126.0–126.2 (m, Ar*C*(6)H), 131.0 (d, *J* 7.6, Ar*C*(5)H), 132.8 (d, *J* 6.9, Ar*C*(1)), 162.8 (d, $^1J_{CF}$ 249.5, Ar*C*(3)F), 182.3 (t, $^2J_{CF}$ 27.5, *C*O); **^{19}F NMR** (471 MHz, $CDCl_3$) δ_F: −115.76 (CF_2), −110.2 – −110.2 (m, Ar*F*), −81.6 (CF_3); **IR** (neat) ν_{max} cm^{-1}: 1717 (CO); **HRMS** (ESI^+) $C_9H_4F_6O$ $[M^+]$ found 242.0169, requires 242.0161 (+3.3 ppm).

2,2,3,3,3-Pentafluoro-1-(4-(trifluoromethyl)phenyl)propan-1-one S14

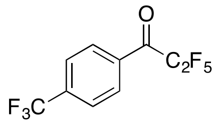

Following General Procedure B, 4-bromobenzotrifuoride (2.8 mL, 20 mmol), *n*-BuLi (2.5 M in hexanes, 8.8 mL, 22 mmol), ethyl pentafluoropropionate (4.14 mL, 28 mmol) and $BF_3{\cdot}OEt_2$ (3 mL, 24 mmol) gave, after column chromatography (Et_2O:hexane, 3:97), 2,2,3,3,3-pentafluoro-1-(4-(trifluoromethyl)phenyl)propan-1-one **S14** as a colourless oil (1.19 g, 20%).

^{1}H NMR (500 MHz, $CDCl_3$) δ_H: 7.80-7.86 (2H, m, ArC(3,5)*H*), 8.19-8.23 (2H, m, ArC(2,6)*H*); **^{19}F{^{1}H} NMR** (376 MHz, $CDCl_3$) δ_F: –115.9 (C*F*$_2$), –81.5 (C*F*$_3$), –63.6 (ArC(4)C*F*$_3$). All data in accordance with the literature.[4]

2,2,3,3,3-Pentafluoro-1-(3-methoxyphenyl)propan-1-one S15

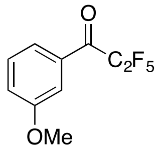

Following General Procedure B, 3-iodoanisole (2.38 mL, 20 mmol), *n*-BuLi (2.5 M in hexanes, 8.8 mL, 22 mmol), ethyl pentafluoropropionate (4.14 mL, 28 mmol) and $BF_3{\cdot}OEt_2$ (3 mL, 24 mmol) gave, after column chromatography (Et_2O:hexanes, 3:97), 2,2,3,3,3-pentafluoro-1-(3-methoxyphenyl)propan-1-one **S15** as a colourless oil (3.93 g, 78%).

^{1}H NMR (400 MHz, $CDCl_3$) δ_H: 3.88 (3H, s, OC*H*$_3$), 7.23–7.28 (m, 1H, ArC(4)*H*), 7.45 (1H, ddd, *J* 8.2, 7.7, 0.4, ArC(5)*H*), 7.57 (1H, dd, *J* 2.6, 1.4, ArC(2)*H*), 7.66–7.71 (1H, m, ArC(6)*H*). All data in accordance with the literature.[4]

2,2,3,3,4,4,5,5,5-Nonafluoro-1-phenylpentan-1-one S16

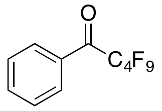

Following General Procedure B, bromobenzene (4.24 g, 40 mmol), *n*-BuLi (2.2 M in hexanes, 20 mL, 44 mmol), ethyl perfluoropentanoate (16.32 g, 56 mmol) and $BF_3{\cdot}OEt_2$ (5.92 mL, 48 mmol) gave, after distillation, 2,2,3,3,4,4,5,5,5-nonafluoro-1-phenylpentan-1-one **S16** as a colourless oil (9.36 g, 72%).

b.p. 54–56 °C, 1 mbar; {lit[1] 130 °C, 70 mbar}; **^{1}H NMR** (500 MHz, $CDCl_3$) δ_H: 7.52–7.58 (2H, m, PhC(3,5)*H*), 7.69–7.74 (1H, m, PhC(4)*H*), 8.04–8.11 (2H, m, PhC(2,6)*H*); **^{19}F NMR** (376 MHz, $CDCl_3$) δ_F:

−125.3 – −125.2 (m, CF_2), −121.9 (app tp, *J* 9.5, 3.8, CF_2), −112.9 (t, *J* 12.9, CF_2), −81.0 (t, *J* 9.9, CF_3). All data in accordance with the literature.[4]

2,2,3,3,3-Pentafluoro-1-(*m*-tolyl)propan-1-one S17

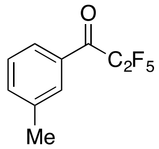

Following General Procedure B, 3-bromotoluene (2.43 mL, 20 mmol), *n*-BuLi (2.5 M in hexanes, 8.8 mL, 22 mmol), ethyl pentafluoropropionate (4.14 mL, 28 mmol) and BF_3·OEt_2 (3 mL, 24 mmol) gave, after distillation, 2,2,3,3,3-pentafluoro-1-(*m*-tolyl)propan-1-one **S17** as a light yellow oil (2.48 g, 52%).

b.p. 47–48 °C, 1 mbar; **^{1}H NMR** (500 MHz, $CDCl_3$) δ_H: 2.45 (3H, s, CH_3), 7.40–7.46 (1H, m, ArC(*5*)*H*), 7.50–7.55 (1H, m, Ar*C*(4)*H*), 7.86–7.92 (2H, m, ArC(2,6)*H*); **^{13}C NMR** (126 MHz, $CDCl_3$) δ_c: 21.5 (*C*H_3), 105.9–122.1 (m, *C*F_2*C*F_3), 127.5 (t, *J* 3.7, Ar*C*(6)H), 129.0 (Ar*C*(5)H), 130.6 (t, *J* 2.8, Ar*C*(2)H), 131.1 (Ar*C*(3)), 136.5 (Ar*C*(4)H), 139.2 (Ar*C*(1)), 183.5 (t, *J* 26.7, *C*O); **^{19}F NMR** (376 MHz, $CDCl_3$) δ_F: −115.4 (CF_2), −81.6 (CF_3); **IR** (neat) ν_{max} cm^{-1}: 1705 (CO); **HRMS** (APCI$^+$) $C_{10}H_8F_5O$ $[M+H]^+$, found 239.0501, requires 239.0490 (+4.6 ppm).

2,2,3,3,3-Pentafluoro-1-(thiophen-2-yl)propan-1-one S18

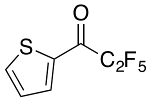

Following a modification of General Procedure B, 2-thienyllithium (1 M in THF/hexanes, 20 mL, 20 mmol), ethyl pentafluoropropionate (4.14 mL, 28 mmol) and BF_3·OEt_2 (3 mL, 24 mmol) gave, after column chromatography (Et_2O:hexane, 1:99), 2,2,3,3,3-pentafluoro-1-(thiophen-2-yl)propan-1-one **S18** as a yellow oil (2.46 g, 54%).

^{1}H NMR (400 MHz, $CDCl_3$) δ_H: 7.26 (1H, dd, *J* 4.9, 4.0, ArC(4)*H*), 7.92 (1H, dd, *J* 4.9, 1.1, ArC(5)*H*), 8.02 (1H, ddd, *J* 3.8, 3.0, 1.7, Ar*C*(3)*H*); **^{13}C{^{1}H} NMR** (101 MHz, $CDCl_3$) δ_C: 104.0–122.9 (m, *C*F_2*C*F_3), 129.3 (Ar*C*(4)H), 136.7 (t, *J* 5.4, Ar*C*(3)H), 137.4 (Ar*C*(5)H), 138.3 (Ar*C*(2)), 176.0 (t, *J* 27.1, *C*O); **^{19}F{^{1}H} NMR** (376 MHz, $CDCl_3$) δ_F: –117.4 (t, *J* 1.5, CF_2), –81.7 (s, CF_3); **IR** (neat) ν_{max} cm^{-1}: 1678 (CO); **HRMS** (APCI$^+$) $C_7H_4F_5OS$ ($[M+H]^+$), found 230.9897, requires 230.9898 (–0.2 ppm).

Synthesis of β-lactones

(3*S*,4*S*)-4-(4-Bromophenyl)-4-(perfluorobutyl)-3-phenyloxetan-2-one 21

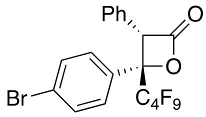

Following General Procedure C, ketone **18** (101 mg, 0.25 mmol), anhydride **17** (158 mg, 0.625 mmol), HyperBTM (4 mg, 0.0125 mmol) and *i*-Pr_2NEt (54 µL, 0.31 mmol) in CH_2Cl_2 gave, after Biotage chromatography (0→2% Et_2O in hexane) (3*S*,4*S*)-4-(4-bromophenyl)-4-(perfluorobutyl)-3-phenyloxetan-2-one **21** as a colourless oil (97 mg, 74%) as an inseparable mixture of diastereoisomers (> 95:5 dr).

$[\alpha]_D^{20}$ = +130.0 (*c* 1.0, $CHCl_3$); **Chiral HPLC analysis**, Chiralcel OD-H (97:3 hexane:IPA, flow rate 1.5 mLmin^{-1}, 211 nm, 40 °C) t_R(3*R*,4*R*): 2.8 min, t_R(3*S*,4*S*): 3.4 min, 97:3 er; **^{1}H NMR** (500 MHz, $CDCl_3$) δ_H: 5.60 (1H, s, C(3)*H*), 6.84–6.89 (2H, m, PhC(2,6)*H*), 6.95 (2H, app d, *J* 8.2, C(4)ArC(2,6)*H*), 7.18–7.23 (2H, m, PhC(3,5)*H*), 7.25–7.29 (1H, m, PhC(4)*H*), 7.33 (2H, app d, *J* 8.2, C(4)ArC(3,5)*H*); ^{13}C {^{1}H} NMR (126 MHz, $CDCl_3$) δ_C: 64.6 (d, *J* 4.5, *C*(3)H), 81.0 (dd, $^2J_{CF}$ 31.3, 23.4, *C*(4)), 105.0-119.5 (m, $CF_2CF_2CF_2CF_3$), 124.3 (C(4)Ar*C*(4)), 127.7 (C(4)Ar*C*(1)), 128.0 (Ph*C*(1)) 129.1 (C(4)Ar*C*(2,6)H + Ph*C*(3,5)H), 129.6 (Ph*C*(4)H), 129.8 (Ph*C*(2,6)H), 131.5 (C(4)Ar*C*(3,5)H), 166.0 (*C*(2)); **^{19}F NMR** (471 MHz, $CDCl_3$) δ_F: −128.0 – −124.3 (m, CF_2), −122.8 – −121.3 (m, CF_2), −118.5 – −115.9 (m, CF_2), −80.9 – −80.8 (m, CF_3); **IR** (neat) ν_{max} cm^{-1}: 1854 (C=O); **HRMS** (ESI$^-$) $C_{19}H_9BrF_9O_2$ [M−H]$^-$ found 518.9652, requires 518.9648 (+0.8 ppm).

(3*S*,4*S*)-4-(4-Bromophenyl)-4-(perfluoroethyl)-3-phenyloxetan-2-one 22

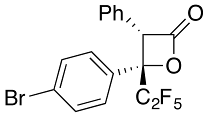

Following General Procedure C, ketone **19** (76 mg, 0.25 mmol), anhydride **17** (155 mg, 0.625 mmol), HyperBTM (4 mg, 0.0125 mmol) and *i*-Pr_2NEt (54 µL, 0.31 mmol) in CH_2Cl_2 gave, after Biotage chromatography (0→5%, Et_2O in hexane), **(**3*S*,4*S*)-4-(4-bromophenyl)-4-(perfluoroethyl)-3-phenyloxetan-2-one **22** as a colourless oil (79 mg, 75%) as an inseparable mixture of diastereoisomers (> 95:5 dr).

$[\alpha]_D^{20}$ = +132.9 (*c* 1.0, $CHCl_3$); **Chiral HPLC analysis,** Chiralcel OD-H (97:3 Hexane:IPA, flow rate 1.5 mLmin^{-1}, 211 nm, 40 °C) t_R(3*R*,4*R*): 3.0 min, t_R(3*S*,4*S*): 3.8 min, 97:3 er; **^{1}H NMR** (500 MHz, $CDCl_3$) δ_H: 5.59 (1H, s, C(3)*H*), 6.80–6.91 (2H, m, PhC(2,6)*H*), 6.89–6.98 (2H, m, C(4)ArC(2,6)*H*)), 7.18–7.24 (2H, m, PhC(3,5)*H*), 7.24–7.29 (1H, m, PhC(4)*H*), 7.31–7.36 (2H, m, C(4)ArC(3,5)*H*); *Minor diastereoisomer (selected signals)* δ_H: 5.28 (1H, s, C(3)*H*), 7.44–7.48 (3H, m, Ar*H*), 7.49–7.53 (2H, m, Ar*H*), 7.64–7.70

(2H, m, Ar*H*); ^{13}**C{^{1}H} NMR** (126 MHz, $CDCl_3$) δ_C: 64.1 (d, $^{3}J_{CF}$ 4.1, *C*(3)H), 80.1 (dd, $^{2}J_{CF}$ 30.4, 23.5, *C*(4)), 110.3–122.3 (m, CF_2CF_3), 124.4 (C(4)Ar*C*(4)), 127.8 (d, $^{3}J_{CF}$ 2.8), 127.8 (C(4)Ar*C*(1)), 128.1 (Ph*C*(1)), 129.0 (d, $^{4}J_{CF}$ 2.7, C(4)Ar*C*(2,6)H), 129.1 (Ph*C*(3,5)H), 129.5 (Ph*C*(4)H), 129.7 (Ph*C*(2,6)H), 131.5 (C(4)Ar*C*(3,5)H), 165.9 (*C*(2)); ^{19}**F NMR** (471 MHz, $CDCl_3$) δ_F: −124.5 (d, *J* 282, CF_2), −121.7 (d, *J* 282, CF_2), −79.0 (CF_3); **IR** (neat) ν_{max} cm^{-1}: 1853 (CO); **HRMS** (APCI$^+$) $C_{17}H_{11}BrF_5O_2$ $[M+H]^+$ found 420.9870 requires 420.9857 (+3.1 ppm).

(3*S*,4*S*)-4-(4-Bromophenyl)-3-phenyl-4-(trifluoromethyl)oxetan-2-one 23

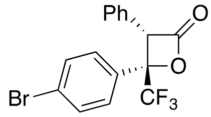

Following General Procedure D, 1-(4-bromophenyl)-2,2,2-trifluoroethan-1-one (63 mg, 0.25 mmol), anhydride **17** (155 mg, 0.625 mmol), HyperBTM (4 mg, 0.0125 mmol) and *i*-Pr_2NEt (54 µL, 0.31 mmol) in CH_2Cl_2 gave, after Biotage chromatography (0→5% Et_2O in hexane), (3*S*,4*S*)-4-(4-bromophenyl)-3-phenyl-4-(trifluoromethyl)oxetan-2-one **23** as a colourless oil (88 mg, 95%) as an inseparable mixture of diastereoisomers (> 95:5 dr).

$[\alpha]_D^{20}$ = +118.9 (*c* 1.0, $CHCl_3$); **Chiral HPLC analysis**, Chiralcel OD-H (97:3 Hexane:IPA, flow rate 1.5 mLmin^{-1}, 211 nm, 40 °C) t_R(3*R*,4*R*): 3.2 min, t_R(3*S*,4*S*): 4.1 min, 95:5 er; ^{1}**H NMR** (500 MHz, $CDCl_3$) δ_H: 5.41 (1H, s, C(3)*H*), 6.87–6.92 (2H, m, PhC(2,6)*H*), 6.96 (2H, app d, *J* 8.3, (C(4)ArC(2,6)*H*), 7.20–7.25 (2H, m, PhC(3,5)*H*), 7.25–7.30 (1H, m, PhC(4)*H*), 7.35 (2H, app d, *J* 8.6, C(4)ArC(3,5)*H*); *Minor diastereoisomer (selected signals)* δ_H: 5.28 (1H, s, C(3)*H*), 7.44–7.49 (3H, m, Ar*H*), 7.52 (2H, app d, *J* 8.4, Ar*H*), 7.66–7.70 (2H, m, Ar*H*); ^{13}**C{^{1}H} NMR** (126 MHz, $CDCl_3$) δ_C: 63.5 (*C*(3)H), 80.2 (q, $^{2}J_{CF}$ 32.8, *C*(4)), 123.4 (q, $^{1}J_{CF}$ 282.3, CF_3) 124.4 (C(4)Ar*C*(4)), 127.7 (Ph*C*(1)), 128.1 (C(4)Ar*C*(1)), 129.0 (C(4)Ar*C*(2,6)H), 129.2 (Ph*C*(3,5)H), 129.5 (Ph*C*(4)H), 129.6 (Ph*C*(2,6)H) 131.6 (C(4)Ar*C*(3,5)H), 165.8 (*C*(2)); ^{19}**F NMR** (471 MHz, $CDCl_3$) δ_F: −78.5 (CF_3); *Minor diastereoisomer* δ_F: −73.4 (CF_3); **IR** (neat) ν_{max} cm^{-1}: 1853 (CO); **HRMS** (APCI$^+$) $C_{16}H_{11}BrF_3O_2$ $[M+H]^+$ found 370.9895, requires 370.9889 (+1.6 ppm).

(3*S*,4*S*)-4-(3-Fluorophenyl)-4-(perfluorobutyl)-3-phenyloxetan-2-one 24

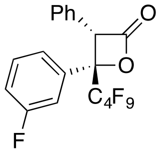

Following General Procedure C, ketone **S12** (86 mg, 0.25 mmol), anhydride **17** (158 mg, 0.625 mmol), HyperBTM (4 mg, 0.0125 mmol) and *i*-Pr_2NEt (54 µL, 0.31 mmol) in CH_2Cl_2 gave, after Biotage chromatography (0→3% Et_2O in hexane), (3*S*,4*S*)-4-(3-fluorophenyl)-4-(perfluorobutyl)-3-

phenyloxetan-2-one **24** as a colourless oil (88 mg, 77%) as an inseparable mixture of diastereoisomers (> 95:5 dr).

$[\alpha]_D^{20}$ = +100.5 (*c* 1.0, $CHCl_3$); **Chiral HPLC analysis**, Chiralpak IC (99.9:0.1 Hexane:IPA, flow rate 1.0 mLmin^{-1}, 211 nm, 30 °C) t_R(3*R*,4*R*): 4.4 min, t_R(3*S*,4*S*): 5.3 min, 96:4 er; **^{1}H NMR** (500 MHz, $CDCl_3$) δ_H: 5.62 (1H, s, C(3)*H*), 6.80–6.91 (4H, m, Ph(2,6)*H* + C(4)ArC(2,6)*H*), 6.92–6.98 (1H, m, C(4)ArC(4)*H*), 7.12–7.17 (1H, m, C(4)ArC(5)*H*), 7.17–7.22 (2H, m, Ph(3,5)*H*), 7.23–7.28 (1H, m, PhC(4)*H*); **^{13}C{^{1}H} NMR** (126 MHz, $CDCl_3$) δ_C: 64.8 (d, $^3J_{CF}$ 4.7, *C*(3)H), 80.8 (dd, $^2J_{CF}$ 30.6, 24.3, *C*(4)), 115.0 (d, *J* 24.4, C(4)Ar*C*(2)H), 116.7 (d, *J* 21.0, C(4)Ar*C*(4)H), 123.3 (C(4)Ar*C*(6)H), 128.0 (Ph*C*(1)), 129.1 (Ph*C*(3,5)H), 129.5 (Ph*C*(4)H), 129.7 (Ph*C*(2,6)H), 129.9 (d, *J* 8.0, C(4)Ar*C*(5)H), 131.1 (d, *J* 8.8, C(4)Ar*C*(1)), 162.3 (d, $^1J_{CF}$ 247.6, C(4)Ar*C*(3)F), 165.9 (*C*(2)); **^{19}F{^{1}H} NMR** (471 MHz, $CDCl_3$) δ_F: −128.0 – −124.4 (m, C*F*$_2$), −122.8 – −121.8 (m, C*F*$_2$), −118.6 – −115.7 (m, C*F*$_2$), −111.6 (Ar*F*), −80.9 – −80.8 (m, C*F*$_3$); **IR** (neat) ν_{max} cm^{-1}: 1855 (CO); **HRMS** (APCI$^+$) $C_{19}H_{10}F_{10}O_2^+$ [M]$^+$ found 460.0521, requires 460.0516 (+1.2 ppm).

(3*S*,4*S*)-4-(3-Fluorophenyl)-4-(perfluoroethyl)-3-phenyloxetan-2-one 25

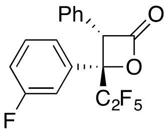

Following General Procedure C, ketone **S13** (61 mg, 0.25 mmol), anhydride **17** (158 mg, 0.625 mmol), HyperBTM (4 mg, 0.0125 mmol) and *i*-Pr_2NEt (54 µL, 0.31 mmol) in CH_2Cl_2 gave, after Biotage chromatography (0→3% Et_2O in hexane), (3*S*,4*S*)-4-(3-fluorophenyl)-4-(perfluoroethyl)-3-phenyloxetan-2-one **25** as a colourless oil (59 mg, 78%) as an inseparable mixture of diastereoisomers (> 95:5 dr).

$[\alpha]_D^{20}$ = +92.3 (*c* 1.0, $CHCl_3$); **Chiral HPLC analysis**, Chiralcel OD-H (99:1 Hexane:IPA, flow rate 1.5 mLmin^{-1}, 211 nm, 40 °C) t_R(3*R*,4*R*): 3.5 min, t_R(3*S*,4*S*): 5.0 min, 95:5 er; **^{1}H NMR** (500 MHz, $CDCl_3$) δ_H: 5.61 (1H, s, C(3)*H*), 6.79–6.87 (2H, m, C(4)ArC(2,6)*H*), 6.87–6.91 (2H, m, PhC(2,6)*H*), 6.93–6.99 (1H, m, C(4)ArC(4)*H*), 7.13–7.17 (C(4)ArC(5)*H*), 7.18–7.22 (2H, m, PhC(3,5)*H*), 7.23–7.28 (1H, m, PhC(4)*H*); **^{13}C{^{1}H} NMR** (126 MHz, $CDCl_3$) δ_C: 64.3 (d, $^3J_{CF}$ 4.2, *C*(3)H), 79.8 (dd, $^2J_{CF}$ 30.4, 23.6, C(4)), 110.4–122.2 (m, *C*F$_2$*C*F$_3$), 114.8 (dd, $^{2,4}J_{CF}$ 24.3, 2.7, C(4)Ar*C*(2)H), 116.8 (d, $^2J_{CF}$ 21.0, C(4)Ar*C*(4)H), 123.2 (app t, $^4J_{CF}$ 3.1, C(4)Ar*C*(6)H), 128.1 (Ph*C*(1)), 129.1 (Ph*C*(3,5)H), 129.5 (Ph*C*(4)H), 129.6 (Ph*C*(2,6)H), 130.0 (d, *J* 8.2, C(4)Ar*C*(5)H), 131.1 (dd, $^3J_{CF}$ 7.9, 2.4, C(4)Ar*C*(1)), 162.3 (d, *J* 247.5, C(4)Ar*C*(3)F), 165.8 (*C*(2)); **^{19}F NMR** (471 MHz, $CDCl_3$) δ_F: −124.4 (d, *J* 282.5, C*F*$_2$), −121.8 – −121.1 (m, C*F*$_2$), −111.6 (Ar*F*), −79.1 (C*F*$_3$); **IR** (neat) ν_{max} cm^{-1}: 1853 (CO); **HRMS** (APCI$^+$) $C_{17}H_{10}O_2F_6^+$ [M]$^+$ found 360.0585 requires 360.0580 (+1.4 ppm).

(3*S*,4*S*)-4-(3-Fluorophenyl)-3-phenyl-4-(trifluoromethyl)oxetan-2-one 26

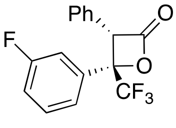

Following General Procedure D, 2,2,2-trifluoro-1-(3-fluorophenyl)ethan-1-one (48 mg, 0.25 mmol), anhydride **17** (158 mg, 0.625 mmol), HyperBTM (4 mg, 0.0125 mmol) and *i*-Pr_2NEt (54 µL, 0.31 mmol) in CH_2Cl_2 gave, after Biotage chromatography (0→3% Et_2O in hexane), (3*S*,4*S*)-4-(3-fluorophenyl)-3-phenyl-4-(trifluoromethyl)oxetan-2-one **26** as a colourless oil (53 mg, 68%) as an inseparable mixture of diastereoisomers (> 95:5 dr).

$[\alpha]_D^{20}$ = +130.6 (*c* 1.0, $CHCl_3$); **Chiral HPLC analysis**, Chiralcel OD-H (97:3 Hexane:IPA, flow rate 1.5 mLmin^{-1}, 211 nm, 40 °C) t_R(3*R*,4*R*): 3.2 min, t_R(3*S*,4*S*): 3.9 min, 97:3 er; **^{1}H NMR** (500 MHz, $CDCl_3$) δ_H: 5.42 (1H, s, C(3)*H*), 6.63–6.69 (2H, m, C(4)ArC(4,6)*H*), 6.69–6.73 (2H, m, PhC(3,5)*H*), 6.74–6.80 (1H, m, C(4)ArC(2)*H*), 6.95–7.06 (4H, m, Ph(2,4,6)*H* + C(4)ArC(5)*H*); *Minor diastereoisomer (selected signals)* δ_H: 5.31 (1H, s, C(3)*H*); **^{13}C{^{1}H} NMR** (126 MHz, $CDCl_3$) δ_C: 63.7 (*C*(3)H), 80.0 (q, $^2J_{CF}$ 32.8, *C*(4)), 114.8 (d, $^2J_{CF}$ 24.4, C(4)Ar*C*(4)H), 116.8 (d, $^2J_{CF}$ 21.0, C(4)Ar*C*(2)H), 123.1 (d, $^4J_{CF}$ 3.1, C(4)Ar*C*(6)H), 123.5 (q, $^1J_{CF}$ 282.3, *C*F_3), 128.1 (Ph*C*(1)), 129.1 (Ph*C*(4)H), 129.4 (Ph*C*(2,6)H), 129.5 (Ph*C*(3,5)H), 130.1 (d, $^3J_{CF}$ 8.2, C(4)Ar*C*(5)H), 131.1 (d, $^3J_{CF}$ 7.6, C(4)Ar*C*(1)), 162.4 (d, $^1J_{CF}$ 247.6, C(4)Ar*C*(3)F), 165.8 (*C*(2)); **^{19}F NMR** (471 MHz, $CDCl_3$) δ_F: −111.6 (Ar*F*), −78.2 (C*F*$_3$); *Minor diastereoisomer* δ_F: −110.4 (Ar*F*), −73.2 (C*F*$_3$); **IR** (neat) ν_{max} cm^{-1}: 1855 (CO); **HRMS** (APCI$^+$) $C_{16}H_{10}F_4O_2^+$ [M]$^+$ found 310.0616, requires 310.0611 (+1.6 ppm).

(3*S*,4*S*)-4-(Perfluoroethyl)-3-phenyl-4-(4-(trifluoromethyl)phenyl)oxetan-2-one 27

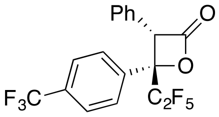

Following General Procedure C, ketone **S14** (73 mg, 0.25 mmol), anhydride **17** (158 mg, 0.625 mmol), HyperBTM (4 mg, 0.0125 mmol) and *i*-Pr_2NEt (54 µL, 0.31 mmol) in CH_2Cl_2 gave, after Biotage chromatography (0→5% Et_2O in hexane), (3*S*,4*S*)-4-(perfluoroethyl)-3-phenyl-4-(4-(trifluoromethyl)phenyl)oxetan-2-one **27** as a colourless oil (97 mg, 94%) as an inseparable mixture of diastereoisomers (> 95:5 dr).

$[\alpha]_D^{20}$ = +107.8 (*c* 1.0, $CHCl_3$); **Chiral HPLC analysis**, Chiralcel OD-H (97:3 Hexane:IPA, flow rate 1.5 mLmin^{-1}, 211 nm, 40 °C) t_R(3*R*,4*R*): 2.8 min, t_R(3*S*,4*S*): 3.8 min, 94:6 er; **^{1}H NMR** (500 MHz, $CDCl_3$) δ_H: 5.65 (1H, s, C(3)*H*), 6.84–6.89 (2H, m, PhC(2,6)*H*), 7.15-7.29 (5H, m, PhC(3,4,5)*H* + C(4)ArC(2,6)*H*), 7.46–7.48 (2H, m, C(4)ArC(3,5)*H*); *Minor diastereoisomer (selected signals)* δ_H: 5.31 (1H, s, C(3)*H*);

^{13}**C{^{1}H} NMR** (126 MHz, $CDCl_3$) δ_C: 64.4 (d, $^3J_{CF}$ 4.3, *C*(3)H), 80.0 (dd, $^2J_{CF}$ 30.5, 23.6, *C*(4)), 110.2–122.3 (CF_2CF_3), 123.7 (q, $^1J_{CF}$ 272.5, ArCF_3), 125.2 (q, $^3J_{CF}$ 3.8, C(4)Ar*C*(3,5)H), 127.9 (Ph*C*(1)), 128.0 (q, *J* 2.8, C(4)Ar*C*(2,6)H), 129.2 (Ph*C*(3,5)H), 129.7 (Ph*C*(2,6)H), 129.7 (Ph*C*(4)H), 131.8 (q, $^2J_{CF}$ 32.9, C(4)Ar*C*(4)), 132.8 (C(4)Ar*C*(1)), 165.6 (*C*(2)); ^{19}**F NMR** (471 MHz, $CDCl_3$) δ_F: −124.42 (d, *J* 282.4, CF_2), −121.49 (d, *J* 282.7, CF_2), −79.02 (CF_2CF_3), −63.10 (Ar-CF_3); *Minor diastereoisomer* δ_F: −121.9 (d, *J* 288.1, CF_2), −119.5 (d, *J* 288.1, CF_2), −79.5 (CF_2CF_3), −63.0 (Ar-CF_3); **IR** (neat) ν_{max} cm^{-1}: 1853 (CO); **HRMS** (APCI$^+$) $C_{18}H_{10}F_8O_2^+$ $[M]^+$ found 410.0550, requires 410.0548 (+0.5 ppm).

(3*S*,4*S*)-4-(3-Methoxyphenyl)-4-(perfluoroethyl)-3-phenyloxetan-2-one 28

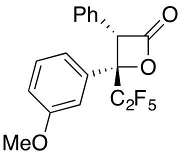

Following General Procedure C, ketone **S15** (64 mg, 0.25 mmol), anhydride **17** (158 mg, 0.625 mmol), HyperBTM (4 mg, 0.0125 mmol) and *i*-Pr_2NEt (54 µL, 0.31 mmol) in CH_2Cl_2 gave, after Biotage chromatography (0→3% Et_2O in hexane), (3*S*,4*S*)-4-(3-methoxyphenyl)-4-(perfluoroethyl)-3-phenyloxetan-2-one **28** as a colourless oil (88 mg, 95%) as an inseparable mixture of diastereoisomers (> 95:5 dr).

$[\alpha]_D^{20}$ = +112.6 (*c* 1.0, $CHCl_3$); **Chiral HPLC analysis**, Chiralcel OD-H (97:3 Hexane:IPA, flow rate 1.5 $mLmin^{-1}$, 211 nm, 40 °C) t_R(3*R*,4*R*): 3.1 min, t_R(3*S*,4*S*): 3.7 min, 88:12 er; ^{1}**H NMR** (500 MHz, $CDCl_3$) δ_H: 3.60 (3H, s, OCH_3), 5.58 (1H, s, C(3)*H*), 6.56 (1H, br s, C(4)ArC(2)*H*), 6.62–6.68 (1H, m, C(4)ArC(6)*H*), 6.75–6.81 (1H, m, C(4)ArC(4)*H*), 6.87–6.92 (2H, m, PhC(2,6)*H*), 7.09 (1H, app t, *J* 8.0, C(4)ArC(5)*H*), 7.17–7.22 (2H, m, Ph(3,5)*H*), 7.22–7.26 (1H, m, PhC(4)*H*); ^{13}**C{^{1}H} NMR** (126 MHz, $CDCl_3$) δ_C: 55.3 (OCH_3), 64.0 (d, $^3J_{CF}$ 4.2, *C*(3)H), 80.3 (dd, $^2J_{CF}$ 30.1, 23.1, *C*(4)), 110.3–120.4 (m, CF_2CF_3), 112.7 (d, *J* 2.8, C(4)Ar*C*(2)H), 115.6 (C(4)Ar*C*(4)H), 119.8 (d, *J* 2.8, C(4)Ar*C*(6)H), 128.5 (Ph*C*(1)), 128.9 (Ph*C*(3,5)H), 129.3 (Ph*C*(4)H), 129.3 (C(4)Ar*C*(5)H), 129.8 (Ph*C*(2,6)H), 129.9 (d, *J* 3.0, C(4)Ar*C*(1)), 159.3 (C(4)Ar*C*(3)OMe), 166.4 (*C*(2)); ^{19}**F NMR** (471 MHz, $CDCl_3$) δ_F: −124.3 (d, *J* 281.6, CF_2), −121.5 (d, *J* 281.5, CF_2), −79.1 (m, CF_3); **IR** (neat) ν_{max} cm^{-1}: 1852 (CO); **HRMS** (NSI$^+$) $C_{18}H_{17}F_5NO_3^+$ $[M+NH_4]^+$ found 390.1125, requires 390.1123 (+0.5 ppm).

(3*S*,4*S*)-4-(Perfluorobutyl)-3,4-diphenyloxetan-2-one 29

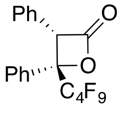

Following General Procedure C, ketone **S16** (81 mg, 0.25 mmol), anhydride **17** (158 mg, 0.625 mmol), HyperBTM (4 mg, 0.0125 mmol) and *i*-Pr_2NEt (54 µL, 0.31 mmol) in CH_2Cl_2 gave, after Biotage

chromatography (0→5% Et_2O in hexane), (3*S*,4*S*)-4-(perfluorobutyl)-3,4-diphenyloxetan-2-one **29** as a colourless oil (55 mg, 50%) as an inseparable mixture of diastereoisomers (> 95:5 dr).

$[\alpha]_D^{20}$ = +72.1 (*c* 0.4, $CHCl_3$); **Chiral HPLC analysis**, Chiralpak IC (99.9:0.1 Hexane:IPA, flow rate 1 $mLmin^{-1}$, 211 nm, 30 °C) t_R(3*R*,4*R*): 3.3 min, t_R(3*S*,4*S*): 3.9 min, 98:2 er; **^{1}H NMR** (500 MHz, $CDCl_3$) δ_H: 5.59 (1H, s, C(3)*H*), 6.83–6.88 (2H, m, C(3)PhC(2,6)*H*), 7.05–7.11 (2H, m, C(4)PhC(2,6)*H*), 7.13–7.20 (4H, m, C(3)PhC(3,5)*H* + C(4)PhC(3,5)*H*), 7.20–7.27 (2H, m, C(3)PhC(4)*H* + C(4)PhC(4)*H*); **^{13}C{^{1}H} NMR** (126 MHz, $CDCl_3$) δ_C: 64.3 (d, $^3J_{CF}$ 4.5, *C*(3)H), 81.1 (dd, *J* 30.8, 23.1, *C*(4)), 108–119 (m, $CF_2CF_2CF_2CF_3$), 127.3 (C(4)Ph*C*(2,6)H), 127.9 (C(4)Ph*C*(3,5)H), 128.1 (C(3)Ar*C*(1)), 128.3 (C(4)Ar*C*(1)), 128.6 (C(3)Ph*C*(3,5)H), 129.0 (C(3)Ph*C*(4)H), 129.2 (C(4)Ph*C*(4)H), 129.6 (C(3)Ph*C*(2,6)H), 166.2 (*C*(2)); ^{19}F (471 MHz, $CDCl_3$) δ_F: −128.0 – −124.5 (m, CF_2), −123.0 – −121.3 (m, CF_2), −118.8 – −115.6 (m, CF_2), −80.8 (dd, *J* 11.4, 8.2, CF_3); **IR** (neat) ν_{max} cm^{-1}: 1846 (CO); HRMS (NSI^+) $C_{19}H_{15}NF_9O_2^+$ $[M+NH_4]^+$ found 460.0950 requires 460.0954 (−0.9 ppm).

(3*S*,4*S*)-4-(Perfluoroethyl)-3-phenyl-4-(*m*-tolyl)oxetan-2-one 30

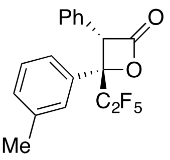

Following General Procedure C, ketone **S17** (60 mg, 0.25 mmol), anhydride **17** (158 mg, 0.625 mmol), HyperBTM (4 mg, 0.0125 mmol) and *i*-Pr_2NEt (54 µL, 0.31 mmol) in CH_2Cl_2 gave, after Biotage chromatography (0→3% Et_2O in hexane), (3*S*,4*S*)-4-(perfluoroethyl)-3-phenyl-4-(*m*-tolyl)oxetan-2-one **30** as a colourless oil (29 mg, 49%) as an inseparable mixture of diastereoisomers (> 95:5 dr).

$[\alpha]_D^{20}$ = +97.5 (*c* 0.5, $CHCl_3$); **Chiral HPLC analysis**, Chiralcel OD-H (99.5:0.5 Hexane:IPA, flow rate 1.5 $mLmin^{-1}$, 211 nm, 40 °C) t_R(3*R*,4*R*): 3.5 min, t_R(3*S*,4*S*): 4.9 min, 92:8 er; **^{1}H NMR** (500 MHz, $CDCl_3$) δ_H: 2.18 (3H, s, C*H*$_3$), 5.56 (1H, s, C(3)*H*), 6.79–6.84 (1H, br m, C(4)Ar*H*), 6.84–6.90 (3H, m, Ph*C*(2,6)*H* + C(4)Ar*H*), 7.03–7.06 (2H, m, C(4)Ar*H*), 7.16–7.20 (2H, m, Ph*C*(3,5)*H*), 7.21–7.25 (1H, m, Ph*C*(4)*H*); *Minor diastereoisomer (selected signals)* δ_H: 2.46 (3H, s, C*H*$_3$), 5.30 (1H, s,C(3)*H*); **^{13}C{^{1}H} NMR** (126 MHz, $CDCl_3$) δ_C: 20.4 (*C*H$_3$), 63.0 (d, $^3J_{CF}$ 4.1, *C*(3)H), 123.5 (C(4)Ar*C*H) , 127.0 (C(4)Ar*C*H), 127.0 (C(4)Ar*C*H), 127.4 (Ph*C*(1)), 127.5 (C(4)Ar*C*(1)), 127.8 (Ph*C*(3,5)H), 128.2 (Ph*C*(4)H), 128.8 (Ph*C*(2,6)H), 129.2 (C(4)Ar*C*H), 137.0 (C(4)Ar*C*(3)), 165.6 (*C*(2)); C(4) and CF_2CF_3 not observed; **^{19}F NMR** (471 MHz, $CDCl_3$) δ_F: −124.34 (d, *J* 281.2, C*F*$_2$), −121.63 (d, *J* 280.7, C*F*$_2$), −79.04 (d, *J* 1.6, C*F*$_3$); *Minor diastereoisomer* δ_F: −121.9 (d, *J* 287.5, C*F*$_2$), −119.0 (d, *J* 286.9, C*F*$_2$), −79.6 (C*F*$_3$); **IR** (neat) ν_{max} cm^{-1}: 1853 (CO); **HRMS** ($APCI^+$) $C_{18}H_{14}F_5O_2^+$ $[M+H]^+$ found 357.0922 requires 357.0908 (+3.9 ppm).

(3*S*,4*S*)-3-Phenyl-4-(*p*-tolyl)-4-(trifluoromethyl)oxetan-2-one 32

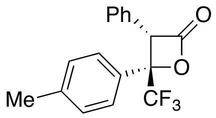

Following General Procedure D, 4-(trifluoroacetyl)toluene (47 mg, 0.25 mmol), anhydride **17** (158 mg, 0.625 mmol), HyperBTM (4 mg, 0.0125 mmol) and *i*-Pr_2NEt (54 µL, 0.31 mmol) in CH_2Cl_2 gave, after Biotage chromatography (0→3% Et_2O in hexane), (3*S*,4*S*)-4-(3-fluorophenyl)-3-phenyl-4-(trifluoromethyl)oxetan-2-one **32** as a colourless oil (59 mg, 78%) as an inseparable mixture of diastereoisomers (90:10 dr).

$[\alpha]_D^{20}$ = +150.9 (*c* 0.5, $CHCl_3$); **Chiral HPLC analysis**, Chiralcel OD-H (97:3 Hexane:IPA, flow rate 1.5 mLmin^{-1}, 211 nm, 40 °C) t_R(3*R*,4*R*): 3.0 min, t_R(3*S*,4*S*): 3.4 min, 99:1 er; ^{1}H NMR (500 MHz, $CDCl_3$) δ_H: 2.26 (3H, s, C*H*$_3$), 5.38 (1H, s, C(3)*H*), 6.88–6.93 (2H, m, Ph*C*(2,6)*H*), 6.97 (2H, d, *J* 8.2, C(4)Ar*C*(2,6)*H*), 7.01 (2H, d, *J* 8.2, C(4)Ar*C*(3,5)*H*), 7.17–7.22 (2H, m, Ph*C*(3,5)*H*), 7.22–7.26 (1H, m, PhC(4)*H*); *Minor diastereoisomer (selected signals)* δ_H: 2.44 (3H, s, C*H*$_3$), 5.31 (1H, s, C(3)*H*); **^{13}C{^{1}H} NMR** (126 MHz, $CDCl_3$) δ_C: 21.3 (*C*H$_3$), 63.3 (*C*(3)H), 80.6 (q, $^3J_{CF}$ 32.6, *C*(4)), 123.8 (q, $^1J_{CF}$ 281.5, *C*F$_3$), 125.5 (C(4)Ar*C*(1)), 127.3 (C(4)Ar*C*(2,6)H), 128.6 (Ph*C*(1)), 128.9 (Ph*C*(3,5)H), 129.0 (C(4)Ar*C*(3,5)H), 129.2 (Ph*C*(4)H), 129.7 (Ph*C*(2,6)H), 139.6 (C(4)Ar*C*(4)Me), 166.5 (*C*(2)); **^{19}F NMR** (471 MHz, $CDCl_3$) δ_F: −78.6 (C*F*$_3$); *Minor diastereoisomer* δ_F: −73.5 (C*F*$_3$); **IR** (neat) ν_{max} cm^{-1}: 1849 (CO); **HRMS** (NSI$^+$) $C_{17}H_{13}F_3O_2Na^+$ $[M+Na]^+$ found 329.0759, requires 329.0765 (−0.3 ppm).

(3*S*,4*S*)-4-(4-Methoxyphenyl)-3-phenyl-4-(trifluoromethyl)oxetan-2-one 33

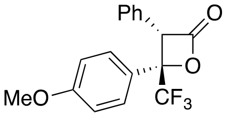

Following General Procedure D, 2,2,2-trifluoro-1-(4-methyoxyphenyl)ethanone (51 mg, 0.25 mmol), anhydride **17** (158 mg, 0.625 mmol), HyperBTM (4 mg, 0.0125 mmol) and *i*-Pr_2NEt (54 µL, 0.31 mmol) in CH_2Cl_2 gave, after Biotage chromatography (0→3% Et_2O in hexane), (3*S*,4*S*)-4-(4-methoxyphenyl)-3-phenyl-4-(trifluoromethyl)oxetan-2-one **33** as a colourless oil (18 mg, 22%) as an inseparable mixture of diastereoisomers (> 95:5 dr).

$[\alpha]_D^{20}$ = +163.6 (*c* 0.5, $CHCl_3$); **Chiral HPLC analysis**, Chiralcel OD-H (97:3 Hexane:IPA, flow rate 1.5 mLmin^{-1}, 211 nm, 40 °C) t_R(3*R*,4*R*): 3.6 min, t_R(3*S*,4*S*): 4.5 min, 99:1 er; **^{1}H NMR** (500 MHz, $CDCl_3$) δ_H: 3.74 (3H, s, OC*H*$_3$), 5.37 (1H, s, C(3)*H*), 6.68–6.74 (2H, m, C(4)ArC(3,5)*H*), 6.86–6.94 (2H, m, PhC(2,6)*H*), 6.98–7.02 (2H, m, C(4)ArC(2,6)*H*), 7.18–7.22 (2H, m, PhC(3,5)*H*), 7.23–7.26 (1H, m, PhC(4)*H*); **^{13}C{^{1}H} NMR** (126 MHz, $CDCl_3$) δ_C: 55.3 (O*C*H$_3$), 63.3 (*C*(3)H), 80.5 (q, $^3J_{CF}$ 32.6, *C*(4)), 113.7 (C(4)Ar*C*(3,5)H), 120.4 (C(4)Ar*C*(1)), 123.8 (q, *J* 282.1, *C*F$_3$), 128.6 (Ph*C*(1)), 128.8 (C(4)Ar*C*(2,6)H),

129.0 (Ph*C*(3,5)H), 129.2 (Ph*C*(4)H), 129.7 (Ph*C*(2,6)H), 160.4 (C(4)Ar*C*(4)OMe), 166.5 (*C*(2)); **^{19}F NMR** (471 MHz, $CDCl_3$) δ_F: −78.8 (C*F*$_3$); **IR** (neat) ν_{max} cm^{-1}: 1851 (CO); **HRMS** (APCI$^+$) $C_{17}H_{14}F_3O_3^+$ [M+H]$^+$ found 323.0896, requires 323.0890 (+1.9 ppm).

(3*S*,4*R*)-4-(Perfluoroethyl)-3-phenyl-4-(thiophen-2-yl)oxetan-2-one 34

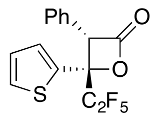

Following General Procedure C, ketone **S18** (57 mg, 0.25 mmol), anhydride **17** (158 mg, 0.625 mmol), HyperBTM (4 mg, 0.0125 mmol) and *i*-Pr$_2$NEt (54 µL, 0.31 mmol) in CH_2Cl_2 gave, after Biotage chromatography (0→3% Et_2O in hexane), (3*S*,4*R*)-4-(perfluoroethyl)-3-phenyl-4-(thiophen-2-yl)oxetan-2-one **34** as a colourless oil (37 mg, 43%) as an inseparable mixture of diastereoisomers (> 95:5 dr).

$[\alpha]_D^{20}$ = +84.1 (*c* 1.0, $CHCl_3$); **Chiral HPLC analysis**, Chiralcel OD-H (99.9:0.1 Hexane:IPA, flow rate 1.5 mLmin^{-1}, 211 nm, 40 °C) t_R(3*R*,4*S*): 5.5 min, t_R(3*S*,4*R*): 11.9 min, 87:13 er; **^{1}H NMR** (500 MHz, $CDCl_3$) δ_H: 5.65 (1H, s, C(3)*H*), 6.80 (1H, dd, *J* 3.4, 1.7, C(4)ArC(3)*H*), 6.87 (1H, dd, *J* 5.1, 3.7, C(4)ArC(4)*H*), 6.98–7.02 (2H, m, PhC(2,6)*H*), 7.21-2.27 (2H, m, PhC(3,5)*H*), 7.28–7.32 (2H, m, PhC(4)*H* + C(4)ArC(5)*H*); **^{13}C{^{1}H} NMR** (126 MHz, $CDCl_3$) δ_C: 64.8 (d, *J* 4.1, *C*(3)H), 79.6 (dd, *J* 32.8, 25.1, *C*(4)), 110.0–124.0 (m, *C*F$_2$*C*F$_3$), 127.2 (C(4)Ar*C*(4)H), 128.3 (C(4)Ar*C*(5)H), 128.5 (C(4)Ar*C*(3)H), 128.8 (Ph*C*(4)H), 128.9 (Ph*C*(3,5)H), 129.3 (Ph*C*(2,6)H), 129.4 (Ph*C*(1)), 130.6 (d, *J* 3.8, C(4)Ar*C*(2)), 165.7 (*C*(2)); **^{19}F NMR** (471 MHz, $CDCl_3$) δ_F: −124.8 (d, *J* 281.9, C*F*$_2$), −122.2 (d, *J* 282.0,C*F*$_2$), −79.0 (C*F*$_3$); **IR** (neat) ν_{max} cm^{-1}: 1857 (CO); **HRMS** (APCI$^+$) $C_{15}H_{10}O_2F_5S^+$ [M+H]$^+$ found 349.0322 requires 349.0316 (+1.7 ppm).

(3*S*,4*R*)-4-Methyl-3-phenyl-4-(trifluoromethyl)oxetan-2-one 35

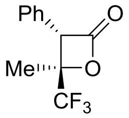

Following a modification to General Procedure D (change in equivalencies and temperature), trifluoroacetone (90 µL, 1 mmol), anhydride **17** (102 mg, 0.4 mmol), HyperBTM (6.2 mg, 0.02 mmol) and *i*-Pr$_2$NEt (87 µL, 0.5 mmol) in CH_2Cl_2 at 0 °C, gave, after Biotage chromatography (0→3% Et_2O in hexane), (3*S*,4*R*)-4-methyl-3-phenyl-4-(trifluoromethyl)oxetan-2-one **35** as a colourless oil (84 mg, 91%). *Note: product moderately volatile – can be lost if left under high vacuum for long periods.*

$[\alpha]_D^{20}$ = +104.0 (*c* 1.0, $CHCl_3$); **Chiral HPLC analysis**, Chiralcel OD-H (97:3 Hexane:IPA, flow rate 1.5 mLmin^{-1}, 254 nm, 40 °C) t_R(3*R*,4*S*): 2.9 min, t_R(3*S*,4*R*): 3.3 min, 93:7 er; **^{1}H NMR** (400 MHz, $CDCl_3$) δ_H:

1.35 (3H, q, *J* 0.9, CH_3), 5.17 (1H, s, C(3)*H*), 7.19–7.25 (2H, m, PhC(2,6)*H*), 7.35–7.48 (3H, m, PhC(3,4,5)*H*); **^{13}C{^{1}H} NMR** (126 MHz, $CDCl_3$) δ_C: 15.3 (CH_3), 60.1 (*C*(3)H), 77.9 (q, *J* 33.6, *C*(4)), 124.2 (q, *J* 281, CF_3), 128.3 (Ph*C*(3,5)H), 128.8 (Ph*C*(1)), 129.2 (Ph*C*(4)H), 129.5 (Ph*C*(2,6)H), 165.8 (*C*(2)); **^{19}F NMR** (471 MHz, $CDCl_3$) δ_F −81.6 (CF_3); **IR** (neat) ν_{max} cm^{-1}: 1848 (CO), 1456, 1159, 1098, 1042; **HRMS** (APCI$^+$) $C_{11}H_{10}O_2F_3$ $[M+H]^+$ found 231.0633 requires 231.0633 (+0.0 ppm).

(3*S*,4*S*)-4-(4-Bromophenyl)-3-(4-dimethylaminophenyl)-4-(perfluorobutyl)oxetan-2-one 36

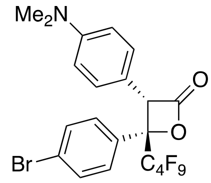

Following General Procedure C, ketone **18** (100 mg, 0.25 mmol), anhydride **S1** (213 mg, 0.63 mmol), HyperBTM (4 mg, 0.0125 mmol) *i*-Pr_2NEt (54µL, 0.31 mmol) in CH_2Cl_2 gave, after Biotage chromatography (0→5% Et_2O in hexane), (3*S*,4*S*)-4-(4-bromophenyl)-3-(4-dimethylaminophenyl)-4-(perfluorobutyl)oxetan-2-one **36** as a colourless oil (130.1 mg, 92%) as an inseparable mixture of diastereoisomers (> 95:5 dr).

$[\alpha]_D^{20}$ = +139.1 (*c* 1.0, $CHCl_3$); **Chiral HPLC analysis**, Chiralcel OD-H (97:3 Hexane:IPA, flow rate 1.5 mLmin^{-1}, 211 nm, 40 °C) t_R(3*R*,4*R*): 3.8 min, t_R(3*S*,4*S*): 4.6 min, 97:3 er; **^{1}H NMR** (500 MHz, $CDCl_3$) δ_H: 2.90 (6H, s, N(CH_3)$_2$), 5.52 (1H, s, C(3)*H*), 6.46–6.51 (2H, m, C(3)ArC(3,5)*H*), 6.65–6.71 (2H, m, C(3)ArC(2,6)*H*), 6.94–7.01 (2H, m, C(4)ArC(2,6)*H*), 7.29–7.41 (2H, m, C(4)Ar(3,5)*H*); *Minor diastereoisomer (selected signals)* δ_H: 3.00 (6H, s, NCH_3), 5.18 (1H, s, C(3)*H*); **^{13}C{^{1}H} NMR** (126 MHz, $CDCl_3$) δ_C: 40.2 (N(CH_3)$_2$), 64.4 (d, $^3J_{CF}$ 4.4, *C*(3)), 81.2 (dd, $^2J_{CF}$ 31.5, 23.4, *C*(4)), 108.0–119.0 (m, 3 x CF_2 & CF_3), 112.1 (C(3)Ar*C*(3,5)H), 114.5 (C(3)Ar*C*(1)), 124.1 (C(4)Ar*C*(1)), 128.2 (C(4)Ar*C*(4)), 129.4 (C(4)Ar*C*(2,6)H), 130.7 (C(3)Ar*C*(2,6)H), 131.4 (C(4)Ar*C*(3,5)H), 150.8 (C(3)Ar*C*(4)), 167.3 (*C*(2)); **^{19}F{^{1}H} NMR** (471 MHz, $CDCl_3$) δ_F: −127.9 – −124.5 (m, CF_2), −122.8 – −121.7 (m, CF_2), −118.6 – −116.1 (m, CF_2), −82.5 – −78.5 (m, CF_3); **IR** (neat) ν_{max} cm^{-1}: 1854 (C=O), 1624, 1528, 932, 864; **HRMS** (APCI$^+$) $C_{21}H_{16}NF_9O_2Br^+$ $[M+H]^+$ found 564.0221, requires 564.0215 (−1.1 ppm).

(3*S*,4*S*)-4-(4-Bromophenyl)-3-(4-methoxyphenyl)-4-(perfluorobutyl)oxetan-2-one 37

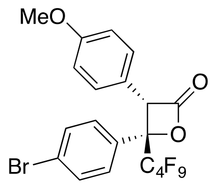

Following General Procedure C, ketone **18** (101 mg, 0.25 mmol), anhydride **S2** (194 mg, 0.625 mmol), HyperBTM (4 mg, 0.0125 mmol) and *i*-Pr_2NEt (54 µL, 0.31 mmol) in CH_2Cl_2 gave, after Biotage

chromatography (0→2% Et_2O in hexane), (3*S*,4*S*)-4-(4-bromophenyl)-3-(4-methoxyphenyl)-4-(perfluorobutyl)oxetan-2-one **37** as a colourless oil (111 mg, 81%) as an inseparable mixture of diastereoisomers (> 95:5 dr).

$[\alpha]_D^{20}$ = +127.0 (*c* 1.0, $CHCl_3$); **Chiral HPLC analysis**, Chiralcel OD-H (97:3 hexane:IPA, flow rate 1.5 mLmin^{-1}, 211 nm, 40 °C), t_R(3*R*,4*R*): 3.3 min, t_R(3*S*,4*S*): 3.8 min, 97:3 er; **^{1}H NMR** (500 MHz, $CDCl_3$) δ_H: 3.75 (3H, s, OC*H*$_3$), 5.56 (1H, s, C(3)*H*), 6.66–6.74 (2H, m, C(3)ArC(3,5)*H*), 6.74–6.82 (2H, m, C(3)ArC(2,6)*H*), 6.88–7.02 (2H, m, C(4)ArC(2,6)*H*), 7.32–7.41 (2H, m, C(4)ArC(3,5)*H*); **^{13}C{^{1}H} NMR** (126 MHz, $CDCl_3$) δ_C: 55.4 (O*C*H$_3$), 64.0 (*C*(3)H), 81.1 (dd, $^2J_{CF}$ 31.3, 23.6, *C*(4)), 105.9–119.9 (*C*F$_2$*C*F$_2$*C*F$_2$*C*F$_3$), 114.5 (C(3)Ar*C*(3,5)H), 119.9 (C(3)Ar*C*(1)), 124.3 (C(4)Ar*C*(1)), 127.9 (C(4)Ar*C*(4)), 129.3 (C(4)Ar*C*(2,6)H), 131.1 (C(3)Ar*C*(2,6)H), 131.5 (C(4)Ar*C*(3,5)H), 160.3 (C(3)Ar*C*(4)), 166.5 (*C*(2)); **^{19}F NMR** (471 MHz, $CDCl_3$) δ_F: −128.7 – −124.3 (m, C*F*$_2$), −122.9 – −121.5 (m, C*F*$_2$), −118.8 – −115.9 (m, C*F*$_2$), −80.8 (t, *J* 9.9, C*F*$_3$). **IR** (neat) ν_{max} cm^{-1}: 1854 (CO).

(3*S*,4*S*)-4-(4-Bromophenyl)-3-(4-fluorophenyl)-4-(perfluorobutyl)oxetan-2-one 38

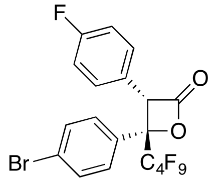

Following General Procedure C, ketone **18** (101 mg, 0.25 mmol), anhydride **S3** (180 mg, 0.625 mmol), HyperBTM (4 mg, 0.0125 mmol) and *i*-Pr_2NEt (54 µL, 0.31 mmol) in CH_2Cl_2 gave, after Biotage chromatography (0→2% Et_2O in hexane), (3*S*,4*S*)-4-(4-bromophenyl)-3-(4-fluorophenyl)-4-(perfluorobutyl)oxetan-2-one **38** as a colourless oil (96 mg, 71%) as an inseparable mixture of diastereoisomers (> 95:5 dr).

$[\alpha]_D^{20}$ = +102.0 (*c* 1.0, $CHCl_3$); **Chiral HPLC analysis**, Chiralcel OD-H (97:3 hexane:IPA, flow rate 1.5 mLmin^{-1}, 211 nm, 40 °C), t_R(3*R*,4*R*): 2.8 min, t_R(3*S*,4*S*): 3.1 min, 96:4 er; **^{1}H NMR** (500 MHz, $CDCl_3$) δ_H: 5.60 (1H, s, C(3)*H*), 6.82–6.88 (2H, m, C(3)ArC(2,6)*H*), 6.89–7.00 (4H, m, C(3)ArC(3,5)*H* + C(4)Ar(2,6)*H*), 7.33–7.40 (2H, m, C(4)ArC(3,5)*H*); **^{13}C{^{1}H} NMR** (126 MHz, $CDCl_3$) δ_C: 63.8 (*C*(3)H), 81.0 (dd, $^2J_{CF}$ 31.4, 23.5, *C*(4)), 105.6–121.3 (*C*F$_2$*C*F$_2$*C*F$_2$*C*F$_3$), 116.4 (d, $^2J_{CF}$ 22.2, C(3)ArC(3,5)H), 124.0 (d, $^3J_{CF}$ 3.3, C(3)Ar*C*(2,6)H), 124.5 (C(4)Ar*C*(1)), 127.5 (C(4)Ar*C*(4)), 129.1 (C(4)Ar*C*(2,6)H), 131.5 (C(4)Ar*C*(3,5)H), 132.5 (C(3)Ar*C*(1)), 163.2 (d, $^1J_{CF}$ 250.5, C(3)Ar*C*(4)), 165.8 (*C*(2)); **^{19}F NMR** (471 MHz, $CDCl_3$) δ_F: −127.9 – −124.5 (m, C*F*$_2$), −122.8 – −121.4 (m, C*F*$_2$), −118.3 – −116.0 (m, C*F*$_2$), −110.6 (m, Ar*F*) −80.9 – −80.8 (m, C*F*$_3$); **IR** (neat) ν_{max} cm^{-1}: 1856 (CO); **HRMS** (APCI$^+$) $C_{19}H_{10}BrF_{10}O_2^+$ $[M+H]^+$ found 538.9716 requires 538.9699 (+3.2 ppm).

(3*S*,4*S*)-3,4-Bis(4-bromophenyl)-4-(perfluorobutyl)oxetan-2-one 39

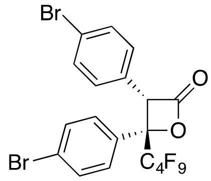

Following General Procedure C, ketone **18** (101 mg, 0.25 mmol), anhydride **S4** (255 mg, 0.625 mmol), HyperBTM (4 mg, 0.0125 mmol) and *i*-Pr_2NEt (54 μL, 0.31 mmol) in CH_2Cl_2 gave, after Biotage chromatography (0→2% Et_2O in hexane), (3*S*,4*S*)-3,4-bis(4-bromophenyl)-4-(perfluorobutyl)oxetan-2-one **39** as a colourless oil (82 mg, 55%), as an inseparable mixture of diastereoisomers (> 95:5 dr).

$[\alpha]_D^{20}$ = +115.0 (*c* 1.0, $CHCl_3$); **Chiral HPLC analysis**, Chiralpak AD-H (99:1 hexane:IPA, flow rate 1.5 mLmin^{-1}, 211 nm, 40 °C) t_R(3*S*,4*S*): 3.6 min, t_R(3*R*,4*R*): 4.1 min, 95:5 er; **^{1}H NMR** (500 MHz, $CDCl_3$) δ_H: 5.56 (1H, s, C(3)*H*), 6.72–6.78 (2H, m, C(3)Ar*C*(2,6)*H*), 6.92–7.01 (2H, m, C(4)ArC(2,6)*H*), 7.33–7.42 (4H, m, C(3)ArC(3,5)*H* + C(4)ArC(3,5)*H*); **^{13}C{^{1}H} NMR** (126 MHz, $CDCl_3$) δ_C: 63.9 (*C*(3)H), 80.8 (dd, $^2J_{CF}$ 31.6, 23.4, *C*(4)), 105.2–120.0 ($CF_2CF_2CF_2CF_3$), 124.1 (C(3)Ar*C*(1)), 124.7 (C(4)Ar*C*(1)), 127.0 (C(3)Ar*C*(4)), 127.4 (C(4)Ar*C*(4)), 129.1 (C(4)Ar*C*(2,6)H), 131.3 (C(3)Ar*C*(2,6)H), 131.8 (C(3Ar*C*(3,5)H), 132.4 (C(4)Ar*C*(3,5)H), 165.3 (*C*(2)); **^{19}F NMR** (471 MHz, $CDCl_3$) δ_F: −128.0 – −124.4 (m, CF_2), −122.9 – −121.5 (m, CF_2), −118.4 – −115.9 (m, CF_2), −80.8 – −80.8 (m, CF_3); **IR** (neat) ν_{max} cm^{-1}: 1711 (CO); **HRMS** (ESI$^-$) $C_{19}H_8Br_2F_9O_2^-$ [M−H]$^-$ found 596.8759, requires 596.8753 (+1.0 ppm).

(3*S*,4*S*)-4-(4-Bromophenyl)-4-(perfluorobutyl)-3-(4-(trifluoromethyl)phenyl)oxetan-2-one 40

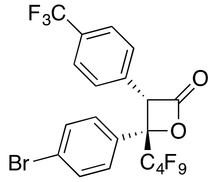

Following General Procedure C, ketone **18** (101 mg, 0.25 mmol), anhydride **S5** (242 mg, 0.625 mmol), HyperBTM (4 mg, 0.0125 mmol) and *i*-Pr_2NEt (54 μL, 0.31 mmol) in CH_2Cl_2 gave, after Biotage chromatography (0→2% Et_2O in hexane), (3*S*,4*S*)-4-(4-bromophenyl)-4-(perfluorobutyl)-3-(4-(trifluoromethyl)phenyl)oxetan-2-one **40** as a colourless oil (18 mg, 12%) as an inseparable mixture of diastereoisomers (> 95:5 dr).

$[\alpha]_D^{20}$ = +53.0 (*c* 1.0, $CHCl_3$); **Chiral HPLC analysis**, Chiralpak AD-H (99:1 hexane:IPA, flow rate 1.5 mLmin^{-1}, 211 nm, 40 °C), t_R(3*S*,4*S*): 3.2 min, t_R(3*R*,4*R*): 4.0 min, 95:5 er; **^{1}H NMR** (500 MHz, $CDCl_3$) δ_H: 5.66 (1H, s, C(3)*H*), 6.90–6.99 (2H, m, C(4)ArC(2,6)*H*), 7.00–7.06 (2H, m, C(3)ArC(2,6)*H*), 7.33–7.41 (2H, m, C(4)ArC(3,5)*H*), 7.47–7.53 (2H, m, C(3)ArC(3,5)*H*); *Minor diastereoisomer (selected signals)* δ_H: 5.30 (1H, s, C(3)*H*); **^{13}C{^{1}H} NMR** (126 MHz, $CDCl_3$) δ_C: 63.9 (d, $^3J_{CF}$ 4.7, *C*(3)H), 80.9 (dd, $^2J_{CF}$ 31.1, 24.0, *C*(4)), 106.2–121.3 ($CF_2CF_2CF_2CF_3$), 123.6 (q, *J* 272.5, ArCF_3), 124.8 (C(4)Ar*C*(4)), 126.1 (q, $^3J_{CF}$ 3.7,

C(3)ArC(3,5)H), 127.2 (d, $^3J_{CF}$ 2.9, C(4)CAr(1)), 129.0 (C(4)ArC(2,6)H), 130.3 (C(3)ArC(2,6)H), 131.8 (C(4)ArC(3,5)H), 131.8 (q, $^2J_{CF}$ 33.3, C(3)ArC(4)), 132.0 (C(3)CAr(1)), 164.9 (C(2)); **^{19}F NMR** (471 MHz, $CDCl_3$) δ_F: −127.9 – −124.5 (m, CF_2), −122.9 – −121.2 (m, CF_2), −118.3 – −115.8 (m, CF_2), −80.9 – −80.7 (m, CF_3), –63.0 ($ArCF_3$); *Minor diastereoisomer (selected signals)* δ_F: −80.9 – −80.9 (m, CF_3), −62.9 ($ArCF_3$); **IR** (neat) ν_{max} cm^{-1}: 1857 (CO); **HRMS** (ESI^-) $C_{20}H_8BrF_{12}O_2^-$ $[M-H]^-$ found 586.9527, requires 586.9522 (+0.9 ppm).

(3S,4S)-4-(4-Bromophenyl)-4-(perfluorobutyl)-3-(*m*-tolyl)oxetan-2-one 41

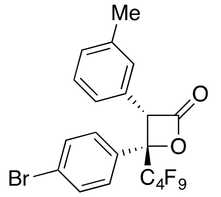

Following General Procedure C, ketone **18** (101 mg, 0.25 mmol), anhydride **S6** (175 mg, 0.625 mmol), HyperBTM (4 mg, 0.0125 mmol) and *i*-Pr_2NEt (54 µL, 0.31 mmol) in CH_2Cl_2 gave, after Biotage chromatography (0→2% Et_2O in hexane), (3*S*,4*S*)-4-(4-bromophenyl)-4-(perfluorobutyl)-3-(*m*-tolyl)oxetan-2-one **41** as a yellow oil (112 mg, 84%) as an inseparable mixture of diastereoisomers (> 95:5 dr).

$[\alpha]_D^{20}$ = +122.0 (*c* 1.0, $CHCl_3$); **Chiral HPLC analysis**, Chiralcel OD-H (97:3 hexane:IPA, flow rate 1.5 mLmin^{-1}, 211 nm, 40 °C), t_R(3*R*,4*R*): 2.6 min, t_R(3*S*,4*S*): 3.3 min, 97:3 er; **^{1}H NMR** (500 MHz, $CDCl_3$) δ_H: 2.20 (3H, s, CH_3), 5.56 (1H, s, C(3)*H*), 6.63–6.68 (2H, m, C(3)Ar(2,6)*H*), 6.90–7.01 (2H, m, C(4) ArC(2,6)*H*), 7.04–7.11 (2H, m, C(3)ArC(4,5)*H*), 7.30–7.37 (2H, m, C(4)ArC(3,5)*H*); *Minor diastereoisomer (selected signals)* δ_H: 2.41 (3H, s, CH_3), 5.23 (1H, s, C(3)*H*); **^{13}C{^{1}H} NMR** (126 MHz, $CDCl_3$) δ_C: 21.3 (CH_3), 64.6 (C(3)H), 81.0 (dd, $^2J_{CF}$ 31.3, 23.6, C(4)), 105.5–121.2 ($CF_2CF_2CF_2CF_3$), 124.2 (C(4)ArC(1)), 126.8 (C(3)ArC(6)H), 127.9 (C(3)ArC(4)), 127.9 (C(4)ArC(4)), 129.0 (C(3)ArC(5)H), 129.2 (C(4)ArC(2,6)H), 130.3 (C(3)ArC(4)H), 130.4 (C(3)ArC(2)H), 131.4 (C(4)ArC(3,5)H), 138.9 (C(3)ArC(3)), 166.1 (C(2)); **^{19}F NMR** (471 MHz, $CDCl_3$) δ_F: −128.0 – −124.6 (m, CF_2), −122.9 – −121.3 (m, CF_2), −118.4 – −116.0 (m, CF_2), −80.9 – −80.8 (m, CF_3); **IR** (neat) ν_{max} cm^{-1}: 1856 (CO); **HRMS** ($APCI^+$) $C_{20}H_{13}BrF_9O_2^+$ $[M+H]^+$ found 534.9959, required 534.9950 (+1.7 ppm).

(3*S*,4*S*)-4-(4-Bromophenyl)-4-(perfluorobutyl)-3-(*o*-tolyl)oxetan-2-one 42

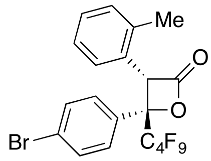

Following General Procedure C, ketone **18** (101 mg, 0.25 mmol), anhydride **S7** (175 mg, 0.625 mmol), HyperBTM (4 mg, 0.0125 mmol) and *i*-Pr_2NEt (54 µL, 0.31 mmol) in CH_2Cl_2 gave, after Biotage chromatography (0→2% Et_2O in hexane), (3*S*,4*S*)-4-(4-bromophenyl)-4-(perfluorobutyl)-3-(*o*-tolyl)oxetan-2-one **42** as a colourless oil (105 mg, 79%) as an inseparable mixture of diastereoisomers (> 95:5 dr).

$[\alpha]_D^{20}$ = +176.0 (*c* 1.0, $CHCl_3$); **Chiral HPLC analysis**, Chiralcel OD-H (97:3 hexane:IPA, flow rate 1.5 mLmin^{-1}, 211 nm, 40 °C), t_R(3*R*,4*R*): 2.8 min, t_R(3*S*,4*S*): 3.8 min, 99:1 er; **^{1}H NMR** (500 MHz, $CDCl_3$) δ_H: 2.39 (3H, s, C*H*$_3$), 5.80 (1H, s, C(6)*H*), 6.3-7.6 (2H, very broad, C(4)ArC(2,6)*H*), 6.46–6.52 (1H, m, C(3)ArC(6)*H*), 6.86–6.92 (1H, C(3)Ar(5)*H*), 7.12–7.19 (2H, m, C(3)ArC(3,4)*H*), 7.21–7.43 (2H, br, C(4)ArC(3,5)*H*); **^{13}C{^{1}H} NMR** (126 MHz, $CDCl_3$) δ_C: 19.6 (*C*H$_3$), 61.5 (d, $^3J_{CF}$ 4.6, *C*(3)H), 81.4 (dd, $^2J_{CF}$ 32.4, 23.8, *C*(4)), 105.5–121.3 (*C*F$_2$*C*F$_2$*C*F$_2$*C*F$_3$), 124.3 (C(4)Ar*C*(1)), 126.6 (C(3)Ar*C*(1)), 126.7 (C(3)Ar*C*(5)H), 128.0 (C(4)Ar*C*(4)), 128.9 (C(4)Ar*C*(2,6)H), 129.7 (C(3)Ar*C*(5)H), 130.2 (C(3)Ar*C*(6)H), 131.0 (C(3)Ar*C*(3)H), 131.5 (C(4)Ar*C*(3,5)H), 136.9 (C(3)Ar*C*(2)), 166.8 (*C*(2)); ^{19}F (471 MHz, $CDCl_3$) δ_F : −128.1 – −124.3 (m, C*F*$_2$), −123.0 – −121.6 (m, C*F*$_2$), −118.1 – −114.2 (m, C*F*$_2$), −80.9 – −80.8 (m, C*F*$_3$); **IR** (neat) ν_{max} cm^{-1}: 1857 (CO); **HRMS** (APCI$^+$) $C_{20}H_{13}BrF_9O_2^+$ [M+H]$^+$ found 534.9951, required 534.9950 (+0.2 ppm).

(3*S*,4*S*)-4-(4-Bromophenyl)-4-(perfluorobutyl)-3-(thiophen-3-yl)oxetan-2-one 43

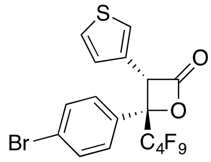

Following General Procedure C, ketone **18** (101 mg, 0.25 mmol), anhydride **S8** (165 mg, 0.625 mmol), HyperBTM (4 mg, 0.0125 mmol) and *i*-Pr_2NEt (54 µL, 0.31 mmol) in CH_2Cl_2 gave, after Biotage chromatography (0→2% Et_2O in hexane), (3*S*,4*S*)-4-(4-bromophenyl)-4-(perfluorobutyl)-3-(thiophen-3-yl)oxetan-2-one **43** as a colourless oil (114 mg, 87%) as an inseparable mixture of diastereoisomers (> 95:5 dr).

$[\alpha]_D^{20}$ = +94.0 (*c* 1.0, $CHCl_3$); **Chiral HPLC analysis**, Chiralcel OD-H (97:3 hexane:IPA, flow rate 1.5 mLmin^{-1}, 211 nm, 40 °C), t_R(3*R*,4*R*): 3.1 min, t_R(3*S*,4*S*): 3.8 min, 94:6 er; **^{1}H NMR** (500 MHz, $CDCl_3$) δ_H: 5.68 (1H, s, C(3)*H*), 6.48 (1H, dd, *J* 5.1, 1.3, C(3)ArC(4)*H*), 6.95–7.06 (3H, m, C(3)ArC(2)*H* + C(4)ArC(2,6)*H*), 7.18 (1H, dd, *J* 5.1, 2.9, C(3)ArC(5)*H*), 7.35–7.41 (2H, m, C(4)ArC(3,5)*H*); **^{13}C{^{1}H} NMR**

(126 MHz, $CDCl_3$) δ_C: 59.5 (*C*(3)), 80.8 (dd, *J* 31.5, 23.8, *C*(4)), 104.5–121.5 ($CF_2CF_2CF_2CF_3$), 124.5 (C(4)Ar*C*(1)), 127.0 (C(3)Ar*C*(2)H), 127.38 (C(3)Ar*C*(5)H), 127.40 (C(3)Ar*C*(4)), 127.8 (C(3)Ar*C*(3)), 127.9 (C(4)Ar*C*(4)), 128.9 (C(4)Ar*C*(2,6)H), 131.6 (C(4)Ar*C*(3,5)H), 165.8 (*C*(2)); **^{19}F NMR** (471 MHz, $CDCl_3$) δ_F : −128.3 – −124.0 (m, CF_2), −123.3 – −121.2 (m, CF_2), −118.7 – −116.0 (m, CF_2), −80.9 – −80.8 (m, CF_3); **IR** (neat) ν_{max} cm^{-1}: 1854 (CO); HRMS (APCI+) $C_{17}H_9BrF_9O_2S$ $[M+H]^+$ found 526.9360 requires 526.9357 (+0.6 ppm).

(3*S*,4*S*)-4-(4-Bromophenyl)-3-(1-methyl-1*H*-indol-3-yl)-4-(perfluorobutyl)oxetan-2-one 44

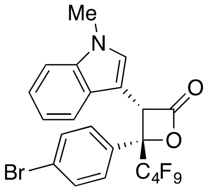

Following General Procedure C, ketone **18** (101 mg, 0.25 mmol), anhydride **S9** (223 mg, 0.625 mmol), HyperBTM (4 mg, 0.0125 mmol) and *i*-Pr_2NEt (54 µL, 0.31 mmol) in CH_2Cl_2 gave, after Biotage chromatography (0→2% Et_2O in hexane), (3*S*,4*S*)-4-(4-bromophenyl)-3-(1-methyl-1*H*-indol-3-yl)-4-(perfluorobutyl)oxetan-2-one **44** as a colourless solid (79 mg, 55%) as an inseparable mixture of diastereoisomers (> 95:5 dr).

m.p. 112–114 °C; $[\alpha]_D^{20} =$ +126.0 (*c* 0.5, $CHCl_3$); **Chiral HPLC analysis**, Chiralpak AS-H (99:1 hexane:IPA, flow rate 1.5 $mLmin^{-1}$, 211 nm, 40 °C), t_R(3*R*,4*R*): 3.9 min, t_R(3*S*,4*S*): 5.2 min, 99:1 er; **^{1}H NMR** (500 MHz, $CDCl_3$) δ_H: 3.64 (3H, s, C*H*$_3$), 5.90 (1H, s, C(3)*H*), 6.60 (1H, s, C(3)ArC(2)*H*), 6.98 (2H, br, C(4)ArC(2,6)*H*), 7.12–7.17 (1H, m, C(3)ArC(5)*H*), 7.20–7.30 (4H, m, C(3)ArC(6,7)*H* + C(4)ArC(3,5)*H*), 7.39 (1H, d, *J* 8.2, C(3)ArC(4)*H*); **^{13}C{^{1}H} NMR** (126 MHz, $CDCl_3$) δ_C: 33.2 (*C*H$_3$), 56.7 (*C*(3)H), 81.7 (dd, *J* 31.8, 23.5, *C*(4)), 101.1 (C(3)Ar*C*(3)), 110.0 (C(3)Ar*C*(6)H), 117.9 (C(3)Ar*C*(4)H), 120.6 (C(3)Ar*C*(5)H), 122.7 (C(3)Ar*C*(7)H), 124.1 (C(4)Ar*C*(1)), 126.8 (C(3)*C*Ar(3a)), 128.4 (C(4)Ar*C*(4)), 129.1 (C(4)Ar*C*(2,6)H), 129.6 (C(3)Ar*C*(2)H), 131.3 (C(4)Ar*C*(3,5)H), 136.9 (C(3)Ar*C*(7a)), 167.5 (*C*(2)), C_4F_9 not observed; **^{19}F NMR** (471 MHz, $CDCl_3$) δ_F : −128.0 – −124.4 (m, CF_2), −122.9 – −121.8 (m, CF_2), −118.4 – −115.6 (m, CF_2), −80.9 – −80.7 (m, CF_3); **IR** (neat) ν_{max} cm^{-1}: 1852 (CO); **HRMS** ($APCI^+$) $C_{22}H_{14}NBrF_9O_2^+$ $[M+H]^+$ found 574.0078, required 574.0059 (+3.3 ppm).

(3*S*,4*S*)-4-(4-Bromophenyl)-4-(perfluorobutyl)-3-((*E*)-prop-1-en-1-yl)oxetan-2-one 45

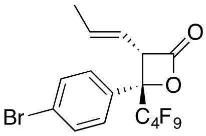

Following General Procedure C, ketone **18** (101 mg, 0.25 mmol), anhydride **S10** (115 mg, 0.625 mmol), HyperBTM (4 mg, 0.0125 mmol) and *i*-Pr_2NEt (54 µL, 0.31 mmol) in CH_2Cl_2 gave, after Biotage

chromatography (0→2% Et_2O in hexane), (3*S*,4*S*)-4-(4-bromophenyl)-4-(perfluorobutyl)-3-((*E*)-prop-1-en-1-yl)oxetan-2-one **45** as a colourless oil (101 mg, 83%) as an inseparable mixture of diastereoisomers (83:17 dr).

$[\alpha]_D^{20}$ = +11.0 (*c* 1.0, $CHCl_3$); **Chiral HPLC analysis**, Inseparable by HPLC, er obtained following ring opening with benzylamine (see below); **^{1}H NMR** (500 MHz, $CDCl_3$) δ_H: 1.66 (3H, dd, *J* 6.6, 1.3, C*H_3*), 4.84–4.94 (2H, m, C(3)*H* + C*H*=CHCH$_3$), 5.80–5.90 (1H, m, CH=C*H*CH$_3$), 7.22–7.28 (2H, m, C(4)ArC(2,6)*H*), 7.56–7.62 (2H, m, C(4)ArC(3,5)*H*); *Minor diastereoisomer (selected signals)* δ_H: 1.84 (3H, dd, *J* 6.3, 1.4, C*H_3*), 4.51 (1H, d, *J* 8.7, C(3)*H*), 5.96 (1H, dq, *J* 15.2, 6.3, CH=C*H*CH$_3$), 7.32–7.37 (2H, m, C(4)ArC(2,6)*H*); **^{13}C{^{1}H} NMR** (126 MHz, $CDCl_3$) δ_C: 18.2 (*C*H$_3$), 61.8 (*C*(3)H), 79.8 (dd, $^2J_{CF}$ 30.6, 23.4, *C*(4)), 105.3–121.6 (*C*F$_2$*C*F$_2$*C*F$_2$*C*F$_3$), 118.0 (*C*H=CHCH$_3$), 124.6 (C(4)Ar*C*(4)), 128.2 (C(4)Ar*C*(1)), 129.1 (C(4)Ar*C*(2,6)H), 131.9 (C(4)Ar*C*(3,5)H), 136.9 (CH=*C*HCH$_3$), 166.1 (*C*(2)); *Minor diastereoisomer (selected signals)* δ_C: 18.3 (*C*H$_3$), 68.6 (*C*(3)H), 124.5 (C(4)Ar*C*(4)), 132.2 (C(4)Ar*C*(3,5)H), 137.0 (CH=*C*HCH$_3$), 166.0 (*C*(2)); **^{19}F NMR** (471 MHz, $CDCl_3$) δ_F: −128.1 – −124.6 (m, C*F*$_2$), −123.0 – −121.7 (m, C*F*$_2$), −118.5 – −116.3 (m, C*F*$_2$), −81.0 – −80.8 (m, C*F*$_3$); **IR** (neat) ν_{max} cm^{-1}: 1852 (CO); **HRMS** (APCI$^+$) $C_{16}H_{11}BrF_9O_2^+$ [M+H]$^+$ found 484.9798, required 484.9793 (+1.0 ppm).

(2*S*,3*S*)-*N*-Benzyl-3-(4-bromophenyl)-4,4,5,5,6,6,7,7,7-nonafluoro-3-hydroxy-2-((*E*)-prop-1-en-1-yl)heptanamide S19

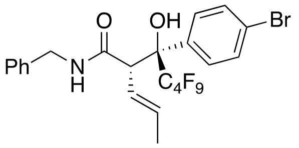

Benzylamine (50 µL, 0.5 mmol, 5.00 equiv.) was added to a solution of (3*S*,4*S*)-4-(4-bromophenyl)-4-(perfluorobutyl)-3-((*E*)-prop-1-en-1-yl)oxetan-2-one **45** (50 mg, 0.1 mmol, 1.00 equiv.) in CH_2Cl_2 (0.1 M) and allowed to stir at r.t. for 16 h. The mixture was diluted with CH_2Cl_2 and washed with 0.1 M HCl (3 × 10 mL) to give (2*S*,3*S*)-*N*-benzyl-3-(4-bromophenyl)-4,4,5,5,6,6,7,7,7-nonafluoro-3-hydroxy-2-((*E*)-prop-1-en-1-yl)heptanamide **S19** as a 90:10 mixture of diastereoisomers as a colourless crystalline solid (47 mg, 78%).

m.p. 96-98 °C; $[\alpha]_D^{20}$ = +12.0 (*c* 1.0, $CHCl_3$); **Chiral HPLC analysis**, Chiralpak AD-H (98:2 hexane:IPA, flow rate 1.5 mLmin^{-1}, 254 nm, 40 °C), t_R(3*R*,4*R*): 9.9 min, t_R(3*S*,4*S*): 13.2 min, 86:14 er; **^{1}H NMR** (500 MHz, $CDCl_3$) δ_H: 1.44 (3H, dd, *J* 6.4, 1.6, C*H_3*), 3.40 (1H, dd, *J* 9.0, 2.3, C(2)*H*), 4.45 (1H, dd, *J* 14.8, 5.7, NHC*H^A*H^B), 4.51 (1H, dd, *J* 14.8, 5.7, NHCH^A*H^B*), 5.18–5.26 (1H, m, C*H*=CHCH$_3$), 5.37 (1H, dq, *J* 15.4, 6.4, CH=C*H*CH$_3$), 6.11 (1H, app t, *J* 5.7, N*H*), 7.22–7.26 (2H, m, NHCH$_2$ArC(2,6)*H*), 7.29–7.38 (5H, m, NHCH$_2$ArC(3,4,5)*H* & C(3)ArC(2,6)*H*), 7.44–7.49 (2H, m, C(3)ArC(3,5)*H*); *Minor diastereoisomer (selected signals)* δ_H: 1.78 (3H, d, *J* 6.6, C*H_3*), 3.57–3.62 (1H, m, C(2)*H*); **^{13}C{^{1}H} NMR** (126 MHz, $CDCl_3$)

δ_C: 18.0 (C*H*$_3$), 43.9 (C*H*$_2$), 51.5 (*C*(2)H), 79.8 (t, *J* 22.3, *C*(3)), 105.7–120.1 (*C*F$_2$*C*F$_2$*C*F$_2$*C*F$_3$), 122.6 (C(3)Ar*C*(4)Br), 123.3 (*C*H=CHCH$_3$), 127.9 (NHCH$_2$Ar*C*(2,6)H), 128.1 (NHCH2Ar*C*(4)H), 128.5 (C(3)Ar*C*(2,6)H), 129.0 (NCH$_2$Ar*C*(3,5)H), 131.3 (C(3)Ar*C*(3,5)H), 132.4 (CH=*C*HCH$_3$), 135.0 (C(3)Ar*C*(1)), 137.0 (NHCH$_2$Ar*C*(1)), 173.6 (*C*=O); *Minor diastereoisomer (selected signals)* δ_C: 18.2 (C*H*$_3$), 43.3 (C*H*$_2$), 56.1 (*C*(2)H), 172.3 (*C*=O); **^{19}F{^{1}H} NMR** (471 MHz, CDCl$_3$) δ_F: −127.6 – −124.6 (m, C*F*$_2$), −120.9 – −117.4 (m, C*F*$_2$), −115.9 – −113.2 (m, C*F*$_2$), −80.9 – −80.8 (m, C*F*$_3$); **IR** (neat) ν_{max} cm^{-1}: 3291 (OH), 2918 (CH), 1626 (C=O), 1454, 1354, 1233, 1198, 1134, 1076, 1011, 964.

(3*S*,4*S*)-4-(4-Bromophenyl)-4-(perfluorobutyl)-3-(phenylthio)oxetan-2-one 46

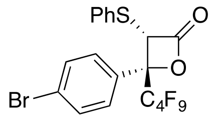

Following General Procedure C, ketone **18** (101 mg, 0.25 mmol), anhydride **S11** (199 mg, 0.625 mmol), HyperBTM (4 mg, 0.0125 mmol) and *i*-Pr$_2$NEt (54 µL, 0.31 mmol) in CH$_2$Cl$_2$ gave, after Biotage chromatography (0→5% Et$_2$O in hexane), (3*S*,4*S*)-4-(4-bromophenyl)-4-(perfluorobutyl)-3-(phenylthio)oxetan-2-one **46** as a colourless oil (49 mg, 35%) as an inseparable mixture of diastereoisomers (> 95:5 dr).

$[\alpha]_D^{20} = -55.2$ (*c* 0.5, CHCl$_3$); **Chiral HPLC analysis**, Chiralcel OD-H (99.5:0.5 Hexane:IPA, flow rate 1.5 mLmin^{-1}, 211 nm, 40 °C) t$_R$(3*R*,4*R*): 3.9 min, t$_R$(3*S*,4*S*): 4.4 min, 83:17 er; **^{1}H NMR** (500 MHz, CDCl$_3$) δ_H: 5.44 (1H, s, C(3)*H*), 7.21–7.26 (4H, m, PhC(2,3,5,6)*H*), 7.27–7.34 (3H, m, C(4)ArC(2,6)*H* + PhC(4)*H*), 7.56–7.60 (2H, m, C(4)ArC(3,5)*H*); *Minor diastereoisomer (selected signals)* δ_H: 5.02 (1H, s, C(3)*H*); **^{13}C{^{1}H} NMR** (126 MHz, CDCl$_3$) δ_C: 62.6 (d, *J* 4.4, *C*(3)H), 80.8 (dd, *J* 31.0, 23.8, *C*(4)), 105.7–121.2 (m, *C*F$_2$*C*F$_2$*C*F$_2$*C*F$_3$), 125.2 (C(4)Ar*C*(4)), 127.0 (C(4)Ar*C*(1)), 129.1 (C(4)Ar*C*(2,6)H), 129.2 (Ph*C*(4)H), 129.5 (Ph*C*(3,5)H), 130.1 (Ph*C*(1)), 131.8 (C(4)Ar*C*(3,5)H), 133.2 (Ph*C*(2,6)H), 164.3 (*C*(2)); *Minor diastereoisomer (selected signals)* δ_C: 69.5 (*C*(3)H); **^{19}F NMR** (471 MHz, CDCl$_3$) δ_F: −128.2 – −124.4 (m, C*F*$_2$), −122.9 – −120.4 (m, C*F*$_2$), −117.9 – −114.8 (m, C*F*$_2$), −80.9 – −80.7 (m, C*F*$_3$); **IR** (neat) ν_{max} cm^{-1}: 1869 (CO); **HRMS** (NSI$^+$) $C_{19}H_{11}BrF_9O_2S^+$ [M+H]$^+$ found 552.9528, requires 552.9514 (+2.5 ppm).

(3*S*,4*S*)-4-(4-Bromophenyl)-3-(4-dimethylaminophenyl)-4-(perfluoroethyl)oxetan-2-one 47

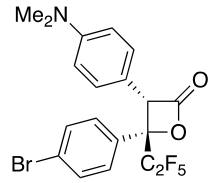

Following General Procedure C, ketone **19** (76 mg, 0.25 mmol), anhydride **S1** (213 mg, 0.625 mmol), HyperBTM (4 mg, 0.0125 mmol) and *i*-Pr_2NEt (54 µL, 0.31 mmol) in CH_2Cl_2 gave, after Biotage chromatography (0→5% Et_2O in hexane), (3*S*,4*S*)-4-(4-bromophenyl)-3-(4-dimethylaminophenyl)-4-(perfluoroethyl)oxetan-2-one **47** as a colourless solid (90 mg, 80%, >95:5 dr).

m.p. 81–83 °C; $[\alpha]_D^{20}$ = +177.8 (*c* 1.0, $CHCl_3$); **Chiral HPLC analysis**, Chiralcel OD-H (97:3 Hexane:IPA, flow rate 1.5 mLmin^{-1}, 211 nm, 40 °C) t_R(3*R*,4*R*): 4.0 min, t_R(3*S*,4*S*): 5.3 min, 96:4 er; **^{1}H NMR** (500 MHz, $CDCl_3$) δ_H: 2.90 (6H, s, N(C*H*$_3$)$_2$), 5.50 (1H, s, C(3)*H*), 6.45–6.50 (2H, m, C(3)-ArC(3,5)*H*), 6.65–6.70 (2H, m, C(3)ArC(2,6)*H*), 6.91–7.01 (2H, m, C(4)ArC(2,6)*H*), 7.33–7.39 (2H, m, C(4)ArC(3,5)*H*); *Minor diastereoisomer (selected signals)* δ_H: 2.99 (6H, s, NC*H*$_3$), 5.19 (1H, s, C(3)*H*); **^{13}C{^{1}H} NMR** (126 MHz, $CDCl_3$) δ_C: 40.2 (N(*C*H$_3$)$_2$), 63.8 (d, $^3J_{CF}$ 4.0, *C*(3)H), 80.3 (dd, $^2J_{CF}$ 30.6, 23.3, *C*(4)), 110.8–121.8 (m, *C*F$_2$*C*F$_3$), 112.2 (C(3)Ar*C*(3,5)H), 114.5 (C(3)Ar*C*(1)), 124.1 (C(4)Ar*C*(4)), 128.2 (d, $^3J_{CF}$ 2.9, C(4)Ar*C*(1)), 129.3 (C(4)Ar*C*(2,6)H), 130.7 (C(3)Ar*C*(2,6)H), 131.4 (C(4)Ar*C*(3,5)H), 150.8 (C(3)Ar*C*(4)), 167.2 (*C*(2)); *Minor diastereoisomer (selected signals)* δ_C: 69.4 (*C*(3)); **^{19}F{^{1}H} NMR** (471 MHz, $CDCl_3$) δ_F: −124.8 (d, *J* 281.5, C*F*$_2$), −122.0 (d, *J* 281.3, C*F*$_2$), −79.0 (C*F*$_3$); *Minor diastereoisomer (selected signals)* δ_F: −79.5 (C*F*$_3$); **IR** (neat) ν_{max} cm^{-1}: 2895, 1850 (CO), 1614, 1528, 1194, 943; **HRMS** (APCI$^+$) $C_{19}H_{16}NO_2F_5Br^+$ $[M+H]^+$ found 464.0285, requires 464.0279 (+1.3 ppm).

(3*S*,4*S*)-4-(4-Bromophenyl)-3-(4-methoxyphenyl)-4-(perfluoroethyl)oxetan-2-one 48

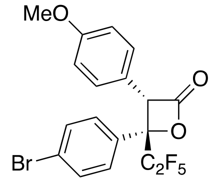

Following General Procedure C, ketone **19** (76 mg, 0.25 mmol), anhydride **S2** (197 mg, 0.63 mmol), HyperBTM (4 mg, 0.0125 mmol) and *i*-Pr_2NEt (54µL, 0.31 mmol) in CH_2Cl_2 gave, after Biotage chromatography (0→5% Et_2O in hexane), (3*S*,4*S*)-4-(4-bromophenyl)-3-(4-methoxyphenyl)-4-(perfluoroethyl)oxetan-2-one **48** as a colourless oil (106 mg, 94%, > 95:5 dr).

$[\alpha]_D^{20}$ = +148.2 (*c* 1.0, $CHCl_3$); **Chiral HPLC analysis**, Chiralcel OD-H (97:3 Hexane : IPA, flow rate 1.5 mLmin^{-1}, 211 nm, 40 °C) t_R(3*R*,4*R*): 3.6 min, t_R(3*S*,4*S*): 4.4 min, 96:4 er; **^{1}H NMR** (500 MHz, $CDCl_3$) δ_H: 3.74 (3H, s, OC*H*$_3$), 5.55 (1H, s, C(3)*H*), 6.69–6.74 (2H, m, C(3)ArC(3,5)*H*), 6.75–6.80 (2H, m, C(3)ArC(2,6)*H*), 6.90–6.98 (2H, m, C(4)ArC(2,6)*H*), 7.33–7.38 (2H, m, C(4)ArC(3,5)*H*); *Minor*

diastereoisomer (selected signals) δ_H: 3.84 (3H, s, OC*H*$_3$), 5.22 (1H, s, C(3)*H*); **^{13}C{^{1}H} NMR** (126 MHz, $CDCl_3$) δ_C: 55.4 (O*C*H$_3$), 63.5 (d, $^3J_{CF}$ 4.2, *C*(3)H), 80.1 (dd, $^2J_{CF}$ 30.6, 23.5, *C*(4)), 110.7–121.8 (*C*F$_2$*C*F$_3$), 114.5 (C(3)Ar*C*(2,6)H), 119.9 (C(3)Ar*C*(1)), 124.3 (C(4)Ar*C*(4)), 127.9 (d, $^3J_{CF}$ 3.0, C(4)Ar*C*(1)), 129.1 (d, $^4J_{CF}$ 2.5, C(4)Ar*C*(2,6)H), 131.1 (C(3)Ar*C*(3,5)H), 131.6 (C(4)Ar*C*(3,5)H), 160.2 (C(3)Ar*C*(4)), 166.5 (*C*(2)); *Minor diastereoisomer (selected signals)* δ_C: 68.8 (*C*(3)H), 165.7 (*C*(2)); **^{19}F{^{1}H} NMR** (471 MHz, $CDCl_3$) δ_F: −124.7 (d, *J* 281.7, C*F*$_2$), −121.8 (d, *J* 281.6, C*F*$_2$), −79.0 (C*F*$_3$); *Minor diastereoisomer (selected signals)* δ_F: −79.5 (C*F*$_3$); **IR** (neat) ν_{max} cm^{-1}: 1851 (CO), 1516, 1196, 810; **HRMS** (APCI$^+$) $C_{18}H_{13}O_3F_5Br^+$ $[M+H]^+$ found 450.9969, requires 450.9963 (+1.3 ppm).

(3*S*,4*S*)-4-(4-Bromophenyl)-3-(4-fluorophenyl)-4-(perfluoroethyl)oxetan-2-one 49

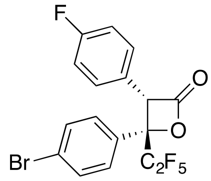

Following General Procedure C, ketone **19** (76 mg, 0.25 mmol), anhydride **S3** (182 mg, 0.625 mmol), HyperBTM (4 mg, 0.0125 mmol) and *i*-Pr$_2$NEt (54 µL, 0.31 mmol) in CH_2Cl_2 gave, after Biotage chromatography (0→5% Et$_2$O in hexane), **(**3*S*,4*S*)-4-(4-bromophenyl)-3-(4-fluorophenyl)-4-(perfluoroethyl)oxetan-2-one **49** as a colourless oil (98 mg, 89%, > 95:5 dr).

$[\alpha]_D^{20}$ = +135.2 (*c* 0.5, $CHCl_3$); **Chiral HPLC analysis**, Chiralcel OD-H (97:3 Hexane:IPA, flow rate 1.5 mLmin^{-1}, 211 nm, 40 °C) t_R(3*R*,4*R*): 3.0 min, t_R(3*S*,4*S*): 3.5 min, 95:5 er; **^{1}H NMR** (500 MHz, $CDCl_3$) δ_H: 5.59 (1H, s, C(3)*H*), 6.84–6.88 (2H, m, C(3)ArC(2,6)*H*), 6.88–6.97 (4H, m, C(4)ArC(2,6)*H* + C(3)ArC(3,5)*H*), 7.35–7.40 (2H, m, C(4)ArC(3,5)*H*); *Minor diastereoisomer (selected signals)* δ_H: 5.24 (1H, s, C(3)*H*); **^{13}C{^{1}H} NMR** (126 MHz, $CDCl_3$) δ_C: 63.3 (d, $^3J_{CF}$ 4.2, *C*(3)H), 80.0 (dd, $^2J_{CF}$ 30.6, 23.5, *C*(4)), 109.2–122.2 (*C*F$_2$*C*F$_3$), 116.4 (d, $^2J_{CF}$ 22.0, C(3)Ar*C*(3,5)H), 124.0 (d, $^4J_{CF}$ 3.5, C(3)Ar*C*(1)), 124.6 (C(4)Ar*C*(4)), 127.6 (d, $^3J_{CF}$ 2.8, C(4)Ar*C*(1)), 129.0 (d, $^4J_{CF}$ 2.7, C(4)Ar*C*(2,6)H), 131.6 (d, $^3J_{CF}$ 8.6, C(3)Ar*C*(2,6)H), 131.7 (C(4)Ar*C*(3,5)H), 163.2 (d, $^1J_{CF}$ 250.5, C(3)Ar*C*(4)), 165.7 (*C*(2)); *Minor diastereoisomer (selected signals)* δ_C: 68.4 (*C*(3)H); **^{19}F NMR** (471 MHz, $CDCl_3$) δ_F: −124.5 (d, *J* 282.7, C*F*$_2$), −121.7 (d, *J* 282.2, C*F*$_2$), −110.6 (Ar*F*), −79.0 (C*F*$_3$); *Minor diastereoisomer (selected signals)* δ_F: −79.5 (C*F*$_3$); **IR** (neat) ν_{max} cm^{-1}: 1857 (CO); **HRMS** (APCI$^+$) $C_{17}H_9BrF_6O_2^+$ $[M]^+$ found 437.9686 requires 437.9685 (+0.2 ppm).

(3*S*,4*S*)-3,4-Bis(4-bromophenyl)-4-(perfluoroethyl)oxetan-2-one 50

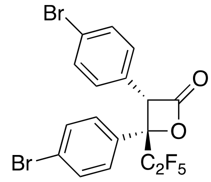

Following General Procedure C, ketone **19** (76 mg, 0.25 mmol), anhydride **S4** (258 mg, 0.63 mmol), HBTM (4 mg, 0.0125 mmol) and *i*-Pr_2NEt (54 µL, 0.31 mmol) in CH_2Cl_2 gave, after Biotage chromatography (0→3% Et_2O in hexane), (3*S*,4*S*)-3,4-bis(4-bromophenyl)-4-(perfluoroethyl)oxetan-2-one **50** as a colourless oil (81 mg, 65%, > 95:5 dr).

$[\alpha]_D^{20}$ = +139.3 (*c* 1.0, $CHCl_3$); **Chiral HPLC analysis**, Chiralcel OD-H (97:3 Hexane:IPA, flow rate 1.5 mLmin^{-1}, 211 nm, 40 °C) t_R(3*R*,4*R*): 3.6 min, t_R(3*S*,4*S*): 4.1 min, 95:5 er; **^{1}H NMR** (500 MHz, $CDCl_3$) δ_H: 5.55 (1H, s, C(3)*H*), 6.72–6.79 (2H, m, C(3)ArC(2,6)*H*), 6.88–7.01 (2H, m, C(4)ArC(2,6)*H*), 7.32–7.42 (4H, m, C(3)ArC(3,5)*H* + C(4)ArC(3,5)*H*); *Minor diastereoisomer (selected signals)* δ_H: 5.22 (1H, s, C(3)*H*); **^{13}C{^{1}H} NMR** (126 MHz, $CDCl_3$) δ_C: 63.3 (d, $^3J_{CF}$ 4.4 , *C*(3)H), 79.9 (dd, $^2J_{CF}$ 30.4, 23.6, *C*(4)), 110.2–122.0 (*C*F_2*C*F_3), 124.0 (C(3)Ar*C*(4)), 124.7 (C(4)Ar*C*(4)), 127.0 (C(3)Ar*C*(1)), 127.4 (d, $^3J_{CF}$ 3.1, C(4)Ar*C*(1)), 128.9 (d, $^4J_{CF}$ 2.6, C(4)Ar*C*(2,6)H), 131.3 (C(3)Ar*C*(2,6)H), 131.8 (C(3)Ar*C*(3,5)H), 132.4 (C(4)Ar*C*(3,5)H), 165.3 (*C*(2)); *Minor diastereoisomer (selected signals)* δ_C: 68.4 (*C*(3)); **^{19}F{^{1}H} NMR** (471 MHz, $CDCl_3$) δ_F: −124.4 (d, *J* 282.1, C*F*$_2$), −121.6 (d, *J* 282.1, C*F*$_2$), −78.9 (C*F*$_3$); *Minor diastereoisomer (selected signals)* δ_F: −79.4 (C*F*$_3$); **IR** (neat) ν_{max} cm^{-1}: 1850 (CO), 1491, 1150, 938, 806; **HRMS** (APCI$^+$) $C_{17}H_{10}F_5O_2Br_2^+$ $[M+H]^+$ found 498.8968, requires 498.8962 (+1.2 ppm).

(3*S*,4*S*)-4-(4-Bromophenyl)-4-(perfluoroethyl)-3-(thiophen-3-yl)oxetan-2-one 51

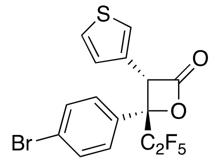

Following General Procedure C, ketone **19** (76 mg, 0.25 mmol), anhydride **S8** (167 mg, 0.63 mmol), HyperBTM (4 mg, 0.0125 mmol) and *i*-Pr_2NEt (54 µL, 0.31 mmol) in CH_2Cl_2 gave, after Biotage chromatography (0→3% Et_2O in hexane), (3*S*,4*S*)-4-(4-bromophenyl)-4-(perfluoroethyl)-3-(thiophen-3-yl)oxetan-2-one **51** as a colourless oil (85 mg, 80%, > 95:5 dr).

$[\alpha]_D^{20}$ = +93.3 (*c* 1.0, $CHCl_3$); **Chiral HPLC analysis**, Chiralcel OD-H (97:3 Hexane:IPA, flow rate 1.5 mLmin^{-1}, 211 nm, 40 °C) t_R(3*R*,4*R*): 3.4 min, t_R(3*S*,4*S*): 4.6 min, 93:7 er; **^{1}H NMR** (500 MHz, $CDCl_3$) δ_H: 5.68 (1H, s, C(3)*H*), 6.49 (1H, dd, *J* 5.0, 1.4, C(3)ArC(5)*H*), 6.95–7.02 (2H, m, C(4)ArC(2,6)*H*), 7.02 (1H, dd, *J* 3.0, 1.3, C(3)ArC(2)*H*), 7.18 (1H, dd, *J* 5.0, 2.9, C(3)ArC(4)*H*), 7.36–7.42 (2H, m, C(4)ArC(3,5)*H*); *Minor diastereoisomer (selected signals)* δ_H: 5.27 (1H, s, C(3)*H*); **^{13}C{^{1}H} NMR** (126 MHz, $CDCl_3$) δ_C:

59.0 (d, $^3J_{CF}$ 4.5, *C*(3)H), 79.8 (dd, $^2J_{CF}$ 30.6, 23.4, *C*(4)), 110.1–122.1 (*C*F_2*C*F_3), 124.5 (C(4)Ar*C*(4)), 126.9 (C(3)Ar*C*(2)H), 127.4 (C(3)Ar*C*(4)H), 127.4 (C(3)Ar*C*(5)H), 127.8 (C(3)Ar*C*(3)), 127.9 (d, $^3J_{CF}$ 3, C(4)Ar*C*(1)), 128.8 (d, $^4J_{CF}$ 2.5, C(4)Ar*C*(2,6)H), 131.6 (C(4)Ar*C*(3,5)H), 165.7 (*C*(2)); **^{19}F{^{1}H} NMR** (471 MHz, $CDCl_3$) δ_F: −124.6 (d, *J* 281.9, C*F*$_2$), −121.8 (d, *J* 281.9, C*F*$_2$), −80.0 (C*F*$_3$); *Minor diastereoisomer (selected signals)* δ_F: −79.3 (C*F*$_3$); **IR** (neat) ν_{max} cm^{-1}: 1856 (CO), 1343, 1090, 976, 814; **HRMS** (APCI$^+$) $C_{15}H_9O_2F_5SBr^+$ [M+H]$^+$ found 426.9425 requires 426.9421 (+0.9 ppm).

(3*S*,4*S*)-4-(4-Bromophenyl)-4-(perfluoroethyl)-3-((*E*)-prop-1-en-1-yl)oxetan-2-one 52

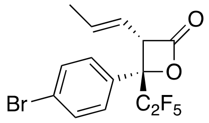

Following General Procedure C, ketone **19** (76 mg, 0.25 mmol), anhydride **S10** (114 mg, 0.63 mmol), HyperBTM (4 mg, 0.0125 mmol) and *i*-Pr_2NEt (54 µL, 0.31 mmol) in CH_2Cl_2 (crude dr 87:13) gave, after Biotage chromatography (0→2% Et_2O in hexane), (3*S*,4*S*)-4-(4-bromophenyl)-4-(perfluoroethyl)-3((*E*)-prop-1-en-1-yl)oxetan-2-one **52** as a colourless oil (84 mg, 87%, 89:11 dr). $[\boldsymbol{\alpha}]_{\boldsymbol{D}}^{\boldsymbol{20}}$ = +4.3 (*c* 1.0, $CHCl_3$); **Chiral HPLC analysis**, Chiralpak AD-H (99:1 Hexane:IPA, flow rate 1.5 mLmin^{-1}, 211 nm, 40 °C) t_R(3*S*,4*S*): 2.9 min, t_R(3*R*,4*R*): 3.3 min, 83:17 er; **^{1}H NMR** (500 MHz, $CDCl_3$) δ_H: 1.66 (3H, dd, *J* 6.6, 1.2, C*H*$_3$), 4.86–4.94 (2H, m, C(3)*H* + C*H*=CHCH$_3$), 5.80–5.90 (1H, m, CH=C*H*CH$_3$), 7.20–7.25 (2H, m, C(4)ArC(2,6)*H*), 7.56–7.62 (2H, m, C(4)ArC(3,5)*H*); *Minor diastereoisomer (selected signals)* δ_H: 1.84 (3H, dd, *J* 6.4, 1.6, C*H*$_3$), 5.92–6.01 (1H, m, CH=C*H*CH$_3$); **^{13}C{^{1}H} NMR** (126 MHz, $CDCl_3$) δ_C: 18.2 (*C*H$_3$), 61.3 (d, $^3J_{CF}$ 4.4, *C*(3)H), 78.9 (dd, $^2J_{CF}$ 30.2, 23.8, *C*(4)), 109.9–121.9 (*C*F$_2$*C*F$_3$), 118.1 (*C*H=CHCH$_3$), 124.6 (C(4)Ar*C*(4)), 128.2 (d, $^3J_{CF}$ 3.3, C(4)Ar*C*(1)), 129.0 (d, $^4J_{CF}$ 2.7, C(4)Ar*C*(2,6)H), 132.0 (C(4)Ar*C*(3,5)H), 136.8 (CH=*C*HCH$_3$), 166.0 (*C*(2)); *Minor diastereoisomer (selected signals)* δ_C: 18.3 (*C*H$_3$), 67.9 (*C*(3)), 124.5 (C(4)Ar*C*(4)), 127.4 (C(4)Ar*C*(2,6)H), 132.2 (C(4)Ar*C*(3,5)H), 137.0 (CH=*C*HCH$_3$), 165.9 (*C*(2)); **^{19}F{^{1}H} NMR** (471 MHz, $CDCl_3$) δ_F: −124.9 (d, *J* 282.0, C*F*$_2$), −121.9 (d, *J* 282.0, C*F*$_2$), −79.0 (C*F*$_3$); *Minor diastereoisomer* δ_F: −122.2 (d, *J* 284.0, C*F*$_2$), −119.7 (d, *J* 284.0, C*F*$_2$), −79.3 (C*F*$_3$); **IR** (neat) ν_{max} cm^{-1}: 1848 (C=O), 1344, 1090, 993, 812; **HRMS** (APCI$^+$) $C_{14}H_{11}O_2F_5Br^+$ [M+H]$^+$ found 384.9863, requires 384.9857 (+1.6 ppm).

(3*S*,4*S*)-4-(4-Bromophenyl)-3-(4-dimethylaminophenyl)-4-(trifluoromethyl)oxetan-2-one 53

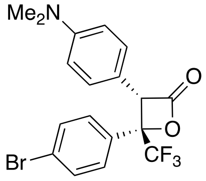

Following General Procedure D, 1-(4-bromophenyl)-2,2,2-trifluoroethan-1-one (64 mg, 0.25 mmol), anhydride **S1** (213 mg, 0.63 mmol), HyperBTM (4 mg, 0.0125 mmol) and *i*-Pr_2NEt (54 µL, 0.31 mmol) in CH_2Cl_2 gave, after Biotage column chromatography (0→5% Et_2O in hexane), (3*S*,4*S*)-4-(4-bromophenyl)-3-(4-dimethylaminophenyl)-4-(trifluoromethyl)oxetan-2-one **53** as a yellow oil (78 mg, 73%, 93:7 dr).

$[\alpha]_D^{20}$ = +161.7 (*c* 1.0, $CHCl_3$); **Chiral HPLC analysis**, Chiralcel OD-H (97:3 Hexane:IPA, flow rate 1.5 mLmin^{-1}, 211 nm, 40 °C) t_R(3*R*,4*R*): 4.7 min, t_R(3*S*,4*S*): 6.2 min, 95:5 er; **^{1}H NMR** (500 MHz, $CDCl_3$) δ_H: 2.90 (6H, s, N(C*H*$_3$)$_2$), 5.33 (1H, s, C(3)*H*), 6.46–6.52 (2H, m, C(3)ArC(3,5)*H*), 6.67–6.72 (2H, m, C(3)ArC(2,6)*H*), 6.96–7.02 (2H, m, C(4)ArC(2,6)*H*), 7.36–7.41 (2H, m, C(4-ArC(3,5)*H*); *Minor diastereoisomer* δ_H: 2.99 (6H, s, NC*H*$_3$), 5.19 (1H, s, C(3)*H*), 6.72–6.75 (2H, m, C(3)ArC(3,5)*H*), 7.25–7.29 (2H, m, C(3)ArC(2,6)*H*), 7.47–7.51 (2H, m, C(4)ArC(2,6)*H*), 7.63–7.68 (2H, m, C(4)ArC(3,5)*H*); **^{13}C{^{1}H} NMR** (126 MHz, $CDCl_3$) δ_C: 40.2 (N(*C*H$_3$)$_2$), 63.2 (*C*(3)H), 80.5 (q, $^2J_{CF}$ 32.4, *C*(4)), 112.2 (C(3)Ar*C*(3,5)H), 114.6 (C(3)Ar*C*(1)), 123.5 (q, $^1J_{CF}$ 282.2, *C*F$_3$), 124.1 (C(4)Ar*C*(1)), 128.2 (C(4)Ar*C*(4)), 129.3 (C(4)Ar*C*(2,6)H), 130.5 (C(3)Ar*C*(3,5)H), 131.5 (C(4)Ar*C*(3,5)H), 150.7 (C(3)Ar*C*(4)), 167.1 (*C*(2)); *Minor diastereoisomer (selected signals)* δ_C: 40.3 (N(*C*H$_3$)$_2$), 66.4 (*C*(3)), 112.3 (C(3)Ar*C*(3,5)H), 114.2 (C(3)Ar*C*(1)), 132.2 (C(4)Ar*C*(3,5)H), 166.4 (*C*(2)); **^{19}F{^{1}H} NMR** (471 MHz, $CDCl_3$) δ_F: −78.5 (C*F*$_3$); *Minor diastereoisomer* δ_F: −73.3 (C*F*$_3$); **IR** (neat) ν_{max} cm^{-1}: 2895, 1850 (C=O), 1526, 1132, 935, 816; **HRMS** (APCI$^+$) $C_{18}H_{16}NO_2F_3Br^+$ $[M+H]^+$ found 414.0316 requires 414.0311 (+1.2 ppm).

(3*S*,4*S*)-4-(4-Bromophenyl)-3-(4-methoxyphenyl)-4-(trifluoromethyl)oxetan-2-one 54

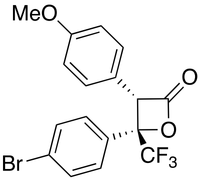

Following General Procedure D, 1-(4-bromophenyl)-2,2,2-trifluoroethan-1-one (64 mg, 0.25 mmol), anhydride **S2** (197 mg, 0.63 mmol), HyperBTM (4 mg, 0.0125 mmol) and *i*-Pr_2NEt (54 µL, 0.31 mmol) in CH_2Cl_2 gave, after Biotage chromatography (0→5% Et_2O in hexane), (3*S*,4*S*)-4-(4-bromophenyl)-3-(4-methoxyphenyl)-4-(trifluoromethyl)oxetan-2-one **54** as a colourless oil (81 mg, 81%, 90:19 dr).

$[\alpha]_D^{20}$ = +109.5 (*c* 1.0, $CHCl_3$); **Chiral HPLC analysis**, Chiralcel OD-H (97:3 Hexane:IPA, flow rate 1.5 mLmin^{-1}, 211 nm, 40 °C) t_R(3*R*,4*R*): 4.1 min, t_R(3*S*,4*S*): 4.5 min, 92:8 er; **^{1}H NMR** (500 MHz, $CDCl_3$) δ_H:

3.74 (3H, s, OCH_3), 5.37 (1H, s, C(3)*H*), 6.71–6.76 (2H, m, C(3)ArC(3,5)*H*), 6.78–6.83 (2H, m, C(3)ArC(2,6)*H*), 6.94–7.00 (2H, m, C(4)ArC(2,6)*H*), 7.36–7.40 (2H, m, C(4)ArC(3,5)H); *Minor diastereoisomer (selected signals)* δ_H: 3.84 (3H, s, OCH_3), 5.37 (1H, s, C(3)*H*), 7.47–7.51 (2H, m, C(4)ArC(2,6)*H*), 7.64–7.68 (2H, m, C(4)ArC(3,5)H); **^{13}C{^{1}H} NMR** (126 MHz, $CDCl_3$) δ_C: 55.4 (O*C*H_3), 63.0 (*C*(3)H), 80.3 (q, $^2J_{CF}$ 32.6, *C*(4)), 114.5 (C(3)Ar*C*(2,6)H), 119.9 (C(3)Ar*C*(1)), 123.4 (q, $^1J_{CF}$ 282.2, *C*F_3), 124.3 (C(1)Ar*C*(4)), 127.8 (C(4)Ar*C*(4)), 129.1 (C(4)Ar*C*(2,6)H), 130.9 (C(3)Ar*C*(3,5)H), 131.6 (C(4)Ar*C*(3,5)H), 160.3 (C(3)Ar*C*(4)), 166.4 (*C*(2)); *Minor diastereoisomer (selected signals)* δ_C: 55.5 (O*C*H_3), 65.9 (*C*(3)), 114.6 (C(3)Ar*C*(2,6)H), 128.0 (Ar*C*), 130.2 (C(3)Ar*C*(3,5)H), 132.3 (C(4)Ar*C*(3,5)H), 160.3 (C(3)Ar*C*(4)), 165.7 (*C*(2)); **^{19}F{^{1}H} NMR** (471 MHz, $CDCl_3$) δ_F: −78.5 (C*F*$_3$); *Minor diastereoisomer* δ_F: −73.3 (C*F*$_3$); **IR** (neat) ν_{max} cm^{-1}: 2961, 1852 (CO), 1516, 1254, 937, 818; **HRMS** (APCI$^+$) $C_{17}H_{13}O_3F_3Br^+$ $[M+H]^+$ found 401.0002, requires 400.9995 (+1.7 ppm).

(3*S*,4*S*)-4-(4-Bromophenyl)-3-(4-fluorophenyl)-4-(trifluoromethyl)oxetan-2-one 55

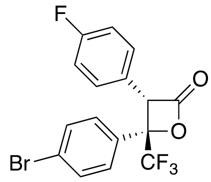

Following General Procedure D, 1-(4-bromophenyl)-2,2,2-trifluoroethan-1-one (64 mg, 0.25 mmol), anhydride **S3** (182 mg, 0.625 mmol), HyperBTM (4 mg, 0.0125 mmol) and *i*-Pr_2NEt (54 µL, 0.31 mmol) in CH_2Cl_2 gave, after Biotage chromatography (0→5% Et_2O in hexane), (3*S*,4*S*)-4-(4-bromophenyl)-3-(4-fluorophenyl)-4-(trifluoromethyl)oxetan-2-one **55** as a colourless oil (91 mg, 93%) as an inseparable mixture of diastereoisomers (90:10 dr).

$[\alpha]_D^{20} = +140.7$ (*c* 1.0, $CHCl_3$); **Chiral HPLC analysis**, Chiralcel OD-H (97:3 Hexane:IPA, flow rate 1.5 mLmin^{-1}, 211 nm, 40 °C) t_R(3*R*,4*R*): 3.3 min, t_R(3*S*,4*S*): 3.9 min, 96:4 er; **^{1}H NMR** (500 MHz, $CDCl_3$) δ_H: 5.40 (1H, s, C(3)*H*), 6.85–6.94 (4H, m, C(3)ArC(2,3,5,6)*H*), 6.94–6.99 (2H, m, C(4)ArC(2,6)*H*), 7.36–7.42 (2H, m, C(4)ArC(3,5)*H*); *Minor diastereoisomer* δ_H: 5.25 (1H, s, C(3)*H*), 7.16 (2H, app t, *J* 8.6, C(3)Ar*H*), 7.43–7.47 (2H, m, C(3)Ar*H*), 7.47–7.52 (2H, m, C(4)ArC(2,6)*H*), 7.65–7.71 (2H, m, C(4)ArC(3,5)*H*); **^{13}C{^{1}H} NMR** (126 MHz, $CDCl_3$) δ_C: 62.7 (*C*(3)H), 80.2 (q, *J* 32.9, *C*(4)), 116.4 (d, *J* 22.0, C(3)Ar*C*(3,5)H), 124.1 (d, *J* 3.6, C(3)Ar*C*(1)), 124.6 (C(4)Ar*C*(4)), 125.8 (q, *J* 230.7, *C*F_3), 127.5 (C(4)Ar*C*(1)), 129.0 (C(4)Ar*C*(2,6)H), 131.4 (d, *J* 8.5, C(3)Ar*C*(2,6)H), 131.8 (C(4)Ar*C*(3,5)H), 163.1 (d, *J* 250.3, C(3)Ar*C*(4)), 165.6 (*C*(2)); *Minor diastereoisomer (selected signals)* δ_C: 65.5 (*C*(3)H), 165.1 (*C*(2)); **^{19}F NMR** (471 MHz, $CDCl_3$) δ_F: −110.7 (Ar*F*), −78.4 (C*F*$_3$); *Minor diastereoisomer* δ_F: −111.2 (Ar*F*), −73.3 (C*F*$_3$); **IR** (neat) ν_{max} cm^{-1}: 1857 (CO); **HRMS** (APCI$^+$) $C_{16}H_9BrF_4O_2^+$ $[M]^+$ found 387.9724, requires 387.9717 (+1.8 ppm).

(3*S*,4*S*)-3,4-Bis(4-bromophenyl)-4-(trifluoromethyl)oxetan-2-one 56

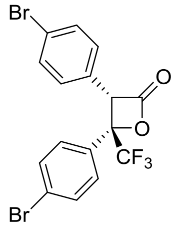

Following General Procedure D, 1-(4-bromophenyl)-2,2,2-trifluoroethan-1-one (64 mg, 0.25 mmol), anhydride **S4** (258 mg, 0.63 mmol), HyperBTM (4 mg, 0.0125 mmol) and *i*-Pr_2NEt (54 µL, 0.31 mmol) in CH_2Cl_2 (crude dr 86:14) gave, after Biotage chromatography (0→3% Et_2O in hexane), (3*S*,4*S*)-3,4-bis(4-bromophenyl)-4-(trifluoromethyl)oxetan-2-one **56** as a colourless oil (97 mg, 86%, 85:15 dr).

$[\alpha]_D^{20}$ = +97.0 (*c* 1.0, $CHCl_3$); **Chiral HPLC analysis**, Chiralcel OD-H (97:3 Hexane:IPA, flow rate 1.5 mLmin^{-1}, 211 nm, 40 °C) t_R(3*R*,4*R*): 4.1 min, t_R(3*S*,4*S*): 4.5 min, 91:9 er; **^{1}H NMR** (500 MHz, $CDCl_3$) δ_H: 5.38 (1H, s, C(3)*H*), 6.75–6.80 (2H, m, C(3)ArC(2,6)*H*), 6.94–7.01 (2H, m, C(4)ArC(2,6)*H*), 7.34–7.38 (2H, m, C(3)ArC(3,5)*H*), 7.38–7.43 (2H, m, C(4)ArC(3,5)*H*); *Minor diastereoisomer* δ_H: 5.23 (1H, s, C(3)*H*), 7.31–7.35 (2H, m, C(3)ArC(2,6)*H*), 7.46–7.51 (2H, m, C(4)ArC(2,6)*H*), 7.57–7.62 (2H, m, C(3)ArC(3,5)*H*), 7.65–7.69 (2H, m, C(4)ArC(3,5)*H*); **^{13}C{^{1}H} NMR** (126 MHz, $CDCl_3$) δ_C: 62.8 (*C*(3)H), 80.0 (q, $^2J_{CF}$ 33.0, *C*(4)), 123.3 (q, $^1J_{CF}$ 282.3, *C*F_3), 124.0 (C(3)Ar*C*(1)), 124.7 (C(4)Ar*C*(1)), 127.1 (C(3)Ar*C*(4)), 127.6 (C(4)Ar*C*(4)), 128.9 (C(4)Ar*C*(2,6)H), 131.1 (C(3)Ar*C*(2,6)H), 131.9 (C(3)Ar*C*(3,5)H), 132.4 (C(4)Ar*C*(3,5)H), 165.2 (*C*(2)). *Minor diastereoisomer (selected signals)* δ_C: 65.4 (*C*(3)H), 128.0 (Ar*C*H), 130.4 (Ar*C*H), 132.5 (Ar*C*H), 164.7 (*C*(2)); **^{19}F{^{1}H} NMR** (471 MHz, $CDCl_3$) δ_F: −78.4 (C*F*$_3$); *Minor diastereoisomer* δ_F: −73.3 (C*F*$_3$); **IR** (neat) ν_{max} cm^{-1}: 1852 (CO), 1491, 1074, 937, 818; **HRMS** (APCI$^+$) $C_{16}H_{10}Br_2F_3O_2^+$ $[M+H]^+$ found 448.9002, requires 448.8994 (+1.8 ppm).

(3*S*,4*S*)-4-(4-Bromophenyl)-3-(thiophen-3-yl)-4-(trifluoromethyl)oxetan-2-one 57

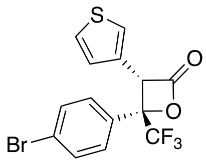

Following General Procedure D, 1-(4-bromophenyl)-2,2,2-trifluoroethan-1-one (64 mg, 0.25 mmol), anhydride **S8** (167 mg, 0.63 mmol), HyperBTM (4 mg, 0.0125 mmol) and *i*-Pr_2NEt (54 µL, 0.31 mmol) in CH_2Cl_2 (crude dr 78:22) gave, after Biotage chromatography (0→3% Et_2O in hexane), (3*S*,4*S*)-4-(4-bromophenyl)-3-(thiophen-3-yl)-4-(trifluoromethyl)oxetan-2-one **57** as a colourless oil (88 mg, 94%, 89:11 dr).

$[\alpha]_D^{20}$ = +76.3 (*c* 1.0, $CHCl_3$); **Chiral HPLC analysis**, Chiralcel OD-H (97:3 Hexane:IPA, flow rate 1.5 mLmin^{-1}, 211 nm, 40 °C) t_R(3*R*,4*R*): 3.9 min, t_R(3*S*,4*S*): 4.7 min, 95:5 er; **^{1}H NMR** (500 MHz, $CDCl_3$) δ_H:

5.51 (1H, s, C(3)*H*), 6.51 (1H, dd, *J* 5.0, 1.4, C(3)ArC(5)*H*), 7.00–7.05 (2H, m, C(4)ArC(2,6)*H*), 7.05 (1H, dd, *J* 3.0, 1.3, C(3)ArC(2)*H*), 7.19 (1H, dd, *J* 5.0, 2.9, C(3)ArC(4)*H*), 7.39–7.43 (2H, m, C(4)ArC(3,5)*H*). *Minor diastereoisomer* δ_H: 5.27 (1H, s, C(3)*H*), 7.14–7.17 (1H, m, C(3)ArC(5)*H*), 7.43–7.51 (4H, m, C(3)ArC(2,4)*H* + C(4)ArC(2,6)*H*), 7.64–7.69 (2H, m, C(4)ArC(3,5)*H*); **^{13}C{^{1}H} NMR** (126 MHz, $CDCl_3$) δ_C: 58.4 (*C*(3)), 80.0 (q, $^2J_{CF}$ 32.4, *C*(4)), 123.3 (q, $^1J_{CF}$ 283.0, *C*F_3), 124.5 (C(4)Ar*C*(4)), 126.7 (C(3)Ar*C*(2)H), 127.2 (C(3)Ar*C*(4)H), 127.4 (C(3)Ar*C*(5)H), 127.8 (C(4)Ar*C*(1)), 127.8 (C(3)Ar*C*(3)), 128.8 (C(4)Ar*C*(2,6)H), 131.7 (C(4)Ar*C*(3,5)H), 165.6 (*C*(2)); *Minor diastereoisomer (selected signals)* δ_C: 61.7 (*C*(3)), 165.3 (*C*(2)); **^{19}F{^{1}H} NMR** (471 MHz, $CDCl_3$) δ_F: −78.5 (C*F*$_3$); *Minor diastereoisomer* δ_F: −74.0 (C*F*$_3$); **IR** (neat) ν_{max} cm^{-1}: 1848 (CO), 1267, 1134, 937, 818; **HRMS** (APCI$^+$) $C_{14}H_9O_2F_3BrS^+$ $[M+H]^+$ found 376.9459 requires 376.9453 (+1.6 ppm).

(3*S*,4*S*)-4-(4-Bromophenyl)-3-((*E*)-prop-1-en-1-yl)-4-(trifluoromethyl)oxetan-2-one 58

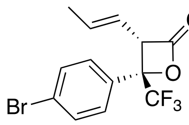

Following General Procedure D, 1-(4-bromophenyl)-2,2,2-trifluoroethan-1-one (64 mg, 0.25 mmol), anhydride **S10** (114 mg, 0.63 mmol), HyperBTM (4 mg, 0.0125 mmol) and *i*-Pr_2NEt (54 µL, 0.31 mmol) in CH_2Cl_2 (crude 69:31 dr) gave, after Biotage chromatography (0→1% Et_2O in hexane), (3*S*,4*S*)-4-(4-bromophenyl)-3-((*E*)-prop-1-en-1-yl)-4-(trifluoromethyl)oxetan-2-one **58** as a colourless oil (70 mg, 84%, > 95:5 dr).

$[\boldsymbol{\alpha}]_{\boldsymbol{D}}^{\mathbf{20}} =$ +12.1 (*c* 1.0, $CHCl_3$); **Chiral HPLC analysis**, Chiralpak AD-H (99:1 Hexane:IPA, flow rate 1.5 mLmin^{-1}, 211 nm, 40 °C) t_R(3*S*,4*S*): 3.2 min, t_R(3*R*,4*R*): 3.5 min, 98:2 er; **^{1}H NMR** (500 MHz, $CDCl_3$) δ_H: 1.67 (3H, dd, *J* 6.6, 1.7, C*H*$_3$), 4.72 (1H, d, *J* 9.3, C(3)*H*), 4.91–4.98 (1H, m, C*H*=CHCH$_3$), 5.81–5.90 (1H, m, CH=C*H*CH$_3$), 7.24–7.29 (2H, m, C(4)ArC(2,6)*H*), 7.57–7.63 (2H, m, C(4)ArC(3,5)*H*); *Minor diastereoisomer (selected signals)* δ_H: 1.77 (3H, dd, *J* 7.0, 1.9, C*H*$_3$); **^{13}C{^{1}H} NMR** (126 MHz, $CDCl_3$) δ_C: 18.2 (*C*H$_3$), 60.8 (*C*(3)H), 79.0 (q, $^2J_{CF}$ 32.7, *C*(4)), 118.0 (*C*H=CHCH$_3$), 123.4 (q, $^1J_{CF}$ 282.2, *C*F$_3$), 124.6 (C(4)Ar*C*(4)), 128.1 (C(4)Ar*C*(1)), 129.0 (C(4)Ar*C*(2,6)H), 132.0 (C(4)Ar*C*(3,5)H), 136.6 (*CH*=*CH*CH$_3$), 166.0 (*C*(2)); **^{19}F{^{1}H} NMR** (471 MHz, $CDCl_3$) δ_F: −78.8 (C*F*$_3$); **IR** (neat) ν_{max} cm^{-1}: 2922, 1850 (CO), 1267, 939, 816; **HRMS** (APCI$^+$) $C_{13}H_{11}F_3O_2Br^+$ $[M+H]^+$ found 334.9895 requires 334.9889 (+1.8 ppm).

Product Derivatisations

(2*S*,3*S*)-3-(4-Bromophenyl)-4,4,5,5,6,6,7,7,7-nonafluoro-3-hydroxy-2-phenylheptanamide 59

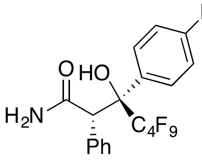

A solution of (3*S*,4*S*)-4-(4-bromophenyl)-4-(perfluorobutyl)-3-phenyloxetan-2-one **21** (104 mg, 0.2 mmol) in ammonia in dioxane (0.5 M, 2 mL, 1 mmol) was allowed to stir at r.t. for 16 h. The mixture was concentrated *in vacuo* to give a residue which was purified *via* Biotage chromatography (0→40% Et_2O in hexane) to give (2*S*,3*S*)-3-(4-bromophenyl)-4,4,5,5,6,6,7,7,7-nonafluoro-3-hydroxy-2-phenylheptanamide **59** as a colourless solid (86 mg, 80%, > 95:5 dr).

m.p. 155–157 °C; $[\alpha]_D^{20} = -26.0$ (*c* 0.5, $CHCl_3$); **Chiral HPLC analysis**, Chiralcel OD-H (95:5 hexane:IPA, flow rate 1.5 mLmin^{-1}, 211 nm, 40 °C) t_R(2*R*,3*R*): 11.3 min, t_R(2*S*,3*S*): 22.2 min, 97:3 er; **^{1}H NMR** (500 MHz, $CDCl_3$) δ_H: 4.07 (1H, d, *J* 1.8, C(2)*H*), 5.88 (2H, d, *J* 11.3, NH_2), 7.08–7.15 (3H, m, PhC(3,4,5)*H*), 7.21–7.25 (2H, m, PhC(2,6)*H*), 7.25–7.32 (4H, m, C(3)ArC(2,3,5,6)*H*), 8.01 (1H, s, O*H*); **^{13}C{^{1}H} NMR** (126 MHz, $CDCl_3$) δ_C: 51.2 (d, *J* 2.9, *C*(2)H), 80.6 (t, *J* 21.9, *C*(3)), 108.0–120.1 ($CF_2CF_2CF_2CF_3$), 122.5 (C(3)Ar*C*(4)), 128.2 (C(3)Ar*C*(2,6)H), 128.5 (Ph*C*(3,5)H), 128.5 (Ph*C*(4)H), 129.7 (Ph*C*(2,6)H), 130.9 (C(3)Ar*C*(3,5)H), 133.0 (Ph*C*(1)), 134.5 (d, *J* 4.9, C(3)Ar*C*(1)), 176.0 (*C*(1)); **^{19}F NMR** (471 MHz, $CDCl_3$) δ_F : −127.1 – −124.9 (m, CF_2), −119.8 – −117.7 (m, CF_2), −115.1 – −112.5 (m, CF_2), −80.9 – −80.7 (m, CF_3); **IR** (neat) ν_{max} cm^{-1}: 3298 (br, OH), 3192 (NH), 1665 (CO); **HRMS** (ESI$^+$) $C_{19}H_{13}O_2NBrF_9Na^+$ $[M+Na]^+$, found 559.9873, requires 559.9878 (−0.9 ppm).

(2*S*,3*S*)-*N*-Benzyl-3-(4-bromophenyl)-4,4,5,5,6,6,7,7,7-nonafluoro-3-hydroxy-2-phenylheptanamide 60

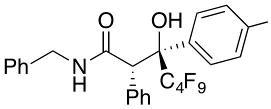

Benzylamine (109 µL, 1 mmol) was added to a solution of (3*S*,4*S*)-4-(4-bromophenyl)-4-(perfluorobutyl)-3-phenyloxetan-2-one **21** (104 mg, 0.2 mmol) in CH_2Cl_2 (0.1 M) and allowed to stir at r.t. for 16 h. The mixture was diluted with CH_2Cl_2 and washed with 1 M HCl (3 × 10 mL). The organic layer was dried ($MgSO_4$), filtered and concentrated *in vacuo* to give **(**2*S*,3*S*)-*N*-benzyl-3-(4-bromophenyl)-4,4,5,5,6,6,7,7,7-nonafluoro-3-hydroxy-2-phenylheptanamide **60** as a colourless solid (122 mg, 97%, > 95:5 dr).

m.p. 159–161 °C; $[\alpha]_D^{20} = -6.0$ (*c* 0.5, $CHCl_3$); **Chiral HPLC analysis**, Chiralpak AD-H (97:3 hexane:IPA, flow rate 1.5 mLmin^{-1}, 211 nm, 40 °C) t_R(2*R*,3*R*): 7.3 min, t_R(2*S*,3*S*): 19.4 min, 94:6 er; **^{1}H NMR** (500 MHz, $CDCl_3$) δ_H: 3.96 (1H, d, *J* 1.8, C(2)*H*), 4.36 (1H, dd, *J* 14.8, 5.3, CH^AH^B), 4.58 (1H, dd, *J* 14.8, 6.0,

CH^AH^B), 6.06 (1H, app t, *J* 5.6, N*H*), 6.98–7.20 (5H, m, NHCH$_2$Ar*H*), 7.15–7.51 (9H, m, Ar*H*), 8.19 (1H, s, O*H*); **^{13}C{^{1}H} NMR** (126 MHz, CDCl$_3$) δ_C: 43.9 (*C*H$_2$), 52.3 (*C*(2)H), 80.5 (t, *J* 21.9, *C*(3)), 107.9–120.2 (m, *C*F$_2$*C*F$_2$*C*F$_2$*C*F$_3$), 122.4 (C(3)Ar*C*(4)), 127.8 (NCH$_2$Ar*C*(2,6)H), 128.0 (Ar*C*H), 128.1 (Ar*C*H), 128.5 (Ar*C*H), 129.0 (Ar*C*H), 129.7 (C(3)Ar*C*(2,6)H), 130.9 (NCH$_2$Ar*C*(3,5)H), 133.3 (Ph*C*(1)), 134.6 (d, *J* 4.5, C(3)Ar*C*(1)), 136.7 (NCH$_2$Ar*C*(1)), 173.4 (*C*(1)); **^{19}F NMR** (471 MHz, CDCl$_3$) δ_F : −127.2 – −124.8 (m, C*F*$_2$), −119.9 – −117.7 (m, C*F*$_2$), −115.2 – −112.5 (m, C*F*$_2$), −80.9 – −80.7 (m, C*F*$_3$); **IR** (neat) ν_{max} cm^{-1}: 3242 (br, OH), 3092 (NH), 1628 (CO); **HRMS** (ESI$^+$) $C_{26}H_{20}BrF_9NO_2$ [M+H]$^+$ found 628.0523, requires 628.0528 (−0.8 ppm).

(2*S*,3*S*)-3-(4-Bromophenyl)-4,4,5,5,6,6,7,7,7-nonafluoro-3-hydroxy-1-morpholino-2-phenylheptan-1-one 61

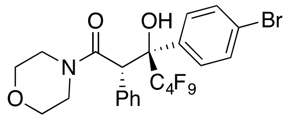

Morpholine (88 µL, 1 mmol) was added to a solution of (3*S*,4*S*)-4-(4-bromophenyl)-4-(perfluorobutyl)-3-phenyloxetan-2-one **21** (104 mg, 0.2 mmol) in CH$_2$Cl$_2$ (0.1 M) and allowed to stir at r.t. for 16 h. The mixture was diluted with CH$_2$Cl$_2$ and washed with 0.1 M HCl (3 × 10 mL). The organic layer was dried (MgSO$_4$), filtered and concentrated *in vacuo* to give a residue, which was purified *via* Biotage chromatography (0→10% EtOAc in hexane) to give (2*S*,3*S*)-3-(4-bromophenyl)-4,4,5,5,6,6,7,7,7-nonafluoro-3-hydroxy-1-morpholino-2-phenylheptan-1-one **61** as a colourless solid (110 mg, 90%, > 95:5 dr).

m.p. 175-177 °C; $[\alpha]_D^{20} = -35.0$ (*c* 1.0, CHCl$_3$); **Chiral HPLC analysis**, Chiralpak AD-H (97:3 hexane:IPA, flow rate 1.5 mLmin^{-1}, 211 nm, 40 °C) t_R(2*R*,3*R*): 4.6 min, t_R(2*S*,3*S*): 5.2 min, 98:2 er; **^{1}H NMR** (500 MHz, CDCl$_3$) δ_H: 3.36–3.81 (8H, m, C*H*$_2$), 4.48 (1H, d, *J* 1.9, C(2)*H*), 6.91-7.75 (9H, m, Ar*H*), 8.76 (1H, s, O*H*); **^{13}C{^{1}H} NMR** (126 MHz, CDCl$_3$) δ_C: 42.6 (N*C*H$_2$), 46.0 (*C*(2)H), 46.6 (N*C*H$_2$), 66.2 (O*C*H$_2$), 66.5 (O*C*H$_2$), 80.7 (t, *J* 21.4, *C*(3)), 106.6–120.1 (m, *C*F$_2$*C*F$_2$*C*F$_2$*C*F$_3$), 122.3 (C(3)Ar*C*(4)), 127.3 (C(2)Ar*C*(1)), 127.9 (Ar*C*H), 128.4 (Ar*C*H), 129.3 (Ar*C*H), 129.9 (Ar*C*H), 130.1 (C(3)Ar*C*(1)), 132.2 (C(2)Ar*C*(1)), 134.8 (C(3)Ar*C*(1)), 171.9 (*C*(1)); **^{19}F NMR** (471 MHz, CDCl$_3$) δ_F: −127.1 – −124.8 (m, C*F*$_2$), −119.6 – −117.7 (m, C*F*$_2$), −114.7 – −112.1 (m, C*F*$_2$), −81.9 – −80.6 (m, C*F*$_3$); **IR** (neat) ν_{max} cm^{-1}: 2970 (OH), 1717 (CO); **HRMS** (NSI$^+$) $C_{23}H_{20}BrF_9NO_3^+$ [M+H]$^+$ found 608.0474, requires 608.0477 (−0.6 ppm).

Methyl (2*S*,3*S*)-3-(4-bromophenyl)-4,4,5,5,6,6,7,7,7-nonafluoro-3-hydroxy-2-phenylheptanoate 62

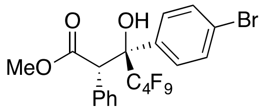

A freshly prepared solution of 1 M NaOMe in MeOH (1 mL, 1 mmol) was added to a solution of (3*S*,4*S*)-4-(4-bromophenyl)-4-(perfluorobutyl)-3-phenyloxetan-2-one **21** (104 mg, 0.2 mmol) in CH_2Cl_2 (0.1 M) at −78 °C under an inert atmosphere and allowed to stir for 2 h. Saturated NH_4Cl was added; the mixture was allowed to warm to r.t., and extracted with CH_2Cl_2 (3 × 10 mL). The combined organic layers were dried ($MgSO_4$), filtered and concentrated *in vacuo* to give a residue, which was purified *via* Biotage chromatography (0→2% Et_2O in hexane) to give methyl (2*S*,3*S*)-3-(4-bromophenyl)-4,4,5,5,6,6,7,7,7-nonafluoro-3-hydroxy-2-phenylheptanoate **62** as a colourless solid (110 mg, 90%, > 95:5 dr).

m.p. 106–108 °C; $[\alpha]_D^{20} = -67.2$ (*c* 1.0, $CHCl_3$); **Chiral HPLC analysis**, Chiralpak AD-H (99:1 hexane:IPA, flow rate 1.5 mLmin^{-1}, 211 nm, 40 °C) t_R(2*S*,3*S*): 3.2 min, t_R(2*R*,3*R*): 3.8 min, 98:2 er; **^{1}H NMR** (500 MHz, $CDCl_3$) δ_H: 3.80 (3H, s, C*H*$_3$), 4.37 (1H, d, *J* 1.8, C(2)*H*), 6.71 (1H, s, O*H*), 7.03–7.18 (3H, m, Ph(2,4,6)*H*), 7.18–7.26 (2H, m, Ph(3,5)*H*), 7.25–7.33 (2H, m, C(3)Ar(2,6)*H*), 7.43 (2H, br, C(3)Ar(3,5)*H*); **^{13}C{^{1}H} NMR** (126 MHz, $CDCl_3$) δ_C: 51.5 (d, $^3J_{CF}$ 2.9, *C*(2)H), 53.4 (*C*H$_3$), 80.0 (t, *J* 22.0, *C*(3)), 122.6 (C(3)Ar(4)), 128.3 (Ph*C*(4)H), 128.5 (Ph*C*(2,6)H), 128.6 (br, C(3)Ar*C*(2,6)H), 130.0 (C(3)Ar*C*(3,5)H), 131.0 (br, C(3)Ar*C*(3,5)H), 131.4 (C(3)Ar(4)*C*), 132.0 (Ph*C*(1)), 133.8 (d, $^3J_{CF}$ 4.9, C(3)Ar*C*(1)), 174.8 (*C*(1)); **^{19}F NMR** (471 MHz, $CDCl_3$) δ_F: −127.0 – −125.0 (m, C*F*$_2$), −119.8 – −117.9 (m, C*F*$_2$), −116.0 – −112.8 (m, C*F*$_2$), −80.9 – −80.8 (m, C*F*$_3$); **IR** (neat) ν_{max} cm^{-1}: 3368 (br, OH), 1712 (CO); **HRMS** (APCI$^+$) $C_{20}H_{15}B_rF_9O_3^+$ [M+H]$^+$ found 553.0065, requires 553.0055 (+1.8 ppm).

***tert*-Butyl 5-methoxy-2-(4-((2*S*,3*S*)-4-oxo-2-(perfluorobutyl)-3-phenyloxetan-2-yl)phenyl)-1*H*-indole-1-carboxylate 64**

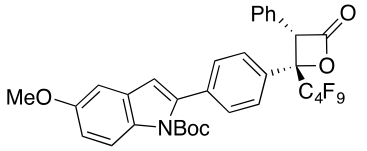

N-Boc-5-methoxyindole-2-boronic acid (88 mg, 0.3 mmol), $Pd(PPh_3)_4$ (29 mg, 0.025 mmol) and 2 M aqueous Na_2CO_3 (0.38 mL, 0.76 mmol) were added to a solution of (3*S*,4*S*)-4-(4-bromophenyl)-4-(perfluorobutyl)-3-phenyloxetan-2-one **21** (130 mg, 0.25 mmol) in degassed DMF (4 mL) under an inert atmosphere, and the mixture was heated at 85 °C for 4 h. After cooling to r.t. and water and CH_2Cl_2 were added, and the mixture was extracted with CH_2Cl_2 (3 × 10 mL). The combined organic layers were dried ($MgSO_4$), filtered and concentrated *in vacuo* to give a residue, which was purified *via* Biotage column chromatography (0→50% CH_2Cl_2 in hexane) to give *tert*-butyl 5-methoxy-2-(4-

((2*S*,3*S*)-4-oxo-2-(perfluorobutyl)-3-phenyloxetan-2-yl)phenyl)-1*H*-indole-1-carboxylate **64** as a colourless oil (60 mg, 35%, > 95:5 dr).

$[\alpha]_D^{20}$ = 120.4 (*c* 1.0, $CHCl_3$); **Chiral HPLC analysis**, Chiralcel OD-H (97:3 Hexane:IPA, flow rate 1.5 mLmin^{-1}, 211 nm, 40 °C) t_R(2*R*,3*R*): 3.7 min, t_R(2*S*,3*S*): 4.8 min, 97:3 er; **^{1}H NMR** (500 MHz, $CDCl_3$) δ_H: 1.28 (9H, s, C(CH_3)$_3$), 3.85 (3H, s, OCH_3), 5.64 (1H, s, C(3)*H*), 6.39 (1H, s, IndC(3)*H*), 6.89–6.92 (2H, m, PhC(2,6)*H*), 6.93 (1H, dd, *J* 9.0, 2.6, IndC(6)*H*), 6.98 (1H, d, *J* 2.6, IndC(4)*H*), 7.11 (2H, d, *J* 7.9, C(4)ArC(3,5)*H*), 7.17–7.22 (2H, m, PhC(3,5)*H*), 7.23–7.27 (3H, m, C(4)ArC(2,6)*H* + PhC(4)*H*), 8.07 (1H, d, *J* 9.0, Ind(7)*H*); **^{13}C{^{1}H} NMR** (126 MHz, $CDCl_3$) δ_C: 27.8 (C(CH_3)$_3$), 55.8 (OCH_3), 64.6 (d, $^3J_{CF}$ 4.3, *C*(3)H), 81.3 (dd, $^2J_{CF}$ 31.4, 23.0, *C*(4)), 83.6 (*C*(CH_3)$_3$), 103.1 (Ind*C*(4)H), 111.2 (Ind*C*(3)H), 113.6 (Ind*C*(6)H), 116.2 (Ind*C*(7)H), 127.1 (C(4)Ar*C*(3,5)H), 127.7 (C(4)Ar*C*(1)), 128.2 (C(4)Ar*C*(2,6)H), 128.2 (Ph*C*(1)), 129.0 (Ph*C*(3,5)H), 129.4 (Ph*C*(4)H), 129.8 (Ind*C*(3a)), 129.9 (Ph*C*(2,6)H), 132.7 (Ind*C*(7a)), 136.1 (Ind*C*(2)), 139.7 (C(4)Ar*C*(4)), 150.1 (N*C*OOC(CH_3)$_3$), 156.2 (Ind*C*(5)), 166.3 (*C*(2)), C_4F_9 not observed; **^{19}F NMR** (471 MHz, $CDCl_3$) δ_F: −128.0 – −124.6 (m, CF_2), −122.4 – −120.9 (m, CF_2), −118.0 – −116.0 (m, CF_2), −80.9 – −80.8 (m, CF_3); **IR** (neat) ν_{max} cm^{-1}: 1854 (CO), 1732 (CO); **HRMS** (NSI$^+$) $C_{33}H_{27}F_9NO_5^+$ [M+H]$^+$ found 688.1727 requires 688.1740 (−1.9 ppm).

(2*R*,3*S*)-3-(4-Bromophenyl)-4,4,5,5,6,6,7,7,7-nonafluoro-2-phenylheptane-1,3-diol 65

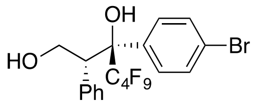

Following General Procedure E, lactone **21** (208 mg, 0.4 mmol) and DIBAL solution (0.8 mL, 0.8 mmol) in CH_2Cl_2 (4 mL) gave, after Biotage column chromatography (0→20% Et_2O in hexane), (2*R*,3*S*)-3-(4-bromophenyl)-4,4,5,5,6,6,7,7,7-nonafluoro-2-phenylheptane-1,3-diol **65** as a colourless solid (117 mg, 56%).

m.p. 121–123 °C; $[\alpha]_D^{20}$ = −36.9 (*c* 1.0, $CHCl_3$); **Chiral HPLC analysis**, Chiralcel OD-H (97:3 Hexane:IPA, flow rate 1.5 mLmin^{-1}, 211 nm, 40 °C) t_R(2*S*,3*R*): 16.7 min, t_R(2*R*,3*S*): 26.5 min, 97:3 er; **^{1}H NMR** (500 MHz, $CDCl_3$) δ_H: 2.16 (1H, s, O*H*), 3.61–3.66 (1H, m, C(2)*H*), 4.02–4.09 (1H, m, C(1)H^AH^B), 4.65 (1H, ddd, *J* 11.3, 3.5, 1.8, C(1)H^AH^B), 6.06 (1H, s, O*H*), 7.07–7.25 (6H, m , Ar*H*), 7.26–7.42 (3H, m, *Ar*H); **^{13}C{^{1}H} NMR** (126 MHz, $CDCl_3$) δ_C: 50.1 (*C*(2)H), 66.3 (d, $^4J_{CF}$ 6.6, *C*(1)H_2), 81.6 (t, $^2J_{CF}$ 22.4, *C*(3)), 115.1–121.7 (m, $CF_2CF_2CF_2CF_3$), 122.3 (C(3)Ar*C*(4)), 127.3 (Ph*C*(4)H), 128.3 (Ph*C*(3,5)H), 128.6 (C(3)Ar*C*(2,6)H), 130.0 (Ph*C*(2,6)H), 130.8 (C(3)Ar*C*(3,5)H), 135.7 (d, *J* 4.4, $^3J_{CF}$, C(3)Ar*C*(1)), 138.0 (Ph*C*(1)); **^{19}F NMR** (471 MHz, $CDCl_3$) δ_F: −127.9 – −124.0 (m, CF_2), −121.7 – −116.0 (m, CF_2), −114.9 – −113.1 (m, CF_2), −80.9 – −80.7 (m, CF_3); **IR** (neat) ν_{max} cm^{-1}: 3383 (br, OH); **HRMS** (NSI$^+$) $C_{19}H_{18}NBrF_9O_2^+$ [M+NH$_4$]$^+$ found 542.0358 requires 542.0372 (−2.6 ppm).

(2*R*,3*S*)-3-(4-Bromophenyl)-4,4,5,5,6,6,7,7,7-nonafluoro-2-(4-methoxyphenyl)heptane-1,3-diol 66

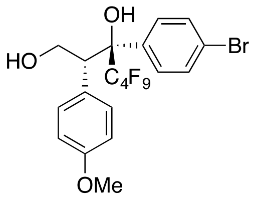

Following General Procedure E, lactone **37** (330 mg, 0.6 mmol) and DIBAL solution (1.2 mL, 1.2 mmol) in CH_2Cl_2 (6 mL) gave, after Biotage column chromatography (0→25% Et_2O in hexane), (2*R*,3*S*)-3-(4-bromophenyl)-4,4,5,5,6,6,7,7,7-nonafluoro-2-(4-methoxyphenyl)heptane-1,3-diol **66** as a colourless oil (210 mg, 63%).

$[\alpha]_D^{20} = -27.3$ (*c* 1.0, $CHCl_3$); **Chiral HPLC analysis**, Chiralcel OD-H (97:3 Hexane:IPA, flow rate 1.5 $mLmin^{-1}$, 211 nm, 40 °C) t_R(2*R*,3*S*): 8.3 min, t_R(2*S*,3*R*): 10.8 min, 97:3 er; **^{1}H NMR** (500 MHz, $CDCl_3$) δ_H: 2.09 (1H, dd, *J* 5.7, 4.3, C(1)O*H*), 3.57-3.62 (1H, m, C(2)*H*), 3.72 (3H, s, OC*H*$_3$), 3.99–4.07 (1H, m, C(1)H^AH^B), 4.56–4.64 (1H, C(1)H^AH^B), 5.96 (1H, s, C(3)O*H*), 6.62–6.67 (2H, m, C(2)ArC(3,5)*H*), 7.02–7.08 (2H, m, C(2)ArC(2,6)*H*), 7.08–7.38 (4H, m, C(3)ArC(2,3,5,6)*H*); **^{13}C{^{1}H} NMR** (126 MHz, $CDCl_3$) δ_C: 49.3 (*C*(2)H), 55.2 (O*C*H$_3$), 66.5 (d, *J* 6.3, *C*(1)H$_2$), 81.6 (t, *J* 22.4, *C*(3)), 113.6 (C(2)Ar*C*(3,5)H), 122.2 (C(3)Ar*C*(4)), 128.6 (C(3)Ar*C*(2,6)H), 129.9 (C(2)Ar*C*(1)), 130.8 (C(3)Ar*C*(3,5)H), 131.0 (C(2)Ar*C*(2,6)H), 135.8 (C(3)Ar*C*(1)), 158.6 (C(2)Ar*C*(4)); **^{19}F NMR** (471 MHz, $CDCl_3$) δ_F: −128.0 – −124.1 (m, C*F*$_2$), −121.7 – −116.0 (m, C*F*$_2$), −114.9 – −113.3 (m, C*F*$_2$), −80.9 – −80.7 (m, C*F*$_3$); **IR** (neat) ν_{max} cm^{-1}: (3336, br OH); **HRMS** (APCI$^+$) $C_{20}H_{20}NO_3F_9Br^+$ $[M+NH_4]^+$ found 572.0485 requires 572.0477 (+1.4 ppm).

(2*R*,3*S*)-3-(4-Bromophenyl)-4,4,5,5,5-pentafluoro-2-phenylpentane-1,3-diol 67

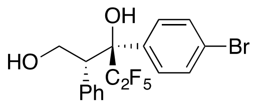

Following General Procedure E, lactone **22** (252 mg, 0.6 mmol) and DIBAL solution (1.2 mL, 1.2 mmol) in CH_2Cl_2 (6 mL) gave, after Biotage column chromatography (0→20% Et_2O in hexane), (2*R*,3*S*)-3-(4-bromophenyl)-4,4,5,5,5-pentafluoro-2-phenylpentane-1,3-diol **67** as a colourless oil (156 mg, 61%).

$[\alpha]_D^{20} = -30.3$ (*c* 1.0, $CHCl_3$); **Chiral HPLC analysis**, Chiralcel OD-H (97:3 Hexane:IPA, flow rate 1.5 $mLmin^{-1}$, 211 nm, 40 °C) t_R(2*S*,3*R*): 20.9 min, t_R(2*R*,3*S*): 30.7 min, 94:6 er; **^{1}H NMR** (500 MHz, $CDCl_3$) δ_H: 2.19 (1H, br, C(1)O*H*), 3.66–3.70 (1H, m, C(2)*H*), 4.08 (1H, app dt, *J* 11.2, 2.7, C(1)H^AH^B), 4.69 (1H, ddd, *J* 11.2, 3.5, 2.1, C(1)H^AH^B), 6.03 (1H, s, C(3)O*H*), 7.11–7.16 (3H, m, PhC(3,4,5)*H*), 7.17–7.23 (2H, m, PhC(2,6)*H*), 7.25–7.40 (4H, m, C(3)ArC(2,3,5,6)*H*); **^{13}C{^{1}H} NMR** (126 MHz, $CDCl_3$) δ_C: 49.5 (*C*(2)H), 66.4 (d, *J* 7.5, *C*(1)H$_2$), 80.3 (t, *J* 22.0, *C*(3)), 112.5–120.7 (m, *C*F$_2$*C*F$_3$), 122.3 (C(3)Ar*C*(4)), 127.3 (Ph*C*(4)H), 128.4 (Ph*C*(3,5)H), 128.4 (C(3)Ar*C*(2,6)H), 129.9 (Ph*C*(2,6)H), 130.9 (C(3)Ar*C*(3,5)H), 136.0

(d, J 5.0, C(3)ArC(1)), 138.1 (PhC(1)); **^{19}F NMR** (471 MHz, $CDCl_3$) δ_F: −119.7 (d, J 271.2, CF_2), −117.7 (dt, J 270.7, 2.2 CF_2), −77.7 (CF_3); **IR** (neat) ν_{max} cm^{-1}: (3304, br, OH); **HRMS** (APCI$^+$) $C_{17}H_{18}NO_2F_5Br^+$ $[M+NH_4]^+$ found 442.0441 requires 442.0436 (+1.6 ppm).

(2*R*,3*S*)-4,4,5,5,5-Pentafluoro-2-phenyl-3-(4-(trifluoromethyl)phenyl)pentane-1,3-diol 68

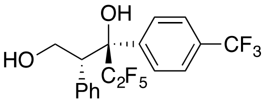

Following General Procedure E, lactone **27** (246 mg, 0.6 mmol) and DIBAL solution (1.2 mL, 1.2 mmol) in CH_2Cl_2 (6 mL) gave, after Biotage column chromatography (0→20% Et_2O in hexane), (2*R*,3*S*)-4,4,5,5,5-pentafluoro-2-phenyl-3-(4-(trifluoromethyl)phenyl)pentane-1,3-diol **68** as a colourless oil (139 mg, 56%).

$[\alpha]_D^{20}$ = −28.1 (c 1.0, $CHCl_3$); **Chiral HPLC analysis**, Chiralpak IC (99:1 Hexane:IPA, flow rate 1.5 mLmin^{-1}, 211 nm, 40 °C) t_R(2*S*,3*R*): 3.9 min, t_R(2*R*,3*S*): 4.7 min, 95:5 er; **^{1}H NMR** (500 MHz, $CDCl_3$) δ_H: 2.14 (1H, dd, J 6.0, 4.1, C(1)OH), 3.67–3.71 (1H, m, C(2)H), 4.03–4.19 (1H, m, C(1)H^AH^B), 4.63–4.81 (1H, m, C(1)H^AH^B), 6.11 (1H, s, C(3)OH), 7.07–7.13 (3H, m, PhC(2,4,6)H), 7.15–7.20 (2H, m, PhC(3,5)H), 7.30–7.90 (4H, m, C(3)ArC(2,3,5,6)H); **^{13}C{^{1}H} NMR** (126 MHz, $CDCl_3$) δ_C: 49.5 (C(2)H), 66.5 (d, $^4J_{CF}$ 7.6, C(1)H_2), 80.4 (t, J 22,1, C(3)), 112.4–122.5 (m, CF_2CF_3), 124.0 (q, J 272.0, ArCF_3), 124.7 (C(3)ArC(2,6)H), 127.1 (C(3)ArC(3,5)H), 127.4 (PhC(4)H), 128.4 (PhC(2,6)H), 129.9 (PhC(3,5)H), 130.2 (q, J 32.7, C(3)ArC(4)), 137.9 (PhC(1)), 140.9 (d, $^3J_{CF}$ 4.9, C(3)ArC(1)); **^{19}F NMR** (471 MHz, $CDCl_3$) δ_F: −119.6 (d, J 271.1, CF_2), −117.8 (d, J 271.1, CF_2), −77.7 (CF_2CF_3), −62.7 (ArCF_3); **IR** (neat) ν_{max} cm^{-1}: (3337, br OH); **HRMS** (APCI$^+$) $C_{18}H_{18}NO_2F_8^+$ $[M+NH_4]^+$ found 432.1203 requires 432.1204 (−0.2 ppm).

(2*R*,3*S*)-3-(4-Bromophenyl)-4,4,4-trifluoro-2-phenylbutane-1,3-diol 69

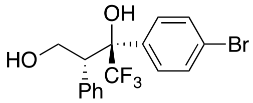

Following General Procedure E, lactone **23** (223 mg, 0.6 mmol) and DIBAL solution (1.2 mL, 1.2 mmol) in CH_2Cl_2 (6 mL) gave, after Biotage column chromatography (0→20% Et_2O in hexane), (2*R*,3*S*)-3-(4-bromophenyl)-4,4,4-trifluoro-2-phenylbutane-1,3-diol **69** as a colourless oil (164 mg, 73%).

$[\alpha]_D^{20}$ = −29.4 (c 1.0, $CHCl_3$); **Chiral HPLC analysis**, Chiralpak IC (97:3 Hexane:IPA, flow rate 1.5 mLmin^{-1}, 211 nm, 40 °C) t_R(2*S*,3*R*): 4.7 min, t_R(2*R*,3*S*): 5.8 min, 94:6 er; **^{1}H NMR** (500 MHz, $CDCl_3$) δ_H: 2.13–2.20 (1H, m, C(1)OH), 3.53 (1H, app t, J 3.2, C(2)H), 4.04–4.11 (1H, m, C(1)H^AH^B), 4.60–4.67 (1H, m, C(1)H^AH^B), 5.76 (1H, s, C(3)OH), 7.12–7.16 (3H, m, PhC(2,4,6)H), 7.17–7.21 (2H, PhC(3,5)H), 7.25–7.29 (2H, m, C(3)ArC(2,6)H), 7.30–7.34 (2H, m, C(3)ArC(3,5)H); **^{13}C{^{1}H} NMR** (126 MHz, $CDCl_3$) δ_C:

49.3 (C(2)H), 65.9 (d, $^4J_{CF}$ 3.1, C(1)H_2), 80.7 (q, $^2J_{CF}$ 27.2, C(3)), 122.3 (C(3)ArC(4)), 125.8 (q, J 288.3, CF_3), 127.3 (PhC(4)H), 128.1 (C(3)ArC(2,6)H), 128.4 (PhC(2,6)H), 129.8 (PhC(3,5)H), 131.1 (C(3)ArC(3,5)H), 136.7 (C(3)ArC(1)), 137.9 (PhC(1)); **^{19}F NMR** (471 MHz, $CDCl_3$) δ_F: −74.9 (CF_3); **IR** (neat) ν_{max} cm^{-1}: 3352 (br, OH); **HRMS** (APCI+) $C_{16}H_{18}NO_2F_3Br$ $[M+NH_4]^+$ found 392.0466 requires 392.0468 (−0.5 ppm).

(2*S*,3*R*)-2-(4-Bromophenyl)-2-(perfluorobutyl)-3-phenyloxetane 70

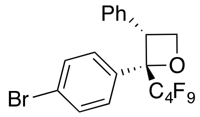

Following General Procedure F, diol **65** (107 mg, 0.21 mmol), NaH (17 mg, 0.42 mmol) and 2,4,6-triisopropylbenzenesulfonyl chloride (56 mg, 0.19 mmol) in THF (10 mL) gave, after Biotage column chromatography (0→1% Et_2O in hexane), (2*S*,3*R*)-2-(4-bromophenyl)-2-(perfluorobutyl)-3-phenyloxetane **XX** as a colourless oil (102 mg, 96%).

$[\alpha]_D^{20}$ = +115.6 (*c* 1.0, $CHCl_3$); **Chiral HPLC analysis**, Chiralcel OD-H (97:3 Hexane:IPA, flow rate 1.5 mLmin^{-1}, 211 nm, 40 °C) t_R(2*S*,3*R*): 3.5 min, t_R(2*R*,3*S*): 6.3 min, 97:3 er; **^{1}H NMR** (500 MHz, $CDCl_3$) δ_H: 4.78 (1H, app t, J 6.5, C(4)H^AH^B), 4.85 (1H, dd, J 8.6, 6.9, C(3)H), 5.14 (1H, dd, J 8.5, 6.0, C(4)H^AH^B), 6.80–7.06 (4H, m, PhC(2,6)H + C(2)ArC(2,6)H), 7.13–7.21 (3H, m, PhC(3,4,5)H), 7.24–7.33 (2H, m, C(2)ArC(3,5)H); **^{13}C{^{1}H} NMR** (126 MHz, $CDCl_3$) δ_C: 45.4 (t, $^3J_{CF}$ 3.6, C(3)H), 73.9 (C(4)H_2), 89.8–90.3 (m, C(2)), 105.9–121.3 (m, $CF_2CF_2CF_2CF_3$), 122.8 (C(2)ArC(4)), 128.2 (PhC(4)H), 128.5 (PhC(3,5)H), 128.6 (C(2)ArC(2,6)H), 129.3 (PhC(2,6)H), 130.9 (C(2)ArC(3,5)H), 132.4 (d, $^3J_{CF}$ 3.8, C(2)ArC(1)), 135.0 (PhC(1)); **^{19}F NMR** (471 MHz, $CDCl_3$) δ_F: −128.2 – −127.3 (m, CF_2), −125.4 – −124.5 (m, CF_2), −123.8 – −123.0 (m, CF_2), −122.9 – −122.1 (m, CF_2), −121.7 – −120.8 (m, CF_2), −118.5 – −117.6 (m, CF_2), −80.9 – −80.8 (m, CF_3); **IR** (neat) ν_{max} cm^{-1}: 1234 (C-O), 1134 (C-O); **HRMS** could not be obtained.

(2*S*,3*R*)-2-(4-Bromophenyl)-3-(4-methoxyphenyl)-2-(perfluorobutyl)oxetane 71

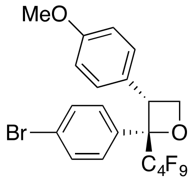

Following General Procedure F, diol **66** (194 mg, 0.35 mmol), NaH (28 mg, 0.70 mmol) and 2,4,6-triisopropylbenzenesulfonyl chloride (93 mg, 0.31 mmol) in THF (17 mL) gave, after Biotage column chromatography (0→3% Et_2O in hexane), (2*S*,3*R*)-2-(4-bromophenyl)-3-(4-methoxyphenyl)-2-(perfluorobutyl)oxetane **71** as a colourless oil (152 mg, 92%).

$[\alpha]_D^{20}$ = +108.5 (*c* 1.0, $CHCl_3$); **Chiral HPLC analysis**, Chiralcel OD-H (97:3 Hexane:IPA, flow rate 1.5 $mLmin^{-1}$, 211 nm, 40 °C) t_R(2*S*,3*R*): 3.7 min, t_R(2*R*,3*S*): 5.1 min, 97:3 er; **^{1}H NMR** (500 MHz, $CDCl_3$) δ_H: 3.75 (3H, s, C*H_3*), 4.72 (1H, app t, *J* 6.6, C(4)H^AH^B), 4.80 (1H, dd, *J* 8.7, 7.1, C(3)*H*), 5.12 (1H, dd, *J* 8.7, 6.1, C(4)H^AH^B), 6.67–6.72 (2H, m, C(3)ArC(3,5)*H*), 6.84–6.90 (2H, m, C(3)ArC(2,6)*H*), 6.91 (2H, br, C(2)ArC(2,6)*H*), 7.28–7.37 (2H, m, C(2)ArC(3,5)*H*); **^{13}C{^{1}H} NMR** (126 MHz, $CDCl_3$) δ_C: 44.8 (t, $^3J_{CF}$ 3.6, *C*(3)H), 55.4 (O*C*H_3), 74.4 (*C*(4)H_2), 89.9–90.5 (m, *C*(2)), 105.4–122.1 ($CF_2CF_2CF_2CF_3$), 113.8 (C(3)Ar*C*(3,5)H), 122.8 (C(2)Ar*C*(4)), 127.1 (C(3)Ar*C*(1)), 128.7 (d, $^4J_{CF}$ 3.1, C(2)Ar*C*(2,6)H), 130.4 (C(3)Ar*C*(2,6)H), 130.9 (C(2)Ar*C*(3,5)H), 132.5 (d, $^3J_{CF}$ 3.5, C(2)Ar*C*(1)), 159.3 (C(3)Ar*C*(4)); **^{19}F NMR** (471 MHz, $CDCl_3$) δ_F: −128.2 – −127.3 (m, CF_2), −125.4 – −124.5 (m, CF_2), −123.8 – −122.9 (m, CF_2), −122.9 – −122.1 (m, CF_2), −121.8 – −121.0 (m, CF_2), −118.5 – −117.6 (m, CF_2), −80.9 – −80.8 (m, CF_3); **IR** (neat) ν_{max} cm^{-1}: 1234 (C-O), 1134 (C-O); **HRMS** ($APCI^+$) $C_{20}H_{15}O_2F_9Br^+$ $[M+H]^+$ found 537.0112 requires 537.0106 (+1.1 ppm).

(2*S*,3*R*)-2-(4-Bromophenyl)-2-(perfluoroethyl)-3-phenyloxetane 72

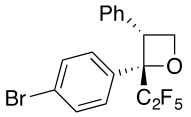

Following General Procedure F, diol **67** (104 mg, 0.24 mmol), NaH (20 mg, 0.49 mmol) and 2,4,6-triisopropylbenzenesulfonyl chloride (64 mg, 0.22 mmol) in THF (12 mL) gave, after Biotage column chromatography (0→1% Et_2O in hexane), (2*S*,3*R*)-2-(4-bromophenyl)-3-phenyl-2-(trifluoromethyl)oxetane **72** as a colourless oil (94 mg, 92%).

$[\alpha]_D^{20}$ = +115.6 (*c* 1.0, $CHCl_3$); **Chiral HPLC analysis**, Chiralcel OD-H (97:3 Hexane:IPA, flow rate 1.5 $mLmin^{-1}$, 211 nm, 40 °C) t_R(2*S*,3*R*): 4.2 min, t_R(2*R*,3*S*): 6.2 min, 94:6 er; **^{1}H NMR** (500 MHz, $CDCl_3$) δ_H: 4.77 (1H, dd, *J* 6.9, 5.9, C(4)H^AH^B), 4.83 (1H, dd, *J* 8.3, 6.9, C(3)*H*), 5.14 (1H, dd, *J* 8.4, 5.9, C(4)H^AH^B), 6.82–6.95 (2H, m, C(2)ArC(2,6)*H*), 6.96–7.01 (2H, m, PhC(2,6)*H*), 7.12–7.21 (3H, m, Ph(3,4,5)*H*), 7.24–7.31 (2H, m, C(2)ArC(3,5)*H*); **^{13}C{^{1}H} NMR** (126 MHz, $CDCl_3$) δ_C: 45.0 (t, *J* 3.3, *C*(3)H), 74.0 (*C*(4)H_2), 88.9 (dd, *J* 27.9, 24.2, *C*(2)), 110.7–120.6 (m, CF_2CF_3), 122.8 (C(2)Ar*C*(4)), 128.1 (Ph*C*(4)H), 128.4 (d, *J* 3.1, C(2)Ar*C*(2,6)H), 128.5 (Ph*C*(3,5)H), 129.3 (Ph*C*(2,6)H), 130.9 (C(2)Ar*C*(3,5)H), 132.6 (d, *J* 3.6, C(2)Ar*C*(1)), 135.2 (Ph*C*(1)); **^{19}F NMR** (471 MHz, $CDCl_3$) δ_F: −126.3 (d, *J* 278.2, CF_2), −125.0 (d, *J* 278.2, CF_2), −78.8 (CF_3); **IR** (neat) ν_{max} cm^{-1}: 1231 (C-O), 1137 (C-O); **HRMS** ($APCI^+$) $C_{17}H_{14}NF_5Br^+$ $[M-H_2O+NH_4]^+$ found 406.0222 requires 406.0224 (−0.5 ppm).

(2*S*,3*R*)-2-(Perfluoroethyl)-3-phenyl-2-(4-(trifluoromethyl)phenyl)oxetane 73

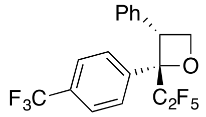

Following General Procedure F, diol **68** (105 mg, 0.25 mmol), NaH (20 mg, 0.50 mmol) and 2,4,6-triisopropylbenzenesulfonyl chloride (67 mg, 0.23 mmol) in THF (12 mL) gave, after Biotage column chromatography (0→3% Et_2O in hexane), (2*S*,3*R*)-2-(4-bromophenyl)-3-phenyl-2-(trifluoromethyl)oxetane **73** as a colourless oil (115 mg, 98%).

$[\alpha]_D^{20}$ = +91.6 (*c* 1.0, $CHCl_3$); **Chiral HPLC analysis**, Chiralcel OD-H (97:3 Hexane:IPA, flow rate 1.5 mLmin^{-1}, 211 nm, 40 °C) t_R(2*S*,3*R*): 4.2 min, t_R(2*R*,3*S*): 6.0 min, 95:5 er; **^{1}H NMR** (500 MHz, $CDCl_3$) δ_H: 4.80 (1H, app t, *J* 6.5, C(4)H^AH^B), 4.87 (1H, dd, *J* 8.5, 6.8, C(3)*H*), 5.18 (1H, dd, *J* 8.5, 6.1, C(4)H^AH^B), 6.93–6.99 (2H, m, PhC(2,6)*H*), 7.07–7.21 (5H, m, PhC(3,4,5)*H* + C(2)ArC(2,6)*H*), 7.34–7.48 (2H, m, C(2)ArC(3,5)*H*); **^{13}C{^{1}H} NMR** (126 MHz, $CDCl_3$) δ_C: 45.2 (d, $^3J_{CF}$ 3.5 *C*(3)H), 74.1 (*C*(4)H_2), 88.9 (dd, *J* 27.4, 24.6, *C*(2)), 112.8–121.1 (m, CF_2CF_3), 124.0 (d, $^1J_{CF}$ 272.3, ArCF_3) 124.6 (q, $^3J_{CF}$ 3.6, C(2)Ar*C*(3,5)H), 127.2 (m, C(2)Ar*C*(2,6)H), 128.2 (Ph*C*(4)H), 128.5 (Ph*C*(3,5)H) 129.2 (Ph*C*(2,6)H), 130.5 (q, *J* 32.4, C(2)Ar*C*(4)), 134.9 (Ph*C*(1)), 137.6 (C(2)Ar*C*(1)); **^{19}F NMR** (471 MHz, $CDCl_3$) δ_F: −126.1 (d, *J* 279, CF_2), −125.0 (d, *J* 279, CF_2), −78.8 (CF_2CF_3), −62.8 (ArCF_3); **IR** (neat) ν_{max} cm^{-1}: 1327 (C-O), 1130 (C-O); **HRMS** could not be obtained.

(2*S*,3*R*)-2-(4-Bromophenyl)-3-phenyl-2-(trifluoromethyl)oxetane 74

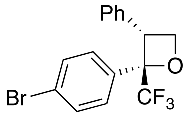

Following General Procedure F, diol **69** (111 mg, 0.30 mmol), NaH (24 mg, 0.60 mmol) and 2,4,6-triisopropylbenzenesulfonyl chloride (79 mg, 0.27 mmol) in THF (15 mL) gave, after Biotage column chromatography (0→3% Et_2O in hexane), (2*S*,3*R*)-2-(4-bromophenyl)-3-phenyl-2-(trifluoromethyl)oxetane **74** as a colourless oil (115 mg, 98%).

$[\alpha]_D^{20}$ = +127.8 (*c* 1.0, $CHCl_3$); **Chiral HPLC analysis**, Chiralcel OD-H (97:3 Hexane:IPA, flow rate 1.5 mLmin^{-1}, 211 nm, 40 °C) t_R(2*S*,3*R*): 4.1 min, t_R(2*R*,3*S*): 6.7 min, 94:6 er; **^{1}H NMR** (500 MHz, $CDCl_3$) δ_H: 4.61 (1H, dd, *J* 8.5, 6.5, C(3)*H*), 4.76 (1H, app t, *J* 6.4, C(4)H^AH^B), 5.19 (1H, dd, *J* 8.5, 6.2, C(4)H^AH^B), 6.87–6.95 (2H, m, C(2)ArC(2,6)*H*), 6.98–7.05 (2H, m, PhC(2,6)*H*), 7.15–7.22 (3H, m, PhC(3,4+5)*H*), 7.27–7.33 (2H, m, C(2)ArC(3,5)*H*); **^{13}C{^{1}H} NMR** (126 MHz, $CDCl_3$) δ_C: 44.9 (*C*(3)H), 74.1 (*C*(4)H_2), 88.6 (q, $^2J_{CF}$ 30.7, *C*(2)), 122.8 (C(2)Ar*C*(4)), 124.9 (q, *J* 284.4, CF_3), 128.1 (Ph*C*(4)H), 128.5 (C(2)Ar*C*(2,6)H), 128.6 (Ph*C*(3,5)H), 129.1 (Ph*C*(2,6)H), 131.0 (C(2)Ar*C*(3,5)H), 132.7 (Ph*C*(1)), 135.4 (C(2)Ar*C*(1)); **^{19}F NMR** (471 MHz, $CDCl_3$) δ_F: -81.6 (CF_3); **IR** (neat) ν_{max} cm^{-1}: 1166 (C-O); **HRMS** could not be obtained.

Epimerisation Studies

i)

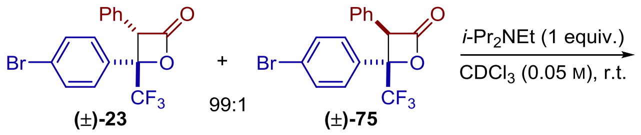

i-Pr_2NEt (5 µL, 0.03 mmol) was added to a sample containing both diastereoisomers of 4-(4-bromophenyl)-3-phenyl-4-(trifluoromethyl)oxetan-2-one **23** and **75** (11 mg, 0.03 mmol, 99:1 dr) in $CDCl_3$ (0.6 mL) at r.t., and the ratio of **23**:**75** assessed over time by ^{1}H NMR spectroscopy. A steady ratio of 90:10 **23**:**75** was attained within 4 h.

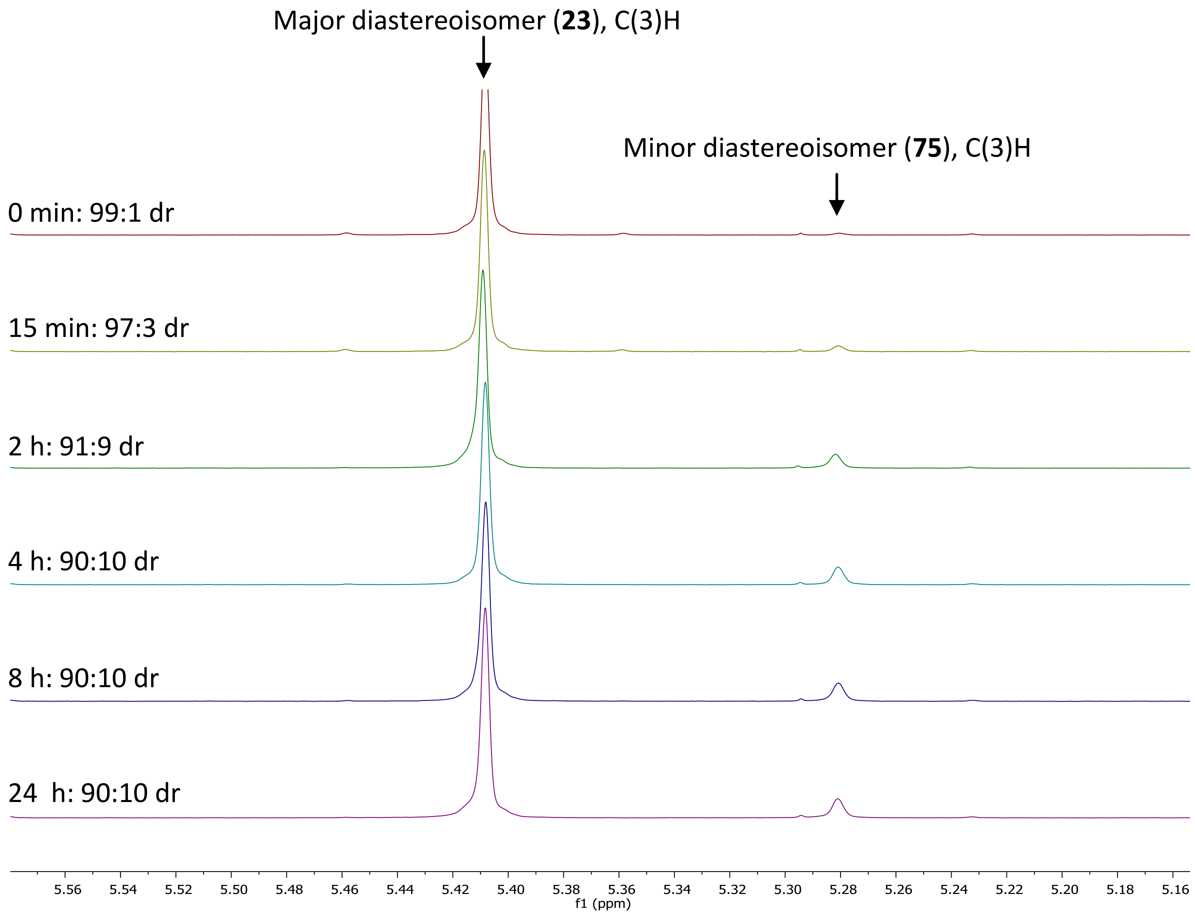

ii)

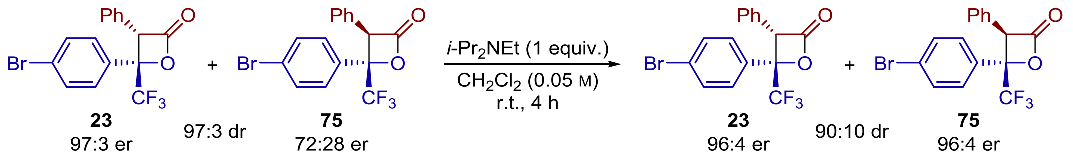

i-Pr_2NEt (5 µL, 0.03 mmol) was added to a sample of **23** and **75** (11 mg, 0.03 mmol, 97:3 dr) [obtained from a catalytic reaction using (2*S*,3*R*)-HyperBTM at 90 °C, in which both diastereoisomers enantioenriched (**23**: 97:3 er; **75**: 72:28 er)] in CH_2Cl_2 (0.6 mL) at r.t., and was allowed to stir for 4 h. The reaction was diluted with CH_2Cl_2 (5 mL), washed sequentially with 0.1 M HCl (2 × 5 mL) and brine.

The organic layer was dried ($MgSO_4$), filtered and concentrated *in vacuo* to give 4-(4-bromophenyl)-3-phenyl-4-(trifluoromethyl)oxetan-2-one **23** and **75** in a 90:10 ratio (determined by ^{1}H NMR spectroscopy). The enantiomeric ratio of each diastereoisomer was determined by HPLC analysis (Chiralcel OD-H, 97:3 Hexane:IPA, flow rate 1.5 mLmin^{-1}, 211 nm, 40 °C):

HPLC analysis prior to epimerisation with *i*-Pr_2NEt:

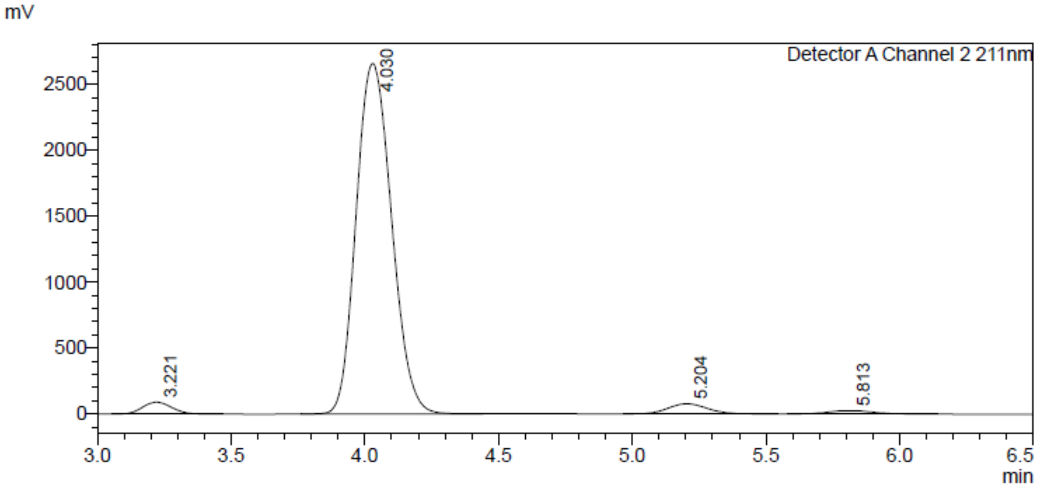

<Peak Table>

Detector A Channel 2 211nm

Peak#	Ret. Time	Area%
1	3.221	2.625
2	4.030	93.137
3	5.204	3.049
4	5.813	1.189
Total		100.000

23: t_R(3*R*,4*R*): 3.2 min, t_R(3*S*,4*S*): 4.0 min, 3:97 er

75: t_R(3*R*,4*S*): 5.2 min, t_R(3*S*,4*R*): 5.8 min, 72:28 er

HPLC analysis after epimerisation with *i*-Pr_2NEt:

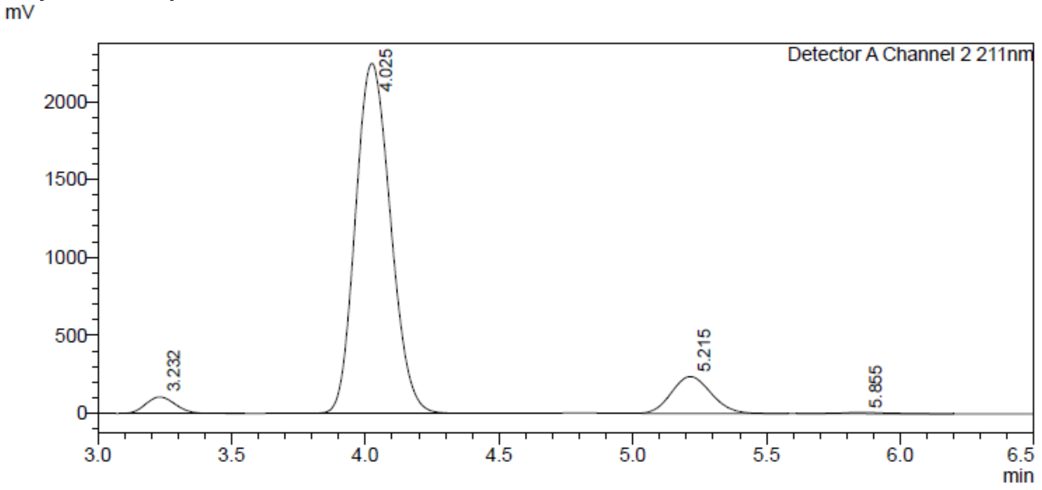

<Peak Table>

Detector A Channel 2 211nm

Peak#	Ret. Time	Area%
1	3.232	3.438
2	4.025	85.851
3	5.215	10.320
4	5.855	0.390
Total		100.000

23: t_R(3*R*,4*R*): 3.2 min, t_R(3*S*,4*S*): 4.0 min, 4:96 er

75: t_R(3*R*,4*S*): 5.2 min, t_R(3*S*,4*R*): 5.9 min, 96:4 er

Following epimerisation, enhancement of the stereoisomer with a retention time of 5.2 min was observed. Assuming this increase in the amount of this stereoisomer is due to epimerisation of the major (3*S*,4*S*) stereoisomer at C(3) indicates this stereoisomer has a (3*R*,4*S*) configuration. This confirms that the major enantiomer of the minor diastereoisomer isolated from the catalytic reaction has a (3*R*,4*S*) configuration. This configuration at C(3) is the opposite to that which is usually expected for products formed under the control of the isothiourea catalyst. In combination with the fact that the dr of the product isolated from the isothiourea-catalysed reaction (90:10) is equal to the thermodynamically-favoured ratio, it is therefore likely that the inherent dr of the reaction is significantly higher, with epimerisation of the major (3*S*,4*S*) stereoisomer leading to formation of the (3*R*,4*S*) stereoisomer and resulting in a reduction in the dr of the isolated product.

An approximation of the abundance of each stereoisomer prior to, and after, the epimerisation experiment is given below, which may aid visualisation of how the er of the minor diastereoisomer is enhanced through this process.

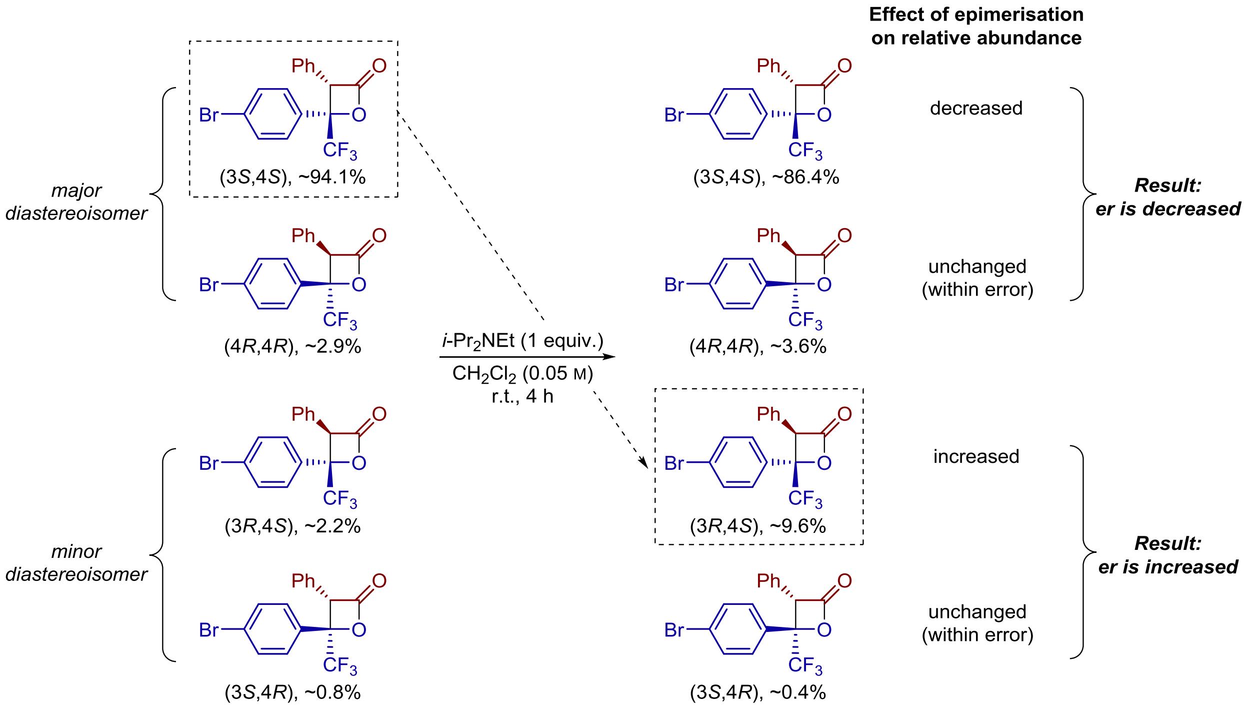

iii)

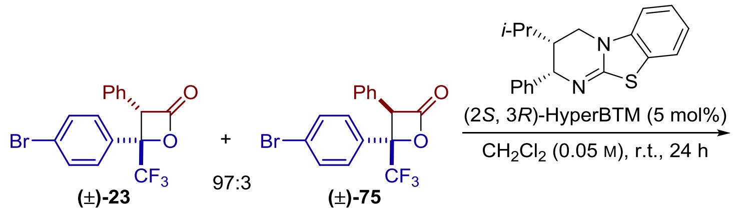

To investigate the possibility of product epimerisation using the chiral isothiourea catalyst leading to a kinetic resolution, (2*S*, 3*R*)-HyperBTM (0.5 mg, 0.0015 mmol) was added to a racemic sample of both diastereoisomers of 4-(4-bromophenyl)-3-phenyl-4-(trifluoromethyl)oxetan-2-one **23** and **75** (11 mg, 0.03 mmol, 97:3 dr) in CH_2Cl_2 (0.6 mL) at r.t. and allowed to stir for 24 h. The reaction was diluted with CH_2Cl_2 (5 mL), washed sequentially with 0.1 M HCl (2 × 5 mL) and brine. The organic layer was dried ($MgSO_4$), filtered and concentrated *in vacuo* to give 4-(4-bromophenyl)-3-phenyl-4-(trifluoromethyl)oxetan-2-one **23** and **75** in a 90:10 ratio (determined by ^{1}H NMR spectroscopy). The enantiomeric ratio of each diastereoisomer was determined by HPLC analysis using a chiral support (Chiralcel OD-H, 97:3 Hexane:IPA, flow rate 1.5 mLmin^{-1}, 211 nm, 40 °C):

HPLC analysis prior to epimerisation with (2*S*, 3*R*)-HyperBTM:

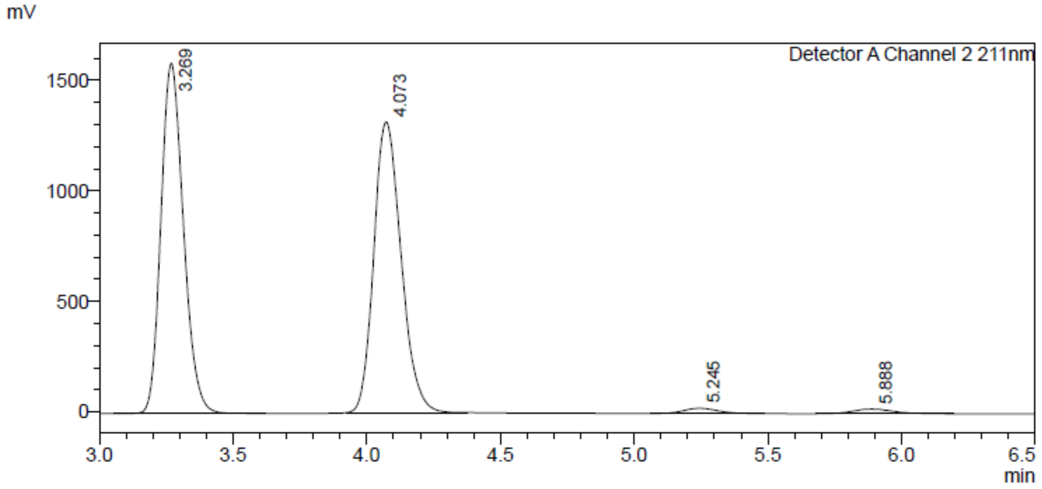

<Peak Table>

Detector A Channel 2 211nm

Peak#	Ret. Time	Area%
1	3.269	48.691
2	4.073	49.034
3	5.245	1.142
4	5.888	1.134
Total		100.000

23: t_R(3*R*,4*R*): 3.2 min, t_R(3*S*,4*S*): 4.1 min, 50:50 er

75: t_R(3*R*,4*S*): 5.2 min, t_R(3*S*,4*R*): 5.9 min, 50:50 er

HPLC analysis after 24 h epimerisation with (2*S*, 3*R*)-HyperBTM:

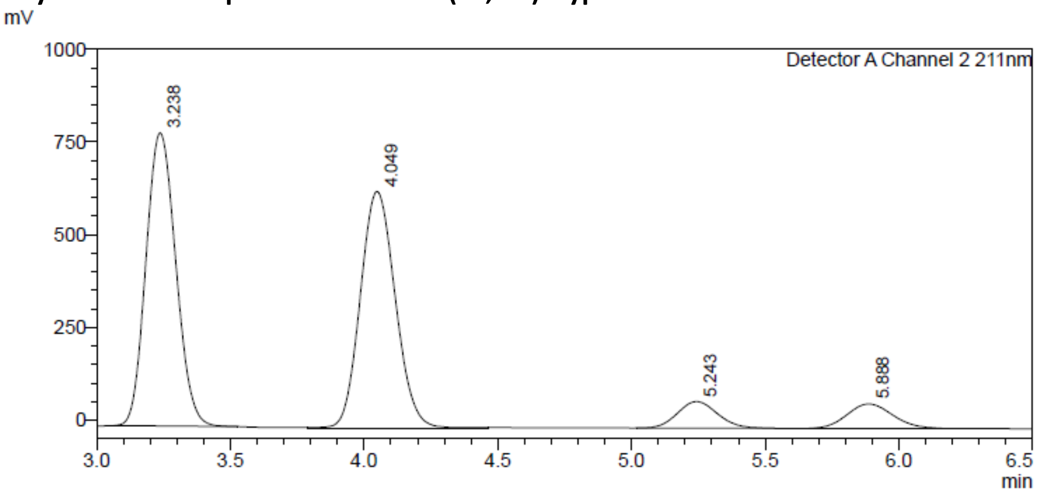

<Peak Table>

Detector A Channel 2 211nm

Peak#	Ret. Time	Area%
1	3.238	45.549
2	4.049	43.005
3	5.243	5.620
4	5.888	5.825
Total		100.000

23: t_R(3*R*,4*R*): 3.2 min, t_R(3*S*,4*S*): 4.0 min, 51:49 er

75: t_R(3*R*,4*S*): 5.2 min, t_R(3*S*,4*R*): 5.9 min, 49:51 er

Only minimal enantioenrichment of each diastereoisomer was observed, indicating that a kinetic resolution process may be operative, but is unlikely to play a significant role in determining the distribution of stereoisomers obtained from the catalytic reaction.

Thermal Stability of β-Lactones 21–23

To test the thermal stability of the β-lactone products, samples of β-lactones **21**–**23** were dissolved in d_6-DMSO and, in an NMR tube, heated for various periods of time. After heating each sample sequentially at 70 °C, 100 °C and 120 °C for 2 h each no decomposition was observed. Upon heating at 140 °C some decomposition was observed in each case after 2 h, and therefore heating was continued at this temperature until the majority of the compound had undergone decomposition. The rate of decomposition was assessed through integration of the C(3) proton of each diastereoisomer of β-lactone (in example below at 6.45 ppm and 6.25 ppm) and compared to the total integration of the aromatic region. An example showing the decomposition of C_4F_9-substituted β-lactone **21** is given below:

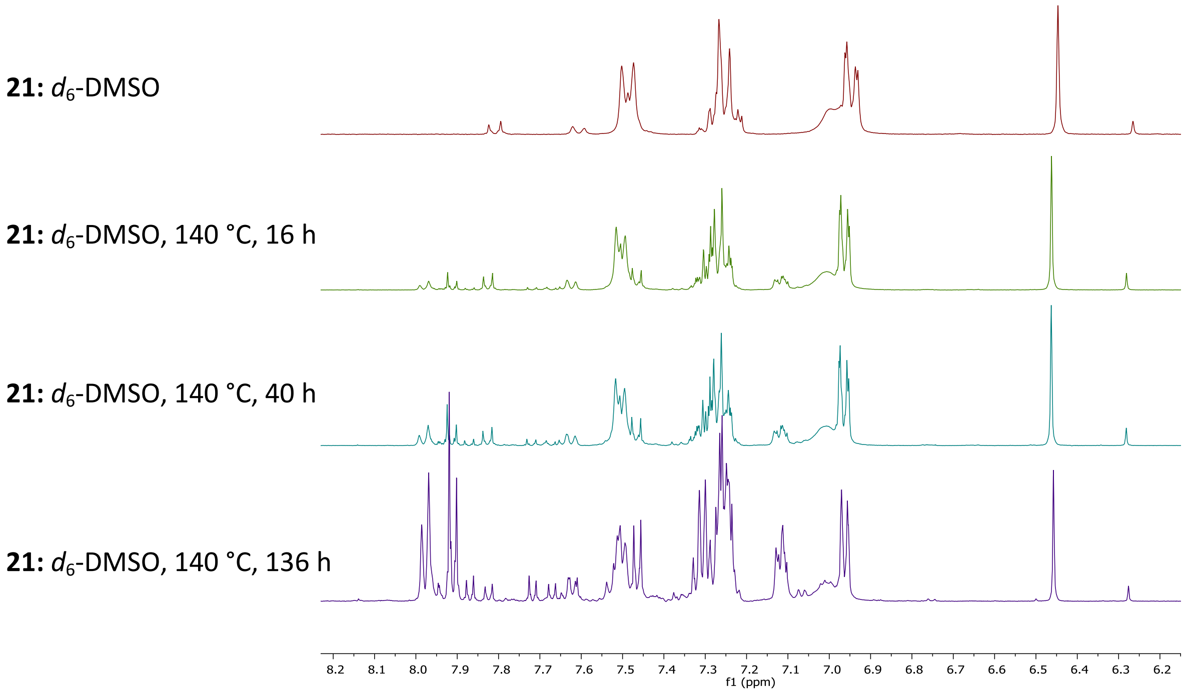

Approximate half-lives for each compound at 140 °C were calculated by plotting decomposition over time and fitting an exponential decay function:

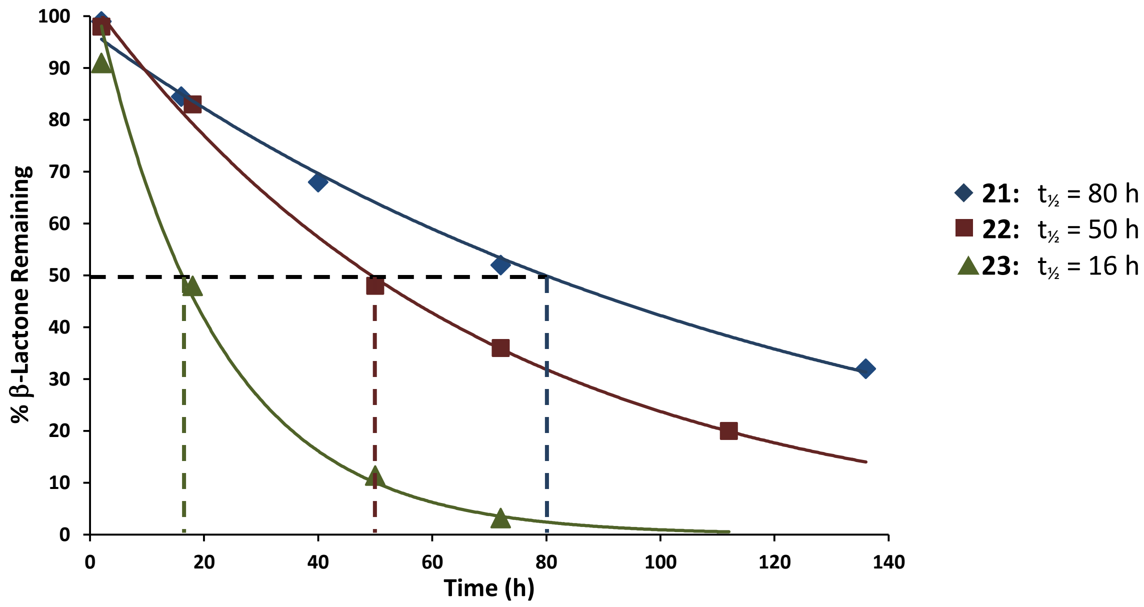

Determination of ^{13}C KIEs at Natural Abundance

i) Preparation of standard sample

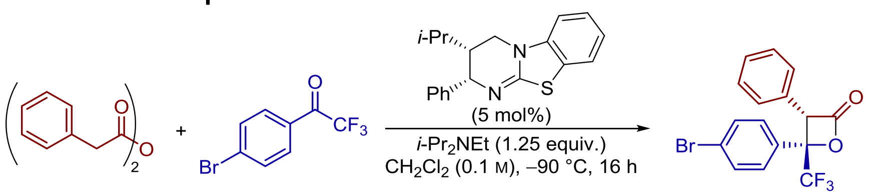

17 (1 equiv.) **20** (1 equiv.) **23**, 92%, 90:10 dr
97:3 er (major)
72:28 (major)

(2*S*,3*R*)-HyperBTM (12 mg, 0.038 mmol) and *i*-Pr_2NEt (163 µL, 0.94 mmol) were added to a solution of 1-(4-bromophenyl)-2,2,2-trifluoroethan-1-one **20** (191 mg, 0.75 mmol) and phenylacetic anhydride **17** (190 mg, 0.75 mmol) in CH_2Cl_2 (7.5 mL) at −90 °C under a nitrogen atmosphere, and the mixture was allowed to stir for 16 h. The reaction mixture was diluted with CH_2Cl_2 (15 mL), 0.1 M HCl (15 mL) was added, and the layers separated. The aqueous layer was extracted with CH_2Cl_2 (15 mL) and the combined organic layers were washed sequentially with 0.1 M HCl (15 mL) and brine (15 mL), dried ($MgSO_4$) and concentrated *in vacuo* to give, after purification by Biotage column chromatography (0→3% Et_2O in hexane), (3*S*,4*S*)-4-(4-bromophenyl)-3-phenyl-4-(trifluoromethyl)oxetan-2-one **23** as a colourless oil (256 mg, 0.69 mmol, 92%) as an inseparable mixture of diastereoisomers [97:3 dr (crude dr = 90:10)].

ii) Preparation of sample at low conversion to assess isotopic composition at C(2) and C(3)

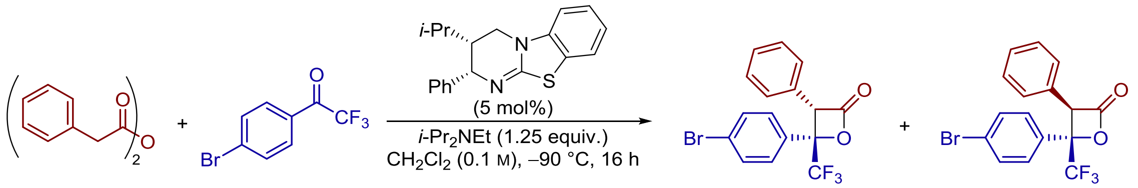

17 (1 equiv.) **20** (0.05 equiv.) **23** **75**

(2*S*,3*R*)-HyperBTM (184 mg, 0.6 mmol) and *i*-Pr_2NEt (2.6 mL, 0.94 mmol) were added to a solution of 1-(4-bromophenyl)-2,2,2-trifluoroethan-1-one (152 mg, 0.6 mmol) and phenylacetic anhydride (3.05 g, 12 mmol) in CH_2Cl_2 (120 mL) at −90 °C under a nitrogen atmosphere, and the mixture was allowed to stir for 16 h. 0.1 M HCl (250 mL) was added and the mixture allowed to warm to r.t. The layers separated and the aqueous layer was extracted with CH_2Cl_2 (2 × 50 mL) and the combined organic layers washed sequentially with 0.1 M HCl (50 mL) and brine (50 mL), dried ($MgSO_4$) and concentrated *in vacuo* to give the crude reaction product mixture. This residue was completely dissolved in $CDCl_3$, 1,2-dimethoxyethane (125 µL, 1.2 mmol) was added as an internal standard, and a sample was taken for analysis by ^1H NMR spectroscopy. The reaction conversion was assessed integrating the C(3)H proton of both diastereoisomers, and comparing the integration to that of the internal standard. Purification of the crude reaction product was performed by Biotage column

chromatography (0→3% Et_2O in hexane) to give (3*S*,4*S*)-4-(4-bromophenyl)-3-phenyl-4-(trifluoromethyl)oxetan-2-one as a colourless oil as an inseparable mixture of diastereoisomers.

Expt. number	Conversion[a,b]	Isolated yield[b]	Isolated dr (23:75)
1	3.9%	102 mg, 2.3%	93:7
2	3.8%	124 mg, 2.8%	90:10
3	3.6%	91 mg, 2.0%	90:10

[a] Determined by ^1H NMR spectroscopic analysis using 1,2-dimethoxyethane as an internal standard.
[b] Reaction conversion and yield given relative to the amount of anhydride used. For example, 3.9% = 12 mmol × 0.039 = 0.47 mmol.

Determination of T1 relaxation times

T1 relaxation time determination was performed using the inversion-recovery method for (3*S*,4*S*)-4-(4-bromophenyl)-3-phenyl-4-(trifluoromethyl)oxetan-2-one using a 93:7 mixture of diastereoisomers of **23** and **75** (180 mg, 0.49 mmol) in $CDCl_3$ (0.6 mL) at r.t. T1 relaxation times were only calculated for the major diastereoisomer **23**.

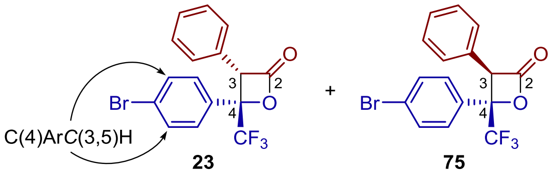

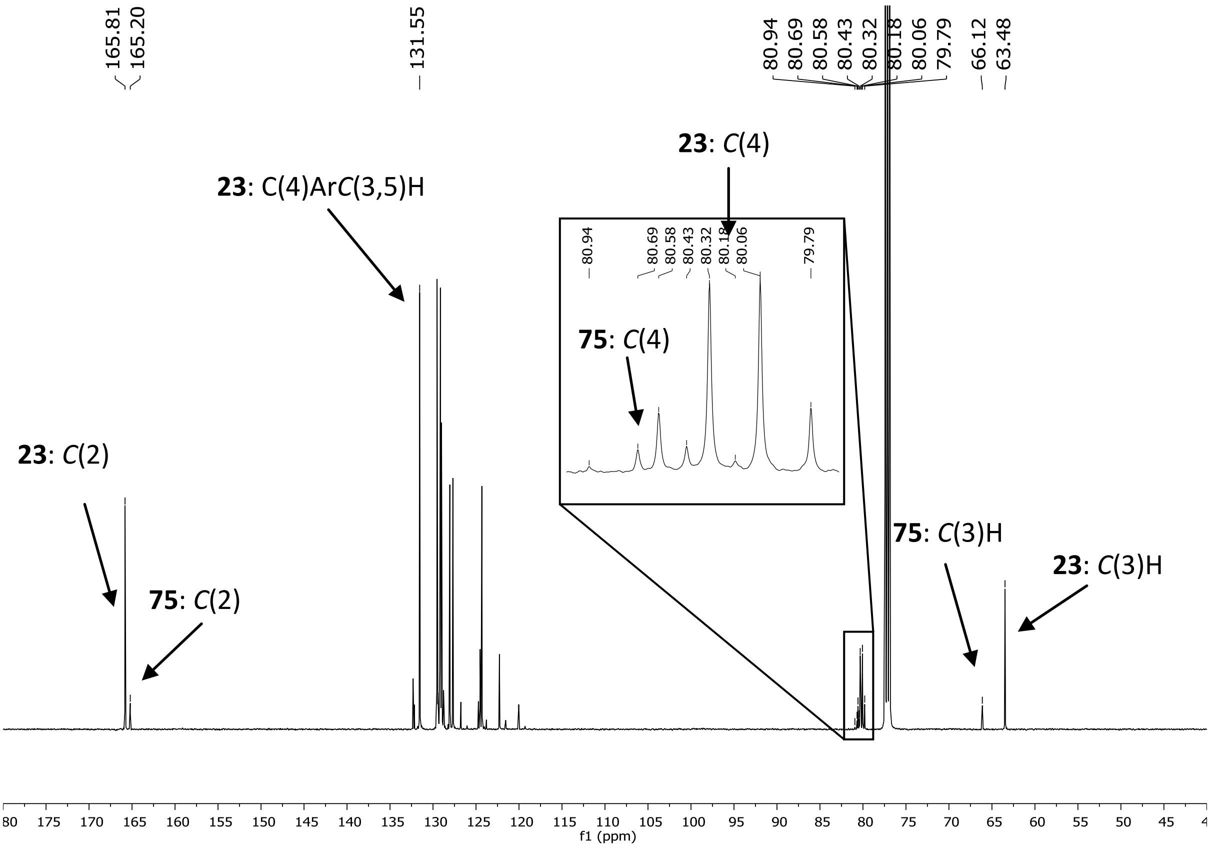

Based on the overlap of the *C*(4) signals for the two diastereoisomers, the natural abundance KIE for this carbon was not calculated, with the T1 relaxation time data given below purely for reference.

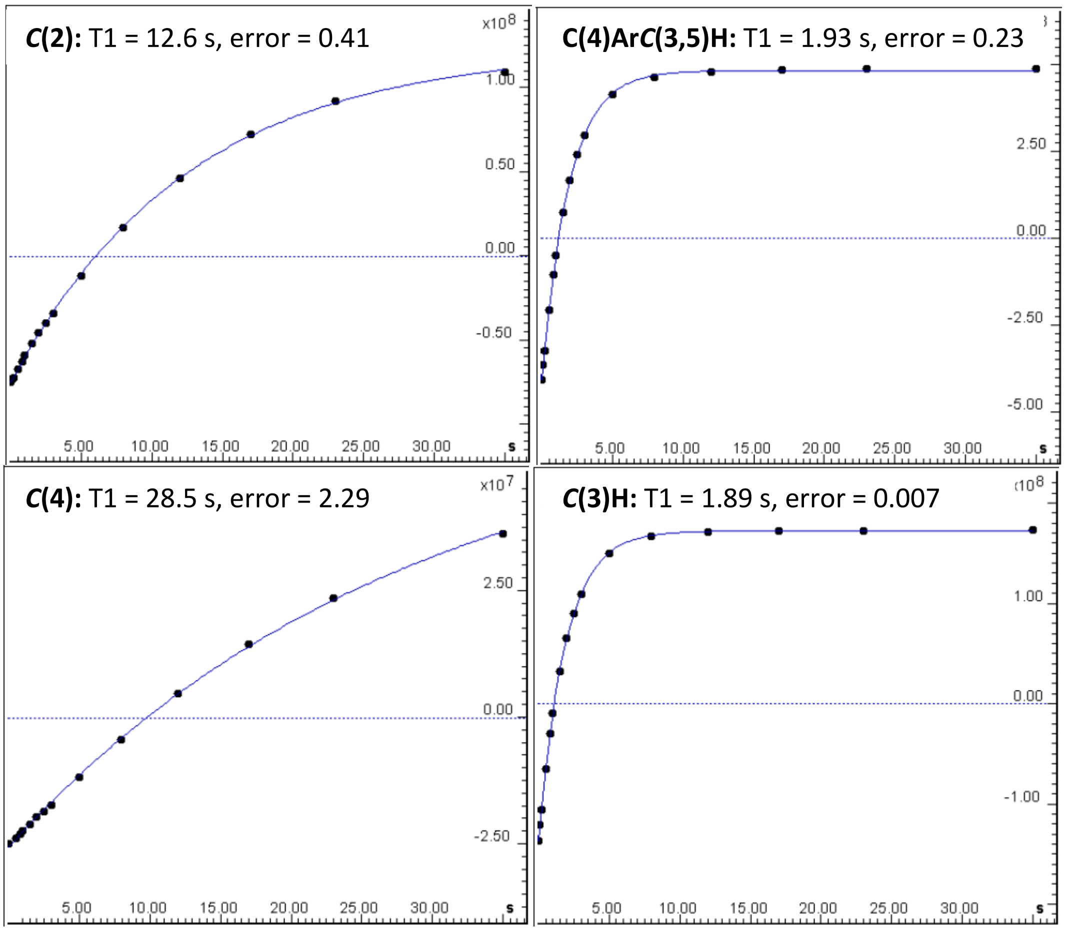

Quantitative ^{13}C NMR spectra used for natural abundance KIE calculation were therefore conducted with a D1 delay of 101 s, as this is equal to 8 times the T1 relaxation time of C(2) (the carbon with the longest T1 of those being used in the subsequent calculations).

Calculation of ^{13}C KIEs at natural abundance

^{13}C NMR spectra were recorded using a Bruker Avance II 500 spectrometer equipped with a CryoProbe Prodigy BBO probe, with inverse gated decoupling, 101 s delays between pulses, at a controlled temperature of 25 °C, collecting 64K points and using a spectral width of 29700 Hz.

Five quantitative ^{13}C NMR spectra were obtained for the standard sample. For each spectra, manual phase and baseline corrections were performed in MestReNova, and the signal corresponding to the C(4)ArC(3,5)H carbon was integrated and set to 1000. The signals for the C(2) and C(3) carbon were integrated in each spectra, and the mean and standard deviation calculated for each set. Using the inbuilt MestReNova script tool, the signal to noise ratio was calculated as > 500 in each case.

Standard Sample

	1	2	3	4	5	Mean	St. dev.
**C(2)**	478.05	482.28	481.21	484.26	487.92	482.744	3.663718
**C(3)H**	486.65	487.45	484.79	484.84	480.23	484.792	2.798718

Five quantitative ^{13}C NMR spectra were next obtained for each of the three experiments conducted to low conversion. For each spectra, manual phase and baseline corrections were performed in MestReNova, and the signal corresponding to the C(4)ArC(3,5)H carbon was integrated and set to 1000. The signals for the C(2) and C(3) carbon were integrated in each spectra, and the mean and standard deviation calculated for each set.

C(2)

	1	2	3	4	5	Mean	St. dev.	R/R_0	$\Delta R/R_0$	F	ΔF	KIE_{calc}	ΔKIE_F	ΔKIE_R	ΔKIE
**Expt. 1**	475.48	478.17	472.96	474.93	473.22	474.952	2.097777	0.983859	0.008639	0.039	0.01	1.016737	0.002117	0.001733	0.002736
**Expt. 2**	467.76	476.86	473.38	475.34	479.04	474.476	4.288529	0.982873	0.0116	0.038	0.01	1.017767	0.002256	0.001917	0.002961
**Expt. 3**	479.88	475.76	475.28	472.82	476.85	476.118	2.568836	0.986274	0.009184	0.036	0.01	1.014175	0.002144	0.001641	0.0027

C(3)H

	1	2	3	4	5	Mean	St. dev.	R/R_0	$\Delta R/R_0$	F	ΔF	KIE_{calc}	ΔKIE_F	ΔKIE_R	ΔKIE
**Expt. 1**	484.58	479.49	482.98	482.04	488.12	483.442	3.200628	0.997215	0.00876	0.039	0.01	1.002849	0.002123	0.001743	0.002747
**Expt. 2**	486.62	480.23	483.08	481.32	485.91	483.432	2.79021	0.997195	0.00814	0.038	0.01	1.002868	0.002091	0.001647	0.002662
**Expt. 3**	484.73	482.25	485.67	483.04	485.21	484.18	1.46697	0.998738	0.006512	0.036	0.01	1.001287	0.001998	0.001426	0.002455

Where, R/R_0 **= mean of the sample/mean of the standard**

$\Delta(R/R_0) = R/R_0 \times [(\text{st. dev. of sample/mean of sample})^2 + (\text{st. dev. of standard/mean of standard})^2]^{0.5}$

F = reaction conversion as a fraction of 1

ΔF = estimated error in conversion

$$KIE_{calc} = \frac{\ln(1-F)}{\ln[(1-F)R/R_0]}$$

$$\Delta KIE_F = \frac{-\ln(R/R_0)}{(1-F)\ln^2[(1-F)R/R_0]} \times \Delta F$$

$$\Delta KIE_R = \frac{-\ln(1-F)}{(R/R_0)\ln^2[(1-F)R/R_0]} \times \Delta(R/R_0)$$

$$\Delta KIE = KIE_{calc} \times [(\Delta KIE_R/KIE_{calc})^2 + (\Delta KIE_F/KIE_{calc})^2]^{0.5}$$

X-Ray Crystallographic Details

Data were recorded at 93 K on a Rigaku XtaLAB P200 diffractometer using multi-layer mirror monochromated Mo-Kσ radiation.

(3*S*,4*S*)-**47**	
**CCDC**	1886142
**empirical formula**	$C_{19}H_{15}BrF_5NO_2$
**fw**	464.23
**crystal description**	colourless prism
**space group**	$P2_1$ (No. 4)
a **[Å]**	11.753(6)
b **[Å]**	6.281(3)
c **[Å]**	13.273(6)
β **[°]**	101.939(9)
**vol [Å]^3**	958.6(8)
Z	2
**reflns collected**	10431
**independent reflns (*R*$_{int}$)**	3443 (0.0639)
R$_1$ **[*I* > 2*σ*(*I*)]**	0.0343
wR$_2$ **(all data)**	0.0626
**Flack parameter**	−0.016(9)

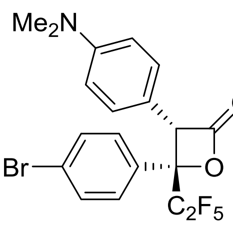

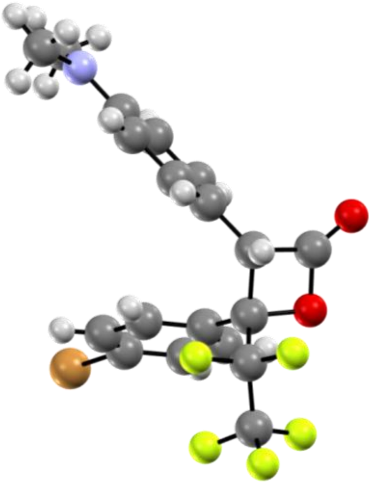

(3*S*,4*S*)-**53**	
**CCDC**	1886144
**empirical formula**	$C_{18}H_{15}BrF_3NO_2$
**fw**	414.22
**crystal description**	colourless prism
**space group**	$P2_12_12_1$ (No. 19)
a **[Å]**	9.2685(13)
b **[Å]**	17.886(3)
c **[Å]**	21.589(3)
**vol [Å]^3**	3578.9(9)
Z	8
**reflns collected**	20510
**independent reflns (*R*$_{int}$)**	6593 (0.0536)
R$_1$ **[*I* > 2*σ*(*I*)]**	0.0400
wR$_2$ **(all data)**	0.0704
**Flack parameter**	−0.003(6)

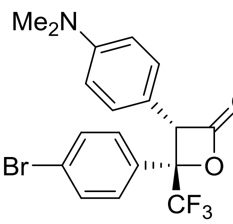

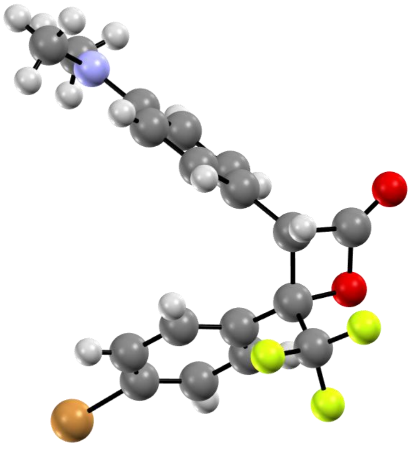

(2*S*,3*S*)-**60**	
**CCDC**	1886143
**empirical formula**	$C_{26}H_{19}BrF_9NO_2$
**fw**	628.33
**crystal description**	colourless prism
**space group**	$P2_12_12_1$ (No. 19)
a **[Å]**	13.6483(15)
b **[Å]**	16.5846(18)
c **[Å]**	23.807(3)
**vol [Å]^3**	5388.8(11)
Z	8
**reflns collected**	23697
**independent reflns (*R*$_{int}$)**	9651 (0.0410)
R$_1$ **[*I* > 2*σ*(*I*)]**	0.0404
wR$_2$ **(all data)**	0.0784
**Flack parameter**	−0.007(5)

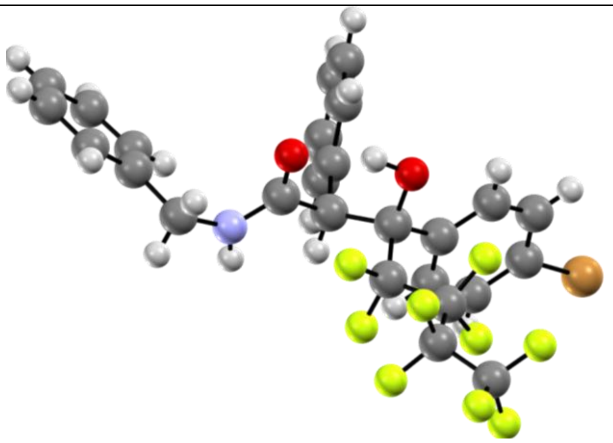

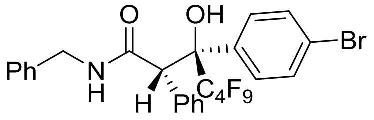

Computational Details

i) Complete Authorship of Gaussian 09

Gaussian 09, Revision **D.01**, M. J. Frisch, G. W. Trucks, H. B. Schlegel, G. E. Scuseria, M. A. Robb, J. R. Cheeseman, G. Scalmani, V. Barone, G. A. Petersson, H. Nakatsuji, X. Li, M. Caricato, A. Marenich, J. Bloino, B. G. Janesko, R. Gomperts, B. Mennucci, H. P. Hratchian, J. V. Ortiz, A. F. Izmaylov, J. L. Sonnenberg, D. Williams-Young, F. Ding, F. Lipparini, F. Egidi, J. Goings, B. Peng, A. Petrone, T. Henderson, D. Ranasinghe, V. G. Zakrzewski, J. Gao, N. Rega, G. Zheng, W. Liang, M. Hada, M. Ehara, K. Toyota, R. Fukuda, J. Hasegawa, M. Ishida, T. Nakajima, Y. Honda, O. Kitao, H. Nakai, T. Vreven, K. Throssell, J. A. Montgomery, Jr., J. E. Peralta, F. Ogliaro, M. Bearpark, J. J. Heyd, E. Brothers, K. N. Kudin, V. N. Staroverov, T. Keith, R. Kobayashi, J. Normand, K. Raghavachari, A. Rendell, J. C. Burant, S. S. Iyengar, J. Tomasi, M. Cossi, J. M. Millam, M. Klene, C. Adamo, R. Cammi, J. W. Ochterski, R. L. Martin, K. Morokuma, O. Farkas, J. B. Foresman, and D. J. Fox, Gaussian, Inc., Wallingford CT, 2016.

ii) General Computational Procedure

The following section describes the methodology for identifying the nature of the potential energy surface for the lactonisation step reported in the manuscript (Section 2.3.1). Manual, exhaustive conformation searches were performed to locate all relevant structures. All conformers were optimized using the Gaussian 09 computational package (see above reference). The stepwise aldol/lactonisation process was located with the M06-2X[5]/6-31G(d)[6] level of theory with implicit solvent modelled using PCM (Polarizable Continuum Model)[7] for dichloromethane. The concerted [2+2] process was located with the PBE[8]/6-31G(d) with implicit solvent modelled using PCM for dichloromethane. Minima were confirmed at both levels of theory with vibrational frequency calculations, with ground states not having imaginary frequencies and transition states (TSs) having one imaginary vibrational frequency. The potential energy surfaces were generated for both levels of theory by fixing the distance between the nucleophilic carbon of the HBTM-enolate complex and the electrophilic carbon of the ketone (CC bond) and varying distances corresponding to the second forming σ-bond (CO bond).

The following section describes the methodology for the computed catalytic cycle reported in the manuscript (Section 2.3.3). Manual, exhaustive conformation searches were performed to locate all relevant structures. All conformers were optimized using the Gaussian 09 computational package (see above reference) using PBE/6-31G(d) with implicit solvent modelled using PCM (Polarizable Continuum

Model) for dichloromethane. Minima were confirmed with vibrational frequency calculations computed at –90 °C, with transition states having one imaginary vibrational frequency. Single point electronic energies were obtained at the PBE/6-311++G(2df,p) level of theory with implicit solvent modelled as before. Final free energies were calculated by adding the single point energies and the thermal correction to Gibbs free energy obtained from vibration frequency calculations. All 3D structure images were rendered in CYLview visualization software.[9]

iii) Temperature Effects on Rate-Limiting Step

Initial reaction opimisation conditions were at room temperature (Table 1, entry 8). To match experiments, we initially computed the reaction coordinate diagram at 25 °C (Figure S1). Under these conditions, the concerted [2+2] **TS-E** is the rate-limiting step (RLS) for the concerted PBE mechanism while the C–C bond forming **TS-I** is rate-limiting for the stepwise M06-2X mechanism. After reaction optimisation for kinetic isotope effect (KIE) experiments (Scheme 5), we recomputed the reaction coordinate diagrams at –90 °C to match experimental conditions. Even at the lower temperature, **TS-E** is the RLS for the concerted PBE mechanism. However, deprotonation **TS-C** is the RLS for the stepwise M06-2X mechanism. The RLSs at –90 °C were used in the KIE calculations.

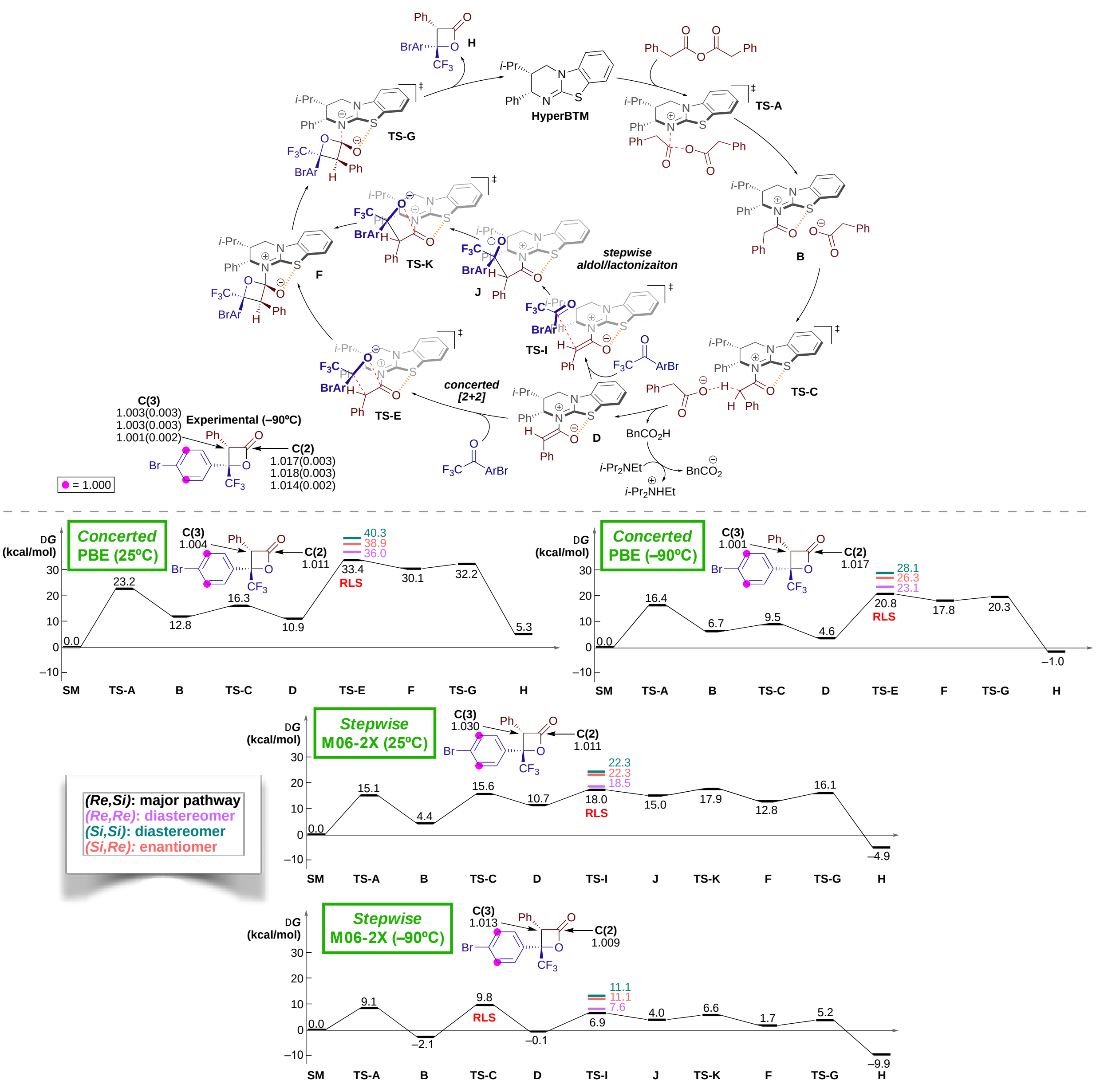

Figure S1. Rate-limiting steps (RLSs) for concerted and stepwise mechanisms at 25 °C and –90 °C. The concerted [2+2] **TS-E** is rate-limiting at both temperatures for the concerted mechanism. The C–C bond forming **TS-I** is rate-limiting for the stepwise mechanism at 25 °C. However, the deprotonation **TS-C** is rate-limiting for the stepwise mechanism at –90 °C and acylated-HBTM phenylacetate complex **B** is the most stable intermediate (MSI). All other MSIs are staring materials (**SM**).

iv) Alternative Computational Method and Mechanisms

The following results are at the same level of theory as the computed catalytic cycle (see Part II for details). The alternative mechanisms are summarized in Figure S2. Alternative 1 features an *in situ*

generated ketene followed by a concerted [2+2] β-lactone formation. This mechanism is not competitive with the mechanism proposed in the manuscript ($\Delta G^‡$ = 30.7 vs 20.8 kcal/mol). Alternative 2 utilizes *N*,*N*-diisopropylethylamine as a catalyst for the concerted [2+2] β-lactone formation. This mechanism is also inaccessible ($\Delta G^‡$ = 42.0 vs 20.8 kcal/mol). The final alternative mechanism features catalyst addition to the ketone followed by a stepwise C–O bond formation and finally by a C–C bond formation. The analogous C–C followed by C–O bond formation process could not be located. Similar to the other alternative mechanisms, this pathway is higher in energy than the mechanism proposed in the manuscript ($\Delta G^‡$ = 43.2 vs 20.8 kcal/mol).

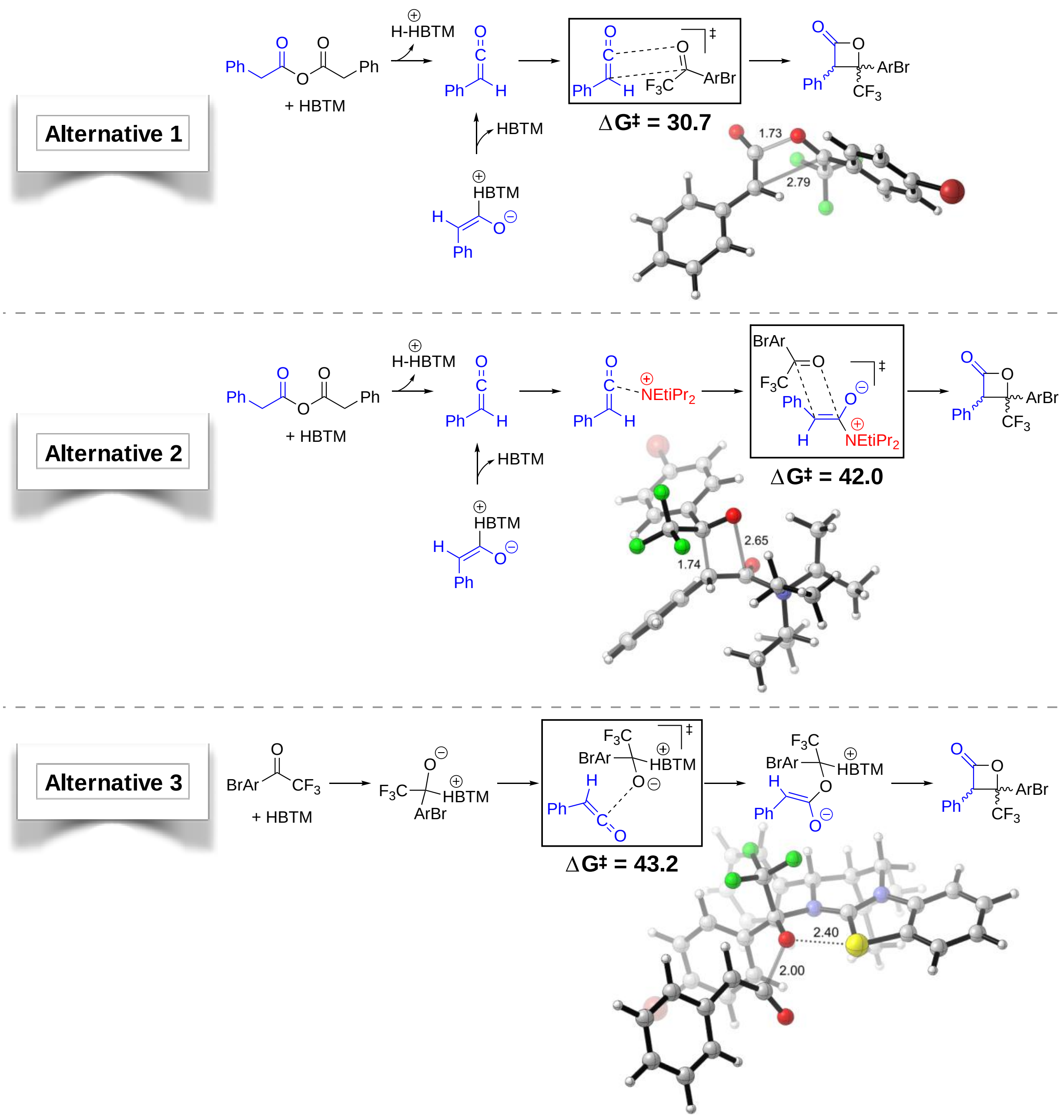

Figure S2. Computed alternative mechanisms. Alternative 1: *in situ* generated ketene, followed by concerted [2+2] process. Alternative 2: *N*,*N*-diisopropylethylamine as a catalyst. Alternative 3: HBTM catalyst addition to the ketone followed by C–O bond formation. All alternative mechanisms are inaccessibly high in energy compared to the mechanism proposed in the manuscript ($\Delta G^{\ddagger}$ = 20.8). Energies are in kcal/mol and distances are in Ångströms.

We also tried B3LYP as an alternative DFT method (Figure S3). B3LYP predicted a stepwise mechanism similar to M06-2X, but also predicted the incorrect lactone product stereoisomer (TS-

(*Re*,*Re*)). Predicted selectivity was still over experimental values. Additionally, computed KIE values do not match experimental KIE results.

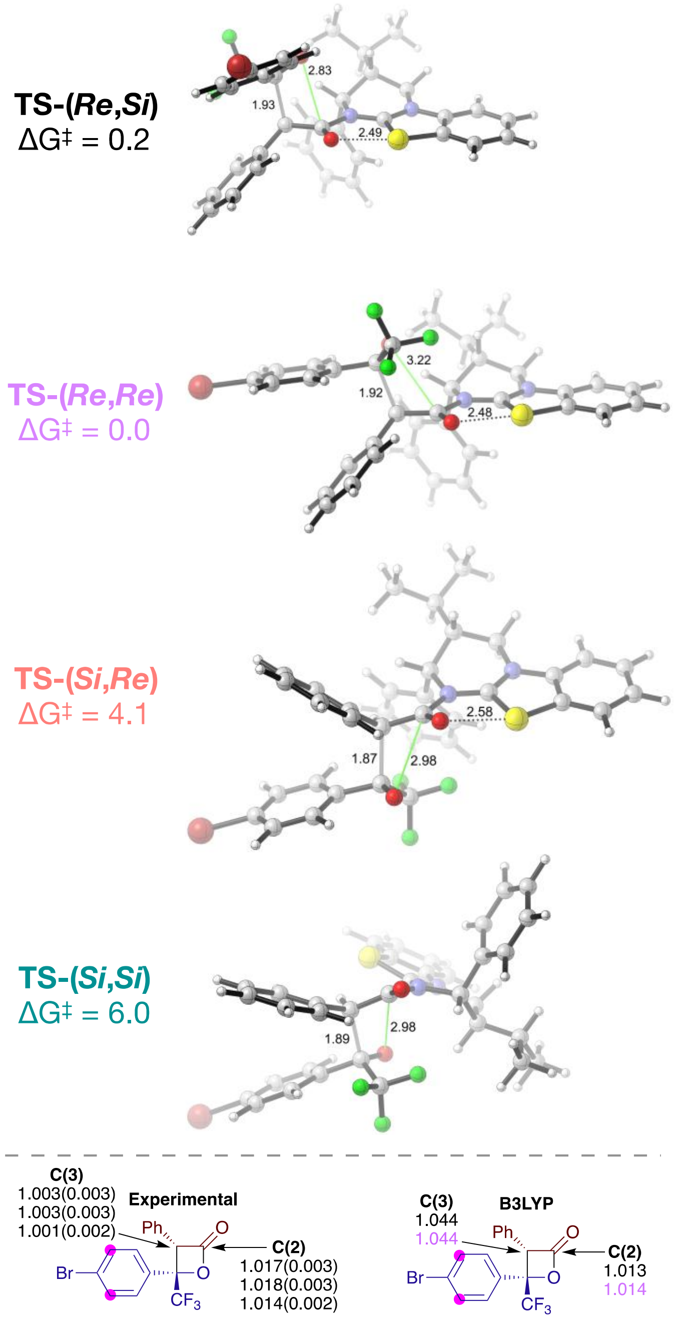

Figure S3. Selectivity and KIE results for B3LYP/6-311++G(2df,p)/PCM(DCM)/B3LYP/6-31G(d)/PCM(DCM). KIE values for TS-(*Re*,*Si*) are in black and TS-(*Re*,*Re*) are in purple.

v) Computed Kinetic Isotope Effects

Each computed KIE was adjusted to accommodate the *meta*-carbons of the 4′-bromo-2,2,2-trifluoroacetophenone substituent used as an internal standard by experimentalists (Figure S4). The average of all four labeling experiments determined the adjustment for subsequent computed KIE values. The concerted PBE mechanism KIE values were adjusted by 0.005. The rate-limiting step for the stepwise M06-2X mechanism does not involve the 4′-bromo-2,2,2-trifluoroacetophenone substituent, therefore the subsequent KIE values were not adjusted.

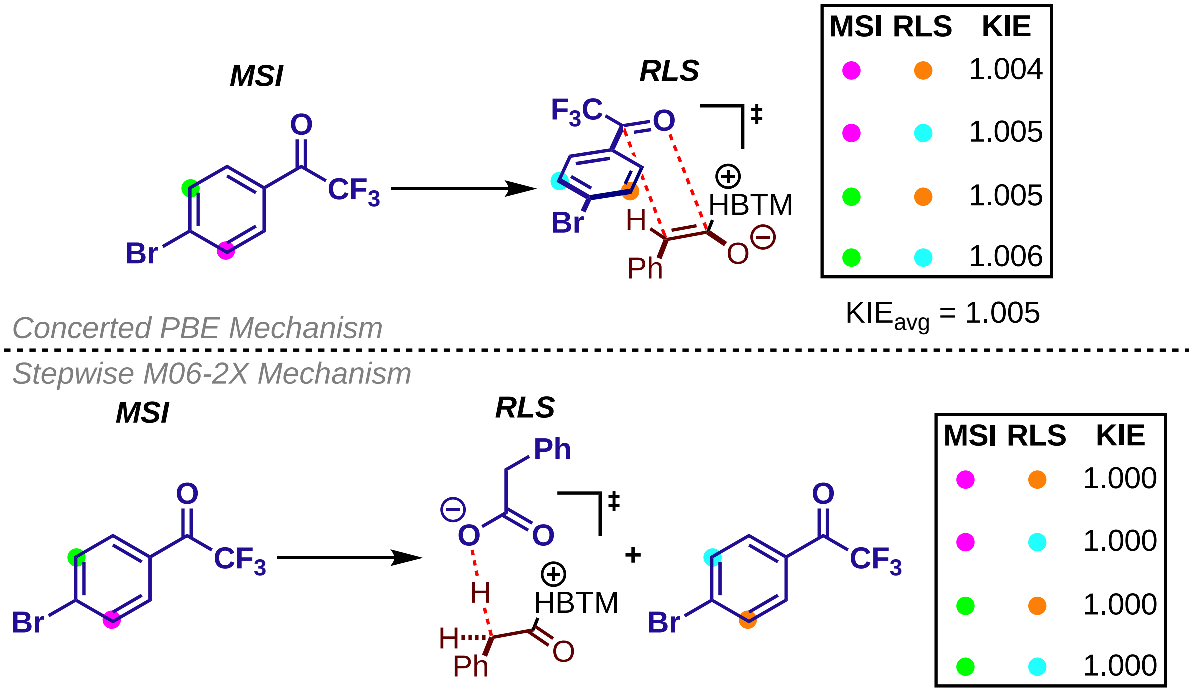

Figure S4. Computed kinetic isotope effects (KIE) for *meta*-carbons of the 4′-bromo-2,2,2-trifluoroacetophenone substituent used as an internal standard. Subsequent concerted PBE mechanism KIEs are adjusted by 0.005. The rate-limiting step (RLS) for the stepwise M06-2X mechanism does not involve the 4′-bromo-2,2,2-trifluoroacetophenone, therefore the M06-2X KIEs are not adjusted.

Due to the symmetric characteristic of the phenylacetic anhydride, the reported KIE values in the manuscript are averages from all labeling experiments (Figure S5).

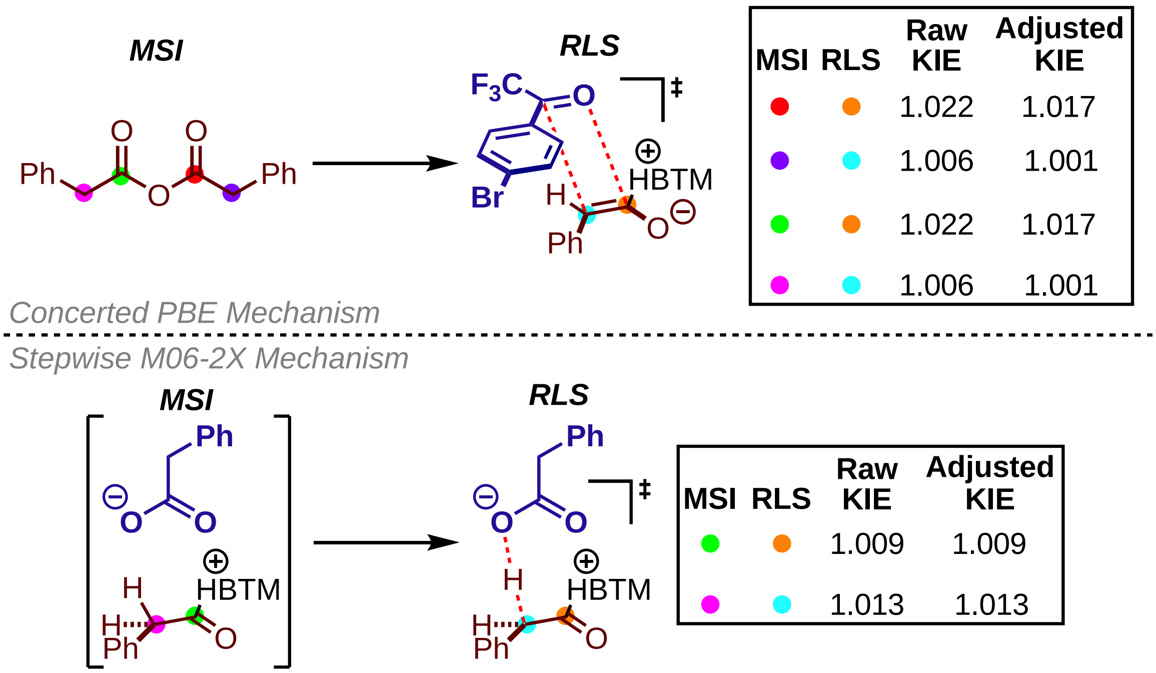

Figure S5. Computed KIE experiments. Concerted PBE mechanism KIE values are adjusted by 0.005 and the stepwise M06-2X mechanism values are not adjusted (*vide supra*). Note that the most stable intermediate (MSI) for the M06-2X mechanism is the acylated-HBTM phenylacetate complex **B**.

Computed Geometries and Energies

Concerted [2+2] Mechanism Coordinate Diagram: PBE

SM: Phenylacetic anhydride

Using Gaussian 09: AM64L-G09RevD.01 24-Apr-2013
==
==============================
# pbepbe/6-31G*/auto gfprint gfinput scf=(direct,tight,maxcycle=300,xqc)
SCRF=(PCM,SOLVENT=Dichloromethane) opt=(maxcycle=250) freq=noraman
iop(1/8=18) Temperature=298.15
#N Geom=AllCheck Guess=TCheck SCRF=Check Test GenChk RPBEPBE/6-31G(d)/Auto
Freq

Pointgroup= C1 Stoichiometry= C16H14O3 C1[X(C16H14O3)] #Atoms= 33
Charge = 0 Multiplicity = 1

SCF Energy= -842.819202108 Predicted Change= -4.533125D-09
==
==============================
Optimization completed. {Found 1 times}
Item Max Val. Criteria Pass? RMS Val. Criteria Pass?
Force 0.00002 || 0.00045 [YES] 0.00000 || 0.00030 [YES]
Displ 0.00362 || 0.00180 [NO] 0.00362 || 0.00180 [YES]

Atomic Type	Coordinates (Angstroms) X	Y	Z
C	1.215945	-0.165702	0.129526
C	2.352661	0.157898	1.089490
C	3.726433	0.079099	0.462715
O	0.000004	-0.000086	0.816311
C	-1.215943	0.165947	0.129642
C	-2.352647	-0.158234	1.089423
C	-3.726421	-0.079314	0.462666
O	-1.300508	0.559506	-1.011167
O	1.300498	-0.558603	-1.011510
C	4.474324	1.248754	0.237949
C	5.751887	1.182363	-0.338243
C	6.295699	-0.058878	-0.699176
C	5.556227	-1.232108	-0.480275
C	4.281369	-1.162611	0.097244
C	-4.281415	1.162466	0.097521
C	-5.556286	1.232055	-0.479957
C	-6.295715	0.058851	-0.699144
C	-5.751843	-1.182460	-0.338541
C	-4.474267	-1.248943	0.237611
H	2.255837	-0.542202	1.942411
H	2.158959	1.160629	1.511199
H	-2.255923	0.541495	1.942661
H	-2.158841	-1.161129	1.510689
H	4.052621	2.220869	0.519056
H	6.322112	2.102447	-0.503760
H	7.293174	-0.113403	-1.147792
H	5.974684	-2.205224	-0.758059
H	3.709079	-2.081626	0.267176
H	-3.709160	2.081462	0.267671
H	-5.974787	2.205224	-0.757487
H	-7.293200	0.113449	-1.147728
H	-6.322031	-2.102526	-0.504284
H	-4.052518	-2.221113	0.518457

Statistical Thermodynamic Analysis
Temperature= 298.150 Kelvin Pressure= 1.00000 Atm
==
==============================
SCF Energy= -842.819202108 Predicted Change= -4.533125D-09
Zero-point correction (ZPE)= -842.5646 0.25454
Internal Energy (U)= -842.5473 0.27182
Enthalpy (H)= -842.5464 0.27277
Gibbs Free Energy (G)= -842.6164 0.20276

Frequencies -- 5.9035 17.8941 20.6541

183.15K thermal correction = 0.227392
Single point SCF = -843.073949

SM: HyperBTM

Using Gaussian 09: AM64L-G09RevD.01 24-Apr-2013
==
==============================
# pbepbe/6-31G*/auto gfprint gfinput scf=(direct,tight,maxcycle=300,xqc)
opt=(maxcycle=250) freq=noraman iop(1/8=18) Temperature=298.15
SCRF=(PCM,SOLVENT=Dichloromethane)
#N Geom=AllCheck Guess=TCheck SCRF=Check Test GenChk RPBEPBE/6-31G(d)/Auto
Freq

Pointgroup= C1 Stoichiometry= C19H20N2S C1[X(C19H20N2S)] #Atoms= 42
Charge = 0 Multiplicity = 1

SCF Energy= -1242.52496543 Predicted Change= -9.655754D-09
==
==============================
Optimization completed. {Found 2 times}
Item Max Val. Criteria Pass? RMS Val. Criteria Pass?
Force 0.00001 || 0.00045 [YES] 0.00000 || 0.00030 [YES]
Displ 0.00122 || 0.00180 [YES] 0.00122 || 0.00180 [YES]

Atomic Type	Coordinates (Angstroms) X	Y	Z
C	4.381114	0.809093	-1.701735
H	4.827274	1.359136	-2.536175
C	3.011755	0.970293	-1.433696
H	2.396988	1.629017	-2.053456
C	2.449137	0.261868	-0.360611
C	3.253890	-0.605412	0.419881
C	4.614839	-0.763170	0.147812
H	5.230394	-1.432447	0.756563
C	5.177231	-0.045163	-0.922654
H	6.242109	-0.159247	-1.146312

N	1.121492	0.293993	0.064065
C	0.809679	-0.531121	1.144101
S	2.297198	-1.383339	1.690725
C	0.079439	1.151118	-0.508676
H	-0.332350	0.684773	-1.425555
H	0.552130	2.103752	-0.796211
C	-1.024895	1.395000	0.534998
C	-2.187050	2.263089	-0.019095
C	-3.115425	2.737213	1.115566
H	-2.563423	3.375238	1.830178
H	-3.556259	1.902116	1.684413
H	-3.948285	3.335128	0.706517
H	-2.779081	1.634870	-0.713044
C	-1.685067	3.489944	-0.806200
H	-1.016912	4.116475	-0.186273
H	-2.539755	4.117856	-1.112111
H	-1.140114	3.211589	-1.724224
H	-0.556098	1.959115	1.367488
C	-1.470280	0.027594	1.159838
C	-2.283099	-0.859404	0.211511
H	-2.124838	0.260588	2.019271
N	-0.327179	-0.719191	1.717471
C	-1.663838	-1.689232	-0.745527
H	-0.571452	-1.740081	-0.799280
C	-2.427289	-2.478928	-1.617793
H	-1.922788	-3.116583	-2.351683
C	-3.828197	-2.463988	-1.543828
H	-4.423754	-3.085108	-2.221104
C	-4.458297	-1.657527	-0.584762
H	-5.550580	-1.648121	-0.504564
C	-3.690868	-0.868466	0.284682
H	-4.195230	-0.255053	1.040336

Statistical Thermodynamic Analysis
Temperature= 298.150 Kelvin Pressure= 1.00000 Atm
==
==============================
SCF Energy= -1242.52496543 Predicted Change= -9.655754D-09
Zero-point correction (ZPE)= -1242.1836 0.34127
Internal Energy (U)= -1242.1642 0.36070
Enthalpy (H)= -1242.1633 0.36164
Gibbs Free Energy (G)= -1242.2319 0.29305

Frequencies -- 27.3394 33.3733 58.4929

183.15K thermal correction = 0.316672
Single point SCF = -1242.790297

SM: *N,N*-diisopropylethylamine

Using Gaussian 09: AM64L-G09RevD.01 24-Apr-2013
==
==============================
# pbepbe/6-31G*/auto gfprint gfinput scf=(direct,tight,maxcycle=300,xqc)
opt=(maxcycle=250) freq=noraman iop(1/8=18) Temperature=298.15
SCRF=(PCM,SOLVENT=Dichloromethane)
#N Geom=AllCheck Guess=TCheck SCRF=Check Test GenChk RPBEPBE/6-31G(d)/Auto
Freq

Pointgroup= C1 Stoichiometry= C8H19N C1[X(C8H19N)] #Atoms= 28
Charge = 0 Multiplicity = 1

SCF Energy= -370.514749575 Predicted Change= -4.774460D-08
==
==============================
Optimization completed. {Found 1 times}

Item	Max Val.	Criteria	Pass?	RMS Val.	Criteria	Pass?
Force	0.00003 ||	0.00045	[YES]	0.00000 ||	0.00030	[YES]
Displ	0.00273 ||	0.00180	[NO]	0.00273 ||	0.00180	[YES]

Atomic Type	Coordinates (Angstroms) X	Y	Z
N	0.002999	0.271176	-0.203343
C	1.048808	-0.779246	-0.198624
C	2.159155	-0.414477	-1.202619
C	1.666903	-1.130092	1.176124
C	-1.396839	-0.191938	-0.074762
C	-1.717617	-1.045100	1.174157
C	-1.867208	-0.900165	-1.357770
C	0.305972	1.461561	0.600518
C	-0.238754	2.755788	-0.020836
H	1.733072	-0.242276	-2.204783
H	2.910385	-1.221591	-1.270636
H	2.689035	0.505794	-0.895230
H	0.557913	-1.694415	-0.576625
H	2.357630	-1.985515	1.070012
H	0.899667	-1.405480	1.917339
H	2.251543	-0.287752	1.586514
H	-1.995460	0.734185	0.008682
H	-2.806747	-1.210647	1.255276
H	-1.386037	-0.546374	2.101751
H	-1.234689	-2.036855	1.122375
H	-1.676711	-0.267148	-2.240407
H	-2.949396	-1.113364	-1.303879
H	-1.352229	-1.865877	-1.510224
H	1.403509	1.553721	0.670786
H	-0.058815	1.373219	1.651147
H	0.168224	2.895274	-1.036735
H	0.045831	3.626773	0.596042
H	-1.340243	2.752115	-0.095128

Statistical Thermodynamic Analysis
Temperature= 298.150 Kelvin Pressure= 1.00000 Atm
==
==============================
SCF Energy= -370.514749575 Predicted Change= -4.774460D-08
Zero-point correction (ZPE)= -370.2589 0.25584
Internal Energy (U)= -370.2464 0.26829
Enthalpy (H)= -370.2455 0.26924
Gibbs Free Energy (G)= -370.2961 0.21859

Frequencies -- 62.3975 66.5372 104.7307

183.15K thermal correction = 0.236373
Single point SCF = -370.620211

SM: 4′-bromo-2,2,2-trifluoroacetophenone

Using Gaussian 09: AM64L-G09RevD.01 24-Apr-2013
==
==============================
pbepbe/6-31G*/auto gfprint gfinput scf=(direct,tight,maxcycle=300,xqc)

SCRF=(PCM,SOLVENT=Dichloromethane)
opt=(maxcycle=250) freq=noraman
iop(1/8=18) Temperature=298.15
#N Geom=AllCheck Guess=TCheck SCRF=Check Test GenChk RPBEPBE/6-31G(d)/Auto
Freq

Pointgroup= C1 Stoichiometry= C8H4BrF3O
C1[X(C8H4BrF3O)] #Atoms= 17
Charge = 0 Multiplicity = 1

SCF Energy= -3252.58908828 Predicted Change= -1.230780D-08
===
Optimization completed. {Found 2 times}

Item	Max Val.		Criteria	Pass?	RMS Val.		Criteria	Pass?
Force	0.00006	||	0.00045	[YES]	0.00001	||	0.00030	[YES]
Displ	0.00028	||	0.00180	[YES]	0.00028	||	0.00180	[YES]

Atomic Type	Coordinates (Angstroms) X	Y	Z
C	-0.861364	0.482695	0.000021
C	-2.312383	0.784777	0.000038
O	-2.776140	1.922494	0.000056
C	-3.319148	-0.408576	0.000013
F	-4.587703	0.036236	-0.000033
F	-3.141342	-1.192189	1.100070
F	-3.141265	-1.192203	-1.100021
C	0.028335	1.581279	0.000053
C	1.408316	1.383907	0.000038
C	1.903737	0.069469	-0.000010
Br	3.786974	-0.213339	-0.000031
C	1.045014	-1.039345	-0.000045
C	-0.336769	-0.829913	-0.000029
H	-0.387372	2.593085	0.000089
H	2.095923	2.233142	0.000064
H	1.452831	-2.052950	-0.000084
H	-0.997982	-1.698721	-0.000057

Statistical Thermodynamic Analysis
Temperature= 298.150 Kelvin Pressure= 1.00000 Atm
===

SCF Energy=	-3252.58908828	Predicted Change= -1.230780D-08
Zero-point correction (ZPE)=	-3252.4871	0.10198
Internal Energy (U)=	-3252.4755	0.11351
Enthalpy (H)=	-3252.4746	0.11445
Gibbs Free Energy (G)=	-3252.5271	0.06191

Frequencies -- 34.2491 53.3532 78.1002

183.15K thermal correction = 0.080579
Single point SCF = -3255.247932

TS-A: HBTM addition to phenylacetic anhydride (Re-face)

Using Gaussian 09: AM64L-G09RevD.01 24-Apr-2013
===
# pbepbe/6-31G(d)/auto gfprint gfinput
scf=(direct,tight,maxcycle=300,xqc)
opt=(verytight,maxcycle=250,calcfc,ts,noeigentest) freq=noraman
SCRF=(PCM,SOLVENT=Dichloromethane) iop(1/8=12)
Temperature=298.15
#N Geom=AllCheck Guess=TCheck SCRF=Check Test GenChk RPBEPBE/6-31G(d)/Auto
Freq

Pointgroup= C1 Stoichiometry= C35H34N2O3S
C1[X(C35H34N2O3S)] #Atoms= 75
Charge = 0 Multiplicity = 1

SCF Energy= -2085.34362314 Predicted Change= -5.071033D-13
===
Optimization completed. {Found 1 times}

Item	Max Val.		Criteria	Pass?	RMS Val.		Criteria	Pass?
Force	0.00000	||	0.00000	[YES]	0.00000	||	0.00000	[YES]
Displ	0.00002	||	0.00000	[NO]	0.00002	||	0.00000	[YES]

Atomic Type	Coordinates (Angstroms) X	Y	Z
C	-6.019057	-0.577845	-1.899186
C	-5.088337	-0.306383	-0.884469
C	-3.738736	-0.598772	-1.129459
C	-3.330752	-1.159455	-2.357723
C	-4.261947	-1.423603	-3.367243
C	-5.614079	-1.129521	-3.126742
N	-2.661657	-0.401709	-0.257293
C	-1.438681	-0.821778	-0.733171
S	-1.577101	-1.417461	-2.411126
C	-2.777948	0.276226	1.041066
C	-1.412079	0.833312	1.464006
C	-1.454294	1.526111	2.852665
C	-0.147333	2.293206	3.130822
C	-2.646059	2.496052	2.981869
C	-0.343343	-0.301227	1.334625
C	-0.488877	-1.432966	2.355573
N	-0.330135	-0.816232	-0.044639
C	-1.369037	-2.515819	2.155097
C	-1.498215	-3.524995	3.120616
C	-0.743285	-3.476061	4.301727
C	0.149085	-2.413731	4.506525
C	0.276346	-1.405954	3.539565
C	1.318695	-1.056939	-0.998031
C	1.965178	-2.144604	-0.123522
C	3.392651	-2.431828	-0.551246
O	1.985274	0.227038	-0.584082
C	1.554019	1.415137	-1.089980
C	2.629604	2.489183	-0.875158
C	2.066772	3.873365	-0.635534
O	0.480429	1.611509	-1.640400
O	1.060574	-1.228979	-2.193041
C	4.477636	-2.037493	0.254189
C	5.798459	-2.302411	-0.137607
C	6.053524	-2.963834	-1.348218
C	4.979344	-3.356133	-2.162972
C	3.660725	-3.092085	-1.767347
C	1.420526	4.582875	-1.666978
C	0.891463	5.859845	-1.433297
C	1.000358	6.448965	-0.163620
C	1.643260	5.752446	0.870299
C	2.171339	4.473705	0.633511
H	-7.076243	-0.356050	-1.724368
H	-5.411262	0.115751	0.071245
H	-3.943112	-1.851712	-4.322567

H	-6.355520	-1.335145	-3.904583
H	-3.179381	-0.428594	1.794574
H	-3.504498	1.093645	0.914219
H	0.008987	3.089592	2.380154
H	0.743677	1.643268	3.117717
H	-0.189824	2.771271	4.124667
H	-1.569867	0.740336	3.624425
H	-2.632898	3.252645	2.175882
H	-2.590890	3.033431	3.944153
H	-3.620175	1.979106	2.953351
H	-1.123094	1.595931	0.713554
H	0.649095	0.158512	1.480981
H	-1.946901	-2.591157	1.228184
H	-2.186416	-4.357890	2.942271
H	-0.841201	-4.267171	5.052309
H	0.756818	-2.371744	5.416468
H	0.988360	-0.589116	3.704400
H	1.332593	-3.040629	-0.244194
H	1.936318	-1.854968	0.938837
H	3.248975	2.476707	-1.794160
H	3.292146	2.176906	-0.051750
H	4.283309	-1.519857	1.200979
H	6.629261	-1.991954	0.505289
H	7.083608	-3.174236	-1.655163
H	5.169054	-3.872544	-3.110250
H	2.823925	-3.390179	-2.407917
H	1.330425	4.126475	-2.658022
H	0.394504	6.398549	-2.247272
H	0.590003	7.448151	0.017130
H	1.738090	6.204498	1.863415
H	2.676539	3.934663	1.443844

Statistical Thermodynamic Analysis
Temperature= 298.150 Kelvin   Pressure= 1.00000 Atm
==
============================
SCF Energy=   -2085.34362314   Predicted Change= -5.071033D-13
Zero-point correction (ZPE)=   -2084.7454   0.59817
Internal Energy (U)=   -2084.7084   0.63513
Enthalpy (H)=   -2084.7075   0.63607
Gibbs Free Energy (G)=   -2084.8181   0.52549

Frequencies --   -122.4556   16.2913   19.2429

183.15K thermal correction = 0.562898
Single point SCF = -2085.856939

B: Acylated-HBTM phenylacetate complex

Using Gaussian 09: AM64L-G09RevD.01 24-Apr-2013
==
============================
# pbepbe/6-31G*/auto gfprint gfinput
scf=(direct,tight,maxcycle=300,xqc)
opt=(maxcycle=250) freq=noraman iop(1/8=18)
Temperature=298.15
SCRF=(PCM,SOLVENT=Dichloromethane)
#N Geom=AllCheck Guess=TCheck SCRF=Check Test GenChk
RPBEPBE/6-31G(d)/Auto
Freq

Pointgroup= C1   Stoichiometry= C35H34N2O3S
C1[X(C35H34N2O3S)] #Atoms= 75
Charge = 0   Multiplicity = 1

SCF Energy= -2085.35330388   Predicted Change= -3.255853D-06
==
============================
Optimization completed.   {Found   1   times}

Item	Max Val.	Criteria	Pass?	RMS Val.	Criteria	Pass?
Force	0.00013 ||	0.00045	[YES]	0.00001 ||	0.00030	[YES]
Displ	0.14330 ||	0.00180	[NO]	0.14330 ||	0.00180	[NO]

Atomic Type	Coordinates (Angstroms) X	Y	Z
C	6.269836	2.288570	-1.569102
C	5.421046	1.190692	-1.385727
C	4.158344	1.432181	-0.822926
C	3.761423	2.729128	-0.446370
C	4.613940	3.824381	-0.635410
C	5.873338	3.589610	-1.201658
N	3.156342	0.483011	-0.557510
C	2.046322	0.997204	0.022317
S	2.124551	2.735166	0.237774
C	3.263535	-0.932961	-0.952101
C	1.863404	-1.519287	-1.130822
C	1.895459	-3.019735	-1.543797
C	0.533349	-3.469756	-2.105486
C	2.995072	-3.323497	-2.581228
C	1.016734	-1.253998	0.148341
C	1.437916	-2.032880	1.388372
N	1.005651	0.221738	0.415424
C	2.490664	-1.622327	2.231524
C	2.860273	-2.395611	3.342154
C	2.182897	-3.589534	3.629254
C	1.124878	-4.001458	2.805279
C	0.752634	-3.226141	1.698462
C	-0.130630	0.863461	1.009063
C	-1.203174	-0.027595	1.589562
C	-2.357026	0.747803	2.189951
O	-1.027580	0.195391	-1.714664
C	-1.919700	-0.701368	-1.742086
C	-2.940197	-0.669435	-2.929877
C	-4.306527	-0.201696	-2.480564
O	-2.095994	-1.640236	-0.888807
O	-0.124262	2.092820	1.091450
C	-3.594110	0.783533	1.520056
C	-4.682504	1.480807	2.066042
C	-4.547121	2.149133	3.291866
C	-3.317632	2.115315	3.969738
C	-2.231275	1.419665	3.421214
C	-5.133419	-1.031734	-1.692395
C	-6.385674	-0.586910	-1.245807
C	-6.839775	0.701066	-1.577099
C	-6.026714	1.537766	-2.356886
C	-4.773883	1.088662	-2.802066
H	7.260339	2.127161	-2.004091
H	5.741409	0.183134	-1.662489
H	4.304343	4.833406	-0.347813
H	6.556839	4.429231	-1.357442
H	3.850417	-1.475328	-0.187942
H	3.819718	-0.954081	-1.900246
H	0.352731	-2.996914	-3.088746
H	-0.320190	-3.202000	-1.460987
H	0.529837	-4.563282	-2.255405
H	2.119394	-3.614675	-0.637580
H	2.880735	-2.694541	-3.483349
H	2.918143	-4.376534	-2.901516
H	4.014551	-3.179236	-2.184167
H	1.354600	-0.949780	-1.933068

H	-0.029107	-1.494652	-0.120899
H	3.019315	-0.680710	2.045091
H	3.677667	-2.057634	3.987359
H	2.470991	-4.190180	4.498036
H	0.578016	-4.922968	3.029535
H	-0.087761	-3.540842	1.069938
H	-0.719283	-0.676559	2.346376
H	-1.572680	-0.705564	0.783881
H	-3.008438	-1.695296	-3.336323
H	-2.552046	-0.006282	-3.720593
H	-3.704909	0.250111	0.569262
H	-5.636761	1.496230	1.528239
H	-5.396312	2.691903	3.720978
H	-3.205363	2.630722	4.929791
H	-1.273996	1.394926	3.955037
H	-4.769023	-2.027020	-1.417862
H	-7.012600	-1.249845	-0.638798
H	-7.821233	1.046482	-1.234360
H	-6.368795	2.544308	-2.622354
H	-4.144844	1.748264	-3.411832

Statistical Thermodynamic Analysis
Temperature= 298.150 Kelvin Pressure= 1.00000 Atm
===
==============================
SCF Energy= -2085.35330388 Predicted Change= -3.255853D-06
Zero-point correction (ZPE)= -2084.7541 0.59916
Internal Energy (U)= -2084.7163 0.63691
Enthalpy (H)= -2084.7154 0.63785
Gibbs Free Energy (G)= -2084.8289 0.52439

Frequencies -- 9.7201 16.0633 22.4384

183.15K thermal correction = 0.562852
Single point SCF = -2085.872416

TS-C: Acylated-HBTM deprotonation *via* phenylacetate

Using Gaussian 09: AM64L-G09RevD.01 24-Apr-2013
===
==============================
# pbepbe/6-31G(d)/auto gfprint gfinput scf=(direct,tight,maxcycle=300,xqc)
opt=(maxcycle=250,calcfc,ts,noeigentest) freq=noraman
SCRF=(PCM,SOLVENT=Dichloromethane) iop(1/8=2) Temperature=298.15
#N Geom=AllCheck Guess=TCheck SCRF=Check Test GenChk RPBEPBE/6-31G(d)/Auto
Freq

Pointgroup= C1 Stoichiometry= C35H34N2O3S C1[X(C35H34N2O3S)] #Atoms= 75
Charge = 0 Multiplicity = 1

SCF Energy= -2085.34371516 Predicted Change= -6.492663D-09
===
==============================
Optimization completed. {Found 1 times}

Item	Max Val.	Criteria	Pass?	RMS Val.	Criteria	Pass?
Force	0.00001 ||	0.00045	[YES]	0.00000 ||	0.00030	[YES]
Displ	0.00187 ||	0.00180	[NO]	0.00187 ||	0.00180	[YES]

Atomic Coordinates (Angstroms)

Type	X	Y	Z
C	-5.602253	-2.713314	-1.202116
C	-4.715373	-1.988030	-0.396036
C	-3.355280	-2.011572	-0.739494
C	-2.893027	-2.744150	-1.848080
C	-3.785102	-3.464648	-2.651763
C	-5.145995	-3.441908	-2.317220
N	-2.305210	-1.354061	-0.074711
C	-1.078536	-1.578721	-0.611069
S	-1.131553	-2.593834	-2.044128
C	-2.526026	-0.426876	1.047616
C	-1.359492	0.557209	1.137309
C	-1.511039	1.547934	2.324374
C	-0.466099	2.677935	2.248922
C	-2.919670	2.173822	2.371829
C	-0.020073	-0.235749	1.155068
C	0.246619	-1.054269	2.416267
N	0.053564	-1.090340	-0.066816
C	-0.290964	-2.343511	2.605560
C	-0.049433	-3.052709	3.790957
C	0.737641	-2.488506	4.805323
C	1.290271	-1.212599	4.623090
C	1.049760	-0.505145	3.436857
C	1.324079	-1.361918	-0.742058
C	2.508833	-0.790130	-0.152073
C	3.853268	-1.219341	-0.627181
O	1.239034	-2.010714	-1.804260
C	4.158340	-1.478251	-1.985128
C	5.459166	-1.821807	-2.377856
C	6.495499	-1.907113	-1.434869
C	6.212639	-1.640745	-0.086056
C	4.912057	-1.301898	0.308279
H	-6.667637	-2.712200	-0.953942
H	-5.076602	-1.434400	0.474498
H	-3.427930	-4.031246	-3.516970
H	-5.859789	-3.999671	-2.930468
H	-2.649894	-1.008840	1.979942
H	-3.467135	0.102283	0.838771
H	-0.571596	3.347406	3.119767
H	-0.608939	3.284685	1.337389
H	0.573799	2.310721	2.243746
H	-1.356639	0.984017	3.264709
H	-2.959643	2.938513	3.166119
H	-3.710278	1.436592	2.592170
H	-3.164801	2.673872	1.416703
H	-1.333397	1.145282	0.199629
H	0.779516	0.506209	1.023980
H	-0.884419	-2.819197	1.817464
H	-0.473080	-4.054413	3.915904
H	0.928680	-3.045632	5.728099
H	1.919866	-0.767686	5.400297
H	1.499796	0.484473	3.300076
H	2.465513	-0.716650	0.944132
H	2.428307	0.570089	-0.474184
H	3.362901	-1.416489	-2.731699
H	5.664896	-2.021048	-3.435815
H	7.510489	-2.174948	-1.747258
H	7.007535	-1.700932	0.665839
H	4.703460	-1.097894	1.365849
O	0.412087	1.560606	-1.536794
C	1.426494	2.240580	-1.287292
C	1.510590	3.721905	-1.726603
C	0.340442	4.558449	-1.250637
O	2.504986	1.812312	-0.677376
C	-0.970440	4.306028	-1.707265
C	-2.049902	5.073142	-1.246482
C	-1.839601	6.112624	-0.325347
C	-0.539991	6.376581	0.131778

C	0.538204	5.603845	-0.327363
H	2.462843	4.151558	-1.377622
H	1.538274	3.709809	-2.833422
H	-1.136387	3.488302	-2.414990
H	-3.060465	4.863493	-1.614622
H	-2.682812	6.714998	0.029414
H	-0.362083	7.185899	0.848328
H	1.551524	5.814679	0.034887

Statistical Thermodynamic Analysis
Temperature= 298.150 Kelvin   Pressure= 1.00000 Atm
===
==============================
SCF Energy=   -2085.34371516   Predicted Change= -6.492663D-09
Zero-point correction (ZPE)=   -2084.7489   0.59475
Internal Energy (U)=   -2084.7117   0.63193
Enthalpy (H)=   -2084.7108
0.63288
Gibbs Free Energy (G)=   -2084.8215
0.52221

Frequencies -- -1001.0331   16.4854   24.1148

183.15K thermal correction = 0.559636
Single point SCF = -2085.864613

***N*,*N*-diisopropylethylamine/phenylacetic acid complex**

Using Gaussian 09: AM64L-G09RevD.01 24-Apr-2013
===
==============================
# pbepbe/6-31G*/auto gfprint gfinput
scf=(direct,tight,maxcycle=300,xqc)
opt=(maxcycle=250) freq=noraman iop(1/8=8)
Temperature=298.15
SCRF=(PCM,SOLVENT=Dichloromethane)
#N Geom=AllCheck Guess=TCheck SCRF=Check Test GenChk
RPBEPBE/6-31G(d)/Auto
Freq

Pointgroup= C1   Stoichiometry= C16H27NO2
C1[X(C16H27NO2)] #Atoms= 46
Charge = 0   Multiplicity = 1

SCF Energy= -830.124407497   Predicted Change= -8.407285D-09
===
==============================
Optimization completed.   {Found   1   times}
Item   Max Val.   Criteria   Pass?   RMS Val.   Criteria   Pass?
Force   0.00000 || 0.00045   [YES]   0.00000 || 0.00030   [YES]
Displ   0.01042 || 0.00180   [NO]   0.01042 || 0.00180   [NO]

Atomic Type	Coordinates (Angstroms) X	Y	Z
N	2.641099	-0.120019	-0.106003
C	2.636592	-0.734256	-1.484015
C	2.263319	-2.224866	-1.389869
C	1.675864	-0.025580	-2.451892
C	2.691362	1.387200	-0.115425
C	2.505016	1.945545	1.304253
C	3.943978	1.978921	-0.786077
C	3.730603	-0.718379	0.736600
C	3.216378	-1.521265	1.935555
H	1.234635	-2.347514	-1.009180
H	2.942805	-2.793029	-0.733616
H	2.316936	-2.680321	-2.393077
H	3.665463	-0.664625	-1.899446
H	1.593712	-0.626383	-3.373099
H	2.028833	0.976257	-2.745120
H	0.669681	0.079986	-2.012589
H	1.799383	1.692225	-0.685445
H	3.377369	1.754198	1.953496
H	1.610710	1.519527	1.787675
H	2.372695	3.039314	1.247850
H	4.863847	1.743902	-0.222197
H	4.075318	1.614803	-1.818849
H	3.854838	3.077980	-0.828408
H	4.367827	-1.358688	0.100262
H	4.390905	0.087348	1.098353
H	2.569535	-2.356008	1.619136
H	4.069764	-1.938759	2.497821
H	2.631260	-0.886124	2.621165
C	-3.736765	1.151318	0.369575
C	-4.924282	1.377210	-0.339999
C	-5.693061	0.294059	-0.795358
C	-5.265004	-1.015870	-0.534864
C	-4.074765	-1.238251	0.174481
C	-3.295811	-0.160576	0.635860
C	-2.014399	-0.414443	1.399178
C	-0.737392	-0.002248	0.648779
O	0.299857	-0.771507	0.985069
O	-0.689002	0.952957	-0.131595
H	-3.138558	1.998798	0.719196
H	-5.252566	2.403745	-0.536197
H	-6.622609	0.471157	-1.346878
H	-5.857935	-1.868520	-0.883024
H	-3.744857	-2.264415	0.375950
H	1.212626	-0.451437	0.507915
H	-1.927479	-1.474071	1.689716
H	-2.008474	0.168069	2.342134

Statistical Thermodynamic Analysis
Temperature= 298.150 Kelvin   Pressure= 1.00000 Atm
===
==============================
SCF Energy=   -830.124407497   Predicted Change= -8.407285D-09
Zero-point correction (ZPE)=   -829.7266   0.39772
Internal Energy (U)=   -829.7044   0.41998
Enthalpy (H)=   -829.7034
0.42092
Gibbs Free Energy (G)=   -829.7805
0.34381

Frequencies --   7.1752   16.8437   39.3467

183.15K thermal correction = 0.370473
Single point SCF = -830.373152

D: (Z)-enolate

Using Gaussian 09: AM64L-G09RevD.01 24-Apr-2013
===
==============================
# pbepbe/6-31G*/auto gfprint gfinput
scf=(direct,tight,maxcycle=300,xqc)
opt=(maxcycle=250) freq=noraman iop(1/8=18)
Temperature=298.15
SCRF=(PCM,SOLVENT=Dichloromethane)
#N Geom=AllCheck Guess=TCheck SCRF=Check Test GenChk
RPBEPBE/6-31G(d)/Auto
Freq

Pointgroup= C1 Stoichiometry= C27H26N2OS
C1[X(C27H26N2OS)] #Atoms= 57
Charge = 0 Multiplicity = 1

SCF Energy= -1625.74443064 Predicted Change= -3.924062D-09
===
Optimization completed. {Found 2 times}
Item Max Val. Criteria Pass? RMS Val. Criteria Pass?
Force 0.00001 || 0.00045 [YES] 0.00000 || 0.00030 [YES]
Displ 0.00122 || 0.00180 [YES] 0.00122 || 0.00180 [YES]

Atomic Coordinates (Angstroms)

Type	X	Y	Z
C	-5.609609	-1.814045	0.472444
C	-4.676193	-0.813184	0.166808
C	-3.324293	-1.180355	0.095811
C	-2.911781	-2.504535	0.332271
C	-3.849872	-3.499223	0.632287
C	-5.204851	-3.141851	0.701348
N	-2.235938	-0.340545	-0.196868
C	-1.020402	-0.959098	-0.163658
S	-1.143145	-2.687444	0.171518
C	-2.385925	1.076545	-0.555750
C	-1.176614	1.509970	-1.381877
C	-1.257471	2.990405	-1.869298
C	-1.993009	3.058423	-3.221596
C	-1.899093	3.970065	-0.868062
C	0.134800	1.152626	-0.625244
C	0.428579	1.977621	0.623115
N	0.139639	-0.310550	-0.341481
C	1.267752	3.104985	0.514218
C	1.521975	3.920976	1.625109
C	0.948414	3.614330	2.868048
C	0.127903	2.483389	2.992300
C	-0.129065	1.669392	1.879389
C	1.407711	-1.101943	-0.296938
C	2.597680	-0.382536	-0.323442
C	3.927655	-0.962637	-0.311621
O	1.218507	-2.357590	-0.230718
C	5.055153	-0.091128	-0.309882
C	6.365112	-0.578517	-0.302025
C	6.611465	-1.962718	-0.297276
C	5.515074	-2.841904	-0.298744
C	4.200625	-2.361274	-0.304800
H	-6.669612	-1.550508	0.535155
H	-4.999497	0.217858	0.000828
H	-3.531906	-4.531198	0.811038
H	-5.951933	-3.905214	0.938401
H	-2.492279	1.673898	0.367141
H	-3.313633	1.175609	-1.141915
H	-3.037419	2.707989	-3.129434
H	-1.492782	2.439419	-3.986484
H	-2.025091	4.097416	-3.592189
H	-0.215958	3.322673	-2.044856
H	-2.976571	3.764224	-0.734734
H	-1.811737	5.000348	-1.253269
H	-1.416687	3.940483	0.121910
H	-1.158810	0.872463	-2.287017
H	0.954782	1.302512	-1.346913
H	1.729724	3.344707	-0.450868
H	2.177824	4.791237	1.520078
H	1.150060	4.246190	3.739001
H	-0.310938	2.226177	3.961777
H	-0.750818	0.776346	2.004616
H	2.565006	0.707806	-0.298586
H	4.882136	0.992724	-0.314272
H	7.203227	0.128465	-0.299986
H	7.637005	-2.347130	-0.291840
H	5.686266	-3.925252	-0.293835
H	3.355686	-3.054444	-0.301465

Statistical Thermodynamic Analysis
Temperature= 298.150 Kelvin Pressure= 1.00000 Atm
===
SCF Energy= -1625.74443064 Predicted Change= -3.924062D-09
Zero-point correction (ZPE)= -1625.2866 0.45773
Internal Energy (U)= -1625.2594 0.48498
Enthalpy (H)= -1625.2585 0.48592
Gibbs Free Energy (G)= -1625.3446 0.39980

Frequencies -- 25.5687 26.3653 37.0329

183.15K thermal correction = 0.429069
Single point SCF = -1626.123085

TS-E-(Re,Si): Concerted [2+2] β-lactone formation

Using Gaussian 09: AM64L-G09RevD.01 24-Apr-2013
===
# pbepbe/6-31G*/auto gfprint gfinput scf=(direct,tight,maxcycle=300,xqc)
SCRF=(PCM,SOLVENT=Dichloromethane)
opt=(maxcycle=250,calcfc,ts,noeigentest) freq=noraman iop(1/8=18)
Temperature=298.15
#N Geom=AllCheck Guess=TCheck SCRF=Check Test GenChk RPBEPBE/6-31G(d)/Auto
Freq

Pointgroup= C1 Stoichiometry= C35H30BrF3N2O2S
C1[X(C35H30BrF3N2O2S)] #Atoms= 74
Charge = 0 Multiplicity = 1

SCF Energy= -4878.33291875 Predicted Change= -3.251482D-09
===
Optimization completed. {Found 2 times}
Item Max Val. Criteria Pass? RMS Val. Criteria Pass?
Force 0.00001 || 0.00045 [YES] 0.00000 || 0.00030 [YES]
Displ 0.00179 || 0.00180 [YES] 0.00179 || 0.00180 [YES]

Atomic Coordinates (Angstroms)

Type	X	Y	Z
N	3.492578	1.499507	-0.138393
C	4.122773	0.513695	-1.039012
C	3.030551	-0.297429	-1.738019
C	3.591039	-1.373872	-2.707893
C	4.726416	-0.824187	-3.593168
C	2.471022	-1.937954	-3.604333
C	2.065975	-0.885071	-0.674286
C	2.659508	-1.953440	0.235021
N	1.489995	0.249128	0.118179

C	0.121692	0.226160	0.567470
C	-0.638744	-1.088896	0.524043
H	0.083703	-1.901329	0.356917
C	-1.365385	-1.418996	1.813833
O	-0.326898	1.265038	1.056680
C	2.219708	1.375193	0.312271
S	1.656650	2.790172	1.180086
C	3.256138	3.530524	0.979448
C	4.118514	2.700181	0.238566
C	3.684597	4.782162	1.439877
C	4.996718	5.177438	1.149923
C	5.859083	4.339693	0.415582
C	5.435234	3.089723	-0.050431
C	3.398592	-1.644137	1.395377
C	3.946945	-2.661969	2.189482
C	3.763331	-4.007577	1.839965
C	3.019092	-4.329422	0.695389
C	2.468490	-3.311851	-0.096363
C	-1.239465	-2.728495	2.328823
C	-1.905113	-3.117951	3.498157
C	-2.709767	-2.198365	4.188496
C	-2.840739	-0.892518	3.692801
C	-2.182239	-0.505999	2.516271
H	4.803954	-0.129109	-0.451552
H	4.719539	1.090148	-1.761103
H	4.006195	-2.199537	-2.098521
H	5.026171	-1.588380	-4.330156
H	5.628531	-0.558930	-3.015673
H	4.399212	0.069512	-4.155766
H	2.864101	-2.758139	-4.229057
H	2.087651	-1.152746	-4.281007
H	1.609321	-2.326982	-3.037972
H	2.421922	0.407831	-2.338183
H	1.177209	-1.241180	-1.233665
H	3.013596	5.430639	2.010605
H	5.354613	6.149272	1.501849
H	6.881746	4.665931	0.205662
H	6.114089	2.440469	-0.608916
H	3.535735	-0.602445	1.705433
H	4.513900	-2.398893	3.088356
H	4.188410	-4.801310	2.462675
H	2.854592	-5.376540	0.421298
H	1.863113	-3.573037	-0.970077
H	-0.607178	-3.451953	1.800702
H	-1.788172	-4.140050	3.874450
H	-3.227661	-2.496531	5.106374
H	-3.464832	-0.164880	4.223216
H	-2.290074	0.514144	2.142768
C	-1.413290	-0.971009	-0.927257
C	-1.728919	-2.432328	-1.390455
F	-2.430513	-2.439877	-2.554430
F	-2.438010	-3.195450	-0.492183
F	-0.570814	-3.133548	-1.628416
C	-2.744940	-0.181110	-0.791331
O	-0.545845	-0.418408	-1.790434
C	-3.911041	-0.638625	-0.143988
C	-5.069663	0.151718	-0.099025
C	-5.053844	1.415309	-0.700866
Br	-6.638066	2.502260	-0.641997
C	-3.913196	1.898822	-1.352558
C	-2.771406	1.088017	-1.394608
H	-3.935458	-1.625246	0.323831
H	-5.972627	-0.212832	0.398810
H	-3.921304	2.885373	-1.825229
H	-1.862106	1.406667	-1.913959

Statistical Thermodynamic Analysis
Temperature= 298.150 Kelvin Pressure= 1.00000 Atm
==

SCF Energy= -4878.33291875 Predicted Change= -3.251482D-09
Zero-point correction (ZPE)= -4877.7713 0.56153
Internal Energy (U)= -4877.7324 0.60044
Enthalpy (H)= -4877.7315 0.60138
Gibbs Free Energy (G)= -4877.8445 0.48836

Frequencies -- -58.3967 17.2912 22.6303

183.15K thermal correction = 0.526412
Single point SCF = -4881.361935

TS-E-(Re,Re): Concerted [2+2] β-lactone formation

Using Gaussian 09: AM64L-G09RevD.01 24-Apr-2013
==
# pbepbe/6-31G(d)/auto scf=(maxcycle=300,direct,tight,xqc) density=current
opt=(maxcycle=250,modredundant) SCRF=(PCM,SOLVENT=Dichloromethane)
iop(1/8=18) Temperature=298.15
Modredundant Input: B 10 64 F
Modredundant Input: B 11 58 F
Modredundant Input:
# pbepbe/6-31G(d)/auto scf=(direct,tight,maxcycle=300,xqc)
opt=(nofreeze,maxcycle=250,ts,calcfc,noeigentest) iop(1/8=18) freq=noraman
SCRF=(PCM,SOLVENT=Dichloromethane) Temperature=298.15 geom=check
guess=read output=WFX
#N Geom=AllCheck Guess=TCheck SCRF=Check Test GenChk RPBEPBE/6-31G(d)/Auto
Freq

Pointgroup= C1 Stoichiometry= C35H30BrF3N2O2S
C1[X(C35H30BrF3N2O2S)] #Atoms= 74
Charge = 0 Multiplicity = 1

SCF Energy= -4878.32888447 Predicted Change= -1.665278D-08
==
Optimization completed. {Found 2 times}

Item	Max Val.	Criteria	Pass?	RMS Val.	Criteria	Pass?
Force	0.00002 ||	0.00045	[YES]	0.00000 ||	0.00030	[YES]
Displ	0.00201 ||	0.00180	[NO]	0.00201 ||	0.00180	[YES]

Atomic Coordinates (Angstroms)

Type	X	Y	Z
N	-3.958029	-0.816610	0.194805
C	-3.461464	-2.203458	0.150446
C	-2.037097	-2.254337	0.705919
C	-1.445518	-3.691353	0.673947
C	-2.438914	-4.739581	1.213838
C	-0.134468	-3.772872	1.477967
C	-1.154649	-1.207470	-0.027896
C	-0.860807	-1.510919	-1.492220
N	-1.785961	0.145517	0.127082
C	-0.992726	1.345602	0.213838
C	0.492717	1.276213	-0.123760
H	0.667941	0.340531	-0.675073
C	0.910774	2.400815	-1.059530

O	-1.598170	2.393360	0.444987
C	-3.133328	0.257515	0.249170
S	-3.992036	1.770851	0.464548
C	-5.529422	0.887676	0.418232
C	-5.324791	-0.496896	0.269500
C	-6.822425	1.416236	0.522219
C	-7.904092	0.528449	0.466061
C	-7.695325	-0.855935	0.308210
C	-6.405521	-1.390223	0.206500
C	-1.754242	-1.180419	-2.531899
C	-1.451876	-1.493982	-3.865117
C	-0.248831	-2.140021	-4.183952
C	0.655274	-2.462647	-3.160994
C	0.353835	-2.145872	-1.828801
C	0.710621	3.770202	-0.777921
C	1.125452	4.754166	-1.685244
C	1.753090	4.398095	-2.889521
C	1.958310	3.042225	-3.183088
C	1.536273	2.057600	-2.278428
H	-3.510355	-2.570912	-0.891152
H	-4.147919	-2.801734	0.766541
H	-1.226821	-3.943768	-0.381439
H	-1.947356	-5.725984	1.262983
H	-3.329889	-4.854707	0.573490
H	-2.776102	-4.484916	2.235440
H	0.634314	-3.067848	1.122590
H	0.287581	-4.790060	1.409886
H	-0.316333	-3.553556	2.545692
H	-2.077940	-1.930009	1.764856
H	-0.229910	-1.090654	0.578964
H	-6.980865	2.491839	0.641977
H	-8.923257	0.917981	0.543206
H	-8.554706	-1.530853	0.260544
H	-6.254296	-2.464224	0.072818
H	-2.688416	-0.650667	-2.314798
H	-2.158411	-1.223904	-4.656571
H	-0.011851	-2.380392	-5.225340
H	1.605258	-2.952039	-3.398606
H	1.079045	-2.380176	-1.041628
H	0.225938	4.063912	0.154697
H	0.958700	5.810315	-1.446515
H	2.076707	5.171801	-3.593932
H	2.440305	2.746738	-4.121271
H	1.690055	0.999439	-2.521814
C	1.251269	0.946256	1.298258
C	1.461727	2.237098	2.156094
F	0.284512	2.864642	2.443764
F	2.279647	3.193157	1.600951
F	2.029861	1.903691	3.350386
C	2.669209	0.370215	0.971931
O	0.468373	0.094569	1.976253
C	2.935484	-0.916140	1.469444
C	4.156904	-1.560453	1.229240
C	5.126828	-0.892568	0.472607
Br	6.808540	-1.756205	0.123378
C	4.901332	0.394241	-0.031273
C	3.672774	1.022269	0.227454
H	2.143170	-1.383966	2.063739
H	4.355312	-2.560376	1.626341
H	5.674319	0.902767	-0.614717
H	3.511346	2.031454	-0.158309

Statistical Thermodynamic Analysis
Temperature= 298.150 Kelvin Pressure= 1.00000 Atm
== ==============================
SCF Energy= -4878.32888447 Predicted Change= -1.665278D-08
Zero-point correction (ZPE)= -4877.7670 0.56183
Internal Energy (U)= -4877.7283 0.60050
Enthalpy (H)= -4877.7274 0.60144
Gibbs Free Energy (G)= -4877.8390 0.48985

Frequencies -- -27.2345 13.9385 23.0592

183.15K thermal correction = 0.527360
Single point SCF = -4881.359206

TS-E-(Si,Re): Concerted [2+2] β-lactone formation

Using Gaussian 09: AM64L-G09RevD.01 24-Apr-2013
== ==============================
#PBEPBE/6-31G(d)/auto scf=(maxcycle=300,direct,tight) density=current
SCRF=(PCM,SOLVENT=Dichloromethane)
opt=(maxcycle=250,ts,calcfc,noeigentest) freq=noraman
#P Geom=AllCheck Guess=TCheck SCRF=Check GenChk RPBEPBE/6-31G(d)/Auto Freq
----
Pointgroup= C1 Stoichiometry= C35H30BrF3N2O2S
C1[X(C35H30BrF3N2O2S)] #Atoms= 74
Charge = 0 Multiplicity = 1

SCF Energy= -4878.31945868 Predicted Change= -1.076491D-08
== ==============================
Optimization completed. {Found 2 times}
Item Max Val. Criteria Pass? RMS Val. Criteria Pass?
Force 0.00002 || 0.00045 [YES] 0.00000 || 0.00030 [YES]
Displ 0.00117 || 0.00180 [YES] 0.00117 || 0.00180 [YES]

Atomic Coordinates (Angstroms)

Type	X	Y	Z
C	5.133979	4.856932	0.209045
H	6.116025	5.290836	0.417805
C	5.015580	3.466929	0.093159
H	5.886075	2.818205	0.218740
C	3.740663	2.943418	-0.168901
C	2.617333	3.782295	-0.302158
C	2.743325	5.172791	-0.191724
H	1.872962	5.826036	-0.300861
C	4.014692	5.700606	0.067305
H	4.136367	6.783363	0.163008
N	3.399121	1.589257	-0.324509
C	2.079432	1.372970	-0.532166
S	1.151273	2.851353	-0.658428
C	4.394380	0.502418	-0.370802
H	4.669292	0.217102	0.661109
H	5.281487	0.910238	-0.876108
C	3.804613	-0.674053	-1.152373
C	4.798791	-1.856845	-1.317412
H	4.917265	-2.348375	-0.333503
C	6.190056	-1.375627	-1.774987
H	6.702247	-0.766935	-1.010828
H	6.124495	-0.782002	-2.705226
H	6.834862	-2.246534	-1.980628
C	4.261860	-2.891899	-2.324269
H	3.289204	-3.321606	-2.030665
H	4.972730	-3.729915	-2.419977
H	4.139359	-2.438737	-3.324774
H	3.563543	-0.306938	-2.170363

C	2.445609	-1.065840	-0.490910
C	2.572767	-1.616422	0.926671
H	1.971208	-1.822511	-1.135541
N	1.544211	0.127826	-0.581078
C	0.114414	0.007931	-0.781256
C	-0.545625	-1.276326	-0.384729
H	0.196003	-1.972536	0.029304
C	-1.309109	-1.966252	-1.485910
O	-0.471558	0.989297	-1.239058
C	2.476254	-0.796606	2.065853
H	2.201063	0.255351	1.965236
C	2.666671	-1.337120	3.345751
H	2.583758	-0.686058	4.221918
C	2.936232	-2.702994	3.507016
H	3.076560	-3.122361	4.508703
C	3.002663	-3.534227	2.379251
H	3.188920	-4.607068	2.492834
C	2.817489	-2.994382	1.099367
H	2.853442	-3.656888	0.227175
C	-2.090318	-1.280549	-2.444270
H	-2.143716	-0.189308	-2.409564
C	-2.788117	-1.982393	-3.437354
H	-3.382664	-1.426406	-4.170790
C	-2.733776	-3.383423	-3.494530
H	-3.282069	-3.928390	-4.270262
C	-1.965964	-4.078611	-2.546499
H	-1.910189	-5.172333	-2.578615
C	-1.260305	-3.377927	-1.560592
H	-0.665430	-3.930202	-0.824074
C	-2.778083	1.294354	1.017202
H	-1.889749	1.781117	1.432703
C	-2.667876	-0.080495	0.743213
C	-1.292007	-0.722168	1.075695
C	-1.509593	-2.024030	1.920609
F	-0.322251	-2.602153	2.259001
F	-2.245003	-3.016742	1.306101
F	-2.154695	-1.733971	3.087063
C	-3.800074	-0.752144	0.237516
H	-3.763931	-1.823180	0.028477
C	-5.003879	-0.067959	0.007566
H	-5.879323	-0.596738	-0.379995
C	-5.069609	1.302798	0.282152
Br	-6.718171	2.243940	-0.029988
C	-3.965820	1.999873	0.788020
H	-4.038442	3.069664	1.005483
O	-0.439868	0.076906	1.686065

Statistical Thermodynamic Analysis
Temperature= 298.150 Kelvin Pressure= 1.00000 Atm
==
==============================

SCF Energy= -4878.31945868 Predicted Change= -1.076491D-08
Zero-point correction (ZPE)= -4877.7586 0.56078
Internal Energy (U)= -4877.7195 0.59993
Enthalpy (H)= -4877.7185 0.60087
Gibbs Free Energy (G)= -4877.8317 0.48770

Frequencies -- -194.0867 17.0598 20.3876

183.15K thermal correction = 0.525778
Single point SCF = -4881.352492

TS-E-(Si,Si): Concerted [2+2] β-lactone formation

Using Gaussian 09: AM64L-G09RevD.01 24-Apr-2013
==
==============================

# pbepbe/6-31G*/auto gfprint gfinput scf=(direct,tight,maxcycle=300,xqc)
SCRF=(PCM,SOLVENT=Dichloromethane)
opt=(maxcycle=250,calcfc,ts,noeigentest) freq=noraman iop(1/8=18)
Temperature=298.15
#N Geom=AllCheck Guess=TCheck SCRF=Check Test GenChk RPBEPBE/6-31G(d)/Auto
Freq

Pointgroup= C1 Stoichiometry= C35H30BrF3N2O2S
C1[X(C35H30BrF3N2O2S)] #Atoms= 74
Charge = 0 Multiplicity = 1

SCF Energy= -4878.31580133 Predicted Change= -3.857569D-09
==
==============================

Optimization completed. {Found 2 times}

Item	Max Val.	Criteria	Pass?	RMS Val.	Criteria	Pass?
Force	0.00000 ||	0.00045	[YES]	0.00000 ||	0.00030	[YES]
Displ	0.00082 ||	0.00180	[YES]	0.00082 ||	0.00180	[YES]

Atomic Type	Coordinates (Angstroms) X	Y	Z
N	3.903297	0.632441	-0.068147
C	3.711522	1.799566	0.812081
C	2.703257	1.436884	1.904422
C	2.466229	2.589462	2.917774
C	3.794767	3.192915	3.416402
C	1.644759	2.103113	4.127117
C	1.406223	0.906111	1.213960
C	0.675512	1.954445	0.380547
N	1.781214	-0.316218	0.433308
C	0.928909	-1.488293	0.345647
C	-0.539274	-1.314685	0.585547
H	-0.756106	-0.271854	0.852891
C	-1.085604	-2.213307	1.669309
O	1.482967	-2.546434	0.050614
C	3.017172	-0.388917	-0.125792
S	3.606597	-1.757978	-1.042737
C	5.062797	-0.835364	-1.455159
C	5.068895	0.424122	-0.825712
C	6.140514	-1.244589	-2.250948
C	7.214122	-0.359475	-2.411438
C	7.212339	0.902741	-1.785506
C	6.141805	1.314249	-0.983167
C	-0.240707	2.812555	1.023188
C	-0.863654	3.853845	0.321276
C	-0.582947	4.049172	-1.039008
C	0.301806	3.180619	-1.694158
C	0.918806	2.133094	-0.994316
C	-1.936303	-1.655875	2.651691
C	-2.479806	-2.443598	3.675963
C	-2.174711	-3.810858	3.750475
C	-1.326893	-4.377852	2.784955
C	-0.793179	-3.594222	1.754253
H	3.375474	2.660272	0.205417
H	4.696820	2.031648	1.241063
H	1.902355	3.391404	2.405117
H	3.592702	3.924633	4.216563
H	4.344888	3.725895	2.622880
H	4.457877	2.414590	3.836644
H	1.482094	2.935688	4.832004

H	2.181346	1.303343	4.669274
H	0.650746	1.710594	3.852477
H	3.124285	0.583784	2.473623
H	0.741115	0.537602	2.009907
H	6.140467	-2.224850	-2.736045
H	8.063874	-0.654526	-3.033721
H	8.059479	1.579445	-1.929230
H	6.141823	2.299794	-0.510557
H	-0.476873	2.664239	2.082718
H	-1.573192	4.506973	0.839201
H	-1.065512	4.862987	-1.589982
H	0.504597	3.305387	-2.762736
H	1.556035	1.431680	-1.538704
H	-2.175187	-0.586651	2.607683
H	-3.136098	-1.983576	4.422936
H	-2.591788	-4.428588	4.552816
H	-1.082088	-5.444893	2.830707
H	-0.144012	-4.049719	1.002447
C	-1.167136	-1.284623	-1.048762
C	-1.468059	-2.744072	-1.538750
F	-2.067586	-2.681215	-2.768423
F	-2.315021	-3.485989	-0.746096
F	-0.345310	-3.493730	-1.698190
C	-2.534745	-0.531166	-0.935020
O	-0.279772	-0.707837	-1.822330
C	-3.662487	-0.954359	-0.201593
C	-4.832699	-0.179447	-0.165761
C	-4.870734	1.026311	-0.875964
Br	-6.471593	2.091534	-0.832114
C	-3.770091	1.471805	-1.617527
C	-2.612702	0.682734	-1.637234
H	-3.645491	-1.897561	0.348488
H	-5.704079	-0.512442	0.405527
H	-3.818338	2.415889	-2.168559
H	-1.719102	0.983549	-2.193849

Statistical Thermodynamic Analysis
Temperature= 298.150 Kelvin   Pressure= 1.00000 Atm
==
==============================
SCF Energy=   -4878.31580133   Predicted Change= -3.857569D-09
Zero-point correction (ZPE)=   -4877.7547   0.56105
Internal Energy (U)=   -4877.7155   0.60024
Enthalpy (H)=   -4877.7146   0.60119
Gibbs Free Energy (G)=   -4877.8289   0.48684

Frequencies -- -167.4953   11.2703   15.1602

183.15K thermal correction = 0.525374
Single point SCF = -4881.349302

F: Product-catalyst complex

Using Gaussian 09: AM64L-G09RevD.01 24-Apr-2013
==
==============================
# pbepbe/6-31G*/auto gfprint gfinput scf=(direct,tight,maxcycle=300,xqc)
opt=(maxcycle=250) freq=noraman iop(1/8=18) Temperature=298.15
SCRF=(PCM,SOLVENT=Dichloromethane)
#N Geom=AllCheck Guess=TCheck SCRF=Check Test GenChk RPBEPBE/6-31G(d)/Auto
Freq

Pointgroup= C1 Stoichiometry= C35H30BrF3N2O2S
C1[X(C35H30BrF3N2O2S)] #Atoms= 74
Charge = 0   Multiplicity = 1

SCF Energy= -4878.34350813 Predicted Change= -3.770202D-08
==
==============================
Optimization completed.   {Found   1   times}

Item	Max Val.	Criteria	Pass?	RMS Val.	Criteria	Pass?
Force	0.00003 ||	0.00045	[YES]	0.00000 ||	0.00030	[YES]
Displ	0.00182 ||	0.00180	[NO]	0.00182 ||	0.00180	[YES]

Atomic Type	Coordinates (Angstroms) X	Y	Z
N	3.683793	1.298021	-0.371778
C	4.359937	0.131780	-0.963285
C	3.329732	-0.757796	-1.664341
C	3.953282	-2.036944	-2.285695
C	5.250136	-1.734766	-3.061766
C	2.952134	-2.738803	-3.223452
C	2.159615	-1.044614	-0.675073
C	2.534812	-1.925022	0.518680
N	1.566934	0.244218	-0.245420
C	0.037932	0.411336	-0.057951
C	-0.686748	-0.833105	0.607488
C	-1.279256	-0.706763	1.983468
O	-0.251779	1.597413	0.290251
C	2.343271	1.321300	-0.124372
S	1.768330	2.907441	0.394105
C	3.450098	3.505571	0.330518
C	4.344571	2.510517	-0.104066
C	3.912125	4.790557	0.637872
C	5.283693	5.056965	0.510697
C	6.173680	4.054416	0.083595
C	5.717848	2.765644	-0.229125
C	3.096682	-1.398043	1.698841
C	3.445808	-2.239756	2.765251
C	3.235167	-3.623116	2.673500
C	2.664713	-4.159182	1.509503
C	2.314931	-3.316886	0.444437
C	-2.017321	0.424089	2.397038
C	-2.540135	0.497079	3.695914
C	-2.343974	-0.555696	4.603127
C	-1.611024	-1.684271	4.205749
C	-1.082832	-1.753928	2.909123
H	-0.020823	-1.710250	0.597968
H	4.904398	-0.416395	-0.171273
H	5.095229	0.521156	-1.683466
H	4.207712	-2.729629	-1.460381
H	5.611176	-2.652406	-3.556590
H	6.063413	-1.376854	-2.408069
H	5.080214	-0.977428	-3.849256
H	3.392699	-3.667928	-3.623466
H	2.702876	-2.088808	-4.082081
H	2.005116	-3.009175	-2.727765
H	2.889436	-0.165784	-2.491363
H	1.357787	-1.531411	-1.246298
H	3.218410	5.568619	0.971686
H	5.662938	6.055148	0.749350
H	7.241275	4.276884	-0.004490
H	6.416921	1.988099	-0.548615
H	3.245196	-0.318671	1.807019
H	3.876416	-1.807621	3.674346
H	3.503624	-4.278281	3.508519
H	2.480758	-5.235614	1.430738

```
H      1.845654    -3.744059    -0.447960
H     -2.154909     1.251014     1.697209
H     -3.107225     1.383528     4.000472
H     -2.757912    -0.495724     5.615532
H     -1.446603    -2.510248     4.906046
H     -0.502514    -2.634188     2.606230
C     -1.528326    -0.876857    -0.717550
C     -2.924739    -0.253863    -0.698880
C     -1.609865    -2.281586    -1.354352
F     -2.189035    -2.241828    -2.580359
F     -2.327686    -3.146127    -0.581937
F     -0.375986    -2.856020    -1.522761
O     -0.572172    -0.080683    -1.432308
C     -3.162842     0.898258    -1.467420
C     -4.429842     1.495602    -1.501856
C     -5.465781     0.925143    -0.753273
Br    -7.205728     1.731802    -0.791223
C     -5.258123    -0.219341     0.026030
C     -3.984822    -0.804183     0.049211
H     -2.331249     1.328468    -2.030893
H     -4.607414     2.391941    -2.102239
H     -6.076187    -0.647658     0.611275
H     -3.825535    -1.694991     0.661778
---------------------------------------------------------------------
 Statistical Thermodynamic Analysis
 Temperature= 298.150 Kelvin       Pressure= 1.00000 Atm
==============================================
==============================
 SCF Energy=     -4878.34350813               Predicted Change= -
3.770202D-08
 Zero-point correction (ZPE)=          -4877.7805             0.56295
 Internal Energy (U)=                  -4877.7414             0.60203
 Enthalpy (H)=                                   -4877.7405
0.60298
 Gibbs Free Energy (G)=                          -4877.8543
0.48916
---------------------------------------------------------------------
 Frequencies --     14.8837               22.0917               26.3967

183.15K thermal correction = 0.527488
Single point SCF = -4881.367879
```

TS-G: Catalyst release

```
---------------------------------------------------------------------
 Using  Gaussian 09:  AM64L-G09RevD.01 24-Apr-2013
==============================================
==============================
 # pbepbe/6-31G(d)/auto scf=(maxcycle=300,direct,tight,xqc)
density=current
 opt=(maxcycle=250,modredundant)
SCRF=(PCM,SOLVENT=Dichloromethane)
 iop(1/8=18) Temperature=298.15
 Modredundant Input:  B       9      10 F
 Modredundant Input:
 # pbepbe/6-31G(d)/auto scf=(direct,tight,maxcycle=300,xqc)
 opt=(nofreeze,maxcycle=250,ts,calcfc,noeigentest) iop(1/8=18)
freq=noraman
 SCRF=(PCM,SOLVENT=Dichloromethane)
Temperature=298.15 geom=check
 guess=read
 #N Geom=AllCheck Guess=TCheck SCRF=Check Test GenChk
RPBEPBE/6-31G(d)/Auto
 Freq
---------------------------------------------------------------------
 Pointgroup= C1   Stoichiometry= C35H30BrF3N2O2S
C1[X(C35H30BrF3N2O2S)]  #Atoms= 74
 Charge = 0                Multiplicity = 1
---------------------------------------------------------------------
 SCF Energy= -4878.33805562    Predicted Change= -3.366135D-
09
==============================================
==============================
 Optimization completed on the basis of negligible forces.
{Found        3        times}
 Item      Max Val.    Criteria    Pass?      RMS Val.    Criteria
Pass?
 Force      0.00000 ||  0.00045   [ YES ]      0.00000 ||  0.00030   [
YES ]
 Displ      0.00274 ||  0.00180   [ NO ]       0.00274 ||  0.00180   [
YES ]
---------------------------------------------------------------------
     Atomic     Coordinates (Angstroms)
      Type    X                  Y              Z
---------------------------------------------------------------------
N      3.913710     1.100310    -0.559937
C      4.517652    -0.164139    -1.003263
C      3.435720    -1.072683    -1.599514
C      3.982975    -2.452029    -2.054030
C      5.283308    -2.323138    -2.872130
C      2.933796    -3.214476    -2.886392
C      2.239232    -1.150365    -0.596061
C      2.557400    -1.893773     0.707538
N      1.720176     0.198878    -0.329571
C     -0.214304     0.580033    -0.246615
C     -0.807433    -0.435747     0.772084
C     -1.439287     0.024075     2.054847
O     -0.285726     1.807744    -0.275290
C      2.557205     1.200224    -0.323312
S      2.103403     2.893928     0.038130
C      3.806369     3.376958    -0.066525
C      4.637273     2.287736    -0.401601
C      4.343014     4.653980     0.126219
C      5.730230     4.826581    -0.010513
C      6.557448     3.738391    -0.336554
C      6.023363     2.455764    -0.535114
C      3.070518    -1.230069     1.840710
C      3.375885    -1.936184     3.013814
C      3.167550    -3.321271     3.081092
C      2.644890    -3.993370     1.966373
C      2.339902    -3.285611     0.794981
C     -2.111566     1.259459     2.165713
C     -2.690329     1.646081     3.382514
C     -2.614517     0.805652     4.503864
C     -1.947479    -0.425074     4.405451
C     -1.361274    -0.807845     3.191286
H     -0.063048    -1.219378     0.985762
H      5.029940    -0.649864    -0.150407
H      5.274815     0.085592    -1.762780
H      4.210952    -3.044594    -1.146889
H      5.589272    -3.313241    -3.251610
H      6.123179    -1.928939    -2.275210
H      5.140763    -1.660640    -3.745862
H      3.316563    -4.211731    -3.163335
H      2.709816    -2.669431    -3.821767
H      1.979743    -3.361681    -2.353232
H      3.036871    -0.560606    -2.498514
H      1.415732    -1.675077    -1.102462
H      3.695911     5.498902     0.381058
H      6.166204     5.818429     0.141752
H      7.637155     3.885903    -0.435943
H      6.674471     1.611897    -0.779050
H      3.217255    -0.145694     1.822070
H      3.770828    -1.396268     3.880797
H      3.401277    -3.871344     3.998513
H      2.463586    -5.072517     2.008529
H      1.913736    -3.823676    -0.057544
H     -2.161654     1.921917     1.297713
H     -3.204383     2.610495     3.454062
```

H	-3.069686	1.110578	5.452179
H	-1.877479	-1.085742	5.275992
H	-0.834043	-1.766652	3.118818
C	-1.634369	-0.906556	-0.488193
C	-3.050816	-0.357156	-0.628318
C	-1.630916	-2.427196	-0.742162
F	-2.243522	-2.737272	-1.911049
F	-2.278027	-3.085732	0.260149
F	-0.371599	-2.948469	-0.802433
O	-0.702465	-0.241644	-1.381650
C	-3.338312	0.550370	-1.662634
C	-4.628581	1.072459	-1.824908
C	-5.635794	0.675540	-0.938163
Br	-7.403000	1.385369	-1.145480
C	-5.377090	-0.225153	0.102432
C	-4.082323	-0.737607	0.253373
H	-2.535190	0.855770	-2.337967
H	-4.844820	1.778740	-2.630812
H	-6.172939	-0.520293	0.791052
H	-3.883646	-1.434678	1.071157

Statistical Thermodynamic Analysis
Temperature= 298.150 Kelvin Pressure= 1.00000 Atm
==
============================
SCF Energy= -4878.33805562 Predicted Change= -3.366135D-09
Zero-point correction (ZPE)= -4877.7767 0.56133
Internal Energy (U)= -4877.7376 0.60045
Enthalpy (H)= -4877.7366 0.60139
Gibbs Free Energy (G)= -4877.8519 0.48615

Frequencies -- -120.1402 11.2233 15.2962

183.15K thermal correction = 0.525055
Single point SCF = -4881.361479

H: Major Product

Using Gaussian 09: AM64L-G09RevD.01 24-Apr-2013
==
============================
# pbepbe/6-31G*/auto gfprint gfinput scf=(direct,tight,maxcycle=300,xqc)
opt=(maxcycle=250) freq=noraman iop(1/8=18) Temperature=298.15
SCRF=(PCM,SOLVENT=Dichloromethane)
#N Geom=AllCheck Guess=TCheck SCRF=Check Test GenChk RPBEPBE/6-31G(d)/Auto
Freq

Pointgroup= C1 Stoichiometry= C16H10BrF3O2 C1[X(C16H10BrF3O2)] #Atoms= 32
Charge = 0 Multiplicity = 1

SCF Energy= -3635.82363720 Predicted Change= -2.628166D-08
==
============================
Optimization completed. {Found 1 times}

Item	Max Val.	Criteria	Pass?	RMS Val.	Criteria	Pass?
Force	0.00005 ||	0.00045	[YES]	0.00000 ||	0.00030	[YES]
Displ	0.00213 ||	0.00180	[NO]	0.00213 ||	0.00180	[YES]

Atomic Coordinates (Angstroms)

Type	X	Y	Z
C	-2.818399	-0.361377	1.535626
C	-2.494213	0.152116	0.128198
C	-2.025293	1.572147	-0.048204
O	-3.516316	-0.050911	2.467277
C	-2.302853	2.248924	-1.251947
C	-1.862455	3.565233	-1.446921
C	-1.145977	4.222612	-0.435769
C	-0.871324	3.558138	0.769546
C	-1.306781	2.240712	0.962757
C	-1.481988	-1.060538	0.131179
C	0.003136	-0.784486	0.110789
C	-1.886350	-2.175222	-0.853913
F	-1.179103	-3.307855	-0.637000
F	-1.669964	-1.775114	-2.135618
F	-3.204941	-2.478289	-0.734090
O	-1.960053	-1.455429	1.472590
C	0.803149	-1.145748	1.208928
C	2.183789	-0.906232	1.196367
C	2.757630	-0.302602	0.071547
Br	4.640235	0.033265	0.044796
C	1.980867	0.065413	-1.034766
C	0.602860	-0.178175	-1.010777
H	-3.343711	-0.054682	-0.545144
H	-2.867990	1.738883	-2.040543
H	-2.086222	4.078955	-2.387366
H	-0.806735	5.252916	-0.584007
H	-0.318257	4.067809	1.564985
H	-1.091454	1.733610	1.909658
H	0.344584	-1.614404	2.083607
H	2.802504	-1.187393	2.052388
H	2.443221	0.541097	-1.903291
H	-0.001958	0.115496	-1.872537

Statistical Thermodynamic Analysis
Temperature= 298.150 Kelvin Pressure= 1.00000 Atm
==
============================
SCF Energy= -3635.82363720 Predicted Change= -2.628166D-08
Zero-point correction (ZPE)= -3635.6047 0.21892
Internal Energy (U)= -3635.5858 0.23781
Enthalpy (H)= -3635.5848 0.23875
Gibbs Free Energy (G)= -3635.6549 0.16870

Frequencies -- 22.5887 33.6879 38.7222

183.15K thermal correction = 0.193071
Single point SCF = -3638.589695

Stepwise Mechanism Coordinate Diagram: M06-2X

SM: Phenylacetic anhydride

Using Gaussian 09: AM64L-G09RevD.01 24-Apr-2013
==
============================
#m062X/6-31G(d) scf=(direct,tight,maxcycle=300,xqc)
SCRF=(PCM,SOLVENT=CH2Cl2) opt=(maxcycle=250,gdiis) iop(1/8=18)
freq=noraman temp=298.15
#N Geom=AllCheck Guess=TCheck SCRF=Check Test GenChk RM062X/6-31G(d) Freq

----
Pointgroup= C1 Stoichiometry= C16H14O3
C1[X(C16H14O3)] #Atoms= 33
Charge = 0 Multiplicity = 1

SCF Energy= -843.486915789 Predicted Change= -3.820527D-09
==
==============================
Optimization completed. {Found 1 times}

Item	Max Val.	Criteria	Pass?	RMS Val.	Criteria	Pass?
Force	0.00001 ||	0.00045	[YES]	0.00000 ||	0.00030	[YES]
Displ	0.00231 ||	0.00180	[NO]	0.00231 ||	0.00180	[YES]

Atomic Coordinates (Angstroms)

Type	X	Y	Z
C	-0.595515	2.145332	-0.692303
C	-2.079794	1.955837	-0.480554
C	-2.375875	0.503949	-0.166258
O	0.079189	1.773280	0.448236
C	1.441701	1.972420	0.584699
C	2.028653	0.874869	1.438034
C	2.057399	-0.428005	0.659573
O	2.022292	2.895958	0.097143
O	-0.057733	2.495045	-1.702311
C	-2.276377	-0.456548	-1.175441
C	-2.489986	-1.802235	-0.890935
C	-2.800560	-2.201456	0.408357
C	-2.901271	-1.247391	1.419126
C	-2.688827	0.099698	1.131895
C	3.236666	-0.837091	0.034358
C	3.276429	-2.025451	-0.691390
C	2.133499	-2.813575	-0.800044
C	0.951232	-2.405748	-0.185072
C	0.909347	-1.215871	0.537429
H	-2.581118	2.283088	-1.393637
H	-2.394275	2.598433	0.347652
H	1.412928	0.770661	2.336883
H	3.035010	1.182300	1.726788
H	-2.026127	-0.147432	-2.187697
H	-2.408935	-2.540318	-1.683320
H	-2.966920	-3.251080	0.630540
H	-3.146825	-1.550489	2.432318
H	-2.763627	0.842536	1.921803
H	4.129991	-0.223817	0.120808
H	4.201006	-2.334763	-1.169219
H	2.163316	-3.741986	-1.362004
H	0.054804	-3.013218	-0.263890
H	-0.019536	-0.900585	1.009598

Statistical Thermodynamic Analysis
Temperature= 298.150 Kelvin Pressure= 1.00000 Atm
==
==============================
SCF Energy= -843.486915789 Predicted Change= -3.820527D-09
Zero-point correction (ZPE)= -843.2210 0.26584
Internal Energy (U)= -843.2049 0.28198
Enthalpy (H)= -843.2039 0.28292
Gibbs Free Energy (G)= -843.2679 0.21898

Frequencies -- 22.6069 28.6918 36.3447

183.15K thermal correction = 0.241399

Single point SCF = -843.767988

SM: HyperBTM

Using Gaussian 09: AM64L-G09RevD.01 24-Apr-2013
==
==============================
#m062X/6-31G(d) scf=(direct,tight,maxcycle=300,xqc) SCRF=(PCM,SOLVENT=CH2Cl2) opt=(maxcycle=250,gdiis) iop(1/8=18) freq=noraman temp=298.15
#N Geom=AllCheck Guess=TCheck SCRF=Check Test GenChk RM062X/6-31G(d) Freq
----
Pointgroup= C1 Stoichiometry= C19H20N2S
C1[X(C19H20N2S)] #Atoms= 42
Charge = 0 Multiplicity = 1

SCF Energy= -1243.38846090 Predicted Change= -4.688311D-09
==
==============================
Optimization completed. {Found 2 times}

Item	Max Val.	Criteria	Pass?	RMS Val.	Criteria	Pass?
Force	0.00002 ||	0.00045	[YES]	0.00000 ||	0.00030	[YES]
Displ	0.00068 ||	0.00180	[YES]	0.00068 ||	0.00180	[YES]

Atomic Coordinates (Angstroms)

Type	X	Y	Z
C	4.268659	0.778416	-1.761556
H	4.685834	1.287067	-2.624624
C	2.913987	0.938619	-1.468712
H	2.279845	1.555938	-2.096148
C	2.393891	0.279654	-0.358831
C	3.216277	-0.535918	0.433783
C	4.561599	-0.692359	0.138996
H	5.191727	-1.322522	0.758345
C	5.086896	-0.024675	-0.969250
H	6.137912	-0.137601	-1.212610
N	1.080211	0.321755	0.091078
C	0.804302	-0.458551	1.198570
S	2.299800	-1.262633	1.753510
C	0.046857	1.171565	-0.484829
H	-0.349705	0.716713	-1.403878
H	0.513671	2.124029	-0.750947
C	-1.066086	1.397002	0.540958
C	-2.245401	2.178898	-0.064401
C	-3.241476	2.603065	1.018486
H	-2.753194	3.262222	1.746472
H	-3.661405	1.755070	1.565103
H	-4.075290	3.154530	0.573146
H	-2.760038	1.518728	-0.776034
C	-1.772951	3.422655	-0.825036
H	-1.157868	4.061667	-0.179272
H	-2.634208	4.011639	-1.154620
H	-1.187671	3.171066	-1.714341
H	-0.638365	2.005685	1.351940
C	-1.465843	0.044348	1.189838
C	-2.206301	-0.879998	0.232102
H	-2.145230	0.262754	2.019998
N	-0.311223	-0.640565	1.779908
C	-1.524389	-1.673177	-0.696633
H	-0.437603	-1.671915	-0.719797
C	-2.221229	-2.485467	-1.587940
H	-1.672513	-3.093996	-2.300510

C	-3.613495	-2.525901	-1.560566
H	-4.156337	-3.162247	-2.252687
C	-4.303160	-1.752056	-0.630747
H	-5.387731	-1.785203	-0.589822
C	-3.602403	-0.940645	0.258481
H	-4.149947	-0.352674	0.990943

Statistical Thermodynamic Analysis
Temperature= 298.150 Kelvin Pressure= 1.00000 Atm

===

SCF Energy= -1243.38846090 Predicted Change= -4.688311D-09
Zero-point correction (ZPE)= -1243.0339 0.35455
Internal Energy (U)= -1243.0154 0.37304
Enthalpy (H)= -1243.0144 0.37398
Gibbs Free Energy (G)= -1243.0808 0.30765

Frequencies -- 31.2209 37.3057 62.4494

183.15K thermal correction = 0.330526
Single point SCF = -1243.687102

SM: *N*,*N*-diisopropylethylamine

Using Gaussian 09: AM64L-G09RevD.01 24-Apr-2013

===

#m062X/6-31G(d) scf=(direct,tight,maxcycle=300,xqc)
SCRF=(PCM,SOLVENT=CH2Cl2) opt=(maxcycle=250,gdiis) iop(1/8=18)
freq=noraman temp=298.15
#N Geom=AllCheck Guess=TCheck SCRF=Check Test GenChk RM062X/6-31G(d) Freq

----

Pointgroup= C1 Stoichiometry= C8H19N C1[X(C8H19N)] #Atoms= 28
Charge = 0 Multiplicity = 1

SCF Energy= -370.843938747 Predicted Change= -3.133830D-08

===

Optimization completed. {Found 1 times}
Item Max Val. Criteria Pass? RMS Val. Criteria Pass?
Force 0.00000 || 0.00045 [YES] 0.00000 || 0.00030 [YES]
Displ 0.00335 || 0.00180 [NO] 0.00335 || 0.00180 [YES]

Atomic Coordinates (Angstroms)

Type	X	Y	Z
N	-0.002078	0.271983	-0.216425
C	1.033848	-0.771828	-0.199573
C	2.121484	-0.430091	-1.219155
C	1.665046	-1.064310	1.171263
C	-1.384017	-0.197054	-0.056551
C	-1.647176	-1.067090	1.180399
C	-1.868572	-0.898517	-1.326005
C	0.311186	1.454585	0.578273
C	-0.271193	2.731235	-0.022839
H	1.684488	-0.313212	-2.214472
H	2.885849	-1.213505	-1.256290
H	2.620152	0.509765	-0.954110
H	0.547169	-1.693820	-0.538197
H	2.314227	-1.943161	1.099619
H	0.911008	-1.260266	1.937432
H	2.284600	-0.227519	1.510904
H	-1.989769	0.710099	0.053370
H	-2.719384	-1.266363	1.278173
H	-1.312263	-0.573513	2.098695
H	-1.135893	-2.033217	1.101436
H	-1.710778	-0.255621	-2.196367
H	-2.935034	-1.133764	-1.249234
H	-1.336741	-1.841284	-1.495621
H	1.399794	1.564328	0.606665
H	-0.013635	1.351184	1.629644
H	0.101929	2.863812	-1.042409
H	0.019401	3.601635	0.573688
H	-1.364202	2.708953	-0.062824

Statistical Thermodynamic Analysis
Temperature= 298.150 Kelvin Pressure= 1.00000 Atm

===

SCF Energy= -370.843938747 Predicted Change= -3.133830D-08
Zero-point correction (ZPE)= -370.5788 0.26513
Internal Energy (U)= -370.5668 0.27709
Enthalpy (H)= -370.5658 0.27804
Gibbs Free Energy (G)= -370.6154 0.22845

Frequencies -- 59.1858 78.9225 119.5055

183.15K thermal correction = 0.245883
Single point SCF = -370.964021

SM: 4′-bromo-2,2,2-trifluoroacetophenone

Using Gaussian 09: AM64L-G09RevD.01 24-Apr-2013

===

#m062X/6-31G(d) scf=(direct,tight,maxcycle=300,xqc)
SCRF=(PCM,SOLVENT=CH2Cl2) opt=(maxcycle=250,gdiis) iop(1/8=18)
freq=noraman temp=298.15
#N Geom=AllCheck Guess=TCheck SCRF=Check Test GenChk RM062X/6-31G(d) Freq

----

Pointgroup= C1 Stoichiometry= C8H4BrF3O C1[X(C8H4BrF3O)] #Atoms= 17
Charge = 0 Multiplicity = 1

SCF Energy= -3253.53921686 Predicted Change= -8.971030D-10

===

Optimization completed. {Found 2 times}
Item Max Val. Criteria Pass? RMS Val. Criteria Pass?
Force 0.00001 || 0.00045 [YES] 0.00000 || 0.00030 [YES]
Displ 0.00029 || 0.00180 [YES] 0.00029 || 0.00180 [YES]

Atomic Coordinates (Angstroms)

Type	X	Y	Z
C	-0.851525	0.487350	-0.000018
C	-2.303281	0.784451	-0.000039
O	-2.767681	1.899333	-0.000129
C	-3.284834	-0.409566	0.000031

F	-4.542343	0.009912	0.000099
F	-3.093733	-1.176350	1.081920
F	-3.093856	-1.176363	-1.081875
C	0.025072	1.578451	0.000046
C	1.396855	1.378392	0.000057
C	1.884013	0.072853	-0.000004
Br	3.750092	-0.214287	0.000007
C	1.030814	-1.026163	-0.000073
C	-0.342688	-0.816307	-0.000077
H	-0.384395	2.583202	0.000088
H	2.082348	2.218012	0.000112
H	1.435680	-2.031533	-0.000124
H	-1.002572	-1.675852	-0.000130

Statistical Thermodynamic Analysis
Temperature= 298.150 Kelvin Pressure= 1.00000 Atm
==
==============================
SCF Energy= -3253.53921686 Predicted Change= -8.971030D-10
Zero-point correction (ZPE)= -3253.4321 0.10704
Internal Energy (U)= -3253.4211 0.11805
Enthalpy (H)= -3253.4202 0.11900
Gibbs Free Energy (G)= -3253.4714 0.06779

Frequencies -- 38.5459 70.3361 89.3870

183.15K thermal correction = 0.086010
Single point SCF = -3256.175110

TS-A: HBTM addition to phenylacetic anhydride (Re-face)

Using Gaussian 09: AM64L-G09RevD.01 24-Apr-2013
==
==============================
#m062x/6-31G(d) scf=(maxcycle=300,direct,tight) density=current
SCRF=(PCM,SOLVENT=CH2Cl2) opt=(maxcycle=250,ts,calcfc,noeigentest,gdiis)
iop(1/8=18) freq=noraman temp=298.15
#N Geom=AllCheck Guess=TCheck SCRF=Check Test GenChk RM062X/6-31G(d) Freq
----
Pointgroup= C1 Stoichiometry= C35H34N2O3S C1[X(C35H34N2O3S)] #Atoms= 75
Charge = 0 Multiplicity = 1

SCF Energy= -2086.88262490 Predicted Change= -1.992655D-09
==
==============================
Optimization completed. {Found 1 times}
Item Max Val. Criteria Pass? RMS Val. Criteria Pass?
Force 0.00000 || 0.00045 [YES] 0.00000 || 0.00030 [YES]
Displ 0.00219 || 0.00180 [NO] 0.00219 || 0.00180 [YES]

Atomic Type	Coordinates (Angstroms) X	Y	Z
C	6.626453	0.092023	-0.225795
C	5.414762	-0.566274	-0.024045
C	4.240543	0.144412	-0.251540
C	4.272367	1.480049	-0.658200
C	5.481728	2.130563	-0.862864
C	6.663221	1.423504	-0.641774
N	2.937635	-0.331765	-0.108110
C	1.955244	0.588265	-0.338030
S	2.649005	2.133805	-0.877561
C	2.627297	-1.724122	0.200763
C	1.192845	-2.038215	-0.215043
C	0.776343	-3.460420	0.195047
C	-0.574623	-3.827920	-0.422104
C	1.824035	-4.497769	-0.221977
C	0.252882	-0.932088	0.323271
C	0.090706	-0.945250	1.836362
N	0.698158	0.378199	-0.153767
C	1.020958	-0.337953	2.685299
C	0.850883	-0.378673	4.067131
C	-0.254965	-1.020866	4.619910
C	-1.194061	-1.617393	3.781896
C	-1.022469	-1.575360	2.400291
C	-0.518677	1.714800	-0.639825
C	-1.000206	2.116875	0.752101
C	-2.191649	3.042366	0.667314
O	-1.550571	0.860216	-1.185755
C	-1.339301	0.144245	-2.306244
C	-2.648408	-0.433854	-2.813422
C	-3.193444	-1.520003	-1.904436
O	-0.262555	-0.047122	-2.813521
O	0.046056	2.500619	-1.394264
C	-2.078846	4.302711	0.071521
C	-3.182455	5.146538	-0.011764
C	-4.415957	4.742738	0.498815
C	-4.537713	3.489206	1.092792
C	-3.430772	2.646133	1.174272
C	-3.612530	-1.232086	-0.599857
C	-4.129023	-2.238737	0.212817
C	-4.237329	-3.544661	-0.265384
C	-3.825911	-3.837062	-1.562687
C	-3.305551	-2.830009	-2.373910
H	7.553920	-0.443558	-0.052286
H	5.390723	-1.598768	0.307739
H	5.504354	3.166652	-1.185037
H	7.617632	1.916433	-0.792446
H	2.782689	-1.911903	1.272028
H	3.330747	-2.341382	-0.363367
H	-0.869232	-4.839863	-0.125956
H	-0.511347	-3.803397	-1.517082
H	-1.383318	-3.152573	-0.127447
H	0.685874	-3.486571	1.289672
H	2.768810	-4.379537	0.316751
H	2.030758	-4.429140	-1.297149
H	1.454010	-5.506798	-0.016934
H	1.139880	-1.965687	-1.311210
H	-0.738829	-1.089953	-0.113110
H	1.876660	0.189950	2.270866
H	1.580984	0.100035	4.712540
H	-0.388942	-1.047011	5.696783
H	-2.067244	-2.107387	4.201947
H	-1.768937	-2.024748	1.748676
H	-0.150879	2.606934	1.240258
H	-1.259560	1.226514	1.330090
H	-2.464361	-0.833527	-3.812032
H	-3.369442	0.387110	-2.889582
H	-1.119723	4.609674	-0.335351
H	-3.079695	6.123261	-0.475436
H	-5.275791	5.402755	0.435726
H	-5.493537	3.165400	1.493912
H	-3.526541	1.669707	1.644542
H	-3.536226	-0.214075	-0.227180
H	-4.454174	-1.999464	1.221578
H	-4.642920	-4.326198	0.369653
H	-3.907076	-4.849464	-1.946710
H	-2.985591	-3.063319	-3.386220

Statistical Thermodynamic Analysis
Temperature= 298.150 Kelvin Pressure= 1.00000 Atm

==
==============================

SCF Energy= -2086.88262490 Predicted Change= -1.992655D-09
Zero-point correction (ZPE)= -2086.2603 0.62226
Internal Energy (U)= -2086.2253 0.65727
Enthalpy (H)= -2086.2244 0.65822
Gibbs Free Energy (G)= -2086.3297 0.55288

Frequencies -- -152.9058 10.4007 14.7433

183.15K thermal correction = 0.588518
Single point SCF = -2087.457250

B: Acylated-HBTM phenylacetate complex

Using Gaussian 09: AM64L-G09RevD.01 24-Apr-2013

==
==============================

#m062x/6-31G(d) gfprint gfinput scf=(direct,tight,maxcycle=300,xqc)
#opt=(maxcycle=250,gdiis) iop(1/8=18) freq=noraman temp=298.15
SCRF=(PCM,SOLVENT=dichloromethane)
#N Geom=AllCheck Guess=TCheck SCRF=Check Test GenChk RM062X/6-31G(d) Freq

----

Pointgroup= C1 Stoichiometry= C35H34N2O3S C1[X(C35H34N2O3S)] #Atoms= 75
Charge = 0 Multiplicity = 1

SCF Energy= -2086.89719461 Predicted Change= -1.388801D-08

==
==============================

Optimization completed. {Found 2 times}
Item Max Val. Criteria Pass? RMS Val. Criteria Pass?
Force 0.00002 || 0.00045 [YES] 0.00000 || 0.00030 [YES]
Displ 0.00110 || 0.00180 [YES] 0.00110 || 0.00180 [YES]

Atomic Type	Coordinates (Angstroms) X	Y	Z
C	4.465086	2.889723	1.173290
C	3.351356	2.108725	1.451073
C	2.477599	1.838399	0.400026
C	2.694736	2.347714	-0.876600
C	3.813975	3.129216	-1.153494
C	4.699028	3.390382	-0.114755
N	1.333080	1.035460	0.451659
C	0.665259	0.958222	-0.703988
S	1.423314	1.863848	-1.981816
C	0.988656	0.268313	1.656300
C	0.049211	-0.866800	1.289002
C	-0.459130	-1.623181	2.529806
C	-1.196127	-2.900195	2.115387
C	0.694149	-1.987179	3.470538
C	-1.086646	-0.325080	0.398807
C	-2.009670	0.667366	1.090957
N	-0.488294	0.278608	-0.818066
C	-1.781767	2.046275	1.075790
C	-2.650195	2.916552	1.731292
C	-3.759783	2.421343	2.409719
C	-3.996705	1.048890	2.426771
C	-3.129233	0.181856	1.771386
C	-1.111820	0.195689	-2.101676
C	-2.245316	-0.779885	-2.280076
C	-3.531618	-0.374032	-1.590085
O	0.644231	-1.660816	-2.585026
C	0.601814	-2.596115	-1.750417
C	1.923519	-3.375691	-1.534854
C	2.837920	-2.584282	-0.633754
O	-0.373088	-2.939742	-1.033652
O	-0.738626	0.945742	-2.975708
C	-4.336332	-1.352258	-1.003590
C	-5.533436	-1.005363	-0.380451
C	-5.928362	0.328636	-0.322986
C	-5.126956	1.312219	-0.899972
C	-3.940748	0.961274	-1.537145
C	3.409156	-1.385124	-1.083679
C	4.256261	-0.649068	-0.261186
C	4.526240	-1.080473	1.039583
C	3.946979	-2.258002	1.504310
C	3.112448	-3.004467	0.669716
H	5.166898	3.111854	1.969919
H	3.177686	1.714679	2.446198
H	3.987505	3.519262	-2.150700
H	5.580193	3.993557	-0.304834
H	0.549131	0.954573	2.391091
H	1.931389	-0.126541	2.043619
H	-1.518934	-3.453041	3.002904
H	-0.537798	-3.536498	1.515191
H	-2.082569	-2.702647	1.504550
H	-1.148833	-0.968720	3.080026
H	0.322732	-2.606045	4.292720
H	1.168265	-1.105930	3.914024
H	1.462865	-2.557251	2.936452
H	0.601905	-1.581212	0.667097
H	-1.649831	-1.188712	0.047921
H	-0.937859	2.466809	0.534957
H	-2.458877	3.984817	1.703026
H	-4.440386	3.100853	2.913312
H	-4.870189	0.650338	2.933952
H	-3.341237	-0.883714	1.764575
H	-1.892375	-1.760517	-1.940550
H	-2.391786	-0.824629	-3.363450
H	2.401064	-3.521935	-2.508992
H	1.709192	-4.350441	-1.090468
H	-4.018576	-2.392227	-1.034578
H	-6.151004	-1.777279	0.069262
H	-6.853546	0.602809	0.174992
H	-5.424612	2.355443	-0.852077
H	-3.319213	1.732344	-1.987866
H	3.179342	-1.045615	-2.090187
H	4.707007	0.269946	-0.629498
H	5.184743	-0.500471	1.679898
H	4.149888	-2.605249	2.513908
H	2.673847	-3.931729	1.032335

Statistical Thermodynamic Analysis
Temperature= 298.150 Kelvin Pressure= 1.00000 Atm

==
==============================

SCF Energy= -2086.89719461 Predicted Change= -1.388801D-08
Zero-point correction (ZPE)= -2086.2739 0.62324
Internal Energy (U)= -2086.2384 0.65874
Enthalpy (H)= -2086.2375 0.65968
Gibbs Free Energy (G)= -2086.3411 0.55605

Frequencies -- 19.3532 29.3641 32.4508

183.15K thermal correction = 0.590951
Single point SCF = -2087.477456

TS-C: Acylated-HBTM deprotonation *via* phenylacetate

Using Gaussian 09: AM64L-G09RevD.01 24-Apr-2013
==
==============================
#m062x/6-31G(d) scf=(maxcycle=300,direct,tight)
density=current
opt=(maxcycle=250,ts,calcfc,noeigentest,gdiis) iop(1/8=18)
freq=noraman
temp=298.15 SCRF=(PCM,SOLVENT=dichloromethane)
#N Geom=AllCheck Guess=TCheck SCRF=Check Test GenChk
RM062X/6-31G(d) Freq

Pointgroup= C1 Stoichiometry= C35H34N2O3S
C1[X(C35H34N2O3S)] #Atoms= 75
Charge = 0 Multiplicity = 1

SCF Energy= -2086.87328740 Predicted Change= -1.338516D-09
==
==============================
Optimization completed. {Found 2 times}

Item	Max Val.	Criteria	Pass?	RMS Val.	Criteria	Pass?
Force	0.00000 ||	0.00045	[YES]	0.00000 ||	0.00030	[YES]
Displ	0.00103 ||	0.00180	[YES]	0.00103 ||	0.00180	[YES]

Atomic Type	X	Y	Z
C	5.042963	-0.400431	-2.370533
C	4.126619	0.303295	-1.599043
C	2.797924	-0.112904	-1.639068
C	2.390881	-1.178621	-2.435798
C	3.312096	-1.882460	-3.207103
C	4.643519	-1.484590	-3.162244
N	1.733485	0.422288	-0.904090
C	0.549833	-0.167452	-1.135682
S	0.664506	-1.469745	-2.290797
C	1.948150	1.451276	0.118897
C	0.805772	1.413943	1.118205
C	0.891490	2.540160	2.165160
C	-0.065388	2.261557	3.327920
C	2.314249	2.705469	2.707296
C	-0.540066	1.397567	0.372141
C	-0.886676	2.677301	-0.366388
N	-0.577731	0.236195	-0.543899
C	-0.402070	2.945119	-1.649644
C	-0.710117	4.144727	-2.286131
C	-1.510462	5.090264	-1.649784
C	-2.007756	4.827299	-0.375777
C	-1.700354	3.626246	0.258545
C	-1.835302	-0.455486	-0.845273
C	-2.917274	-0.179522	0.060347
C	-4.188410	-0.907170	-0.160979
O	-1.787872	-1.277004	-1.747715
C	-4.202694	-2.298590	-0.350303
C	-5.402790	-2.984696	-0.501101
C	-6.619892	-2.303957	-0.455109
C	-6.620296	-0.925999	-0.252359
C	-5.417758	-0.237759	-0.108792
H	6.085748	-0.102145	-2.356777
H	4.439229	1.140694	-0.985315
H	2.998161	-2.719938	-3.821187
H	5.380560	-2.019418	-3.751424
H	2.042016	2.429109	-0.370223
H	2.890933	1.203119	0.612617
H	0.006113	3.056897	4.075605
H	0.196878	1.312785	3.811747
H	-1.111928	2.192452	3.017571
H	0.602704	3.482746	1.681354
H	2.324157	3.456717	3.502504
H	3.018525	3.033889	1.936569
H	2.680044	1.761661	3.126899
H	0.868280	0.453245	1.648092
H	-1.295865	1.200507	1.128257
H	0.207778	2.213857	-2.174531
H	-0.327355	4.335624	-3.283736
H	-1.752255	6.023477	-2.148429
H	-2.643175	5.552300	0.123021
H	-2.101375	3.421433	1.248852
H	-3.055711	0.875461	0.297595
H	-2.259547	-0.675436	1.239869
H	-3.255526	-2.829805	-0.365009
H	-5.389803	-4.061326	-0.647821
H	-7.555906	-2.841906	-0.571558
H	-7.559648	-0.381575	-0.209518
H	-5.427945	0.839110	0.044876
O	-0.827365	-2.564617	0.811399
C	-0.773879	-1.939247	1.868215
C	0.295001	-2.281496	2.907115
C	1.684856	-1.914779	2.427049
O	-1.560309	-0.957793	2.194631
C	2.171341	-2.377452	1.196443
C	3.452454	-2.038185	0.769530
C	4.266918	-1.223200	1.557649
C	3.792786	-0.761427	2.782693
C	2.510881	-1.107293	3.212590
H	0.240370	-3.362781	3.077115
H	0.074913	-1.771946	3.847594
H	1.531868	-3.003787	0.581700
H	3.820217	-2.407759	-0.184875
H	5.263841	-0.957344	1.218265
H	4.419645	-0.133275	3.409385
H	2.146750	-0.743847	4.170983

Statistical Thermodynamic Analysis
Temperature= 298.150 Kelvin Pressure= 1.00000 Atm
==
==============================
SCF Energy= -2086.87328740 Predicted Change= -1.338516D-09
Zero-point correction (ZPE)= -2086.2546 0.61859
Internal Energy (U)= -2086.2194 0.65383
Enthalpy (H)= -2086.2185 0.65478
Gibbs Free Energy (G)= -2086.3226 0.55066

Frequencies -- -1172.8565 20.1902 28.3632

183.15K thermal correction = 0.585794
Single point SCF = -2087.453409

***N,N*-diisopropylethylamine/phenylacetic acid complex**

Using Gaussian 09: AM64L-G09RevD.01 24-Apr-2013
==
==============================
#m062X/6-31G(d) scf=(direct,tight,maxcycle=300,xqc)

SCRF=(PCM,SOLVENT=CH2Cl2) opt=(maxcycle=250,gdiis) iop(1/8=18)
freq=noraman temp=298.15
#N Geom=AllCheck Guess=TCheck SCRF=Check Test GenChk RM062X/6-31G(d) Freq
----
Pointgroup= C1 Stoichiometry= C16H27NO2
C1[X(C16H27NO2)] #Atoms= 46
Charge = 0 Multiplicity = 1

SCF Energy= -830.814674709 Predicted Change= -1.147318D-08
==
Optimization completed. {Found 1 times}
Item Max Val. Criteria Pass? RMS Val. Criteria Pass?
Force 0.00001 || 0.00045 [YES] 0.00000 || 0.00030 [YES]
Displ 0.00700 || 0.00180 [NO] 0.00700 || 0.00180 [NO]

Atomic Type	Coordinates (Angstroms) X	Y	Z
N	2.642650	0.008434	-0.043499
C	2.908147	-0.938196	-1.161426
C	2.839656	-2.376880	-0.643626
C	1.909702	-0.770591	-2.305673
C	2.373808	1.406140	-0.478328
C	1.918476	2.253139	0.708400
C	3.539509	2.058149	-1.224225
C	3.702143	-0.076119	0.994512
C	3.172048	-0.594705	2.326394
H	1.831328	-2.598345	-0.277959
H	3.548382	-2.567369	0.166529
H	3.072454	-3.070717	-1.456418
H	3.924087	-0.756993	-1.549692
H	2.045490	-1.587711	-3.019754
H	2.043076	0.168322	-2.848459
H	0.881662	-0.805280	-1.929316
H	1.513348	1.340274	-1.147252
H	2.723612	2.436714	1.427329
H	1.087032	1.769894	1.229775
H	1.572182	3.225423	0.345973
H	4.420552	2.170816	-0.583059
H	3.832760	1.479463	-2.105661
H	3.246801	3.056836	-1.562439
H	4.512539	-0.718647	0.633772
H	4.152553	0.909093	1.142656
H	2.731146	-1.589774	2.214114
H	3.980428	-0.652553	3.061487
H	2.395397	0.070265	2.716615
C	-3.579215	0.974356	0.586966
C	-4.759139	1.466150	0.039487
C	-5.640478	0.603909	-0.613087
C	-5.331188	-0.749209	-0.713776
C	-4.146288	-1.237435	-0.163742
C	-3.259731	-0.383577	0.491960
C	-1.985418	-0.907282	1.097821
C	-0.717350	-0.339917	0.473944
O	0.366622	-0.875387	1.008189
O	-0.702182	0.507882	-0.398311
H	-2.890247	1.648703	1.089103
H	-4.992954	2.523478	0.121184
H	-6.562966	0.986825	-1.038793
H	-6.011140	-1.428473	-1.219313
H	-3.908894	-2.295477	-0.243763
H	1.232935	-0.487401	0.573264
H	-1.929056	-1.998248	1.022908
H	-1.934678	-0.675708	2.169035

Statistical Thermodynamic Analysis
Temperature= 298.150 Kelvin Pressure= 1.00000 Atm
==
SCF Energy= -830.814674709 Predicted Change= -1.147318D-08
Zero-point correction (ZPE)= -830.4022 0.41244
Internal Energy (U)= -830.3806 0.43403
Enthalpy (H)= -830.3796 0.43498
Gibbs Free Energy (G)= -830.4554 0.35927

Frequencies -- 9.0284 16.1266 38.9416

183.15K thermal correction = 0.385493
Single point SCF = -831.093076

D: (Z)-enolate/ketone complex

Using Gaussian 09: AM64L-G09RevD.01 24-Apr-2013
==
#m062x/6-31G(d) gfprint gfinput scf=(direct,tight,maxcycle=300,xqc)
opt=(maxcycle=250,gdiis) iop(1/8=18) freq=noraman temp=298.15
SCRF=(PCM,SOLVENT=dichloromethane)
#N Geom=AllCheck Guess=TCheck SCRF=Check Test GenChk RM062X/6-31G(d) Freq
----
Pointgroup= C1 Stoichiometry= C35H30BrF3N2O2S
C1[X(C35H30BrF3N2O2S)] #Atoms= 74
Charge = 0 Multiplicity = 1

SCF Energy= -4880.47694907 Predicted Change= -5.469429D-09
==
Optimization completed. {Found 2 times}
Item Max Val. Criteria Pass? RMS Val. Criteria Pass?
Force 0.00000 || 0.00045 [YES] 0.00000 || 0.00030 [YES]
Displ 0.00155 || 0.00180 [YES] 0.00155 || 0.00180 [YES]

Atomic Type	Coordinates (Angstroms) X	Y	Z
N	3.412260	1.201882	0.014712
C	4.126398	0.036733	-0.503874
C	3.127002	-0.971180	-1.047486
C	3.828308	-2.264300	-1.509123
C	5.049074	-1.964751	-2.386110
C	2.880039	-3.184770	-2.284014
C	2.027182	-1.228869	0.005311
C	2.486773	-2.028087	1.211955
N	1.414396	0.044515	0.432104
C	0.012107	0.093041	0.923000
C	-0.673697	-1.100376	0.984402
C	-2.064191	-1.211387	1.395109
O	-0.377604	1.251952	1.195466
C	2.120499	1.165811	0.399195
S	1.525404	2.743413	0.879299
C	3.118676	3.428631	0.559916
C	4.014719	2.463195	0.109100

C	3.520368	4.750624	0.718082
C	4.838926	5.080420	0.419430
C	5.735857	4.103558	-0.023841
C	5.339046	2.779879	-0.184415
C	3.137659	-1.414174	2.285200
C	3.593155	-2.170538	3.362374
C	3.399110	-3.549525	3.381704
C	2.741206	-4.167905	2.320811
C	2.286257	-3.410157	1.245532
C	-2.817660	-0.160058	1.956027
C	-4.152432	-0.341200	2.302283
C	-4.790534	-1.563837	2.098365
C	-4.058609	-2.619659	1.554160
C	-2.723824	-2.445263	1.210507
H	4.749812	-0.394229	0.290120
H	4.776384	0.394712	-1.303731
H	4.178540	-2.792780	-0.613225
H	5.450219	-2.898120	-2.791975
H	5.856473	-1.477185	-1.832376
H	4.773397	-1.324059	-3.232497
H	3.356992	-4.155559	-2.447692
H	2.649751	-2.751768	-3.262880
H	1.928394	-3.361766	-1.774825
H	2.624419	-0.511642	-1.909880
H	1.230221	-1.771793	-0.504389
H	-0.183475	-2.034657	0.753845
H	2.821201	5.503146	1.068308
H	5.174133	6.105454	0.535633
H	6.762117	4.376392	-0.245441
H	6.043985	2.026308	-0.517719
H	3.277779	-0.335579	2.297266
H	4.093542	-1.678037	4.190107
H	3.748689	-4.137822	4.224194
H	2.572784	-5.240119	2.333058
H	1.763284	-3.898469	0.426627
H	-2.341445	0.799966	2.108098
H	-4.710715	0.495174	2.716883
H	-5.836631	-1.692505	2.360730
H	-4.532017	-3.584564	1.391346
H	-2.168562	-3.274302	0.775352
C	-0.883238	0.105030	-1.970695
C	-2.243382	0.404118	-1.487212
C	-0.644184	-1.262211	-2.657744
F	-1.446946	-1.394036	-3.721656
F	-0.873636	-2.305660	-1.847379
F	0.617217	-1.352570	-3.080197
O	0.048084	0.882860	-1.959527
C	-3.280061	-0.537755	-1.523108
C	-4.539566	-0.204936	-1.047584
C	-4.749053	1.070963	-0.536583
Br	-6.443602	1.490085	0.192280
C	-3.737274	2.023902	-0.493528
C	-2.477526	1.680364	-0.964116
H	-3.115457	-1.539293	-1.903581
H	-5.341521	-0.933926	-1.043467
H	-3.927800	3.006365	-0.076425
H	-1.657807	2.388208	-0.920371

Statistical Thermodynamic Analysis
Temperature= 298.150 Kelvin Pressure= 1.00000 Atm
==
==============================
SCF Energy= -4880.47694907 Predicted Change= -5.469429D-09
Zero-point correction (ZPE)= -4879.8927 0.58424
Internal Energy (U)= -4879.8542 0.62265
Enthalpy (H)= -4879.8533 0.62359
Gibbs Free Energy (G)= -4879.9647 0.51224

Frequencies -- 13.9754 24.0653 30.7936

183.15K thermal correction = 0.549746
Single point SCF = -4883.532648

TS-I-(Re,Si): C–C bond formation

Using Gaussian 09: AM64L-G09RevD.01 24-Apr-2013
==
==============================
#m062x/6-31G(d) scf=(maxcycle=300,direct,tight) density=current
SCRF=(PCM,SOLVENT=CH2Cl2) opt=(maxcycle=250,ts,calcfc,noeigentest,gdiis)
iop(1/8=18) freq=noraman temp=298.15
#N Geom=AllCheck Guess=TCheck SCRF=Check Test GenChk RM062X/6-31G(d) Freq
----
Pointgroup= C1 Stoichiometry= C35H30BrF3N2O2S C1[X(C35H30BrF3N2O2S)] #Atoms= 74
Charge = 0 Multiplicity = 1

SCF Energy= -4880.46632468 Predicted Change= -1.238786D-08
==
==============================
Optimization completed. {Found 2 times}
Item Max Val. Criteria Pass? RMS Val. Criteria Pass?
Force 0.00000 || 0.00045 [YES] 0.00000 || 0.00030 [YES]
Displ 0.00179 || 0.00180 [YES] 0.00179 || 0.00180 [YES]

Atomic Type	Coordinates (Angstroms) X	Y	Z
N	3.274695	1.527402	-0.108654
C	4.021058	0.523256	-0.873369
C	3.046749	-0.462357	-1.492459
C	3.774389	-1.600044	-2.234514
C	4.903298	-1.063003	-3.120503
C	2.795473	-2.401436	-3.095872
C	2.073246	-0.973752	-0.414819
C	2.692373	-1.857449	0.649667
N	1.371694	0.185926	0.194285
C	0.032306	0.063117	0.730687
C	-0.618300	-1.216039	0.612356
C	-1.697228	-1.528684	1.580710
O	-0.465009	1.089366	1.179939
C	2.015002	1.356109	0.317353
S	1.363019	2.792779	1.065655
C	2.891857	3.617586	0.803024
C	3.812822	2.791494	0.165985
C	3.228581	4.922540	1.153962
C	4.509968	5.369443	0.855291
C	5.435301	4.529464	0.224282
C	5.102619	3.227747	-0.129471
C	3.384702	-1.336465	1.745736
C	3.967127	-2.187212	2.682344
C	3.859272	-3.568125	2.536514
C	3.156767	-4.096026	1.455635
C	2.573031	-3.244526	0.521554
C	-2.661687	-0.606506	2.018154
C	-3.650207	-0.986574	2.923812
C	-3.709805	-2.289573	3.410064
C	-2.764249	-3.219387	2.977497
C	-1.775790	-2.840418	2.078341

H	4.741397	0.028991	-0.209785
H	4.566932	1.067055	-1.645802
H	4.220231	-2.267080	-1.484631
H	5.330702	-1.878931	-3.710394
H	5.717917	-0.618140	-2.540876
H	4.524122	-0.308190	-3.820092
H	3.315807	-3.230543	-3.584481
H	2.371288	-1.759674	-3.876661
H	1.961041	-2.815696	-2.526426
H	2.425560	0.081071	-2.218212
H	1.301470	-1.515263	-0.953125
H	0.064633	-2.053173	0.506264
H	2.509947	5.568912	1.646535
H	4.796206	6.381828	1.118731
H	6.432693	4.896797	0.008351
H	5.827363	2.577006	-0.605460
H	3.460555	-0.260969	1.890475
H	4.498897	-1.767797	3.530600
H	4.309984	-4.230296	3.268926
H	3.053035	-5.170708	1.343489
H	2.004431	-3.657213	-0.309295
H	-2.640113	0.409417	1.641995
H	-4.384842	-0.253205	3.245221
H	-4.484057	-2.579986	4.113925
H	-2.794989	-4.241419	3.344183
H	-1.042487	-3.571731	1.744853
C	-1.202249	-0.997146	-1.347092
C	-2.472025	-0.204535	-1.148237
C	-1.383856	-2.499323	-1.642227
F	-1.931896	-2.655616	-2.858646
F	-2.150358	-3.186363	-0.780297
F	-0.191225	-3.128215	-1.665452
O	-0.270124	-0.481484	-2.024806
C	-3.727771	-0.748757	-0.863993
C	-4.832294	0.077263	-0.671854
C	-4.668924	1.453768	-0.746925
Br	-6.163289	2.587450	-0.444139
C	-3.432795	2.022317	-1.035319
C	-2.344458	1.184932	-1.244653
H	-3.865910	-1.820029	-0.779147
H	-5.805566	-0.347729	-0.451908
H	-3.327602	3.099921	-1.098325
H	-1.370034	1.596763	-1.485442

Statistical Thermodynamic Analysis
Temperature= 298.150 Kelvin Pressure= 1.00000 Atm
==
==============================
SCF Energy= -4880.46632468 Predicted Change= -1.238786D-08
Zero-point correction (ZPE)= -4879.8821 0.58415
Internal Energy (U)= -4879.8447 0.62160
Enthalpy (H)= -4879.8437 0.62254
Gibbs Free Energy (G)= -4879.9536 0.51267

Frequencies -- -209.1648 14.3232 16.5788

183.15K thermal correction = 0.549715
Single point SCF = -4883.521485

TS-I-(Re,Re): C–C bond formation

Using Gaussian 09: AM64L-G09RevD.01 24-Apr-2013
==
==============================
# m062x/6-31G(d) scf=(maxcycle=300,direct,tight,xqc) density=current
opt=(maxcycle=250,modredundant)
SCRF=(PCM,SOLVENT=CH2Cl2) iop(1/8=18)
Temperature=298.15
Modredundant Input: B 11 58 F
Modredundant Input:
# m062x/6-31G(d) scf=(direct,tight,maxcycle=300,xqc)
opt=(nofreeze,maxcycle=250,ts,calcfc,noeigentest) iop(1/8=18) freq=noraman
SCRF=(PCM,SOLVENT=CH2Cl2) Temperature=298.15 geom=check guess=read
#N Geom=AllCheck Guess=TCheck SCRF=Check Test GenChk RM062X/6-31G(d) Freq

----
Pointgroup= C1 Stoichiometry= C35H30BrF3N2O2S
C1[X(C35H30BrF3N2O2S)] #Atoms= 74
Charge = 0 Multiplicity = 1

SCF Energy= -4880.46368920 Predicted Change= -1.770657D-09
==
==============================
Optimization completed on the basis of negligible forces.
{Found 3 times}

Item	Max Val.	Criteria	Pass?	RMS Val.	Criteria	Pass?
Force	0.00000 ||	0.00045	[YES]	0.00000 ||	0.00030	[YES]
Displ	0.00733 ||	0.00180	[NO]	0.00733 ||	0.00180	[YES]

Atomic Type	Coordinates (Angstroms) X	Y	Z
N	-4.003677	0.063142	-0.287097
C	-3.902247	1.428396	-0.814645
C	-2.465511	1.708083	-1.216121
C	-2.263292	3.164357	-1.673716
C	-3.359330	3.605680	-2.649017
C	-0.895291	3.341143	-2.339123
C	-1.520437	1.310168	-0.070843
C	-1.603714	2.161988	1.179390
N	-1.724343	-0.126423	0.247366
C	-0.645304	-0.960121	0.747426
C	0.695937	-0.433433	0.667427
H	0.752442	0.650473	0.667900
C	1.733627	-1.005692	1.561688
O	-0.963158	-2.081797	1.123047
C	-2.955398	-0.648299	0.155242
S	-3.394553	-2.279511	0.597962
C	-5.062440	-1.943822	0.159537
C	-5.219705	-0.633433	-0.282036
C	-6.151281	-2.808574	0.229077
C	-7.396772	-2.323189	-0.150921
C	-7.550044	-1.001334	-0.585856
C	-6.465951	-0.135329	-0.656995
C	-2.590467	1.983557	2.153642
C	-2.642816	2.819308	3.266728
C	-1.707228	3.839484	3.422875
C	-0.711511	4.015654	2.465007
C	-0.659887	3.178215	1.354305
C	1.825664	-2.359129	1.922599
C	2.878198	-2.813125	2.714076
C	3.868511	-1.940142	3.158163
C	3.790126	-0.593286	2.807743
C	2.734698	-0.136871	2.027758
H	-4.260839	2.132551	-0.053304
H	-4.564160	1.476801	-1.680718
H	-2.316440	3.810224	-0.786822
H	-3.131435	4.602133	-3.038645
H	-4.345668	3.657142	-2.177917

H	-3.419018	2.918966	-3.502130
H	-0.064407	3.007859	-1.711960
H	-0.734789	4.393530	-2.591844
H	-0.849519	2.754585	-3.263507
H	-2.203010	1.044817	-2.052473
H	-0.525122	1.368616	-0.500401
H	-6.027586	-3.830235	0.571880
H	-8.260924	-2.977048	-0.107171
H	-8.532277	-0.641403	-0.872090
H	-6.593376	0.890247	-0.984735
H	-3.319495	1.181187	2.063648
H	-3.413187	2.667638	4.016066
H	-1.747417	4.486691	4.293165
H	0.031236	4.797729	2.585850
H	0.125550	3.310996	0.613377
H	1.073362	-3.055792	1.578412
H	2.924792	-3.865452	2.980783
H	4.689439	-2.302799	3.769377
H	4.551933	0.104809	3.143432
H	2.685853	0.915494	1.755909
C	1.133847	-0.514344	-1.312907
C	2.632346	-0.332783	-1.183694
C	0.676466	-1.939097	-1.678897
F	1.166381	-2.271939	-2.887688
F	-0.660617	-2.001191	-1.766194
F	1.055725	-2.909743	-0.829982
O	0.471430	0.378309	-1.913300
C	3.098982	0.981394	-1.243267
C	4.443455	1.276561	-1.048807
C	5.327763	0.231251	-0.807745
Br	7.162260	0.614166	-0.501566
C	4.896771	-1.088730	-0.775602
C	3.545566	-1.366631	-0.960929
H	2.382105	1.773463	-1.438574
H	4.801663	2.299868	-1.081670
H	5.604750	-1.889801	-0.593403
H	3.215863	-2.397457	-0.909470

Statistical Thermodynamic Analysis
Temperature= 298.150 Kelvin Pressure= 1.00000 Atm
===
==============================
SCF Energy= -4880.46368920 Predicted Change= -1.770657D-09
Zero-point correction (ZPE)= -4879.8796 0.58407
Internal Energy (U)= -4879.8420 0.62164
Enthalpy (H)= -4879.8411 0.62258
Gibbs Free Energy (G)= -4879.9520 0.51166

Frequencies -- -216.9053 6.7705 15.6703

183.15K thermal correction = 0.549101
Single point SCF = -4883.519764

TS-I-(Si,Re): C–C bond formation

Using Gaussian 09: AM64L-G09RevD.01 24-Apr-2013
===
==============================
# m062x/6-31G(d) gfprint gfinput scf=(direct,tight,maxcycle=300,xqc)
opt=(maxcycle=250,calcfc,ts,noeigentest) freq=noraman
SCRF=(PCM,SOLVENT=Dichloromethane) iop(1/8=18) Temperature=298.15
#N Geom=AllCheck Guess=TCheck SCRF=Check Test GenChk RM062X/6-31G(d) Freq

----

Pointgroup= C1 Stoichiometry= C35H30BrF3N2O2S
C1[X(C35H30BrF3N2O2S)] #Atoms= 74
Charge = 0 Multiplicity = 1

SCF Energy= -4880.45816957 Predicted Change= -1.186941D-08
===
==============================
Optimization completed. {Found 1 times}

Item	Max Val.	Criteria	Pass?	RMS Val.	Criteria	Pass?
Force	0.00001 ||	0.00045	[YES]	0.00000 ||	0.00030	[YES]
Displ	0.00226 ||	0.00180	[NO]	0.00226 ||	0.00180	[YES]

Atomic Coordinates (Angstroms)

Type	X	Y	Z
C	4.802021	4.835537	0.326224
H	5.761631	5.296406	0.533581
C	4.746292	3.468874	0.080366
H	5.643142	2.859839	0.101350
C	3.496636	2.913450	-0.180830
C	2.339223	3.689567	-0.186032
C	2.399870	5.058869	0.054802
H	1.500923	5.665440	0.045945
C	3.644376	5.622246	0.313106
H	3.716362	6.686552	0.508717
N	3.221985	1.568602	-0.460753
C	1.921235	1.295522	-0.628382
S	0.924903	2.723204	-0.571358
C	4.287465	0.576531	-0.642478
H	4.679933	0.287401	0.339938
H	5.077317	1.072819	-1.209043
C	3.741990	-0.621453	-1.406866
C	4.791039	-1.740061	-1.547396
H	4.957282	-2.180088	-0.555910
C	6.127545	-1.188299	-2.055634
H	6.608169	-0.524129	-1.331444
H	5.989728	-0.635641	-2.992886
H	6.819393	-2.012159	-2.252225
C	4.299882	-2.832802	-2.501077
H	3.370017	-3.304184	-2.171556
H	5.052889	-3.620894	-2.590470
H	4.128482	-2.415642	-3.500312
H	3.473473	-0.281284	-2.417471
C	2.430687	-1.083761	-0.730380
C	2.635390	-1.636950	0.667992
H	1.982743	-1.855608	-1.359815
N	1.470685	0.045883	-0.774103
C	0.057772	-0.171020	-1.035907
C	-0.513528	-1.385386	-0.495255
H	0.224645	-2.081992	-0.103561
C	-1.571484	-2.071375	-1.279159
O	-0.545332	0.744900	-1.568035
C	2.699333	-0.813598	1.793710
H	2.490025	0.247722	1.711661
C	2.971282	-1.357682	3.045598
H	3.013627	-0.707244	3.913534
C	3.163241	-2.728986	3.190397
H	3.366535	-3.150821	4.169887
C	3.072189	-3.560061	2.076768
H	3.200496	-4.632895	2.181684
C	2.808504	-3.014772	0.823844
H	2.729870	-3.670536	-0.040566
C	-2.519289	-1.398168	-2.066113
H	-2.500319	-0.315412	-2.112038
C	-3.487001	-2.106002	-2.775283
H	-4.209253	-1.560429	-3.376245

C	-3.544735	-3.495678	-2.709180
H	-4.305854	-4.041723	-3.258208
C	-2.614393	-4.177011	-1.925031
H	-2.643470	-5.261028	-1.861090
C	-1.641444	-3.472818	-1.225590
H	-0.919760	-4.012450	-0.617006
C	-2.479777	1.429644	0.711794
H	-1.568095	1.998785	0.855907
C	-2.412488	0.042279	0.863144
C	-1.043161	-0.527297	1.198819
C	-1.097451	-1.790182	2.085172
F	0.125901	-2.271308	2.325913
F	-1.819320	-2.820616	1.595419
F	-1.643695	-1.477816	3.276126
C	-3.584895	-0.705456	0.709674
H	-3.571129	-1.783821	0.810585
C	-4.794961	-0.082134	0.415591
H	-5.702609	-0.665164	0.302345
C	-4.824728	1.297471	0.260516
Br	-6.472658	2.155739	-0.137441
C	-3.675908	2.066835	0.403337
H	-3.721317	3.144325	0.285829
O	-0.142088	0.285446	1.571328

Statistical Thermodynamic Analysis
Temperature= 298.150 Kelvin Pressure= 1.00000 Atm
==
==============================
SCF Energy= -4880.45816957 Predicted Change= -
1.186941D-08
Zero-point correction (ZPE)= -4879.8742 0.58390
Internal Energy (U)= -4879.8368 0.62130
Enthalpy (H)= -4879.8359
0.62224
Gibbs Free Energy (G)= -4879.9444
0.51370

Frequencies -- -211.6955 20.2448 24.2683

183.15K thermal correction = 0.550230
Single point SCF = -4883.515365

TS-I-(Si,Si): C–C bond formation

Using Gaussian 09: AM64L-G09RevD.01 24-Apr-2013
==
==============================
# m062x/6-31G(d) scf=(maxcycle=300,direct,tight,xqc)
density=current
opt=(maxcycle=250,modredundant)
SCRF=(PCM,SOLVENT=CH2Cl2) iop(1/8=18)
Temperature=298.15
Modredundant Input: B 11 58 F
Modredundant Input:
# m062x/6-31G(d) scf=(direct,tight,maxcycle=300,xqc)
opt=(nofreeze,maxcycle=250,ts,calcfc,noeigentest) iop(1/8=18)
freq=noraman
SCRF=(PCM,SOLVENT=CH2Cl2) Temperature=298.15
geom=check guess=read
#N Geom=AllCheck Guess=TCheck SCRF=Check Test GenChk
RM062X/6-31G(d) Freq

Pointgroup= C1 Stoichiometry= C35H30BrF3N2O2S
C1[X(C35H30BrF3N2O2S)] #Atoms= 74
Charge = 0 Multiplicity = 1

SCF Energy= -4880.45830817 Predicted Change= -4.414332D-
08

==
==============================
Optimization completed. {Found 2 times}
Item Max Val. Criteria Pass? RMS Val. Criteria
Pass?
Force 0.00003 || 0.00045 [YES] 0.00000 || 0.00030 [
YES]
Displ 0.00329 || 0.00180 [NO] 0.00329 || 0.00180 [
YES]

Atomic Type	Coordinates (Angstroms) X	Y	Z
N	-1.952354	1.849899	-0.748452
C	-1.539225	3.178019	-0.281814
C	-0.044032	3.365763	-0.502573
C	0.463104	4.665899	0.148982
C	-0.397663	5.863823	-0.265843
C	1.923839	4.933583	-0.222332
C	0.717896	2.113555	-0.018822
C	0.698719	1.919056	1.486849
N	0.205761	0.928683	-0.754565
C	1.119611	-0.097027	-1.268786
C	2.335612	-0.310703	-0.521469
H	2.416410	0.314086	0.362652
C	3.592915	-0.359034	-1.304363
O	0.737590	-0.721461	-2.244367
C	-1.092719	0.874721	-1.076885
S	-1.870863	-0.471080	-1.857417
C	-3.434143	0.269591	-1.561256
C	-3.297131	1.527756	-0.978834
C	-4.692591	-0.246602	-1.856098
C	-5.802917	0.531934	-1.551285
C	-5.658778	1.796895	-0.968169
C	-4.404061	2.315298	-0.671823
C	-0.451135	1.513445	2.173459
C	-0.435036	1.368729	3.557777
C	0.730918	1.627624	4.275991
C	1.880942	2.028241	3.602261
C	1.862465	2.170574	2.216854
C	3.700559	-1.078794	-2.501240
C	4.902218	-1.096980	-3.206704
C	6.018064	-0.414732	-2.728603
C	5.925050	0.292102	-1.529665
C	4.724825	0.318777	-0.828864
H	-1.813623	3.290717	0.773754
H	-2.107588	3.902549	-0.869077
H	0.389577	4.548877	1.238691
H	0.032415	6.786891	0.132579
H	-1.422622	5.793950	0.110114
H	-0.436173	5.955835	-1.357930
H	2.604172	4.153839	0.130860
H	2.255903	5.878546	0.216720
H	2.033502	5.010690	-1.310430
H	0.128526	3.436650	-1.585950
H	1.754499	2.213548	-0.345215
H	-4.800489	-1.230654	-2.299603
H	-6.795297	0.149541	-1.764072
H	-6.540857	2.382988	-0.734657
H	-4.294150	3.289370	-0.207956
H	-1.358304	1.262450	1.630434
H	-1.333325	1.043750	4.073872
H	0.743308	1.509355	5.354648
H	2.797553	2.221225	4.150139
H	2.771281	2.464740	1.696368
H	2.839574	-1.625028	-2.864059
H	4.966825	-1.656762	-4.135728
H	6.952313	-0.434641	-3.281798
H	6.788051	0.826092	-1.141956
H	4.657015	0.868889	0.108054

C	2.116031	-2.204971	0.120338
C	2.943089	-2.057298	1.418120
F	4.246987	-1.941075	1.153184
F	2.786556	-3.164475	2.170055
F	2.615076	-1.016140	2.215939
C	0.619323	-2.346793	0.378490
O	2.635687	-2.990261	-0.709120
C	-0.060862	-3.282299	-0.402917
C	-1.433874	-3.462779	-0.276309
C	-2.128926	-2.681135	0.638072
Br	-4.022659	-2.781475	0.691352
C	-1.474935	-1.766860	1.455356
C	-0.097446	-1.608739	1.323868
H	0.513097	-3.854193	-1.123609
H	-1.963880	-4.174210	-0.900842
H	-2.034865	-1.176758	2.174573
H	0.408171	-0.888338	1.956616

Statistical Thermodynamic Analysis
Temperature= 298.150 Kelvin   Pressure= 1.00000 Atm
==
==============================
SCF Energy=   -4880.45830817   Predicted Change= -4.414332D-08
Zero-point correction (ZPE)=   -4879.8746   0.58367
Internal Energy (U)=   -4879.8371   0.62118
Enthalpy (H)=   -4879.8361   0.62212
Gibbs Free Energy (G)=   -4879.9455   0.51274

Frequencies --   -173.0946   11.1301   20.3685

183.15K thermal correction = 0.549589
Single point SCF = -4883.514706

J: Tetrahedral Intermediate

Using Gaussian 09: AM64L-G09RevD.01 24-Apr-2013
==
==============================
#m062x/6-31G(d) gfprint gfinput scf=(direct,tight,maxcycle=300,xqc)
opt=(maxcycle=250,gdiis) iop(1/8=18) freq=noraman temp=298.15
SCRF=(PCM,SOLVENT=dichloromethane)
#N Geom=AllCheck Guess=TCheck SCRF=Check Test GenChk RM062X/6-31G(d) Freq
----
Pointgroup= C1   Stoichiometry= C35H30BrF3N2O2S
C1[X(C35H30BrF3N2O2S)] #Atoms= 74
Charge = 0   Multiplicity = 1

SCF Energy= -4880.47223239   Predicted Change= -2.849755D-09
==
==============================
Optimization completed.   {Found   2   times}
Item   Max Val.   Criteria   Pass?   RMS Val.   Criteria   Pass?
Force   0.00000 || 0.00045   [YES]   0.00000 || 0.00030   [YES]
Displ   0.00072 || 0.00180   [YES]   0.00072 || 0.00180   [YES]

Atomic Type	Coordinates (Angstroms) X	Y	Z
N	3.499709	1.412834	-0.218480
C	4.100990	0.381315	-1.075967
C	2.990492	-0.426115	-1.726331
C	3.521027	-1.576440	-2.599775
C	4.649097	-1.106832	-3.522893
C	2.388106	-2.174469	-3.439520
C	2.012495	-0.909821	-0.642859
C	2.585241	-1.906638	0.345400
N	1.462651	0.281735	0.056846
C	0.111435	0.300129	0.523629
C	-0.654222	-0.999088	0.511552
C	-1.450412	-1.217312	1.777683
O	-0.333547	1.347195	0.939175
C	2.235168	1.371104	0.218311
S	1.760697	2.827134	1.046450
C	3.381512	3.467288	0.837566
C	4.190430	2.579859	0.132926
C	3.875459	4.695023	1.270410
C	5.200859	5.000072	0.986477
C	6.012799	4.099413	0.285775
C	5.522067	2.876142	-0.151784
C	3.287446	-1.514568	1.488632
C	3.809763	-2.467877	2.360174
C	3.631892	-3.824687	2.102489
C	2.923176	-4.225197	0.971747
C	2.399631	-3.271841	0.102751
C	-2.340657	-0.275087	2.308065
C	-3.048989	-0.552706	3.475166
C	-2.886095	-1.771458	4.129932
C	-2.003818	-2.716318	3.610509
C	-1.293721	-2.436112	2.447149
H	0.058071	-1.822846	0.447756
H	4.764634	-0.245925	-0.468501
H	4.695191	0.910068	-1.823351
H	3.925010	-2.352578	-1.936190
H	4.928933	-1.912753	-4.207356
H	5.549358	-0.819365	-2.971214
H	4.326507	-0.250921	-4.128050
H	2.757686	-3.028087	-4.015509
H	2.010643	-1.426541	-4.146518
H	1.535849	-2.511120	-2.843112
H	2.413297	0.250200	-2.373255
H	1.147955	-1.310640	-1.176999
H	3.243869	5.388115	1.815419
H	5.611127	5.948206	1.316395
H	7.046202	4.356698	0.080750
H	6.156257	2.177055	-0.685269
H	3.421577	-0.460479	1.721173
H	4.350936	-2.147373	3.244822
H	4.035200	-4.565747	2.785214
H	2.766737	-5.280194	0.770069
H	1.824982	-3.585797	-0.764613
H	-2.477530	0.676428	1.807333
H	-3.735593	0.189206	3.872160
H	-3.442081	-1.983614	5.038170
H	-1.864770	-3.669941	4.110818
H	-0.603034	-3.174949	2.045811
C	-1.391898	-0.974472	-0.940713
C	-2.727910	-0.210536	-0.839710
C	-1.658995	-2.456540	-1.281520
F	-2.384245	-2.585032	-2.400083
F	-2.296158	-3.164111	-0.319483
F	-0.494732	-3.112610	-1.493599
O	-0.558292	-0.469415	-1.846775
C	-2.718645	1.099943	-1.317567
C	-3.854849	1.901205	-1.259252
C	-5.022896	1.365552	-0.730613
Br	-6.593871	2.434323	-0.672506
C	-5.070285	0.059975	-0.258402
C	-3.917212	-0.720244	-0.308485
H	-1.793737	1.467568	-1.748139

H	-3.839426	2.920895	-1.629412
H	-5.993319	-0.342335	0.145025
H	-3.962690	-1.733096	0.076085

Statistical Thermodynamic Analysis
Temperature= 298.150 Kelvin Pressure= 1.00000 Atm
==
==============================
SCF Energy= -4880.47223239 Predicted Change= -2.849755D-09
Zero-point correction (ZPE)= -4879.8858 0.58633
Internal Energy (U)= -4879.8483 0.62389
Enthalpy (H)= -4879.8473 0.62484
Gibbs Free Energy (G)= -4879.9577 0.51449

Frequencies -- 12.2055 21.6276 24.5581

183.15K thermal correction = 0.551707
Single point SCF = -4883.528179

TS-K: C–O bond formation

Using Gaussian 09: AM64L-G09RevD.01 24-Apr-2013
==
==============================
#m062x/6-31G(d) scf=(maxcycle=300,direct,tight) density=current
SCRF=(PCM,SOLVENT=CH2Cl2) opt=(maxcycle=250,ts,calcfc,noeigentest,gdiis)
iop(1/8=18) freq=noraman temp=298.15
#N Geom=AllCheck Guess=TCheck SCRF=Check Test GenChk RM062X/6-31G(d) Freq
----
Pointgroup= C1 Stoichiometry= C35H30BrF3N2O2S C1[X(C35H30BrF3N2O2S)] #Atoms= 74
Charge = 0 Multiplicity = 1

SCF Energy= -4880.46924768 Predicted Change= -2.310261D-10
==
==============================
Optimization completed. {Found 2 times}
Item Max Val. Criteria Pass? RMS Val. Criteria Pass?
Force 0.00000 || 0.00045 [YES] 0.00000 || 0.00030 [YES]
Displ 0.00055 || 0.00180 [YES] 0.00055 || 0.00180 [YES]

Atomic Coordinates (Angstroms)
Type X Y Z

N	3.622798	1.335747	-0.272215
C	4.277836	0.191541	-0.916730
C	3.230425	-0.643687	-1.637252
C	3.829416	-1.905972	-2.283751
C	5.093342	-1.581420	-3.086518
C	2.806778	-2.582360	-3.201495
C	2.076096	-0.958242	-0.667238
C	2.445860	-1.887676	0.476183
N	1.519054	0.314316	-0.152619
C	0.098538	0.420081	0.137985
C	-0.666236	-0.876433	0.420462
C	-1.341729	-0.948646	1.765853
O	-0.328368	1.520592	0.425672
C	2.308842	1.379841	-0.004654
S	1.797797	2.937474	0.595227
C	3.462933	3.497371	0.536056
C	4.315120	2.512697	0.042531
C	3.952581	4.747644	0.901129
C	5.316741	4.979616	0.767428
C	6.169957	3.983021	0.280009
C	5.683773	2.734475	-0.089280
C	2.991390	-1.412780	1.671413
C	3.315690	-2.293765	2.701035
C	3.093335	-3.659766	2.550180
C	2.540032	-4.141788	1.365393
C	2.214782	-3.260622	0.338017
C	-2.167201	0.064706	2.268701
C	-2.782467	-0.080688	3.509655
C	-2.590257	-1.237223	4.263435
C	-1.772016	-2.251115	3.771834
C	-1.152211	-2.101927	2.533982
H	0.026373	-1.716966	0.374027
H	4.816428	-0.389193	-0.157572
H	4.999069	0.605232	-1.623442
H	4.105668	-2.602969	-1.481615
H	5.430728	-2.472828	-3.623132
H	5.920302	-1.250731	-2.451267
H	4.893731	-0.799773	-3.829512
H	3.225422	-3.504233	-3.615604
H	2.555633	-1.920334	-4.038369
H	1.872518	-2.838949	-2.694963
H	2.800869	-0.023913	-2.437650
H	1.257510	-1.372219	-1.258533
H	3.287050	5.515362	1.281033
H	5.723440	5.944987	1.048469
H	7.232118	4.183080	0.190122
H	6.350823	1.961989	-0.454995
H	3.149226	-0.347168	1.820392
H	3.733158	-1.907559	3.625474
H	3.340233	-4.344360	3.355409
H	2.350054	-5.203479	1.243149
H	1.755653	-3.637623	-0.572233
H	-2.323503	0.965811	1.685569
H	-3.419955	0.713342	3.887224
H	-3.074435	-1.346510	5.229265
H	-1.611504	-3.155513	4.351058
H	-0.506569	-2.891600	2.153690
C	-1.447229	-0.882503	-0.942248
C	-2.826036	-0.215436	-0.849073
C	-1.616207	-2.340368	-1.392449
F	-2.336521	-2.448992	-2.515553
F	-2.206799	-3.123507	-0.462135
F	-0.419067	-2.918204	-1.643174
O	-0.578838	-0.239844	-1.770186
C	-2.968563	1.032688	-1.453129
C	-4.170936	1.731111	-1.387453
C	-5.241181	1.159429	-0.711372
Br	-6.888605	2.103913	-0.615547
C	-5.132284	-0.085537	-0.103070
C	-3.920191	-0.766346	-0.172949
H	-2.106999	1.439247	-1.970697
H	-4.278136	2.704149	-1.854910
H	-5.978641	-0.514209	0.422700
H	-3.836047	-1.729247	0.319393

Statistical Thermodynamic Analysis
Temperature= 298.150 Kelvin Pressure= 1.00000 Atm
==
==============================
SCF Energy= -4880.46924768 Predicted Change= -2.310261D-10
Zero-point correction (ZPE)= -4879.8836 0.58561
Internal Energy (U)= -4879.8466 0.62261
Enthalpy (H)= -4879.8456 0.62355

Gibbs Free Energy (G)= -4879.9543 0.51487

Frequencies -- -202.1004 17.8628 21.3091

183.15K thermal correction = 0.551510
Single point SCF = -4883.523861

F: Product-catalyst complex

Using Gaussian 09: AM64L-G09RevD.01 24-Apr-2013
==
#m062X/6-31G(d) scf=(direct,tight,maxcycle=300,xqc)
SCRF=(PCM,SOLVENT=CH2Cl2) opt=(maxcycle=250,gdiis) iop(1/8=18)
freq=noraman temp=298.15
#N Geom=AllCheck Guess=TCheck SCRF=Check Test GenChk RM062X/6-31G(d) Freq
----
Pointgroup= C1 Stoichiometry= C35H30BrF3N2O2S
C1[X(C35H30BrF3N2O2S)] #Atoms= 74
Charge = 0 Multiplicity = 1

SCF Energy= -4880.48043572 Predicted Change= -1.925280D-08
==
Optimization completed. {Found 1 times}

Item	Max Val.	Criteria	Pass?	RMS Val.	Criteria	Pass?
Force	0.00003 ||	0.00045	[YES]	0.00000 ||	0.00030	[YES]
Displ	0.00206 ||	0.00180	[NO]	0.00206 ||	0.00180	[YES]

Atomic Coordinates (Angstroms)

Type	X	Y	Z
N	3.650144	1.201120	-0.509009
C	4.329848	-0.012090	-0.970192
C	3.319608	-0.948140	-1.617569
C	3.944669	-2.295029	-2.020162
C	5.251047	-2.096723	-2.795946
C	2.970979	-3.114949	-2.872122
C	2.104882	-1.090038	-0.676510
C	2.410917	-1.805433	0.633285
N	1.521544	0.238620	-0.439463
C	0.000585	0.402398	-0.264481
C	-0.659034	-0.749794	0.584377
C	-1.201880	-0.442029	1.941926
O	-0.314344	1.605517	-0.061444
C	2.313123	1.287327	-0.344183
S	1.791354	2.916892	0.048560
C	3.477082	3.442164	0.035364
C	4.342359	2.397101	-0.278468
C	3.972023	4.715807	0.293026
C	5.348935	4.913930	0.237084
C	6.211117	3.857557	-0.070289
C	5.720766	2.581848	-0.332755
C	2.837695	-1.120157	1.773587
C	3.094434	-1.806811	2.958746
C	2.921294	-3.186728	3.022532
C	2.486305	-3.877989	1.893242
C	2.230530	-3.190919	0.709903
C	-2.020196	0.666588	2.194522
C	-2.484328	0.915524	3.483329
C	-2.138846	0.068340	4.535387
C	-1.317872	-1.031072	4.295695
C	-0.854044	-1.280497	3.006956
H	0.006204	-1.611582	0.659446
H	4.836997	-0.488550	-0.121271
H	5.082296	0.304693	-1.695347
H	4.174024	-2.852636	-1.102752
H	5.615343	-3.058959	-3.167169
H	6.042666	-1.663143	-2.177774
H	5.092122	-1.442104	-3.661609
H	3.401615	-4.094324	-3.100193
H	2.778408	-2.602330	-3.821917
H	2.005257	-3.281857	-2.386682
H	2.947923	-0.460330	-2.530412
H	1.329606	-1.638717	-1.209300
H	3.299669	5.532714	0.534698
H	5.755814	5.899541	0.436564
H	7.281649	4.029338	-0.103488
H	6.393176	1.761978	-0.560363
H	2.953623	-0.039721	1.758454
H	3.419736	-1.256402	3.835957
H	3.115506	-3.719225	3.948151
H	2.335781	-4.952235	1.934271
H	1.867566	-3.734426	-0.158883
H	-2.273602	1.332999	1.378028
H	-3.119282	1.777075	3.668295
H	-2.505899	0.266552	5.538109
H	-1.038179	-1.692762	5.110012
H	-0.205164	-2.134651	2.818451
C	-1.525517	-0.951198	-0.688041
C	-2.910409	-0.332619	-0.697600
C	-1.597731	-2.403062	-1.153785
F	-2.183589	-2.509859	-2.350910
F	-2.283832	-3.167206	-0.289127
F	-0.375701	-2.961474	-1.265296
O	-0.595090	-0.249601	-1.499101
C	-3.152335	0.778990	-1.502574
C	-4.412427	1.369268	-1.536372
C	-5.427564	0.834900	-0.753208
Br	-7.157292	1.615185	-0.819372
C	-5.206417	-0.261807	0.071662
C	-3.942192	-0.840966	0.097323
H	-2.336771	1.186119	-2.088148
H	-4.604323	2.232389	-2.164373
H	-6.008020	-0.656173	0.686274
H	-3.762869	-1.685830	0.753560

Statistical Thermodynamic Analysis
Temperature= 298.150 Kelvin Pressure= 1.00000 Atm
==
SCF Energy= -4880.48043572 Predicted Change= -1.925280D-08
Zero-point correction (ZPE)= -4879.8943 0.58610
Internal Energy (U)= -4879.8570 0.62339
Enthalpy (H)= -4879.8560 0.62434
Gibbs Free Energy (G)= -4879.9658 0.51462

Frequencies -- 15.8490 22.3150 24.0400

183.15K thermal correction = 0.551625
Single point SCF = -4883.531745

TS-G: Catalyst release

Using Gaussian 09: AM64L-G09RevD.01 24-Apr-2013
==

# m062x/6-31G(d) scf=(maxcycle=300,direct,tight,xqc)
density=current
opt=(maxcycle=250,modredundant)
SCRF=(PCM,SOLVENT=CH2Cl2) iop(1/8=18)
Temperature=298.15
Modredundant Input: B 9 43 F
Modredundant Input:
# m062x/6-31G(d) scf=(direct,tight,maxcycle=300,xqc)
opt=(nofreeze,maxcycle=250,ts,calcfc,noeigentest) iop(1/8=18)
freq=noraman
SCRF=(PCM,SOLVENT=CH2Cl2) Temperature=298.15
geom=check guess=read
#N Geom=AllCheck Guess=TCheck SCRF=Check Test GenChk
RM062X/6-31G(d) Freq

Pointgroup= C1 Stoichiometry= C35H30BrF3N2O2S
C1[X(C35H30BrF3N2O2S)] #Atoms= 74
Charge = 0 Multiplicity = 1

SCF Energy= -4880.47109192 Predicted Change= -7.038111D-09
==
==============================
Optimization completed. {Found 2 times}

Item	Max Val.	Criteria	Pass?	RMS Val.	Criteria	Pass?
Force	0.00000 ||	0.00045	[YES]	0.00000 ||	0.00030	[YES]
Displ	0.00186 ||	0.00180	[NO]	0.00186 ||	0.00180	[YES]

Atomic Coordinates (Angstroms)

Type	X	Y	Z
N	-3.915909	0.613732	0.404479
C	-4.495497	-0.638195	0.887129
C	-3.425536	-1.479605	1.583932
C	-3.961490	-2.878282	1.938715
C	-5.283429	-2.793395	2.708608
C	-2.939570	-3.667913	2.759820
C	-2.119543	-1.521975	0.757327
C	-2.162276	-2.408980	-0.473252
N	-1.691157	-0.167961	0.389405
C	-2.569519	0.769373	0.275376
S	-2.146597	2.449916	-0.076934
C	-3.855352	2.883427	-0.094068
C	-4.660362	1.774133	0.182150
C	-4.413371	4.130841	-0.335048
C	-5.801711	4.251252	-0.298084
C	-6.604941	3.142505	-0.027507
C	-6.046103	1.889456	0.214458
C	-1.368281	-3.554627	-0.524155
C	-1.374453	-4.376653	-1.648711
C	-2.176513	-4.057615	-2.740669
C	-2.958633	-2.904134	-2.707854
C	-2.947089	-2.083008	-1.584375
H	-4.959030	-1.182620	0.054902
H	-5.280940	-0.371982	1.598788
H	-4.144609	-3.415804	0.997748
H	-5.599679	-3.792551	3.022229
H	-6.092393	-2.369078	2.106640
H	-5.168754	-2.179610	3.610462
H	-2.667939	-3.115635	3.667296
H	-2.023675	-3.873681	2.200260
H	-3.361673	-4.630104	3.064760
H	-3.169193	-0.962823	2.520490
H	-1.328998	-1.896727	1.413969
H	-3.783778	4.988604	-0.546496
H	-6.257967	5.217743	-0.482410
H	-7.683938	3.252231	-0.005818
H	-6.673588	1.028063	0.416485
H	-0.710845	-3.784583	0.310382
H	-0.743560	-5.259883	-1.673946
H	-2.181396	-4.694111	-3.620058
H	-3.569656	-2.635951	-3.564353
H	-3.523927	-1.161429	-1.597545
C	-0.003828	0.242821	0.826320
C	0.628220	0.895519	-0.448331
C	1.347737	2.200184	-0.303696
O	-0.008075	0.671490	1.971196
C	1.270901	3.138235	-1.337356
C	1.950103	4.351370	-1.253169
C	2.712833	4.641620	-0.124872
C	2.789239	3.716222	0.915253
C	2.112430	2.503425	0.829504
H	-0.084937	0.943872	-1.274489
H	0.674235	2.910397	-2.217872
H	1.881061	5.067567	-2.066243
H	3.243825	5.586097	-0.053884
H	3.379573	3.940793	1.798435
H	2.168304	1.785925	1.641373
C	1.410731	-0.450111	-0.517981
C	2.884749	-0.421899	-0.177807
C	1.167802	-1.223471	-1.809958
F	1.851778	-0.689474	-2.836798
F	-0.122378	-1.222104	-2.158289
F	1.553383	-2.498341	-1.680475
O	0.626031	-1.037723	0.517682
C	3.791308	0.292893	-0.966628
C	5.142298	0.318103	-0.642297
C	5.579548	-0.376862	0.480649
Br	7.423612	-0.350336	0.926630
C	4.695910	-1.085512	1.283366
C	3.344144	-1.102769	0.947384
H	3.444101	0.849084	-1.831442
H	5.846515	0.875307	-1.249812
H	5.054809	-1.615658	2.158635
H	2.635200	-1.639854	1.567242

Statistical Thermodynamic Analysis
Temperature= 298.150 Kelvin Pressure= 1.00000 Atm
==
==============================
SCF Energy= -4880.47109192 Predicted Change= -7.038111D-09
Zero-point correction (ZPE)= -4879.8869 0.58415
Internal Energy (U)= -4879.8495 0.62154
Enthalpy (H)= -4879.8486 0.62248
Gibbs Free Energy (G)= -4879.9592 0.51186

Frequencies -- -132.3537 13.2376 24.6362

183.15K thermal correction = 0.549236
Single point SCF = -4883.523794

H: Major Product

Using Gaussian 09: AM64L-G09RevD.01 24-Apr-2013
==
==============================
#m062X/6-31G(d) scf=(direct,tight,maxcycle=300,xqc)
SCRF=(PCM,SOLVENT=CH2Cl2) opt=(maxcycle=250,gdiis)
iop(1/8=18)
freq=noraman temp=298.15
#N Geom=AllCheck Guess=TCheck SCRF=Check Test GenChk
RM062X/6-31G(d) Freq

Pointgroup= C1 Stoichiometry= C16H10BrF3O2
C1[X(C16H10BrF3O2)] #Atoms= 32
Charge = 0 Multiplicity = 1

SCF Energy= -3637.08787315 Predicted Change= -7.301590D-09

==
==============================

Optimization completed. {Found 2 times}

Item	Max Val.	Criteria	Pass?	RMS Val.	Criteria	Pass?
Force	0.00001 ||	0.00045	[YES]	0.00000 ||	0.00030	[YES]
Displ	0.00136 ||	0.00180	[YES]	0.00136 ||	0.00180	[YES]

Atomic Coordinates (Angstroms)

Type	X	Y	Z
C	-2.830749	-0.193651	1.517463
C	-2.484256	0.291395	0.114455
C	-1.871391	1.651225	-0.045266
O	-3.476965	0.175324	2.443996
C	-2.053378	2.347776	-1.240855
C	-1.442953	3.584374	-1.434630
C	-0.650513	4.133979	-0.429916
C	-0.470807	3.445680	0.768754
C	-1.077902	2.208563	0.960811
C	-1.572022	-0.978703	0.131102
C	-0.080469	-0.790202	0.107736
C	-2.034096	-2.051500	-0.847119
F	-1.401610	-3.206661	-0.637650
F	-1.797691	-1.662486	-2.106355
F	-3.348264	-2.273690	-0.728275
O	-2.069287	-1.331935	1.445750
C	0.692278	-1.199040	1.192386
C	2.072536	-1.016893	1.178654
C	2.663558	-0.424995	0.069942
Br	4.538615	-0.162599	0.049507
C	1.907908	-0.012727	-1.023555
C	0.531342	-0.198041	-1.000462
H	-3.340198	0.161454	-0.554487
H	-2.671693	1.916786	-2.024227
H	-1.589826	4.117310	-2.368602
H	-0.176231	5.098988	-0.578072
H	0.142992	3.871990	1.555745
H	-0.930542	1.676627	1.898026
H	0.216316	-1.654398	2.053891
H	2.678467	-1.329441	2.021423
H	2.388191	0.452405	-1.876741
H	-0.065407	0.134233	-1.844157

Statistical Thermodynamic Analysis
Temperature= 298.150 Kelvin Pressure= 1.00000 Atm

==
==============================

SCF Energy= -3637.08787315 Predicted Change= -7.301590D-09
Zero-point correction (ZPE)= -3636.8591 0.22870
Internal Energy (U)= -3636.8410 0.24680
Enthalpy (H)= -3636.8401 0.24774
Gibbs Free Energy (G)= -3636.9084 0.17944

Frequencies -- 24.8201 36.1145 42.2829

183.15K thermal correction = 0.203257
Single point SCF = -3639.845287

Concerted [2+2] Mechanism Additional Structures: PBE

TS-A: HBTM addition to phenylacetic anhydride (Si-face)

Using Gaussian 09: AM64L-G09RevD.01 24-Apr-2013

==
==============================

# pbepbe/6-31G(d)/auto scf=(maxcycle=300,direct,tight,xqc) density=current
opt=(maxcycle=250,modredundant) SCRF=(PCM,SOLVENT=Dichloromethane)
iop(1/8=18) Temperature=298.15
Modredundant Input: B 17 23 F
Modredundant Input:
# pbepbe/6-31G(d)/auto scf=(direct,tight,maxcycle=300,xqc)
opt=(nofreeze,maxcycle=250,ts,calcfc,noeigentest) iop(1/8=18) freq=noraman
SCRF=(PCM,SOLVENT=Dichloromethane) Temperature=298.15 geom=check
guess=read
#N Geom=AllCheck Guess=TCheck SCRF=Check Test GenChk RPBEPBE/6-31G(d)/Auto
Freq

Pointgroup= C1 Stoichiometry= C35H34N2O3S
C1[X(C35H34N2O3S)] #Atoms= 75
Charge = 0 Multiplicity = 1

SCF Energy= -2085.34021963 Predicted Change= -5.563560D-10

==
==============================

Optimization completed on the basis of negligible forces.
{Found 3 times}

Item	Max Val.	Criteria	Pass?	RMS Val.	Criteria	Pass?
Force	0.00000 ||	0.00045	[YES]	0.00000 ||	0.00030	[YES]
Displ	0.00193 ||	0.00180	[NO]	0.00193 ||	0.00180	[YES]

Atomic Coordinates (Angstroms)

Type	X	Y	Z
C	-5.902319	-2.852028	-0.280716
C	-4.977204	-2.039677	0.392835
C	-3.799584	-1.681421	-0.278942
C	-3.548791	-2.136029	-1.590444
C	-4.472515	-2.947663	-2.256395
C	-5.657279	-3.300345	-1.589819
N	-2.764679	-0.870179	0.205960
C	-1.714401	-0.685245	-0.662684
S	-1.966525	-1.590506	-2.181568
C	-2.802390	-0.190368	1.508711
C	-1.889256	1.042864	1.497084
C	-1.837377	1.744381	2.882430
C	-1.139080	3.114139	2.792419
C	-3.241527	1.934990	3.491422
C	-0.490227	0.644787	0.920425
C	0.326913	-0.264748	1.842395
N	-0.663574	0.054841	-0.420425
C	1.262633	0.310256	2.727024
C	1.990429	-0.480606	3.627706
C	1.801762	-1.870678	3.655159
C	0.888843	-2.458561	2.767374
C	0.160849	-1.663674	1.867902
C	0.583435	0.182479	-1.802403
C	0.117255	1.432726	-2.584228

C	-0.074342	2.749920	-1.867509
O	1.715088	0.507783	-0.842213
C	2.940902	0.714766	-1.376239
C	4.015717	0.720414	-0.284242
C	4.679965	-0.645158	-0.193205
O	3.188408	0.858698	-2.569505
O	0.647040	-0.914212	-2.371205
C	1.021217	3.587524	-1.570006
C	0.832374	4.823948	-0.936243
C	-0.460410	5.255790	-0.599142
C	-1.561656	4.441611	-0.904380
C	-1.366909	3.201157	-1.531184
C	5.931255	-0.877347	-0.794006
C	6.535625	-2.141540	-0.721685
C	5.891865	-3.192438	-0.051404
C	4.639745	-2.971064	0.543742
C	4.036635	-1.707912	0.471349
H	-6.826467	-3.140419	0.229182
H	-5.170770	-1.703918	1.415239
H	-4.275515	-3.300118	-3.273393
H	-6.391439	-3.933128	-2.097220
H	-2.504472	-0.904079	2.300837
H	-3.846659	0.106850	1.688570
H	-0.105163	3.049532	2.415235
H	-1.097420	3.585400	3.789496
H	-1.692412	3.792990	2.118976
H	-1.255900	1.099341	3.569189
H	-3.904005	2.493056	2.803950
H	-3.168492	2.516209	4.426551
H	-3.732545	0.979221	3.742090
H	-2.313738	1.765218	0.772427
H	0.087693	1.567536	0.757012
H	1.429273	1.393147	2.707054
H	2.712189	-0.008597	4.302511
H	2.370832	-2.492128	4.354204
H	0.743399	-3.543957	2.766533
H	-0.525296	-2.151400	1.167991
H	-0.820232	1.132690	-3.084195
H	0.885116	1.537809	-3.371804
H	3.557883	0.991825	0.681196
H	4.759534	1.487177	-0.555530
H	2.029536	3.274490	-1.862198
H	1.697400	5.459731	-0.718511
H	-0.609086	6.226178	-0.113802
H	-2.576644	4.776236	-0.663702
H	-2.233108	2.577536	-1.782805
H	6.438129	-0.058620	-1.317695
H	7.512794	-2.303693	-1.189173
H	6.363901	-4.178923	0.007867
H	4.128354	-3.784509	1.069857
H	3.058292	-1.542876	0.935179

Statistical Thermodynamic Analysis
Temperature= 298.150 Kelvin Pressure= 1.00000 Atm
==
=============================
SCF Energy= -2085.34021963 Predicted Change= -5.563560D-10
Zero-point correction (ZPE)= -2084.7419 0.59827
Internal Energy (U)= -2084.7050 0.63516
Enthalpy (H)= -2084.7041 0.63610
Gibbs Free Energy (G)= -2084.8143 0.52589

Frequencies -- -85.3683 8.8157 16.5877

183.15K thermal correction = 0.563158
Single point SCF = -2085.854273

B: Acylated-HBTM (S•••O syn)

Using Gaussian 09: AM64L-G09RevD.01 24-Apr-2013
==
=============================
# pbepbe/6-31G*/auto gfprint gfinput scf=(direct,tight,maxcycle=300,xqc)
opt=(maxcycle=250) freq=noraman iop(1/8=18) Temperature=298.15
SCRF=(PCM,SOLVENT=Dichloromethane)
#N Geom=AllCheck Guess=TCheck SCRF=Check Test GenChk RPBEPBE/6-31G(d)/Auto
Freq

Pointgroup= C1 Stoichiometry= C27H27N2OS(1+)
C1[X(C27H27N2OS)] #Atoms= 58
Charge = 1 Multiplicity = 1

SCF Energy= -1626.21690894 Predicted Change= -2.215250D-09
==
=============================
Optimization completed. {Found 2 times}
Item Max Val. Criteria Pass? RMS Val. Criteria Pass?
Force 0.00000 || 0.00045 [YES] 0.00000 || 0.00030 [YES]
Displ 0.00166 || 0.00180 [YES] 0.00166 || 0.00180 [YES]

Atomic Type	Coordinates (Angstroms) X	Y	Z
C	-4.892403	-3.498032	0.652155
C	-5.416876	-2.199984	0.485500
C	-4.587237	-1.110708	0.200293
C	-3.210727	-1.359232	0.084461
C	-2.682442	-2.652952	0.256283
C	-3.518682	-3.741003	0.539750
N	-2.202549	-0.420090	-0.199871
C	-0.958169	-0.943512	-0.226993
S	-0.922682	-2.664819	0.064838
C	-2.489771	1.000601	-0.480819
C	-1.347418	1.608335	-1.294287
C	-1.563072	3.118441	-1.598176
C	-0.549410	3.621680	-2.643484
C	-2.993525	3.404758	-2.095975
C	0.004362	1.313727	-0.582247
C	0.223024	2.020450	0.753412
N	0.153303	-0.173527	-0.442325
C	1.429449	-0.809092	-0.448457
C	2.651257	0.073243	-0.661765
C	3.949237	-0.680833	-0.458091
O	1.486300	-2.026469	-0.287228
C	-0.308907	1.533788	1.964747
C	-0.108558	2.234139	3.162767
C	0.628254	3.427240	3.170290
C	1.174682	3.912606	1.973721
C	0.978501	3.211094	0.776145
C	4.579974	-1.326573	-1.536628
C	5.783888	-2.019306	-1.345061
C	6.369473	-2.072939	-0.071148
C	5.746344	-1.430475	1.009562
C	4.542880	-0.737738	0.816130
H	-5.566950	-4.329526	0.874834
H	-6.493574	-2.035738	0.583079
H	-5.001225	-0.106433	0.082623
H	-3.110184	-4.746960	0.670652
H	-2.645298	1.525827	0.479322

H	-3.429405	1.024023	-1.050106
H	0.500384	3.511735	-2.323089
H	-0.715668	4.693460	-2.842491
H	-0.669494	3.079636	-3.598924
H	-1.410383	3.681529	-0.657742
H	-3.078072	4.465352	-2.385849
H	-3.759474	3.219192	-1.324285
H	-3.240186	2.796104	-2.985036
H	-1.311043	1.075501	-2.265165
H	0.797427	1.617643	-1.279651
H	2.592196	0.941180	0.020329
H	2.602819	0.484218	-1.689243
H	-0.866021	0.591404	1.995504
H	-0.526271	1.838844	4.093887
H	0.785419	3.970359	4.107364
H	1.765256	4.833950	1.969867
H	1.424229	3.591706	-0.149728
H	4.128119	-1.283469	-2.534408
H	6.266944	-2.514361	-2.193762
H	7.311585	-2.610442	0.078085
H	6.199972	-1.463874	2.005434
H	4.061357	-0.233034	1.661770

Statistical Thermodynamic Analysis
Temperature= 298.150 Kelvin Pressure= 1.00000 Atm
==
==============================

SCF Energy= -1626.21690894 Predicted Change= -2.215250D-09
Zero-point correction (ZPE)= -1625.7458 0.47101
Internal Energy (U)= -1625.7184 0.49849
Enthalpy (H)= -1625.7174 0.49943
Gibbs Free Energy (G)= -1625.8061 0.41077

Frequencies -- 11.2355 20.0472 28.4408

183.15K thermal correction = 0.441018
Single point SCF = -1626.585470

B: Acylated-HBTM (S•••O anti)

Using Gaussian 09: AM64L-G09RevD.01 24-Apr-2013
==
==============================

# pbepbe/6-31G*/auto gfprint gfinput scf=(direct,tight,maxcycle=300,xqc)
opt=(maxcycle=250) freq=noraman iop(1/8=18) Temperature=298.15
SCRF=(PCM,SOLVENT=Dichloromethane)
#N Geom=AllCheck Guess=TCheck SCRF=Check Test GenChk RPBEPBE/6-31G(d)/Auto
Freq

Pointgroup= C1 Stoichiometry= C27H27N2OS(1+)
C1[X(C27H27N2OS)] #Atoms= 58
Charge = 1 Multiplicity = 1

SCF Energy= -1626.20455775 Predicted Change= -8.912282D-09
==
==============================

Optimization completed. {Found 1 times}

Item	Max Val.	Criteria	Pass?	RMS Val.	Criteria	Pass?
Force	0.00001 ||	0.00045	[YES]	0.00000 ||	0.00030	[YES]
Displ	0.00559 ||	0.00180	[NO]	0.00559 ||	0.00180	[YES]

Atomic Coordinates (Angstroms)

Type	X	Y	Z
C	-3.686452	-4.511269	0.596843
C	-4.523619	-3.460807	0.171238
C	-4.006481	-2.202740	-0.156027
C	-2.619488	-2.021032	-0.044279
C	-1.787890	-3.068718	0.392207
C	-2.303104	-4.330318	0.712552
N	-1.893582	-0.851276	-0.331119
C	-0.558562	-0.944786	-0.109159
S	-0.111251	-2.545334	0.457480
C	-2.564310	0.361888	-0.840142
C	-1.545635	1.311795	-1.463137
C	-2.186196	2.667582	-1.874193
C	-1.209494	3.510802	-2.714558
C	-3.496380	2.463352	-2.660406
C	-0.334868	1.475108	-0.510123
C	-0.585063	2.213486	0.801602
N	0.278926	0.121304	-0.264288
C	1.732470	0.135813	-0.185257
C	2.488788	-1.174172	-0.010810
C	3.989217	-0.994057	-0.138448
O	2.287375	1.218335	-0.280281
C	-1.240947	1.631252	1.905602
C	-1.463751	2.371206	3.075793
C	-1.034992	3.703344	3.163077
C	-0.369362	4.288894	2.076650
C	-0.139995	3.546373	0.910611
C	4.632519	-1.251154	-1.362138
C	6.021040	-1.093892	-1.481915
C	6.779451	-0.679511	-0.377113
C	6.143860	-0.423697	0.847770
C	4.756275	-0.581409	0.966154
H	-4.120221	-5.484014	0.844183
H	-5.601583	-3.626797	0.094137
H	-4.665568	-1.393627	-0.478201
H	-1.649781	-5.141326	1.044273
H	-3.110136	0.830349	-0.000887
H	-3.295319	0.026762	-1.590417
H	-1.687968	4.460706	-3.006015
H	-0.927533	2.978160	-3.640784
H	-0.282461	3.766157	-2.175106
H	-2.423986	3.222556	-0.946316
H	-4.294644	2.007546	-2.050430
H	-3.334679	1.828554	-3.550848
H	-3.874629	3.438134	-3.011074
H	-1.156612	0.832200	-2.383454
H	0.452713	2.015719	-1.051696
H	2.131982	-1.909735	-0.754807
H	2.247584	-1.585084	0.989008
H	-1.566893	0.586385	1.881786
H	-1.970959	1.899159	3.923140
H	-1.209158	4.278512	4.077911
H	-0.014577	5.322452	2.138610
H	0.402329	4.005444	0.076915
H	4.044785	-1.580002	-2.227045
H	6.510214	-1.300681	-2.439295
H	7.863970	-0.560454	-0.468594
H	6.730007	-0.104539	1.715641
H	4.264565	-0.384058	1.925432

Statistical Thermodynamic Analysis
Temperature= 298.150 Kelvin Pressure= 1.00000 Atm
==
==============================

 SCF Energy=   -1626.20455775   Predicted Change= -8.912282D-09
 Zero-point correction (ZPE)=   -1625.7339   0.47060
 Internal Energy (U)=   -1625.7063   0.49820
 Enthalpy (H)=   -1625.7054   0.49914
 Gibbs Free Energy (G)=   -1625.7940   0.41050

 Frequencies --   15.1174   21.8270   30.8820

183.15K thermal correction = 0.440730
Single point SCF = -1626.574352

TS-C: Acylated-HBTM deprotonation *via* phenylacetate (making E)

 Using Gaussian 09: AM64L-G09RevD.01 24-Apr-2013
==
 # pbepbe/6-31G(d)/auto scf=(maxcycle=300,direct,tight,xqc) density=current
 opt=(maxcycle=250,modredundant) SCRF=(PCM,SOLVENT=Dichloromethane)
 iop(1/8=18) Temperature=298.15
 Modredundant Input: B   24   52 F
 Modredundant Input: B   52   63 F
 Modredundant Input:
 # pbepbe/6-31G(d)/auto scf=(direct,tight,maxcycle=300,xqc)
 opt=(nofreeze,maxcycle=250,ts,calcfc,noeigentest) iop(1/8=18) freq=noraman
 SCRF=(PCM,SOLVENT=Dichloromethane) Temperature=298.15 geom=check
 guess=read
 #N Geom=AllCheck Guess=TCheck SCRF=Check Test GenChk RPBEPBE/6-31G(d)/Auto
 Freq

 Pointgroup= C1   Stoichiometry= C35H34N2O3S
C1[X(C35H34N2O3S)] #Atoms= 75
 Charge = 0   Multiplicity = 1

 SCF Energy= -2085.32995526   Predicted Change= -1.417691D-09
==
 Optimization completed.   {Found   3   times}
 Item   Max Val.   Criteria   Pass?   RMS Val.   Criteria   Pass?
 Force   0.00001 || 0.00045   [YES]   0.00000 || 0.00030   [YES]
 Displ   0.00072 || 0.00180   [YES]   0.00072 || 0.00180   [YES]

Atomic Type	Coordinates (Angstroms) X	Y	Z
C	-5.004534	-2.801717	0.512805
C	-3.752371	-2.352368	0.952953
C	-2.879041	-1.816768	-0.005734
C	-3.237259	-1.741819	-1.363619
C	-4.491965	-2.189734	-1.795934
C	-5.373346	-2.720740	-0.843701
N	-1.592746	-1.297353	0.223486
C	-0.957465	-0.865231	-0.895980
S	-1.937739	-1.020914	-2.346854
C	-1.028938	-1.164626	1.576976
C	0.111578	-0.147021	1.582373
C	0.811116	-0.074942	2.969172
C	1.802884	1.101286	3.042940
C	-0.215784	0.054619	4.113215
C	1.076404	-0.451579	0.397133
C	1.846813	-1.767621	0.513143
N	0.303099	-0.383270	-0.876139
C	1.323335	-3.000503	0.074055
C	2.045580	-4.190923	0.246105
C	3.306390	-4.170662	0.858703
C	3.845565	-2.948087	1.284435
C	3.125150	-1.758966	1.107466
C	0.843446	0.167796	-2.130063
C	2.008141	0.997647	-2.159309
C	3.323641	0.838903	-1.439188
O	0.087240	0.000487	-3.122566
C	3.731723	1.707521	-0.402163
C	5.010692	1.602678	0.169419
C	5.918380	0.637326	-0.292342
C	5.533989	-0.226460	-1.329867
C	4.254373	-0.124036	-1.893551
H	-5.703380	-3.224718	1.240486
H	-3.470806	-2.426681	2.006537
H	-4.775364	-2.128743	-2.851252
H	-6.357255	-3.078632	-1.161152
H	-0.692526	-2.161145	1.919809
H	-1.851261	-0.828638	2.226544
H	2.263565	1.141583	4.045148
H	1.288576	2.058865	2.853533
H	2.618859	1.023151	2.305764
H	1.369536	-1.019565	3.120008
H	0.309695	0.195084	5.073118
H	-0.852105	-0.840417	4.219611
H	-0.873334	0.930376	3.960439
H	-0.314745	0.852764	1.371977
H	1.789163	0.380460	0.342131
H	0.352186	-3.047073	-0.429107
H	1.619215	-5.135476	-0.107290
H	3.870550	-5.099641	0.991095
H	4.839054	-2.913722	1.743490
H	3.574890	-0.808506	1.411888
H	1.254487	2.330736	-1.804462
H	2.179677	1.225740	-3.225011
H	3.023004	2.459235	-0.035387
H	5.301357	2.289153	0.972859
H	6.918486	0.561587	0.148205
H	6.232794	-0.983765	-1.702517
H	3.963375	-0.799308	-2.706807
O	0.867500	3.097235	0.510249
C	0.246312	3.498424	-0.486241
C	-1.028995	4.348329	-0.332204
C	-2.149889	3.461691	0.179225
O	0.543824	3.218357	-1.742346
C	-2.954044	2.733540	-0.719896
C	-3.943938	1.857923	-0.249506
C	-4.143063	1.693220	1.130646
C	-3.348191	2.414720	2.035471
C	-2.358308	3.289817	1.562126
H	-1.293639	4.800051	-1.301268
H	-0.816959	5.153569	0.391112
H	-2.797832	2.852923	-1.798213
H	-4.560242	1.300697	-0.963045
H	-4.916343	1.009751	1.497024
H	-3.502196	2.301945	3.114356
H	-1.737925	3.848910	2.271554

 Statistical Thermodynamic Analysis
 Temperature= 298.150 Kelvin   Pressure= 1.00000 Atm
==
 SCF Energy=   -2085.32995526   Predicted Change= -1.417691D-09

Zero-point correction (ZPE)= -2084.7351 0.59483
Internal Energy (U)= -2084.6981 0.63184
Enthalpy (H)= -2084.6971
0.63279
Gibbs Free Energy (G)= -2084.8066
0.52334

Frequencies -- -356.6253 18.2467 20.6046

183.15K thermal correction = 0.560310
Single point SCF = -2085.849566

TS-C: Acylated-HBTM deprotonation *via N,N*-diisopropylethylamine (making E)

Using Gaussian 09: AM64L-G09RevD.01 24-Apr-2013
==
==============================
# pbepbe/6-31G(d)/auto gfprint gfinput
scf=(direct,tight,maxcycle=300,xqc)
opt=(maxcycle=250,calcfc,ts,noeigentest) freq=noraman
SCRF=(PCM,SOLVENT=Dichloromethane) iop(1/8=18)
Temperature=298.15
#N Geom=AllCheck Guess=TCheck SCRF=Check Test GenChk
RPBEPBE/6-31G(d)/Auto
Freq

Pointgroup= C1 Stoichiometry= C35H46N3OS(1+)
C1[X(C35H46N3OS)] #Atoms= 86
Charge = 1 Multiplicity = 1

SCF Energy= -1996.71555859 Predicted Change= -1.876866D-10
==
==============================
Optimization completed. {Found 2 times}
Item Max Val. Criteria Pass? RMS Val. Criteria Pass?
Force 0.00000 || 0.00045 [YES] 0.00000 || 0.00030 [YES]
Displ 0.00023 || 0.00180 [YES] 0.00023 || 0.00180 [YES]

Atomic Type	Coordinates (Angstroms) X	Y	Z
C	6.358903	-2.209897	-0.518428
C	5.503723	-1.205872	-0.048655
C	4.136488	-1.328751	-0.340192
C	3.642283	-2.413469	-1.086285
C	4.503073	-3.413907	-1.556007
C	5.867614	-3.301700	-1.260635
N	3.107792	-0.446752	0.039832
C	1.875312	-0.803247	-0.398829
S	1.890388	-2.284724	-1.340177
C	3.357362	0.746996	0.863632
C	2.077616	1.145296	1.595527
C	2.260479	2.432553	2.450647
C	1.067728	2.648941	3.400666
C	3.565063	2.395616	3.271270
C	0.904254	1.240876	0.576301
C	0.974381	2.403369	-0.410900
N	0.761099	-0.069141	-0.137018
C	1.734772	2.341051	-1.595772
C	1.792462	3.440056	-2.464738
C	1.089753	4.616069	-2.165757
C	0.321980	4.685620	-0.994338
C	0.261429	3.586008	-0.127594
C	-0.497973	-0.483338	-0.767011
C	-1.711561	0.238930	-0.461046
C	-2.751244	0.405715	-1.529326
O	-0.421280	-1.503617	-1.484236
C	-3.607692	1.530744	-1.445104
C	-4.621932	1.750803	-2.384929
C	-4.808175	0.853087	-3.448171
C	-3.963979	-0.261578	-3.551555
C	-2.953827	-0.490869	-2.604579
H	7.429131	-2.138180	-0.304732
H	5.897193	-0.357138	0.516188
H	4.120116	-4.257902	-2.137170
H	6.559114	-4.071532	-1.614916
H	3.732364	1.559135	0.213709
H	4.146669	0.479748	1.580099
H	1.224073	3.562352	3.998734
H	0.964544	1.801327	4.102463
H	0.106988	2.769293	2.872603
H	2.318823	3.294270	1.758721
H	3.612241	3.278202	3.930803
H	4.467314	2.417214	2.637410
H	3.613309	1.497128	3.913311
H	1.822104	0.317698	2.287622
H	-0.008728	1.344551	1.179080
H	2.272720	1.426627	-1.867732
H	2.384138	3.370191	-3.382903
H	1.132372	5.470798	-2.848099
H	-0.241928	5.593260	-0.757270
H	-0.353830	3.649556	0.777557
H	-2.390740	-0.526005	0.565161
H	-1.546772	1.196102	0.046555
H	-3.465011	2.250543	-0.629411
H	-5.263278	2.633837	-2.289936
H	-5.598514	1.023208	-4.186547
H	-4.090129	-0.968913	-4.378827
H	-2.301318	-1.360431	-2.708211
N	-3.139835	-1.120729	1.536755
C	-2.871231	-2.622012	1.511961
C	-2.870286	-3.232754	0.101680
C	-3.765014	-3.414902	2.478917
C	-2.676171	-0.509329	2.861561
C	-2.887629	1.015625	2.905391
C	-1.212573	-0.859641	3.186226
C	-4.581200	-0.718205	1.324601
C	-5.367121	-1.349073	0.175938
H	-2.249869	-2.650802	-0.597430
H	-3.874590	-3.355163	-0.326177
H	-2.421831	-4.238540	0.179602
H	-4.824482	-3.400312	2.174707
H	-3.697292	-3.050318	3.517195
H	-3.435253	-4.467118	2.479014
H	-1.832780	-2.708102	1.864480
H	-2.457130	1.397572	3.845183
H	-3.949743	1.302496	2.898886
H	-2.385597	1.534754	2.071832
H	-1.087219	-1.894933	3.539235
H	-0.859201	-0.201530	3.996197
H	-0.547292	-0.712168	2.318273
H	-3.318559	-0.947393	3.649533
H	-5.624787	-2.403662	0.355170
H	-4.846291	-1.261430	-0.789939
H	-6.316090	-0.791903	0.093533
H	-4.556744	0.367673	1.152815
H	-5.119995	-0.886052	2.276577

Statistical Thermodynamic Analysis
Temperature= 298.150 Kelvin Pressure= 1.00000 Atm
==
==============================
SCF Energy= -1996.71555859 Predicted Change= -1.876866D-10

Zero-point correction (ZPE)= -1995.9891 0.72638
Internal Energy (U)= -1995.9487 0.76681
Enthalpy (H)= -1995.9478 0.76775
Gibbs Free Energy (G)= -1996.0618 0.65371

Frequencies -- -1210.8386 15.8932 23.4710

183.15K thermal correction = 0.691925
Single point SCF = -1997.185500

TS-C: Acylated-HBTM deprotonation *via N,N*-diisopropylethylamine (making Z)

Using Gaussian 09: AM64L-G09RevD.01 24-Apr-2013
==
# pbepbe/6-31G(d)/auto scf=(maxcycle=300,direct,tight,xqc) density=current
opt=(maxcycle=250,modredundant) SCRF=(PCM,SOLVENT=Dichloromethane)
iop(1/8=18) Temperature=298.15
Modredundant Input: B 53 59 F
Modredundant Input: B 24 53 F
Modredundant Input:
# pbepbe/6-31G(d)/auto scf=(direct,tight,maxcycle=300,xqc)
opt=(nofreeze,maxcycle=250,ts,calcfc,noeigentest) iop(1/8=18) freq=noraman
SCRF=(PCM,SOLVENT=Dichloromethane) Temperature=298.15 geom=check
guess=read
#N Geom=AllCheck Guess=TCheck SCRF=Check Test GenChk RPBEPBE/6-31G(d)/Auto
Freq

Pointgroup= C1 Stoichiometry= C35H46N3OS(1+)
C1[X(C35H46N3OS)] #Atoms= 86
Charge = 1 Multiplicity = 1

SCF Energy= -1996.70507884 Predicted Change= -1.261661D-10
==
Optimization completed. {Found 3 times}
Item Max Val. Criteria Pass? RMS Val. Criteria Pass?
Force 0.00000 || 0.00045 [YES] 0.00000 || 0.00030 [YES]
Displ 0.00029 || 0.00180 [YES] 0.00029 || 0.00180 [YES]

Atomic Type	X	Y	Z
C	6.076180	-2.494441	0.389368
C	5.217560	-1.419834	0.649752
C	3.897482	-1.516593	0.182999
C	3.453070	-2.644803	-0.528606
C	4.316662	-3.717943	-0.784423
C	5.633804	-3.630776	-0.316393
N	2.872738	-0.565546	0.343208
C	1.693880	-0.909790	-0.231668
S	1.758237	-2.471359	-1.028747
C	3.088346	0.696178	1.071195
C	1.751608	1.294973	1.497997
C	1.930520	2.682355	2.178125
C	0.628170	3.150778	2.851197
C	3.066196	2.671113	3.221542
C	0.786007	1.309618	0.276175
C	1.181443	2.244772	-0.865626
N	0.598758	-0.098297	-0.198528
C	2.117535	1.897215	-1.860695
C	2.471786	2.813767	-2.861593
C	1.895788	4.091907	-2.886352
C	0.952703	4.443852	-1.910083
C	0.594595	3.526147	-0.913123
C	-0.614910	-0.575151	-0.852171
C	-1.927061	0.046326	-0.772686
C	-2.327142	1.441364	-0.394266
O	-0.474946	-1.670217	-1.452771
C	-2.416608	1.906700	0.940019
C	-2.895596	3.192854	1.236613
C	-3.321433	4.046632	0.207955
C	-3.266528	3.595805	-1.120135
C	-2.777841	2.315588	-1.412344
H	7.110591	-2.444539	0.741148
H	5.574403	-0.539544	1.189947
H	3.971462	-4.596983	-1.336681
H	6.327161	-4.455444	-0.505126
H	3.653718	1.384971	0.415846
H	3.707994	0.459208	1.947741
H	0.782224	4.134990	3.325090
H	0.318540	2.443300	3.642595
H	-0.211474	3.255241	2.145038
H	2.200651	3.412185	1.390746
H	3.103532	3.644828	3.738418
H	4.060076	2.506446	2.772326
H	2.899881	1.893351	3.989633
H	1.297353	0.610184	2.242303
H	-0.197782	1.622119	0.643047
H	2.570734	0.900789	-1.882655
H	3.198206	2.521541	-3.626558
H	2.172123	4.805479	-3.669154
H	0.481721	5.431898	-1.929013
H	-0.165162	3.802707	-0.174150
H	-2.384525	-0.206224	-1.743107
H	-2.746683	-0.976828	-0.061005
H	-2.126226	1.247518	1.764662
H	-2.951032	3.518970	2.280864
H	-3.703533	5.045858	0.440098
H	-3.602554	4.245396	-1.935453
H	-2.738839	1.978217	-2.454626
N	-3.503119	-1.982143	0.394939
C	-3.571099	-2.993552	-0.752233
C	-4.277248	-2.418282	-1.988236
C	-4.230795	-4.329194	-0.365001
C	-2.785605	-2.546783	1.622008
C	-2.414148	-1.418588	2.597133
C	-1.527227	-3.361533	1.291037
C	-4.888686	-1.513362	0.793130
C	-5.305310	-0.144726	0.255978
H	-3.843776	-1.465470	-2.326555
H	-5.357466	-2.272993	-1.820167
H	-4.168536	-3.142278	-2.812753
H	-5.320692	-4.229265	-0.224562
H	-3.809627	-4.783010	0.545063
H	-4.076072	-5.041926	-1.192377
H	-2.517275	-3.164946	-1.020124
H	-2.119704	-1.864323	3.561349
H	-3.241954	-0.719269	2.795672
H	-1.552694	-0.842319	2.219155
H	-1.741698	-4.310905	0.777943
H	-1.022749	-3.609385	2.239696
H	-0.821466	-2.790363	0.668403
H	-3.515217	-3.214430	2.118162
H	-5.247549	-0.083641	-0.842063
H	-4.691627	0.665421	0.678104
H	-6.354398	0.034878	0.547137

H	-4.938337	-1.499861	1.892542
H	-5.609183	-2.281718	0.468649

Statistical Thermodynamic Analysis
Temperature= 298.150 Kelvin Pressure= 1.00000 Atm

==

SCF Energy= -1996.70507884 Predicted Change= -1.261661D-10
Zero-point correction (ZPE)= -1995.9787 0.72635
Internal Energy (U)= -1995.9384 0.76663
Enthalpy (H)= -1995.9374 0.76758
Gibbs Free Energy (G)= -1996.0507 0.65431

Frequencies -- -1279.9778 14.3501 26.0309

183.15K thermal correction = 0.692230
Single point SCF = -1997.174801

D: (Z)-enolate (S•••O anti)

Using Gaussian 09: AM64L-G09RevD.01 24-Apr-2013

==

# pbepbe/6-31G*/auto gfprint gfinput scf=(direct,tight,maxcycle=300,xqc)
opt=(maxcycle=250) freq=noraman iop(1/8=18) Temperature=298.15
SCRF=(PCM,SOLVENT=Dichloromethane)
#N Geom=AllCheck Guess=TCheck SCRF=Check Test GenChk RPBEPBE/6-31G(d)/Auto
Freq

Pointgroup= C1 Stoichiometry= C27H26N2OS
C1[X(C27H26N2OS)] #Atoms= 57
Charge = 0 Multiplicity = 1

SCF Energy= -1625.74017858 Predicted Change= -9.818437D-09

==

Optimization completed. {Found 2 times}

Item	Max Val.	Criteria	Pass?	RMS Val.	Criteria	Pass?
Force	0.00002 ||	0.00045	[YES]	0.00000 ||	0.00030	[YES]
Displ	0.00072 ||	0.00180	[YES]	0.00072 ||	0.00180	[YES]

Atomic Type	Coordinates (Angstroms) X	Y	Z
C	3.325906	4.502535	0.725783
C	3.258182	3.122419	0.485897
C	1.995632	2.553819	0.265785
C	0.830612	3.347632	0.294630
C	0.901861	4.722391	0.538870
C	2.165086	5.295377	0.752084
N	1.707695	1.204108	0.002049
C	0.379238	0.935375	-0.170306
S	-0.634152	2.368173	0.027785
C	2.720256	0.153381	-0.187011
C	2.189336	-0.884946	-1.182733
C	3.221889	-2.008092	-1.511532
C	4.144192	-1.561405	-2.662427
C	4.064805	-2.485391	-0.313388
C	0.799201	-1.410562	-0.729349
C	0.783677	-2.387723	0.440817
N	-0.103706	-0.264891	-0.462248
C	0.693981	-3.768146	0.175846
C	0.723799	-4.703307	1.220050
C	0.829496	-4.268479	2.549407
C	0.895167	-2.894508	2.827005
C	0.868498	-1.960770	1.781154
C	-1.578618	-0.457088	-0.894925
C	-2.475013	-0.007041	0.070580
C	-3.920160	0.037212	-0.075666
O	-1.704029	-0.987529	-2.020259
C	-4.606119	-0.337215	-1.265439
C	-6.001655	-0.271101	-1.345872
C	-6.770626	0.166298	-0.253609
C	-6.112336	0.539052	0.930918
C	-4.717856	0.476577	1.017539
H	4.302840	4.963681	0.898290
H	4.166005	2.513590	0.477815
H	-0.004403	5.334868	0.561596
H	2.242272	6.369664	0.943288
H	2.959707	-0.296750	0.792334
H	3.631128	0.633357	-0.578003
H	4.749037	-0.680646	-2.377854
H	3.567073	-1.299824	-3.566308
H	4.846566	-2.369768	-2.929318
H	2.636766	-2.874087	-1.876866
H	4.748129	-1.692989	0.041776
H	4.692766	-3.338885	-0.621658
H	3.445335	-2.813936	0.536304
H	1.988989	-0.343116	-2.127373
H	0.334301	-1.902214	-1.602739
H	0.596418	-4.113011	-0.859958
H	0.653102	-5.772217	0.993899
H	0.847232	-4.996089	3.367321
H	0.959977	-2.545395	3.862757
H	0.895616	-0.891465	2.018491
H	-2.071425	0.257651	1.053152
H	-4.015823	-0.685120	-2.117735
H	-6.498068	-0.567937	-2.277577
H	-7.862611	0.214069	-0.323027
H	-6.691699	0.882080	1.796206
H	-4.219072	0.771661	1.949434

Statistical Thermodynamic Analysis
Temperature= 298.150 Kelvin Pressure= 1.00000 Atm

==

SCF Energy= -1625.74017858 Predicted Change= -9.818437D-09
Zero-point correction (ZPE)= -1625.2830 0.45711
Internal Energy (U)= -1625.2555 0.48463
Enthalpy (H)= -1625.2546 0.48557
Gibbs Free Energy (G)= -1625.3420 0.39808

Frequencies -- 16.6347 23.4095 38.7354

183.15K thermal correction = 0.427861
Single point SCF = -1626.119655

D: (E)-enolate (S•••O syn)

Using Gaussian 09: AM64L-G09RevD.01 24-Apr-2013

==

pbepbe/6-31G*/auto gfprint gfinput scf=(direct,tight,maxcycle=300,xqc)

opt=(maxcycle=250) freq=noraman iop(1/8=18)
Temperature=298.15
SCRF=(PCM,SOLVENT=Dichloromethane)
#N Geom=AllCheck Guess=TCheck SCRF=Check Test GenChk RPBEPBE/6-31G(d)/Auto
Freq

Pointgroup= C1 Stoichiometry= C27H26N2OS
C1[X(C27H26N2OS)] #Atoms= 57
Charge = 0 Multiplicity = 1

SCF Energy= -1625.73165731 Predicted Change= -1.139425D-08
==
Optimization completed. {Found 2 times}

Item	Max Val.	Criteria	Pass?	RMS Val.	Criteria	Pass?
Force	0.00002 ||	0.00045	[YES]	0.00000 ||	0.00030	[YES]
Displ	0.00117 ||	0.00180	[YES]	0.00117 ||	0.00180	[YES]

Atomic Type	X	Y	Z
C	5.565261	-0.165844	1.313455
C	4.239046	0.277220	1.416945
C	3.287991	-0.296691	0.560811
C	3.653339	-1.277096	-0.383067
C	4.980122	-1.710079	-0.485333
C	5.933686	-1.149058	0.377349
N	1.911323	-0.014942	0.515793
C	1.227716	-0.718812	-0.430038
S	2.271302	-1.754245	-1.400349
C	1.258907	0.977957	1.384791
C	-0.242124	0.695891	1.471328
C	-1.018679	1.739403	2.332913
C	-0.973519	1.337183	3.819987
C	-0.558214	3.199239	2.161413
C	-0.843301	0.467415	0.056519
C	-0.938830	1.680655	-0.861825
N	-0.098439	-0.650706	-0.589655
C	-2.188960	2.315128	-1.006201
C	-2.326750	3.460719	-1.802193
C	-1.215600	3.982144	-2.481181
C	0.029124	3.345839	-2.363982
C	0.166832	2.203230	-1.562716
C	-0.717220	-1.442815	-1.715889
C	-2.058617	-1.809557	-1.616548
C	-2.963115	-1.938193	-0.487852
O	0.078891	-1.664133	-2.675464
C	-2.550443	-2.100377	0.867833
C	-3.477896	-2.238785	1.908187
C	-4.857323	-2.254134	1.644875
C	-5.290481	-2.136896	0.311667
C	-4.368396	-1.982440	-0.726825
H	6.322708	0.266607	1.973855
H	3.964490	1.047925	2.141908
H	5.265822	-2.468372	-1.220710
H	6.974788	-1.479484	0.315941
H	1.465807	1.982846	0.974833
H	1.725003	0.910401	2.381023
H	0.062472	1.330941	4.205886
H	-1.402353	0.332305	3.978854
H	-1.549252	2.052650	4.432083
H	-2.076115	1.679725	2.009454
H	0.448392	3.359939	2.588275
H	-1.248468	3.869600	2.701556
H	-0.540506	3.515654	1.106120
H	-0.359846	-0.283120	1.974847
H	-1.861860	0.085892	0.216681
H	-3.064599	1.902614	-0.491912
H	-3.307096	3.938108	-1.900960
H	-1.321069	4.872901	-3.108951
H	0.898800	3.734926	-2.903454
H	1.143996	1.712051	-1.507226
H	-2.456598	-2.156053	-2.578019
H	-1.479500	-2.158811	1.093856
H	-3.114553	-2.361025	2.935541
H	-5.580095	-2.371409	2.458850
H	-6.361853	-2.160393	0.080453
H	-4.726016	-1.883378	-1.758989

Statistical Thermodynamic Analysis
Temperature= 298.150 Kelvin Pressure= 1.00000 Atm
==
SCF Energy= -1625.73165731 Predicted Change= -1.139425D-08
Zero-point correction (ZPE)= -1625.2742 0.45739
Internal Energy (U)= -1625.2469 0.48470
Enthalpy (H)= -1625.2460 0.48565
Gibbs Free Energy (G)= -1625.3325 0.39907

Frequencies -- 17.0245 29.5215 35.8084

183.15K thermal correction = 0.428512
Single point SCF = -1626.111285

D: (E)-enolate (S•••O anti)

Using Gaussian 09: AM64L-G09RevD.01 24-Apr-2013
==
# pbepbe/6-31G*/auto gfprint gfinput scf=(direct,tight,maxcycle=300,xqc)
opt=(maxcycle=250) freq=noraman iop(1/8=18)
Temperature=298.15
SCRF=(PCM,SOLVENT=Dichloromethane)
#N Geom=AllCheck Guess=TCheck SCRF=Check Test GenChk RPBEPBE/6-31G(d)/Auto
Freq

Pointgroup= C1 Stoichiometry= C27H26N2OS
C1[X(C27H26N2OS)] #Atoms= 57
Charge = 0 Multiplicity = 1

SCF Energy= -1625.72956714 Predicted Change= -5.417843D-08
==
Optimization completed. {Found 1 times}

Item	Max Val.	Criteria	Pass?	RMS Val.	Criteria	Pass?
Force	0.00004 ||	0.00045	[YES]	0.00000 ||	0.00030	[YES]
Displ	0.00213 ||	0.00180	[NO]	0.00213 ||	0.00180	[YES]

Atomic Type	X	Y	Z
C	1.598162	4.740841	-1.573161
C	0.545178	3.858210	-1.293867
C	0.832157	2.710637	-0.540827
C	2.140626	2.450918	-0.084525

C	3.188125	3.334149	-0.366490
C	2.903757	4.484950	-1.116693
N	-0.070589	1.708618	-0.147831
C	0.495871	0.692948	0.570485
S	2.223340	0.927807	0.828724
C	-1.530840	1.808132	-0.310299
C	-2.215630	1.040390	0.824703
C	-3.772396	1.130185	0.783915
C	-4.247377	2.404842	1.508185
C	-4.397221	1.059316	-0.622718
C	-1.657891	-0.407131	0.908485
C	-2.106177	-1.363053	-0.188984
N	-0.173601	-0.362128	1.030898
C	-3.105550	-2.310356	0.107458
C	-3.596746	-3.173937	-0.882229
C	-3.084155	-3.112155	-2.186201
C	-2.074094	-2.186981	-2.490691
C	-1.588317	-1.320087	-1.500802
C	0.430140	-1.150595	2.228626
C	1.651819	-1.786887	2.007466
C	2.327644	-2.245005	0.800517
O	-0.248893	-1.030951	3.271119
C	3.701203	-2.611791	0.892142
C	4.416658	-3.083325	-0.213093
C	3.794438	-3.209618	-1.466905
C	2.436060	-2.869002	-1.579047
C	1.714397	-2.407537	-0.472235
H	1.394877	5.641170	-2.160446
H	-0.464352	4.059578	-1.662140
H	4.202724	3.131145	-0.011092
H	3.709749	5.187094	-1.349206
H	-1.813832	1.420628	-1.304229
H	-1.794541	2.877101	-0.270370
H	-3.885412	3.316758	0.998441
H	-3.890885	2.434545	2.552427
H	-5.349957	2.452645	1.523877
H	-4.148605	0.263722	1.362049
H	-4.151848	1.955343	-1.221367
H	-5.496557	1.022266	-0.534272
H	-4.075280	0.169269	-1.186378
H	-1.888809	1.512450	1.771899
H	-1.975836	-0.813133	1.884645
H	-3.503280	-2.370849	1.126951
H	-4.374302	-3.902278	-0.629943
H	-3.461579	-3.788703	-2.959768
H	-1.657158	-2.140406	-3.502076
H	-0.784436	-0.620873	-1.755552
H	2.137715	-2.074131	2.948691
H	4.206455	-2.514189	1.861058
H	5.472694	-3.353283	-0.094909
H	4.352766	-3.578010	-2.333877
H	1.920260	-2.982204	-2.540232
H	0.647432	-2.196735	-0.583481

Statistical Thermodynamic Analysis
Temperature= 298.150 Kelvin Pressure= 1.00000 Atm
==
==============================

SCF Energy= -1625.72956714 Predicted Change= -5.417843D-08
Zero-point correction (ZPE)= -1625.2724 0.45710
Internal Energy (U)= -1625.2450 0.48452
Enthalpy (H)= -1625.2440 0.48547
Gibbs Free Energy (G)= -1625.3308 0.39869

Frequencies -- 20.1716 28.7155 29.4143

183.15K thermal correction = 0.428198

Single point SCF = -1626.109982

D: (Z)-enolate/ketone complex

Using Gaussian 09: AM64L-G09RevD.01 24-Apr-2013
==
==============================

# pbepbe/6-31G*/auto gfprint gfinput scf=(direct,tight,maxcycle=300,xqc)
opt=(maxcycle=250) freq=noraman iop(1/8=18) Temperature=298.15
SCRF=(PCM,SOLVENT=Dichloromethane)
#N Geom=AllCheck Guess=TCheck SCRF=Check Test GenChk RPBEPBE/6-31G(d)/Auto
Freq

Pointgroup= C1 Stoichiometry= C35H30BrF3N2O2S
C1[X(C35H30BrF3N2O2S)] #Atoms= 74
Charge = 0 Multiplicity = 1

SCF Energy= -4878.34580530 Predicted Change= -4.200142D-08
==
==============================

Optimization completed. {Found 1 times}

Item	Max Val.	Criteria	Pass?	RMS Val.	Criteria	Pass?
Force	0.00001 ||	0.00045	[YES]	0.00000 ||	0.00030	[YES]
Displ	0.00376 ||	0.00180	[NO]	0.00376 ||	0.00180	[YES]

Atomic Type	Coordinates (Angstroms) X	Y	Z
N	-3.250129	1.248506	-0.287598
C	-3.994022	0.310289	0.565179
C	-3.022709	-0.658400	1.240940
C	-3.758886	-1.719924	2.106819
C	-4.817924	-1.075377	3.024632
C	-2.774886	-2.536927	2.966229
C	-2.081544	-1.277508	0.163597
C	-2.756011	-2.245954	-0.807564
N	-1.377383	-0.185171	-0.563386
C	0.003248	-0.354691	-1.105668
C	0.552498	-1.632998	-1.039156
C	1.893988	-1.996979	-1.453908
O	0.499398	0.716325	-1.576114
C	-1.991656	0.991677	-0.743763
S	-1.295706	2.346169	-1.632725
C	-2.818001	3.247751	-1.401083
C	-3.753377	2.509044	-0.653293
C	-3.127060	4.535705	-1.853998
C	-4.390967	5.065306	-1.552864
C	-5.324760	4.317929	-0.812173
C	-5.020933	3.028542	-0.351908
C	-3.444447	-1.798734	-1.952981
C	-4.081936	-2.709889	-2.807394
C	-4.039594	-4.084811	-2.534576
C	-3.346274	-4.543599	-1.405279
C	-2.707472	-3.631677	-0.553281
C	2.874491	-1.075631	-1.921576
C	4.165376	-1.499466	-2.257505
C	4.531718	-2.853202	-2.164543
C	3.575040	-3.783116	-1.720820
C	2.288788	-3.363801	-1.369486
H	-4.745833	-0.223003	-0.046833
H	-4.523392	0.913496	1.317157
H	-4.282294	-2.412946	1.420152

H	-5.260918	-1.843834	3.680742
H	-5.645875	-0.613404	2.460654
H	-4.367728	-0.301962	3.673547
H	-3.323539	-3.300616	3.543424
H	-2.242989	-1.886748	3.681222
H	-2.011986	-3.062318	2.368369
H	-2.371332	-0.064108	1.910930
H	-1.282822	-1.803941	0.707750
H	-0.067735	-2.463180	-0.697529
H	-2.399471	5.114587	-2.431547
H	-4.651108	6.069226	-1.901508
H	-6.308082	4.743244	-0.590379
H	-5.756854	2.450797	0.213702
H	-3.468323	-0.733268	-2.204613
H	-4.606993	-2.340555	-3.694262
H	-4.534313	-4.795315	-3.204593
H	-3.291474	-5.615507	-1.189319
H	-2.155392	-4.005616	0.316215
H	2.602252	-0.020373	-1.997158
H	4.904032	-0.753836	-2.575650
H	5.543226	-3.178201	-2.430800
H	3.836290	-4.845057	-1.642228
H	1.558173	-4.101556	-1.013610
C	0.940544	0.894683	2.222568
C	2.289392	0.963729	1.625009
C	0.643882	-0.217435	3.276701
F	1.560191	-0.227759	4.280959
F	0.652866	-1.450145	2.692629
F	-0.573277	-0.027315	3.834392
O	0.017021	1.660531	1.940986
C	3.319503	0.035981	1.906987
C	4.563228	0.147674	1.281393
C	4.778528	1.196007	0.374936
Br	6.456511	1.318530	-0.520646
C	3.778876	2.136850	0.084835
C	2.536407	2.009584	0.705203
H	3.158203	-0.788494	2.604499
H	5.354355	-0.577963	1.484382
H	3.968604	2.942896	-0.628411
H	1.731655	2.715292	0.484736

Statistical Thermodynamic Analysis
Temperature= 298.150 Kelvin Pressure= 1.00000 Atm

==
=============================

SCF Energy= -4878.34580530 Predicted Change= -4.200142D-08
Zero-point correction (ZPE)= -4877.7855 0.56025
Internal Energy (U)= -4877.7445 0.60125
Enthalpy (H)= -4877.7436 0.60220
Gibbs Free Energy (G)= -4877.8637 0.48208

Frequencies -- 14.3572 19.8108 21.7110

183.15K thermal correction = 0.522695
Single point SCF = -4881.375311

F: Product-catalyst complex (Re,Re)

Using Gaussian 09: AM64L-G09RevD.01 24-Apr-2013

==
=============================

# pbepbe/6-31G*/auto gfprint gfinput scf=(direct,tight,maxcycle=300,xqc)
opt=(maxcycle=250) freq=noraman iop(1/8=18) Temperature=298.15
SCRF=(PCM,SOLVENT=Dichloromethane)
#N Geom=AllCheck Guess=TCheck SCRF=Check Test GenChk RPBEPBE/6-31G(d)/Auto
Freq

Pointgroup= C1 Stoichiometry= C35H30BrF3N2O2S
C1[X(C35H30BrF3N2O2S)] #Atoms= 74
Charge = 0 Multiplicity = 1

SCF Energy= -4878.33998277 Predicted Change= -7.628162D-08

==
=============================

Optimization completed. {Found 1 times}

Item	Max Val.	Criteria	Pass?	RMS Val.	Criteria	Pass?
Force	0.00004 ||	0.00045	[YES]	0.00000 ||	0.00030	[YES]
Displ	0.00499 ||	0.00180	[NO]	0.00499 ||	0.00180	[YES]

Atomic Type	Coordinates (Angstroms) X	Y	Z
N	3.550230	-1.275449	-0.244693
C	2.887638	-2.541433	0.101388
C	1.426980	-2.508201	-0.357373
C	0.660760	-3.804860	0.023985
C	1.467888	-5.074660	-0.313879
C	-0.707323	-3.871045	-0.679722
C	0.764756	-1.195064	0.166266
C	0.571612	-1.146189	1.683455
N	1.537052	-0.030803	-0.331347
C	0.830031	1.291490	-0.790996
C	-0.391166	1.695999	0.131893
C	-0.363119	2.974009	0.934961
O	1.681867	2.139589	-1.189483
C	2.852427	-0.134605	-0.511440
S	3.884605	1.175975	-1.094728
C	5.305035	0.114823	-0.881301
C	4.940925	-1.163546	-0.421168
C	6.647034	0.423555	-1.131900
C	7.615582	-0.565959	-0.905119
C	7.244893	-1.839484	-0.436038
C	5.902034	-2.157210	-0.185768
C	1.596146	-0.736494	2.560175
C	1.390180	-0.723539	3.947568
C	0.154367	-1.113961	4.483224
C	-0.878338	-1.510991	3.620846
C	-0.671693	-1.522968	2.233967
C	0.208564	4.178230	0.469832
C	0.207488	5.324684	1.276922
C	-0.364927	5.298239	2.558016
C	-0.926544	4.105738	3.037253
C	-0.916511	2.956855	2.234256
H	-0.622256	0.879874	0.834331
H	2.967489	-2.712900	1.191331
H	3.437478	-3.341556	-0.416197
H	0.492785	-3.792163	1.118219
H	0.853420	-5.969993	-0.118868
H	2.383640	-5.170720	0.293361
H	1.756179	-5.094463	-1.381066
H	-1.244551	-4.787585	-0.381522
H	-0.580830	-3.897922	-1.777663
H	-1.359539	-3.015089	-0.440930
H	1.426633	-2.428863	-1.462885
H	-0.217540	-1.102856	-0.325416
H	6.933355	1.415563	-1.495380
H	8.669688	-0.341177	-1.093582
H	8.012897	-2.598238	-0.258461
H	5.623118	-3.145404	0.190013

H	2.559335	-0.397364	2.165117
H	2.198680	-0.397009	4.609587
H	-0.006726	-1.098317	5.565961
H	-1.852672	-1.803862	4.025326
H	-1.493589	-1.813404	1.569852
H	0.671732	4.199204	-0.517363
H	0.659972	6.248432	0.899697
H	-0.366195	6.198938	3.181295
H	-1.365583	4.065389	4.039898
H	-1.338956	2.022281	2.623836
C	-1.287229	1.429698	-1.126105
C	-1.761897	2.668430	-1.922092
F	-2.627421	3.429372	-1.190507
F	-2.417861	2.281928	-3.054194
F	-0.743346	3.469458	-2.316559
C	-2.492817	0.524746	-0.892483
O	-0.224962	0.784515	-1.844131
C	-2.684220	-0.602554	-1.710125
C	-3.788236	-1.447035	-1.524439
C	-4.706429	-1.146449	-0.510885
Br	-6.216582	-2.295366	-0.243082
C	-4.543923	-0.025190	0.313481
C	-3.436109	0.809592	0.113225
H	-1.952256	-0.821400	-2.493050
H	-3.932205	-2.324387	-2.160580
H	-5.270292	0.193496	1.100719
H	-3.308224	1.689901	0.751199

Statistical Thermodynamic Analysis
Temperature= 298.150 Kelvin Pressure= 1.00000 Atm
==
==============================
SCF Energy= -4878.33998277 Predicted Change= -7.628162D-08
Zero-point correction (ZPE)= -4877.7774 0.56258
Internal Energy (U)= -4877.7382 0.60177
Enthalpy (H)= -4877.7372 0.60271
Gibbs Free Energy (G)= -4877.8515 0.48847

Frequencies -- 10.0984 18.6472 27.7774

183.15K thermal correction = 0.526955
Single point SCF = -4881.365776

F: Product-catalyst complex (Si,Re)

Using Gaussian 09: AM64L-G09RevD.01 24-Apr-2013
==
==============================
# pbepbe/6-31G*/auto gfprint gfinput scf=(direct,tight,maxcycle=300,xqc)
opt=(maxcycle=250) freq=noraman iop(1/8=18) Temperature=298.15
SCRF=(PCM,SOLVENT=Dichloromethane)
#N Geom=AllCheck Guess=TCheck SCRF=Check Test GenChk RPBEPBE/6-31G(d)/Auto
Freq

Pointgroup= C1 Stoichiometry= C35H30BrF3N2O2S C1[X(C35H30BrF3N2O2S)] #Atoms= 74
Charge = 0 Multiplicity = 1

SCF Energy= -4878.34073625 Predicted Change= -1.090354D-08
==
==============================
Optimization completed. {Found 2 times}

Item	Max Val.	Criteria	Pass?	RMS Val.	Criteria	Pass?
Force	0.00002 ||	0.00045	[YES]	0.00000 ||	0.00030	[YES]
Displ	0.00120 ||	0.00180	[YES]	0.00120 ||	0.00180	[YES]

Atomic Type	Coordinates (Angstroms) X	Y	Z
N	-3.744492	0.961484	-0.271830
C	-4.414822	-0.192925	0.348477
C	-3.408492	-1.030003	1.145702
C	-4.041785	-2.322445	1.729339
C	-5.407106	-2.052963	2.391418
C	-3.100915	-2.985133	2.753467
C	-2.147611	-1.288005	0.265727
C	-2.371656	-2.214648	-0.931377
N	-1.577678	0.015212	-0.143115
C	-0.031253	0.272549	-0.205892
C	0.674406	0.091114	1.211646
C	1.262455	1.274515	1.926618
O	0.228174	1.260590	-0.958411
C	-2.392851	1.020160	-0.448135
S	-1.857536	2.559874	-1.129104
C	-3.552420	3.115817	-1.137584
C	-4.432107	2.123780	-0.665048
C	-4.042046	4.356708	-1.560967
C	-5.425758	4.583550	-1.508481
C	-6.300051	3.585420	-1.041699
C	-5.816459	2.340288	-0.614179
C	-2.977337	-1.782775	-2.128556
C	-3.199508	-2.679291	-3.184294
C	-2.817413	-4.023015	-3.064090
C	-2.196448	-4.460533	-1.885059
C	-1.970623	-3.562271	-0.833036
C	1.953384	2.304690	1.250902
C	2.474681	3.395873	1.960397
C	2.327047	3.476806	3.353828
C	1.645035	2.458913	4.037716
C	1.116761	1.372330	3.327050
C	1.529219	-0.995267	0.469493
C	2.924565	-0.591523	-0.007116
C	1.614543	-2.344185	1.215808
F	2.309343	-2.227473	2.383511
F	2.220647	-3.296121	0.464449
F	0.381487	-2.838597	1.555855
O	0.580349	-1.093391	-0.608023
C	3.167090	-0.487349	-1.387148
C	4.432486	-0.132600	-1.873608
C	5.461315	0.119945	-0.959038
Br	7.198245	0.608074	-1.609152
C	5.248529	0.029053	0.421798
C	3.977212	-0.326449	0.891721
H	-0.001244	-0.438864	1.905196
H	-4.902815	-0.793403	-0.442288
H	-5.197417	0.208449	1.009844
H	-4.202384	-3.028805	0.892239
H	-5.781915	-2.975934	2.865612
H	-6.172895	-1.725219	1.668187
H	-5.324831	-1.282930	3.180541
H	-3.551936	-3.915391	3.138970
H	-2.929479	-2.313741	3.614957
H	-2.116414	-3.247151	2.332339
H	-3.063450	-0.410085	1.998001
H	-1.379726	-1.748967	0.897643
H	-3.360481	5.131254	-1.926199
H	-5.826078	5.547178	-1.837528
H	-7.376876	3.776508	-1.011455
H	-6.503004	1.566701	-0.260178

H	-3.259982	-0.733676	-2.262797
H	-3.667167	-2.320132	-4.106895
H	-2.989506	-4.721253	-3.889683
H	-1.872504	-5.501824	-1.786417
H	-1.451307	-3.905939	0.067379
H	2.049397	2.249216	0.164440
H	3.002687	4.188724	1.419337
H	2.740057	4.329272	3.903873
H	1.521131	2.511348	5.124735
H	0.581823	0.580927	3.866312
H	2.341546	-0.676296	-2.077429
H	4.613867	-0.053289	-2.948943
H	6.060913	0.237576	1.123041
H	3.813310	-0.390364	1.970094

Statistical Thermodynamic Analysis
Temperature= 298.150 Kelvin   Pressure= 1.00000 Atm
==
==============================
SCF Energy=   -4878.34073625   Predicted Change= -1.090354D-08
Zero-point correction (ZPE)=   -4877.7779   0.56277
Internal Energy (U)=   -4877.7387   0.60195
Enthalpy (H)=   -4877.7378   0.60289
Gibbs Free Energy (G)=   -4877.8524   0.48833

Frequencies --   12.7730   18.4261   22.6933

183.15K thermal correction = 0.526942
Single point SCF = -4881.365054

F: Product-catalyst complex (Si,Si)

Using Gaussian 09: AM64L-G09RevD.01 24-Apr-2013
==
==============================
# pbepbe/6-31G*/auto gfprint gfinput scf=(direct,tight,maxcycle=300,xqc)
opt=(maxcycle=250) freq=noraman iop(1/8=18) Temperature=298.15
SCRF=(PCM,SOLVENT=Dichloromethane)
#N Geom=AllCheck Guess=TCheck SCRF=Check Test GenChk RPBEPBE/6-31G(d)/Auto
Freq

Pointgroup= C1   Stoichiometry= C35H30BrF3N2O2S
C1[X(C35H30BrF3N2O2S)] #Atoms= 74
Charge = 0   Multiplicity = 1

SCF Energy= -4878.33758727   Predicted Change= -8.545895D-09
==
==============================
Optimization completed.   {Found   2   times}

Item	Max Val.	Criteria	Pass?	RMS Val.	Criteria	Pass?
Force	0.00001 ||	0.00045	[YES]	0.00000 ||	0.00030	[YES]
Displ	0.00080 ||	0.00180	[YES]	0.00080 ||	0.00180	[YES]

Atomic Type	Coordinates (Angstroms) X	Y	Z
N	-3.404563	-1.145707	0.495265
C	-2.675983	-2.227174	1.180160
C	-1.217924	-1.816127	1.410021
C	-0.377658	-2.938338	2.077769
C	-1.095254	-3.542973	3.301037
C	1.005361	-2.412990	2.508454
C	-0.645315	-1.271742	0.062207
C	-0.551910	-2.314492	-1.056703
N	-1.449896	-0.097046	-0.332284
C	-0.804917	1.238409	-0.902941
C	0.233115	1.846064	0.139311
H	0.408910	1.128445	0.959505
C	0.012820	3.201842	0.763943
O	-1.685468	1.930209	-1.492517
C	-2.766315	-0.128555	-0.152072
S	-3.879444	1.112726	-0.739480
C	-5.238156	0.180893	-0.055232
C	-4.802722	-1.010459	0.554938
C	-6.598627	0.507959	-0.084488
C	-7.513511	-0.380954	0.500374
C	-7.072282	-1.573059	1.102691
C	-5.710339	-1.905984	1.138191
C	0.580395	-3.154188	-1.113192
C	0.694394	-4.149416	-2.093489
C	-0.322143	-4.320574	-3.044602
C	-1.445576	-3.483196	-3.008511
C	-1.558500	-2.487903	-2.025608
C	0.448347	3.411625	2.090723
C	0.275429	4.648378	2.727123
C	-0.356805	5.700978	2.048839
C	-0.813377	5.500507	0.737166
C	-0.629260	4.267118	0.096307
H	-2.745880	-3.150198	0.574506
H	-3.187883	-2.400574	2.138646
H	-0.233071	-3.748843	1.337622
H	-0.432339	-4.268257	3.802734
H	-2.018906	-4.080835	3.028536
H	-1.354915	-2.762307	4.039779
H	1.596715	-3.226660	2.961839
H	0.901277	-1.613320	3.264763
H	1.597755	-2.004685	1.671758
H	-1.221378	-0.952747	2.105818
H	0.370222	-0.888486	0.245831
H	-6.940596	1.434994	-0.555223
H	-8.581180	-0.142360	0.483859
H	-7.799028	-2.257487	1.550411
H	-5.374878	-2.838028	1.600968
H	1.393242	-3.017558	-0.392644
H	1.586070	-4.784548	-2.118230
H	-0.233843	-5.093477	-3.814974
H	-2.239697	-3.594937	-3.753999
H	-2.434011	-1.831866	-2.039097
H	0.928868	2.588202	2.633606
H	0.627297	4.785773	3.755213
H	-0.499045	6.668674	2.541772
H	-1.319693	6.313133	0.204640
H	-1.002620	4.106264	-0.915932
C	1.314597	1.525481	-0.948338
C	1.817706	2.718660	-1.793572
F	2.641220	2.282113	-2.788584
F	2.529672	3.599184	-1.031842
F	0.814751	3.407398	-2.388517
C	2.524619	0.729605	-0.472275
O	0.395278	0.743210	-1.733498
C	3.309125	1.168361	0.611587
C	4.423944	0.431324	1.033453
C	4.754579	-0.748417	0.353978
Br	6.274445	-1.764311	0.926459
C	3.997197	-1.201175	-0.733258
C	2.884904	-0.452136	-1.143092
H	3.052122	2.094018	1.136353
H	5.026819	0.770796	1.879740
H	4.271623	-2.121572	-1.255834

H 2.275777 -0.794721 -1.984002

Statistical Thermodynamic Analysis
Temperature= 298.150 Kelvin Pressure= 1.00000 Atm
==
SCF Energy= -4878.33758727 Predicted Change= -8.545895D-09
Zero-point correction (ZPE)= -4877.7750 0.56250
Internal Energy (U)= -4877.7359 0.60168
Enthalpy (H)= -4877.7349 0.60263
Gibbs Free Energy (G)= -4877.8491 0.48842

Frequencies -- 11.6007 15.8217 24.6120

183.15K thermal correction = 0.526891
Single point SCF = -4881.363540

TS-G: Catalyst release (Re,Re)

Using Gaussian 09: AM64L-G09RevD.01 24-Apr-2013
==
# pbepbe/6-31G(d)/auto scf=(maxcycle=300,direct,tight,xqc) density=current
opt=(maxcycle=250,modredundant) SCRF=(PCM,SOLVENT=Dichloromethane)
iop(1/8=18) Temperature=298.15
Modredundant Input: B 9 10 F
Modredundant Input:
# pbepbe/6-31G(d)/auto scf=(direct,tight,maxcycle=300,xqc)
opt=(nofreeze,maxcycle=250,ts,calcfc,noeigentest) iop(1/8=18) freq=noraman
SCRF=(PCM,SOLVENT=Dichloromethane) Temperature=298.15 geom=check
guess=read
#N Geom=AllCheck Guess=TCheck SCRF=Check Test GenChk RPBEPBE/6-31G(d)/Auto
Freq

Pointgroup= C1 Stoichiometry= C35H30BrF3N2O2S
C1[X(C35H30BrF3N2O2S)] #Atoms= 74
Charge = 0 Multiplicity = 1

SCF Energy= -4878.33760722 Predicted Change= -2.033138D-09
==
Optimization completed. {Found 3 times}
Item Max Val. Criteria Pass? RMS Val. Criteria Pass?
Force 0.00000 || 0.00045 [YES] 0.00000 || 0.00030 [YES]
Displ 0.00111 || 0.00180 [YES] 0.00111 || 0.00180 [YES]

Atomic Coordinates (Angstroms)

Type	X	Y	Z
N	-3.523270	-1.381010	0.194579
C	-2.842183	-2.683119	0.159327
C	-1.497875	-2.585409	0.890887
C	-0.702204	-3.918916	0.864954
C	-1.589908	-5.134233	1.201213
C	0.487309	-3.871961	1.842209
C	-0.717287	-1.348941	0.334273
C	-0.252578	-1.503250	-1.118360
N	-1.525901	-0.133979	0.525538
C	-0.783785	1.485615	1.086705
C	0.245870	1.883040	-0.022395
C	0.107834	3.170857	-0.796107
O	-1.716565	2.145498	1.560764
C	-2.830259	-0.219314	0.448399
S	-3.941823	1.168171	0.611362
C	-5.312779	0.112175	0.215909
C	-4.906617	-1.225896	0.036016
C	-6.660183	0.471038	0.106791
C	-7.597793	-0.528735	-0.198557
C	-7.189133	-1.860583	-0.384785
C	-5.839251	-2.227288	-0.271267
C	-1.062961	-1.133475	-2.211484
C	-0.616282	-1.309122	-3.529946
C	0.652161	-1.852257	-3.781411
C	1.474358	-2.211685	-2.703154
C	1.026214	-2.034896	-1.386552
C	-0.491222	4.331254	-0.262006
C	-0.605501	5.493658	-1.037287
C	-0.124362	5.523455	-2.355117
C	0.459801	4.371913	-2.903106
C	0.564205	3.206683	-2.131587
H	0.348232	1.056310	-0.742721
H	-2.708461	-3.003230	-0.891796
H	-3.503860	-3.407202	0.658802
H	-0.308451	-4.062273	-0.159870
H	-0.970984	-6.045851	1.261275
H	-2.365262	-5.317202	0.438231
H	-2.091042	-5.007010	2.178648
H	1.054969	-4.817227	1.798559
H	0.134126	-3.739424	2.881271
H	1.192504	-3.053205	1.621560
H	-1.717088	-2.348563	1.951478
H	0.173496	-1.196568	0.966810
H	-6.975503	1.508699	0.253075
H	-8.655020	-0.263492	-0.292962
H	-7.930301	-2.628792	-0.625185
H	-5.527731	-3.263950	-0.426080
H	-2.044777	-0.680662	-2.040356
H	-1.262350	-1.011412	-4.362425
H	1.002052	-1.983480	-4.810581
H	2.475320	-2.618468	-2.881752
H	1.690350	-2.295464	-0.555557
H	-0.885782	4.309367	0.755250
H	-1.077910	6.383381	-0.607094
H	-0.213779	6.435480	-2.954795
H	0.825185	4.375433	-3.935495
H	0.998701	2.302256	-2.574755
C	1.314381	1.610011	1.098217
C	1.878472	2.858086	1.814229
F	2.658649	3.591457	0.972600
F	2.648310	2.492292	2.876284
F	0.905253	3.670932	2.294148
C	2.465126	0.684615	0.742696
O	0.321825	0.996285	1.960543
C	2.806939	-0.377296	1.599375
C	3.867465	-1.238730	1.283877
C	4.591720	-1.018035	0.105101
Br	6.023255	-2.204922	-0.350809
C	4.286642	0.047470	-0.751245
C	3.223975	0.898893	-0.422047
H	2.231668	-0.535660	2.516455
H	4.126969	-2.067789	1.947537
H	4.865881	0.208338	-1.664146
H	2.984423	1.734016	-1.088029

Statistical Thermodynamic Analysis
Temperature= 298.150 Kelvin Pressure= 1.00000 Atm

==
==============================
SCF Energy= -4878.33760722 Predicted Change= -2.033138D-09
Zero-point correction (ZPE)= -4877.7764 0.56119
Internal Energy (U)= -4877.7373 0.60023
Enthalpy (H)= -4877.7364 0.60117
Gibbs Free Energy (G)= -4877.8509 0.48664

Frequencies -- -133.4370 9.5947 18.0182

183.15K thermal correction = 0.525275
Single point SCF = -4881.362327

TS-G: Catalyst release (Si,Re)

Using Gaussian 09: AM64L-G09RevD.01 24-Apr-2013
==
==============================
# pbepbe/6-31G(d)/auto gfprint gfinput scf=(direct,tight,maxcycle=300,xqc)
opt=(maxcycle=250,calcfc,ts,noeigentest) freq=noraman
SCRF=(PCM,SOLVENT=Dichloromethane) iop(1/8=18) Temperature=298.15
#N Geom=AllCheck Guess=TCheck SCRF=Check Test GenChk RPBEPBE/6-31G(d)/Auto
Freq

Pointgroup= C1 Stoichiometry= C35H30BrF3N2O2S
C1[X(C35H30BrF3N2O2S)] #Atoms= 74
Charge = 0 Multiplicity = 1

SCF Energy= -4878.33782991 Predicted Change= -2.311936D-09
==
==============================
Optimization completed on the basis of negligible forces.
{Found 2 times}

Item	Max Val.	Criteria	Pass?	RMS Val.	Criteria	Pass?
Force	0.00000 ||	0.00045	[YES]	0.00000 ||	0.00030	[YES]
Displ	0.00182 ||	0.00180	[NO]	0.00182 ||	0.00180	[YES]

Atomic Type	X	Y	Z
C	-6.523121	3.302003	-0.334680
H	-7.590146	3.471676	-0.162084
C	-5.977836	2.042049	-0.044416
H	-6.607907	1.236362	0.341966
C	-4.608304	1.845813	-0.272365
C	-3.805962	2.885688	-0.790328
C	-4.354445	4.138734	-1.079944
H	-3.730609	4.942618	-1.481946
C	-5.724082	4.339829	-0.844098
H	-6.169219	5.314666	-1.063858
N	-3.872041	0.677034	-0.035421
C	-2.540334	0.758732	-0.364092
S	-2.133310	2.351226	-1.043733
C	-4.429575	-0.538639	0.577399
H	-4.984056	-1.109722	-0.192037
H	-5.145222	-0.217205	1.349515
C	-3.311996	-1.385892	1.204721
C	-3.838495	-2.731472	1.773728
H	-4.082121	-3.388595	0.916199
C	-5.119776	-2.550518	2.611612
H	-5.975206	-2.197340	2.011184
H	-4.959055	-1.833174	3.437749
H	-5.413999	-3.514420	3.061164
C	-2.765464	-3.428712	2.631230
H	-1.838143	-3.639622	2.074214
H	-3.149051	-4.390405	3.012755
H	-2.493842	-2.804943	3.502591
H	-2.893177	-0.805526	2.051556
C	-2.138117	-1.540898	0.184064
C	-2.454171	-2.410124	-1.035952
H	-1.288367	-2.002661	0.703614
N	-1.667461	-0.202296	-0.205374
C	0.062360	0.185599	-0.613968
C	0.692900	0.799923	0.695270
H	-0.032116	0.767625	1.523073
C	1.344152	2.153560	0.648811
O	0.083145	0.666178	-1.753955
C	-3.150575	-1.924030	-2.160748
H	-3.454696	-0.873614	-2.212045
C	-3.440087	-2.765408	-3.245012
H	-3.978823	-2.364670	-4.110031
C	-3.033236	-4.107549	-3.228392
H	-3.255937	-4.761884	-4.077582
C	-2.324974	-4.599979	-2.122552
H	-1.985596	-5.640926	-2.103994
C	-2.034314	-3.755651	-1.041736
H	-1.462250	-4.145073	-0.191990
C	2.075380	2.600777	-0.472784
H	2.157790	1.952655	-1.348813
C	2.669475	3.870264	-0.475220
H	3.230321	4.202415	-1.355452
C	2.550319	4.713323	0.640693
H	3.018365	5.703500	0.635756
C	1.824709	4.281392	1.761224
H	1.721228	4.932058	2.636097
C	1.224769	3.014360	1.760044
H	0.654755	2.682201	2.636183
C	3.422411	-1.135758	-0.851522
H	2.683898	-1.671537	-1.453352
C	2.997353	-0.465537	0.308509
C	1.529131	-0.532256	0.712614
C	1.380462	-1.323844	2.029424
F	0.074227	-1.461088	2.409899
F	2.015264	-0.694807	3.058463
F	1.896080	-2.573101	1.920408
C	3.945093	0.242047	1.074774
H	3.636063	0.781615	1.973306
C	5.292337	0.275088	0.694236
H	6.023010	0.829149	1.289091
C	5.688929	-0.407015	-0.462793
Br	7.531795	-0.367698	-0.986531
C	4.767558	-1.112461	-1.244953
H	5.091979	-1.636730	-2.147739
O	0.709830	-1.132619	-0.318158

Statistical Thermodynamic Analysis
Temperature= 298.150 Kelvin Pressure= 1.00000 Atm
==
==============================
SCF Energy= -4878.33782991 Predicted Change= -2.311936D-09
Zero-point correction (ZPE)= -4877.7764 0.56137
Internal Energy (U)= -4877.7373 0.60045
Enthalpy (H)= -4877.7364 0.60139
Gibbs Free Energy (G)= -4877.8520 0.48577

Frequencies -- -86.7448 10.0600 15.7465

183.15K thermal correction = 0.524831
Single point SCF = -4881.362843

TS-G: Catalyst release (Si,Si)

Using Gaussian 09: AM64L-G09RevD.01 24-Apr-2013
==
==============================
# pbepbe/6-31G(d)/auto gfprint gfinput
scf=(direct,tight,maxcycle=300,xqc)
opt=(maxcycle=250,calcfc,ts,noeigentest) freq=noraman
SCRF=(PCM,SOLVENT=Dichloromethane) iop(1/8=18)
Temperature=298.15
#N Geom=AllCheck Guess=TCheck SCRF=Check Test GenChk
RPBEPBE/6-31G(d)/Auto
Freq

Pointgroup= C1 Stoichiometry= C35H30BrF3N2O2S
C1[X(C35H30BrF3N2O2S)] #Atoms= 74
Charge = 0 Multiplicity = 1

SCF Energy= -4878.33608681 Predicted Change= -9.422267D-10
==
==============================
Optimization completed on the basis of negligible forces.
{Found 2 times}
Item Max Val. Criteria Pass? RMS Val. Criteria Pass?
Force 0.00000 || 0.00045 [YES] 0.00000 || 0.00030 [YES]
Displ 0.00229 || 0.00180 [NO] 0.00229 || 0.00180 [YES]

Atomic Type	X	Y	Z
N	3.294944	-1.280151	-0.546266
C	2.535884	-2.450095	-1.013223
C	1.049266	-2.093065	-1.145861
C	0.179403	-3.294521	-1.603754
C	0.814809	-4.061285	-2.780914
C	-1.233754	-2.830319	-2.005425
C	0.579822	-1.409487	0.182601
C	0.556825	-2.342170	1.398083
N	1.403197	-0.217101	0.425859
C	0.734409	1.355798	1.126899
C	-0.143675	1.961619	-0.026061
H	-0.211814	1.244762	-0.860551
C	0.142777	3.328106	-0.599795
O	1.661587	1.878072	1.760584
C	2.670259	-0.237332	0.096190
S	3.810215	1.094672	0.429782
C	5.108481	0.173594	-0.357859
C	4.659470	-1.086643	-0.803229
C	6.440339	0.561744	-0.535083
C	7.321219	-0.332699	-1.164469
C	6.872634	-1.590943	-1.601194
C	5.537531	-1.985896	-1.425042
C	-0.603446	-3.095971	1.672862
C	-0.646631	-3.990255	2.751716
C	0.471721	-4.144474	3.584156
C	1.626597	-3.391057	3.330827
C	1.667274	-2.496475	2.250553
C	-0.219998	3.589991	-1.938418
C	0.018087	4.840786	-2.523758
C	0.645432	5.852219	-1.781171
C	1.033628	5.597001	-0.457249
C	0.785038	4.349273	0.131804
H	2.688324	-3.291698	-0.310842
H	2.952630	-2.736491	-1.990672
H	0.091074	-3.996719	-0.752577
H	0.127260	-4.851169	-3.128894
H	1.762715	-4.553533	-2.505131
H	1.010755	-3.389921	-3.637502
H	-1.850193	-3.695412	-2.304294
H	-1.185840	-2.139499	-2.867349
H	-1.768098	-2.310751	-1.192371
H	0.970469	-1.310549	-1.928062
H	-0.447443	-1.036767	0.031609
H	6.787661	1.540314	-0.189673
H	8.366413	-0.044392	-1.310795
H	7.571707	-2.280455	-2.084008
H	5.198739	-2.970760	-1.757961
H	-1.490443	-2.974262	1.041682
H	-1.561067	-4.560796	2.945485
H	0.439217	-4.838698	4.430179
H	2.501911	-3.490173	3.981281
H	2.570909	-1.899452	2.093224
H	-0.691878	2.797218	-2.531941
H	-0.277786	5.020618	-3.562720
H	0.839105	6.830164	-2.234429
H	1.537836	6.375901	0.124953
H	1.107229	4.150016	1.155480
C	-1.342777	1.628689	0.934779
C	-1.927824	2.825234	1.718330
F	-2.825375	2.396136	2.647634
F	-2.577315	3.682413	0.882324
F	-0.978244	3.529891	2.380153
C	-2.488013	0.810829	0.360577
O	-0.483742	0.869128	1.822532
C	-3.125100	1.192355	-0.834482
C	-4.189723	0.440257	-1.349219
C	-4.620834	-0.693312	-0.648257
Br	-6.064133	-1.736154	-1.350849
C	-4.015416	-1.083545	0.553197
C	-2.950618	-0.321819	1.054434
H	-2.791941	2.085247	-1.372780
H	-4.676389	0.732279	-2.283356
H	-4.370397	-1.966676	1.091055
H	-2.459377	-0.617315	1.985853

Statistical Thermodynamic Analysis
Temperature= 298.150 Kelvin Pressure= 1.00000 Atm
==
==============================
SCF Energy= -4878.33608681 Predicted Change= -9.422267D-10
Zero-point correction (ZPE)= -4877.7750 0.56100
Internal Energy (U)= -4877.7359 0.60011
Enthalpy (H)= -4877.7350 0.60106
Gibbs Free Energy (G)= -4877.8504 0.48560

Frequencies -- -125.7535 9.5595 13.3613

183.15K thermal correction = 0.524588
Single point SCF = -4881.361088

H: Minor Product

Using Gaussian 09: AM64L-G09RevD.01 24-Apr-2013
==
==============================
# pbepbe/6-31G*/auto gfprint gfinput
scf=(direct,tight,maxcycle=300,xqc)

opt=(maxcycle=250) freq=noraman iop(1/8=18)
Temperature=298.15
SCRF=(PCM,SOLVENT=Dichloromethane)
#N Geom=AllCheck Guess=TCheck SCRF=Check Test GenChk RPBEPBE/6-31G(d)/Auto
Freq

Pointgroup= C1   Stoichiometry= C16H10BrF3O2
C1[X(C16H10BrF3O2)] #Atoms= 32
Charge = 0   Multiplicity = 1

SCF Energy= -3635.82253447   Predicted Change= -1.220381D-08
==
Optimization completed.   {Found   1   times}

Item	Max Val.	Criteria	Pass?	RMS Val.	Criteria	Pass?
Force	0.00003 ||	0.00045	[YES]	0.00000 ||	0.00030	[YES]
Displ	0.00193 ||	0.00180	[NO]	0.00193 ||	0.00180	[YES]

Atomic Type	Coordinates (Angstroms) X	Y	Z
C	-1.968733	-1.663843	-1.467707
C	-1.719156	-0.220710	-1.025610
C	-2.858459	0.697176	-0.656764
O	-2.679403	-2.264197	-2.233913
C	-4.144394	0.204716	-0.362203
C	-5.180415	1.086730	-0.025812
C	-4.944458	2.468487	0.025823
C	-3.667814	2.968212	-0.273541
C	-2.634627	2.088217	-0.620636
H	-1.079705	0.266622	-1.783796
H	-4.341555	-0.870377	-0.408465
H	-6.176788	0.690338	0.194731
H	-5.755346	3.155017	0.289867
H	-3.477146	4.045879	-0.247125
H	-1.643619	2.484302	-0.871529
C	-0.751400	-0.911022	0.015532
C	-1.289723	-1.000757	1.457626
F	-1.336939	0.230344	2.029642
F	-0.492618	-1.789202	2.218715
F	-2.539527	-1.527990	1.489156
C	0.707345	-0.515471	-0.024266
O	-1.001997	-2.215025	-0.628295
C	1.684869	-1.448025	-0.411036
C	3.037550	-1.085034	-0.455627
C	3.403443	0.220273	-0.106975
Br	5.247339	0.723313	-0.162630
C	2.446112	1.166660	0.281952
C	1.097671	0.792725	0.322800
H	1.389170	-2.464028	-0.685641
H	3.795759	-1.811914	-0.757705
H	2.747043	2.183065	0.548316
H	0.351411	1.530555	0.632086

Statistical Thermodynamic Analysis
Temperature= 298.150 Kelvin   Pressure= 1.00000 Atm
==
SCF Energy=   -3635.82253447   Predicted Change= -1.220381D-08
Zero-point correction (ZPE)=   -3635.6036   0.21886
Internal Energy (U)=   -3635.5848   0.23772
Enthalpy (H)=   -3635.5838   0.23866
Gibbs Free Energy (G)=   -3635.6537   0.16876

Frequencies --   23.2384   29.7192   41.6898

183.15K thermal correction = 0.193073
Single point SCF = -3638.587140

Stepwise Mechanism Additional Structures: M06-2X

TS-A: HBTM addition to phenylacetic anhydride (Si-face)

183.15K thermal correction = 0.589661
Single point SCF = -2087.457971

Using Gaussian 09: AM64L-G09RevD.01 24-Apr-2013
==
#m062x/6-31G(d) scf=(maxcycle=300,direct,tight) density=current
SCRF=(PCM,SOLVENT=CH2Cl2)
opt=(maxcycle=250,ts,calcfc,noeigentest,gdiis)
iop(1/8=18) freq=noraman temp=298.15
#N Geom=AllCheck Guess=TCheck SCRF=Check Test GenChk RM062X/6-31G(d) Freq
----
Pointgroup= C1   Stoichiometry= C35H34N2O3S
C1[X(C35H34N2O3S)] #Atoms= 75
Charge = 0   Multiplicity = 1

SCF Energy= -2086.88267267   Predicted Change= -9.816682D-10
==
Optimization completed.   {Found   2   times}

Item	Max Val.	Criteria	Pass?	RMS Val.	Criteria	Pass?
Force	0.00000 ||	0.00045	[YES]	0.00000 ||	0.00030	[YES]
Displ	0.00083 ||	0.00180	[YES]	0.00083 ||	0.00180	[YES]

Atomic Type	Coordinates (Angstroms) X	Y	Z
C	-5.864664	-2.564475	0.368713
C	-4.803869	-1.845185	0.915674
C	-3.781695	-1.438314	0.063781
C	-3.812964	-1.752536	-1.297264
C	-4.869070	-2.472867	-1.837985
C	-5.901896	-2.873553	-0.991625
N	-2.645529	-0.705236	0.411005
C	-1.786085	-0.452523	-0.617597
S	-2.366997	-1.181235	-2.128052
C	-2.412310	-0.174218	1.751702
C	-1.473138	1.028381	1.685974
C	-1.087261	1.521241	3.093003
C	-0.366050	2.869806	3.027849
C	-2.310745	1.653164	4.006109
C	-0.250430	0.696047	0.797094
C	0.718822	-0.294810	1.422949
N	-0.695913	0.229587	-0.521701
C	0.474401	-1.670479	1.426735
C	1.374713	-2.547742	2.027147
C	2.536476	-2.065430	2.622765
C	2.792922	-0.696648	2.616315

C	1.888363	0.178925	2.021483
C	0.368788	0.371185	-2.023029
C	-0.126631	1.689244	-2.628515
C	-0.109077	2.887569	-1.719474
O	1.567351	0.560523	-1.212646
C	2.737138	0.672210	-1.854166
C	3.889410	0.485376	-0.895233
C	4.119622	-0.968287	-0.514307
O	2.852156	0.892557	-3.037862
O	0.296124	-0.673264	-2.665964
C	-1.274245	3.315018	-1.078498
C	-1.249427	4.394274	-0.197402
C	-0.052641	5.061579	0.055168
C	1.113972	4.652235	-0.589163
C	1.082345	3.577577	-1.474167
C	5.273779	-1.282883	0.211558
C	5.544673	-2.593103	0.588133
C	4.663652	-3.615256	0.235210
C	3.515895	-3.309616	-0.487911
C	3.238778	-1.993623	-0.859186
H	-6.671296	-2.889802	1.017173
H	-4.775192	-1.618747	1.975974
H	-4.887186	-2.717702	-2.895012
H	-6.737317	-3.434419	-1.396780
H	-2.001716	-0.965339	2.393597
H	-3.382003	0.127717	2.153847
H	-0.040977	3.172399	4.028076
H	-1.042304	3.641863	2.642767
H	0.514459	2.855827	2.379311
H	-0.409724	0.778818	3.535456
H	-2.018876	2.102824	4.960073
H	-2.774313	0.687712	4.228533
H	-3.068339	2.301895	3.548975
H	-2.011104	1.840420	1.175042
H	0.292158	1.626542	0.610092
H	-0.408954	-2.073965	0.937073
H	1.175942	-3.615314	2.009018
H	3.249989	-2.753779	3.064804
H	3.706783	-0.310160	3.058045
H	2.101604	1.245801	2.003801
H	-1.137681	1.494765	-3.000870
H	0.521605	1.850650	-3.496753
H	3.680768	1.067028	0.010331
H	4.785599	0.894548	-1.368544
H	-2.209073	2.791984	-1.271064
H	-2.165140	4.712872	0.293196
H	-0.030322	5.899918	0.744942
H	2.048362	5.175000	-0.407790
H	1.989335	3.269241	-1.989549
H	5.965239	-0.487594	0.481713
H	6.445802	-2.818631	1.150941
H	4.873646	-4.641459	0.521885
H	2.821007	-4.097864	-0.763518
H	2.326900	-1.775982	-1.410335

Statistical Thermodynamic Analysis
Temperature= 298.150 Kelvin Pressure= 1.00000 Atm
==
==============================
SCF Energy= -2086.88267267 Predicted Change= -9.816682D-10
Zero-point correction (ZPE)= -2086.2608 0.62186
Internal Energy (U)= -2086.2260 0.65664
Enthalpy (H)= -2086.2250 0.65758
Gibbs Free Energy (G)= -2086.3275 0.55515

Frequencies -- -142.5381 20.5772 28.3091

B: Acylated-HBTM (S•••O syn)

Using Gaussian 09: AM64L-G09RevD.01 24-Apr-2013
==
==============================
#m062X/6-31G(d) scf=(direct,tight,maxcycle=300,xqc)
SCRF=(PCM,SOLVENT=CH2Cl2) opt=(maxcycle=250,gdiis) iop(1/8=18)
freq=noraman temp=298.15
#N Geom=AllCheck Guess=TCheck SCRF=Check Test GenChk RM062X/6-31G(d) Freq
----
Pointgroup= C1 Stoichiometry= C27H27N2OS(1+)
C1[X(C27H27N2OS)] #Atoms= 58
Charge = 1 Multiplicity = 1

SCF Energy= -1627.39010271 Predicted Change= -1.335823D-09
==
==============================
Optimization completed. {Found 2 times}
Item Max Val. Criteria Pass? RMS Val. Criteria Pass?
Force 0.00000 || 0.00045 [YES] 0.00000 || 0.00030 [YES]
Displ 0.00086 || 0.00180 [YES] 0.00086 || 0.00180 [YES]

Atomic Type	Coordinates (Angstroms) X	Y	Z
C	5.913120	-0.936267	0.480225
C	5.475822	0.021115	1.406107
C	4.141094	0.391899	1.485320
C	3.253129	-0.225872	0.606045
C	3.683605	-1.170572	-0.322012
C	5.023991	-1.544130	-0.395398
N	1.871361	-0.005408	0.518853
C	1.269245	-0.710365	-0.439612
S	2.352919	-1.753139	-1.302931
C	1.170036	0.889591	1.454009
C	-0.312831	0.558700	1.452325
C	-1.140383	1.513820	2.331386
C	-2.574196	0.995693	2.483173
C	-0.506822	1.679536	3.716240
C	-0.812598	0.501933	-0.002768
C	-0.767723	1.813537	-0.764731
N	-0.054132	-0.562645	-0.709247
C	-0.652698	-1.348756	-1.724864
C	-2.127435	-1.148529	-1.985467
C	-2.996743	-1.613609	-0.829456
O	0.017758	-2.160972	-2.320926
C	-1.913132	2.616042	-0.781160
C	-1.909674	3.842646	-1.438945
C	-0.762085	4.277449	-2.097333
C	0.378214	3.477819	-2.098719
C	0.375651	2.251968	-1.438522
C	-2.611873	-2.691560	-0.028469
C	-3.408553	-3.090460	1.042466
C	-4.597571	-2.419415	1.319011
C	-4.990714	-1.349560	0.517511
C	-4.191854	-0.946093	-0.549324
H	6.962941	-1.205465	0.444424
H	6.192501	0.483855	2.075426
H	3.809658	1.139701	2.196533
H	5.359702	-2.282303	-1.115505
H	1.364229	1.926939	1.155761
H	1.609176	0.712137	2.436587
H	-3.110956	0.914303	1.532931
H	-3.149965	1.667026	3.126246

H	-2.573548	0.001524	2.946523
H	-1.163441	2.497615	1.844052
H	-1.158639	2.283536	4.353059
H	0.463700	2.182278	3.672689
H	-0.371353	0.705611	4.201572
H	-0.430650	-0.456770	1.858736
H	-1.841935	0.152558	0.037232
H	-2.337563	-1.724862	-2.890090
H	-2.323250	-0.094122	-2.210968
H	-2.816884	2.277871	-0.279085
H	-2.807042	4.452642	-1.443199
H	-0.758692	5.231002	-2.615314
H	1.272663	3.803142	-2.619958
H	1.273157	1.639588	-1.472520
H	-1.689641	-3.227746	-0.242193
H	-3.099340	-3.928605	1.658783
H	-5.215625	-2.729184	2.155455
H	-5.916544	-0.822717	0.725771
H	-4.494165	-0.104527	-1.168112

Statistical Thermodynamic Analysis
Temperature= 298.150 Kelvin Pressure= 1.00000 Atm
==
==============================
SCF Energy= -1627.39010271 Predicted Change= -1.335823D-09
Zero-point correction (ZPE)= -1626.9005 0.48959
Internal Energy (U)= -1626.8743 0.51572
Enthalpy (H)= -1626.8734 0.51666
Gibbs Free Energy (G)= -1626.9576 0.43248

Frequencies -- 21.6527 24.9674 36.1116

183.15K thermal correction = 0.461176
Single point SCF = -1627.806482

B: Acylated-HBTM (S•••O anti)

Using Gaussian 09: AM64L-G09RevD.01 24-Apr-2013
==
==============================
#m062X/6-31G(d) scf=(direct,tight,maxcycle=300,xqc)
SCRF=(PCM,SOLVENT=CH2Cl2) opt=(maxcycle=250,gdiis) iop(1/8=18)
freq=noraman temp=298.15
#N Geom=AllCheck Guess=TCheck SCRF=Check Test GenChk RM062X/6-31G(d) Freq
----
Pointgroup= C1 Stoichiometry= C27H27N2OS(1+)
C1[X(C27H27N2OS)] #Atoms= 58
Charge = 1 Multiplicity = 1

SCF Energy= -1627.37658948 Predicted Change= -1.405462D-08
==
==============================
Optimization completed. {Found 1 times}

Item	Max Val.	Criteria	Pass?	RMS Val.	Criteria	Pass?
Force	0.00000 ‖	0.00045	[YES]	0.00000 ‖	0.00030	[YES]
Displ	0.01090 ‖	0.00180	[NO]	0.01090 ‖	0.00180	[NO]

Atomic Type	Coordinates (Angstroms) X	Y	Z
C	-4.808917	2.369981	0.932375
C	-3.828052	3.169708	1.532477
C	-2.477269	2.969583	1.280741
C	-2.133321	1.942595	0.403617
C	-3.107993	1.143318	-0.185450
C	-4.462181	1.341079	0.066774
N	-0.838301	1.570198	0.011486
C	-0.788547	0.510032	-0.804421
S	-2.376754	-0.073127	-1.203363
C	0.329176	2.369615	0.408877
C	1.459741	2.149826	-0.580148
C	2.754991	2.846198	-0.119172
C	3.844414	2.748020	-1.189630
C	2.498813	4.318172	0.219622
C	1.665297	0.652027	-0.833076
C	2.263617	-0.148643	0.308278
N	0.381102	0.007825	-1.256064
C	0.582948	-1.187782	-2.039813
C	-0.499415	-2.234113	-2.195769
C	-0.798606	-2.932387	-0.879681
O	1.692651	-1.377467	-2.468819
C	3.560690	-0.649917	0.178271
C	4.133929	-1.406682	1.197128
C	3.410972	-1.676739	2.356286
C	2.109555	-1.196697	2.487172
C	1.536977	-0.442097	1.467151
C	0.222808	-3.173637	0.042314
C	-0.048169	-3.846662	1.231681
C	-1.337034	-4.298096	1.502137
C	-2.356490	-4.074333	0.577938
C	-2.089034	-3.391066	-0.604343
H	-5.855661	2.552056	1.148708
H	-4.125499	3.961090	2.211360
H	-1.724223	3.583398	1.761426
H	-5.215344	0.713162	-0.395529
H	0.614040	2.103011	1.432723
H	0.004797	3.410697	0.393132
H	4.151007	1.718341	-1.389816
H	4.734378	3.295882	-0.868176
H	3.498436	3.190448	-2.131069
H	3.104545	2.336675	0.788900
H	2.043673	4.839835	-0.630854
H	3.444884	4.815609	0.449441
H	1.847119	4.442708	1.089471
H	1.153947	2.595624	-1.537526
H	2.312051	0.551289	-1.702781
H	-0.071485	-2.943597	-2.909068
H	-1.404131	-1.840382	-2.664701
H	4.116741	-0.468282	-0.737482
H	5.140403	-1.794560	1.078467
H	3.854688	-2.269980	3.149460
H	1.530876	-1.422695	3.377101
H	0.502001	-0.125256	1.571789
H	1.239338	-2.843258	-0.163995
H	0.754051	-4.016297	1.943365
H	-1.547360	-4.824655	2.427622
H	-3.362728	-4.428402	0.778810
H	-2.886693	-3.216668	-1.322384

Statistical Thermodynamic Analysis
Temperature= 298.150 Kelvin Pressure= 1.00000 Atm
==
==============================
SCF Energy= -1627.37658948 Predicted Change= -1.405462D-08
Zero-point correction (ZPE)= -1626.8875 0.48901
Internal Energy (U)= -1626.8613 0.51524
Enthalpy (H)= -1626.8604 0.51618

Gibbs Free Energy (G)= -1626.9458
0.43070

Frequencies -- 6.6929 23.8008 31.9658

183.15K thermal correction = 0.459887
Single point SCF = -1627.794143

TS-C: Acylated-HBTM deprotonation *via* phenylacetate (making E)

Using Gaussian 09: AM64L-G09RevD.01 24-Apr-2013
==
#m062x/6-31G(d) scf=(maxcycle=300,direct,tight) density=current
opt=(maxcycle=250,ts,calcfc,noeigentest,gdiis) iop(1/8=18) freq=noraman
temp=298.15 SCRF=(PCM,SOLVENT=dichloromethane)
#N Geom=AllCheck Guess=TCheck SCRF=Check Test GenChk RM062X/6-31G(d) Freq
----
Pointgroup= C1 Stoichiometry= C35H34N2O3S
C1[X(C35H34N2O3S)] #Atoms= 75
Charge = 0 Multiplicity = 1

SCF Energy= -2086.86747302 Predicted Change= -3.900977D-10
==
Optimization completed. {Found 2 times}

Item	Max Val.	Criteria	Pass?	RMS Val.	Criteria	Pass?
Force	0.00000 ||	0.00045	[YES]	0.00000 ||	0.00030	[YES]
Displ	0.00104 ||	0.00180	[YES]	0.00104 ||	0.00180	[YES]

Atomic Coordinates (Angstroms)

Type	X	Y	Z
C	-5.047862	-2.604998	0.802908
C	-3.822575	-2.060339	1.169049
C	-2.964615	-1.662090	0.145901
C	-3.310715	-1.808057	-1.194269
C	-4.541593	-2.348419	-1.556142
C	-5.405878	-2.747102	-0.542743
N	-1.702639	-1.076969	0.296718
C	-1.080908	-0.790788	-0.855082
S	-2.049823	-1.194209	-2.254214
C	-1.185784	-0.721006	1.620515
C	-0.061979	0.291594	1.488478
C	0.613069	0.534050	2.852227
C	1.629064	1.677110	2.794757
C	-0.426222	0.848816	3.934334
C	0.911457	-0.177856	0.385587
C	1.646451	-1.471745	0.704574
N	0.163649	-0.297907	-0.889136
C	1.202397	-2.718647	0.256698
C	1.902190	-3.881020	0.577113
C	3.058901	-3.813065	1.347500
C	3.512908	-2.573358	1.793656
C	2.813492	-1.415276	1.473359
C	0.764563	0.000730	-2.187752
C	2.050846	0.620267	-2.278497
C	3.283225	0.281360	-1.484620
O	0.008042	-0.163415	-3.147585
C	3.875842	1.208928	-0.619008
C	5.069396	0.915256	0.039656
C	5.700168	-0.310383	-0.163178
C	5.126755	-1.241338	-1.027694
C	3.933504	-0.944644	-1.680388
H	-5.735644	-2.926660	1.577479
H	-3.544997	-1.959230	2.212381
H	-4.815154	-2.454783	-2.600606
H	-6.369166	-3.174189	-0.800016
H	-0.851757	-1.638309	2.122678
H	-2.025778	-0.292456	2.172749
H	2.132672	1.770152	3.762459
H	1.122364	2.619729	2.567522
H	2.392887	1.542265	2.022794
H	1.129420	-0.390480	3.142806
H	0.078107	1.127335	4.864530
H	-1.075460	-0.003118	4.158717
H	-1.056455	1.691646	3.626390
H	-0.486267	1.243289	1.137424
H	1.621392	0.632219	0.225316
H	0.318020	-2.802691	-0.368913
H	1.542699	-4.838584	0.213289
H	3.608293	-4.717223	1.590486
H	4.426457	-2.503612	2.376491
H	3.200532	-0.451943	1.793016
H	1.532715	1.997812	-2.125572
H	2.279788	0.657777	-3.346957
H	3.373962	2.158191	-0.448928
H	5.507737	1.648989	0.710776
H	6.630513	-0.539130	0.348542
H	5.606255	-2.202652	-1.189529
H	3.488465	-1.673833	-2.353551
O	1.196956	2.944202	0.081947
C	0.610400	3.334407	-0.925090
C	-0.619019	4.230829	-0.786619
C	-1.709571	3.405831	-0.143120
O	0.905745	2.992703	-2.140563
C	-2.370215	2.422227	-0.887757
C	-3.336633	1.614769	-0.293464
C	-3.643090	1.764677	1.059560
C	-2.982247	2.735676	1.809550
C	-2.021482	3.550100	1.209926
H	-0.925973	4.594939	-1.769331
H	-0.361012	5.081464	-0.150171
H	-2.120707	2.291882	-1.939498
H	-3.862151	0.871299	-0.887763
H	-4.397991	1.132709	1.519742
H	-3.218528	2.866004	2.861840
H	-1.506541	4.305741	1.797839

Statistical Thermodynamic Analysis
Temperature= 298.150 Kelvin Pressure= 1.00000 Atm
==
SCF Energy= -2086.86747302 Predicted Change= -3.900977D-10
Zero-point correction (ZPE)= -2086.2496 0.61782
Internal Energy (U)= -2086.2146 0.65286
Enthalpy (H)= -2086.2136
0.65380
Gibbs Free Energy (G)= -2086.3163
0.55109

Frequencies -- -1054.6525 18.2585 25.4597

183.15K thermal correction = 0.585679
Single point SCF = -2087.448562

TS-C: Acylated-HBTM deprotonation *via N,N*-diisopropylethylamine (making E)

Using Gaussian 09: AM64L-G09RevD.01 24-Apr-2013
==
==============================
#m062x/6-31G(d) scf=(maxcycle=300,direct,tight) density=current
opt=(maxcycle=250,ts,calcfc,noeigentest,gdiis) iop(1/8=18) freq=noraman
temp=298.15 SCRF=(PCM,SOLVENT=dichloromethane)
#N Geom=AllCheck Guess=TCheck SCRF=Check Test GenChk RM062X/6-31G(d) Freq
----
Pointgroup= C1 Stoichiometry= C35H46N3OS(1+)
C1[X(C35H46N3OS)] #Atoms= 86
Charge = 1 Multiplicity = 1

SCF Energy= -1998.21989417 Predicted Change= -3.676111D-08
==
==============================
Optimization completed. {Found 1 times}

Item	Max Val.	Criteria	Pass?	RMS Val.	Criteria	Pass?
Force	0.00006 ||	0.00045	[YES]	0.00000 ||	0.00030	[YES]
Displ	0.00355 ||	0.00180	[NO]	0.00355 ||	0.00180	[YES]

Atomic Coordinates (Angstroms)

Type	X	Y	Z
C	6.392815	-2.037127	-0.339536
C	5.499457	-1.037578	0.022636
C	4.147931	-1.261585	-0.234413
C	3.707439	-2.432182	-0.843663
C	4.606243	-3.431880	-1.206814
C	5.953891	-3.222203	-0.943291
N	3.082779	-0.395532	0.053444
C	1.882132	-0.846155	-0.331268
S	1.970118	-2.414951	-1.086105
C	3.292587	0.871579	0.760585
C	2.004805	1.279607	1.451959
C	2.136694	2.646454	2.152402
C	0.926868	2.932960	3.045321
C	3.411733	2.718389	3.000119
C	0.842098	1.228196	0.440713
C	0.892789	2.286699	-0.647260
N	0.752752	-0.131020	-0.151787
C	1.632141	2.099441	-1.817542
C	1.685793	3.099629	-2.785244
C	0.997499	4.294958	-2.596047
C	0.248170	4.484526	-1.437331
C	0.193078	3.483737	-0.471793
C	-0.488802	-0.607273	-0.753922
C	-1.679302	0.177544	-0.545534
C	-2.775151	0.148526	-1.561145
O	-0.425204	-1.689361	-1.324753
C	-3.698375	1.207005	-1.518798
C	-4.803744	1.242867	-2.359041
C	-5.012568	0.220014	-3.283809
C	-4.094162	-0.822504	-3.356166
C	-2.988571	-0.865592	-2.505520
H	7.450960	-1.890990	-0.152463
H	5.849220	-0.117365	0.476378
H	4.261609	-4.344561	-1.680976
H	6.675299	-3.985426	-1.214030
H	3.636267	1.628809	0.045446
H	4.079947	0.693186	1.493279
H	1.024500	3.921604	3.502174
H	0.868528	2.195435	3.854926
H	-0.024716	2.917106	2.506549
H	2.198167	3.420346	1.376880
H	3.421114	3.648974	3.574067
H	4.320642	2.702628	2.392299
H	3.455083	1.885663	3.712589
H	1.790373	0.521943	2.220521
H	-0.071397	1.353905	1.022967
H	2.160381	1.166022	-1.997245
H	2.261120	2.938032	-3.691109
H	1.038046	5.071936	-3.352632
H	-0.301575	5.408023	-1.286899
H	-0.405928	3.634864	0.423856
H	-2.308733	-0.401861	0.579892
H	-1.493004	1.203960	-0.245495
H	-3.541697	2.014618	-0.805871
H	-5.500883	2.073183	-2.295022
H	-5.875597	0.240861	-3.941625
H	-4.235010	-1.619222	-4.080905
H	-2.287269	-1.687156	-2.581366
N	-3.025600	-0.876721	1.633426
C	-2.791996	-2.359922	1.707214
C	-2.953870	-3.080367	0.366834
C	-3.597081	-3.035380	2.815770
C	-2.459641	-0.170969	2.839978
C	-2.606033	1.349960	2.725153
C	-0.993667	-0.545084	3.079386
C	-4.447652	-0.449199	1.465643
C	-5.302911	-1.169415	0.432525
H	-2.518482	-2.506115	-0.453806
H	-3.989216	-3.318781	0.125631
H	-2.406836	-4.025936	0.433343
H	-4.673495	-2.959473	2.633650
H	-3.383223	-2.617206	3.803621
H	-3.339545	-4.097832	2.842224
H	-1.732087	-2.450659	1.949771
H	-2.081322	1.814807	3.563804
H	-3.644263	1.681122	2.770350
H	-2.166787	1.731854	1.796969
H	-0.876532	-1.530411	3.534826
H	-0.545203	0.180783	3.762546
H	-0.420996	-0.535432	2.144663
H	-3.047635	-0.490220	3.710305
H	-5.596243	-2.171244	0.750733
H	-4.814851	-1.227692	-0.543213
H	-6.218733	-0.583276	0.311146
H	-4.406016	0.603233	1.180516
H	-4.937586	-0.501084	2.448263

Statistical Thermodynamic Analysis
Temperature= 298.150 Kelvin Pressure= 1.00000 Atm
==
==============================
SCF Energy= -1998.21989417 Predicted Change= -3.676111D-08
Zero-point correction (ZPE)= -1997.4659 0.75398
Internal Energy (U)= -1997.4275 0.79238
Enthalpy (H)= -1997.4265 0.79332
Gibbs Free Energy (G)= -1997.5352 0.68461

Frequencies -- -1282.2824 17.2359 25.1330

183.15K thermal correction = 0.721022
Single point SCF = -1998.754831

TS-C: Acylated-HBTM deprotonation *via N,N*-diisopropylethylamine (making Z)

Using Gaussian 09: AM64L-G09RevD.01 24-Apr-2013

==
==============================
#m062x/6-31G(d) scf=(maxcycle=300,direct,tight) density=current
opt=(maxcycle=250,ts,calcfc,noeigentest,gdiis) iop(1/8=18) freq=noraman
temp=298.15 SCRF=(PCM,SOLVENT=dichloromethane)
#N Geom=AllCheck Guess=TCheck SCRF=Check Test GenChk RM062X/6-31G(d) Freq
----
Pointgroup= C1 Stoichiometry= C35H46N3OS(1+)
C1[X(C35H46N3OS)] #Atoms= 86
Charge = 1 Multiplicity = 1

SCF Energy= -1998.21300152 Predicted Change= -1.529631D-08
==
==============================
Optimization completed. {Found 1 times}

Item	Max Val.	Criteria	Pass?	RMS Val.	Criteria	Pass?
Force	0.00001 ||	0.00045	[YES]	0.00000 ||	0.00030	[YES]
Displ	0.00602 ||	0.00180	[NO]	0.00602 ||	0.00180	[NO]

Atomic Coordinates (Angstroms)

Type	X	Y	Z
C	6.254912	-1.566960	0.187991
C	5.181433	-0.794966	0.613216
C	3.942429	-1.042370	0.025223
C	3.781061	-2.015104	-0.956652
C	4.858963	-2.790728	-1.376402
C	6.097367	-2.554791	-0.792639
N	2.735592	-0.389323	0.311662
C	1.698008	-0.795394	-0.430375
S	2.116439	-2.095515	-1.512221
C	2.635709	0.585756	1.404639
C	1.179524	0.750833	1.803863
C	1.002413	1.833977	2.883633
C	-0.441550	1.879744	3.389412
C	1.943582	1.587205	4.068342
C	0.353572	0.988555	0.519767
C	0.718583	2.266296	-0.220632
N	0.488397	-0.203917	-0.347721
C	1.576168	2.274392	-1.322971
C	1.888291	3.469564	-1.967804
C	1.345464	4.670330	-1.520245
C	0.484859	4.669148	-0.424982
C	0.173439	3.476323	0.217580
C	-0.603790	-0.720600	-1.158316
C	-1.937101	-0.170891	-1.073410
C	-2.232464	1.311402	-1.042917
O	-0.330800	-1.732796	-1.803638
C	-2.938184	1.935926	-0.009143
C	-3.265608	3.291400	-0.071106
C	-2.896026	4.050866	-1.176566
C	-2.195618	3.446033	-2.219465
C	-1.872071	2.095326	-2.149676
H	7.232931	-1.397641	0.624900
H	5.311067	-0.024581	1.364866
H	4.733022	-3.552271	-2.138390
H	6.954241	-3.142609	-1.103031
H	3.075076	1.534452	1.073811
H	3.225782	0.185041	2.230089
H	-0.550611	2.663201	4.144551
H	-0.708589	0.923909	3.856390
H	-1.173614	2.082942	2.599990
H	1.260889	2.804908	2.443715
H	1.722256	2.294966	4.872031
H	2.996178	1.718202	3.801646
H	1.811031	0.574301	4.467165
H	0.833708	-0.202643	2.230713
H	-0.700049	1.025747	0.794345
H	1.992903	1.349501	-1.711483
H	2.550681	3.456111	-2.827475
H	1.583595	5.599724	-2.027764
H	0.037801	5.596099	-0.079196
H	-0.531036	3.485586	1.044457
H	-2.440820	-0.584940	-1.952048
H	-2.609952	-1.164771	-0.169673
H	-3.248026	1.357382	0.856206
H	-3.815228	3.748587	0.746579
H	-3.150252	5.105311	-1.225992
H	-1.896652	4.028405	-3.085929
H	-1.327876	1.628270	-2.967482
N	-3.175138	-2.175329	0.412961
C	-2.718472	-3.406679	-0.337727
C	-3.199471	-3.403390	-1.785556
C	-3.123085	-4.710745	0.351391
C	-2.675722	-2.154511	1.832598
C	-2.881894	-0.770161	2.441289
C	-1.195273	-2.519224	1.930418
C	-4.665577	-2.023108	0.365383
C	-5.131046	-0.944451	-0.602854
H	-2.872808	-2.513569	-2.324695
H	-4.287030	-3.497319	-1.862059
H	-2.759136	-4.268922	-2.288255
H	-4.207477	-4.856489	0.347072
H	-2.773680	-4.776535	1.384169
H	-2.680083	-5.543684	-0.201440
H	-1.629506	-3.332606	-0.362864
H	-2.690813	-0.816481	3.516711
H	-3.891396	-0.376283	2.298576
H	-2.170792	-0.062032	2.006019
H	-0.987489	-3.567653	1.711633
H	-0.855689	-2.318249	2.950741
H	-0.598121	-1.900140	1.250930
H	-3.268269	-2.885927	2.396481
H	-4.813351	-1.154023	-1.627792
H	-4.746350	0.039375	-0.321737
H	-6.223357	-0.901225	-0.588957
H	-5.023988	-1.798631	1.371464
H	-5.104655	-2.986218	0.095491

Statistical Thermodynamic Analysis
Temperature= 298.150 Kelvin Pressure= 1.00000 Atm
==
==============================
SCF Energy= -1998.21300152 Predicted Change= -1.529631D-08
Zero-point correction (ZPE)= -1997.4588 0.75414
Internal Energy (U)= -1997.4206 0.79232
Enthalpy (H)= -1997.4197 0.79327
Gibbs Free Energy (G)= -1997.5280 0.68490

Frequencies -- -1311.7412 6.7543 24.0906

183.15K thermal correction = 0.721188
Single point SCF = -1998.748098

D: (Z)-enolate (S•••O syn)

Using Gaussian 09: AM64L-G09RevD.01 24-Apr-2013
==
==============================

#m062X/6-31G(d) scf=(direct,tight,maxcycle=300,xqc)
SCRF=(PCM,SOLVENT=CH2Cl2) opt=(maxcycle=250,gdiis)
iop(1/8=18)
freq=noraman temp=298.15
#N Geom=AllCheck Guess=TCheck SCRF=Check Test GenChk
RM062X/6-31G(d) Freq

Pointgroup= C1 Stoichiometry= C27H26N2OS
C1[X(C27H26N2OS)] #Atoms= 57
Charge = 0 Multiplicity = 1

SCF Energy= -1626.90998011 Predicted Change= -1.315205D-08
===
Optimization completed. {Found 2 times}

Item	Max Val.	Criteria	Pass?	RMS Val.	Criteria	Pass?
Force	0.00002 ‖	0.00045	[YES]	0.00000 ‖	0.00030	[YES]
Displ	0.00120 ‖	0.00180	[YES]	0.00120 ‖	0.00180	[YES]

Atomic Type	Coordinates (Angstroms) X	Y	Z
C	-5.522808	-1.801019	0.540274
C	-4.599475	-0.820490	0.191830
C	-3.265401	-1.202003	0.078020
C	-2.859504	-2.512951	0.314014
C	-3.787485	-3.490940	0.655239
C	-5.124748	-3.121840	0.767170
N	-2.184927	-0.376189	-0.255962
C	-0.982709	-0.988049	-0.237007
S	-1.117112	-2.703362	0.109289
C	-2.355454	1.034359	-0.603893
C	-1.154718	1.486935	-1.417615
C	-1.238121	2.975887	-1.830214
C	-1.984188	3.104155	-3.161725
C	-1.887477	3.891338	-0.785145
C	0.144354	1.115742	-0.675889
C	0.386488	1.915611	0.588682
N	0.158256	-0.339527	-0.420140
C	1.187915	3.059090	0.530930
C	1.348697	3.867471	1.652411
C	0.717617	3.533698	2.848932
C	-0.059756	2.379892	2.921657
C	-0.221691	1.573060	1.798291
C	1.445312	-1.102402	-0.371449
C	2.589143	-0.345633	-0.282610
C	3.933944	-0.894585	-0.255563
O	1.285644	-2.344708	-0.407878
C	5.020837	-0.003283	-0.127198
C	6.335451	-0.448698	-0.097925
C	6.622112	-1.810602	-0.197950
C	5.563237	-2.708189	-0.325912
C	4.243252	-2.268204	-0.354202
H	-6.568548	-1.530136	0.638908
H	-4.912801	0.204560	0.027768
H	-3.473327	-4.514145	0.834533
H	-5.864258	-3.868055	1.037484
H	-2.471992	1.614844	0.317768
H	-3.273613	1.121064	-1.191319
H	-3.006299	2.716413	-3.076185
H	-1.477209	2.551181	-3.958101
H	-2.052486	4.153396	-3.464294
H	-0.208458	3.321452	-1.991553
H	-2.960268	3.683186	-0.695978
H	-1.785631	4.933414	-1.102401
H	-1.434915	3.795468	0.205664
H	-1.142875	0.886243	-2.336696
H	0.967060	1.291712	-1.373263
H	1.688880	3.318083	-0.399459
H	1.971787	4.754339	1.593635
H	0.842457	4.162300	3.724983
H	-0.539131	2.103170	3.855383
H	-0.817980	0.666553	1.874853
H	2.521530	0.727905	-0.176117
H	4.814873	1.062381	-0.048449
H	7.142338	0.272456	0.003665
H	7.648572	-2.163597	-0.175846
H	5.766133	-3.773473	-0.405112
H	3.432549	-2.979357	-0.451701

Statistical Thermodynamic Analysis
Temperature= 298.150 Kelvin Pressure= 1.00000 Atm
===
SCF Energy= -1626.90998011 Predicted Change= -1.315205D-08
Zero-point correction (ZPE)= -1626.4348 0.47518
Internal Energy (U)= -1626.4087 0.50120
Enthalpy (H)= -1626.4078 0.50215
Gibbs Free Energy (G)= -1626.4916 0.41834

Frequencies -- 23.2994 25.8743 36.0946

183.15K thermal correction = 0.446892
Single point SCF = -1627.335940

D: (Z)-enolate (S•••O anti)

Using Gaussian 09: AM64L-G09RevD.01 24-Apr-2013
===
#m062X/6-31G(d) scf=(direct,tight,maxcycle=300,xqc)
SCRF=(PCM,SOLVENT=CH2Cl2) opt=(maxcycle=250,gdiis)
iop(1/8=18)
freq=noraman temp=298.15
#N Geom=AllCheck Guess=TCheck SCRF=Check Test GenChk
RM062X/6-31G(d) Freq

Pointgroup= C1 Stoichiometry= C27H26N2OS
C1[X(C27H26N2OS)] #Atoms= 57
Charge = 0 Multiplicity = 1

SCF Energy= -1626.90587454 Predicted Change= -1.855391D-09
===
Optimization completed. {Found 2 times}

Item	Max Val.	Criteria	Pass?	RMS Val.	Criteria	Pass?
Force	0.00001 ‖	0.00045	[YES]	0.00000 ‖	0.00030	[YES]
Displ	0.00097 ‖	0.00180	[YES]	0.00097 ‖	0.00180	[YES]

Atomic Type	Coordinates (Angstroms) X	Y	Z
C	3.500153	4.280962	0.756344
C	3.358631	2.921705	0.490540
C	2.079343	2.441318	0.229066
C	0.973233	3.292407	0.245341
C	1.116266	4.647417	0.514672
C	2.395606	5.135975	0.766890

N	1.721874	1.119994	-0.068175
C	0.397987	0.930761	-0.261030
S	-0.523315	2.411747	-0.066627
C	2.685744	0.030748	-0.242076
C	2.113281	-0.985232	-1.224441
C	3.077011	-2.165210	-1.493459
C	4.040258	-1.805555	-2.628418
C	3.875164	-2.627645	-0.268257
C	0.705498	-1.419152	-0.777210
C	0.666937	-2.314057	0.444613
N	-0.133446	-0.227454	-0.567314
C	0.592709	-3.698387	0.279796
C	0.643945	-4.545937	1.383735
C	0.758910	-4.014774	2.666507
C	0.807719	-2.633095	2.840974
C	0.757025	-1.787524	1.736070
C	-1.607610	-0.388387	-0.940282
C	-2.453956	0.047567	0.052441
C	-3.901726	0.101249	-0.061281
O	-1.774916	-0.902006	-2.055536
C	-4.606574	-0.256869	-1.229411
C	-5.994117	-0.172767	-1.280554
C	-6.729631	0.264615	-0.179544
C	-6.048519	0.619827	0.984883
C	-4.662642	0.540442	1.041156
H	4.488719	4.677775	0.961102
H	4.219627	2.262828	0.496410
H	0.253018	5.304220	0.526472
H	2.532147	6.191121	0.977125
H	2.897190	-0.415692	0.735271
H	3.608811	0.464428	-0.634708
H	4.652633	-0.935788	-2.361936
H	3.500602	-1.570674	-3.550729
H	4.720968	-2.637572	-2.832708
H	2.460278	-3.007430	-1.834184
H	4.610681	-1.870275	0.028283
H	4.431119	-3.535883	-0.519420
H	3.241707	-2.849084	0.595048
H	1.960996	-0.456661	-2.174956
H	0.231548	-1.935956	-1.616710
H	0.494502	-4.115379	-0.719430
H	0.586353	-5.620540	1.241340
H	0.796098	-4.673589	3.528274
H	0.880145	-2.211578	3.838671
H	0.776913	-0.710219	1.886914
H	-2.022089	0.327926	1.007440
H	-4.044956	-0.600557	-2.090055
H	-6.509062	-0.453869	-2.195830
H	-7.812565	0.326359	-0.226794
H	-6.601391	0.961640	1.856046
H	-4.144495	0.822347	1.955411

Statistical Thermodynamic Analysis
Temperature= 298.150 Kelvin Pressure= 1.00000 Atm
==
==============================
SCF Energy= -1626.90587454 Predicted Change= -
1.855391D-09
Zero-point correction (ZPE)= -1626.4308 0.47505
Internal Energy (U)= -1626.4045 0.50130
Enthalpy (H)= -1626.4036
0.50224
Gibbs Free Energy (G)= -1626.4883
0.41756

Frequencies -- 17.4937 25.1069 38.5078

183.15K thermal correction = 0.446436
Single point SCF = -1627.332841

D: (E)-enolate (S•••O syn)

Using Gaussian 09: AM64L-G09RevD.01 24-Apr-2013
==
==============================
#m062X/6-31G(d) scf=(direct,tight,maxcycle=300,xqc)
SCRF=(PCM,SOLVENT=CH2Cl2) opt=(maxcycle=250,gdiis)
iop(1/8=18)
freq=noraman temp=298.15
#N Geom=AllCheck Guess=TCheck SCRF=Check Test GenChk
RM062X/6-31G(d) Freq

Pointgroup= C1 Stoichiometry= C27H26N2OS
C1[X(C27H26N2OS)] #Atoms= 57
Charge = 0 Multiplicity = 1

SCF Energy= -1626.90045745 Predicted Change= -6.588697D-
09
==
==============================
Optimization completed. {Found 2 times}
Item Max Val. Criteria Pass? RMS Val. Criteria
Pass?
Force 0.00001 || 0.00045 [YES] 0.00000 || 0.00030 [
YES]
Displ 0.00153 || 0.00180 [YES] 0.00153 || 0.00180 [
YES]

Atomic Type	Coordinates (Angstroms) X	Y	Z
C	5.368493	-0.233450	1.435598
C	4.043707	0.183535	1.522194
C	3.151136	-0.292924	0.566635
C	3.569264	-1.149277	-0.452776
C	4.894673	-1.558741	-0.539213
C	5.790575	-1.096877	0.421315
N	1.782223	-0.009178	0.483523
C	1.157741	-0.589987	-0.561066
S	2.255052	-1.509544	-1.569289
C	1.081267	0.857592	1.433349
C	-0.404052	0.519174	1.430987
C	-1.230533	1.430827	2.368069
C	-1.251838	0.842890	3.781870
C	-0.762742	2.890305	2.415577
C	-0.942052	0.467259	-0.010183
C	-1.030245	1.804842	-0.716856
N	-0.136792	-0.498051	-0.782782
C	-2.254581	2.477502	-0.720348
C	-2.363152	3.750759	-1.272296
C	-1.247764	4.359555	-1.843333
C	-0.030765	3.682189	-1.873489
C	0.077230	2.409504	-1.317483
C	-0.710704	-1.106733	-2.048615
C	-1.956499	-1.678135	-1.931408
C	-2.695719	-2.064279	-0.737205
O	0.031731	-0.974902	-3.041196
C	-2.081727	-2.435449	0.477465
C	-2.832200	-2.770582	1.600797
C	-4.224858	-2.790330	1.544643
C	-4.852858	-2.474045	0.339451
C	-4.104369	-2.115697	-0.775326
H	6.082929	0.124582	2.168998
H	3.724681	0.862784	2.304790
H	5.220140	-2.220226	-1.335128
H	6.828629	-1.407834	0.375488
H	1.264323	1.899342	1.147414
H	1.513543	0.686194	2.422710
H	-0.238725	0.779237	4.196752

H	-1.684762	-0.162562	3.784033
H	-1.845008	1.472430	4.451996
H	-2.261463	1.417213	1.989731
H	0.205239	2.975121	2.923398
H	-1.481240	3.484697	2.987824
H	-0.669471	3.338860	1.422299
H	-0.503241	-0.510277	1.801617
H	-1.946346	0.048034	0.045142
H	-3.128497	1.999179	-0.284426
H	-3.320189	4.263057	-1.263637
H	-1.330267	5.351251	-2.276830
H	0.837150	4.141403	-2.336189
H	1.030141	1.887922	-1.370487
H	-2.420922	-1.904812	-2.886812
H	-0.997186	-2.482705	0.527789
H	-2.320415	-3.043207	2.520430
H	-4.809408	-3.061562	2.418023
H	-5.937095	-2.497356	0.270081
H	-4.609818	-1.854796	-1.702135

Statistical Thermodynamic Analysis
Temperature= 298.150 Kelvin Pressure= 1.00000 Atm
==
==============================
SCF Energy= -1626.90045745 Predicted Change= -6.588697D-09
Zero-point correction (ZPE)= -1626.4256 0.47478
Internal Energy (U)= -1626.3995 0.50095
Enthalpy (H)= -1626.3985 0.50189
Gibbs Free Energy (G)= -1626.4827 0.41773

Frequencies -- 21.5161 26.6595 32.0764

183.15K thermal correction = 0.446411
Single point SCF = -1627.327826

D: (E)-enolate (S•••O anti)

Using Gaussian 09: AM64L-G09RevD.01 24-Apr-2013
==
==============================
#m062X/6-31G(d) scf=(direct,tight,maxcycle=300,xqc)
SCRF=(PCM,SOLVENT=CH2Cl2) opt=(maxcycle=250,gdiis) iop(1/8=18)
freq=noraman temp=298.15
#N Geom=AllCheck Guess=TCheck SCRF=Check Test GenChk RM062X/6-31G(d) Freq
----
Pointgroup= C1 Stoichiometry= C27H26N2OS
C1[X(C27H26N2OS)] #Atoms= 57
Charge = 0 Multiplicity = 1

SCF Energy= -1626.89881907 Predicted Change= -6.833766D-09
==
==============================
Optimization completed. {Found 2 times}

Item	Max Val.	Criteria	Pass?	RMS Val.	Criteria	Pass?
Force	0.00002 ||	0.00045	[YES]	0.00000 ||	0.00030	[YES]
Displ	0.00064 ||	0.00180	[YES]	0.00064 ||	0.00180	[YES]

Atomic Type	Coordinates (Angstroms) X	Y	Z
C	-1.874188	4.463343	1.645731
C	-0.779662	3.673077	1.307325
C	-1.010414	2.548235	0.521375
C	-2.296096	2.216634	0.092611
C	-3.387065	3.008359	0.431532
C	-3.162638	4.137892	1.213396
N	-0.052095	1.635742	0.064901
C	-0.559055	0.610511	-0.654970
S	-2.294812	0.734554	-0.858539
C	1.390169	1.855317	0.206244
C	2.122341	1.109259	-0.902488
C	3.659415	1.254770	-0.809362
C	4.109152	2.518121	-1.549456
C	4.219219	1.268171	0.618428
C	1.633805	-0.351366	-0.959859
C	2.071374	-1.193644	0.220546
N	0.167589	-0.372747	-1.140649
C	3.173138	-2.039154	0.080778
C	3.663644	-2.753821	1.171844
C	3.046816	-2.639231	2.414743
C	1.929361	-1.817967	2.558355
C	1.444627	-1.101149	1.468579
C	-0.352106	-1.170252	-2.353716
C	-1.477332	-1.931716	-2.135826
C	-2.063589	-2.380798	-0.876602
O	0.307510	-0.939012	-3.375684
C	-3.450829	-2.608389	-0.791884
C	-4.046810	-3.033339	0.390213
C	-3.278565	-3.237787	1.536687
C	-1.900307	-3.039358	1.467054
C	-1.301672	-2.640391	0.276596
H	-1.718924	5.344896	2.258518
H	0.217015	3.923827	1.653610
H	-4.385617	2.749097	0.096106
H	-4.000308	4.768259	1.491160
H	1.704081	1.524758	1.201254
H	1.565621	2.931777	0.125403
H	3.666102	3.414345	-1.098656
H	3.816418	2.488577	-2.603273
H	5.196847	2.627008	-1.499405
H	4.089802	0.390255	-1.332775
H	3.933829	2.188143	1.142210
H	5.312385	1.245556	0.578410
H	3.886470	0.414850	1.216247
H	1.800358	1.546963	-1.857226
H	2.023034	-0.787278	-1.883249
H	3.653574	-2.136050	-0.889746
H	4.523158	-3.405135	1.047390
H	3.425524	-3.196536	3.265799
H	1.431488	-1.736691	3.519723
H	0.552643	-0.489726	1.590015
H	-1.953469	-2.276255	-3.049959
H	-4.064082	-2.430161	-1.672655
H	-5.120725	-3.197178	0.419844
H	-3.744258	-3.562032	2.462302
H	-1.276744	-3.222172	2.339194
H	-0.222173	-2.552794	0.224864

Statistical Thermodynamic Analysis
Temperature= 298.150 Kelvin Pressure= 1.00000 Atm
==
==============================
SCF Energy= -1626.89881907 Predicted Change= -6.833766D-09
Zero-point correction (ZPE)= -1626.4245 0.47429
Internal Energy (U)= -1626.3983 0.50044
Enthalpy (H)= -1626.3974 0.50138
Gibbs Free Energy (G)= -1626.4809 0.41787

Frequencies -- 24.6722 29.5215 39.6264

183.15K thermal correction = 0.446295
Single point SCF = -1627.326147

J: Tetrahedral Intermediate (Re,Re)

Using Gaussian 09: AM64L-G09RevD.01 24-Apr-2013
==
==============================
#m062x/6-31G(d) gfprint gfinput
scf=(direct,tight,maxcycle=300,xqc)
opt=(maxcycle=250,gdiis) iop(1/8=18) freq=noraman
temp=298.15
SCRF=(PCM,SOLVENT=dichloromethane)
#N Geom=AllCheck Guess=TCheck SCRF=Check Test GenChk
RM062X/6-31G(d) Freq

Pointgroup= C1 Stoichiometry= C35H30BrF3N2O2S
C1[X(C35H30BrF3N2O2S)] #Atoms= 74
Charge = 0 Multiplicity = 1

SCF Energy= -4880.46842385 Predicted Change= -6.642921D-09
==
==============================
Optimization completed. {Found 2 times}

Item	Max Val.	Criteria	Pass?	RMS Val.	Criteria	Pass?
Force	0.00003 ‖	0.00045	[YES]	0.00000 ‖	0.00030	[YES]
Displ	0.00103 ‖	0.00180	[YES]	0.00103 ‖	0.00180	[YES]

Atomic Coordinates (Angstroms)

Type	X	Y	Z
N	3.782341	-0.961380	-0.203268
C	3.182828	-2.300758	-0.206381
C	1.775924	-2.227329	-0.777670
C	1.061724	-3.590341	-0.702412
C	1.939807	-4.712618	-1.265532
C	-0.266875	-3.554854	-1.460809
C	0.985988	-1.100719	-0.088953
C	0.643603	-1.352727	1.366985
N	1.731365	0.179299	-0.251213
C	1.033796	1.429751	-0.363227
C	-0.439125	1.435916	-0.002869
C	-0.779760	2.590039	0.920039
O	1.678670	2.414890	-0.636173
C	3.074951	0.170300	-0.302563
S	4.086273	1.583340	-0.436343
C	5.512547	0.571613	-0.283715
C	5.170503	-0.772715	-0.168264
C	6.846056	0.972296	-0.279632
C	7.820421	-0.009354	-0.147278
C	7.469954	-1.359257	-0.021354
C	6.141158	-1.762994	-0.029672
C	1.564924	-1.141538	2.396264
C	1.214957	-1.388079	3.721829
C	-0.063466	-1.842218	4.035220
C	-0.995075	-2.039605	3.017751
C	-0.644541	-1.791428	1.693348
C	-0.522919	3.932116	0.615053
C	-0.855970	4.936420	1.518973
C	-1.452281	4.623464	2.739430
C	-1.707274	3.292151	3.056372
C	-1.365602	2.287283	2.154768
H	-0.637751	0.537844	0.578758
H	3.190935	-2.697894	0.816045
H	3.823746	-2.923004	-0.832816
H	0.860141	-3.809448	0.354777
H	1.374112	-5.648188	-1.291968
H	2.834547	-4.890778	-0.661780
H	2.254476	-4.482603	-2.290635
H	-0.751471	-4.534670	-1.414882
H	-0.097127	-3.308700	-2.515824
H	-0.967463	-2.820338	-1.056945
H	1.854654	-1.939523	-1.835980
H	0.088077	-0.940689	-0.692810
H	7.112936	2.019287	-0.374432
H	8.866532	0.276526	-0.138638
H	8.247695	-2.106921	0.088406
H	5.874615	-2.808177	0.079830
H	2.559544	-0.759724	2.176691
H	1.941695	-1.216387	4.509394
H	-0.336356	-2.030220	5.068750
H	-2.000215	-2.374543	3.253853
H	-1.387485	-1.912939	0.906751
H	-0.062568	4.188733	-0.331242
H	-0.650079	5.972353	1.266215
H	-1.712456	5.412367	3.438698
H	-2.164998	3.032637	4.006268
H	-1.558418	1.246125	2.408587
C	-1.210191	1.139002	-1.387202
C	-1.551243	2.446523	-2.123555
F	-2.395328	3.272743	-1.465575
F	-2.138708	2.176096	-3.300616
F	-0.446407	3.165100	-2.395579
C	-2.545792	0.439901	-1.024847
O	-0.412706	0.401277	-2.163459
C	-2.775921	-0.803625	-1.611082
C	-3.900646	-1.559925	-1.287793
C	-4.805606	-1.048593	-0.365849
Br	-6.335399	-2.071909	0.106486
C	-4.617350	0.199861	0.217083
C	-3.488713	0.942733	-0.121476
H	-2.037721	-1.158124	-2.323286
H	-4.072014	-2.529429	-1.744262
H	-5.342330	0.585939	0.925433
H	-3.351073	1.919503	0.329991

Statistical Thermodynamic Analysis
Temperature= 298.150 Kelvin Pressure= 1.00000 Atm
==
==============================
SCF Energy= -4880.46842385 Predicted Change= -6.642921D-09
Zero-point correction (ZPE)= -4879.8816 0.58674
Internal Energy (U)= -4879.8443 0.62410
Enthalpy (H)= -4879.8433 0.62504
Gibbs Free Energy (G)= -4879.9522 0.51615

Frequencies -- 14.6079 20.8613 26.6036

183.15K thermal correction = 0.552812
Single point SCF = -4883.525280

J: Tetrahedral Intermediate (Si,Re)

Using Gaussian 09: AM64L-G09RevD.01 24-Apr-2013
==
==============================
# M062X/6-31G* gfprint gfinput
scf=(direct,tight,maxcycle=300,xqc)

opt=(maxcycle=250) freq=noraman iop(1/8=18)
Temperature=298.15
SCRF=(PCM,SOLVENT=Dichloromethane)
#N Geom=AllCheck Guess=TCheck SCRF=Check Test GenChk RM062X/6-31G(d) Freq
----
Pointgroup= C1 Stoichiometry= C35H30BrF3N2O2S
C1[X(C35H30BrF3N2O2S)] #Atoms= 74
Charge = 0 Multiplicity = 1

SCF Energy= -4880.46075000 Predicted Change= -1.101517D-08
==
Optimization completed. {Found 2 times}

Item	Max Val.	Criteria	Pass?	RMS Val.	Criteria	Pass?
Force	0.00002 ||	0.00045	[YES]	0.00000 ||	0.00030	[YES]
Displ	0.00098 ||	0.00180	[YES]	0.00098 ||	0.00180	[YES]

Atomic Coordinates (Angstroms)

Type	X	Y	Z
C	5.116136	4.719363	0.193160
C	4.987924	3.348658	0.005876
C	3.711797	2.852576	-0.248870
C	2.600421	3.691588	-0.304664
C	2.733881	5.064839	-0.122059
C	4.004696	5.568219	0.129019
N	3.362424	1.514205	-0.471355
C	2.053218	1.314144	-0.653779
S	1.136533	2.791443	-0.660262
C	4.361935	0.442555	-0.546667
C	3.762718	-0.759065	-1.264166
C	4.743626	-1.945857	-1.318322
C	6.126386	-1.503884	-1.809615
C	4.213190	-3.052257	-2.234451
C	2.406225	-1.101047	-0.604550
C	2.527340	-1.572492	0.833956
N	1.527317	0.085450	-0.748729
C	0.104092	-0.042962	-0.921752
C	-0.550512	-1.261760	-0.338458
C	-1.413302	-2.031978	-1.304377
O	-0.481755	0.853774	-1.479955
C	2.499942	-0.686817	1.911821
C	2.682953	-1.158445	3.209014
C	2.871983	-2.517204	3.444197
C	2.868238	-3.410404	2.375824
C	2.694507	-2.938429	1.078446
C	-2.276858	-1.416168	-2.218392
C	-3.066092	-2.182803	-3.072392
C	-3.015378	-3.574183	-3.023683
C	-2.163439	-4.197481	-2.114337
C	-1.368199	-3.430560	-1.267777
H	6.097200	5.135499	0.394558
H	5.849093	2.692749	0.066942
H	1.869950	5.718615	-0.168937
H	4.134708	6.634356	0.279347
H	4.688756	0.190283	0.469328
H	5.207580	0.846599	-1.105473
H	4.855187	-2.346262	-0.303023
H	6.631049	-0.841789	-1.100089
H	6.051903	-0.986421	-2.773689
H	6.766552	-2.379548	-1.949156
H	3.240847	-3.442505	-1.921307
H	4.912594	-3.892917	-2.251203
H	4.109658	-2.679364	-3.260058
H	3.544438	-0.457043	-2.298822
H	1.931302	-1.882050	-1.203734
H	0.209389	-1.932330	0.062527
H	2.281770	0.363742	1.755036
H	2.656545	-0.460669	4.039973
H	3.004797	-2.881936	4.458061
H	2.993573	-4.474452	2.550165
H	2.679900	-3.642199	0.248909
H	-2.337195	-0.334030	-2.250777
H	-3.730537	-1.687902	-3.774697
H	-3.635920	-4.168844	-3.687183
H	-2.114887	-5.281229	-2.064722
H	-0.708712	-3.919024	-0.553597
C	-2.671026	1.409338	0.766115
C	-2.571615	0.019170	0.713942
C	-1.175072	-0.572797	1.012140
C	-1.324382	-1.730816	2.022548
F	-0.131590	-2.248092	2.356722
F	-2.065210	-2.778461	1.578222
F	-1.903513	-1.311809	3.159717
C	-3.719762	-0.720529	0.407191
C	-4.935220	-0.086281	0.159955
C	-4.996004	1.300463	0.213943
Br	-6.655661	2.170545	-0.112351
C	-3.873436	2.062663	0.513122
O	-0.298029	0.307915	1.472220
H	-1.769714	1.956169	1.022178
H	-3.682371	-1.802712	0.353699
H	-5.823354	-0.663670	-0.073673
H	-3.943075	3.144751	0.554672

Statistical Thermodynamic Analysis
Temperature= 298.150 Kelvin Pressure= 1.00000 Atm
==
SCF Energy= -4880.46075000 Predicted Change= -1.101517D-08
Zero-point correction (ZPE)= -4879.8761 0.58461
Internal Energy (U)= -4879.8383 0.62243
Enthalpy (H)= -4879.8373 0.62338
Gibbs Free Energy (G)= -4879.9478 0.51287

Frequencies -- 14.0675 20.8703 26.3214

183.15K thermal correction = 0.550112
Single point SCF = -4883.519544

J: Tetrahedral Intermediate (Si,Si)

Using Gaussian 09: AM64L-G09RevD.01 24-Apr-2013
==
# M062X/6-31G* gfprint gfinput scf=(direct,tight,maxcycle=300,xqc)
opt=(maxcycle=250) freq=noraman iop(1/8=18)
Temperature=298.15
SCRF=(PCM,SOLVENT=Dichloromethane)
#N Geom=AllCheck Guess=TCheck SCRF=Check Test GenChk RM062X/6-31G(d) Freq
----
Pointgroup= C1 Stoichiometry= C35H30BrF3N2O2S
C1[X(C35H30BrF3N2O2S)] #Atoms= 74
Charge = 0 Multiplicity = 1

SCF Energy= -4880.46088821 Predicted Change= -3.187508D-08
==

Optimization completed. {Found 1 times}
Item Max Val. Criteria Pass? RMS Val. Criteria Pass?
Force 0.00001 || 0.00045 [YES] 0.00000 || 0.00030 [YES]
Displ 0.00750 || 0.00180 [NO] 0.00750 || 0.00180 [NO]

Atomic Type	Coordinates (Angstroms) X	Y	Z
N	-2.335317	2.015725	-0.180436
C	-2.263058	2.461424	1.216493
C	-0.809367	2.666494	1.613515
C	-0.665497	3.004299	3.109243
C	-1.625327	4.126163	3.519083
C	0.770301	3.420094	3.441966
C	0.016378	1.432616	1.189598
C	-0.336912	0.167388	1.947428
N	-0.112716	1.261013	-0.278350
C	1.022357	0.915870	-1.105495
C	2.224557	0.332465	-0.446144
C	3.404712	1.260759	-0.578651
O	0.916410	1.110019	-2.294334
C	-1.287468	1.552870	-0.870047
S	-1.646509	1.300296	-2.553806
C	-3.318961	1.741214	-2.255602
C	-3.516174	2.115081	-0.928805
C	-4.365965	1.783099	-3.172296
C	-5.612980	2.192338	-2.716679
C	-5.807403	2.557510	-1.378583
C	-4.763441	2.526229	-0.463409
C	-1.473887	-0.585966	1.640366
C	-1.845775	-1.660925	2.441823
C	-1.069439	-2.015060	3.542483
C	0.090822	-1.300236	3.828206
C	0.453683	-0.214505	3.034722
C	3.839964	1.694797	-1.835500
C	4.941486	2.539733	-1.936524
C	5.621029	2.958326	-0.792951
C	5.197264	2.519353	0.459420
C	4.095876	1.673325	0.564165
H	2.035831	0.117625	0.605378
H	-2.762107	1.721301	1.853229
H	-2.813801	3.402201	1.267488
H	-0.920331	2.106537	3.687409
H	-1.434870	4.415782	4.556202
H	-2.674824	3.824730	3.453090
H	-1.479081	5.013483	2.891229
H	1.506576	2.638823	3.232755
H	0.851595	3.667058	4.504217
H	1.051925	4.309493	2.866477
H	-0.420794	3.516571	1.034437
H	1.064505	1.680181	1.358702
H	-4.210369	1.499742	-4.207476
H	-6.447344	2.227699	-3.408466
H	-6.791044	2.870325	-1.046105
H	-4.920210	2.802565	0.573320
H	-2.074271	-0.358536	0.762678
H	-2.721989	-2.245743	2.179972
H	-1.356198	-2.859803	4.161078
H	0.715176	-1.583961	4.669353
H	1.358969	0.342047	3.266953
H	3.316886	1.335956	-2.714224
H	5.275744	2.871311	-2.915434
H	6.478121	3.619798	-0.877216
H	5.724371	2.833028	1.355723
H	3.774551	1.317803	1.541433
C	2.493832	-1.136689	-1.204736
C	3.766607	-1.689169	-0.496399
F	4.886654	-1.167847	-1.007065
F	3.860405	-3.024066	-0.651026
F	3.821043	-1.462195	0.845132
C	1.270230	-1.998206	-0.782858
O	2.685786	-1.059391	-2.484961
C	0.222279	-2.068152	-1.701635
C	-0.936627	-2.789998	-1.423016
C	-1.014524	-3.484785	-0.222935
Br	-2.583724	-4.473169	0.194486
C	0.024174	-3.459433	0.698508
C	1.151887	-2.692522	0.425036
H	0.351208	-1.557249	-2.651210
H	-1.755500	-2.831266	-2.134054
H	-0.057960	-4.011007	1.628639
H	1.947682	-2.660339	1.162052

Statistical Thermodynamic Analysis
Temperature= 298.150 Kelvin Pressure= 1.00000 Atm

===

SCF Energy= -4880.46088821 Predicted Change= -3.187508D-08
Zero-point correction (ZPE)= -4879.8756 0.58521
Internal Energy (U)= -4879.8380 0.62281
Enthalpy (H)= -4879.8371 0.62375
Gibbs Free Energy (G)= -4879.9464 0.51442

Frequencies -- 10.6968 22.4048 31.4219

183.15K thermal correction = 0.551220
Single point SCF = -4883.519505

TS-K: C–O bond formation (Re,Re)

Using Gaussian 09: AM64L-G09RevD.01 24-Apr-2013

===

#m062x/6-31G(d) scf=(maxcycle=300,direct,tight) density=current
SCRF=(PCM,SOLVENT=CH2Cl2)
opt=(maxcycle=250,ts,calcfc,noeigentest,gdiis)
iop(1/8=18) freq=noraman temp=298.15
#N Geom=AllCheck Guess=TCheck SCRF=Check Test GenChk RM062X/6-31G(d) Freq

---

Pointgroup= C1 Stoichiometry= C35H30BrF3N2O2S
C1[X(C35H30BrF3N2O2S)] #Atoms= 74
Charge = 0 Multiplicity = 1

SCF Energy= -4880.46685695 Predicted Change= -1.751084D-10

===

Optimization completed. {Found 2 times}
Item Max Val. Criteria Pass? RMS Val. Criteria Pass?
Force 0.00000 || 0.00045 [YES] 0.00000 || 0.00030 [YES]
Displ 0.00062 || 0.00180 [YES] 0.00062 || 0.00180 [YES]

Atomic Type	Coordinates (Angstroms) X	Y	Z
N	3.637901	-1.140278	-0.180804
C	2.965485	-2.442911	-0.114492
C	1.592207	-2.335089	-0.760037

C	0.800689	-3.652288	-0.652029
C	1.644732	-4.847818	-1.106449
C	-0.480622	-3.585984	-1.486560
C	0.840882	-1.125910	-0.170186
C	0.439916	-1.280992	1.285916
N	1.658244	0.095107	-0.389385
C	1.015441	1.381009	-0.646212
C	-0.375384	1.574569	-0.035754
C	-0.495232	2.781553	0.869629
O	1.724501	2.285538	-1.030019
C	2.990479	0.013509	-0.404125
S	4.080160	1.357268	-0.641840
C	5.444868	0.297513	-0.323379
C	5.031857	-1.014106	-0.104517
C	6.794243	0.635657	-0.295676
C	7.714827	-0.372476	-0.033180
C	7.293959	-1.687637	0.193682
C	5.946948	-2.029619	0.161868
C	1.322667	-1.014993	2.335640
C	0.911188	-1.159055	3.659125
C	-0.389474	-1.563644	3.948273
C	-1.281778	-1.816214	2.907677
C	-0.870332	-1.671234	1.585843
C	-0.114992	4.074149	0.490028
C	-0.248984	5.136948	1.378422
C	-0.765496	4.931664	2.657032
C	-1.139645	3.649261	3.048514
C	-0.997095	2.584737	2.161692
H	-0.585830	0.714805	0.598539
H	2.902566	-2.765309	0.932007
H	3.597358	-3.143818	-0.662742
H	0.530148	-3.803211	0.401746
H	1.028645	-5.751095	-1.128042
H	2.487446	-5.044649	-0.437317
H	2.037156	-4.685370	-2.117626
H	-1.021146	-4.534928	-1.422559
H	-0.240011	-3.402748	-2.540651
H	-1.160601	-2.795911	-1.158683
H	1.740940	-2.116150	-1.827365
H	-0.039545	-0.946297	-0.792809
H	7.114807	1.656993	-0.471718
H	8.772366	-0.133949	-0.003160
H	8.029292	-2.457664	0.400048
H	5.624653	-3.047921	0.348616
H	2.333809	-0.669495	2.131554
H	1.607634	-0.945162	4.463690
H	-0.710520	-1.669370	4.979673
H	-2.304240	-2.111950	3.121828
H	-1.583050	-1.833847	0.779067
H	0.295771	4.239551	-0.497745
H	0.051847	6.133502	1.069064
H	-0.870960	5.765372	3.344641
H	-1.535540	3.474112	4.044331
H	-1.277475	1.579710	2.474664
C	-1.221778	1.320371	-1.332896
C	-1.670031	2.610024	-2.029646
F	-2.476695	3.385701	-1.273400
F	-2.354424	2.323665	-3.150807
F	-0.628970	3.373598	-2.398119
C	-2.485406	0.522453	-0.973288
O	-0.357064	0.652073	-2.143589
C	-2.759083	-0.640008	-1.690375
C	-3.839168	-1.453568	-1.350506
C	-4.646017	-1.083347	-0.281066
Br	-6.117239	-2.183680	0.199846
C	-4.404265	0.080153	0.442174
C	-3.325142	0.882720	0.084404
H	-2.090518	-0.901220	-2.505119
H	-4.049160	-2.362668	-1.904551
H	-5.047869	0.351799	1.271864
H	-3.131668	1.791654	0.647405

Statistical Thermodynamic Analysis
Temperature= 298.150 Kelvin   Pressure= 1.00000 Atm
==
SCF Energy= -4880.46685695   Predicted Change= -1.751084D-10
Zero-point correction (ZPE)= -4879.8819 0.58495
Internal Energy (U)= -4879.8447 0.62207
Enthalpy (H)= -4879.8438 0.62301
Gibbs Free Energy (G)= -4879.9532 0.51360

Frequencies -- -198.6191 10.7846 19.5330

183.15K thermal correction = 0.550511
Single point SCF = -4883.522045

TS-K: C–O bond formation (Si,Re)

Using Gaussian 09: AM64L-G09RevD.01 24-Apr-2013
==
#m062x/6-31G(d) scf=(maxcycle=300,direct,tight) density=current
SCRF=(PCM,SOLVENT=CH2Cl2) opt=(maxcycle=250,ts,calcfc,noeigentest,gdiis)
iop(1/8=18) freq=noraman temp=298.15
#N Geom=AllCheck Guess=TCheck SCRF=Check Test GenChk RM062X/6-31G(d) Freq

----

Pointgroup= C1 Stoichiometry= C35H30BrF3N2O2S
C1[X(C35H30BrF3N2O2S)] #Atoms= 74
Charge = 0 Multiplicity = 1

SCF Energy= -4880.46112948 Predicted Change= -3.013245D-09
==
Optimization completed. {Found 2 times}

Item	Max Val.	Criteria	Pass?	RMS Val.	Criteria	Pass?
Force	0.00001 ||	0.00045	[YES]	0.00000 ||	0.00030	[YES]
Displ	0.00149 ||	0.00180	[YES]	0.00149 ||	0.00180	[YES]

Atomic Type	Coordinates (Angstroms) X	Y	Z
N	-3.749050	0.996060	0.067733
C	-4.346325	-0.287555	0.461738
C	-3.283181	-1.239355	0.996970
C	-3.844492	-2.661811	1.174886
C	-5.162304	-2.648070	1.955713
C	-2.834309	-3.560325	1.891890
C	-2.042747	-1.184194	0.084812
C	-2.269927	-1.720298	-1.319945
N	-1.544555	0.211653	0.081997
C	-0.138108	0.543718	0.220384
C	0.695753	-0.304609	1.177768
C	1.453266	0.474827	2.227193
O	0.227103	1.601053	-0.239620
C	-2.432232	1.200303	-0.073042
S	-2.044683	2.849616	-0.484505
C	-3.754201	3.254030	-0.445610
C	-4.533727	2.135958	-0.156428

C	-4.342172	4.493236	-0.680401
C	-5.728581	4.577918	-0.625201
C	-6.507453	3.449979	-0.341597
C	-5.923376	2.211918	-0.102519
C	-2.967365	-1.033297	-2.316389
C	-3.189829	-1.622566	-3.561301
C	-2.725178	-2.906852	-3.823234
C	-2.015458	-3.593567	-2.839450
C	-1.781185	-2.998838	-1.605058
C	2.183984	1.636300	1.946280
C	2.905978	2.275934	2.950369
C	2.925818	1.761980	4.245352
C	2.213271	0.600437	4.532956
C	1.481338	-0.031210	3.531094
C	1.457352	-1.178203	0.098326
C	2.822914	-0.572781	-0.278826
C	1.681218	-2.574584	0.702860
F	2.325118	-2.563511	1.892873
F	2.377023	-3.373417	-0.114586
F	0.502254	-3.198445	0.940228
O	0.594329	-1.267070	-0.932593
C	2.926534	-0.022157	-1.554666
C	4.110094	0.565143	-1.994106
C	5.203240	0.586079	-1.137855
Br	6.837205	1.356598	-1.734142
C	5.133470	0.044295	0.140115
C	3.938530	-0.529224	0.565592
H	0.022741	-0.981984	1.711722
H	-4.854683	-0.710275	-0.413617
H	-5.090418	-0.065128	1.229053
H	-4.038437	-3.074832	0.175744
H	-5.494990	-3.672780	2.144620
H	-5.965694	-2.139302	1.414531
H	-5.033258	-2.153317	2.926128
H	-3.247208	-4.565547	2.016006
H	-2.613243	-3.162504	2.889880
H	-1.889507	-3.655927	1.351378
H	-2.964451	-0.871861	1.983350
H	-1.240594	-1.778268	0.511000
H	-3.735389	5.363757	-0.905017
H	-6.211305	5.531847	-0.807066
H	-7.587694	3.539477	-0.306779
H	-6.529940	1.339756	0.114144
H	-3.332404	-0.022863	-2.154902
H	-3.726725	-1.069429	-4.325637
H	-2.900382	-3.364332	-4.791987
H	-1.624648	-4.586330	-3.040349
H	-1.175524	-3.508890	-0.862347
H	2.191004	2.033967	0.937890
H	3.465158	3.176484	2.714632
H	3.494261	2.261399	5.024115
H	2.222631	0.186990	5.537029
H	0.929925	-0.940070	3.760266
H	2.050254	-0.075531	-2.190893
H	4.187391	0.992231	-2.988316
H	5.998680	0.071819	0.793420
H	3.887800	-0.936202	1.570004

Statistical Thermodynamic Analysis
Temperature= 298.150 Kelvin Pressure= 1.00000 Atm
==
==============================
SCF Energy= -4880.46112948 Predicted Change= -3.013245D-09
Zero-point correction (ZPE)= -4879.8757 0.58536
Internal Energy (U)= -4879.8386 0.62244
Enthalpy (H)= -4879.8377 0.62339
Gibbs Free Energy (G)= -4879.9469 0.51416

Frequencies -- -103.0372 18.0861 23.8775

183.15K thermal correction = 0.551005
Single point SCF = -4883.516236

TS-K: C–O bond formation (Si,Si)

Using Gaussian 09: AM64L-G09RevD.01 24-Apr-2013
==
==============================
#m062x/6-31G(d) scf=(maxcycle=300,direct,tight) density=current
opt=(maxcycle=250,ts,calcfc,noeigentest,gdiis) iop(1/8=18) freq=noraman
temp=298.15 SCRF=(PCM,SOLVENT=dichloromethane)
#N Geom=AllCheck Guess=TCheck SCRF=Check Test GenChk RM062X/6-31G(d) Freq
----
Pointgroup= C1 Stoichiometry= C35H30BrF3N2O2S
C1[X(C35H30BrF3N2O2S)] #Atoms= 74
Charge = 0 Multiplicity = 1

SCF Energy= -4880.44435372 Predicted Change= -1.767556D-08
==
==============================
Optimization completed. {Found 2 times}

Item	Max Val.	Criteria	Pass?	RMS Val.	Criteria	Pass?
Force	0.00001 ||	0.00045	[YES]	0.00000 ||	0.00030	[YES]
Displ	0.00141 ||	0.00180	[YES]	0.00141 ||	0.00180	[YES]

Atomic Coordinates (Angstroms)

Type	X	Y	Z
N	-3.233148	0.498248	-1.288690
C	-3.427601	1.895671	-0.884048
C	-2.071146	2.533658	-0.610474
C	-2.189184	3.997788	-0.123897
C	-3.375274	4.272790	0.808683
C	-2.250358	4.944365	-1.326555
C	-1.240961	1.636439	0.326927
C	-1.772704	1.555044	1.740677
N	-1.112293	0.290576	-0.289342
C	0.292936	-0.158496	-0.619550
C	0.892347	-1.414060	0.035834
C	-0.098808	-2.362513	0.662682
O	0.798148	0.403798	-1.553064
C	-2.095309	-0.173352	-1.042796
S	-2.072410	-1.745220	-1.805757
C	-3.668727	-1.445864	-2.480712
C	-4.156998	-0.204107	-2.073548
C	-4.424475	-2.284761	-3.291087
C	-5.686405	-1.848242	-3.683036
C	-6.177634	-0.607278	-3.267064
C	-5.422177	0.231790	-2.454918
C	-1.166544	2.334516	2.726931
C	-1.688335	2.377346	4.016626
C	-2.814761	1.623369	4.339999
C	-3.405495	0.814948	3.371165
C	-2.887719	0.782003	2.078327
C	-0.145330	-3.690694	0.231172
C	-1.095542	-4.573008	0.741711
C	-2.014675	-4.132226	1.690619
C	-1.958508	-2.814526	2.145758
C	-1.001038	-1.939218	1.644812

C	1.817773	-0.586633	0.998138
C	2.223859	-1.369985	2.262153
F	2.629258	-2.628438	1.973268
F	1.240778	-1.476244	3.162476
F	3.247368	-0.769721	2.897763
C	3.139003	-0.255338	0.265729
O	1.071162	0.503278	1.302636
C	3.974747	-1.232967	-0.279172
C	5.151850	-0.881342	-0.933826
C	5.487210	0.463856	-1.035125
Br	7.075780	0.955655	-1.956851
C	4.678904	1.455168	-0.492817
C	3.505534	1.082897	0.157615
H	1.443927	-1.941828	-0.748965
H	-4.077468	1.913863	-0.003784
H	-3.937133	2.403098	-1.707493
H	-1.266202	4.218399	0.428858
H	-3.293548	5.288425	1.207160
H	-3.422712	3.582502	1.655747
H	-4.324829	4.215600	0.263373
H	-1.360331	4.849212	-1.955778
H	-2.324161	5.983915	-0.993269
H	-3.129585	4.732164	-1.946556
H	-1.516969	2.539867	-1.559125
H	-0.219858	2.015620	0.368325
H	-4.040663	-3.248408	-3.607270
H	-6.295352	-2.483101	-4.317067
H	-7.166150	-0.289082	-3.579519
H	-5.810635	1.188922	-2.125162
H	-0.272418	2.900177	2.480372
H	-1.208137	2.990747	4.772681
H	-3.220811	1.653082	5.346247
H	-4.270197	0.207422	3.620184
H	-3.345443	0.122747	1.342940
H	0.559039	-4.032682	-0.523837
H	-1.121593	-5.600543	0.391766
H	-2.763852	-4.814228	2.081377
H	-2.659200	-2.464265	2.899085
H	-0.924377	-0.923965	2.017830
H	3.714937	-2.285097	-0.199377
H	5.797322	-1.639506	-1.364056
H	4.960525	2.499027	-0.583420
H	2.838387	1.822544	0.587092

Statistical Thermodynamic Analysis
Temperature= 298.150 Kelvin Pressure= 1.00000 Atm
==
==============================
SCF Energy= -4880.44435372 Predicted Change= -1.767556D-08
Zero-point correction (ZPE)= -4879.8592 0.58506
Internal Energy (U)= -4879.8223 0.62205
Enthalpy (H)= -4879.8213 0.62299
Gibbs Free Energy (G)= -4879.9288 0.51547

Frequencies -- -178.8855 20.4693 27.6730

183.15K thermal correction = 0.551652
Single point SCF = -4883.500615

F: Product-catalyst complex (Re,Re)

Using Gaussian 09: AM64L-G09RevD.01 24-Apr-2013
==
==============================
#m062X/6-31G(d) scf=(direct,tight,maxcycle=300,xqc)
SCRF=(PCM,SOLVENT=CH2Cl2) opt=(maxcycle=250,gdiis) iop(1/8=18)
freq=noraman temp=298.15
#N Geom=AllCheck Guess=TCheck SCRF=Check Test GenChk RM062X/6-31G(d) Freq
----
Pointgroup= C1 Stoichiometry= C35H30BrF3N2O2S
C1[X(C35H30BrF3N2O2S)] #Atoms= 74
Charge = 0 Multiplicity = 1

SCF Energy= -4880.48047793 Predicted Change= -5.845818D-09
==
==============================
Optimization completed. {Found 1 times}

Item	Max Val.	Criteria	Pass?	RMS Val.	Criteria	Pass?
Force	0.00001 ||	0.00045	[YES]	0.00000 ||	0.00030	[YES]
Displ	0.00206 ||	0.00180	[NO]	0.00206 ||	0.00180	[YES]

Atomic Type	Coordinates (Angstroms) X	Y	Z
N	3.235595	-1.534621	-0.215116
C	2.480848	-2.788021	-0.279231
C	1.213827	-2.579765	-1.098609
C	0.316359	-3.829251	-1.110540
C	1.114204	-5.085896	-1.474842
C	-0.840318	-3.661087	-2.101121
C	0.507809	-1.302220	-0.589121
C	-0.018264	-1.423093	0.836523
N	1.422163	-0.161545	-0.760368
C	0.905845	1.293413	-1.141350
C	-0.072786	1.817132	-0.027139
C	0.235399	3.078879	0.727473
O	1.843049	1.979570	-1.610061
C	2.704956	-0.336000	-0.544041
S	3.916196	0.941461	-0.562164
C	5.123998	-0.214662	0.004328
C	4.593159	-1.497912	0.123626
C	6.463896	0.024104	0.287065
C	7.252757	-1.044659	0.706060
C	6.710422	-2.326265	0.833244
C	5.371084	-2.572105	0.543955
C	0.737785	-1.063540	1.957171
C	0.200526	-1.176718	3.238106
C	-1.099810	-1.645381	3.418829
C	-1.860563	-2.005083	2.309299
C	-1.319230	-1.894346	1.031994
C	0.895787	4.174012	0.157023
C	1.158360	5.309613	0.918966
C	0.770388	5.374459	2.255958
C	0.127418	4.284532	2.837486
C	-0.128621	3.146255	2.078147
H	-0.279741	1.032205	0.702832
H	2.251229	-3.125328	0.739888
H	3.132708	-3.525037	-0.753706
H	-0.095678	-3.967195	-0.102447
H	0.438771	-5.940029	-1.578629
H	1.855429	-5.345310	-0.713255
H	1.634457	-4.951378	-2.430904
H	-1.477062	-4.550569	-2.093953
H	-0.452091	-3.532200	-3.118359
H	-1.476135	-2.799370	-1.876105
H	1.515040	-2.372352	-2.135524
H	-0.332999	-1.084676	-1.249602
H	6.882274	1.020294	0.186542
H	8.299301	-0.876758	0.936594

H	7.338571	-3.146412	1.164122
H	4.949544	-3.565453	0.652818
H	1.740310	-0.659018	1.841164
H	0.798679	-0.886960	4.096350
H	-1.518282	-1.721188	4.417484
H	-2.885329	-2.349711	2.422874
H	-1.929629	-2.157976	0.173146
H	1.218565	4.114122	-0.873780
H	1.673451	6.150336	0.463447
H	0.977393	6.264503	2.842562
H	-0.167249	4.315172	3.882208
H	-0.609136	2.286736	2.542494
C	-1.157531	1.692567	-1.128185
C	-1.622608	2.994160	-1.770889
F	-2.276046	3.760722	-0.883273
F	-2.465065	2.746383	-2.787306
F	-0.611934	3.710593	-2.268274
C	-2.364866	0.864783	-0.744998
O	-0.274942	1.025879	-2.023230
C	-2.831792	-0.135750	-1.596665
C	-3.874138	-0.970089	-1.198867
C	-4.441252	-0.780817	0.056890
Br	-5.733972	-2.010897	0.694089
C	-4.037542	0.255767	0.890725
C	-2.998207	1.081454	0.478481
H	-2.351744	-0.284422	-2.559962
H	-4.218428	-1.774136	-1.840713
H	-4.503520	0.391436	1.860445
H	-2.654387	1.878155	1.133343

Statistical Thermodynamic Analysis
Temperature= 298.150 Kelvin Pressure= 1.00000 Atm
===
=============================
SCF Energy= -4880.48047793 Predicted Change= -5.845818D-09
Zero-point correction (ZPE)= -4879.8936 0.58681
Internal Energy (U)= -4879.8566 0.62380
Enthalpy (H)= -4879.8557 0.62474
Gibbs Free Energy (G)= -4879.9639 0.51650

Frequencies -- 7.2337 25.0185 30.5485

183.15K thermal correction = 0.552943
Single point SCF = -4883.530863

F: Product-catalyst complex (Si,Re)

Using Gaussian 09: AM64L-G09RevD.01 24-Apr-2013
===
=============================
#m062X/6-31G(d) scf=(direct,tight,maxcycle=300,xqc)
SCRF=(PCM,SOLVENT=CH2Cl2) opt=(maxcycle=250,gdiis) iop(1/8=18)
freq=noraman temp=298.15
#N Geom=AllCheck Guess=TCheck SCRF=Check Test GenChk RM062X/6-31G(d) Freq

----

Pointgroup= C1 Stoichiometry= C35H30BrF3N2O2S
C1[X(C35H30BrF3N2O2S)] #Atoms= 74
Charge = 0 Multiplicity = 1

SCF Energy= -4880.47711884 Predicted Change= -6.486395D-09
===
=============================
Optimization completed. {Found 2 times}

Item	Max Val.	Criteria	Pass?	RMS Val.	Criteria	Pass?
Force	0.00001 ||	0.00045	[YES]	0.00000 ||	0.00030	[YES]
Displ	0.00128 ||	0.00180	[YES]	0.00128 ||	0.00180	[YES]

Atomic Type	Coordinates (Angstroms) X	Y	Z
N	-3.675372	1.014784	-0.213593
C	-4.368473	-0.131171	0.378817
C	-3.377440	-1.009759	1.131213
C	-4.029980	-2.306771	1.640754
C	-5.346872	-2.022673	2.370177
C	-3.082364	-3.061508	2.576956
C	-2.136618	-1.254637	0.248104
C	-2.388518	-2.131894	-0.967047
N	-1.551965	0.037675	-0.134857
C	-0.014428	0.222537	-0.208618
C	0.669926	0.021177	1.200998
C	1.335230	1.170059	1.891702
O	0.294790	1.191129	-0.950380
C	-2.337299	1.059500	-0.392325
S	-1.796479	2.614436	-1.006542
C	-3.466607	3.184325	-0.990432
C	-4.347615	2.194121	-0.560821
C	-3.939509	4.435941	-1.367838
C	-5.310780	4.670317	-1.308920
C	-6.188837	3.669619	-0.884239
C	-5.720380	2.414959	-0.504744
C	-2.992323	-1.644390	-2.129286
C	-3.244508	-2.492865	-3.205092
C	-2.894069	-3.838594	-3.133971
C	-2.274915	-4.328673	-1.986595
C	-2.018969	-3.478228	-0.914713
C	2.085820	2.127646	1.196040
C	2.702661	3.170134	1.882206
C	2.587985	3.271975	3.267803
C	1.846130	2.324401	3.968566
C	1.225384	1.283976	3.281579
C	1.468832	-1.077876	0.446372
C	2.853191	-0.685726	-0.035690
C	1.525405	-2.423997	1.160669
F	2.240126	-2.347740	2.294485
F	2.074829	-3.369018	0.391754
F	0.302275	-2.869263	1.512129
O	0.509680	-1.133745	-0.600550
C	3.072132	-0.495886	-1.398535
C	4.321390	-0.098550	-1.867528
C	5.348236	0.109421	-0.955602
Br	7.048600	0.672942	-1.581756
C	5.153116	-0.069952	0.409075
C	3.900335	-0.466302	0.864291
H	-0.016766	-0.473574	1.896836
H	-4.883012	-0.690857	-0.412757
H	-5.117462	0.271918	1.063289
H	-4.248910	-2.940880	0.771169
H	-5.737337	-2.944939	2.809799
H	-6.117419	-1.627558	1.701655
H	-5.193357	-1.302039	3.182781
H	-3.553040	-3.984189	2.928654
H	-2.848185	-2.447665	3.455124
H	-2.137205	-3.334809	2.100359
H	-3.027589	-0.440136	2.005373
H	-1.378848	-1.744649	0.853402
H	-3.255027	5.208537	-1.702803
H	-5.699685	5.639777	-1.601416
H	-7.254795	3.867736	-0.850933
H	-6.405808	1.637822	-0.184871

H	-3.253781	-0.592854	-2.217716
H	-3.709420	-2.096782	-4.102520
H	-3.090315	-4.498372	-3.973358
H	-1.978242	-5.371396	-1.929285
H	-1.504820	-3.857620	-0.035701
H	2.166852	2.049015	0.118208
H	3.279795	3.906508	1.330648
H	3.075263	4.085070	3.797579
H	1.749786	2.393410	5.048012
H	0.648370	0.544064	3.831946
H	2.247589	-0.638741	-2.086792
H	4.492257	0.054072	-2.927397
H	5.963147	0.109452	1.107123
H	3.739539	-0.580285	1.931044

Statistical Thermodynamic Analysis
Temperature= 298.150 Kelvin Pressure= 1.00000 Atm
==
==============================
SCF Energy= -4880.47711884 Predicted Change= -6.486395D-09
Zero-point correction (ZPE)= -4879.8914 0.58569
Internal Energy (U)= -4879.8540 0.62311
Enthalpy (H)= -4879.8530 0.62405
Gibbs Free Energy (G)= -4879.9641 0.51295

Frequencies -- 13.0991 17.2418 25.1432

183.15K thermal correction = 0.550483
Single point SCF = -4883.528244

F: Product-catalyst complex (Si,Si)

Using Gaussian 09: AM64L-G09RevD.01 24-Apr-2013
==
==============================
#m062x/6-31G(d) gfprint gfinput scf=(direct,tight,maxcycle=300,xqc)
opt=(maxcycle=250,gdiis) iop(1/8=18) freq=noraman temp=298.15
SCRF=(PCM,SOLVENT=dichloromethane)
#N Geom=AllCheck Guess=TCheck SCRF=Check Test GenChk RM062X/6-31G(d) Freq
----
Pointgroup= C1 Stoichiometry= C35H30BrF3N2O2S C1[X(C35H30BrF3N2O2S)] #Atoms= 74
Charge = 0 Multiplicity = 1

SCF Energy= -4880.46481161 Predicted Change= -3.875149D-09
==
==============================
Optimization completed. {Found 2 times}

Item	Max Val.	Criteria	Pass?	RMS Val.	Criteria	Pass?
Force	0.00000 ||	0.00045	[YES]	0.00000 ||	0.00030	[YES]
Displ	0.00143 ||	0.00180	[YES]	0.00143 ||	0.00180	[YES]

Atomic Type	Coordinates (Angstroms) X	Y	Z
N	-3.121574	-1.288305	-0.368709
C	-4.051265	-0.167661	-0.523017
C	-3.294769	1.154049	-0.577631
C	-4.253014	2.357384	-0.528937
C	-5.413442	2.193065	-1.516704
C	-3.515345	3.663378	-0.836300
C	-2.226175	1.154297	0.539366
C	-2.822823	1.155519	1.941441
N	-1.308201	0.024063	0.311448
C	0.129520	0.269243	0.989426
C	1.038951	1.146112	0.032232
C	0.565486	2.216444	-0.915166
O	0.020829	0.507603	2.196045
C	-1.826276	-1.139534	0.002272
S	-1.000676	-2.695254	0.087235
C	-2.488915	-3.516595	-0.364879
C	-3.529418	-2.610854	-0.567216
C	-2.690284	-4.881314	-0.531942
C	-3.962068	-5.320587	-0.891425
C	-5.004702	-4.410431	-1.081747
C	-4.804171	-3.042049	-0.922742
C	-3.084783	2.385714	2.550060
C	-3.687621	2.451886	3.803325
C	-4.032371	1.281391	4.473823
C	-3.761191	0.049798	3.883126
C	-3.159684	-0.012968	2.628869
C	0.921278	3.539856	-0.617197
C	0.515057	4.600974	-1.422124
C	-0.252601	4.359243	-2.557782
C	-0.616542	3.050833	-2.867302
C	-0.221158	1.992743	-2.053311
C	1.708858	-0.197769	-0.356766
C	1.511668	-0.756666	-1.764420
F	2.093318	-1.959243	-1.875166
F	2.047776	0.044689	-2.695967
F	0.218588	-0.935844	-2.084889
C	3.192076	-0.256346	-0.041485
O	0.921626	-0.946795	0.555383
C	4.061747	0.686284	-0.593304
C	5.423090	0.638236	-0.313618
C	5.901342	-0.363291	0.524770
Br	7.755772	-0.430316	0.920343
C	5.051920	-1.309109	1.085486
C	3.691441	-1.251058	0.795046
H	1.724245	1.623599	0.737189
H	-4.602254	-0.333299	-1.451654
H	-4.761718	-0.185448	0.314397
H	-4.675913	2.414017	0.482065
H	-6.076517	1.365113	-1.248856
H	-5.037088	2.018776	-2.532302
H	-6.018511	3.104304	-1.536650
H	-4.175966	4.518991	-0.665855
H	-3.201721	3.680927	-1.885792
H	-2.615895	3.807989	-0.229066
H	-2.750013	1.196425	-1.531067
H	-1.608049	2.049324	0.435715
H	-1.878653	-5.585311	-0.381244
H	-4.142512	-6.382128	-1.021775
H	-5.989726	-4.770961	-1.358141
H	-5.617098	-2.338838	-1.066358
H	-2.804774	3.304981	2.040794
H	-3.876787	3.419360	4.258291
H	-4.497796	1.328563	5.453383
H	-4.009330	-0.870210	4.403326
H	-2.932770	-0.989116	2.210538
H	1.529564	3.738649	0.261476
H	0.806940	5.614274	-1.163658
H	-0.565073	5.181215	-3.194419
H	-1.218717	2.846773	-3.748265
H	-0.527578	0.989952	-2.318638
H	3.675060	1.470072	-1.239701
H	6.103030	1.370398	-0.734732
H	5.446508	-2.076591	1.741911
H	3.006639	-1.971140	1.229589

Statistical Thermodynamic Analysis
Temperature= 298.150 Kelvin Pressure= 1.00000 Atm
==
==============================
SCF Energy= -4880.46481161 Predicted Change= -3.875149D-09
Zero-point correction (ZPE)= -4879.8797 0.58505
Internal Energy (U)= -4879.8422 0.62254
Enthalpy (H)= -4879.8413 0.62349
Gibbs Free Energy (G)= -4879.9512 0.51359

Frequencies -- 15.4153 26.1363 27.6666

183.15K thermal correction = 0.550637
Single point SCF = -4883.516679

TS-G: Catalyst release (Re,Re)

Using Gaussian 09: AM64L-G09RevD.01 24-Apr-2013
==
==============================
#m062x/6-31G(d) scf=(maxcycle=300,direct,tight) density=current
opt=(maxcycle=250,ts,calcfc,noeigentest,gdiis) iop(1/8=18) freq=noraman
temp=298.15 SCRF=(PCM,SOLVENT=dichloromethane)
#N Geom=AllCheck Guess=TCheck SCRF=Check Test GenChk RM062X/6-31G(d) Freq
----
Pointgroup= C1 Stoichiometry= C35H30BrF3N2O2S C1[X(C35H30BrF3N2O2S)] #Atoms= 74
Charge = 0 Multiplicity = 1

SCF Energy= -4880.47359971 Predicted Change= 2.199179D-09
==
==============================
Optimization completed. {Found 2 times}

Item	Max Val.		Criteria	Pass?	RMS Val.		Criteria	Pass?
Force	0.00000	‖	0.00045	[YES]	0.00000	‖	0.00030	[YES]
Displ	0.00127	‖	0.00180	[YES]	0.00127	‖	0.00180	[YES]

Atomic Type	Coordinates (Angstroms) X	Y	Z
N	-2.984714	-1.492874	-0.338326
C	-2.677908	-2.791604	0.263419
C	-1.986907	-2.567475	1.607537
C	-1.627528	-3.893951	2.299445
C	-2.837736	-4.833231	2.367360
C	-1.107420	-3.646110	3.718301
C	-0.802169	-1.585239	1.394810
C	0.293663	-2.148043	0.496446
N	-1.338484	-0.325589	0.867355
C	-0.867932	1.212645	1.489622
C	-0.169017	1.948678	0.288880
C	-0.595339	3.335293	-0.112936
O	-1.768797	1.655206	2.201648
C	-2.376314	-0.359445	0.091877
S	-3.145081	1.083832	-0.565196
C	-4.227723	0.071454	-1.518995
C	-4.011759	-1.285178	-1.261529
C	-5.211110	0.482207	-2.408543
C	-5.973813	-0.494652	-3.045744
C	-5.752694	-1.849696	-2.791673
C	-4.768544	-2.263931	-1.897461
C	0.407974	-1.782905	-0.846635
C	1.397366	-2.338595	-1.656389
C	2.278754	-3.284469	-1.141057
C	2.181585	-3.647635	0.200615
C	1.208134	-3.074107	1.013026
C	-1.206556	4.234025	0.768971
C	-1.580116	5.502843	0.331230
C	-1.352298	5.895660	-0.985576
C	-0.760653	5.002121	-1.875454
C	-0.396510	3.731523	-1.440682
H	-0.147591	1.314840	-0.601788
H	-2.049376	-3.379458	-0.417556
H	-3.629707	-3.308782	0.401403
H	-0.847889	-4.394522	1.712368
H	-2.595443	-5.710098	2.974965
H	-3.143111	-5.194300	1.381257
H	-3.694730	-4.329950	2.831423
H	-0.842917	-4.594496	4.195242
H	-1.884302	-3.168447	4.326847
H	-0.223427	-3.002877	3.747400
H	-2.699241	-2.040633	2.259696
H	-0.364184	-1.330531	2.363945
H	-5.379179	1.536450	-2.601486
H	-6.745582	-0.194598	-3.746242
H	-6.352364	-2.597473	-3.299588
H	-4.590748	-3.317461	-1.710342
H	-0.269807	-1.046584	-1.270017
H	1.478933	-2.026027	-2.693299
H	3.057009	-3.705946	-1.770066
H	2.879250	-4.364559	0.623284
H	1.169422	-3.342283	2.065599
H	-1.401239	3.925782	1.788322
H	-2.054721	6.188657	1.026829
H	-1.643048	6.887314	-1.318698
H	-0.590409	5.288906	-2.908802
H	0.039598	3.028327	-2.147210
C	1.127901	1.663970	1.091737
C	1.735832	2.868359	1.805109
F	2.204280	3.763384	0.922062
F	2.762070	2.491094	2.583733
F	0.855942	3.488940	2.596030
C	2.220167	0.922406	0.360430
O	0.442424	0.873184	2.062100
C	2.881047	-0.129587	0.991177
C	3.872513	-0.835718	0.321756
C	4.187759	-0.477335	-0.983350
Br	5.441259	-1.526082	-1.942295
C	3.571504	0.593858	-1.617163
C	2.588591	1.299478	-0.929521
H	2.590151	-0.417658	1.996566
H	4.370585	-1.675544	0.792701
H	3.841752	0.860878	-2.632752
H	2.095806	2.140293	-1.410400

Statistical Thermodynamic Analysis
Temperature= 298.150 Kelvin Pressure= 1.00000 Atm
==
==============================
SCF Energy= -4880.47359971 Predicted Change= 2.199179D-09
Zero-point correction (ZPE)= -4879.8889 0.58463
Internal Energy (U)= -4879.8521 0.62147
Enthalpy (H)= -4879.8511 0.62242
Gibbs Free Energy (G)= -4879.9590 0.51455

Frequencies -- -55.5479 13.2478 24.6150

183.15K thermal correction = 0.550880
Single point SCF = -4883.525472

TS-G: Catalyst release (Si,Re)

Using Gaussian 09: AM64L-G09RevD.01 24-Apr-2013
==
==============================
#m062x/6-31G(d) scf=(maxcycle=300,direct,tight)
density=current
SCRF=(PCM,SOLVENT=CH2Cl2)
opt=(maxcycle=250,ts,calcfc,noeigentest,gdiis)
iop(1/8=18) freq=noraman temp=298.15
#N Geom=AllCheck Guess=TCheck SCRF=Check Test GenChk
RM062X/6-31G(d) Freq

Pointgroup= C1 Stoichiometry= C35H30BrF3N2O2S
C1[X(C35H30BrF3N2O2S)] #Atoms= 74
Charge = 0 Multiplicity = 1

SCF Energy= -4880.47409106 Predicted Change= -1.425042D-09
==
==============================
Optimization completed on the basis of negligible forces.
{Found 2 times}
Item Max Val. Criteria Pass? RMS Val. Criteria Pass?
Force 0.00000 || 0.00045 [YES] 0.00000 || 0.00030 [YES]
Displ 0.00265 || 0.00180 [NO] 0.00265 || 0.00180 [YES]

Atomic Coordinates (Angstroms)

Type	X	Y	Z
N	-3.878220	0.551010	0.174098
C	-4.381069	-0.756185	0.597787
C	-3.224213	-1.609966	1.117645
C	-3.665725	-3.036208	1.487592
C	-4.940794	-3.034830	2.337333
C	-2.553447	-3.763543	2.250020
C	-2.053843	-1.566697	0.106535
C	-2.334445	-2.263558	-1.216208
N	-1.655331	-0.177545	-0.103738
C	0.009778	0.228368	-0.755882
C	0.685737	1.021277	0.412806
C	1.464848	2.244325	0.035778
O	-0.110882	0.562895	-1.921602
C	-2.560448	0.738090	-0.117028
S	-2.222552	2.421894	-0.536366
C	-3.909699	2.837889	-0.232963
C	-4.657451	1.707153	0.113666
C	-4.507342	4.086150	-0.335160
C	-5.874707	4.188434	-0.082977
C	-6.620820	3.058838	0.255845
C	-6.023782	1.803783	0.356035
C	-2.988081	-1.624961	-2.273898
C	-3.256876	-2.308663	-3.457261
C	-2.867990	-3.637797	-3.604672
C	-2.201261	-4.278404	-2.562863
C	-1.934300	-3.592467	-1.381136
C	2.236265	2.290485	-1.132100
C	2.970678	3.429356	-1.445141
C	2.944998	4.538118	-0.599775
C	2.174891	4.504733	0.559711
C	1.437484	3.363955	0.871128
C	1.424054	-0.332055	0.652392
C	2.915506	-0.390181	0.409183
C	1.086039	-0.980406	1.992950
F	1.763262	-0.415633	3.001544
F	1.379063	-2.285934	1.972919
F	-0.219870	-0.875572	2.295265
O	0.682720	-0.995450	-0.373157
C	3.421018	-1.259677	-0.554782
C	4.790644	-1.324380	-0.799204
C	5.643498	-0.508491	-0.068111
Br	7.509024	-0.579119	-0.402081
C	5.160226	0.368177	0.898594
C	3.791820	0.422704	1.133412
H	-0.002700	1.245011	1.230301
H	-4.890768	-1.242956	-0.244463
H	-5.111435	-0.580207	1.390619
H	-3.875653	-3.581565	0.557679
H	-5.169745	-4.051344	2.670822
H	-5.810803	-2.668604	1.784295
H	-4.812158	-2.410664	3.230306
H	-2.860981	-4.786196	2.487784
H	-2.343343	-3.246181	3.193882
H	-1.615490	-3.821686	1.690718
H	-2.847174	-1.127431	2.031501
H	-1.190133	-2.056006	0.555849
H	-3.923158	4.959237	-0.606118
H	-6.359479	5.155798	-0.157406
H	-7.685433	3.152839	0.442095
H	-6.610364	0.927442	0.609280
H	-3.274472	-0.579606	-2.192209
H	-3.762868	-1.796005	-4.269459
H	-3.073230	-4.167166	-4.529880
H	-1.878638	-5.309274	-2.672130
H	-1.399985	-4.094058	-0.577907
H	2.254147	1.430905	-1.794492
H	3.566580	3.451391	-2.352400
H	3.521059	5.424612	-0.846897
H	2.145467	5.365109	1.221237
H	0.834397	3.339155	1.775840
H	2.736810	-1.877579	-1.125462
H	5.187655	-1.998636	-1.549641
H	5.842358	1.003337	1.452563
H	3.405451	1.119422	1.870855

Statistical Thermodynamic Analysis
Temperature= 298.150 Kelvin Pressure= 1.00000 Atm
==
==============================
SCF Energy= -4880.47409106 Predicted Change= -1.425042D-09
Zero-point correction (ZPE)= -4879.8895 0.58449
Internal Energy (U)= -4879.8521 0.62193
Enthalpy (H)= -4879.8512 0.62288
Gibbs Free Energy (G)= -4879.9629 0.51118

Frequencies -- -141.7196 14.6497 17.1040

183.15K thermal correction = 0.548971
Single point SCF = -4883.525698

TS-G: Catalyst release (Si,Si)

Using Gaussian 09: AM64L-G09RevD.01 24-Apr-2013
==
==============================
#m062x/6-31G(d) scf=(maxcycle=300,direct,tight)
density=current

opt=(maxcycle=250,ts,calcfc,noeigentest,gdiis) iop(1/8=18) freq=noraman
temp=298.15 SCRF=(PCM,SOLVENT=dichloromethane)
#N Geom=AllCheck Guess=TCheck SCRF=Check Test GenChk RM062X/6-31G(d) Freq
----
Pointgroup= C1 Stoichiometry= C35H30BrF3N2O2S
C1[X(C35H30BrF3N2O2S)] #Atoms= 74
Charge = 0 Multiplicity = 1

SCF Energy= -4880.46341812 Predicted Change= -1.303582D-10
==
Optimization completed. {Found 2 times}

Item	Max Val.	Criteria	Pass?	RMS Val.	Criteria	Pass?
Force	0.00000 ||	0.00045	[YES]	0.00000 ||	0.00030	[YES]
Displ	0.00049 ||	0.00180	[YES]	0.00049 ||	0.00180	[YES]

Atomic Coordinates (Angstroms)

Type	X	Y	Z
N	3.229465	1.319421	-0.311339
C	4.157955	0.201223	-0.465037
C	3.394595	-1.111601	-0.618334
C	4.342574	-2.322945	-0.571582
C	5.561945	-2.125102	-1.478784
C	3.620824	-3.608947	-0.982031
C	2.263435	-1.156006	0.437706
C	2.784309	-1.252052	1.866698
N	1.367102	-0.010221	0.236699
C	-0.274848	-0.285000	1.027091
C	-1.103686	-1.150458	0.017041
C	-0.571543	-2.188973	-0.936274
O	-0.069201	-0.519790	2.199454
C	1.910495	1.139065	-0.008641
S	1.068743	2.698489	0.071870
C	2.569205	3.541775	-0.292771
C	3.629502	2.647953	-0.458378
C	2.765239	4.910429	-0.418655
C	4.049194	5.372508	-0.703305
C	5.109062	4.477574	-0.857825
C	4.914703	3.103197	-0.737126
C	2.928922	-2.518857	2.438812
C	3.445774	-2.672693	3.722586
C	3.821136	-1.553450	4.461218
C	3.669378	-0.285179	3.906042
C	3.153569	-0.135374	2.620808
C	-0.888837	-3.525409	-0.657659
C	-0.431461	-4.563832	-1.464809
C	0.346777	-4.283265	-2.583808
C	0.670535	-2.959545	-2.874049
C	0.225266	-1.923607	-2.058206
C	-1.761845	0.207263	-0.356347
C	-1.544151	0.807602	-1.743161
F	-2.132151	2.008630	-1.826247
F	-2.069288	0.024539	-2.695849
F	-0.251821	0.999736	-2.043162
C	-3.246407	0.263721	-0.051413
O	-0.979899	0.921692	0.599695
C	-4.108851	-0.675497	-0.619610
C	-5.472530	-0.631863	-0.351382
C	-5.959680	0.361657	0.491549
Br	-7.816345	0.421178	0.873192
C	-5.116953	1.304917	1.066623
C	-3.754077	1.252467	0.787329
H	-1.821492	-1.659790	0.667025
H	4.761903	0.399036	-1.354182
H	4.824399	0.168891	0.407931
H	4.702689	-2.429798	0.459889
H	6.211769	-1.313672	-1.137640
H	5.249356	-1.905675	-2.507279
H	6.163715	-3.038658	-1.501109
H	4.272237	-4.474246	-0.824502
H	3.360396	-3.571807	-2.045884
H	2.692117	-3.782941	-0.429209
H	2.899278	-1.104881	-1.600582
H	1.654909	-2.044104	0.251409
H	1.938657	5.602390	-0.294845
H	4.223030	6.438630	-0.801875
H	6.103612	4.853373	-1.073915
H	5.742040	2.411039	-0.850591
H	2.624985	-3.397725	1.874734
H	3.546191	-3.666818	4.147447
H	4.220759	-1.667555	5.464022
H	3.944831	0.595832	4.477633
H	3.019718	0.867639	2.225902
H	-1.504856	-3.754942	0.207901
H	-0.692203	-5.589146	-1.221173
H	0.700134	-5.086728	-3.222729
H	1.281255	-2.726266	-3.741626
H	0.508788	-0.909296	-2.302587
H	-3.714981	-1.452767	-1.269666
H	-6.147320	-1.361864	-0.784255
H	-5.518431	2.066403	1.725721
H	-3.076266	1.972451	1.233096

Statistical Thermodynamic Analysis
Temperature= 298.150 Kelvin Pressure= 1.00000 Atm
==
SCF Energy= -4880.46341812 Predicted Change= -1.303582D-10
Zero-point correction (ZPE)= -4879.8789 0.58451
Internal Energy (U)= -4879.8417 0.62164
Enthalpy (H)= -4879.8408 0.62258
Gibbs Free Energy (G)= -4879.9502 0.51313

Frequencies -- -157.8353 16.9452 18.0468

183.15K thermal correction = 0.550059
Single point SCF = -4883.513988

H: Minor Product

Using Gaussian 09: AM64L-G09RevD.01 24-Apr-2013
==
#m062X/6-31G(d) scf=(direct,tight,maxcycle=300,xqc)
SCRF=(PCM,SOLVENT=CH2Cl2) opt=(maxcycle=250,gdiis) iop(1/8=18)
freq=noraman temp=298.15
#N Geom=AllCheck Guess=TCheck SCRF=Check Test GenChk RM062X/6-31G(d) Freq
----
Pointgroup= C1 Stoichiometry= C16H10BrF3O2
C1[X(C16H10BrF3O2)] #Atoms= 32
Charge = 0 Multiplicity = 1

SCF Energy= -3637.08627199 Predicted Change= -1.114704D-08
==
Optimization completed. {Found 1 times}

Item Max Val. Criteria Pass? RMS Val. Criteria Pass?
Force 0.00002 || 0.00045 [YES] 0.00000 || 0.00030 [YES]
Displ 0.00209 || 0.00180 [NO] 0.00209 || 0.00180 [YES]

Atomic Coordinates (Angstroms)

Type	X	Y	Z
C	-1.989676	-1.709437	-1.394624
C	-1.714521	-0.253184	-1.047770
C	-2.839855	0.669177	-0.668318
O	-2.737302	-2.332919	-2.077058
C	-4.108105	0.182060	-0.346761
C	-5.126473	1.062551	0.007953
C	-4.885570	2.433730	0.048328
C	-3.623894	2.925908	-0.279882
C	-2.608833	2.047180	-0.643889
H	-1.106234	0.188185	-1.845045
H	-4.304089	-0.885302	-0.382426
H	-6.110363	0.674386	0.251554
H	-5.680963	3.117763	0.326958
H	-3.431670	3.993891	-0.261673
H	-1.631572	2.434710	-0.921633
C	-0.742631	-0.903159	-0.011795
C	-1.267272	-0.916768	1.421306
F	-1.299075	0.322624	1.922345
F	-0.486660	-1.664214	2.205170
F	-2.503711	-1.424209	1.478553
C	0.708851	-0.516493	-0.062074
O	-1.006478	-2.210027	-0.577677
C	1.685041	-1.479111	-0.309489
C	3.029277	-1.119515	-0.340825
C	3.379132	0.206734	-0.118203
Br	5.207211	0.702386	-0.152098
C	2.417483	1.180181	0.133011
C	1.077754	0.811370	0.161375
H	1.396694	-2.510180	-0.484107
H	3.794675	-1.862882	-0.532865
H	2.711126	2.209637	0.303797
H	0.320998	1.564682	0.362097

Statistical Thermodynamic Analysis
Temperature= 298.150 Kelvin Pressure= 1.00000 Atm

===

SCF Energy= -3637.08627199 Predicted Change= -1.114704D-08
Zero-point correction (ZPE)= -3636.8576 0.22859
Internal Energy (U)= -3636.8396 0.24661
Enthalpy (H)= -3636.8387 0.24755
Gibbs Free Energy (G)= -3636.9065 0.17971

Frequencies -- 28.4101 34.3366 46.9163

183.15K thermal correction = 0.203346
Single point SCF = -3639.842206

Alternative Mechanisms: PBE

Alt 1: [2+2] β-lactone formation TS

Using Gaussian 09: AM64L-G09RevD.01 24-Apr-2013

===

# pbepbe/6-31G(d)/auto gfprint gfinput scf=(direct,tight,maxcycle=300,xqc)
opt=(maxcycle=250,calcfc,ts,noeigentest) freq=noraman
SCRF=(PCM,SOLVENT=Dichloromethane) iop(1/8=18)
Temperature=298.15
#N Geom=AllCheck Guess=TCheck SCRF=Check Test GenChk RPBEPBE/6-31G(d)/Auto
Freq

Pointgroup= C1 Stoichiometry= C16H10BrF3O2
C1[X(C16H10BrF3O2)] #Atoms= 32
Charge = 0 Multiplicity = 1

SCF Energy= -3635.76594985 Predicted Change= -4.566671D-10

===

Optimization completed. {Found 2 times}
Item Max Val. Criteria Pass? RMS Val. Criteria Pass?
Force 0.00000 || 0.00045 [YES] 0.00000 || 0.00030 [YES]
Displ 0.00030 || 0.00180 [YES] 0.00030 || 0.00180 [YES]

Atomic Coordinates (Angstroms)

Type	X	Y	Z
C	-2.384868	-0.261640	0.424416
H	-1.401664	-0.650680	0.160364
C	-3.540922	-1.072270	0.089831
C	-2.468309	0.866985	1.231495
O	-3.252201	1.569089	1.816413
C	-4.878858	-0.709187	0.405911
H	-5.065320	0.216310	0.958446
C	-5.947892	-1.524177	0.024790
H	-6.969859	-1.224646	0.280967
C	-5.723641	-2.719786	-0.679997
H	-6.565991	-3.354009	-0.974384
C	-4.407460	-3.095552	-0.998536
H	-4.218536	-4.025234	-1.545830
C	-3.332623	-2.290322	-0.614572
H	-2.308458	-2.589537	-0.865870
C	-0.155210	1.420681	0.402158
C	-0.689022	2.346445	-0.708397
F	-1.867952	2.912275	-0.387175
F	-0.826981	1.694034	-1.892185
F	0.206311	3.359465	-0.908025
C	1.147871	0.802367	0.261292
O	-0.814572	1.283918	1.503819
C	1.595350	-0.054418	1.306000
H	0.950220	-0.212112	2.173944
C	2.832355	-0.687401	1.231317
H	3.172013	-1.343585	2.036428
C	3.639487	-0.475193	0.099955
Br	5.326503	-1.339070	-0.013738
C	3.222863	0.358296	-0.952705
H	3.864971	0.506008	-1.824393
C	1.986083	0.993372	-0.872767
H	1.675585	1.639719	-1.695392

Statistical Thermodynamic Analysis
Temperature= 298.150 Kelvin Pressure= 1.00000 Atm

===

SCF Energy= -3635.76594985 Predicted Change= -4.566671D-10
Zero-point correction (ZPE)= -3635.5510 0.21490
Internal Energy (U)= -3635.5315 0.23438

Enthalpy (H)= -3635.5306
0.23532
Gibbs Free Energy (G)= -3635.6025
0.16337

Frequencies -- -199.1147 14.1523 29.9279

183.15K thermal correction = 0.188402
Single point SCF = -3638.534496

Alt 2: *N,N*-diisopropylethylamine catalyst/ketone TS

Using Gaussian 09: AM64L-G09RevD.01 24-Apr-2013
===
==============================
# pbepbe/6-31G(d)/auto scf=(maxcycle=300,direct,tight,xqc)
density=current
opt=(maxcycle=250,modredundant)
SCRF=(PCM,SOLVENT=Dichloromethane)
iop(1/8=18) Temperature=298.15
Modredundant Input: B 1 44 F
Modredundant Input:
# pbepbe/6-31G(d)/auto scf=(direct,tight,maxcycle=300,xqc)
opt=(nofreeze,maxcycle=250,ts,calcfc,noeigentest) iop(1/8=18)
freq=noraman
SCRF=(PCM,SOLVENT=Dichloromethane)
Temperature=298.15 geom=check
guess=read
#N Geom=AllCheck Guess=TCheck SCRF=Check Test GenChk
RPBEPBE/6-31G(d)/Auto
Freq

Pointgroup= C1 Stoichiometry= C24H29BrF3NO2
C1[X(C24H29BrF3NO2)] #Atoms= 60
Charge = 0 Multiplicity = 1

SCF Energy= -4006.28947729 Predicted Change= -6.498281D-08
===
==============================
Optimization completed. {Found 2 times}

Item	Max Val.	Criteria	Pass?	RMS Val.	Criteria	Pass?
Force	0.00002 ||	0.00045	[YES]	0.00000 ||	0.00030	[YES]
Displ	0.02980 ||	0.00180	[NO]	0.02980 ||	0.00180	[NO]

Atomic Type	Coordinates (Angstroms) X	Y	Z
C	-1.072865	0.772753	0.280972
H	-1.907301	1.109742	0.904551
C	-0.586919	1.944216	-0.547794
C	-1.457606	-0.404478	-0.547250
N	-3.028652	-0.997977	-0.440769
C	-3.548264	-0.758792	0.971807
H	-2.733825	-1.092687	1.641147
C	-4.912268	-1.348977	1.323111
H	-5.177467	-0.964283	2.322521
H	-4.911158	-2.446568	1.387821
H	-5.712884	-1.034688	0.632639
H	-3.609832	0.330205	1.088580
C	-2.917361	-2.512557	-0.802009
H	-2.178161	-2.507006	-1.615115
C	-4.202498	-3.157771	-1.340702
H	-3.963959	-4.220785	-1.514894
H	-4.532120	-2.745579	-2.306935
H	-5.044828	-3.124953	-0.634729
C	-2.313554	-3.315458	0.356790
H	-1.469819	-2.782458	0.827600
H	-1.951415	-4.272499	-0.054718
H	-3.057349	-3.551978	1.135777
C	-3.880409	-0.241261	-1.505552
C	-4.181167	1.219141	-1.142542
H	-4.792169	1.330335	-0.234084
H	-4.769358	1.640516	-1.974580
H	-3.269656	1.831355	-1.046716
H	-4.833224	-0.793001	-1.503737
C	-3.265128	-0.312963	-2.910756
H	-4.023099	0.051471	-3.623247
H	-2.982627	-1.329989	-3.219394
H	-2.379990	0.337223	-3.002923
O	-0.763542	-0.976888	-1.347584
C	0.240376	1.815237	-1.685735
H	0.550947	0.821947	-2.019192
C	0.666689	2.948563	-2.393021
H	1.305946	2.822138	-3.273393
C	0.284362	4.233517	-1.980527
H	0.619894	5.115677	-2.535593
C	-0.536413	4.376486	-0.850979
H	-0.846455	5.372457	-0.517152
C	-0.970473	3.245660	-0.147922
H	-1.606340	3.369017	0.735923
C	-0.065978	0.013698	1.480334
C	1.351310	-0.201674	0.895618
C	-0.022391	1.054229	2.653092
F	0.447316	2.305529	2.329212
F	0.764720	0.592373	3.661571
F	-1.269609	1.248647	3.176114
O	-0.670840	-1.083400	1.895489
C	2.265379	0.819120	0.559579
H	2.000772	1.868936	0.697682
C	3.539021	0.513172	0.057088
H	4.242462	1.311582	-0.195317
C	3.897696	-0.828503	-0.116101
Br	5.638620	-1.253795	-0.807943
C	3.017945	-1.866019	0.214234
H	3.318441	-2.909709	0.084871
C	1.755147	-1.538312	0.723040
H	1.048868	-2.318560	1.022025

Statistical Thermodynamic Analysis
Temperature= 298.150 Kelvin Pressure= 1.00000 Atm
===
==============================
SCF Energy= -4006.28947729 Predicted Change= -6.498281D-08
Zero-point correction (ZPE)= -4005.8122 0.47720
Internal Energy (U)= -4005.7807 0.50877
Enthalpy (H)= -4005.7797
0.50971
Gibbs Free Energy (G)= -4005.8765
0.41294

Frequencies -- -156.9751 2.1905 25.5969

183.15K thermal correction = 0.445777
Single point SCF = -4009.157805

Alt 3: HBTM-ketone/ketene TS

Using Gaussian 09: AM64L-G09RevD.01 24-Apr-2013
===
==============================
# pbepbe/6-31G(d)/auto scf=(maxcycle=300,direct,tight,xqc)
density=current

opt=(maxcycle=250,modredundant)
SCRF=(PCM,SOLVENT=Dichloromethane)
iop(1/8=18) Temperature=298.15
Modredundant Input: B 24 62 F
Modredundant Input:
# pbepbe/6-31G(d)/auto scf=(direct,tight,maxcycle=300,xqc)
opt=(nofreeze,maxcycle=250,ts,calcfc,noeigentest) iop(1/8=18)
freq=noraman
SCRF=(PCM,SOLVENT=Dichloromethane)
Temperature=298.15 geom=check
guess=read
#N Geom=AllCheck Guess=TCheck SCRF=Check Test GenChk
RPBEPBE/6-31G(d)/Auto
Freq

Pointgroup= C1 Stoichiometry= C35H30BrF3N2O2S
C1[X(C35H30BrF3N2O2S)] #Atoms= 74
Charge = 0 Multiplicity = 1

SCF Energy= -4878.29366039 Predicted Change= -2.013965D-09
==
Optimization completed on the basis of negligible forces.
{Found 3 times}

Item	Max Val.	Criteria	Pass?	RMS Val.	Criteria	Pass?
Force	0.00000 ||	0.00045	[YES]	0.00000 ||	0.00030	[YES]
Displ	0.00430 ||	0.00180	[NO]	0.00430 ||	0.00180	[YES]

Atomic Type	Coordinates (Angstroms) X	Y	Z
C	6.451511	-2.995958	-0.895203
C	5.909078	-1.796483	-0.414833
C	4.511000	-1.694037	-0.340140
C	3.682581	-2.762489	-0.722677
C	4.230022	-3.964046	-1.190038
C	5.624687	-4.067562	-1.281460
N	3.760578	-0.594251	0.113102
C	2.409874	-0.768014	0.103546
S	1.970226	-2.386707	-0.461975
C	4.399027	0.644893	0.579074
C	3.493561	1.841031	0.292705
C	3.591618	2.370186	-1.169725
C	4.979341	2.993062	-1.422835
C	3.250147	1.360530	-2.280853
C	2.085248	1.526458	0.898924
C	1.165326	2.735651	0.768578
N	1.526607	0.205430	0.418009
C	0.076381	-0.323222	0.911392
C	-1.028857	0.598873	0.382732
C	0.195641	-0.369675	2.483295
F	0.329355	0.845380	3.101936
F	1.270285	-1.115425	2.846034
F	-0.911259	-0.949538	3.016770
O	-0.069645	-1.590466	0.508428
C	0.581055	3.154811	-0.443196
C	-0.149668	4.348672	-0.509787
C	-0.292241	5.157449	0.628148
C	0.295108	4.758309	1.836646
C	1.013708	3.556232	1.903549
C	-1.932064	1.296975	1.202331
C	-2.995665	2.021478	0.643937
C	-3.155144	2.038308	-0.744469
Br	-4.594652	3.040075	-1.514811
C	-2.280021	1.338105	-1.585472
C	-1.225463	0.621528	-1.013063
H	7.538715	-3.096974	-0.962256
H	6.565402	-0.981607	-0.099486
H	3.583455	-4.797735	-1.480384
H	6.072933	-4.993342	-1.654170
H	5.357113	0.745140	0.049201
H	4.617241	0.544400	1.656731
H	4.991740	3.496530	-2.404656
H	5.785230	2.236416	-1.440309
H	5.234210	3.744785	-0.655233
H	2.865618	3.199729	-1.236421
H	3.265851	1.870045	-3.259842
H	2.248798	0.914422	-2.156258
H	3.985670	0.537905	-2.334153
H	3.860613	2.664268	0.933118
H	2.272094	1.395704	1.979692
H	0.668502	2.531902	-1.336886
H	-0.611813	4.645407	-1.456929
H	-0.861755	6.090920	0.572507
H	0.189511	5.377585	2.733404
H	1.462014	3.248685	2.854760
H	-1.824765	1.297322	2.288487
H	-3.690264	2.566492	1.288297
H	-2.421441	1.351297	-2.669398
H	-0.551569	0.055939	-1.662135
C	-2.464479	-2.658644	0.487578
C	-3.732711	-3.343133	0.245233
C	-1.585769	-2.399791	-0.512807
O	-1.242148	-2.475210	-1.661925
C	-4.151059	-3.808412	-1.029706
C	-5.378154	-4.460173	-1.194102
C	-6.234175	-4.673578	-0.100160
C	-5.836580	-4.220231	1.168262
C	-4.610521	-3.567514	1.338899
H	-2.214170	-2.336883	1.496028
H	-3.499874	-3.653172	-1.896696
H	-5.670118	-4.806417	-2.192407
H	-7.193496	-5.184069	-0.234509
H	-6.487845	-4.375378	2.036226
H	-4.313800	-3.218752	2.335392

Statistical Thermodynamic Analysis
Temperature= 298.150 Kelvin Pressure= 1.00000 Atm
==
SCF Energy= -4878.29366039 Predicted Change= -2.013965D-09
Zero-point correction (ZPE)= -4877.7345 0.55915
Internal Energy (U)= -4877.6946 0.59897
Enthalpy (H)= -4877.6937 0.59992
Gibbs Free Energy (G)= -4877.8106 0.48304

Frequencies -- -61.6958 8.5901 11.5453

183.15K thermal correction = 0.522477
Single point SCF = -4881.322305

Stepwise Mechanism: B3LYP

SM: Phenylacetic anhydride

Using Gaussian 09: AM64L-G09RevD.01 24-Apr-2013
==
# B3LYP/6-31G* gfprint gfinput
scf=(direct,tight,maxcycle=300,xqc)
opt=(maxcycle=250) freq=noraman iop(1/8=14)
Temperature=298.15
SCRF=(PCM,SOLVENT=Dichloromethane)

#N Geom=AllCheck Guess=TCheck SCRF=Check Test GenChk RB3LYP/6-31G(d) Freq
----
Pointgroup= C1 Stoichiometry= C16H14O3
C1[X(C16H14O3)] #Atoms= 33
Charge = 0 Multiplicity = 1

SCF Energy= -843.835296614 Predicted Change= -4.931914D-09
==
Optimization completed. {Found 1 times}
Item Max Val. Criteria Pass? RMS Val. Criteria Pass?
Force 0.00000 || 0.00045 [YES] 0.00000 || 0.00030 [YES]
Displ 0.01005 || 0.00180 [NO] 0.01005 || 0.00180 [NO]

Atomic Coordinates (Angstroms)

Type	X	Y	Z
C	1.218493	0.040662	0.035879
C	2.310296	0.984756	-0.434421
C	3.708843	0.450443	-0.231246
O	-0.000361	0.715010	0.021163
C	-1.215211	0.036801	-0.053720
C	-2.306898	0.918933	0.524137
C	-3.707259	0.409364	0.275391
O	-1.334717	-1.056358	-0.534953
O	1.342462	-1.095667	0.402294
C	4.263461	-0.462348	-1.137923
C	5.552652	-0.960871	-0.946690
C	6.306154	-0.551224	0.156371
C	5.762540	0.359101	1.064839
C	4.471789	0.855750	0.870574
C	-4.525788	1.019832	-0.682063
C	-5.821490	0.552386	-0.914317
C	-6.312857	-0.534984	-0.189815
C	-5.503045	-1.150650	0.768524
C	-4.210126	-0.680159	0.999255
H	2.166481	1.941686	0.079284
H	2.108966	1.183969	-1.495887
H	-2.169027	1.927929	0.120427
H	-2.096731	0.999201	1.599844
H	3.683369	-0.783263	-1.999885
H	5.969418	-1.666578	-1.660217
H	7.311235	-0.936653	0.304457
H	6.342575	0.685849	1.923713
H	4.054915	1.566511	1.580495
H	-4.148566	1.868395	-1.248142
H	-6.445225	1.039292	-1.659106
H	-7.320998	-0.899269	-0.367757
H	-5.879297	-1.995906	1.338429
H	-3.585509	-1.161791	1.747659

Statistical Thermodynamic Analysis
Temperature= 298.150 Kelvin Pressure= 1.00000 Atm
==
SCF Energy= -843.835296614 Predicted Change= -4.931914D-09
Zero-point correction (ZPE)= -843.5733 0.26190
Internal Energy (U)= -843.5566 0.27865
Enthalpy (H)= -843.5557 0.27959
Gibbs Free Energy (G)= -843.6244 0.21087

Frequencies -- 6.7021 14.5331 21.1244

183.15K thermal correction = 0.235085
Single point SCF = -844.113666

TS-I-(Re,Si): C–C bond formation

Using Gaussian 09: AM64L-G09RevD.01 24-Apr-2013
==
# B3LYP/6-31G(d) scf=(maxcycle=300,direct,tight,xqc) density=current
opt=(maxcycle=250,modredundant)
SCRF=(PCM,SOLVENT=CH2Cl2) iop(1/8=18)
Temperature=298.15
Modredundant Input: B 9 61 F
Modredundant Input:
# B3LYP/6-31G(d) scf=(direct,tight,maxcycle=300,xqc)
opt=(nofreeze,maxcycle=250,ts,calcfc,noeigentest) iop(1/8=18) freq=noraman
SCRF=(PCM,SOLVENT=CH2Cl2) Temperature=298.15 geom=check guess=read
#N Geom=AllCheck Guess=TCheck SCRF=Check Test GenChk RB3LYP/6-31G(d) Freq
----
Pointgroup= C1 Stoichiometry= C35H30BrF3N2O2S
C1[X(C35H30BrF3N2O2S)] #Atoms= 74
Charge = 0 Multiplicity = 1

SCF Energy= -4881.15710620 Predicted Change= -1.297977D-08
==
Optimization completed. {Found 3 times}
Item Max Val. Criteria Pass? RMS Val. Criteria Pass?
Force 0.00002 || 0.00045 [YES] 0.00000 || 0.00030 [YES]
Displ 0.00156 || 0.00180 [YES] 0.00156 || 0.00180 [YES]

Atomic Coordinates (Angstroms)

Type	X	Y	Z
C	-2.191463	-3.462813	3.206702
C	-1.407878	-2.991189	2.155666
C	-1.470235	-1.648448	1.733382
C	-2.359116	-0.793730	2.412126
C	-3.139721	-1.265496	3.470048
C	-3.064328	-2.599253	3.874214
H	-2.115230	-4.504592	3.508305
H	-0.729239	-3.673581	1.649583
C	-0.589563	-1.227383	0.596447
H	-2.430111	0.245144	2.119400
H	-3.814288	-0.580829	3.978592
H	-3.674803	-2.961380	4.697277
C	0.103724	0.052312	0.766168
N	1.422044	0.205338	0.191980
C	2.080515	1.368416	0.384893
N	3.313830	1.591323	-0.108067
C	4.003146	0.661362	-1.021523
C	2.979772	-0.259367	-1.676541
C	2.081441	-0.895910	-0.584663
H	4.757635	0.103973	-0.455593
H	4.510241	1.276671	-1.764594
C	3.629101	-1.304936	-2.622768
H	2.307796	0.365036	-2.280170
C	2.762697	-1.898589	0.338993
H	1.258524	-1.357446	-1.125123
O	-0.360371	1.039498	1.332607

H	0.104386	-2.030434	0.371679
C	3.511581	-1.515752	1.461751
C	4.140007	-2.472343	2.261487
C	4.028335	-3.829516	1.954984
C	3.274972	-4.224634	0.848131
C	2.644346	-3.267522	0.051624
H	3.598017	-0.469313	1.739199
H	4.712041	-2.153309	3.128199
H	4.515332	-4.572974	2.579556
H	3.167359	-5.278571	0.607630
H	2.040378	-3.587721	-0.792441
S	1.471884	2.723059	1.316424
C	2.993495	3.583022	1.043907
C	3.871304	2.827711	0.261113
C	5.140178	3.307665	-0.072725
C	5.497692	4.571253	0.393189
C	4.616301	5.335878	1.171964
C	3.353897	4.848858	1.506400
H	5.833023	2.720723	-0.664637
H	6.478802	4.965727	0.148548
H	4.920106	6.317429	1.521815
H	2.670515	5.436263	2.111268
C	2.572822	-1.981068	-3.515054
H	4.105932	-2.073665	-2.001038
C	4.721476	-0.685527	-3.514159
H	2.140237	-1.254337	-4.213682
H	1.743870	-2.418677	-2.953128
H	3.037706	-2.777974	-4.105890
H	5.584979	-0.331078	-2.941178
H	4.329027	0.155918	-4.099330
H	5.090132	-1.435920	-4.221714
C	-2.555508	1.161236	-1.339775
H	-1.620935	1.519607	-1.756624
C	-2.627457	-0.184963	-0.954872
C	-1.354061	-1.012335	-1.159253
C	-3.844138	-0.665829	-0.447609
H	-3.945506	-1.699757	-0.144637
C	-4.956587	0.171072	-0.332760
H	-5.894888	-0.214679	0.051530
C	-4.844926	1.504716	-0.714199
Br	-6.370294	2.661945	-0.553769
C	-3.650034	2.014701	-1.217767
H	-3.578783	3.054601	-1.519055
C	-1.632897	-2.497251	-1.535663
F	-2.386150	-3.216695	-0.663386
F	-0.470487	-3.189495	-1.677688
F	-2.267129	-2.557973	-2.729611
O	-0.469962	-0.510387	-1.946231

Statistical Thermodynamic Analysis
Temperature= 298.150 Kelvin Pressure= 1.00000 Atm
==
SCF Energy= -4881.15710620 Predicted Change= -1.297977D-08
Zero-point correction (ZPE)= -4880.5796 0.57746
Internal Energy (U)= -4880.5416 0.61549
Enthalpy (H)= -4880.5406 0.61643
Gibbs Free Energy (G)= -4880.6521 0.50496

Frequencies -- -184.7330 11.9192 21.0829

183.15K thermal correction = 0.542559
Single point SCF = -4884.241838

TS-I-(Re,Re): C–C bond formation

Using Gaussian 09: AM64L-G09RevD.01 24-Apr-2013
==
# B3LYP/6-31G(d) scf=(maxcycle=300,direct,tight,xqc) density=current
opt=(maxcycle=250,modredundant)
SCRF=(PCM,SOLVENT=CH2Cl2) iop(1/8=18)
Temperature=298.15
Modredundant Input: B 11 58 F
Modredundant Input:
# B3LYP/6-31G(d) scf=(direct,tight,maxcycle=300,xqc)
opt=(nofreeze,maxcycle=250,ts,calcfc,noeigentest) iop(1/8=18) freq=noraman
SCRF=(PCM,SOLVENT=CH2Cl2) Temperature=298.15 geom=check guess=read
#N Geom=AllCheck Guess=TCheck SCRF=Check Test GenChk RB3LYP/6-31G(d) Freq
----
Pointgroup= C1 Stoichiometry= C35H30BrF3N2O2S
C1[X(C35H30BrF3N2O2S)] #Atoms= 74
Charge = 0 Multiplicity = 1

SCF Energy= -4881.15530347 Predicted Change= -5.351702D-09
==
Optimization completed. {Found 3 times}

Item	Max Val.	Criteria	Pass?	RMS Val.	Criteria	Pass?
Force	0.00001 ||	0.00045	[YES]	0.00000 ||	0.00030	[YES]
Displ	0.00114 ||	0.00180	[YES]	0.00114 ||	0.00180	[YES]

Atomic Coordinates (Angstroms)

Type	X	Y	Z
N	-4.154099	0.109123	-0.240554
C	-4.054866	1.547288	-0.543745
C	-2.652747	1.865954	-1.052320
C	-2.454892	3.376292	-1.354601
C	-3.661147	3.992806	-2.085900
C	-1.183539	3.613186	-2.188466
C	-1.601592	1.292057	-0.065979
C	-1.535810	1.965185	1.299998
N	-1.829596	-0.185696	0.068338
C	-0.742699	-1.107270	0.336809
C	0.624235	-0.579771	0.455008
H	0.627176	0.471310	0.725032
C	1.510515	-1.328268	1.408155
O	-1.053636	-2.292702	0.434261
C	-3.083974	-0.677067	-0.013014
S	-3.519455	-2.365091	0.183667
C	-5.224386	-1.940915	-0.024081
C	-5.385512	-0.568569	-0.234392
C	-6.326589	-2.795043	0.008890
C	-7.593532	-2.241287	-0.167943
C	-7.751875	-0.862347	-0.370642
C	-6.653017	-0.006068	-0.405789
C	-0.593418	2.986404	1.499684
C	-0.505150	3.652834	2.722597
C	-1.353321	3.301628	3.774544
C	-2.284724	2.277884	3.594103
C	-2.373891	1.614405	2.368435
C	2.287754	-0.575126	2.308064
C	3.152566	-1.186997	3.214698
C	3.260684	-2.578809	3.250237
C	2.495819	-3.341718	2.365205
C	1.634680	-2.729165	1.453336
H	-4.304998	2.116963	0.358354

H	-4.805091	1.756381	-1.306101
H	-2.347761	3.899839	-0.396038
H	-4.569509	4.002915	-1.474050
H	-3.880259	3.456392	-3.018125
H	-3.438790	5.032423	-2.349665
H	-0.995355	4.688146	-2.286889
H	-1.308677	3.202739	-3.198397
H	-0.292846	3.144855	-1.762411
H	-2.508858	1.317399	-1.993549
H	-0.637990	1.365645	-0.578745
H	-6.199944	-3.860774	0.170081
H	-8.466576	-2.885800	-0.144928
H	-8.747060	-0.448899	-0.499919
H	-6.787850	1.059567	-0.551643
H	0.081916	3.256596	0.692517
H	0.233250	4.438784	2.854179
H	-1.282595	3.814543	4.729492
H	-2.941516	1.987260	4.409201
H	-3.092399	0.806479	2.265442
H	2.209877	0.509456	2.295746
H	3.737178	-0.574692	3.896774
H	3.930820	-3.061451	3.956754
H	2.569979	-4.426537	2.379825
H	1.054513	-3.337711	0.774039
C	1.330095	-0.228028	-1.300338
C	0.948895	-1.475545	-2.143586
F	-0.376405	-1.494368	-2.408456
F	1.245162	-2.683566	-1.591208
F	1.584565	-1.439293	-3.340895
C	2.840387	-0.116401	-1.044159
O	0.759044	0.829677	-1.758909
C	3.730692	-1.195842	-0.948676
C	5.094298	-0.986741	-0.735109
C	5.569571	0.315030	-0.604702
Br	7.444067	0.608691	-0.304599
C	4.711545	1.408529	-0.693813
C	3.355213	1.180217	-0.920433
H	3.375656	-2.214253	-1.040277
H	5.774075	-1.829414	-0.669547
H	5.095258	2.418804	-0.598302
H	2.668383	2.013253	-1.021279

Statistical Thermodynamic Analysis
Temperature= 298.150 Kelvin Pressure= 1.00000 Atm
==
==============================
SCF Energy= -4881.15530347 Predicted Change= -5.351702D-09
Zero-point correction (ZPE)= -4880.5782 0.57709
Internal Energy (U)= -4880.5400 0.61524
Enthalpy (H)= -4880.5391 0.61618
Gibbs Free Energy (G)= -4880.6516 0.50367

Frequencies -- -159.3080 9.7840 15.8161

183.15K thermal correction = 0.541664
Single point SCF = -4884.241314

TS-I-(Si,Re): C–C bond formation

Using Gaussian 09: AM64L-G09RevD.01 24-Apr-2013
==
==============================
# B3LYP/6-31G(d) scf=(maxcycle=300,direct,tight,xqc) density=current
opt=(maxcycle=250,modredundant)
SCRF=(PCM,SOLVENT=CH2Cl2) iop(1/8=18)
Temperature=298.15
Modredundant Input: B 11 58 F
Modredundant Input:
# B3LYP/6-31G(d) scf=(direct,tight,maxcycle=300,xqc)
opt=(nofreeze,maxcycle=250,ts,calcfc,noeigentest) iop(1/8=18) freq=noraman
SCRF=(PCM,SOLVENT=CH2Cl2) Temperature=298.15 geom=check guess=read
#N Geom=AllCheck Guess=TCheck SCRF=Check Test GenChk RB3LYP/6-31G(d) Freq

----

Pointgroup= C1 Stoichiometry= C35H30BrF3N2O2S
C1[X(C35H30BrF3N2O2S)] #Atoms= 74
Charge = 0 Multiplicity = 1

SCF Energy= -4881.14729205 Predicted Change= -1.013583D-09
==
==============================
Optimization completed. {Found 3 times}

Item	Max Val.	Criteria	Pass?	RMS Val.	Criteria	Pass?
Force	0.00000 ||	0.00045	[YES]	0.00000 ||	0.00030	[YES]
Displ	0.00124 ||	0.00180	[YES]	0.00124 ||	0.00180	[YES]

Atomic Type	Coordinates (Angstroms) X	Y	Z
N	4.153122	0.006380	0.149049
C	4.230602	1.447668	0.447673
C	3.035866	1.855502	1.306846
C	3.019446	3.374539	1.623821
C	4.394800	3.876683	2.100828
C	1.961315	3.710514	2.689038
C	1.732003	1.337675	0.632985
C	1.388550	2.010956	-0.691107
N	1.825246	-0.152938	0.524562
C	0.692515	-1.025875	0.859119
C	-0.668624	-0.494279	0.736245
H	-0.691308	0.537269	0.394246
C	-1.493517	-0.665051	1.981363
O	0.968677	-2.171993	1.182830
C	3.028778	-0.718164	0.306704
S	3.305574	-2.438370	0.130337
C	4.987833	-2.113721	-0.307257
C	5.279316	-0.748242	-0.223590
C	5.974013	-3.036280	-0.654634
C	7.253898	-2.556908	-0.930863
C	7.540482	-1.186278	-0.853324
C	6.559298	-0.262231	-0.498227
C	0.467668	3.068734	-0.689515
C	0.157272	3.750877	-1.867053
C	0.761847	3.380933	-3.069405
C	1.668791	2.320236	-3.085373
C	1.977150	1.638462	-1.907647
C	-1.633652	-1.906458	2.625031
C	-2.411685	-2.021444	3.778878
C	-3.067322	-0.908806	4.311116
C	-2.942296	0.327739	3.673284
C	-2.163680	0.445534	2.521668
H	4.270151	2.007256	-0.492887
H	5.166702	1.602417	0.984628
H	2.767604	3.912755	0.701273
H	4.745850	3.309821	2.972515
H	4.321423	4.928155	2.397798
H	5.160783	3.815577	1.320899
H	0.945303	3.437868	2.388106
H	1.961121	4.786860	2.890763

H	2.180019	3.193889	3.631848
H	3.120494	1.320575	2.263181
H	0.916877	1.510724	1.334310
H	5.749591	-4.096294	-0.713260
H	8.036029	-3.255324	-1.211046
H	8.542351	-0.833111	-1.075837
H	6.786755	0.796618	-0.449880
H	-0.016688	3.362324	0.238159
H	-0.560341	4.566040	-1.842575
H	0.520026	3.907478	-3.988182
H	2.131899	2.012038	-4.018389
H	2.653705	0.791429	-1.958396
H	-1.140863	-2.771349	2.202019
H	-2.508074	-2.990420	4.263111
H	-3.669829	-1.003821	5.210785
H	-3.448936	1.203065	4.072289
H	-2.074781	1.412764	2.031938
C	-1.497974	-1.519089	-0.592088
C	-2.933387	-0.930820	-0.624809
C	-0.725328	-1.134438	-1.888402
F	-0.685200	0.195130	-2.178728
F	-1.287794	-1.738298	-2.961887
F	0.567616	-1.548946	-1.846669
O	-1.402331	-2.770467	-0.351988
C	-3.266398	0.404178	-0.897918
C	-4.597213	0.827592	-0.908580
C	-5.603728	-0.096293	-0.639410
Br	-7.437563	0.479492	-0.651257
C	-5.306712	-1.428437	-0.364080
C	-3.971506	-1.831100	-0.361995
H	-2.496074	1.135275	-1.113342
H	-4.843709	1.862361	-1.122610
H	-6.101214	-2.138574	-0.158901
H	-3.700832	-2.860765	-0.156365

Statistical Thermodynamic Analysis
Temperature= 298.150 Kelvin Pressure= 1.00000 Atm
==
==============================
SCF Energy= -4881.14729205 Predicted Change= -1.013583D-09
Zero-point correction (ZPE)= -4880.5706 0.57668
Internal Energy (U)= -4880.5325 0.61478
Enthalpy (H)= -4880.5315 0.61572
Gibbs Free Energy (G)= -4880.6432 0.50402

Frequencies -- -61.6962 13.5944 15.6215

183.15K thermal correction = 0.541698
Single point SCF = -4884.234891

TS-I-(Si,Si): C–C bond formation

Using Gaussian 09: AM64L-G09RevD.01 24-Apr-2013
==
==============================
# B3LYP/6-31G(d) scf=(maxcycle=300,direct,tight,xqc) density=current
opt=(maxcycle=250,modredundant)
SCRF=(PCM,SOLVENT=CH2Cl2) iop(1/8=18)
Temperature=298.15
Modredundant Input: B 11 58 F
Modredundant Input:
# B3LYP/6-31G(d) scf=(direct,tight,maxcycle=300,xqc)
opt=(nofreeze,maxcycle=250,ts,calcfc,noeigentest) iop(1/8=18) freq=noraman
SCRF=(PCM,SOLVENT=CH2Cl2) Temperature=298.15
geom=check guess=read
#N Geom=AllCheck Guess=TCheck SCRF=Check Test GenChk RB3LYP/6-31G(d) Freq
----
Pointgroup= C1 Stoichiometry= C35H30BrF3N2O2S
C1[X(C35H30BrF3N2O2S)] #Atoms= 74
Charge = 0 Multiplicity = 1

SCF Energy= -4881.14688418 Predicted Change= -1.288239D-09
==
==============================
Optimization completed. {Found 3 times}
Item Max Val. Criteria Pass? RMS Val. Criteria Pass?
Force 0.00000 || 0.00045 [YES] 0.00000 || 0.00030 [YES]
Displ 0.00111 || 0.00180 [YES] 0.00111 || 0.00180 [YES]

Atomic Coordinates (Angstroms)

Type	X	Y	Z
N	-2.382223	1.800085	0.086698
C	-3.272352	1.447684	1.207431
C	-3.072553	-0.006933	1.623726
C	-4.148435	-0.475991	2.635314
C	-4.311734	0.521482	3.796757
C	-3.814688	-1.867749	3.200365
C	-2.961488	-0.904477	0.363386
C	-4.214469	-1.074943	-0.488966
N	-1.784362	-0.422794	-0.430620
C	-0.808134	-1.453851	-0.773004
C	0.613262	-1.069617	-0.824029
H	0.761418	-0.067059	-1.205811
C	1.529038	-2.002741	-1.558755
O	-1.247782	-2.591616	-0.859813
C	-1.632513	0.906060	-0.582943
S	-0.725354	1.674597	-1.883573
C	-1.357066	3.251633	-1.421661
C	-2.246520	3.129383	-0.348043
C	-1.065672	4.491835	-1.984807
C	-1.703728	5.614428	-1.456690
C	-2.602250	5.493051	-0.388260
C	-2.885238	4.252058	0.181529
C	-4.716271	-0.084910	-1.348299
C	-5.881440	-0.301235	-2.086514
C	-6.570490	-1.510421	-1.980101
C	-6.077392	-2.508008	-1.137894
C	-4.908012	-2.291919	-0.407828
C	2.503145	-1.438946	-2.403962
C	3.397339	-2.233539	-3.120803
C	3.340917	-3.624072	-3.009813
C	2.381937	-4.201355	-2.173930
C	1.487874	-3.405954	-1.456187
H	-4.302628	1.647198	0.889905
H	-3.027789	2.121460	2.030423
H	-5.109922	-0.538978	2.107002
H	-3.359825	0.679362	4.319452
H	-5.030281	0.131640	4.525791
H	-4.684496	1.496857	3.466423
H	-3.815801	-2.647688	2.433142
H	-4.553938	-2.155300	3.955839
H	-2.826544	-1.869756	3.675934
H	-2.088696	-0.071704	2.100914
H	-2.659727	-1.895148	0.689435
H	-0.369015	4.580612	-2.811608
H	-1.499469	6.591930	-1.881676
H	-3.087642	6.378655	0.009108

H	-3.579906	4.169639	1.009374
H	-4.197816	0.860121	-1.470892
H	-6.246893	0.479023	-2.748387
H	-7.476870	-1.676500	-2.555461
H	-6.593844	-3.460314	-1.055787
H	-4.521858	-3.087022	0.224130
H	2.559912	-0.357040	-2.499283
H	4.135276	-1.764615	-3.766796
H	4.033637	-4.249519	-3.566746
H	2.326578	-5.283017	-2.077696
H	0.750713	-3.872523	-0.817755
C	1.046594	-0.485152	0.922570
C	0.828700	-1.706150	1.860371
F	1.331575	-1.442836	3.090549
F	1.393728	-2.873167	1.455253
F	-0.489975	-1.971547	2.033482
C	2.538283	-0.116157	0.799365
O	0.255318	0.487881	1.224625
C	3.603737	-1.027846	0.775320
C	4.925441	-0.586791	0.682934
C	5.181372	0.778959	0.600388
Br	6.997156	1.391496	0.467987
C	4.145132	1.709766	0.617013
C	2.832961	1.250969	0.723378
H	3.420685	-2.093207	0.833080
H	5.741841	-1.301124	0.671728
H	4.358019	2.772244	0.559847
H	2.006190	1.951371	0.765281

Statistical Thermodynamic Analysis
Temperature= 298.150 Kelvin Pressure= 1.00000 Atm
===
==============================
SCF Energy= -4881.14688418 Predicted Change= -1.288239D-09
Zero-point correction (ZPE)= -4880.5699 0.57696
Internal Energy (U)= -4880.5316 0.61522
Enthalpy (H)= -4880.5307 0.61617
Gibbs Free Energy (G)= -4880.6433 0.50358

Frequencies -- -144.7230 12.7018 18.7286

183.15K thermal correction = 0.541593
Single point SCF = -4884.231724

NMR Spectra

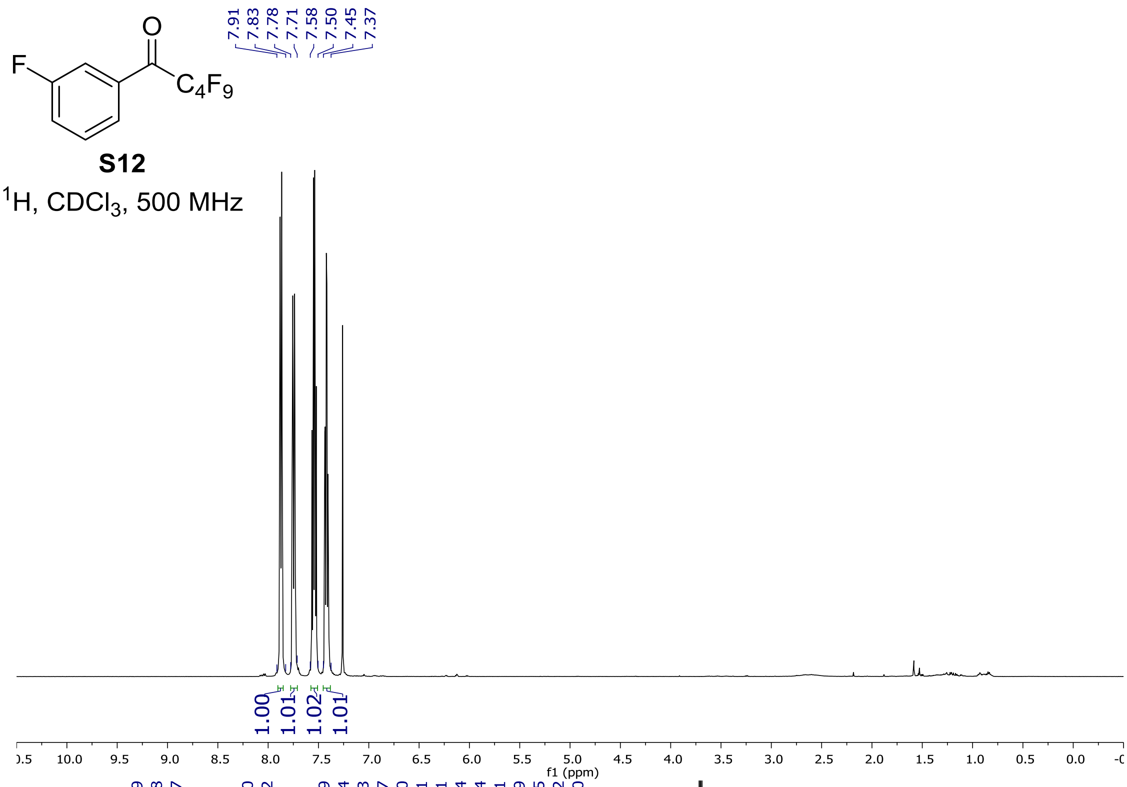

F
O
C_4F_9
S12
^{1}H, $CDCl_3$, 500 MHz
7.91
7.83
7.78
7.71
7.58
7.50
7.45
7.37
1.00
1.01
1.02
1.01
f1 (ppm)

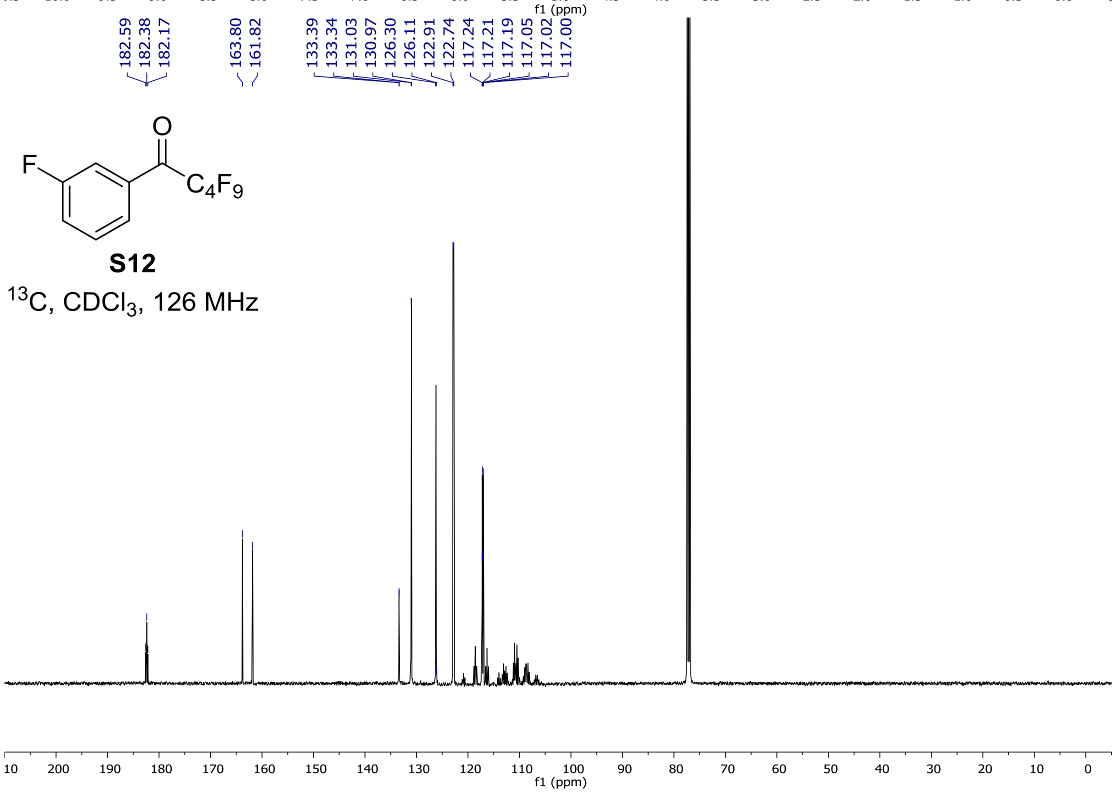

182.59
182.38
182.17
163.80
161.82
133.39
133.34
131.03
130.97
126.30
126.11
122.91
122.74
117.24
117.21
117.19
117.05
117.02
117.00
F
O
C_4F_9
S12
^{13}C, $CDCl_3$, 126 MHz
f1 (ppm)

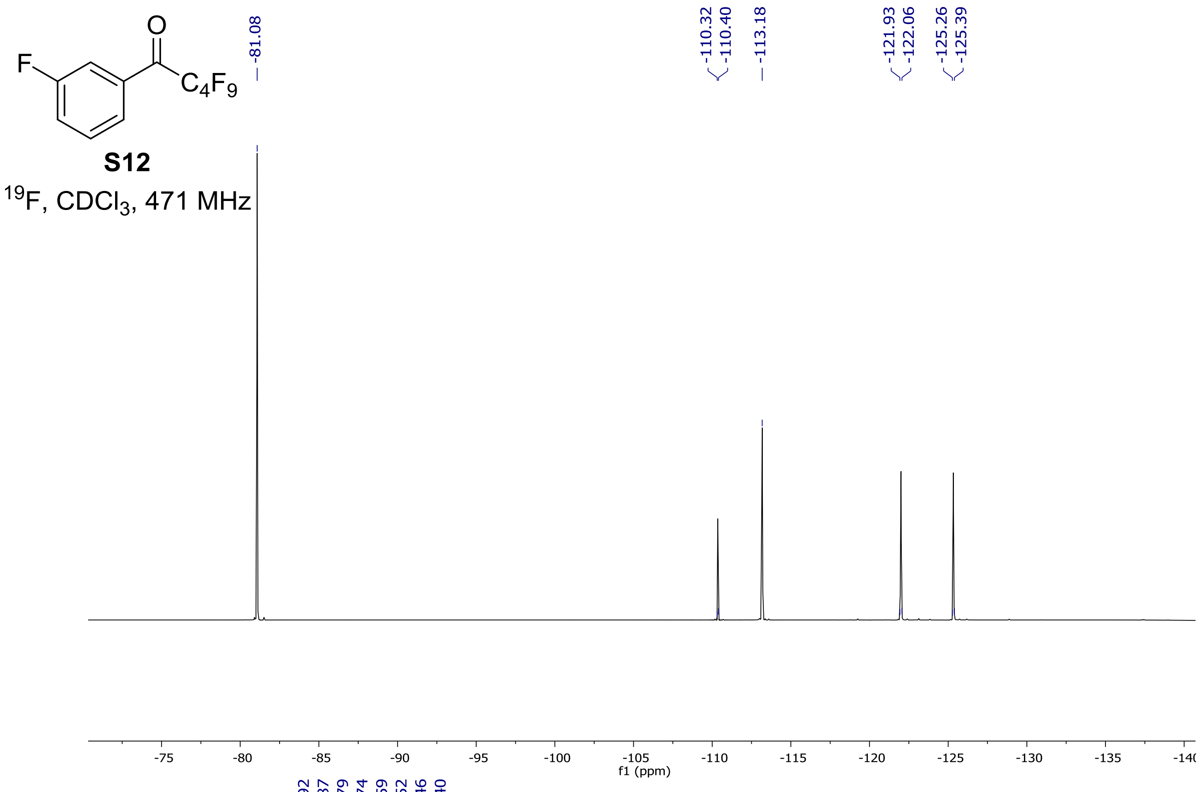
S12
19F, CDCl3, 471 MHz
-81.08
-110.32
-110.40
-113.18
-121.93
-122.06
-125.26
-125.39
f1 (ppm)

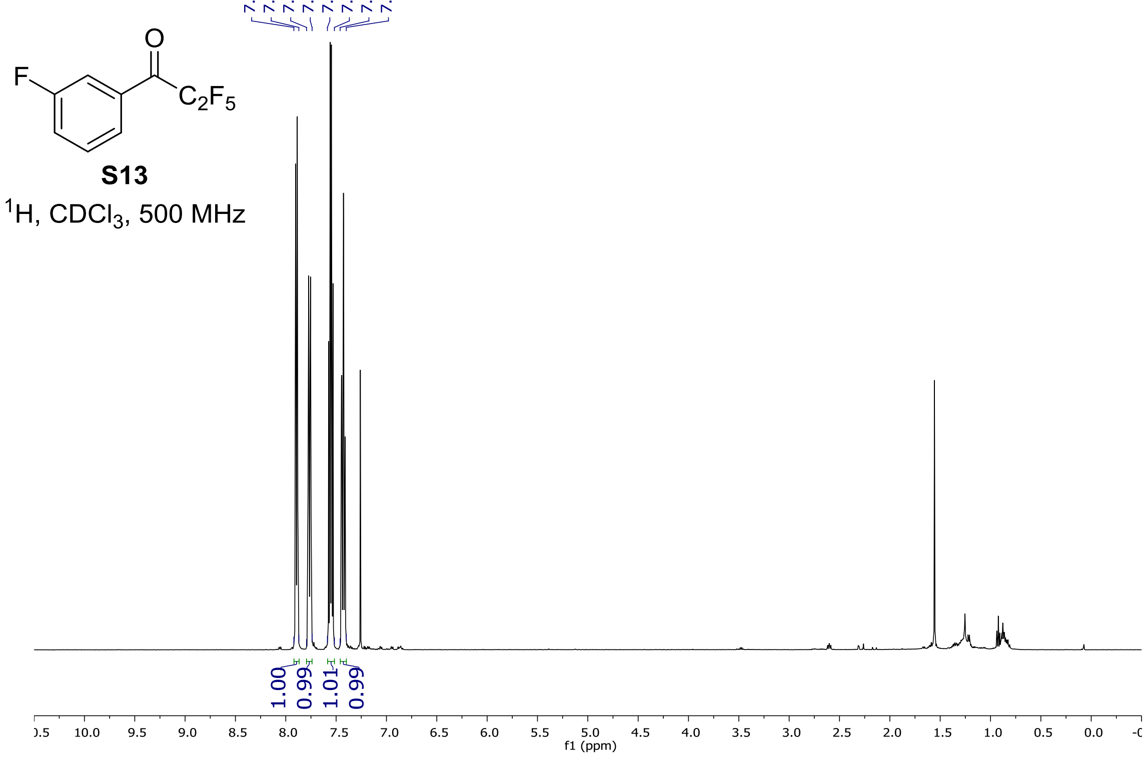
S13
1H, CDCl3, 500 MHz
7.92
7.87
7.79
7.74
7.59
7.52
7.46
7.40
1.00
0.99
1.01
0.99
f1 (ppm)

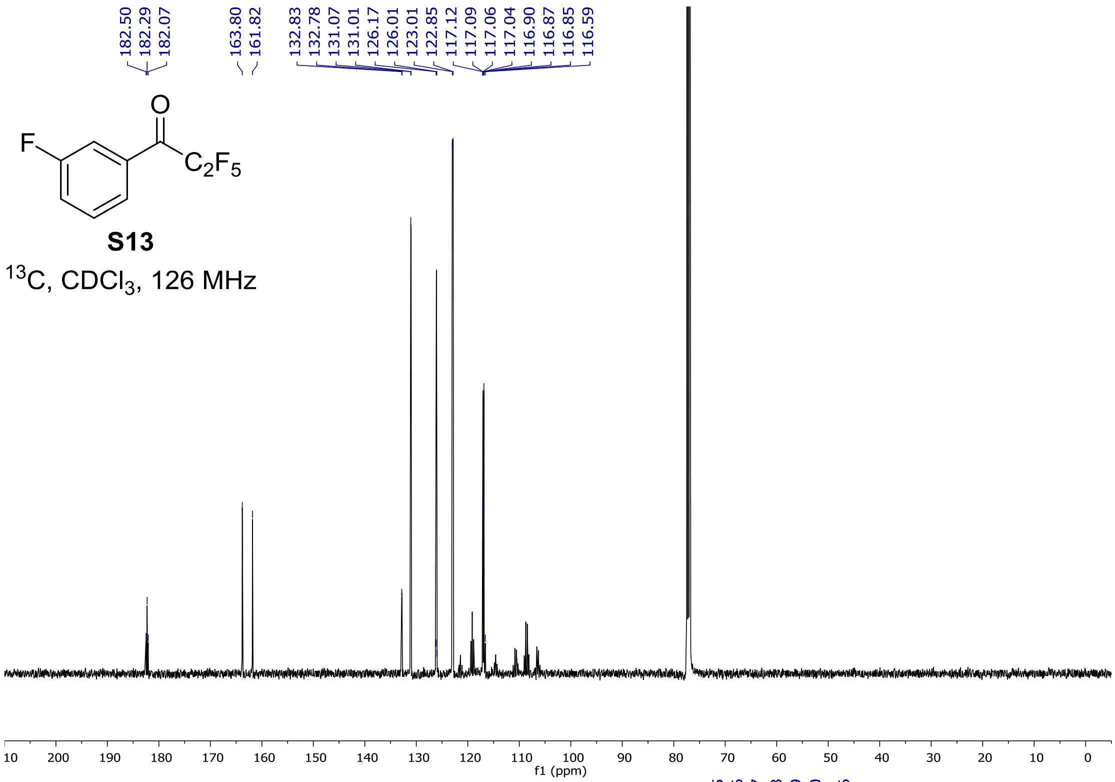
182.50
182.29
182.07
163.80
161.82
132.83
132.78
131.07
131.01
126.17
126.01
123.01
122.85
117.12
117.09
117.06
117.04
116.90
116.87
116.85
116.59
O
F
C_2F_5
S13
^{13}C, $CDCl_3$, 126 MHz
f1 (ppm)

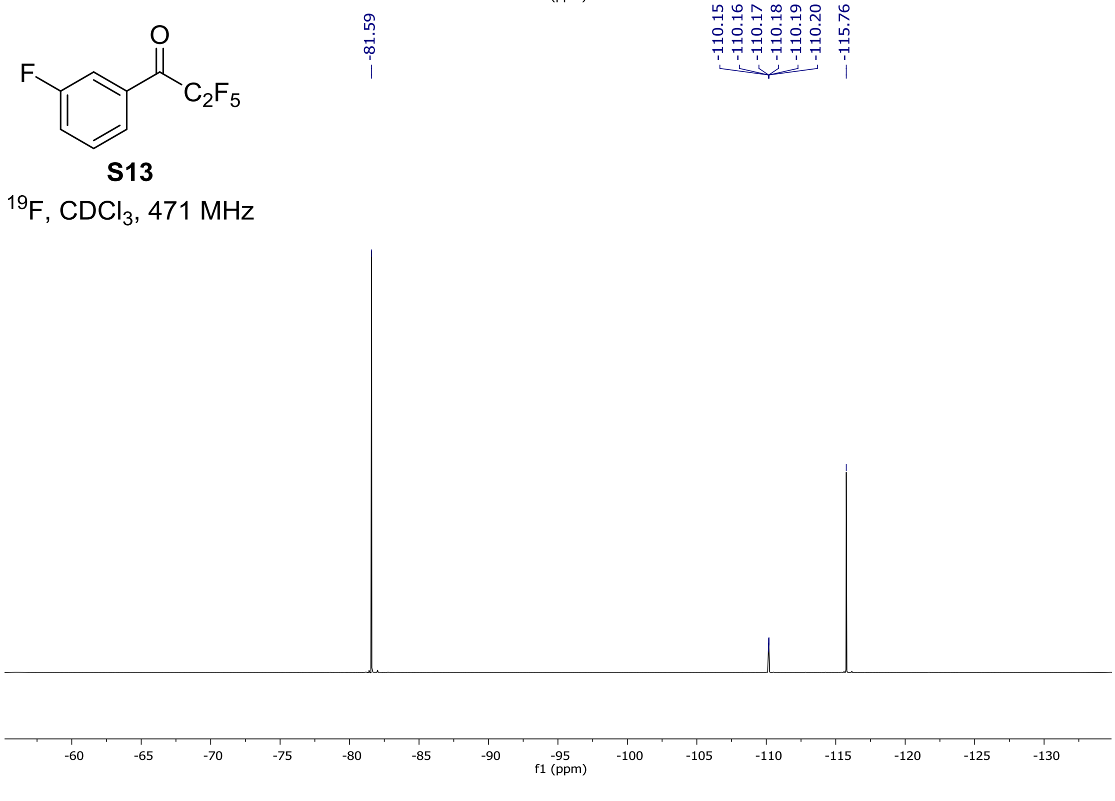
-81.59
-110.15
-110.16
-110.17
-110.18
-110.19
-110.20
-115.76
O
F
C_2F_5
S13
^{19}F, $CDCl_3$, 471 MHz
f1 (ppm)

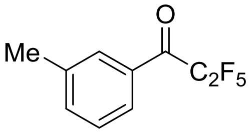

S17

^{1}H, $CDCl_3$, 500 MHz

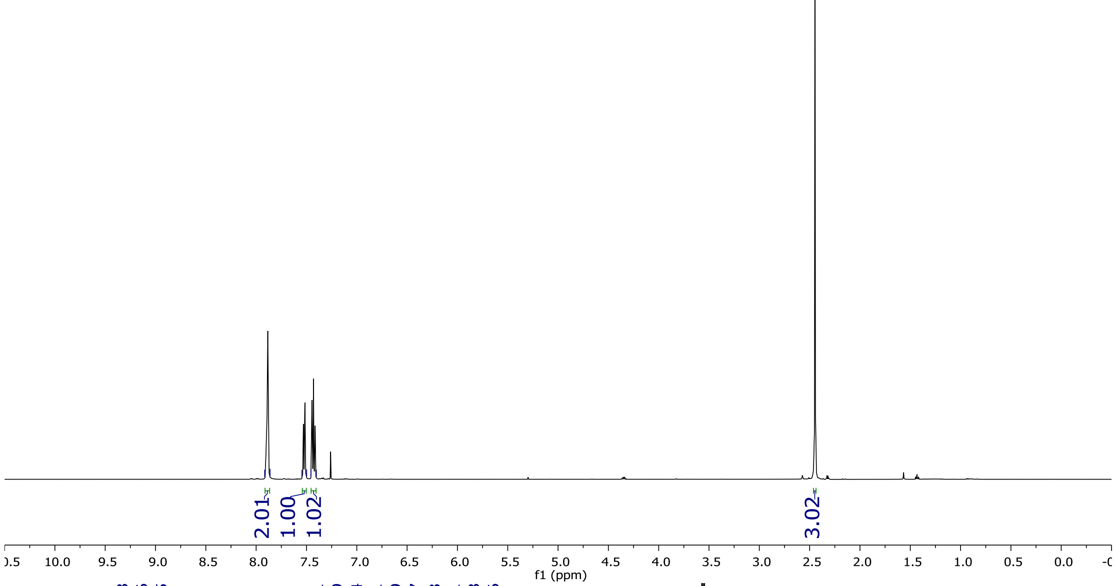

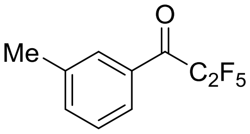

S17

^{13}C, $CDCl_3$, 126 MHz

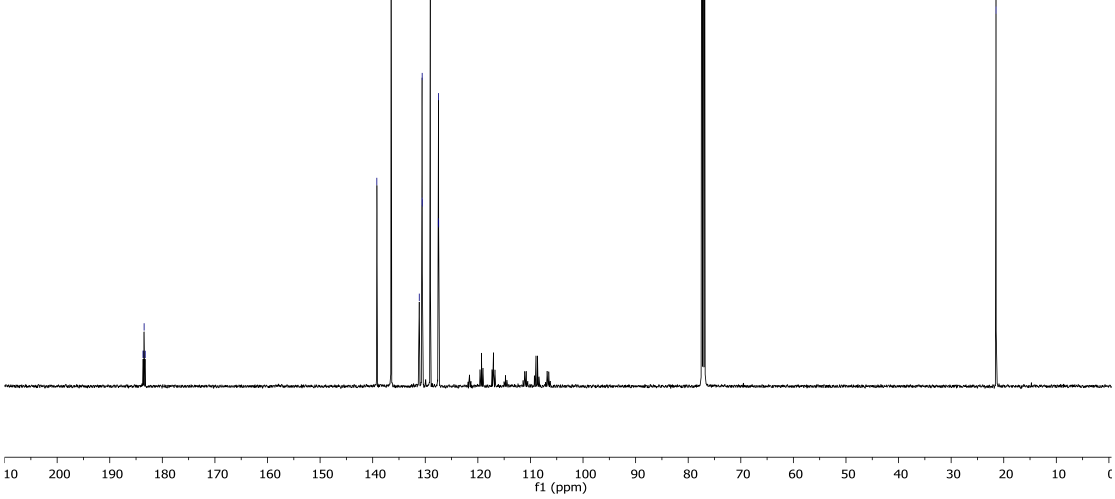

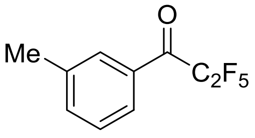

S17

^{19}F, $CDCl_3$, 471 MHz

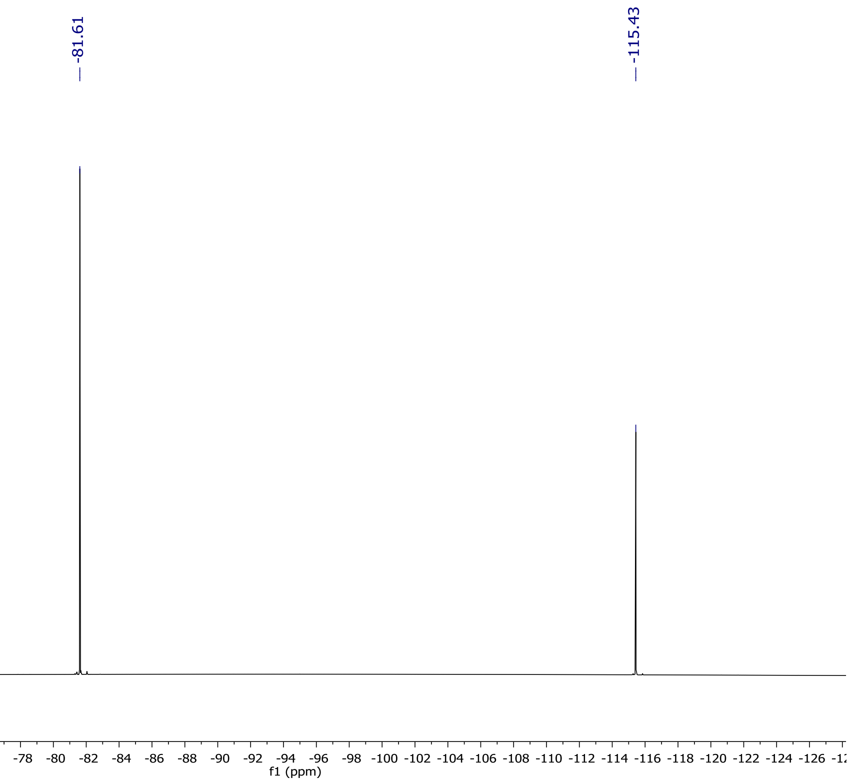

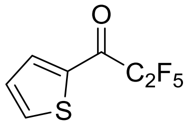

S18

^{1}H, $CDCl_3$, 400 MHz

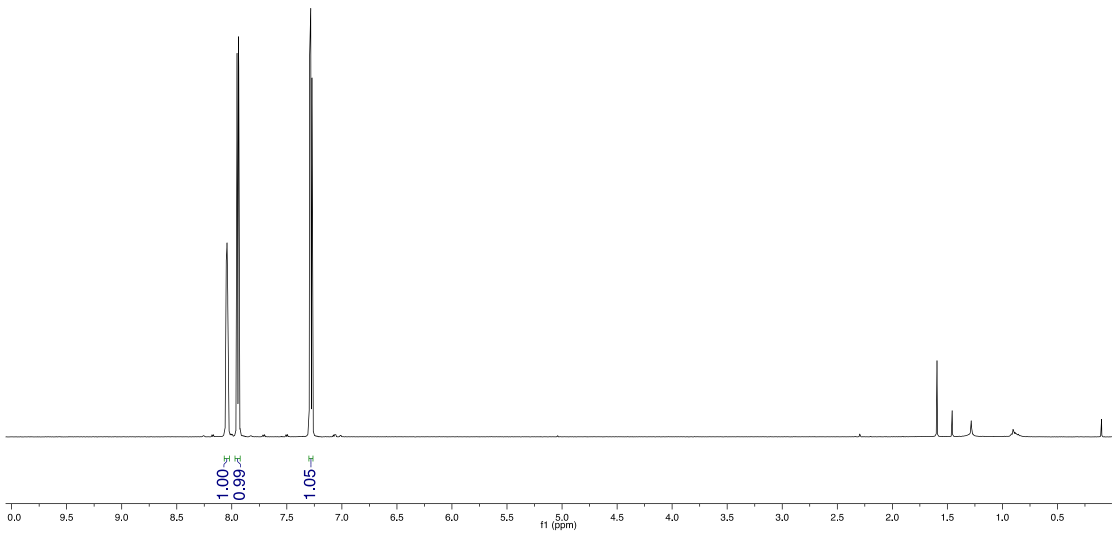

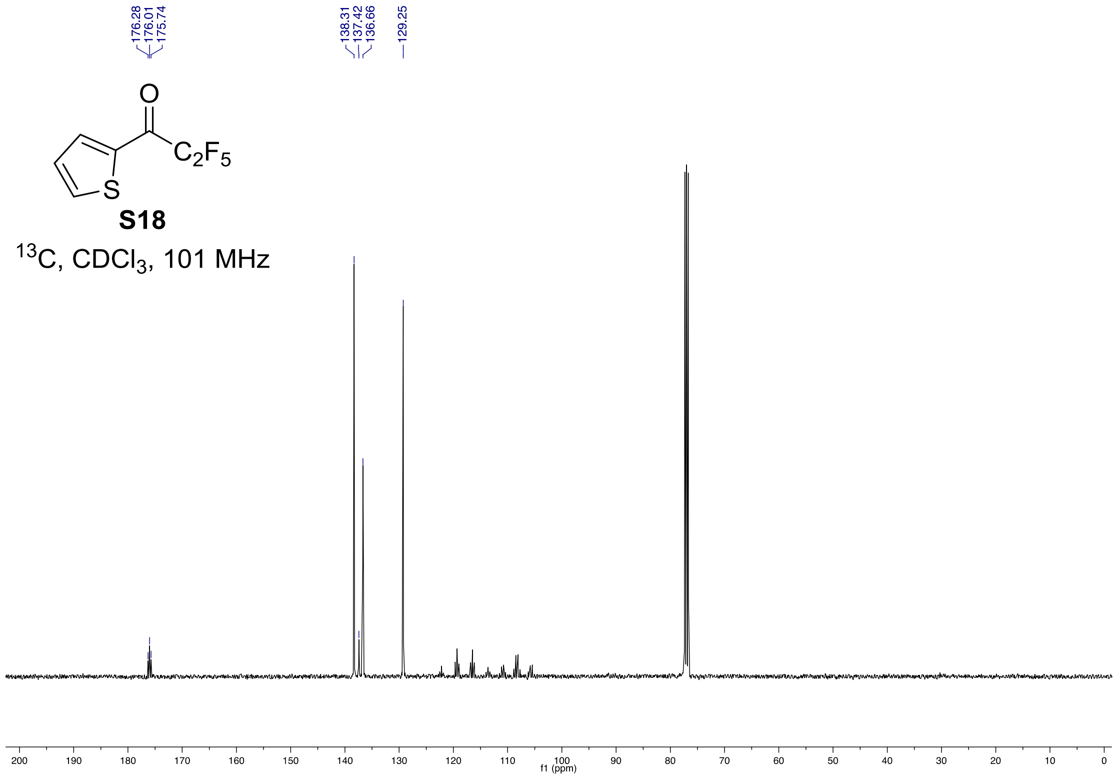
176.28
176.01
175.74
138.31
137.42
136.66
129.25
O
C2F5
S
S18
13C, CDCl3, 101 MHz
f1 (ppm)

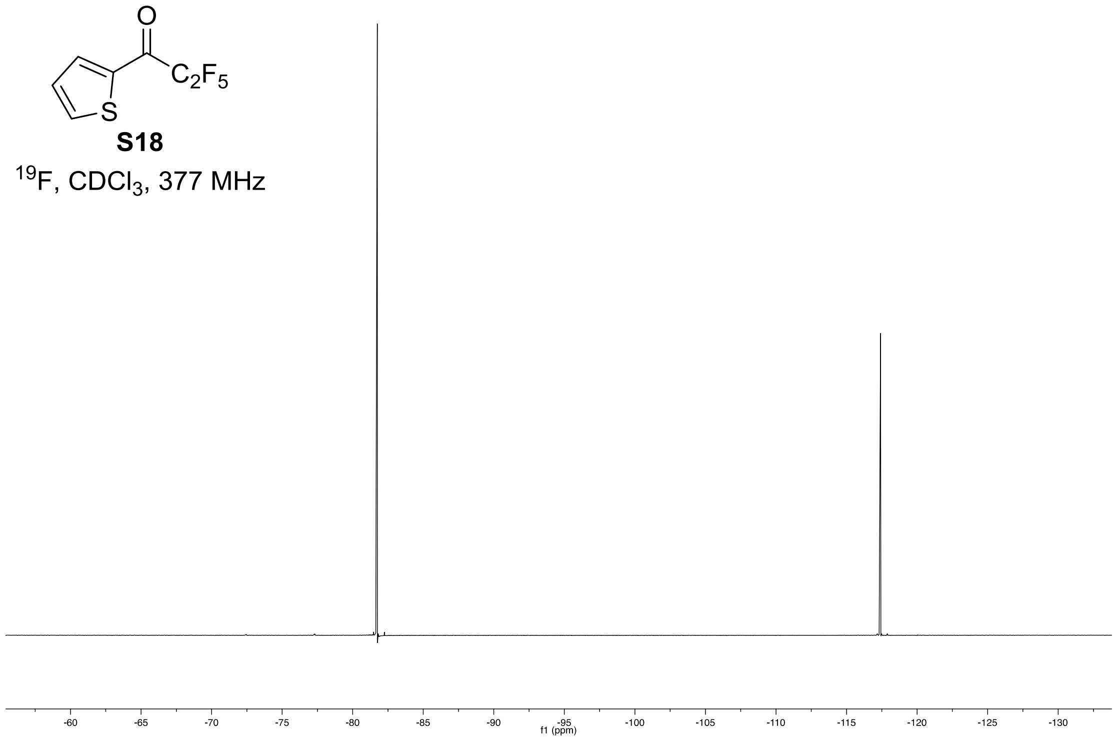
O
C2F5
S
S18
19F, CDCl3, 377 MHz
f1 (ppm)

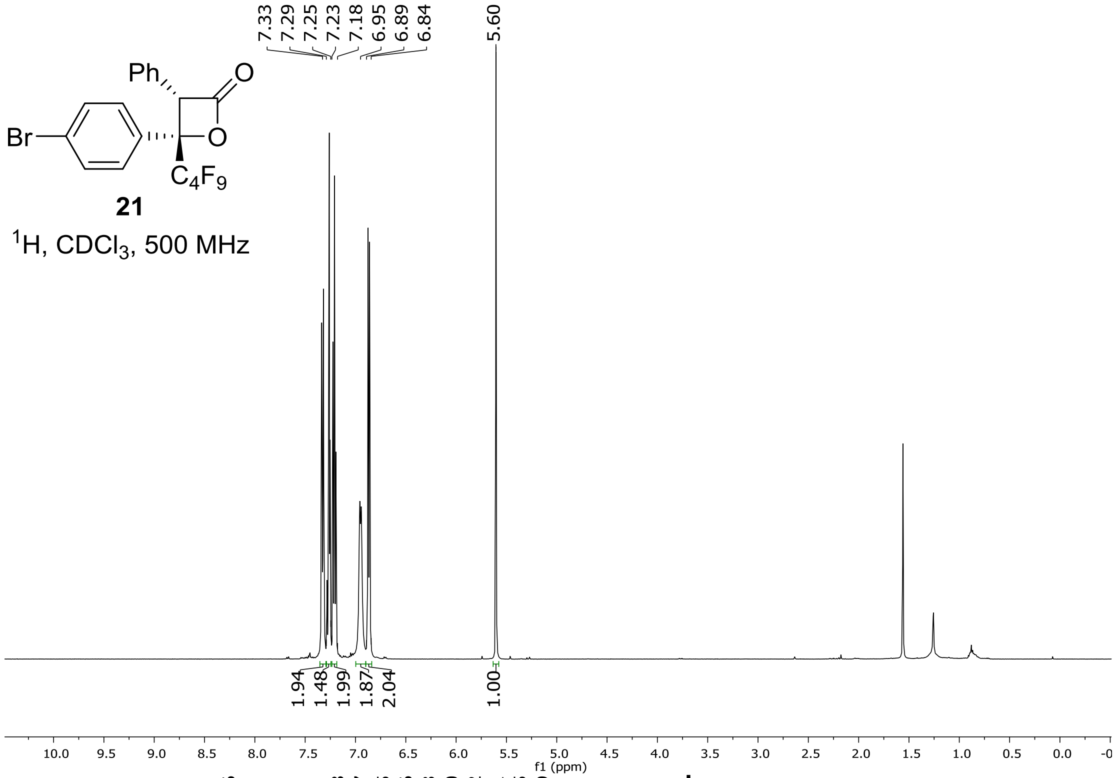
7.33
7.29
7.25
7.23
7.18
6.95
6.89
6.84
5.60
Ph
O
Br
O
C_4F_9
21
^{1}H, $CDCl_3$, 500 MHz
1.94
1.48
1.99
1.87
2.04
1.00
10.0 9.5 9.0 8.5 8.0 7.5 7.0 6.5 6.0 5.5 5.0 4.5 4.0 3.5 3.0 2.5 2.0 1.5 1.0 0.5 0.0 -0
f1 (ppm)

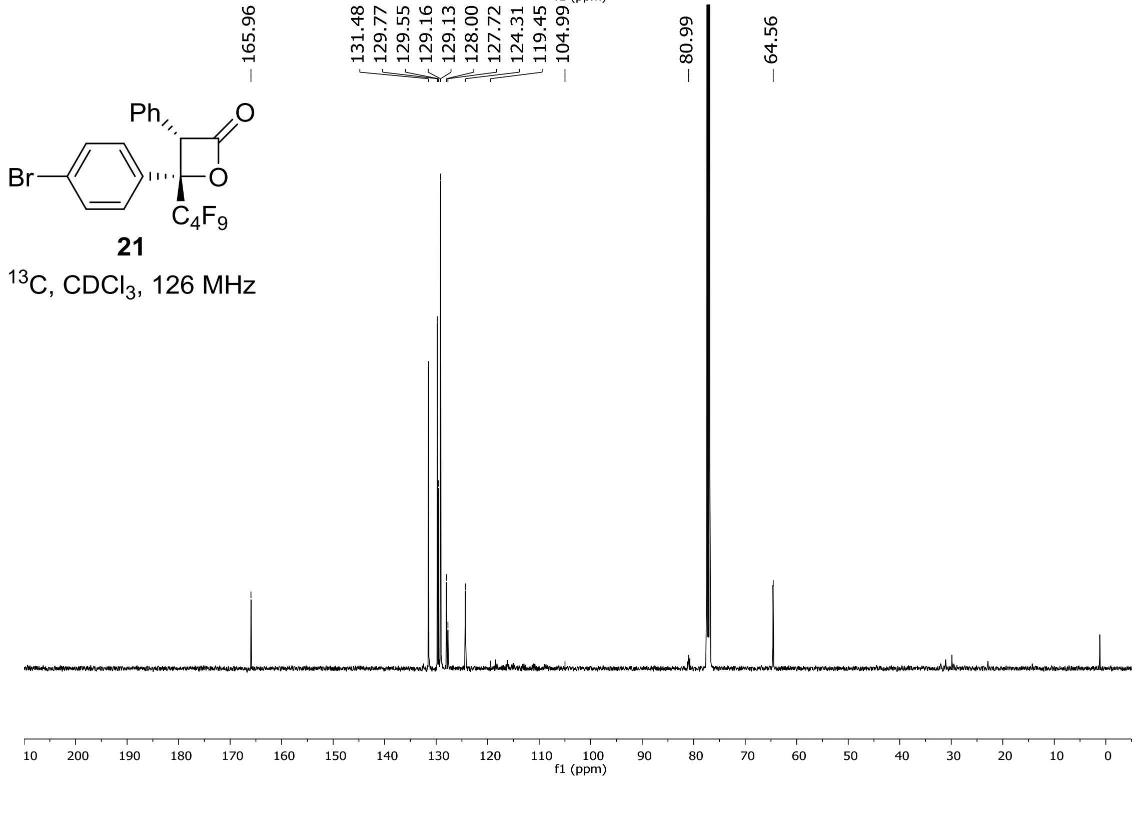
165.96
131.48
129.77
129.55
129.16
129.13
128.00
127.72
124.31
119.45
104.99
80.99
64.56
Ph
O
Br
O
C_4F_9
21
^{13}C, $CDCl_3$, 126 MHz
10 200 190 180 170 160 150 140 130 120 110 100 90 80 70 60 50 40 30 20 10 0
f1 (ppm)

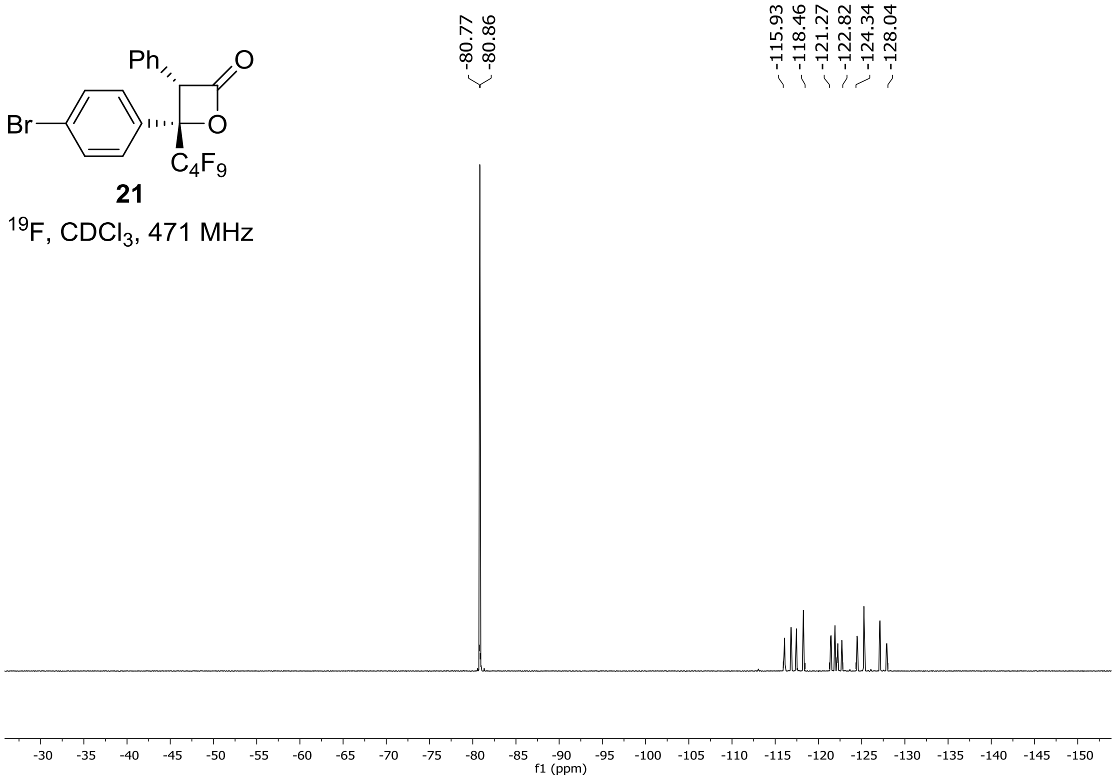

Ph
O
Br
O
C_4F_9
21
^{19}F, $CDCl_3$, 471 MHz
-80.77
-80.86
-115.93
-118.46
-121.27
-122.82
-124.34
-128.04
f1 (ppm)

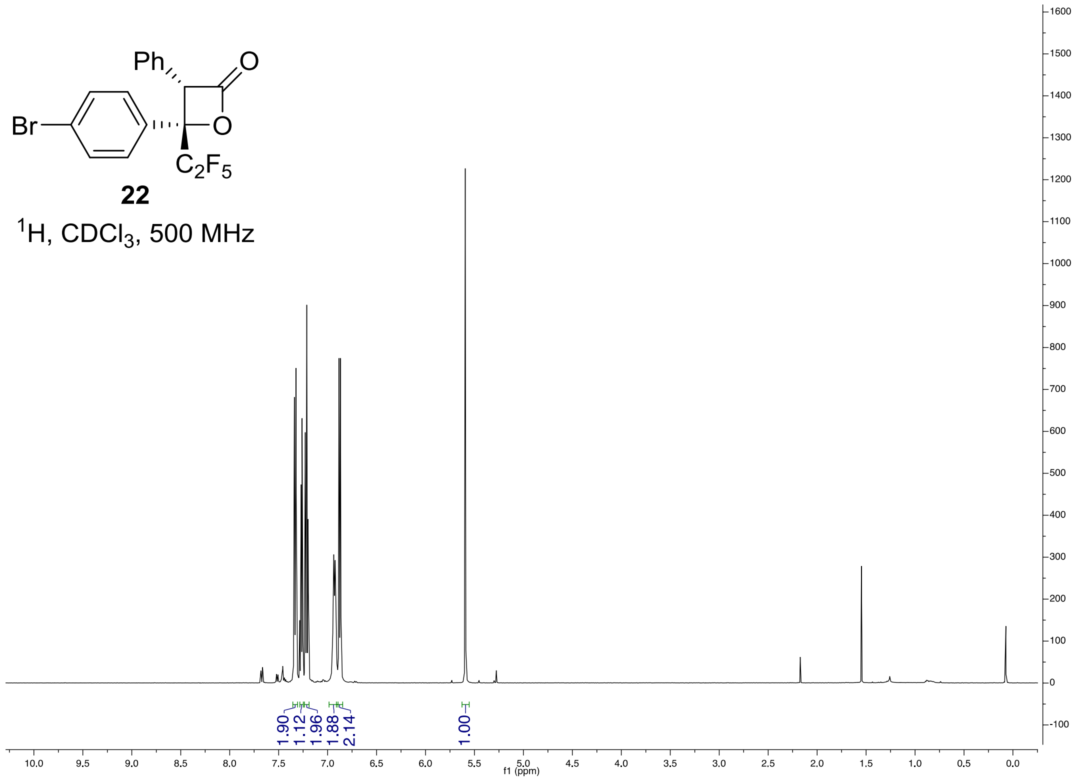

Ph
O
Br
O
C_2F_5
22
^1H, $CDCl_3$, 500 MHz
1.90
1.12
1.96
1.88
2.14
1.00
f1 (ppm)

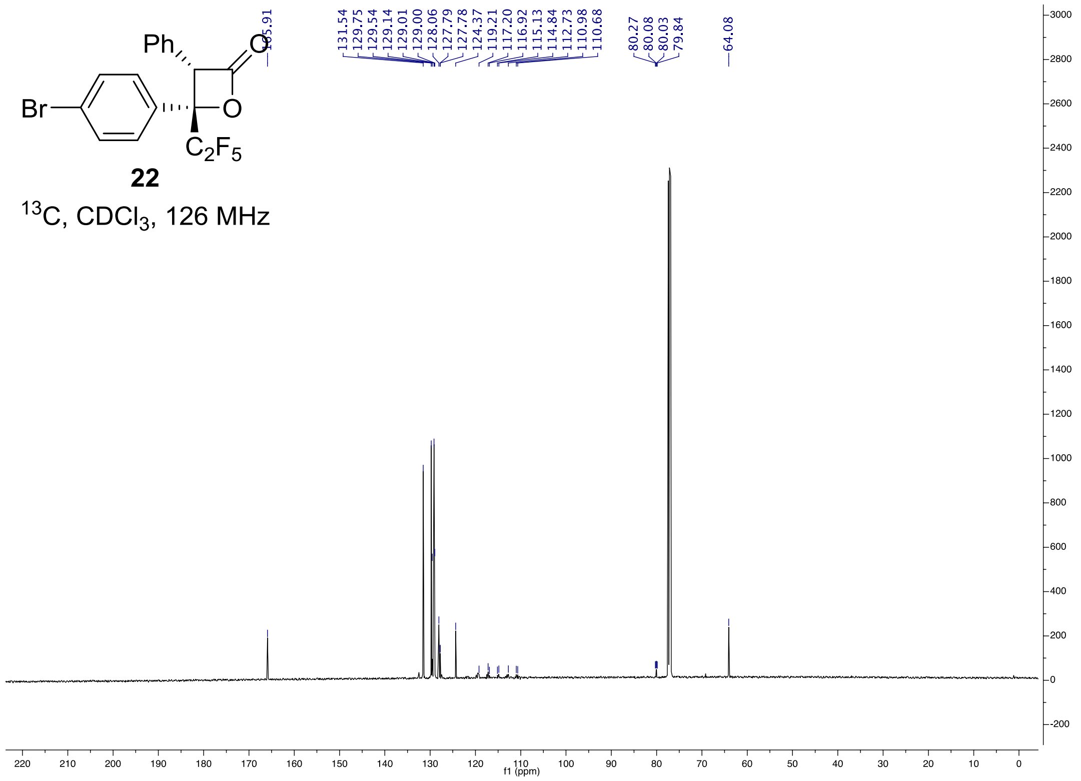

165.91
131.54
129.75
129.54
129.14
129.01
129.00
128.06
127.79
127.78
124.37
119.21
117.20
116.92
115.13
114.84
112.73
110.98
110.68
80.27
80.08
80.03
79.84
64.08
Ph
O
Br
O
C_2F_5
22
^{13}C, $CDCl_3$, 126 MHz
f1 (ppm)

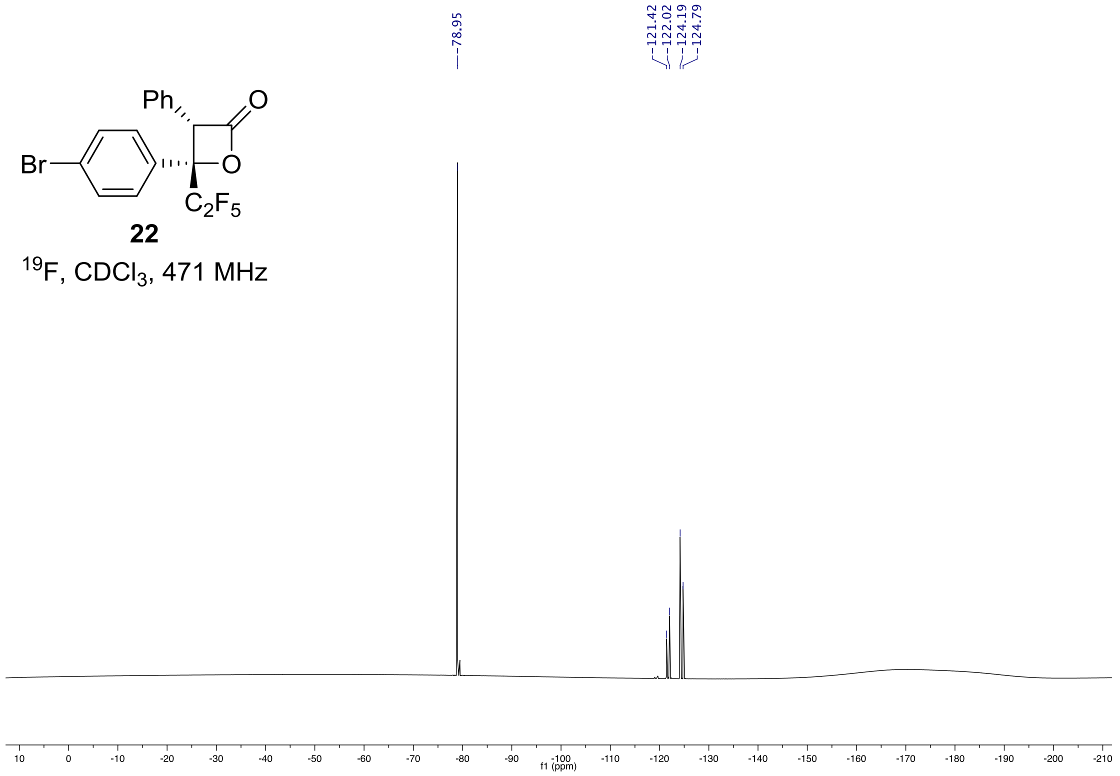

-78.95
-121.42
-122.02
-124.19
-124.79
Ph
O
Br
O
C_2F_5
22
^{19}F, $CDCl_3$, 471 MHz
f1 (ppm)

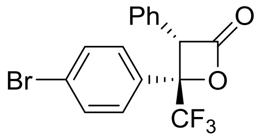

23

^{1}H, $CDCl_3$, 500 MHz

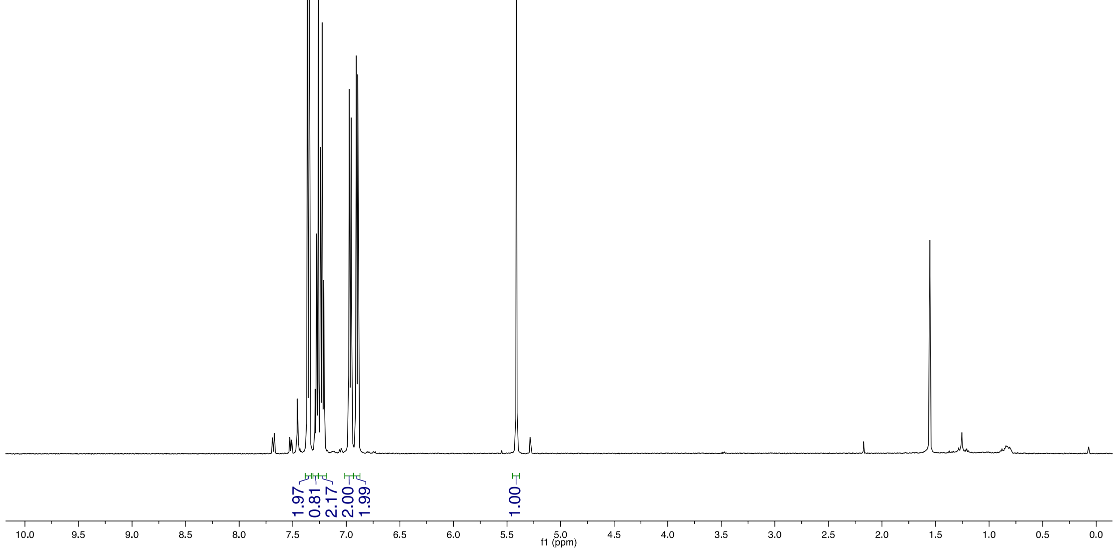

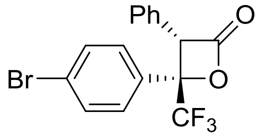

23

^{13}C, $CDCl_3$, 126 MHz

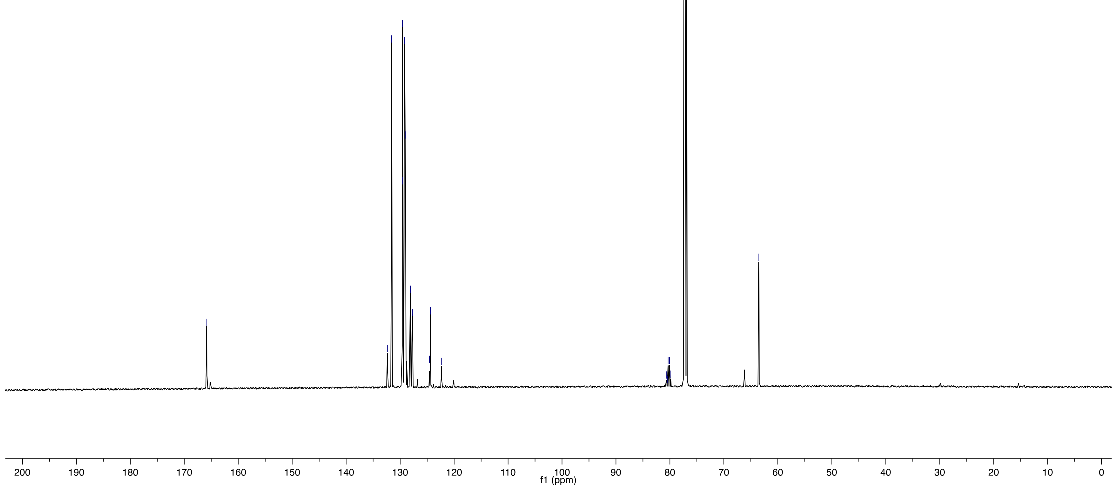

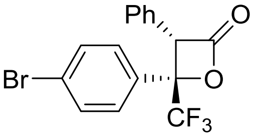

23

^{19}F, $CDCl_3$, 471 MHz

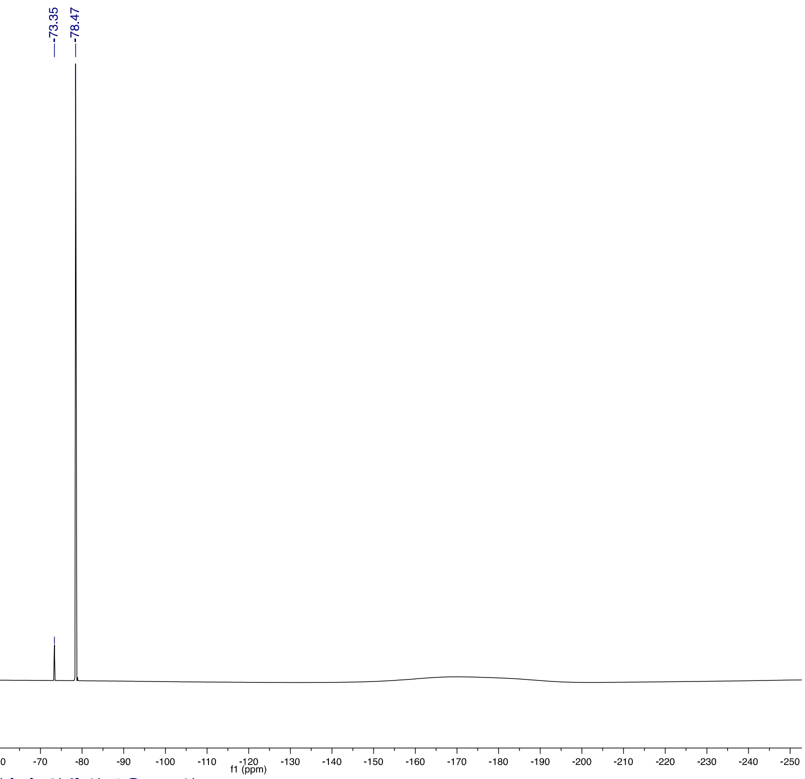

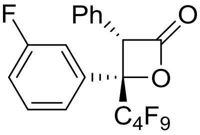

24

^{1}H, $CDCl_3$, 126 MHz

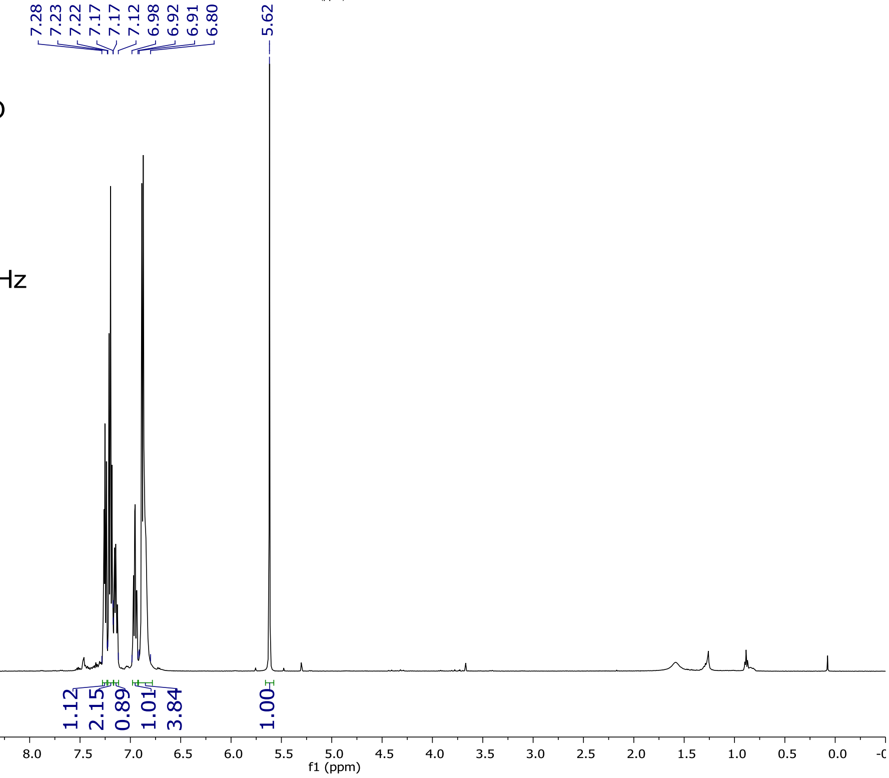

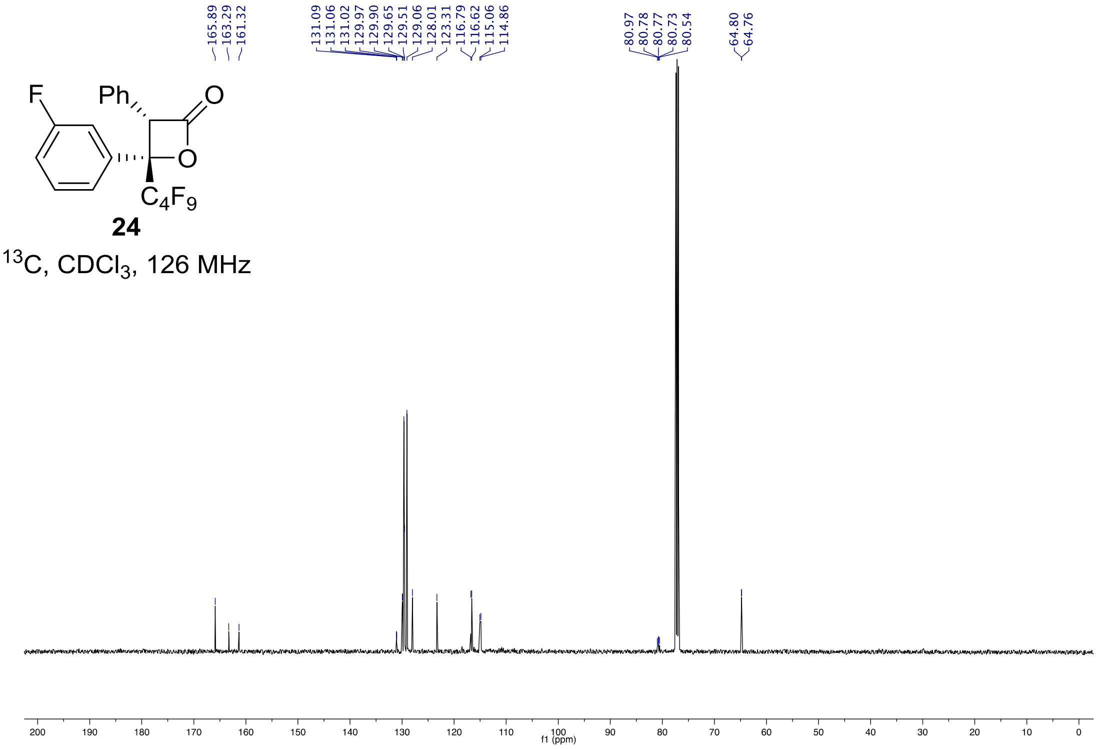
165.89
163.29
161.32
131.09
131.06
131.02
129.97
129.90
129.65
129.51
129.06
128.01
123.31
116.79
116.62
115.06
114.86
80.97
80.78
80.77
80.73
80.54
64.80
64.76
F
Ph
O
O
C_4F_9
24
^{13}C, $CDCl_3$, 126 MHz
f1 (ppm)

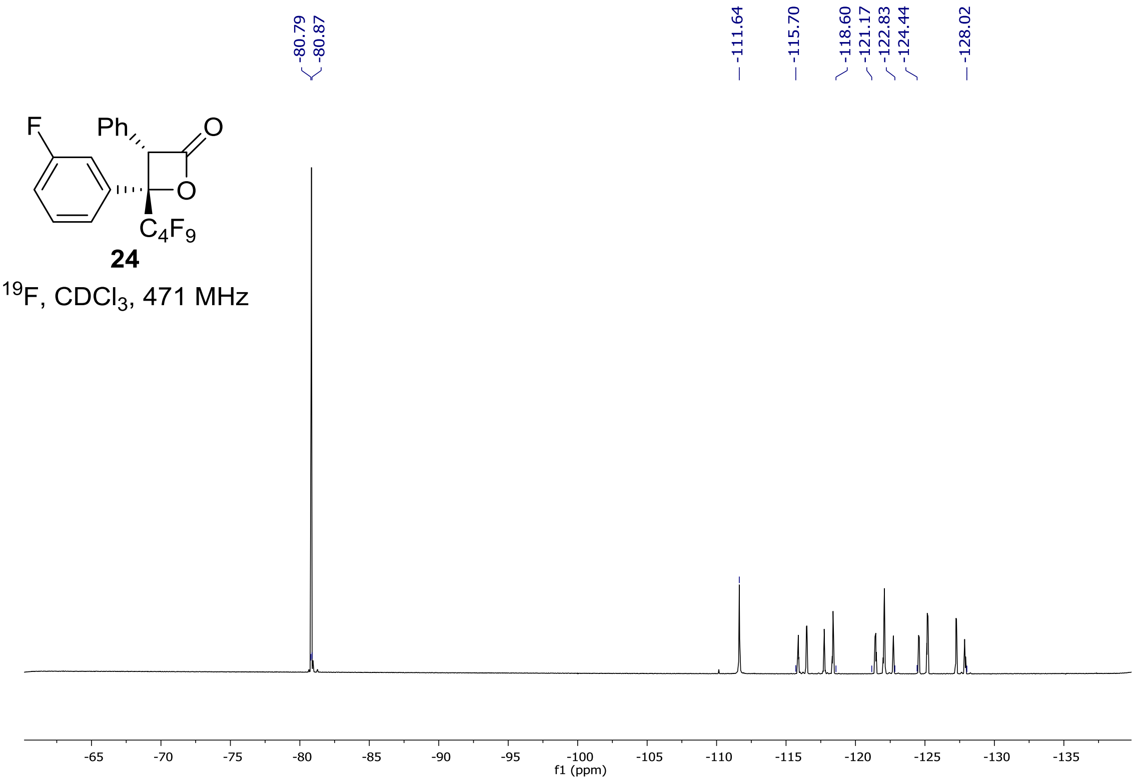
-80.79
-80.87
-111.64
-115.70
-118.60
-121.17
-122.83
-124.44
-128.02
F
Ph
O
O
C_4F_9
24
^{19}F, $CDCl_3$, 471 MHz
f1 (ppm)

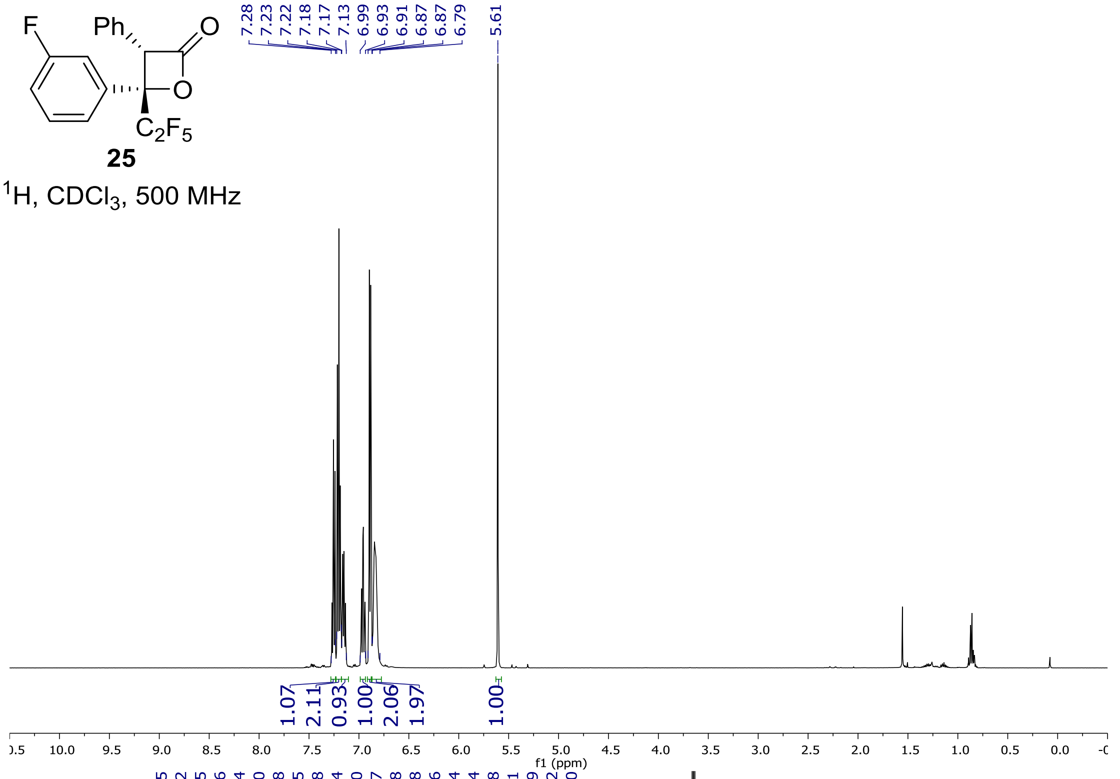
F
Ph
O
O
C_2F_5
25
^1H, $CDCl_3$, 500 MHz
7.28 7.23 7.22 7.18 7.17 7.13 6.99 6.93 6.91 6.87 6.87 6.79
5.61
1.07 2.11 0.93 1.00 2.06 1.97
1.00
f1 (ppm)

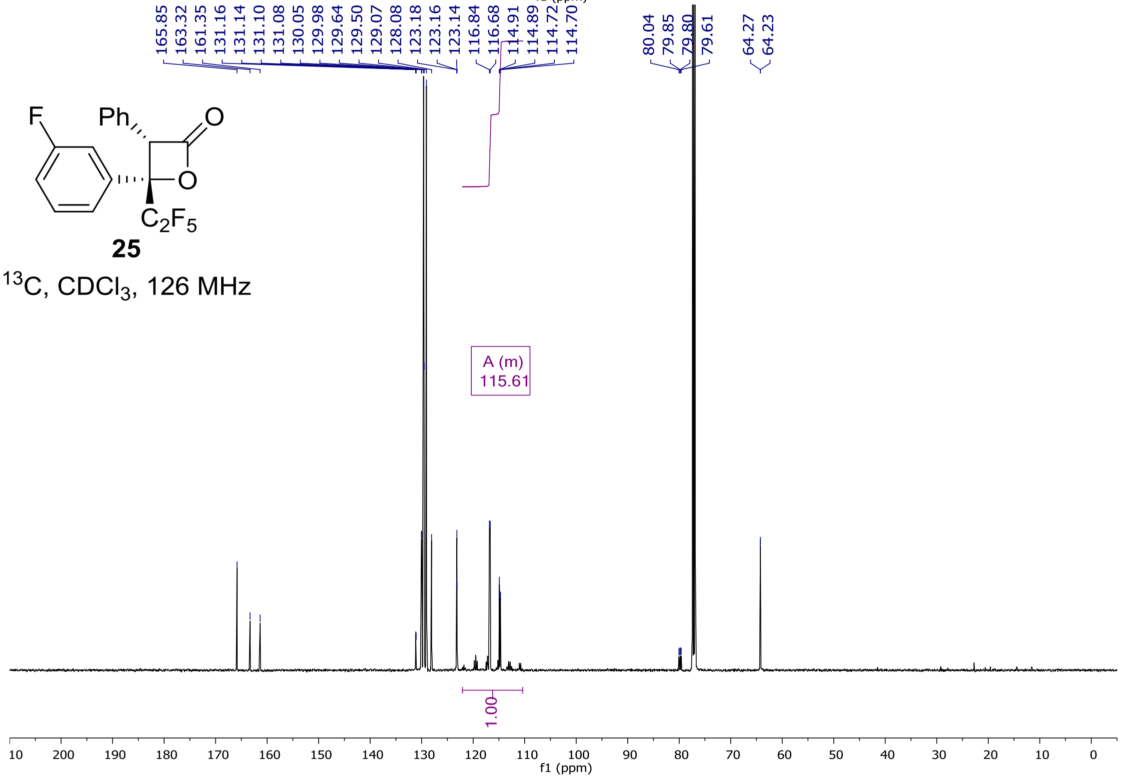
165.85 163.32 161.35 131.16 131.14 131.10 131.08 130.05 129.98 129.64 129.50 129.07 128.08 123.18 123.16 123.14 116.84 116.68 114.91 114.89 114.72 114.70
80.04 79.85 79.80 79.61
64.27 64.23
F
Ph
O
O
C_2F_5
25
^{13}C, $CDCl_3$, 126 MHz
A (m)
115.61
1.00
f1 (ppm)

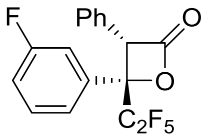

25

^{19}F, $CDCl_3$, 471 MHz

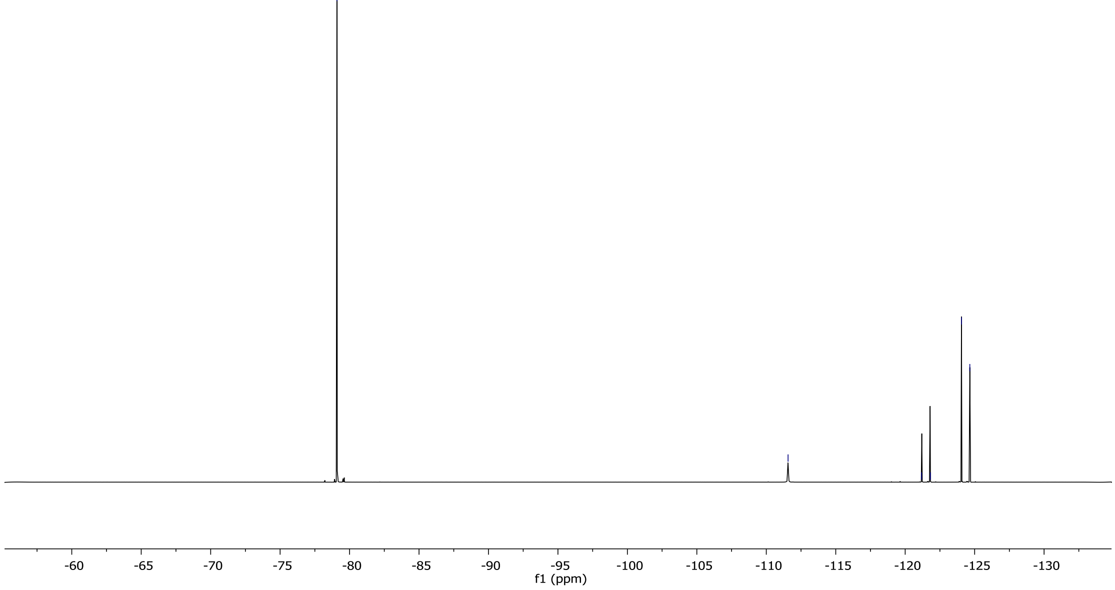

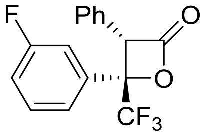

26

^{1}H, $CDCl_3$, 500 MHz

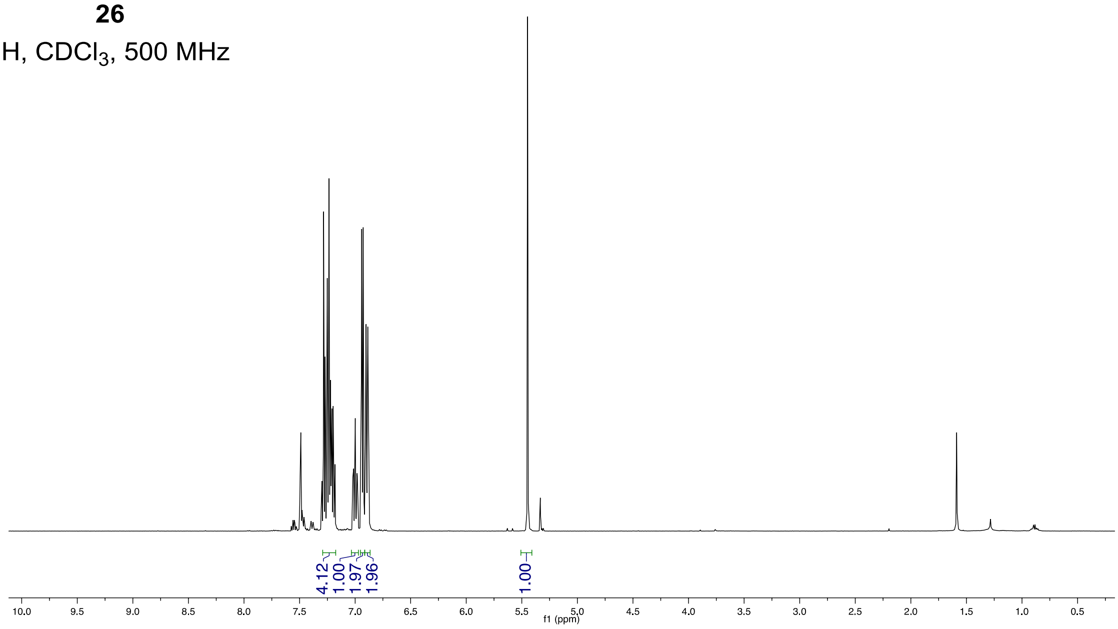

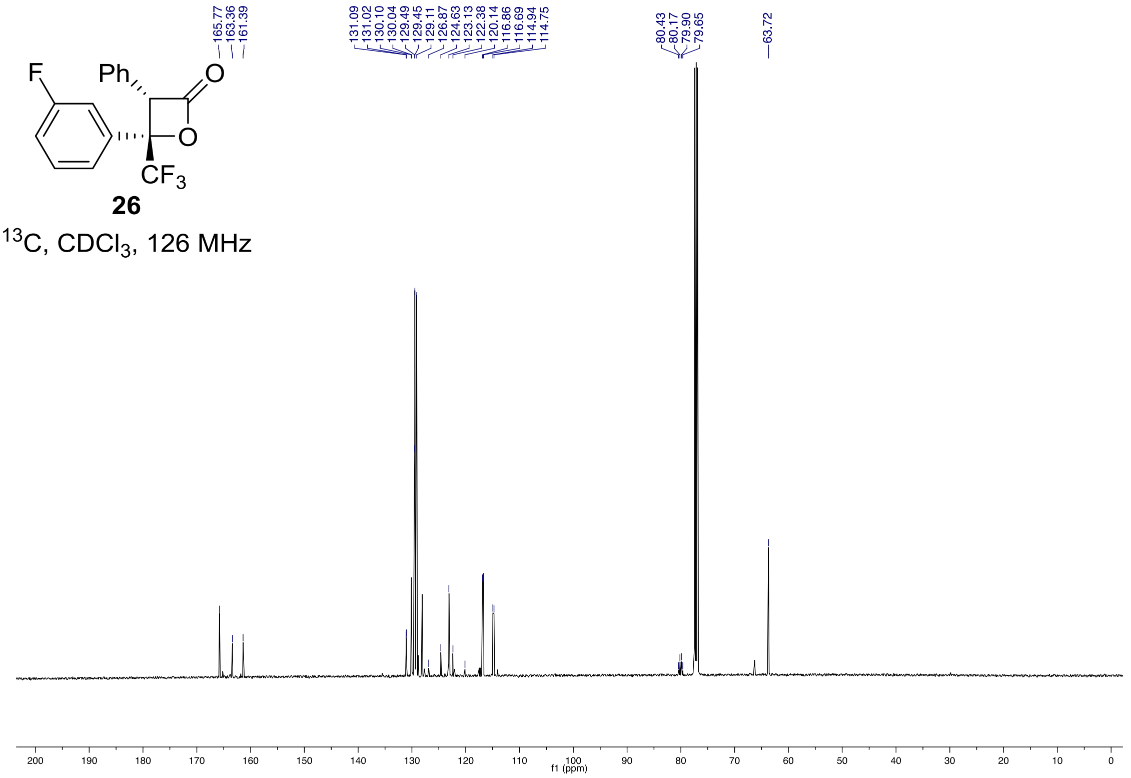
F
Ph
O
O
CF3
26
13C, CDCl3, 126 MHz
165.77
163.36
161.39
131.09
131.02
130.10
130.04
129.49
129.45
129.11
126.87
124.63
123.13
122.38
120.14
116.86
116.69
114.94
114.75
80.43
80.17
79.90
79.65
63.72
f1 (ppm)

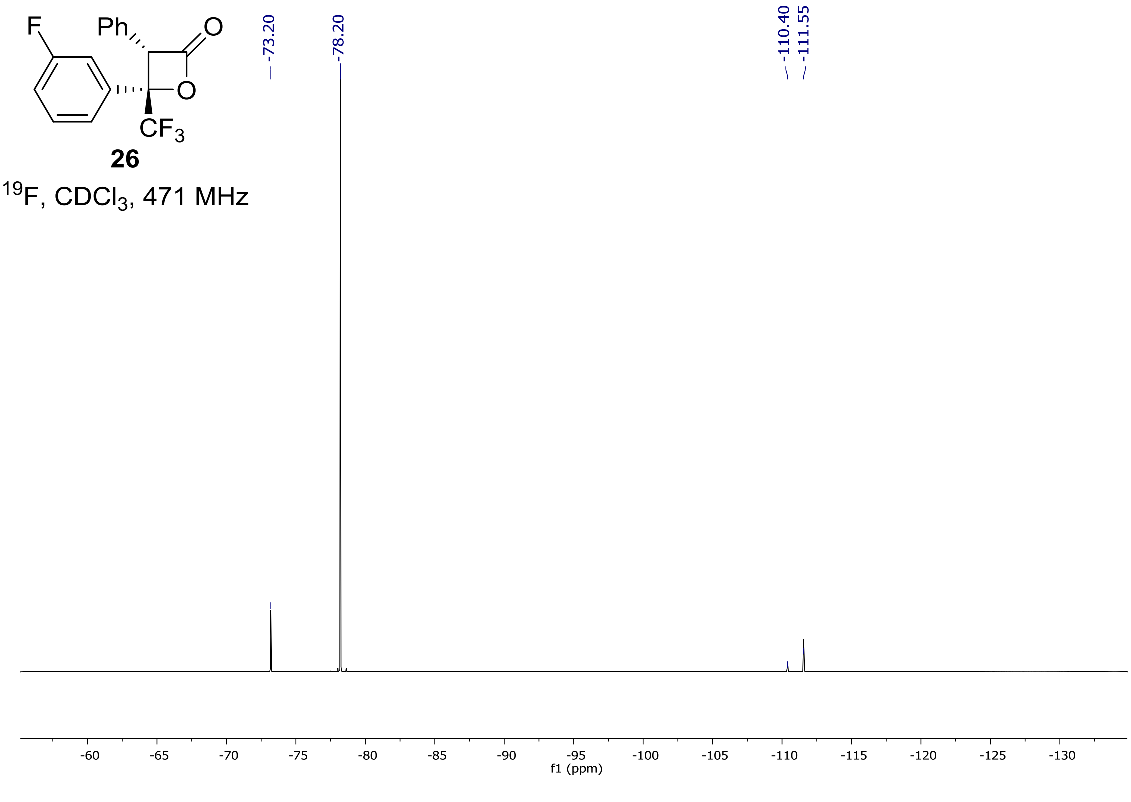
F
Ph
O
O
CF3
26
19F, CDCl3, 471 MHz
-73.20
-78.20
-110.40
-111.55
f1 (ppm)

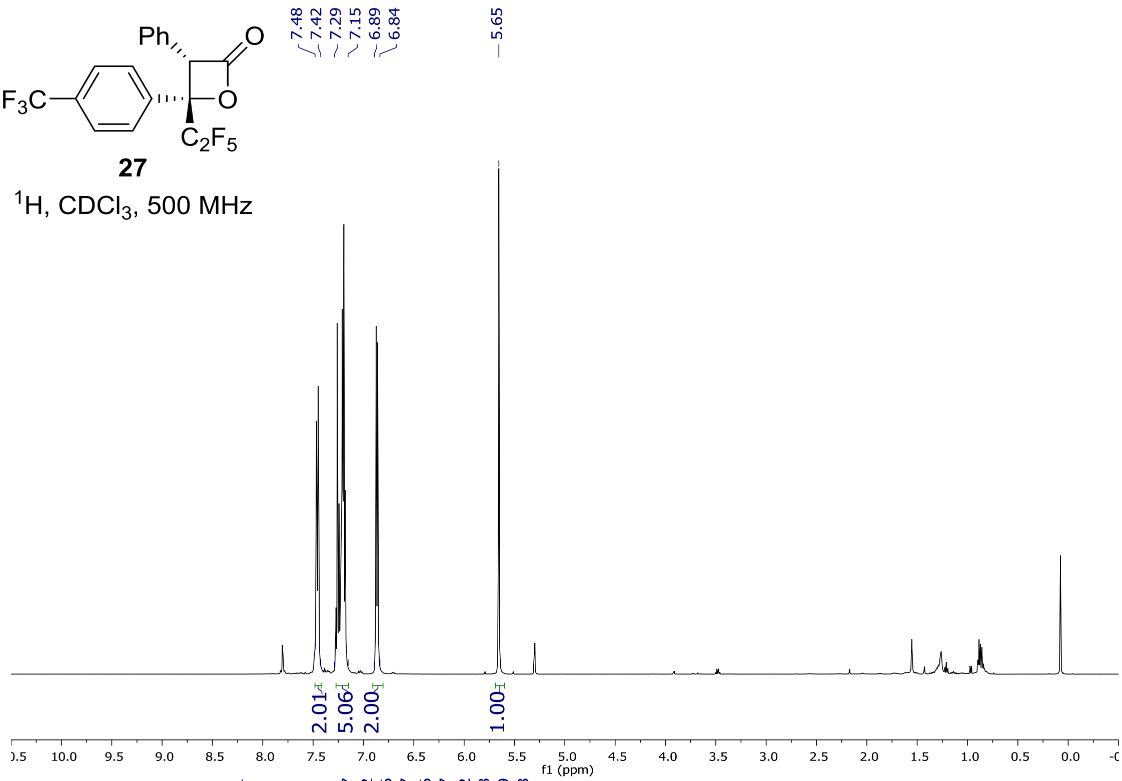
7.48
7.42
7.29
7.15
6.89
6.84
5.65
Ph
O
F_3C
O
C_2F_5
27
^1H, $CDCl_3$, 500 MHz
2.01
5.06
2.00
1.00
f1 (ppm)

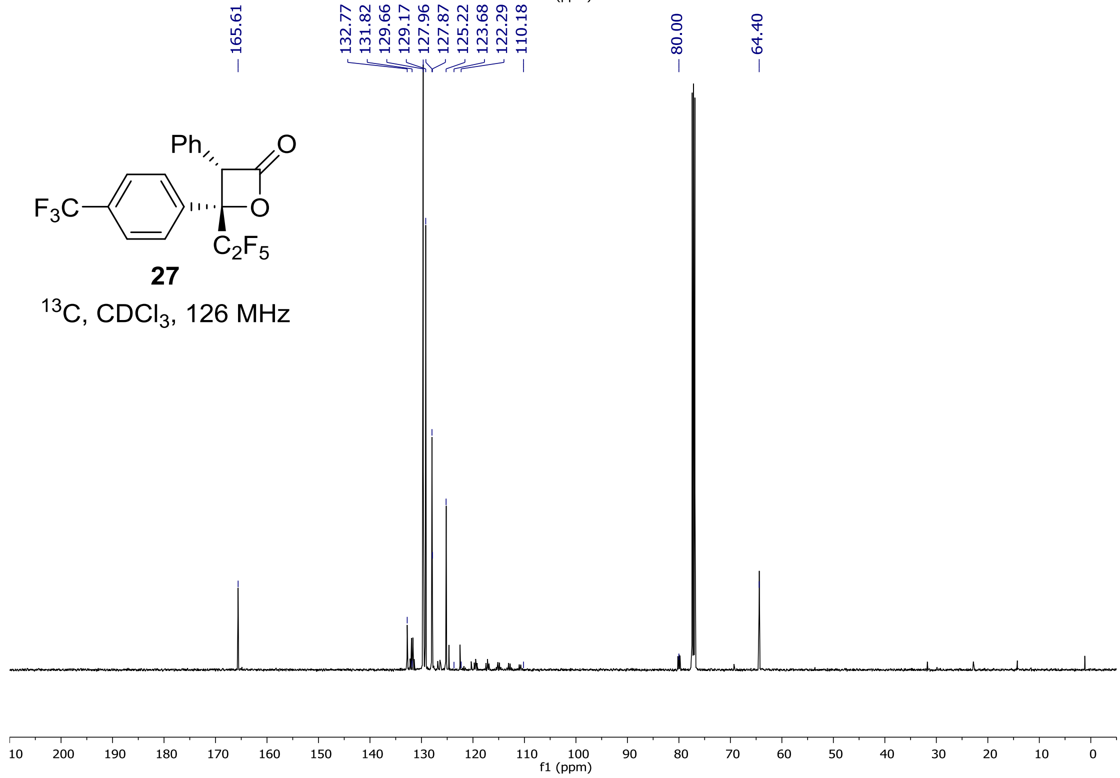
165.61
132.77
131.82
129.66
129.17
127.96
127.87
125.22
123.68
122.29
110.18
80.00
64.40
Ph
O
F_3C
O
C_2F_5
27
^{13}C, $CDCl_3$, 126 MHz
f1 (ppm)

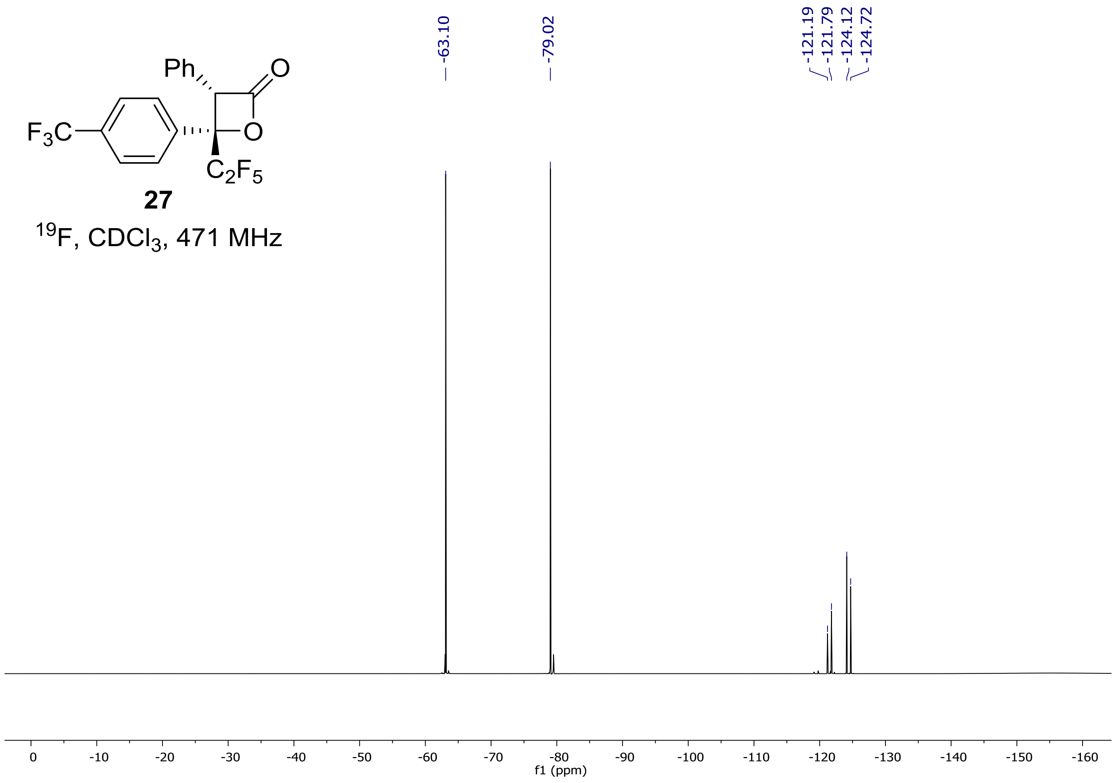

27

^{19}F, $CDCl_3$, 471 MHz

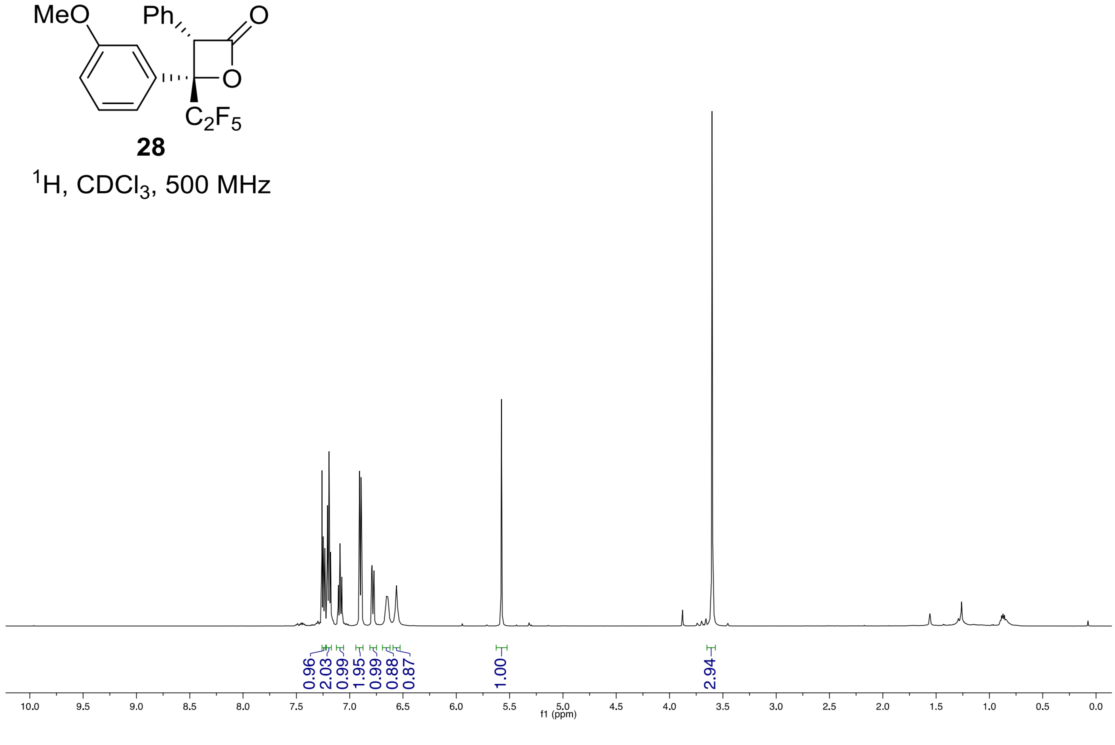

28

^{1}H, $CDCl_3$, 500 MHz

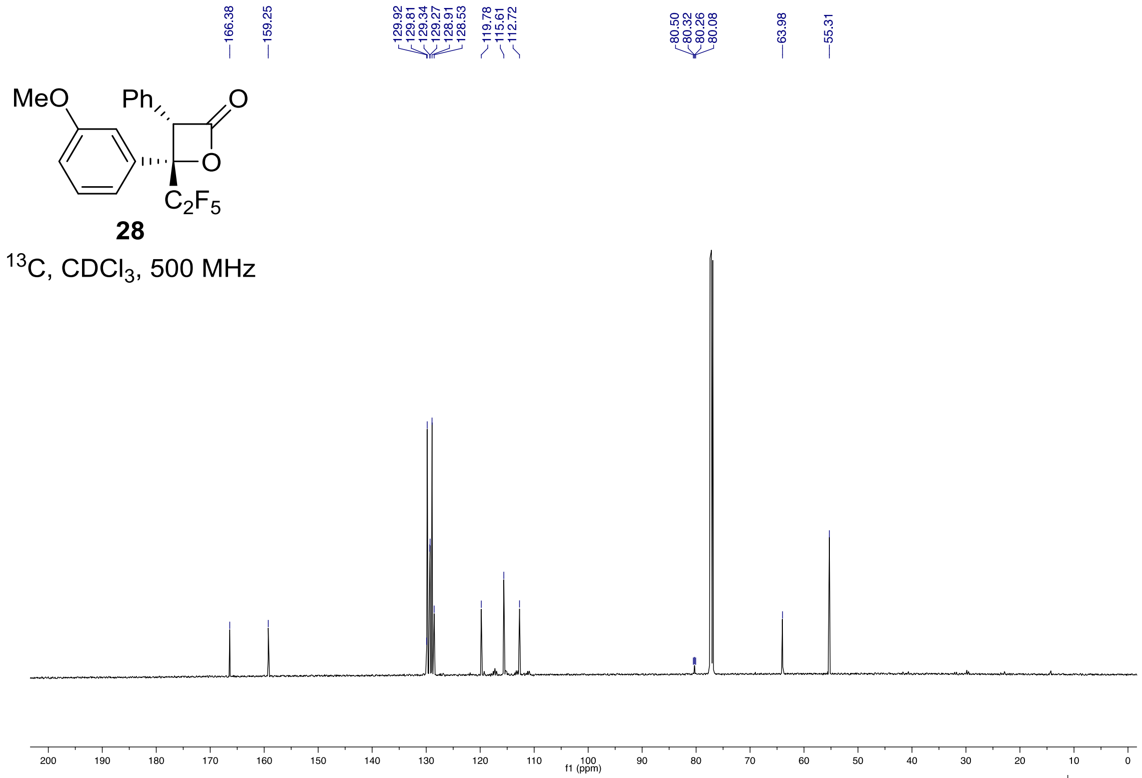
166.38
159.25
129.92
129.81
129.34
129.27
128.91
128.53
119.78
115.61
112.72
80.50
80.32
80.26
80.08
63.98
55.31
MeO
Ph
O
O
C_2F_5
28
^{13}C, $CDCl_3$, 500 MHz
f1 (ppm)

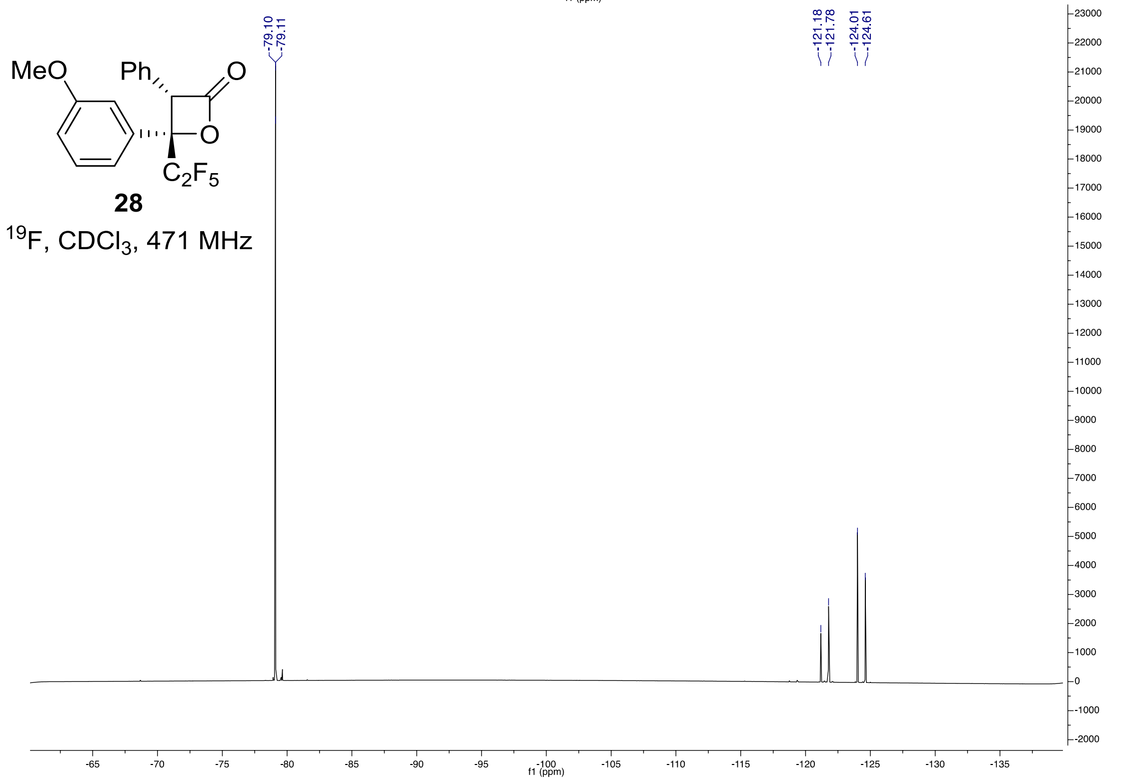
-79.10
-79.11
-121.18
-121.78
-124.01
-124.61
MeO
Ph
O
O
C_2F_5
28
^{19}F, $CDCl_3$, 471 MHz
f1 (ppm)

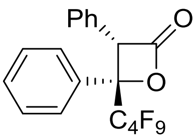

29

^1H, $CDCl_3$, 500 MHz

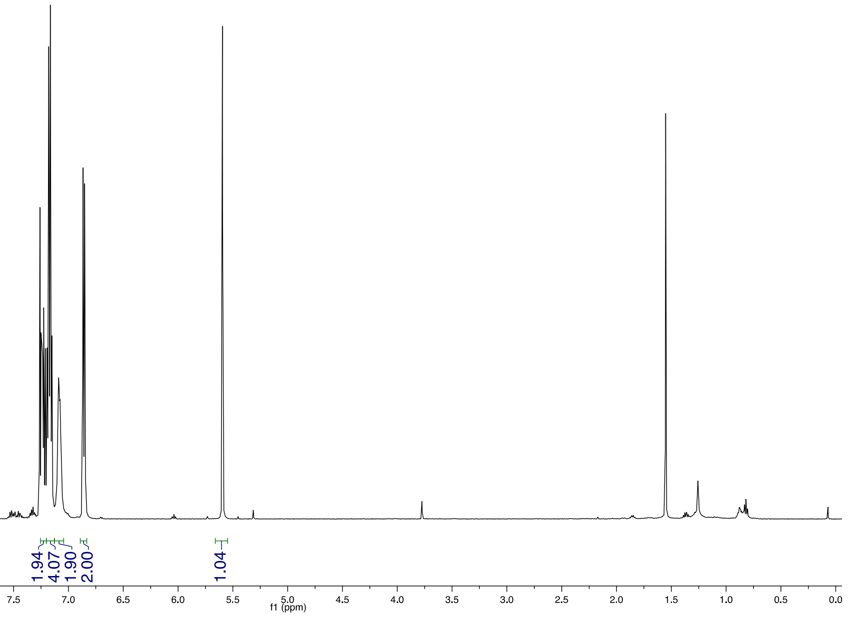

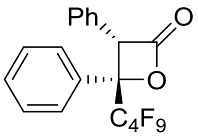

29

^{13}C, $CDCl_3$, 126 MHz

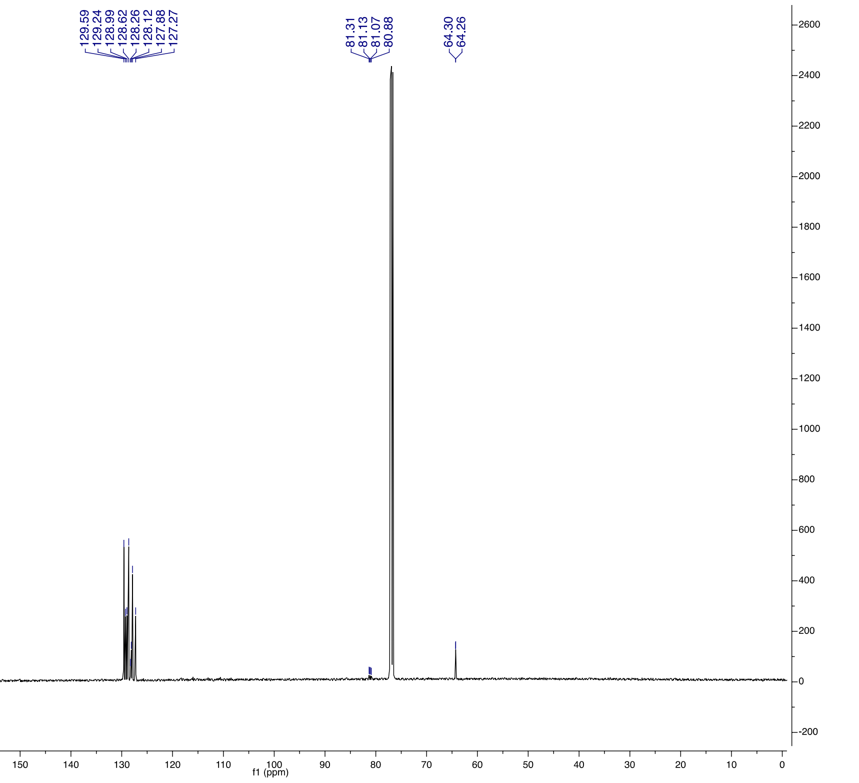

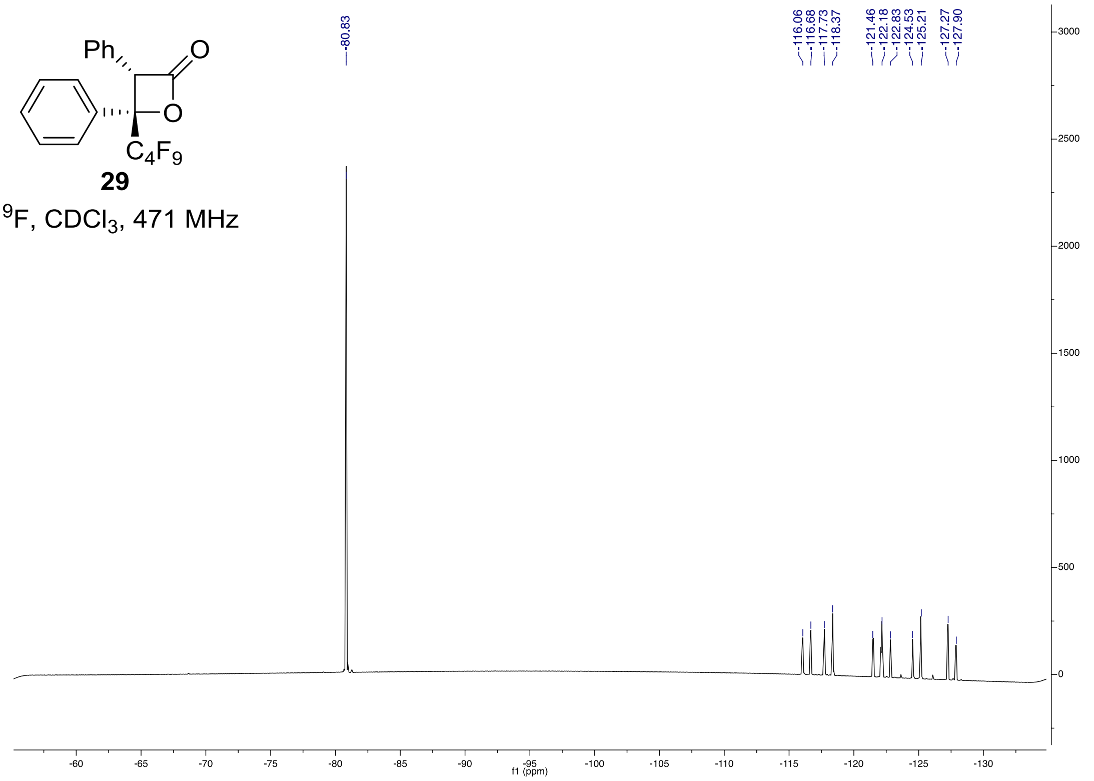

29

^{19}F, $CDCl_3$, 471 MHz

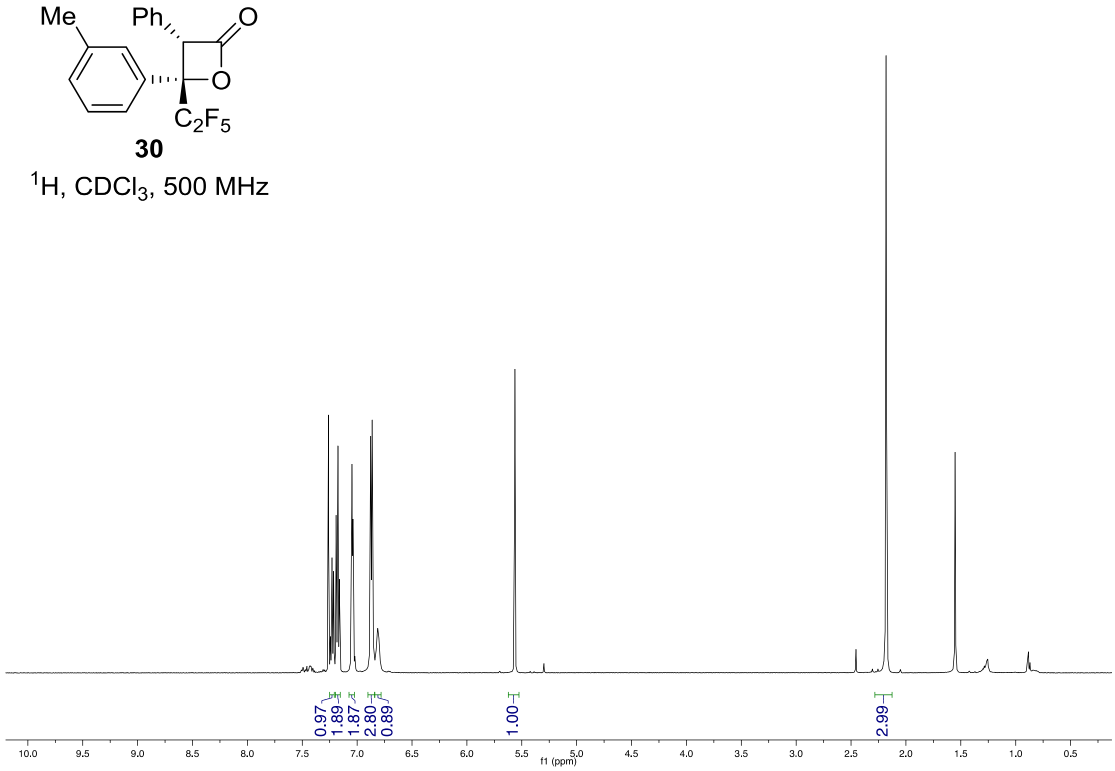

30

^{1}H, $CDCl_3$, 500 MHz

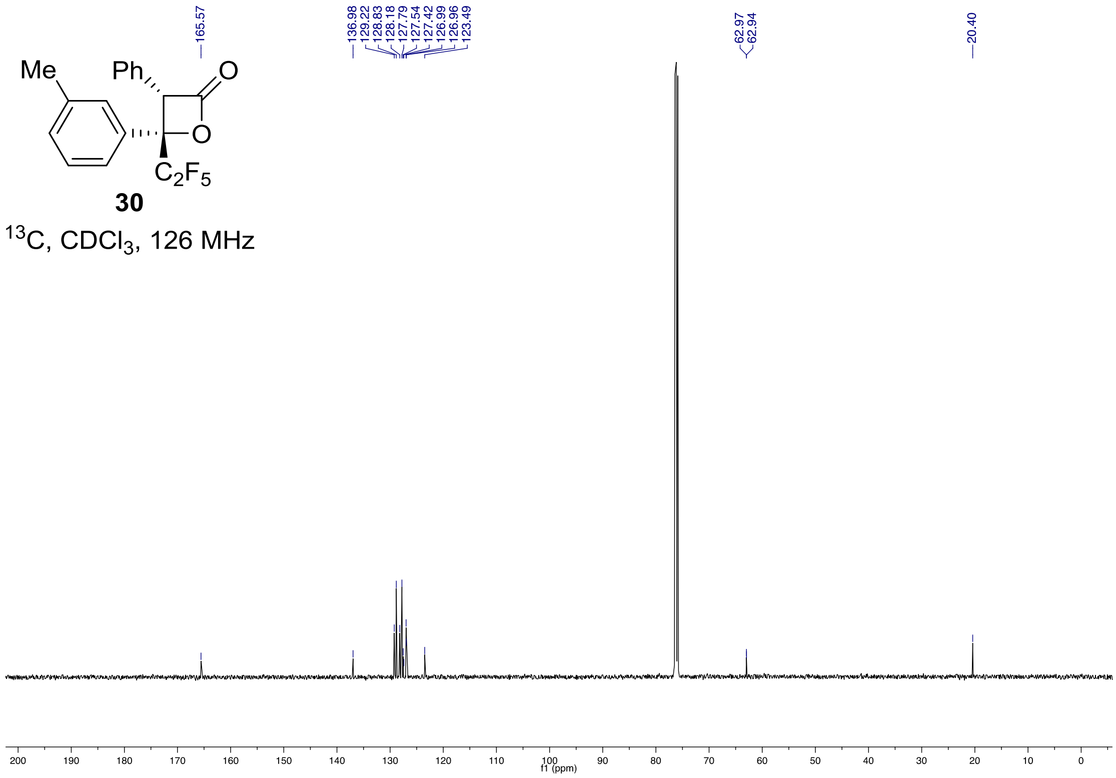
Me
Ph
O
O
C_2F_5
30
^{13}C, $CDCl_3$, 126 MHz
165.57
136.98
129.22
128.83
128.18
127.79
127.54
127.42
126.99
126.96
123.49
62.97
62.94
20.40
f1 (ppm)

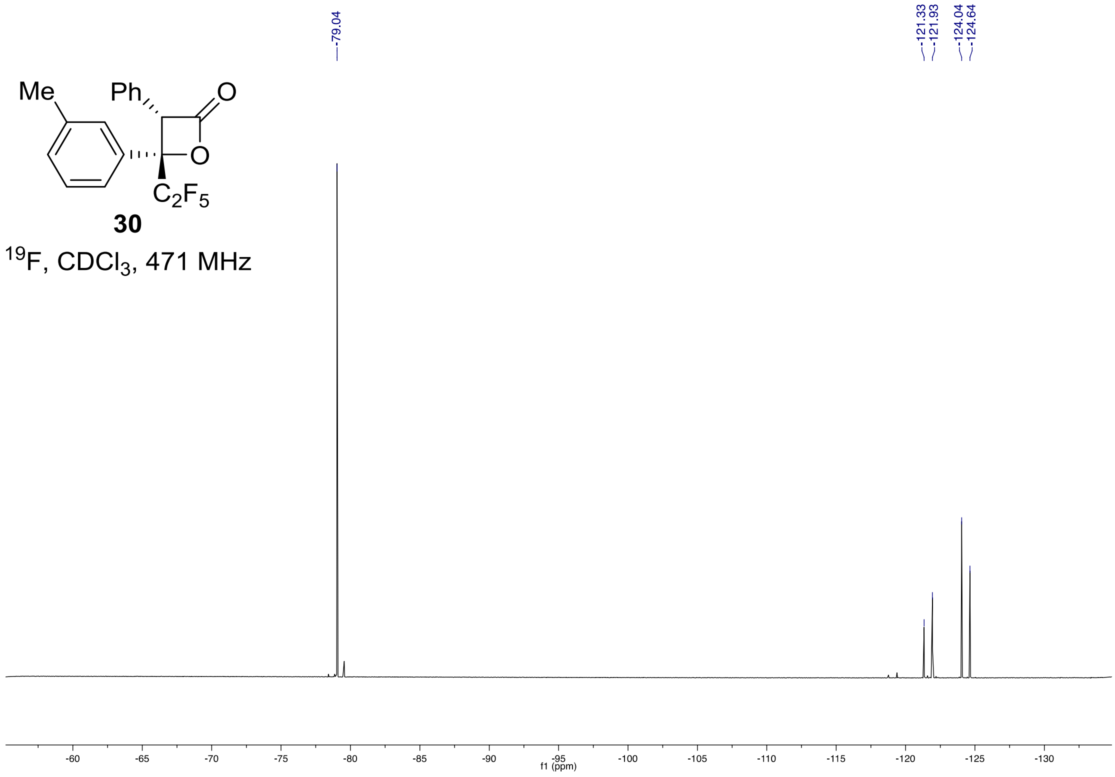
Me
Ph
O
O
C_2F_5
30
^{19}F, $CDCl_3$, 471 MHz
-79.04
-121.33
-121.93
-124.04
-124.64
f1 (ppm)

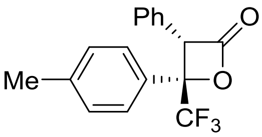

32

^{1}H, $CDCl_3$, 500 MHz

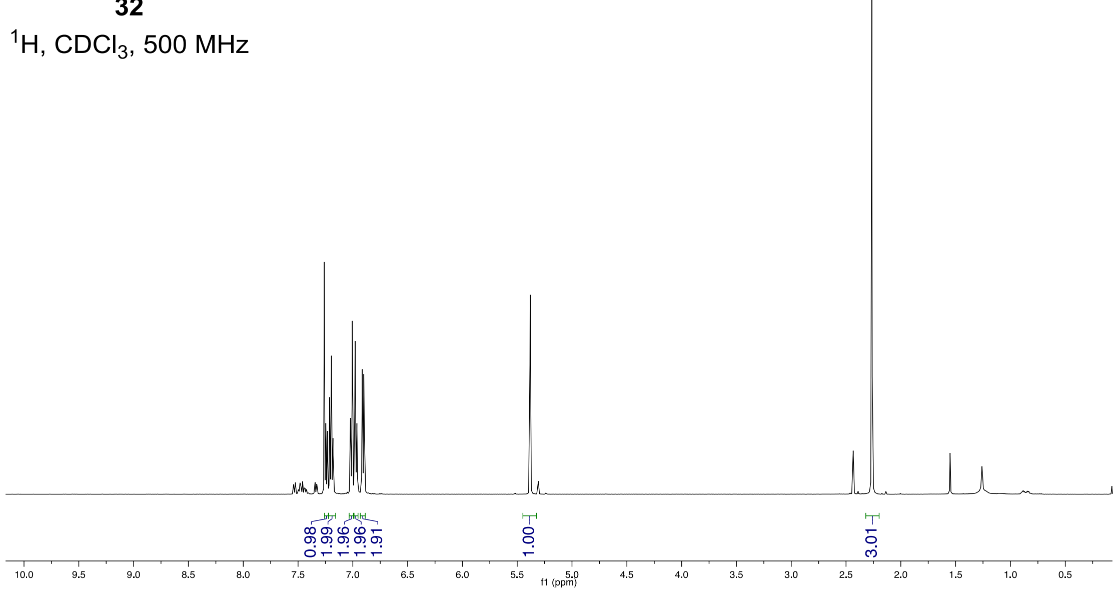

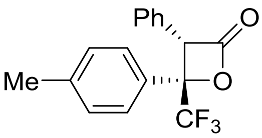

32

^{13}C, $CDCl_3$, 126 MHz

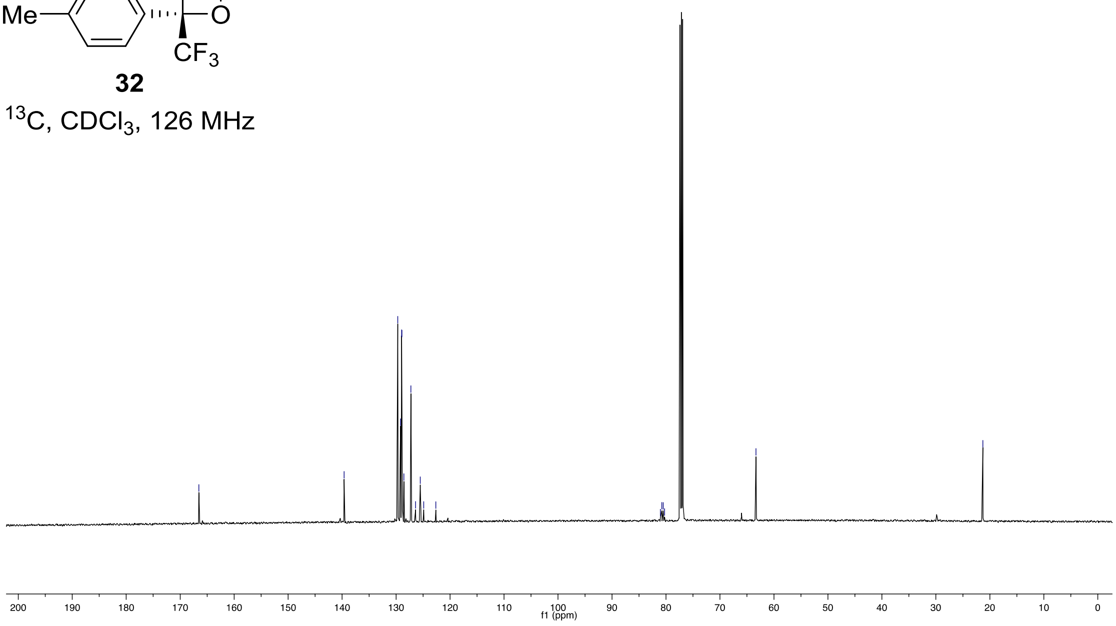

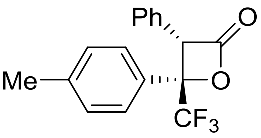

32

^{19}F, $CDCl_3$, 471 MHz

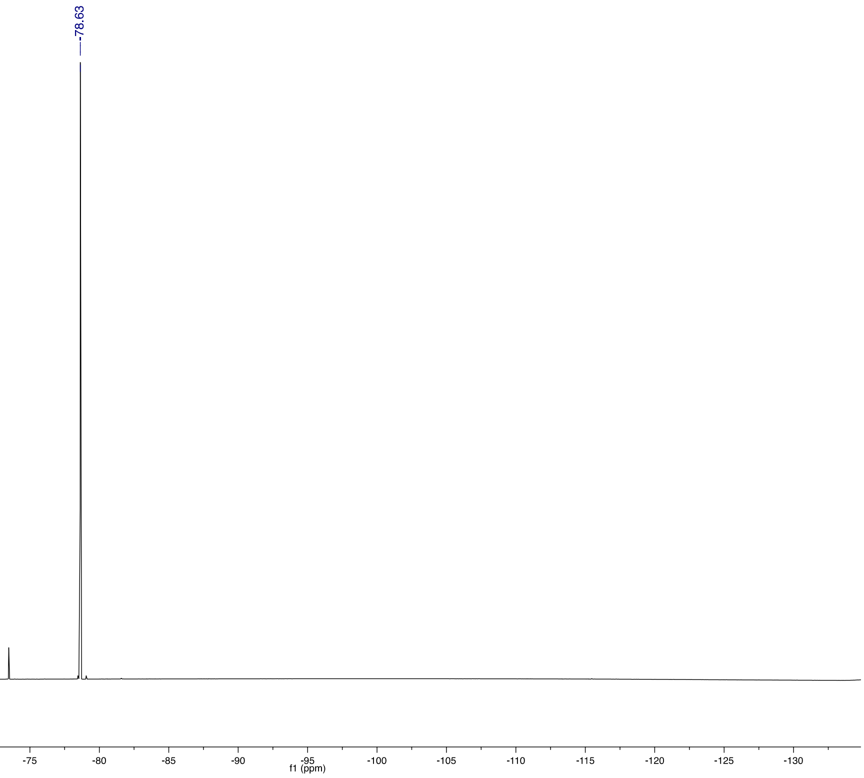

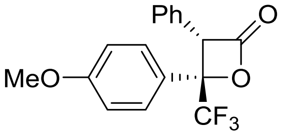

33

^{1}H, $CDCl_3$, 500 MHz

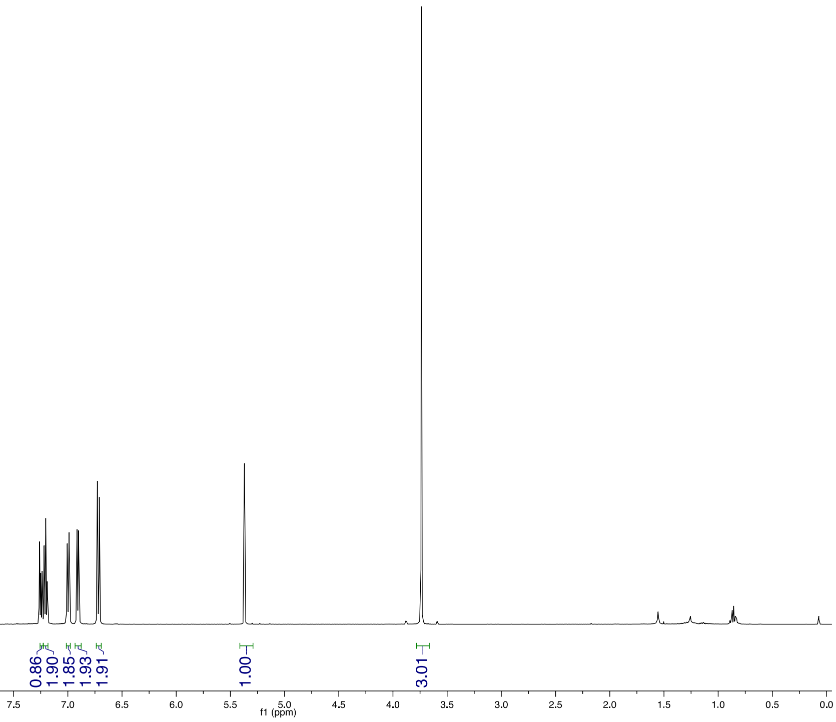

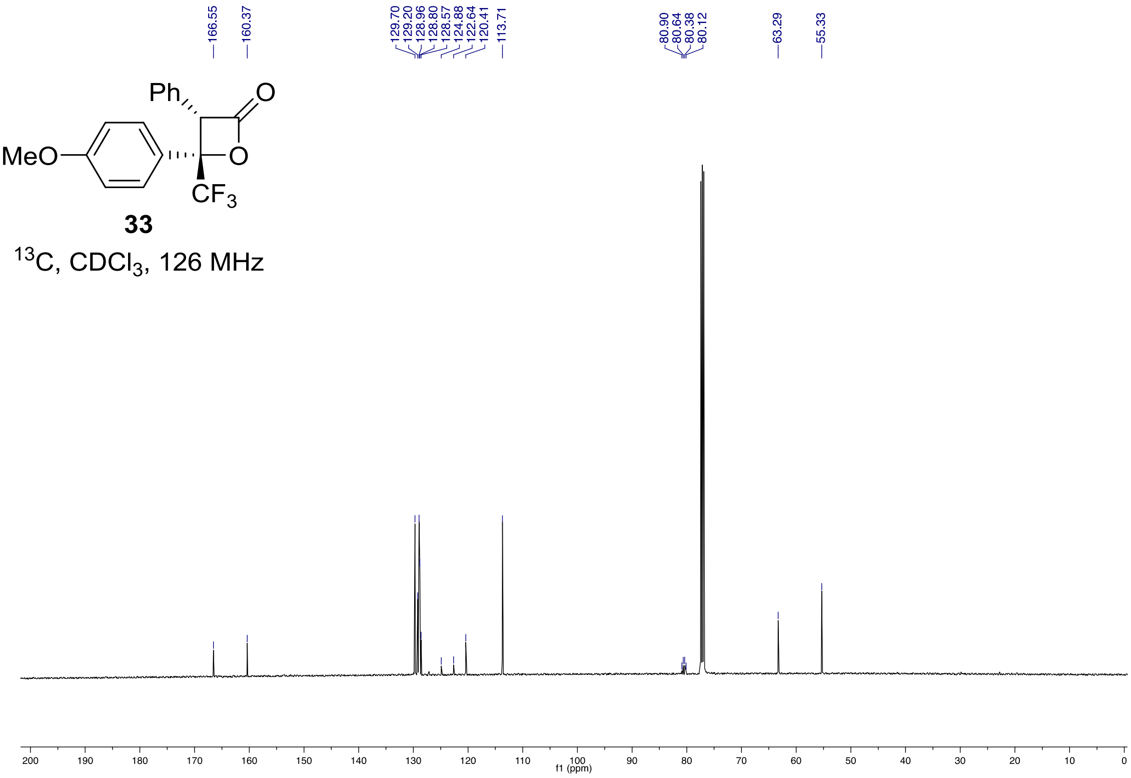
Ph
O
MeO
O
CF3
33
13C, CDCl3, 126 MHz
166.55
160.37
129.70
129.20
128.96
128.80
128.57
124.88
122.64
120.41
113.71
80.90
80.64
80.38
80.12
63.29
55.33
f1 (ppm)

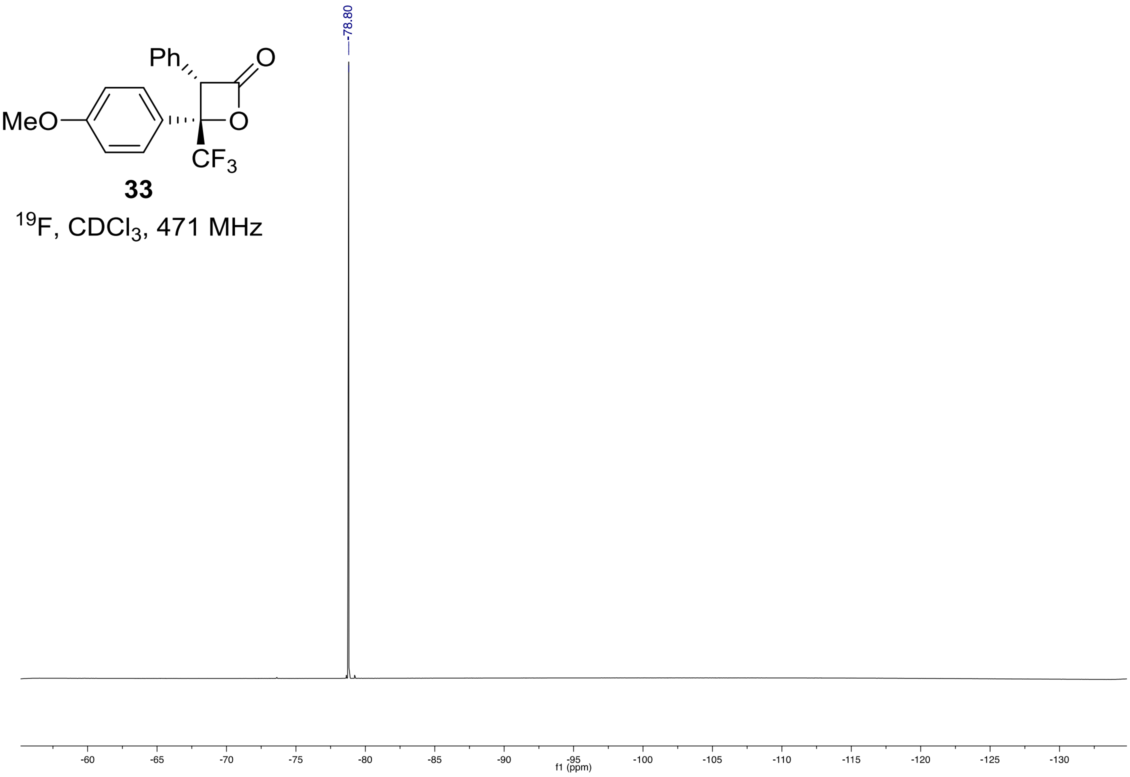
Ph
O
MeO
O
CF3
33
19F, CDCl3, 471 MHz
-78.80
f1 (ppm)

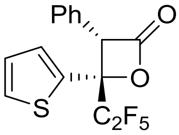

34

^{1}H, $CDCl_3$, 500 MHz

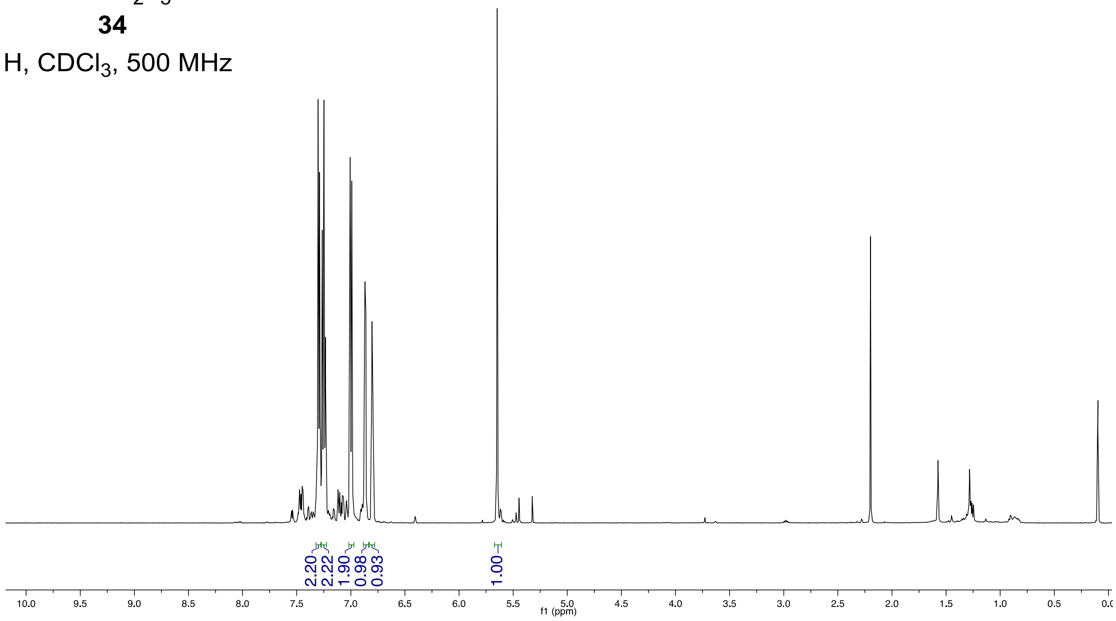

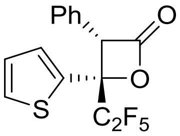

34

^{13}C, $CDCl_3$, 126 MHz

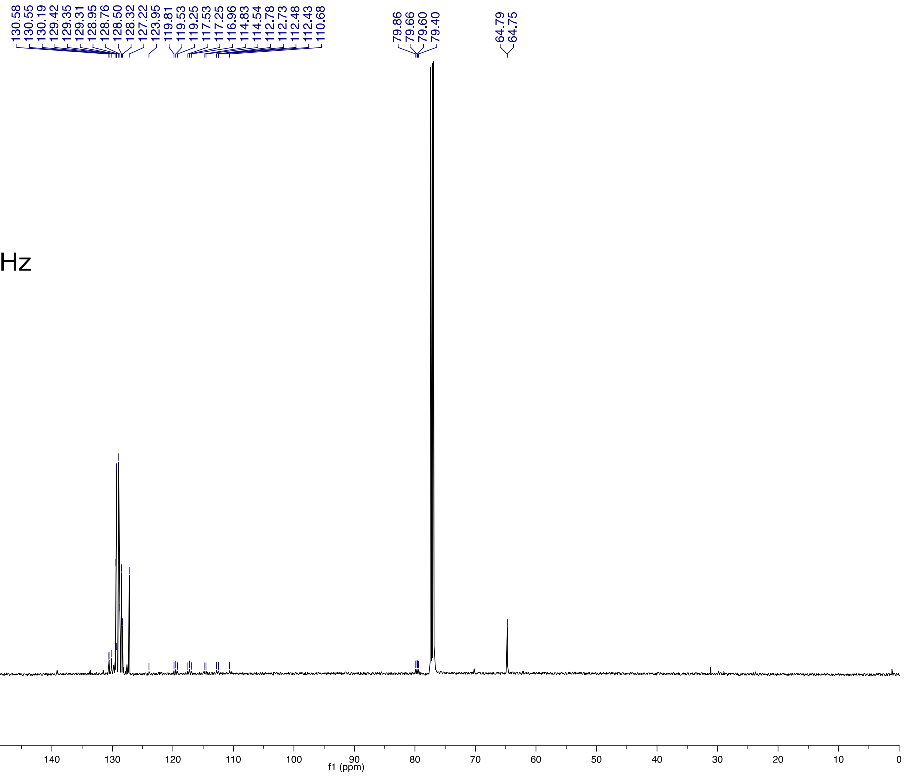

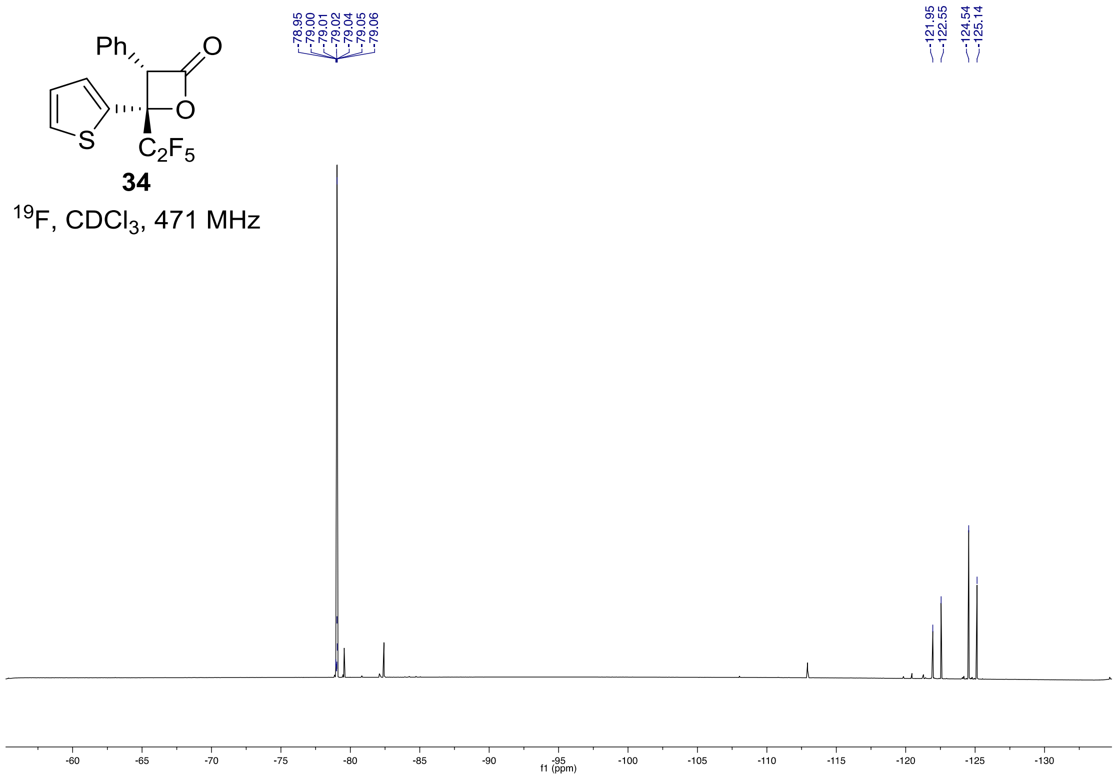
Ph
O
O
S
C_2F_5
34
^{19}F, $CDCl_3$, 471 MHz
-78.95
-79.00
-79.01
-79.02
-79.04
-79.05
-79.06
-121.95
-122.55
-124.54
-125.14
f1 (ppm)

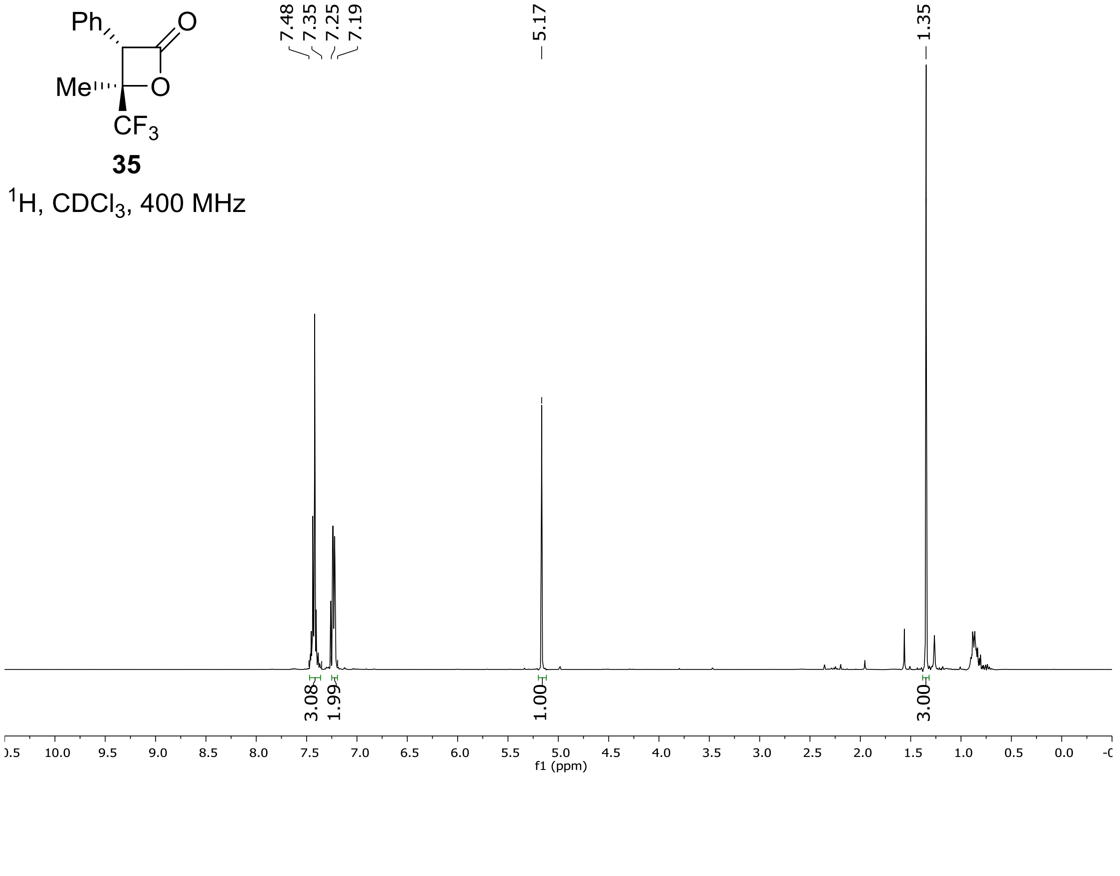
Ph
O
Me
O
CF_3
35
^1H, $CDCl_3$, 400 MHz
7.48
7.35
7.25
7.19
5.17
1.35
3.08
1.99
1.00
3.00
f1 (ppm)

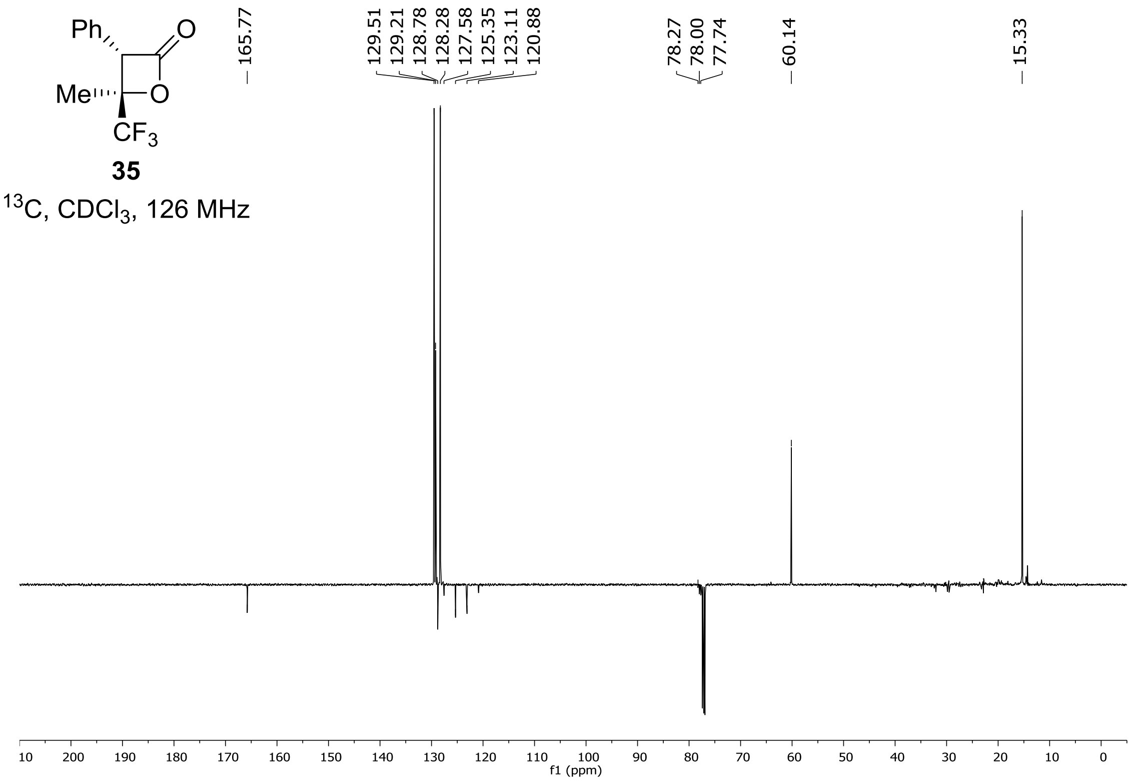

Ph
O
Me
O
CF3
35
13C, CDCl3, 126 MHz
165.77
129.51
129.21
128.78
128.28
127.58
125.35
123.11
120.88
78.27
78.00
77.74
60.14
15.33
10 200 190 180 170 160 150 140 130 120 110 100 90 80 70 60 50 40 30 20 10 0
f1 (ppm)

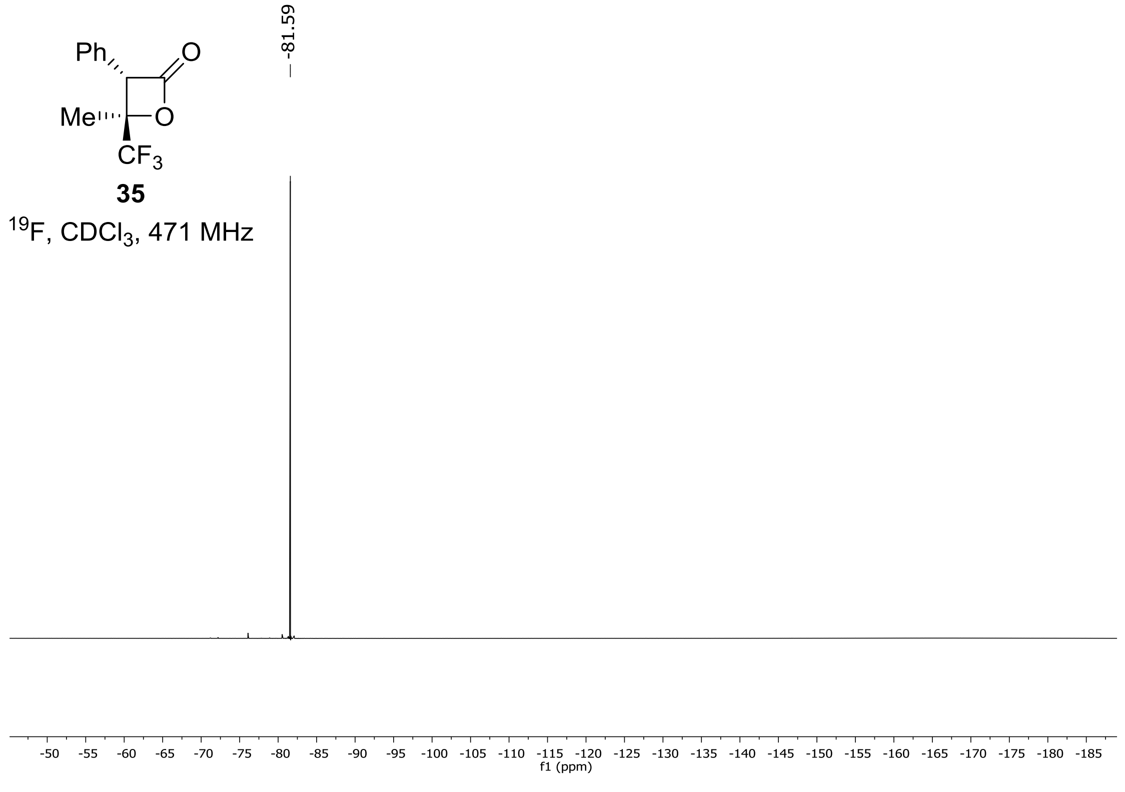

Ph
O
Me
O
CF3
35
19F, CDCl3, 471 MHz
-81.59
-50 -55 -60 -65 -70 -75 -80 -85 -90 -95 -100 -105 -110 -115 -120 -125 -130 -135 -140 -145 -150 -155 -160 -165 -170 -175 -180 -185
f1 (ppm)

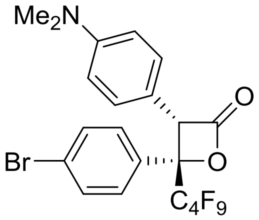

36

^1H, $CDCl_3$, 500 MHz

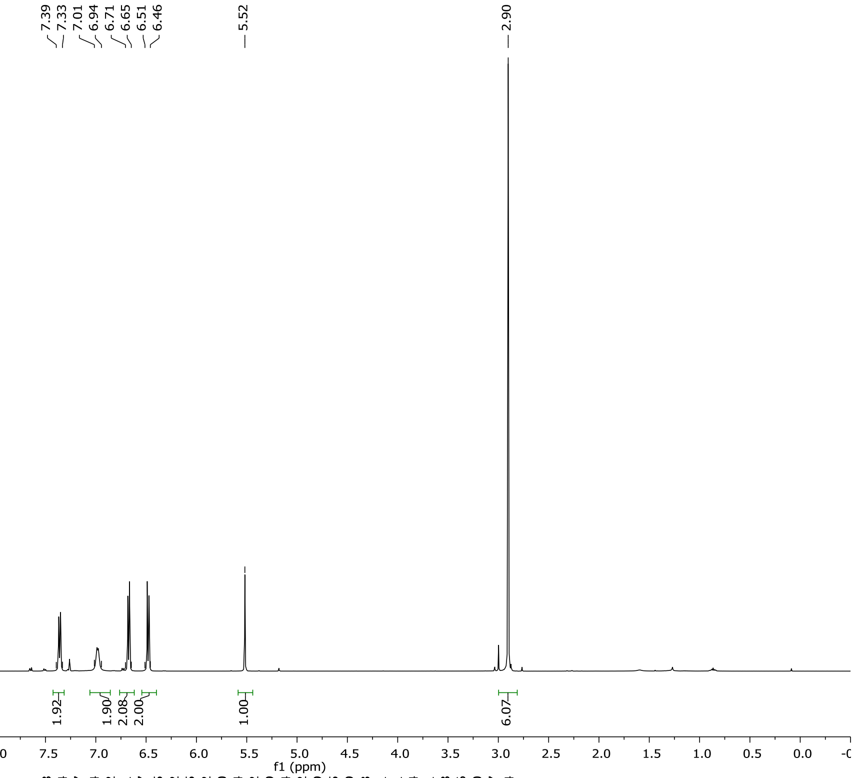

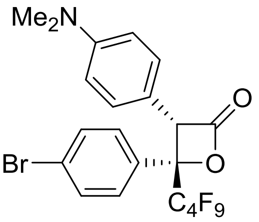

36

^{13}C, $CDCl_3$, 126 MHz

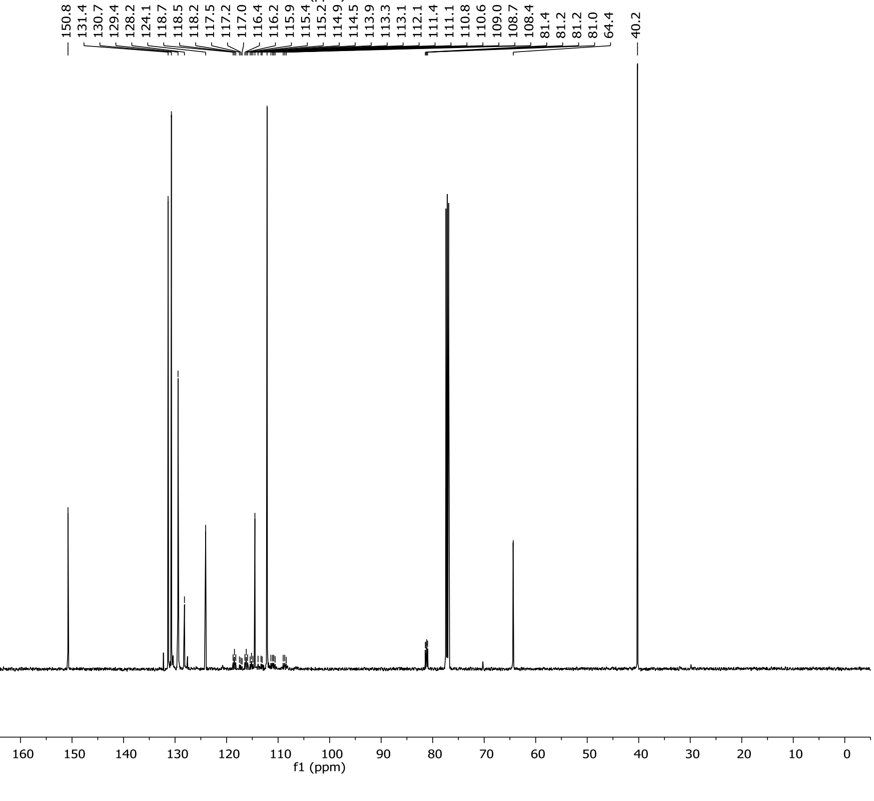

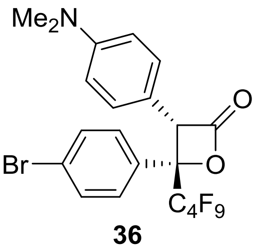

36

^{19}F, $CDCl_3$, 471 MHz

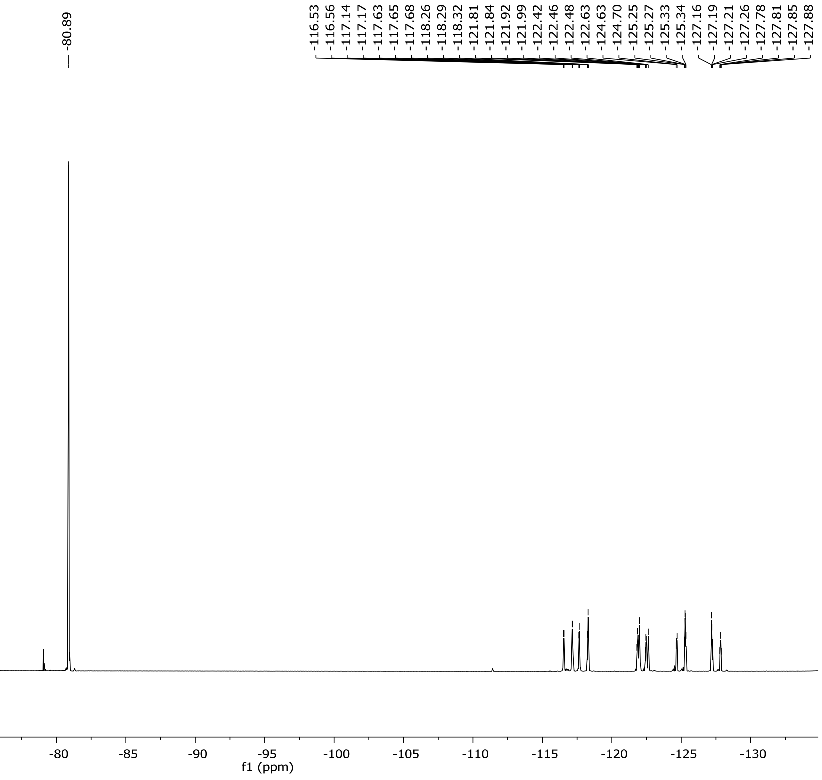

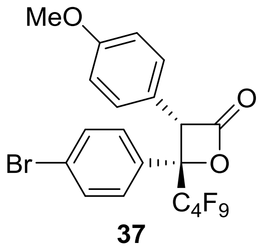

37

^{1}H, $CDCl_3$, 500 MHz

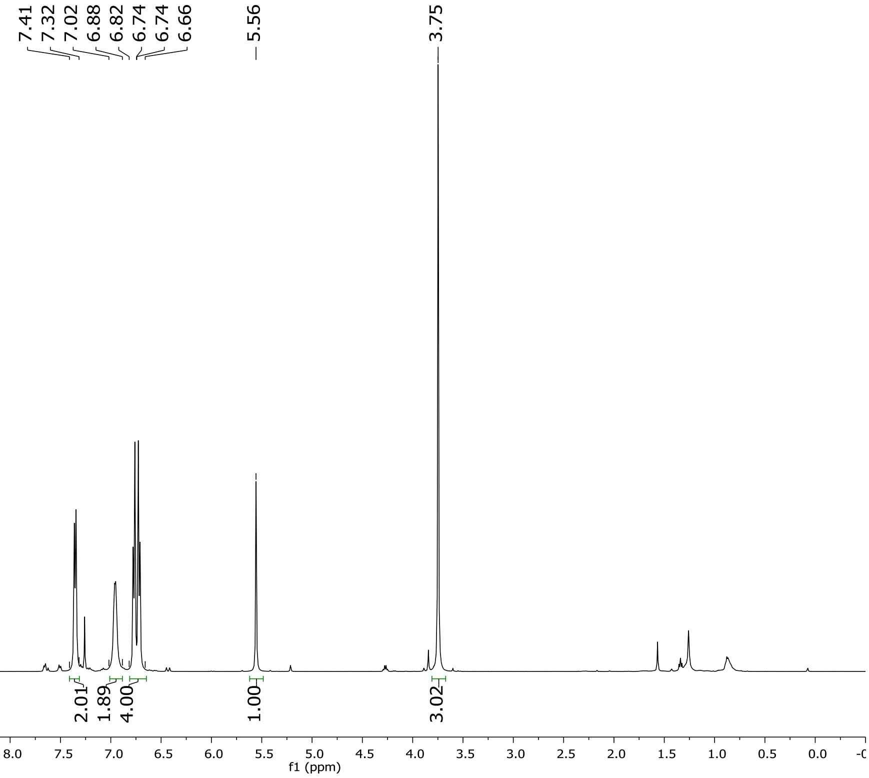

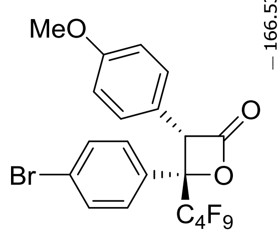

37

^{13}C, $CDCl_3$, 500 MHz

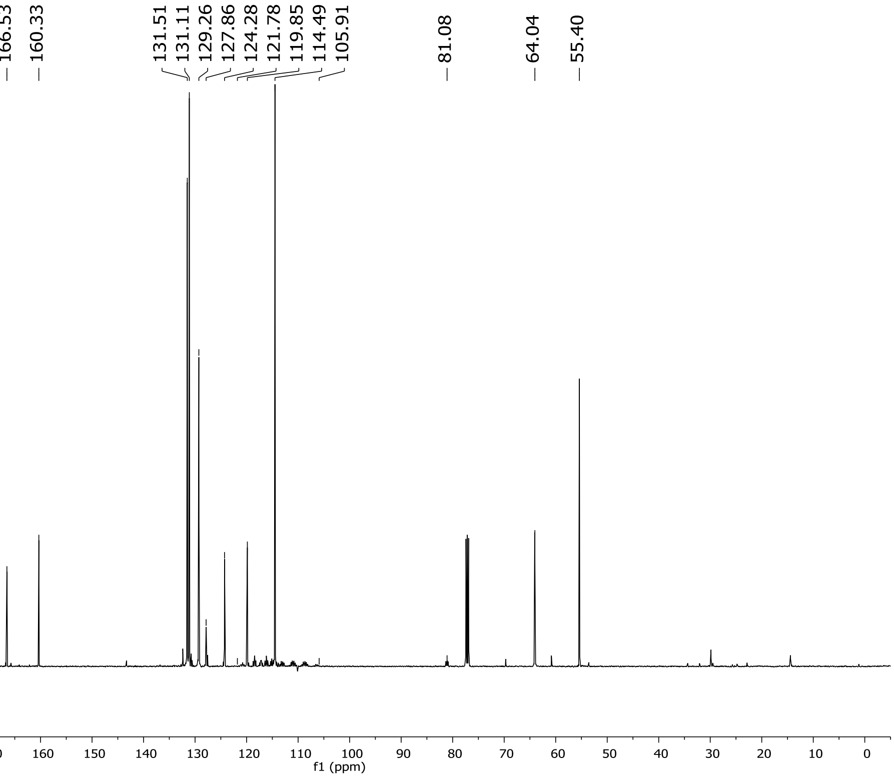

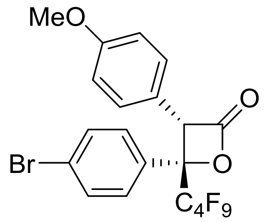

37

^{19}F, $CDCl_3$, 471 MHz

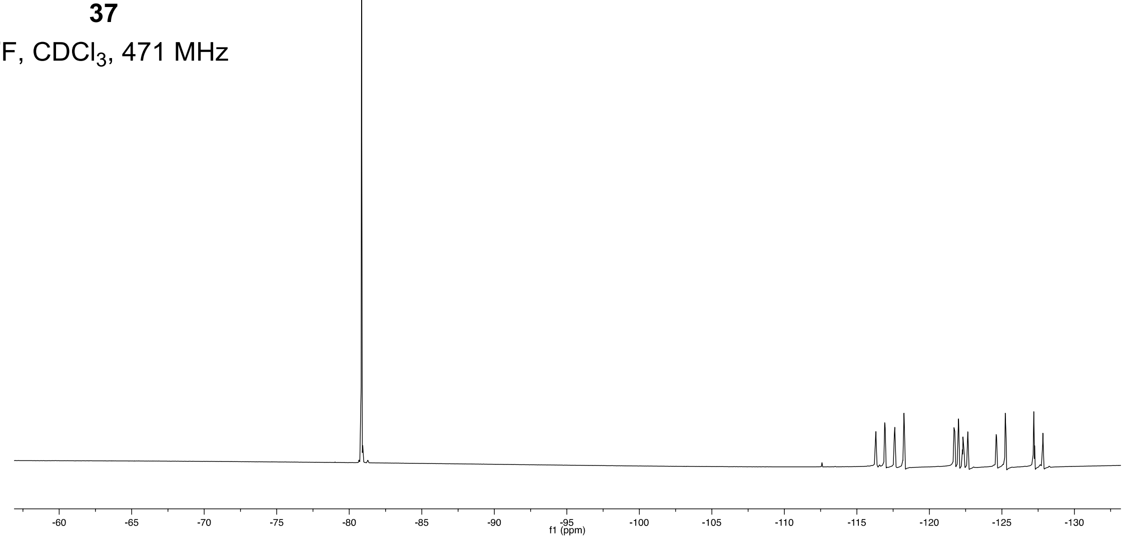

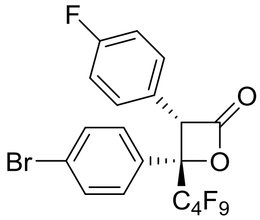

38

^{1}H, $CDCl_3$, 500 MHz

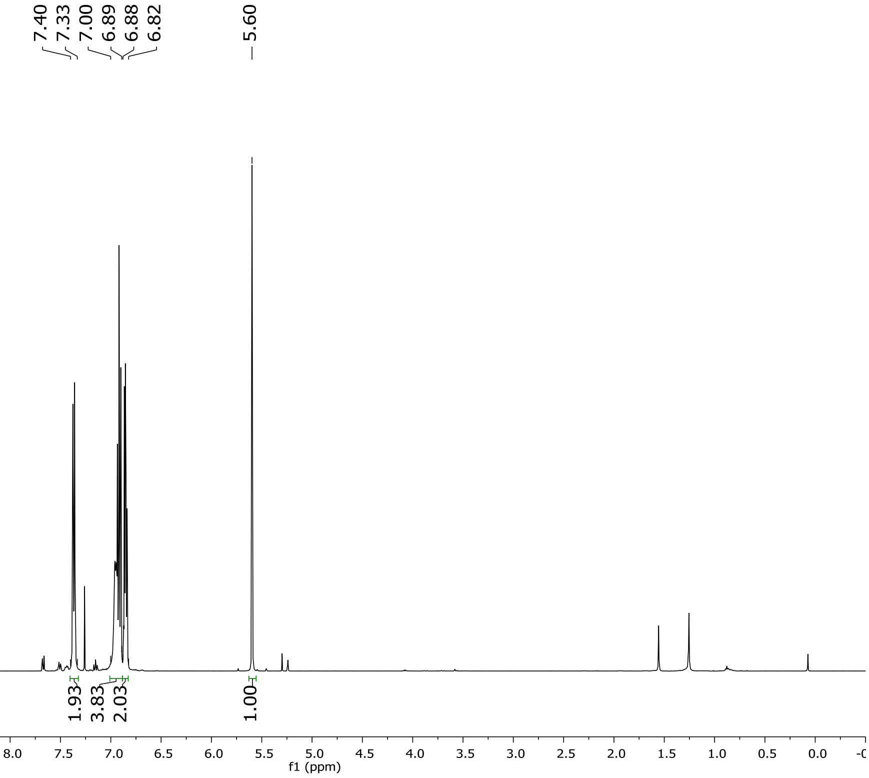

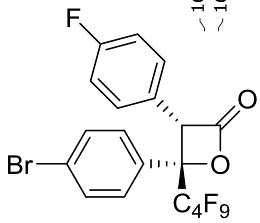

38

^{13}C, $CDCl_3$, 126 MHz

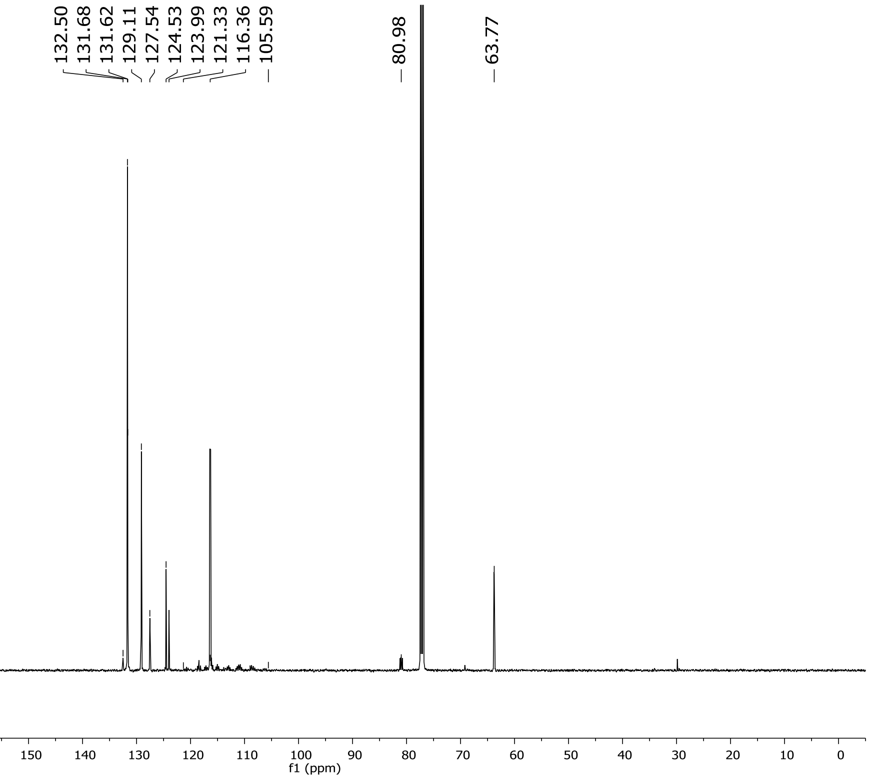

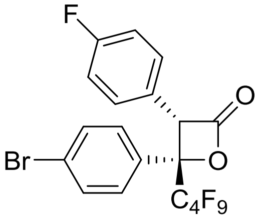

38

^{19}F, $CDCl_3$, 471 MHz

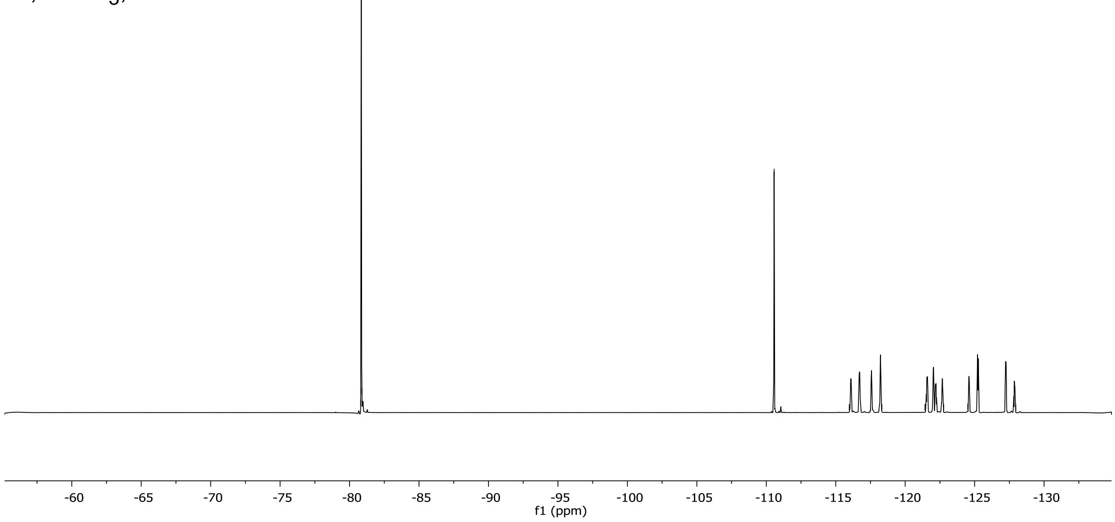

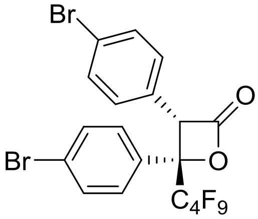

39

^{1}H, $CDCl_3$, 500 MHz

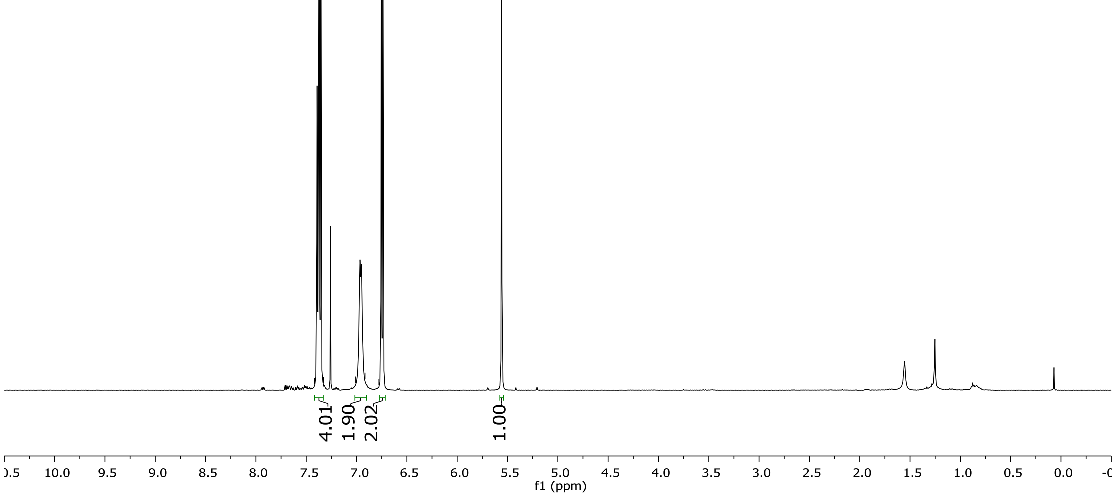

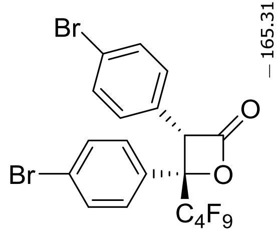

39

^{13}C, $CDCl_3$, 126 MHz

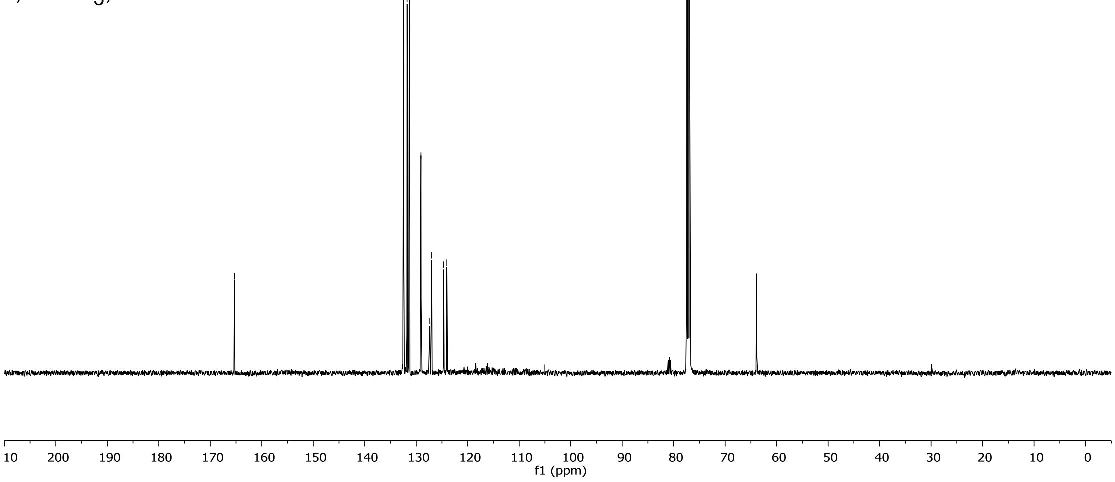

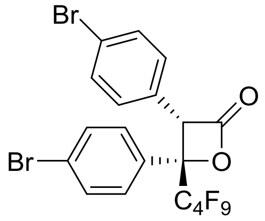

39

^{19}F, $CDCl_3$, 471 MHz

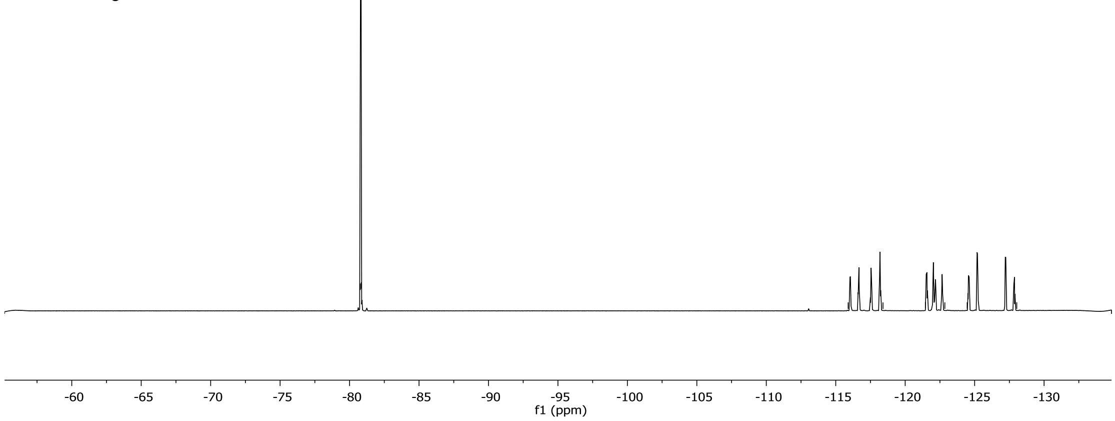

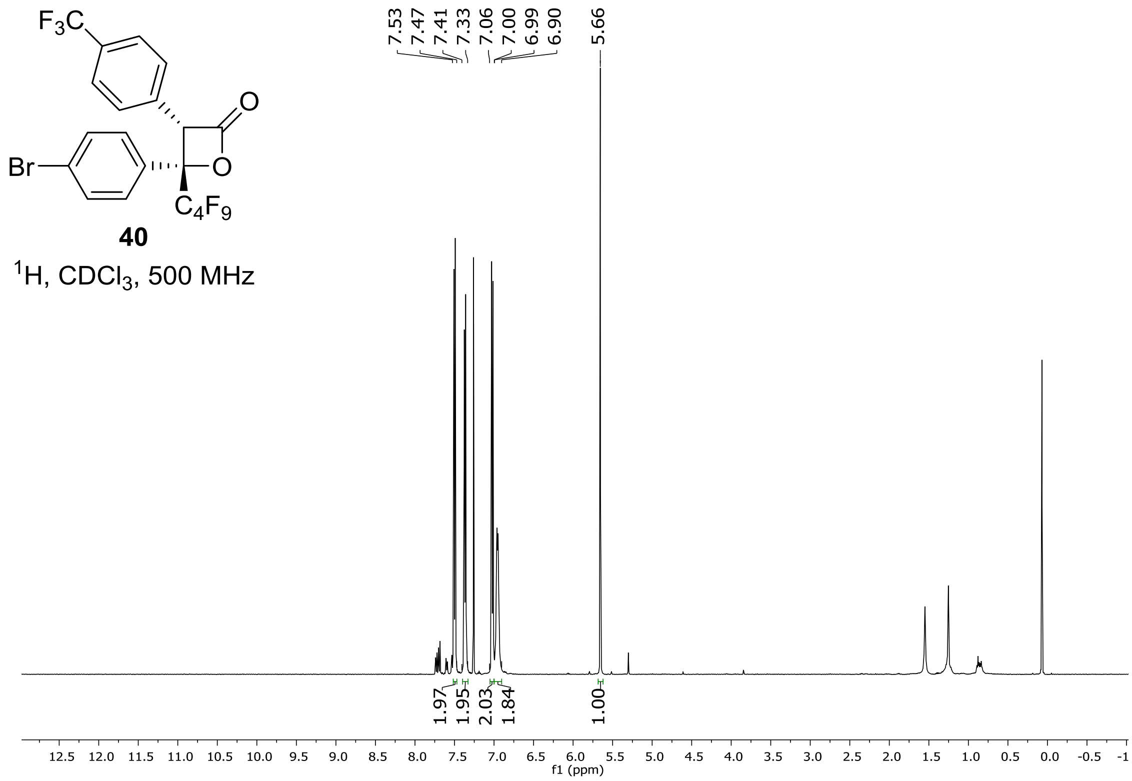
F3C
O
Br
O
C4F9
40
1H, CDCl3, 500 MHz
7.53
7.47
7.41
7.33
7.06
7.00
6.99
6.90
5.66
1.97
1.95
2.03
1.84
1.00
f1 (ppm)

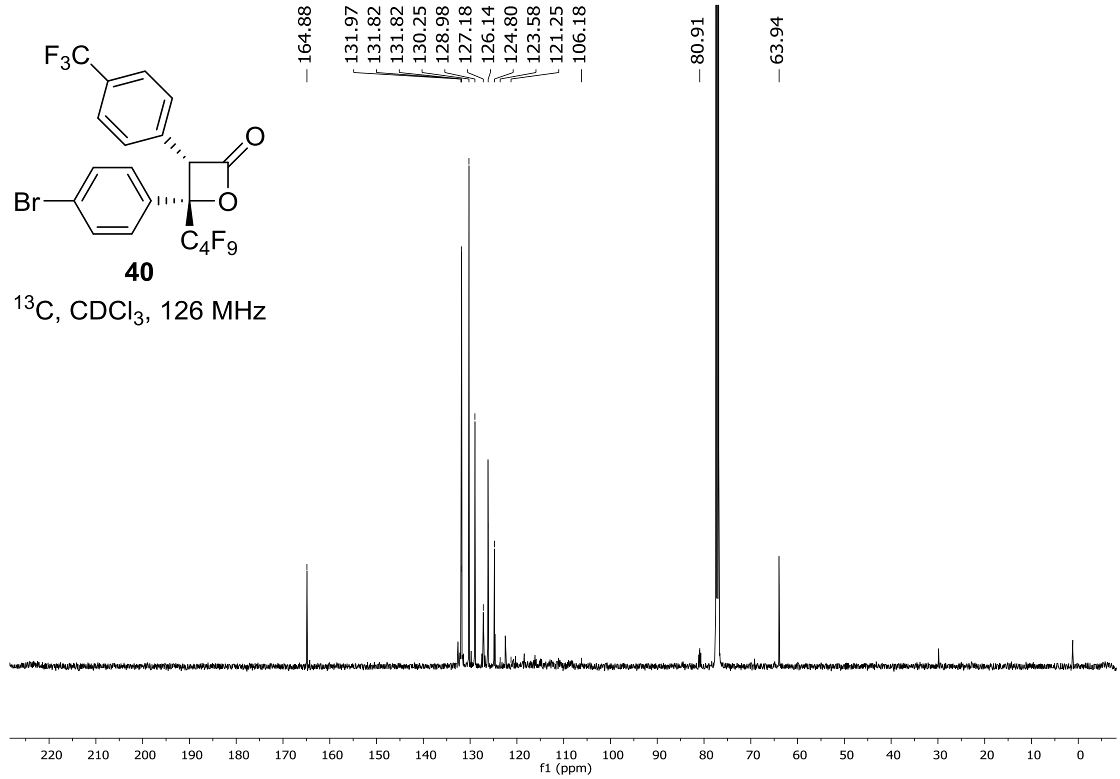
F3C
O
Br
O
C4F9
40
13C, CDCl3, 126 MHz
164.88
131.97
131.82
131.82
130.25
128.98
127.18
126.14
124.80
123.58
121.25
106.18
80.91
63.94
f1 (ppm)

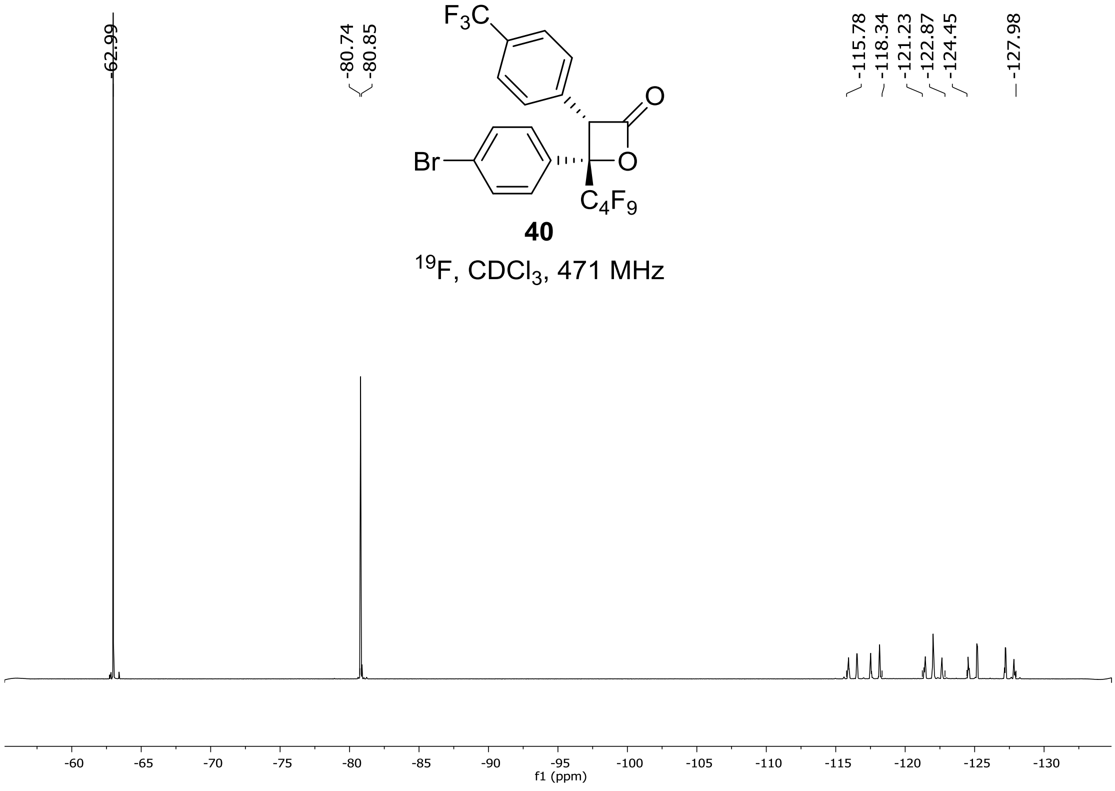
-62.99
-80.74
-80.85
F_3C
O
Br
O
C_4F_9
40
^{19}F, $CDCl_3$, 471 MHz
-115.78
-118.34
-121.23
-122.87
-124.45
-127.98
f1 (ppm)

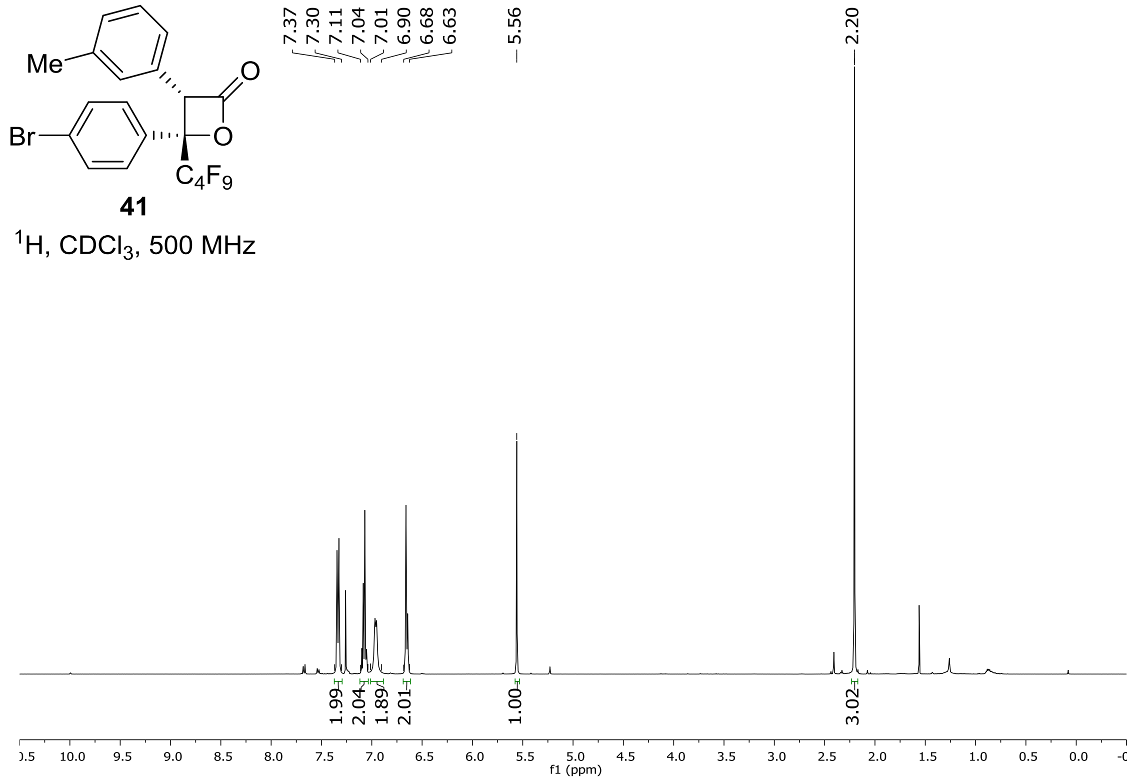
Me
O
Br
O
C_4F_9
41
^1H, $CDCl_3$, 500 MHz
7.37
7.30
7.11
7.04
7.01
6.90
6.68
6.63
5.56
2.20
1.99
2.04
1.89
2.01
1.00
3.02
f1 (ppm)

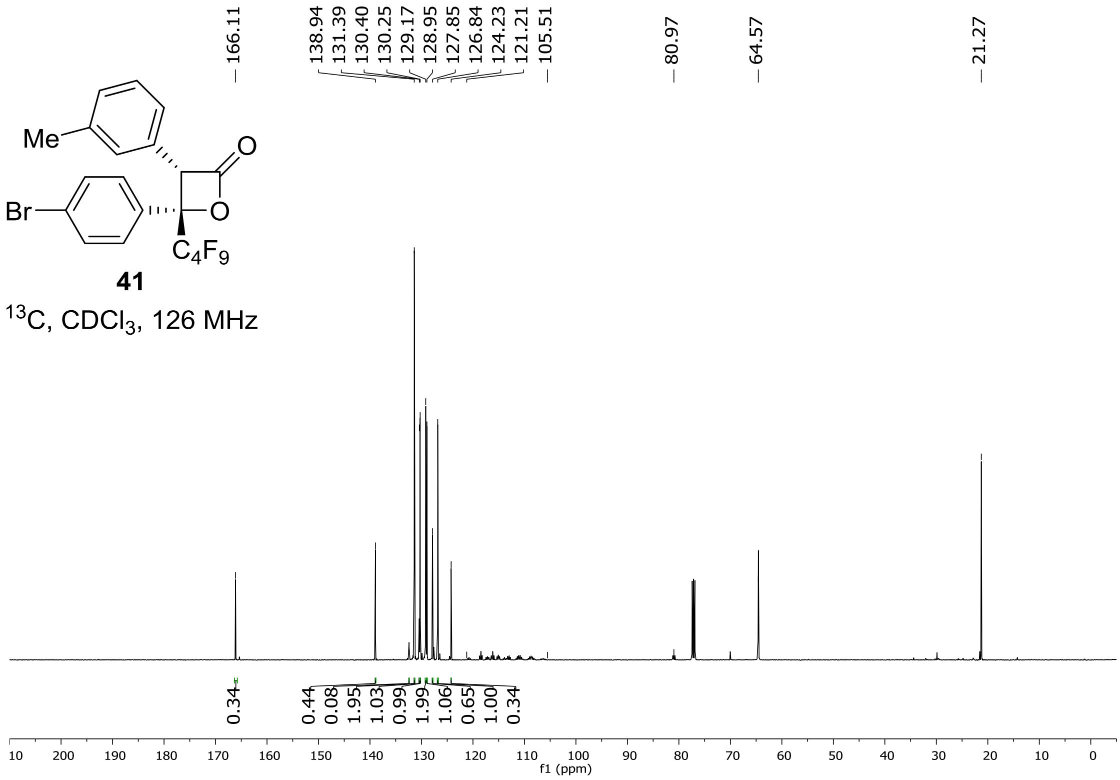

166.11
138.94
131.39
130.40
130.25
129.17
128.95
127.85
126.84
124.23
121.21
105.51
80.97
64.57
21.27
Me
O
Br
O
C_4F_9
41
^{13}C, $CDCl_3$, 126 MHz
0.34
0.44
0.08
1.95
1.03
0.99
1.99
1.06
0.65
1.00
0.34
f1 (ppm)

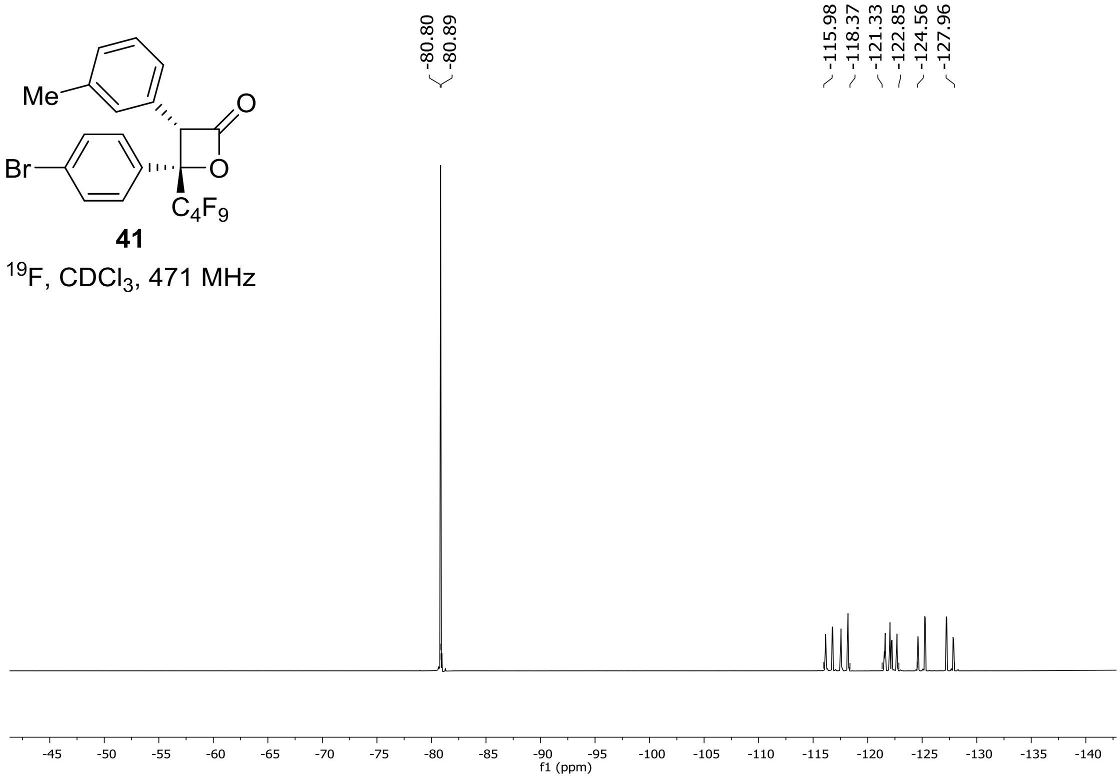

-80.80
-80.89
-115.98
-118.37
-121.33
-122.85
-124.56
-127.96
Me
O
Br
O
C_4F_9
41
^{19}F, $CDCl_3$, 471 MHz
f1 (ppm)

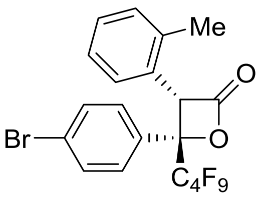

42

^1H, $CDCl_3$, 500 MHz

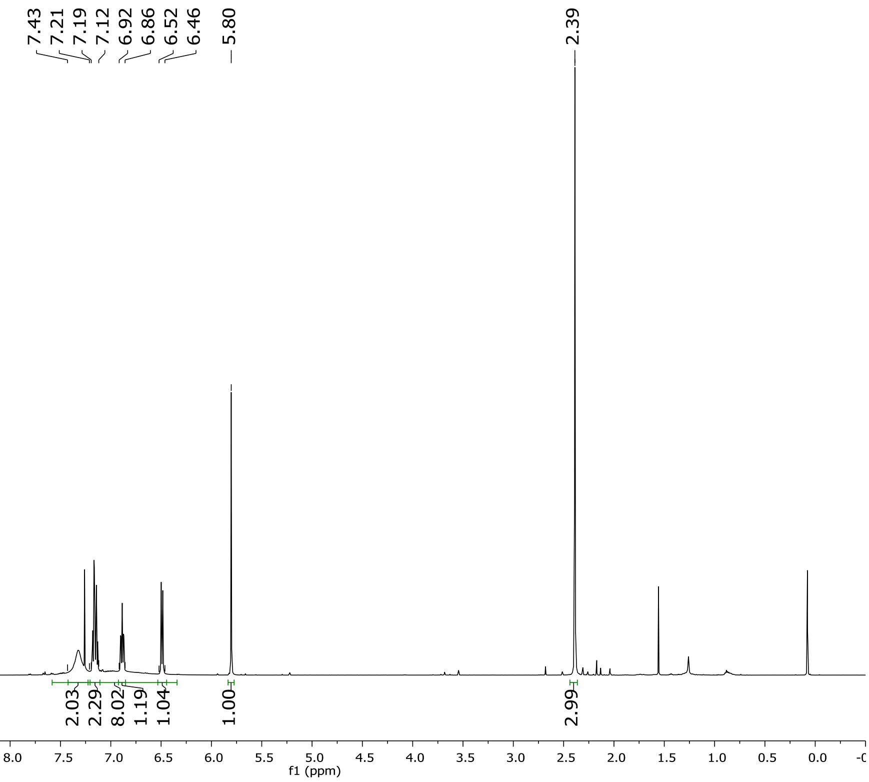

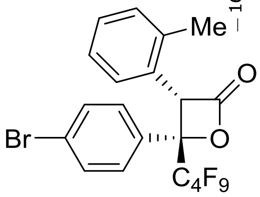

42

^{13}C, $CDCl_3$, 126 MHz

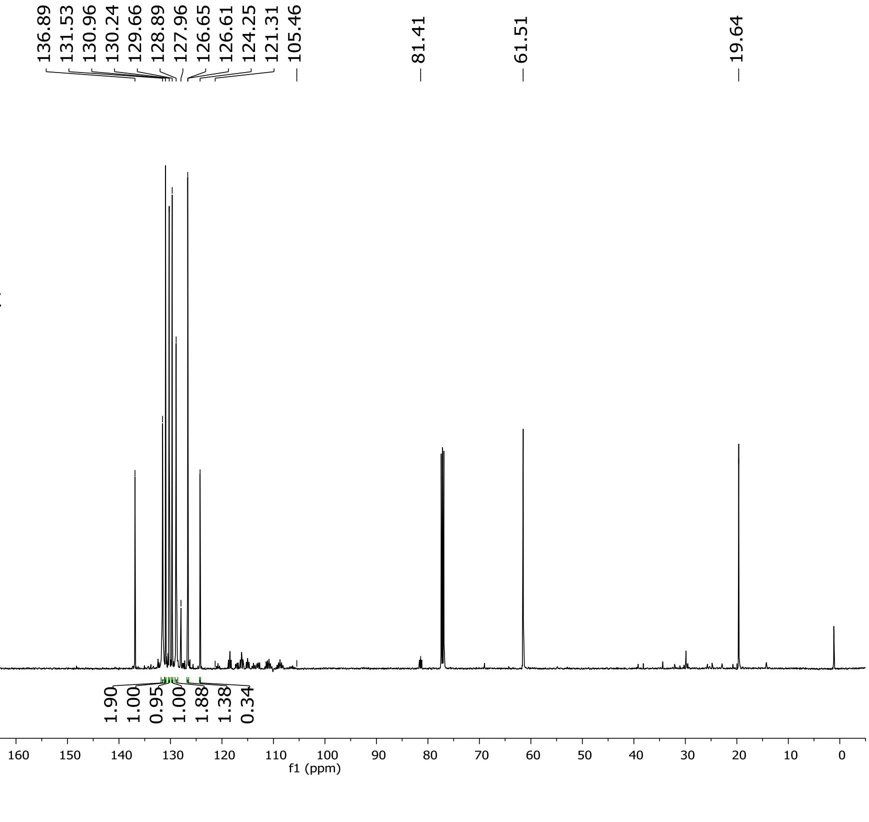

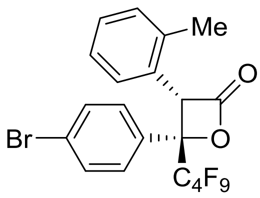

42

^{19}F, $CDCl_3$, 471 MHz

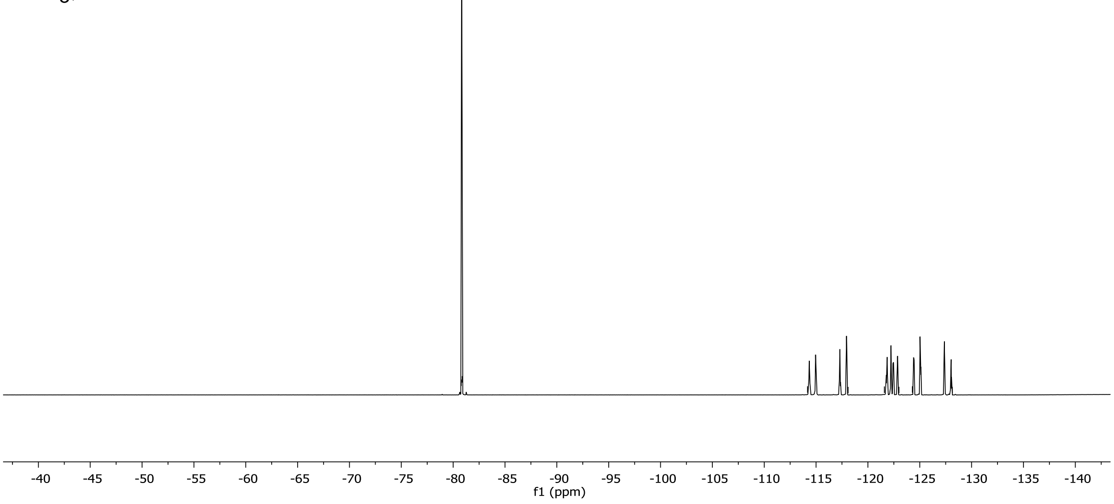

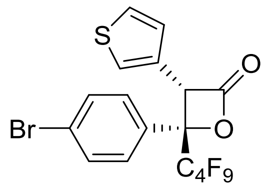

43

^{1}H, $CDCl_3$, 500 MHz

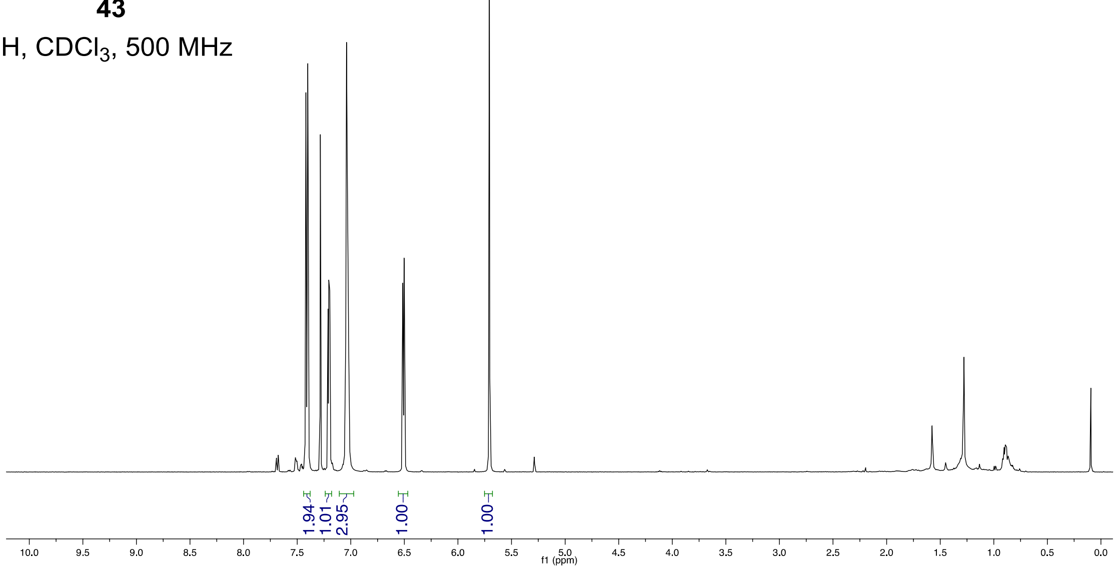

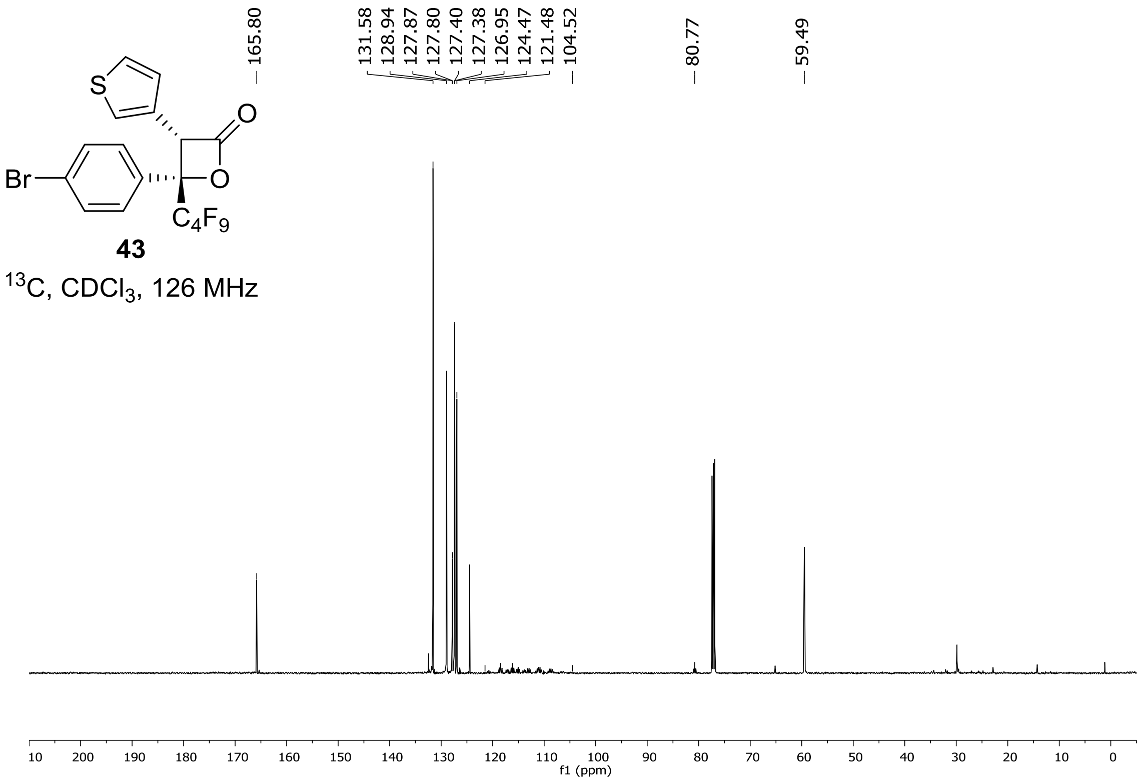
165.80
131.58
128.94
127.87
127.80
127.40
127.38
126.95
124.47
121.48
104.52
80.77
59.49
Br
S
O
O
C_4F_9
43
^{13}C, $CDCl_3$, 126 MHz
f1 (ppm)

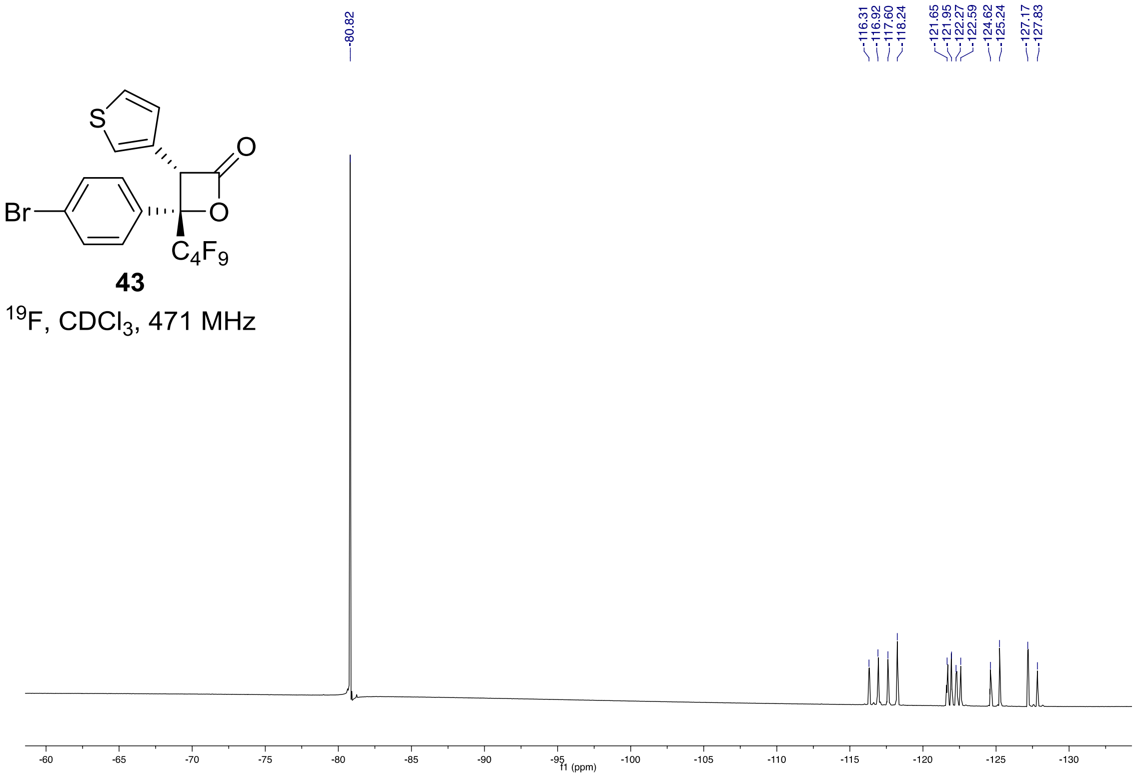
-80.82
-116.31
-116.92
-117.60
-118.24
-121.65
-121.95
-122.27
-122.59
-124.62
-125.24
-127.17
-127.83
Br
S
O
O
C_4F_9
43
^{19}F, $CDCl_3$, 471 MHz
f1 (ppm)

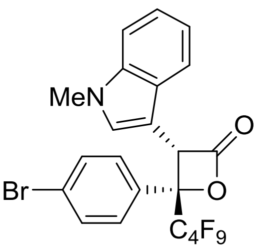

44

^{1}H, $CDCl_3$, 500 MHz

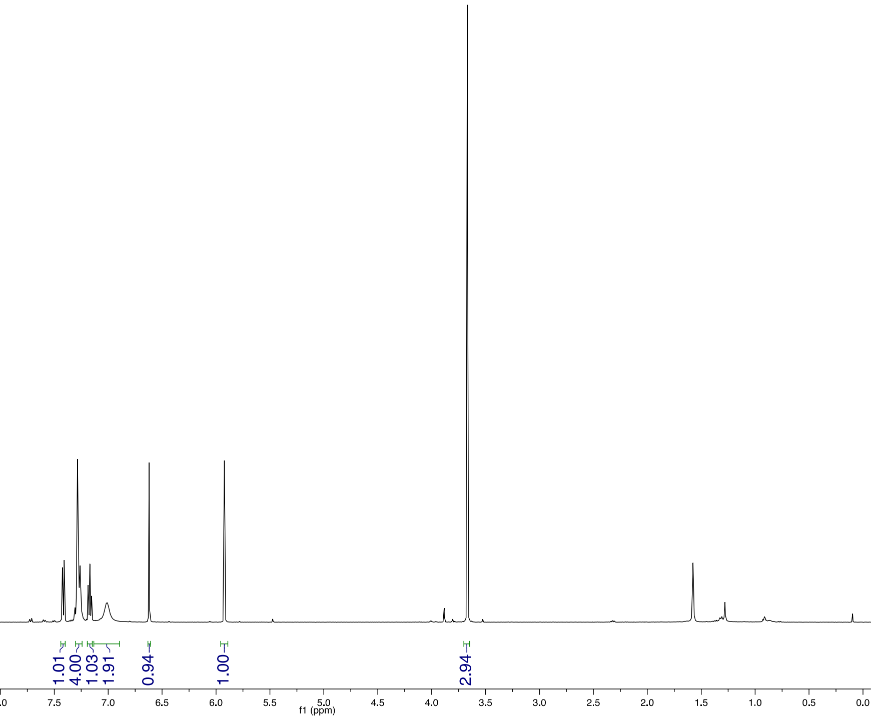

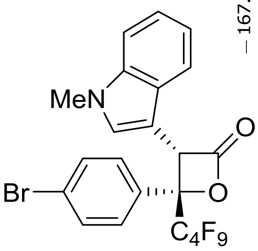

44

^{13}C, $CDCl_3$, 126 MHz

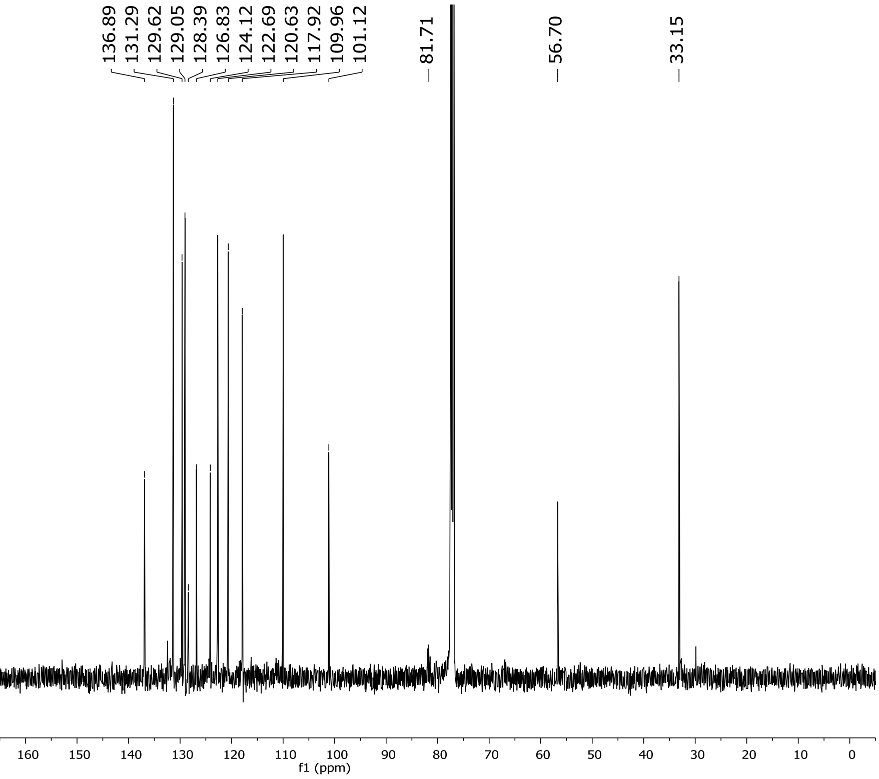

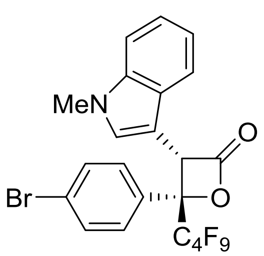

44

^{19}F, $CDCl_3$, 471 MHz

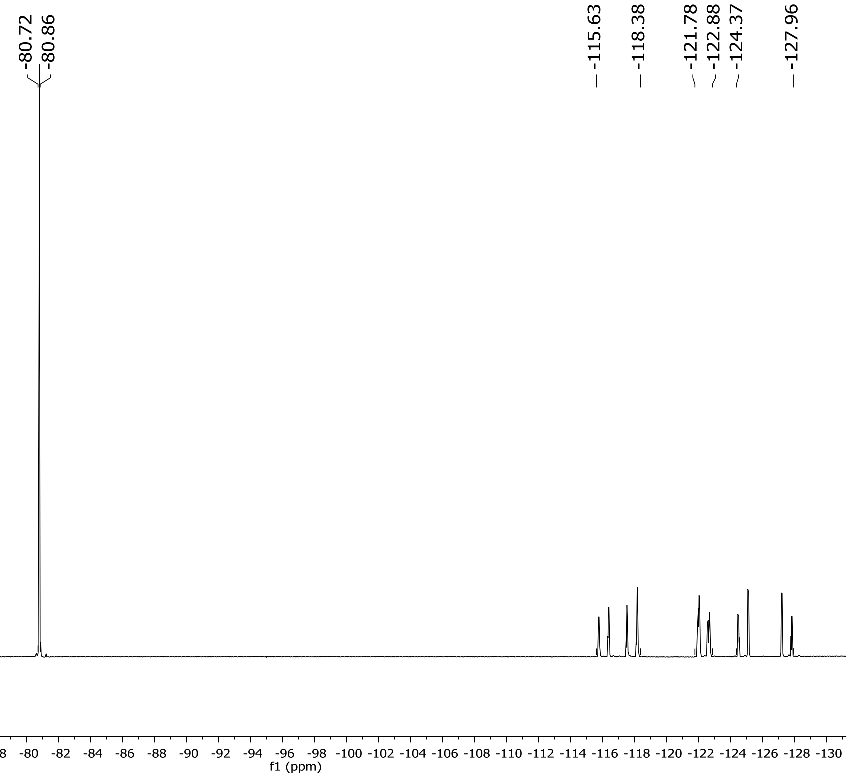

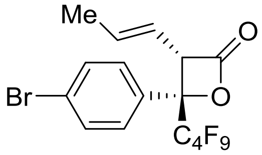

45

^{1}H, $CDCl_3$, 500 MHz

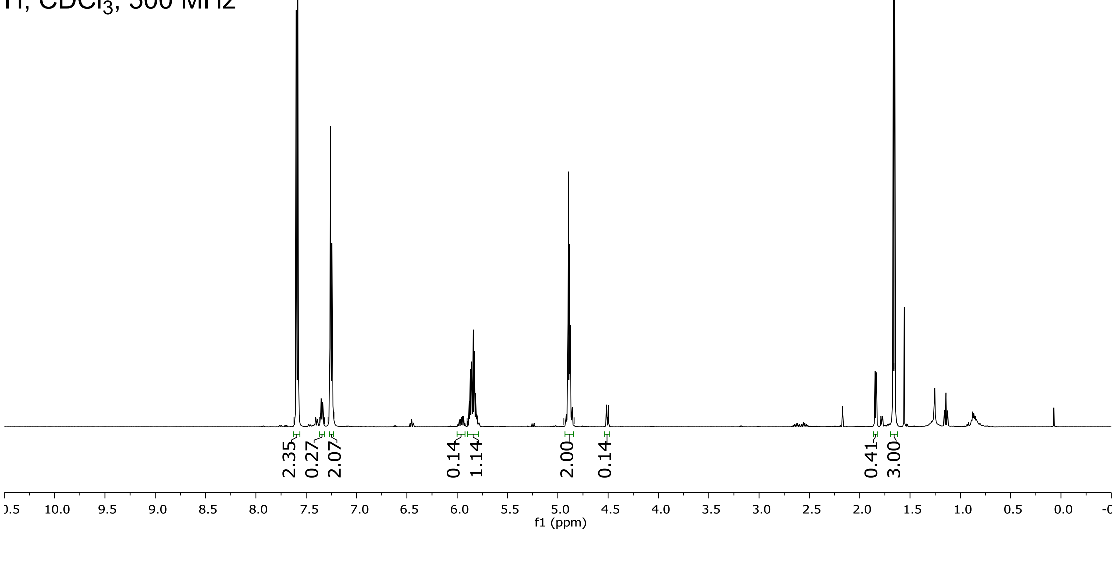

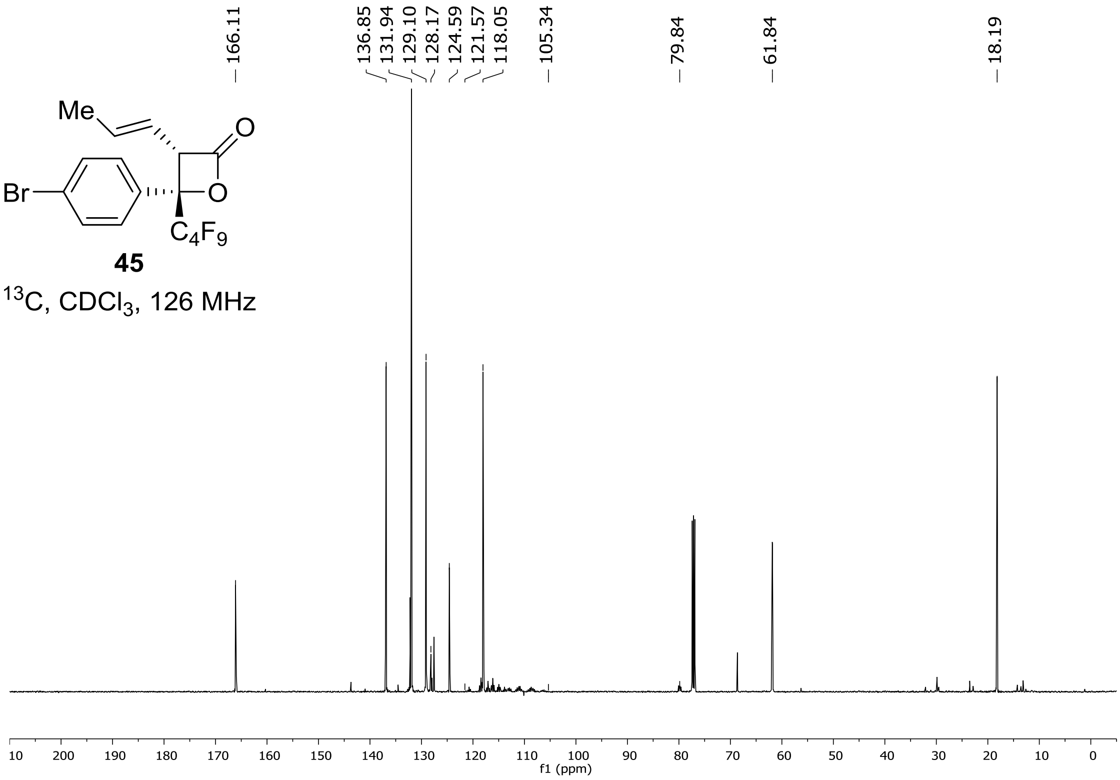
166.11
136.85
131.94
129.10
128.17
124.59
121.57
118.05
105.34
79.84
61.84
18.19
Me
O
Br
O
C_4F_9
45
^{13}C, $CDCl_3$, 126 MHz
f1 (ppm)

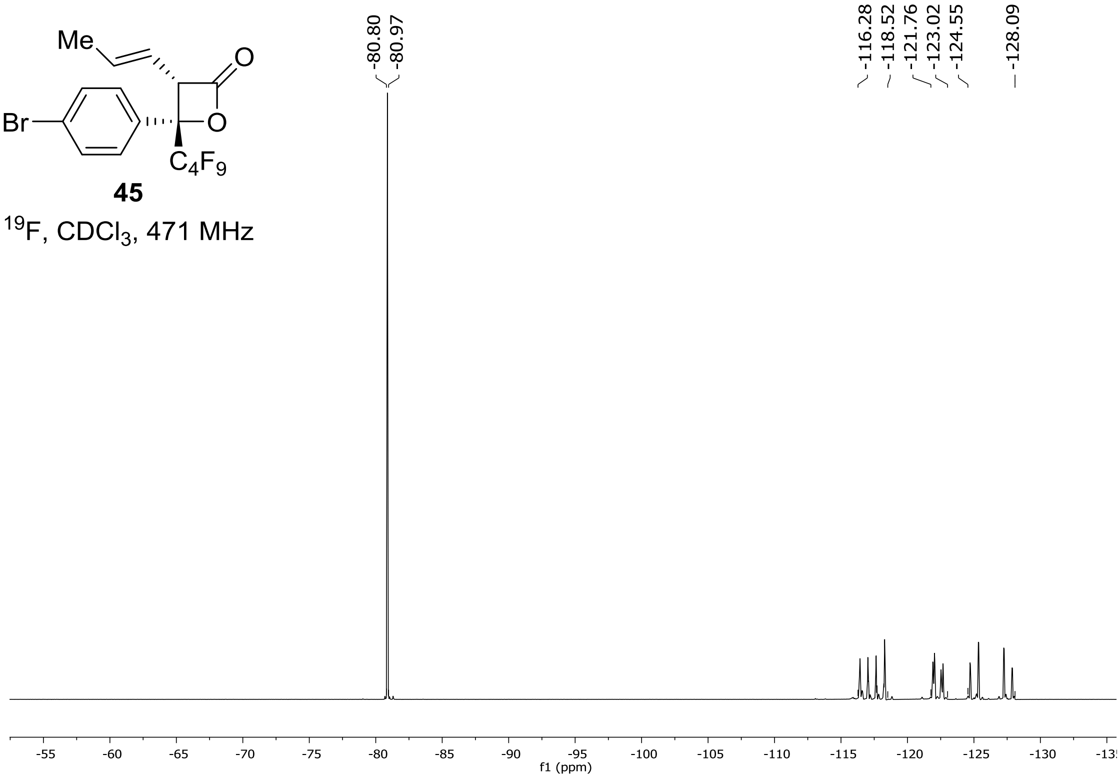
-80.80
-80.97
-116.28
-118.52
-121.76
-123.02
-124.55
-128.09
Me
O
Br
O
C_4F_9
45
^{19}F, $CDCl_3$, 471 MHz
f1 (ppm)

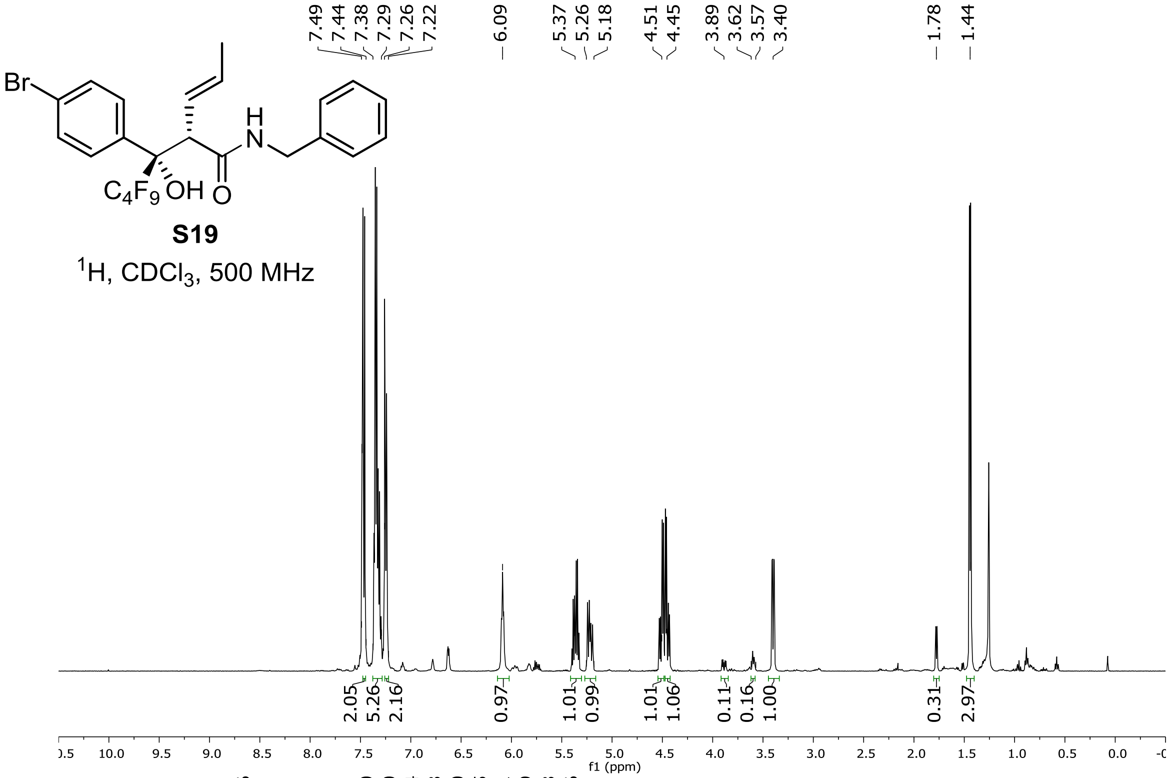

S19

^1H, $CDCl_3$, 500 MHz

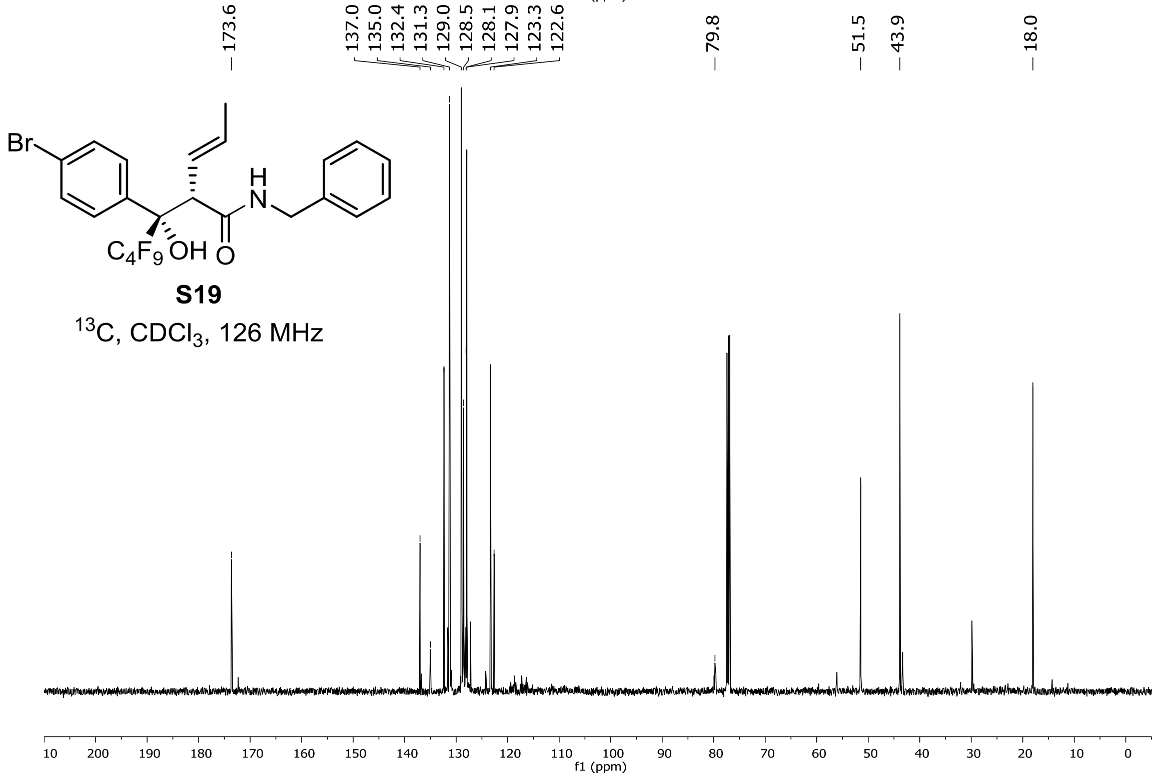

S19

^{13}C, $CDCl_3$, 126 MHz

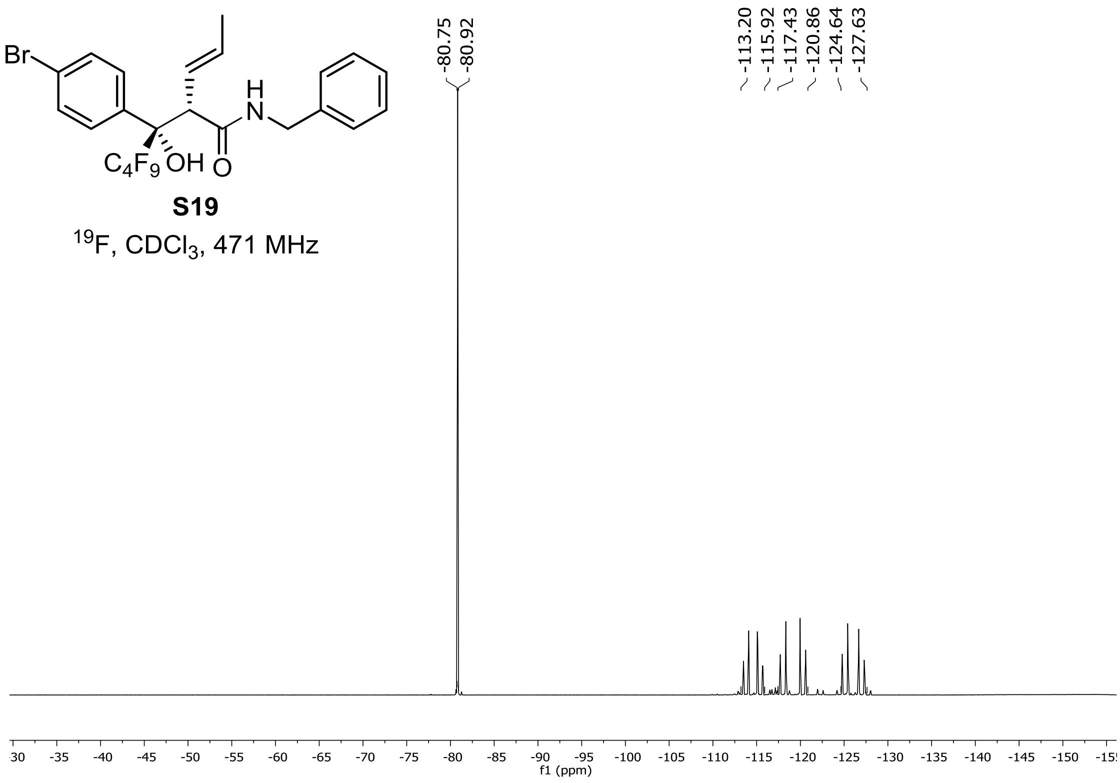
Br
C_4F_9 OH O
H
N
S19
^{19}F, CDCl$_3$, 471 MHz
-80.75
-80.92
-113.20
-115.92
-117.43
-120.86
-124.64
-127.63
f1 (ppm)

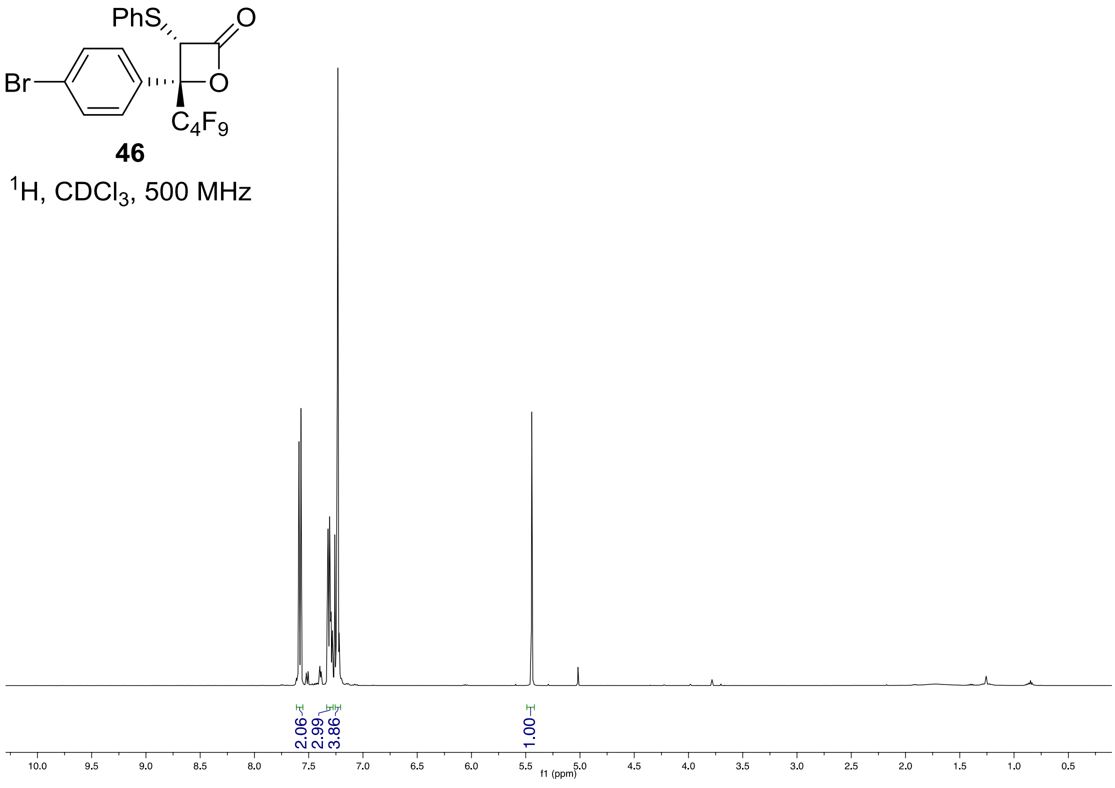
PhS
O
Br
O
C_4F_9
46
^{1}H, CDCl$_3$, 500 MHz
2.06
2.99
3.86
1.00
f1 (ppm)

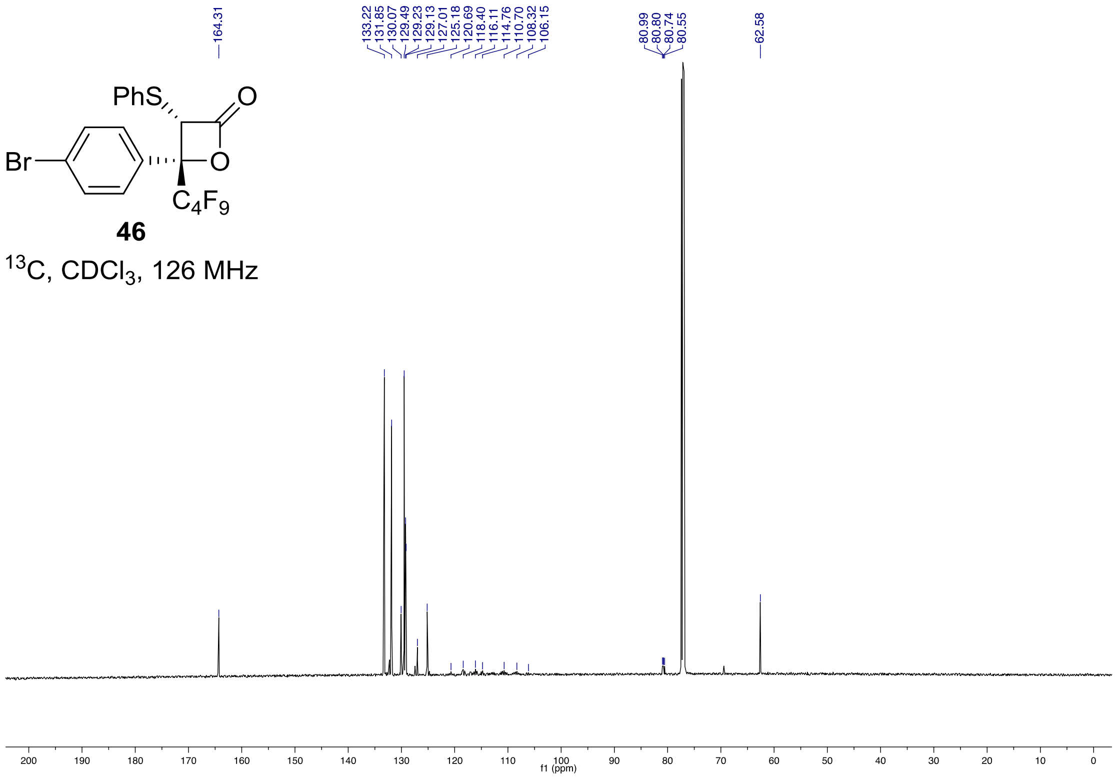

46

^{13}C, $CDCl_3$, 126 MHz

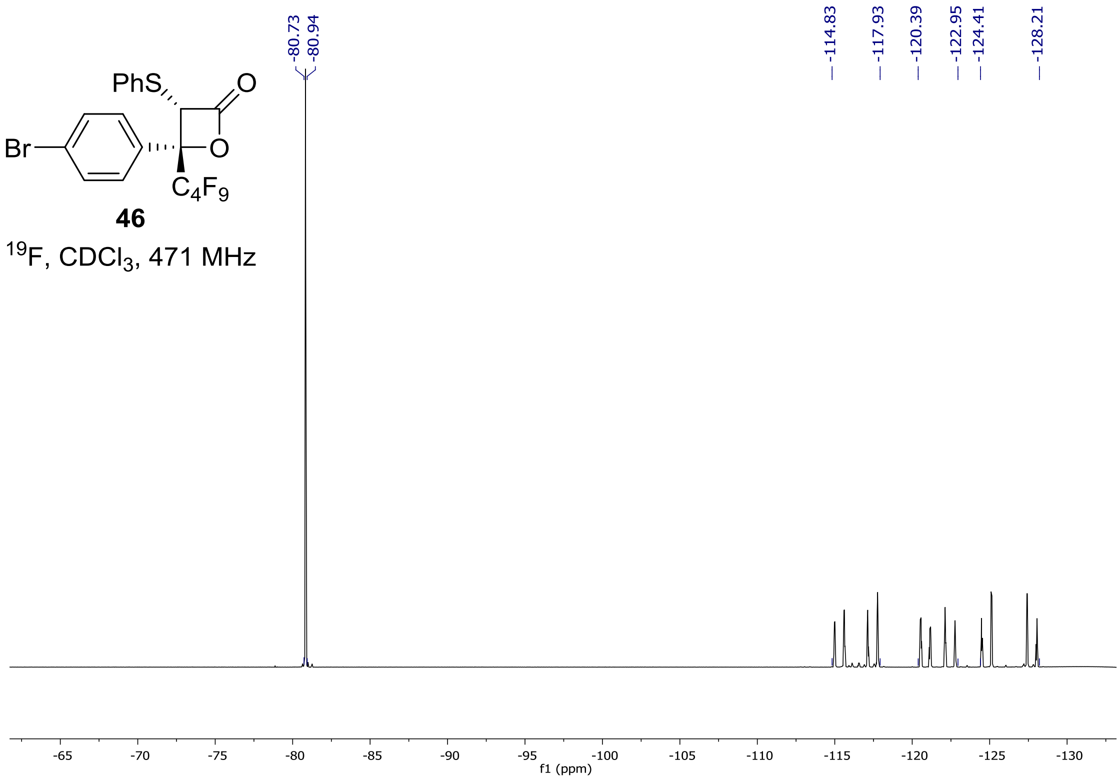

46

^{19}F, $CDCl_3$, 471 MHz

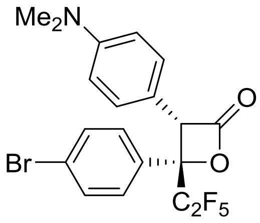

47

^{1}H, $CDCl_3$, 500 MHz

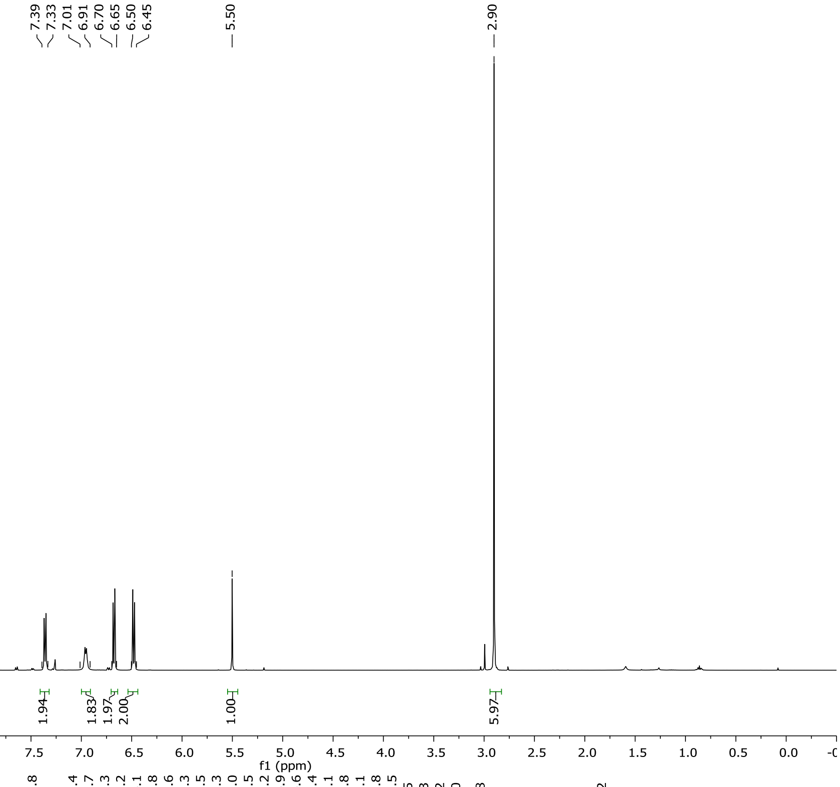

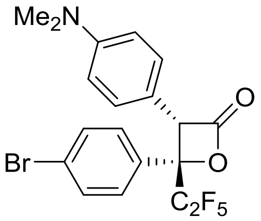

47

^{13}C, $CDCl_3$, 126 MHz

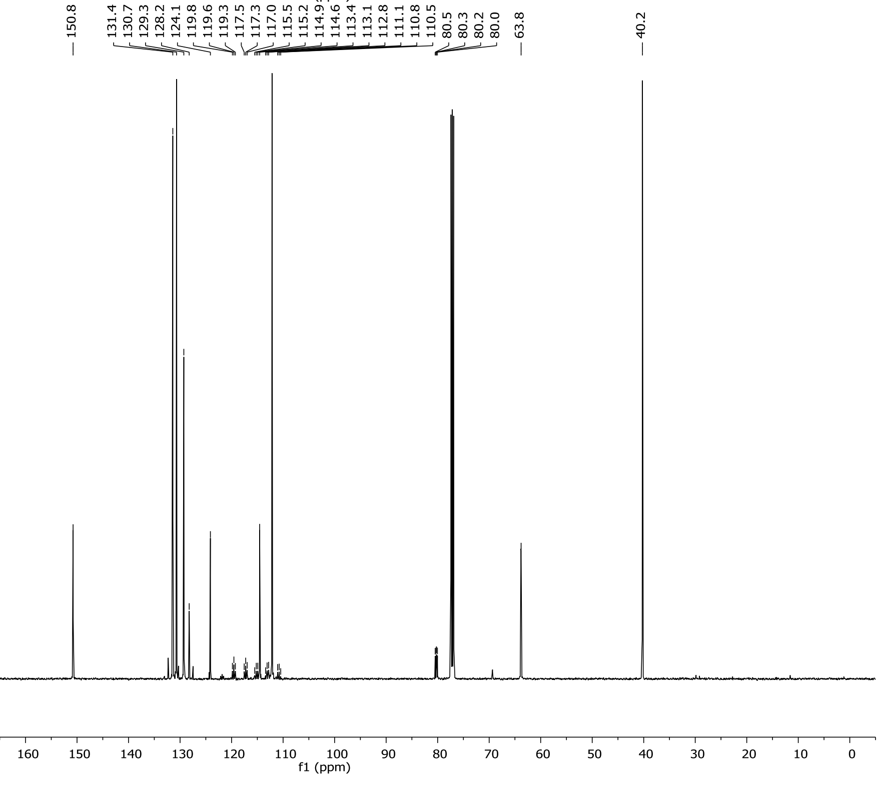

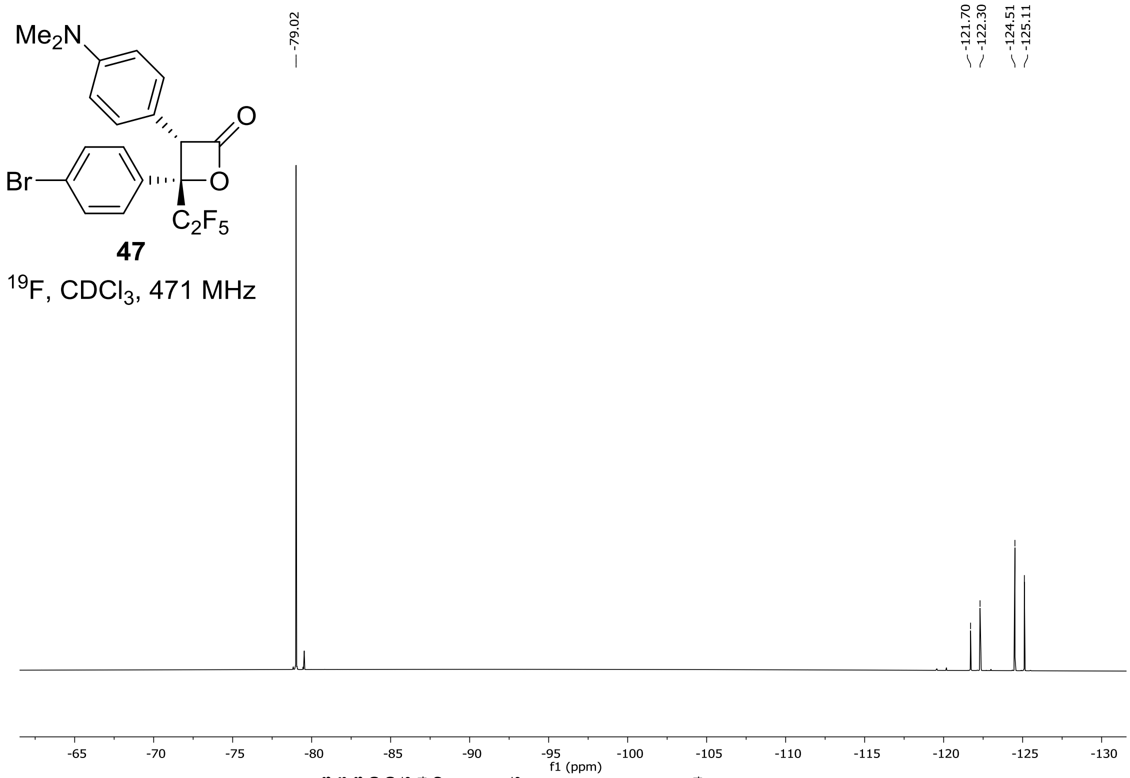
Me2N
O
Br
O
C2F5
47
19F, CDCl3, 471 MHz
-79.02
-121.70
-122.30
-124.51
-125.11
f1 (ppm)

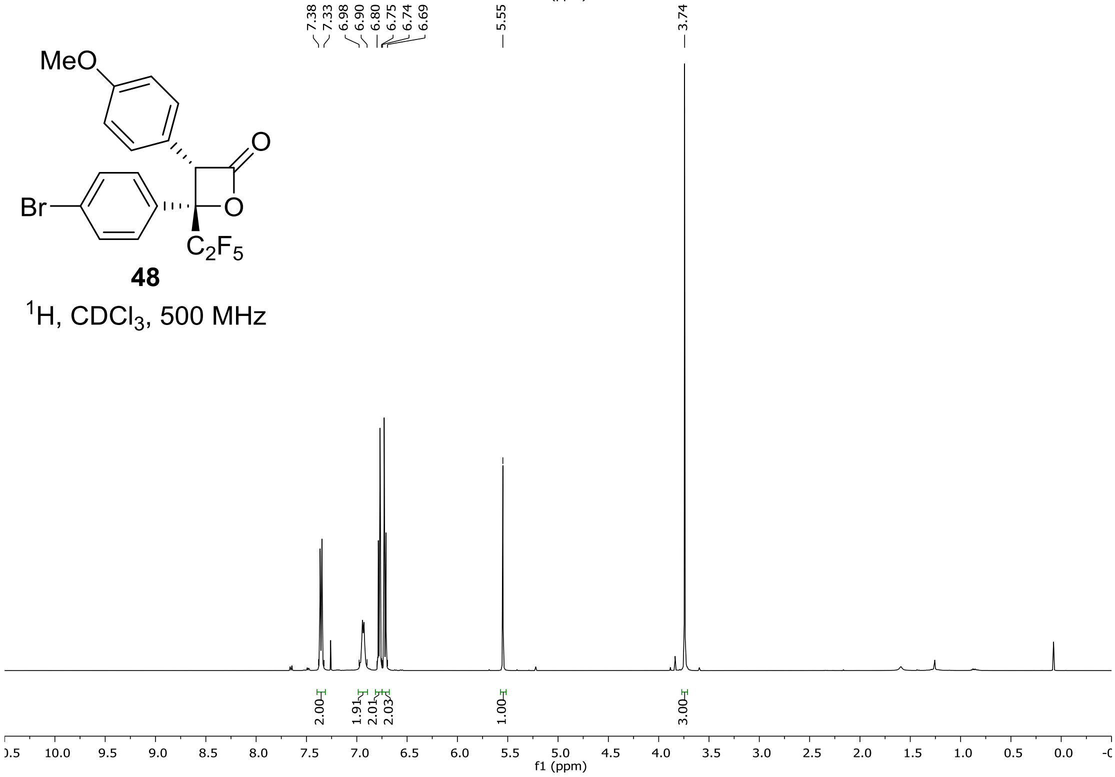
MeO
O
Br
O
C2F5
48
1H, CDCl3, 500 MHz
7.38
7.33
6.98
6.90
6.80
6.75
6.74
6.69
5.55
3.74
2.00
1.91
2.01
2.03
1.00
3.00
f1 (ppm)

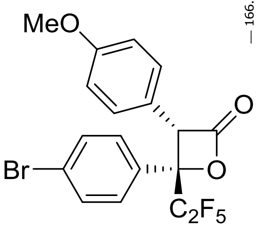

48

^{13}C, $CDCl_3$, 126 MHz

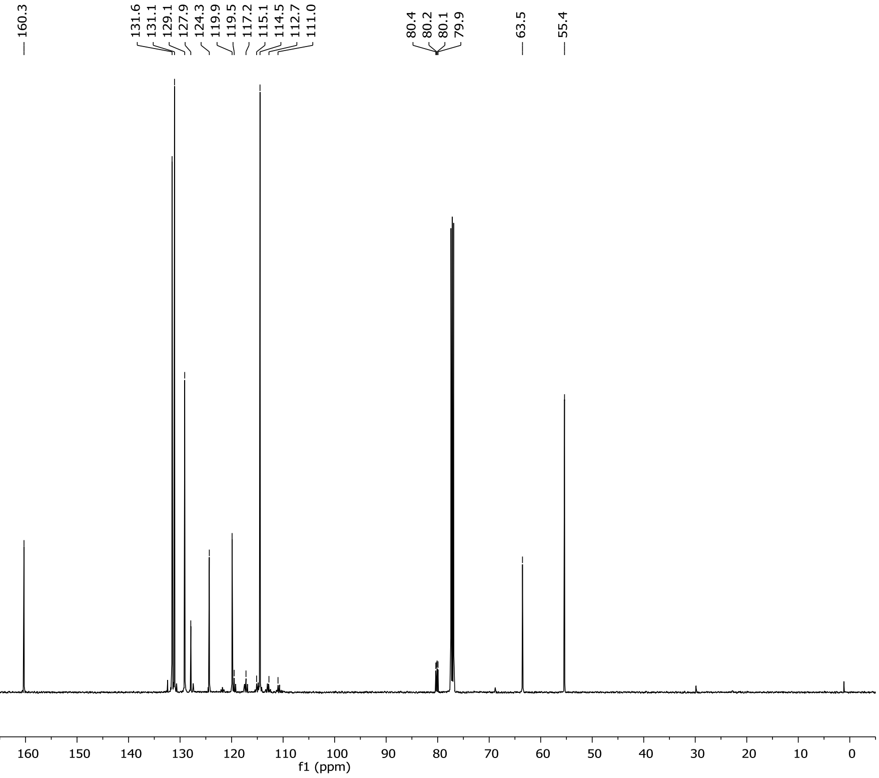

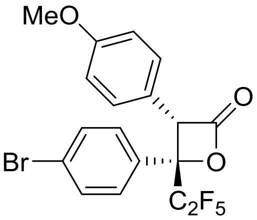

48

^{19}F, $CDCl_3$, 471 MHz

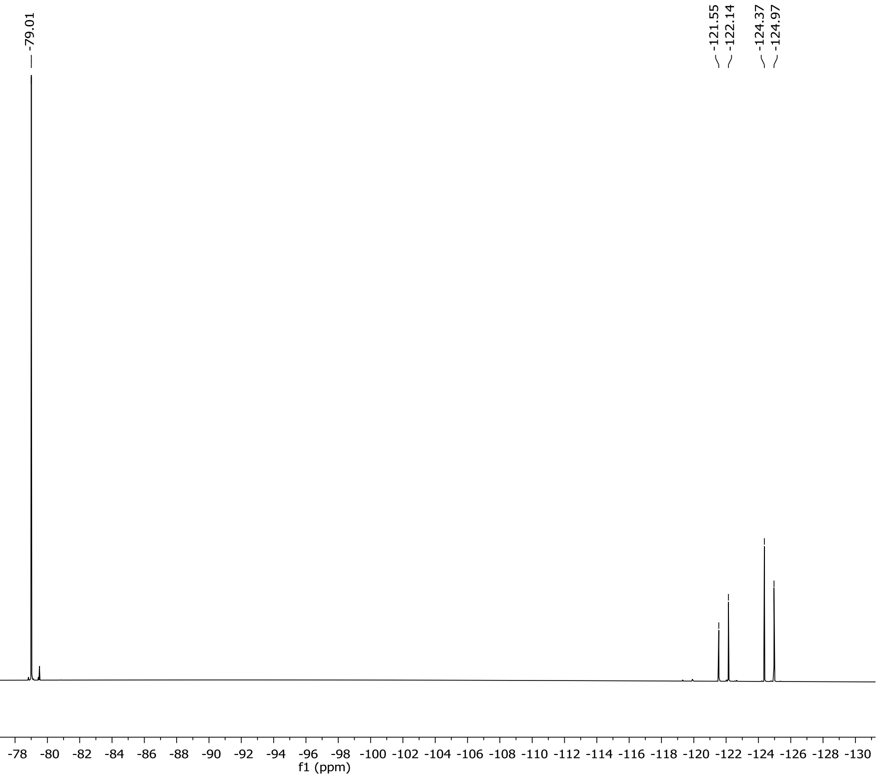

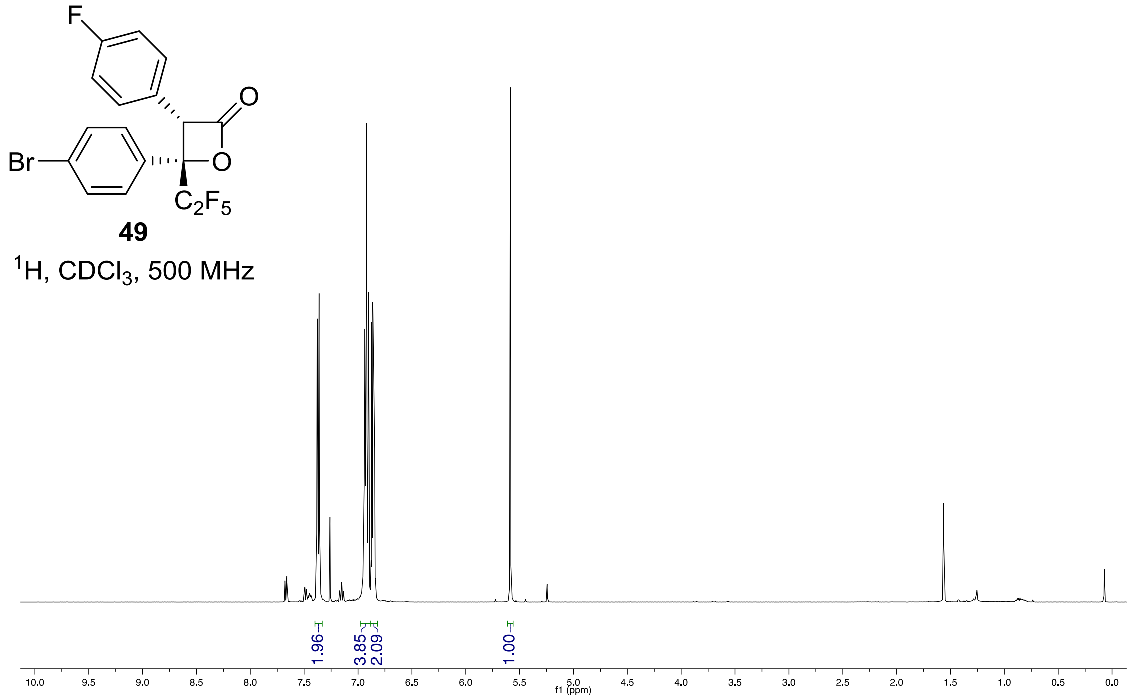
F
O
Br
O
C_2F_5
49
^1H, $CDCl_3$, 500 MHz
1.96
3.85
2.09
1.00
10.0 9.5 9.0 8.5 8.0 7.5 7.0 6.5 6.0 5.5 5.0 4.5 4.0 3.5 3.0 2.5 2.0 1.5 1.0 0.5 0.0
f1 (ppm)

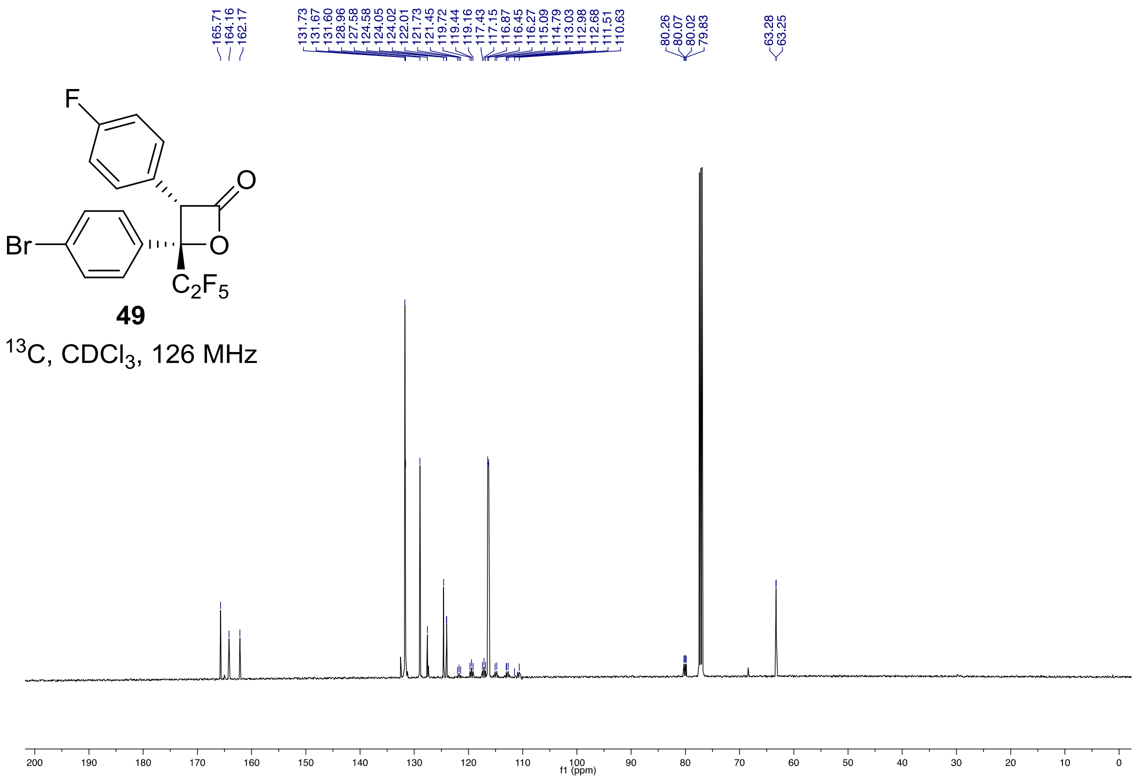
165.71
164.16
162.17
131.73
131.67
131.60
128.96
127.58
124.58
124.05
124.02
122.01
121.73
121.45
119.72
119.44
119.16
117.43
117.15
116.87
116.45
116.27
115.09
114.79
113.03
112.98
112.68
111.51
110.63
80.26
80.07
80.02
79.83
63.28
63.25
F
O
Br
O
C_2F_5
49
^{13}C, $CDCl_3$, 126 MHz
200 190 180 170 160 150 140 130 120 110 100 90 80 70 60 50 40 30 20 10 0
f1 (ppm)

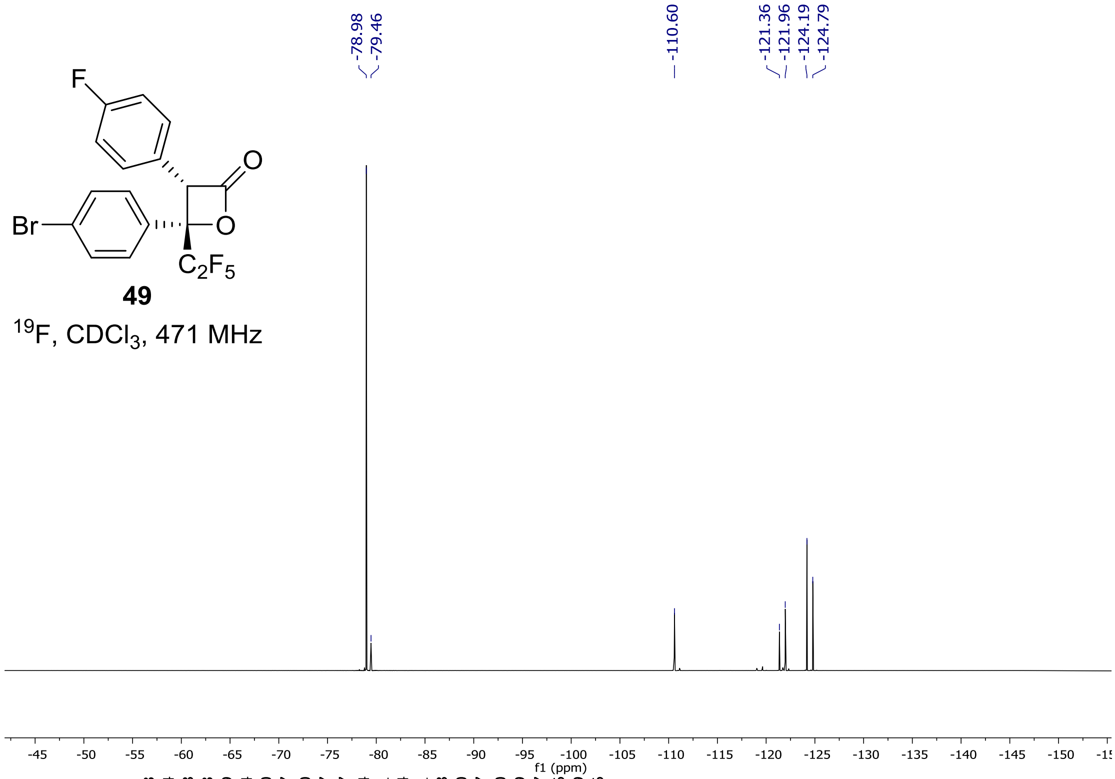
-78.98
-79.46
-110.60
-121.36
-121.96
-124.19
-124.79
F
O
Br
O
C_2F_5
49
^{19}F, $CDCl_3$, 471 MHz
-45 -50 -55 -60 -65 -70 -75 -80 -85 -90 -95 -100 -105 -110 -115 -120 -125 -130 -135 -140 -145 -150
f1 (ppm)

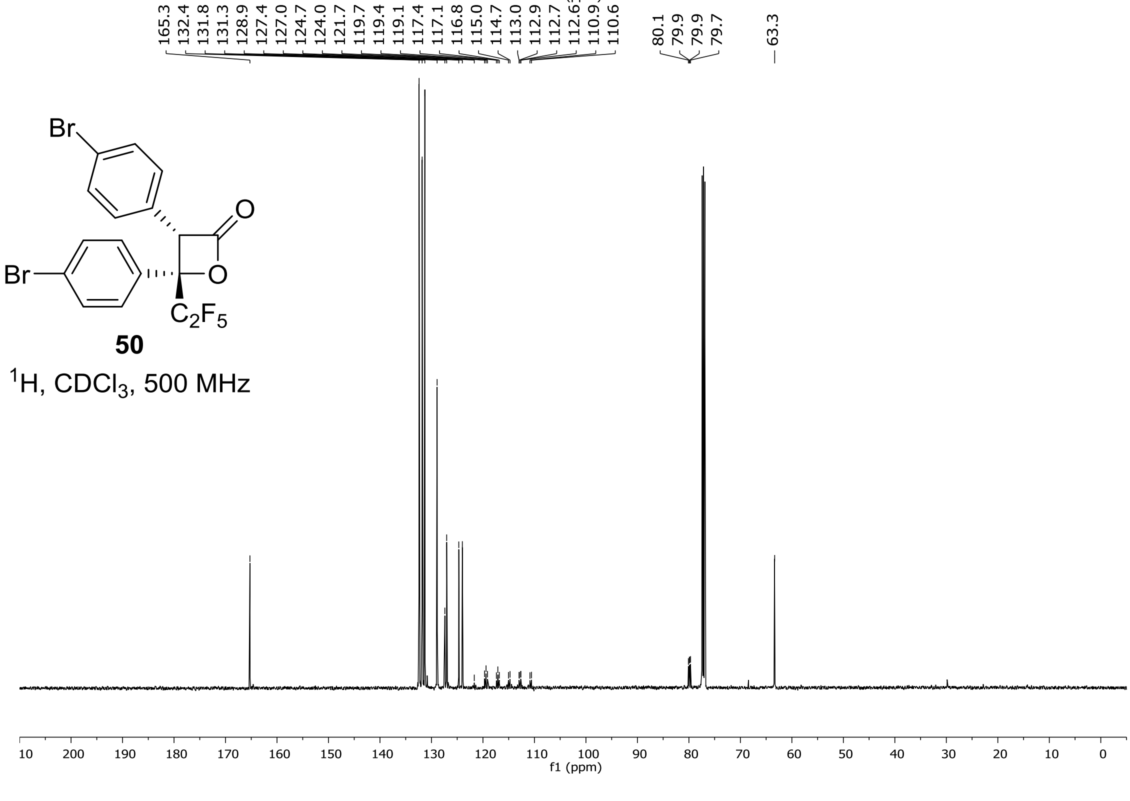
165.3
132.4
131.8
131.3
128.9
127.4
127.0
124.7
124.0
121.7
119.7
119.4
119.1
117.4
117.1
116.8
115.0
114.7
113.0
112.9
112.7
112.6
110.9
110.6
80.1
79.9
79.9
79.7
63.3
Br
O
Br
O
C_2F_5
50
^1H, $CDCl_3$, 500 MHz
200 190 180 170 160 150 140 130 120 110 100 90 80 70 60 50 40 30 20 10 0
f1 (ppm)

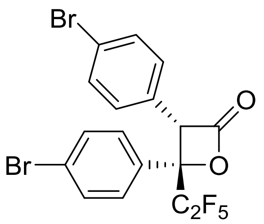

50

^{13}C, $CDCl_3$, 126 MHz

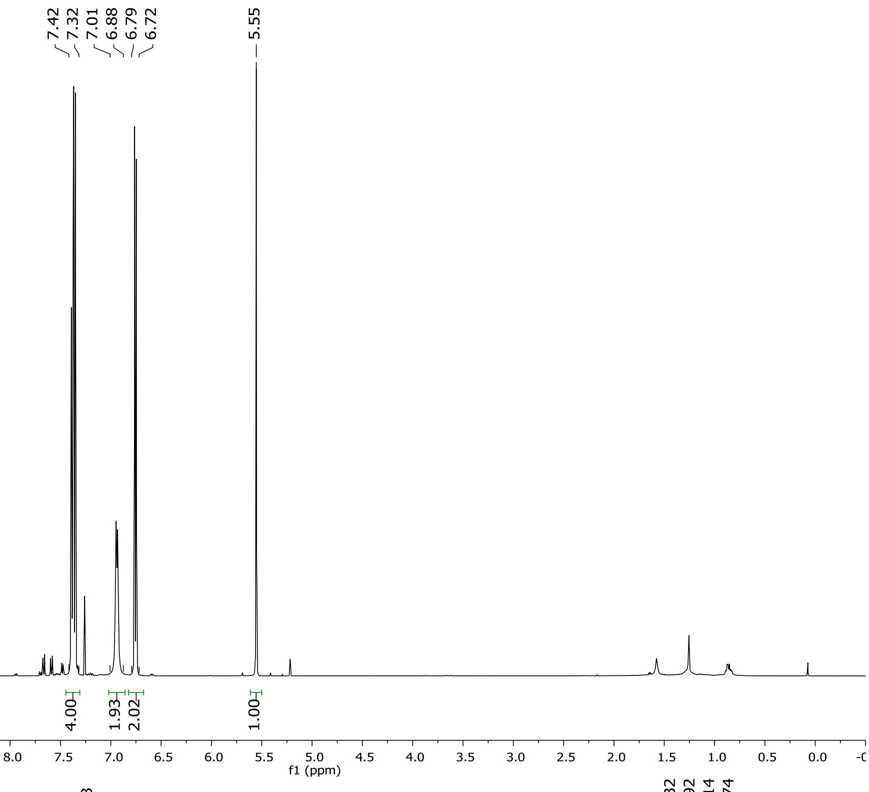

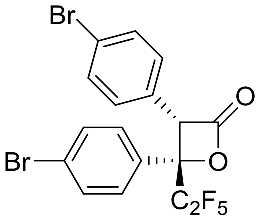

50

^{19}F, $CDCl_3$, 471 MHz

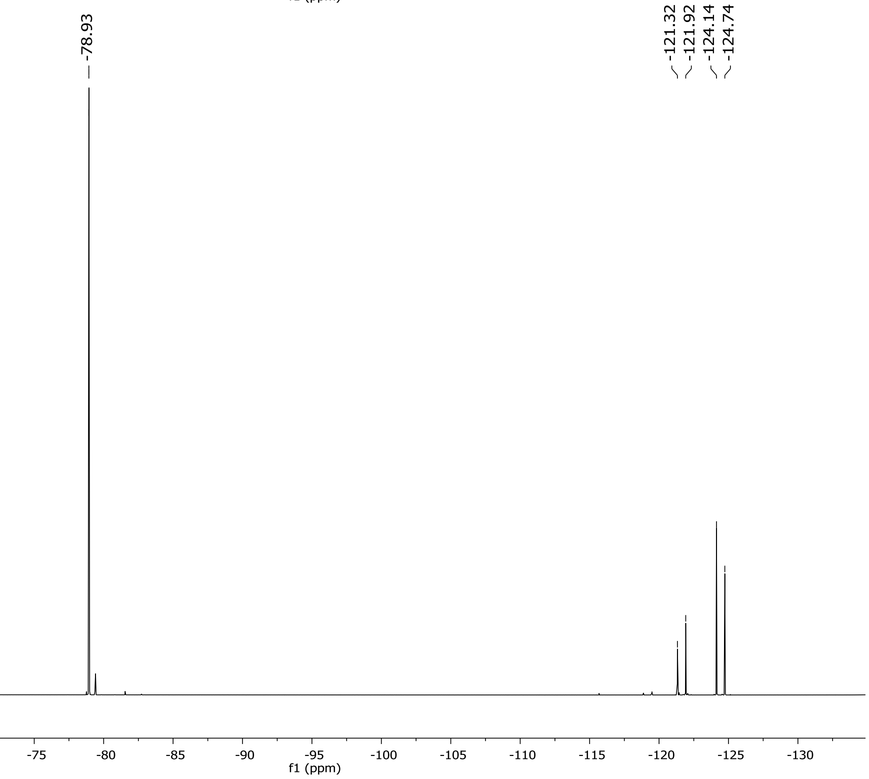

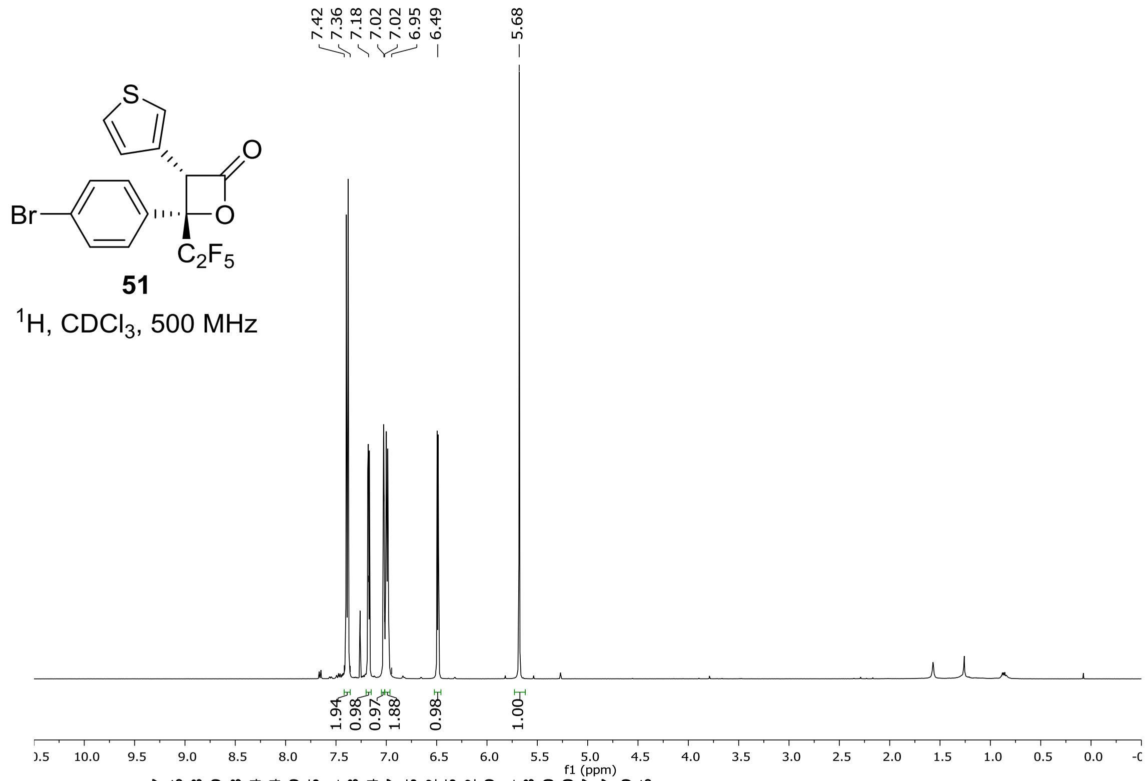

7.42
7.36
7.18
7.02
7.02
6.95
6.49
5.68
S
O
O
Br
C2F5
51
1H, CDCl3, 500 MHz
1.94
0.98
0.97
1.88
0.98
1.00
f1 (ppm)

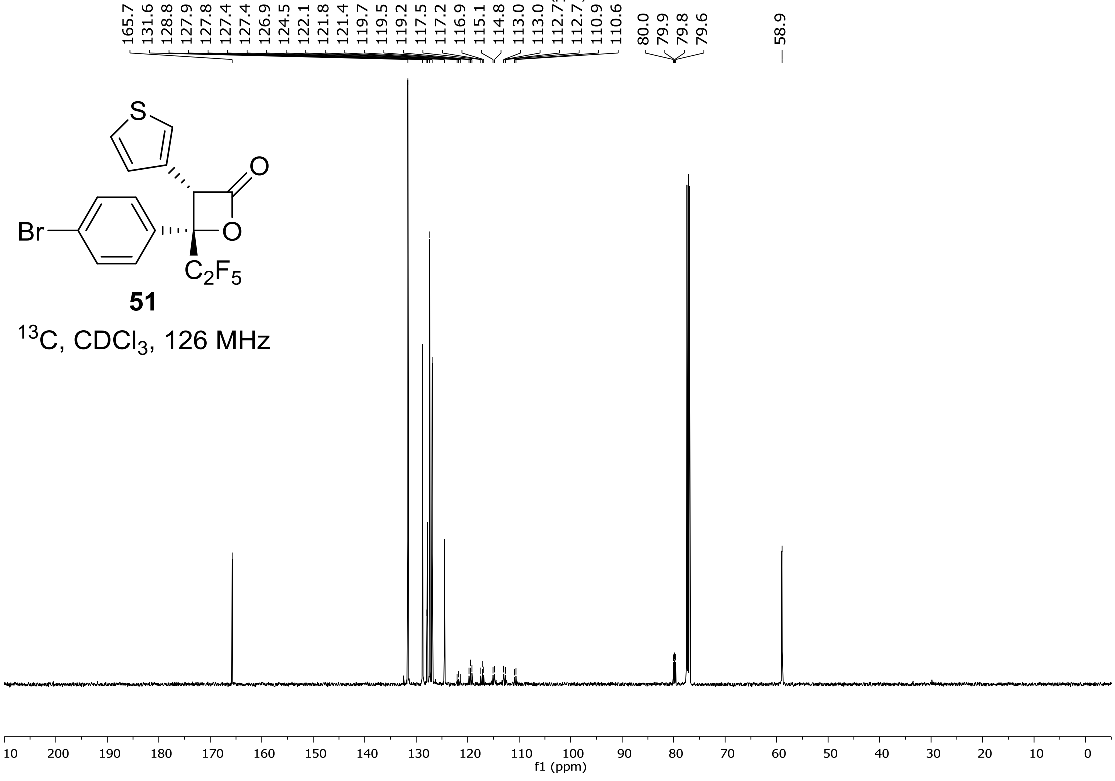

165.7
131.6
128.8
127.9
127.8
127.4
127.4
126.9
124.5
122.1
121.8
121.4
119.7
119.5
119.2
117.5
117.2
116.9
115.1
114.8
113.0
113.0
112.7
112.7
110.9
110.6
80.0
79.9
79.8
79.6
58.9
S
O
O
Br
C2F5
51
13C, CDCl3, 126 MHz
f1 (ppm)

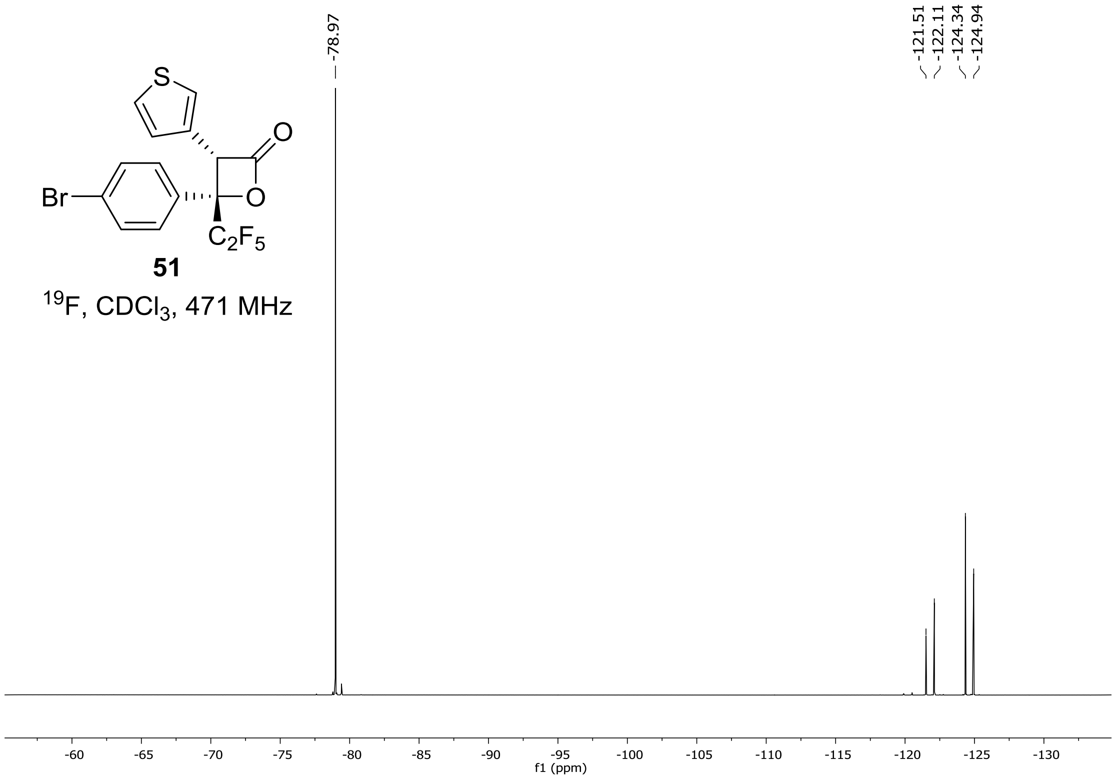

-78.97
-121.51
-122.11
-124.34
-124.94
Br
S
O
O
C_2F_5
51
^{19}F, $CDCl_3$, 471 MHz
-60 -65 -70 -75 -80 -85 -90 -95 -100 -105 -110 -115 -120 -125 -130
f1 (ppm)

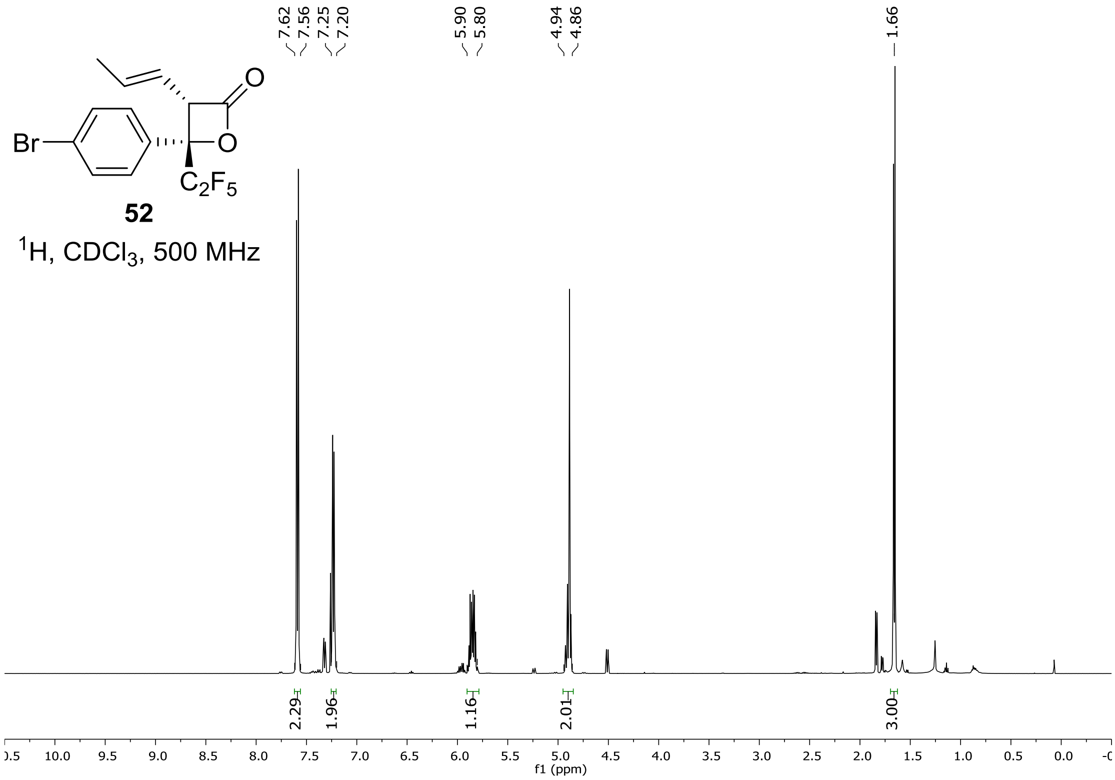

7.62
7.56
7.25
7.20
5.90
5.80
4.94
4.86
1.66
Br
O
O
C_2F_5
52
^1H, $CDCl_3$, 500 MHz
2.29
1.96
1.16
2.01
3.00
10.0 9.5 9.0 8.5 8.0 7.5 7.0 6.5 6.0 5.5 5.0 4.5 4.0 3.5 3.0 2.5 2.0 1.5 1.0 0.5 0.0
f1 (ppm)

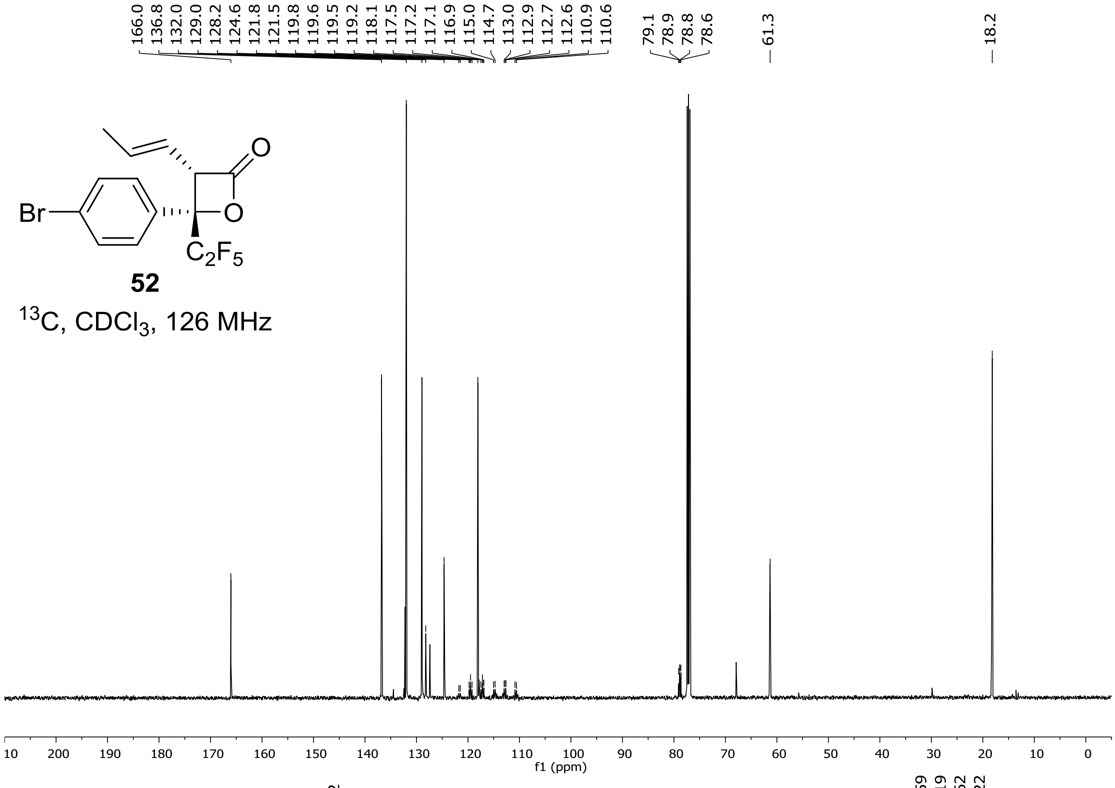
166.0
136.8
132.0
129.0
128.2
124.6
121.8
121.5
119.8
119.6
119.5
119.2
118.1
117.5
117.2
117.1
116.9
115.0
114.7
113.0
112.9
112.7
112.6
110.9
110.6
79.1
78.9
78.8
78.6
61.3
18.2
O
Br
O
C2F5
52
13C, CDCl3, 126 MHz
f1 (ppm)

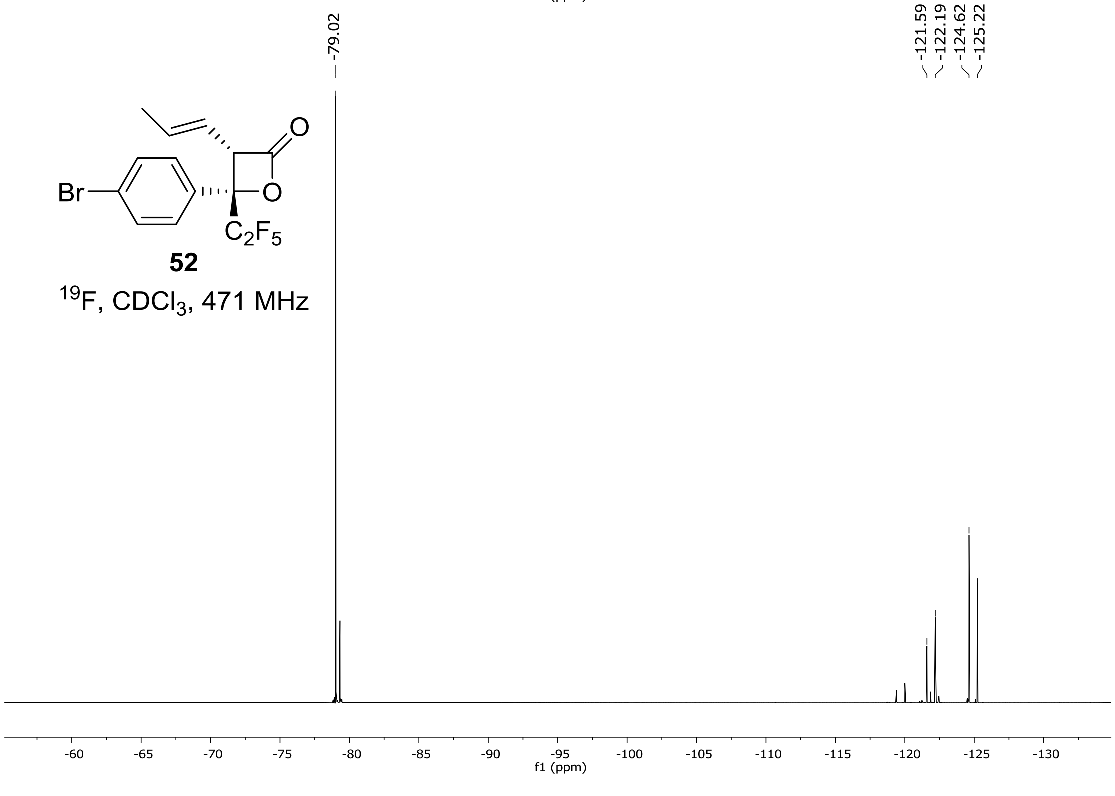
-79.02
-121.59
-122.19
-124.62
-125.22
O
Br
O
C2F5
52
19F, CDCl3, 471 MHz
f1 (ppm)

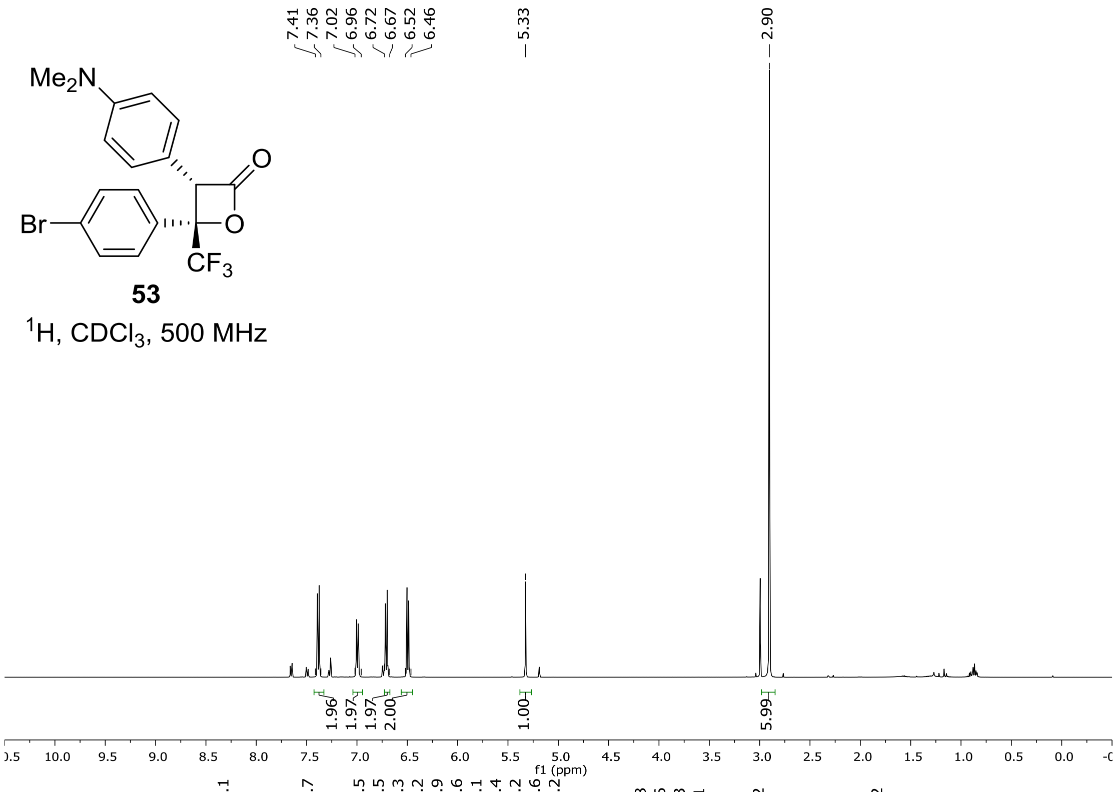
Me2N
O
Br
O
CF3
53
1H, CDCl3, 500 MHz
7.41
7.36
7.02
6.96
6.72
6.67
6.52
6.46
5.33
2.90
1.96
1.97
1.97
2.00
1.00
5.99
f1 (ppm)

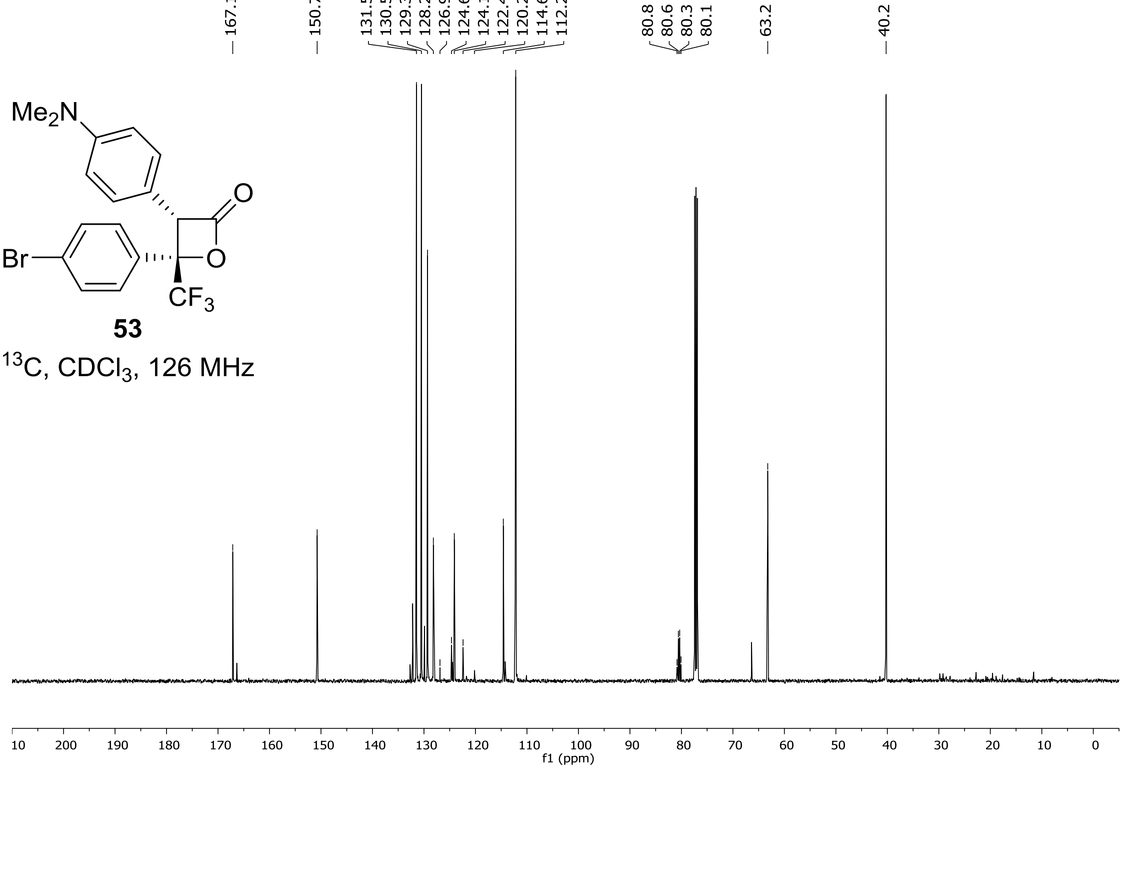
Me2N
O
Br
O
CF3
53
13C, CDCl3, 126 MHz
167.1
150.7
131.5
130.5
129.3
128.2
126.9
124.6
124.1
122.4
120.2
114.6
112.2
80.8
80.6
80.3
80.1
63.2
40.2
f1 (ppm)

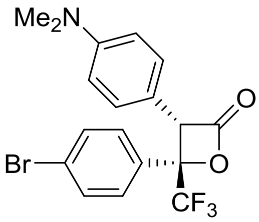

53

^{19}F, $CDCl_3$, 471 MHz

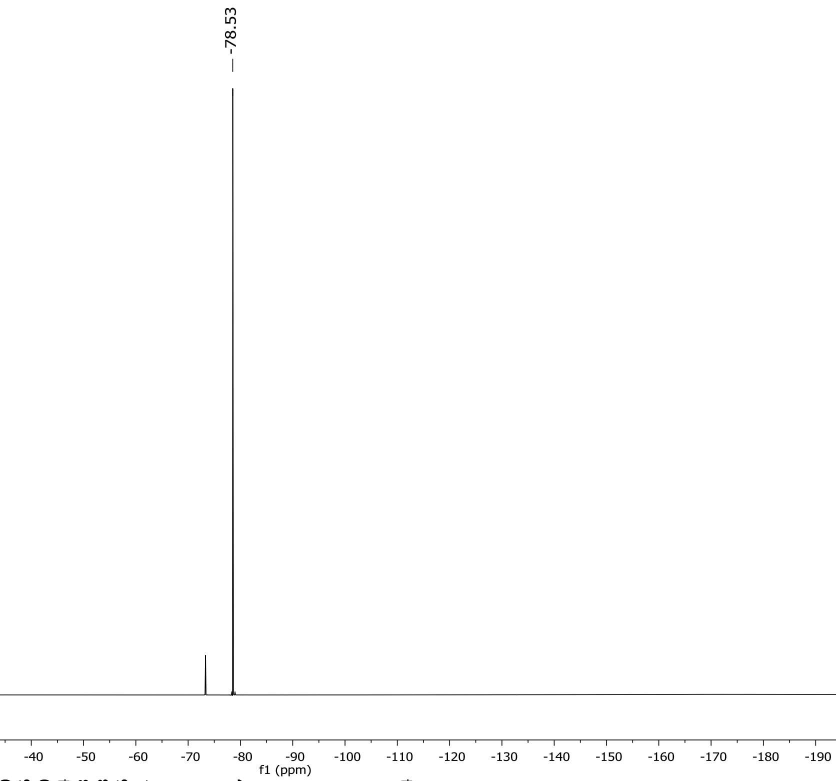

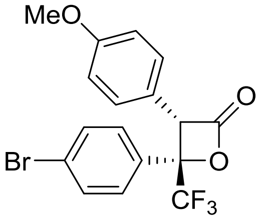

54

^{1}H, $CDCl_3$, 500 MHz

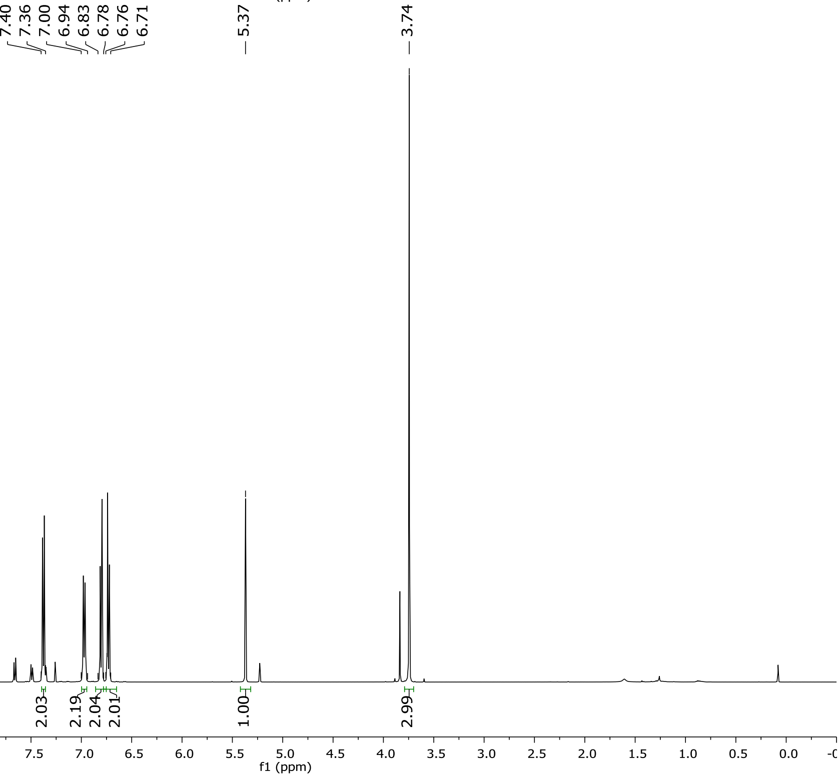

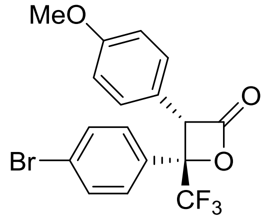

54

^{13}C, $CDCl_3$, 126 MHz

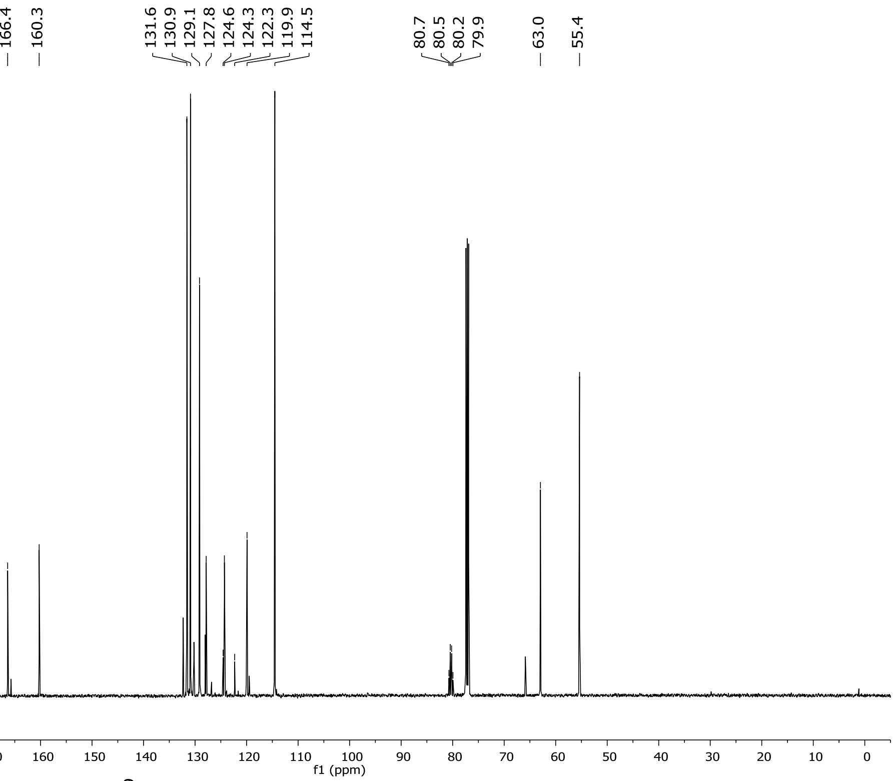

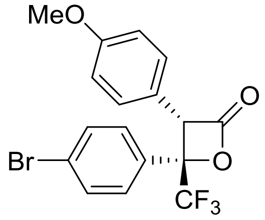

54

^{19}F, $CDCl_3$, 471 MHz

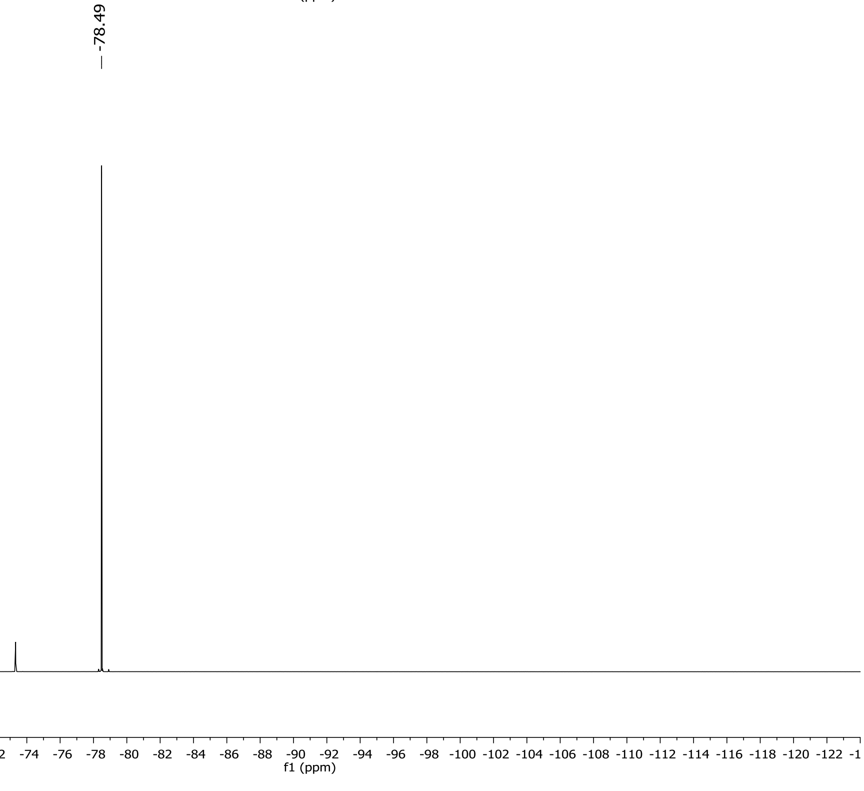

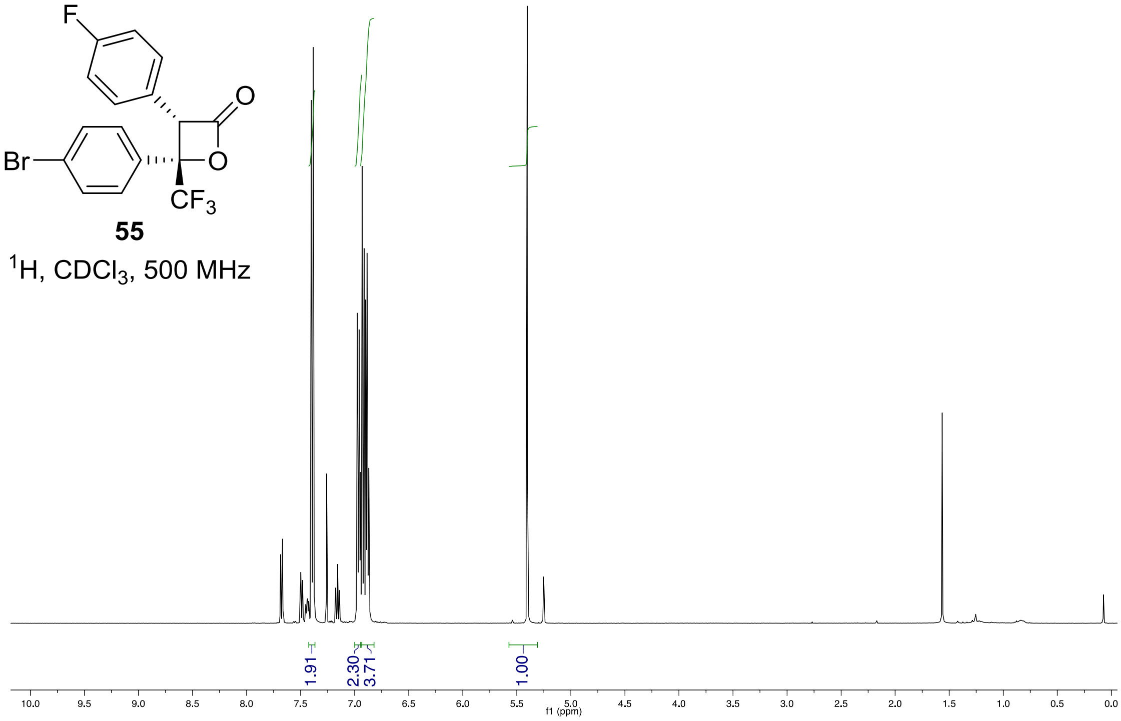

55

^{1}H, $CDCl_3$, 500 MHz

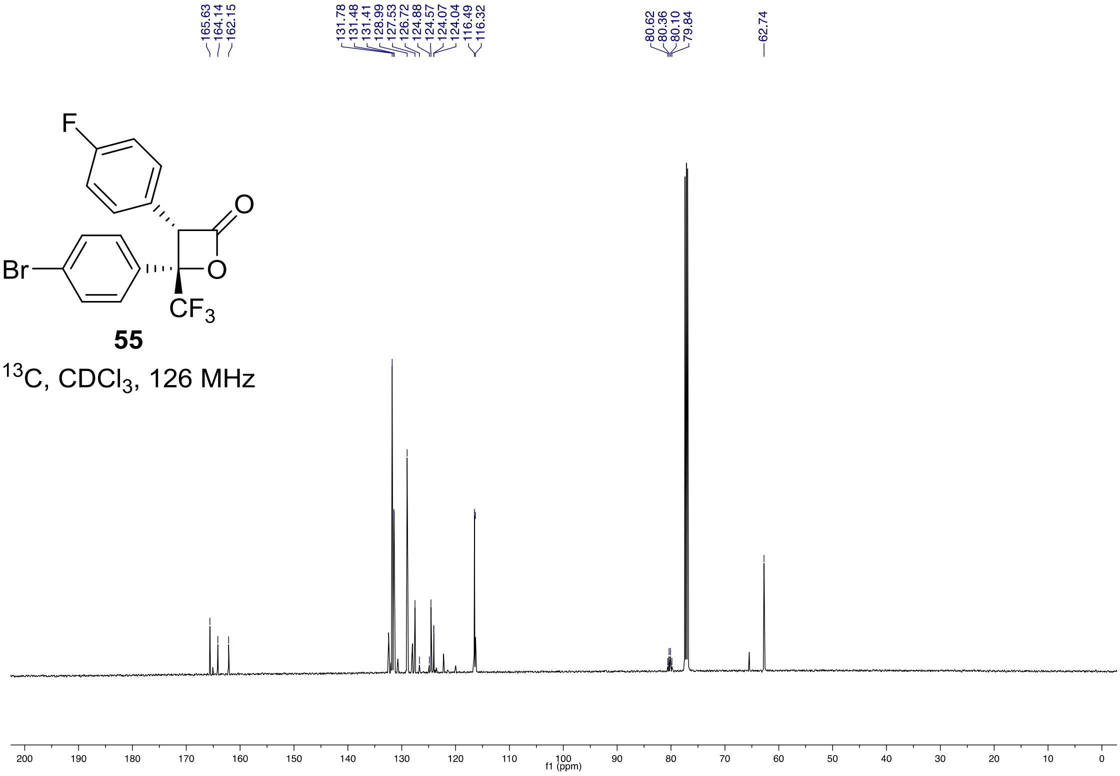

55

^{13}C, $CDCl_3$, 126 MHz

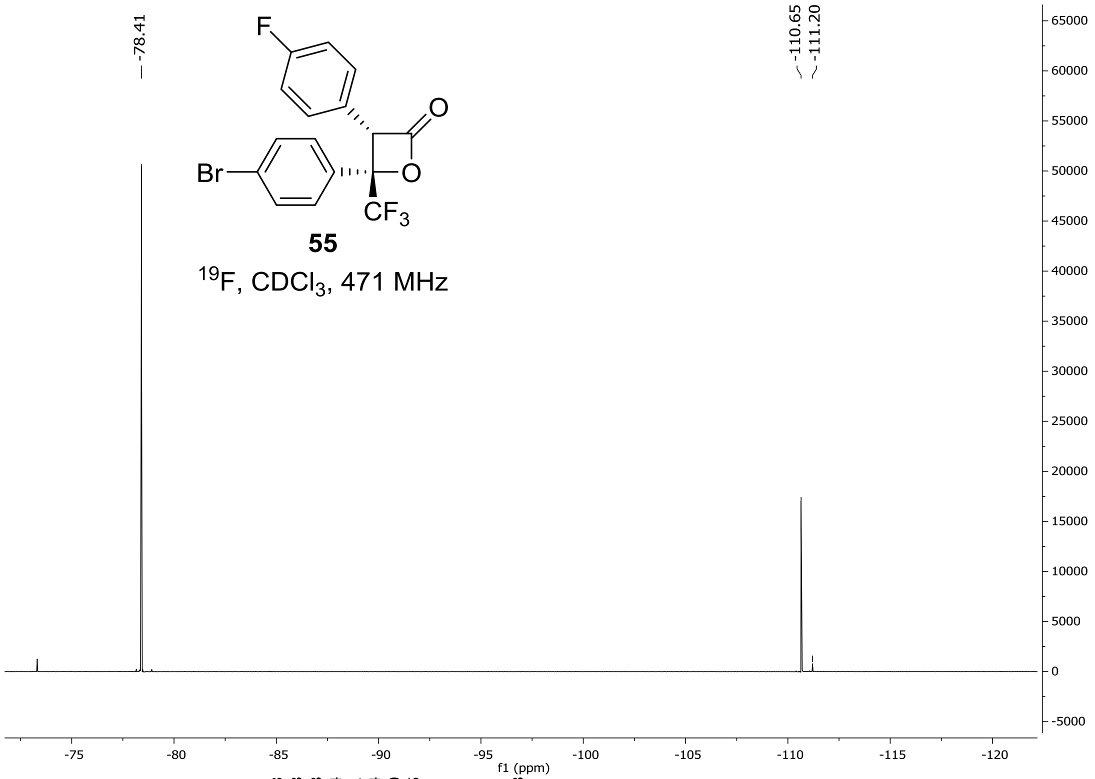
-78.41
-110.65
-111.20
F
O
Br
O
CF3
55
19F, CDCl3, 471 MHz
f1 (ppm)

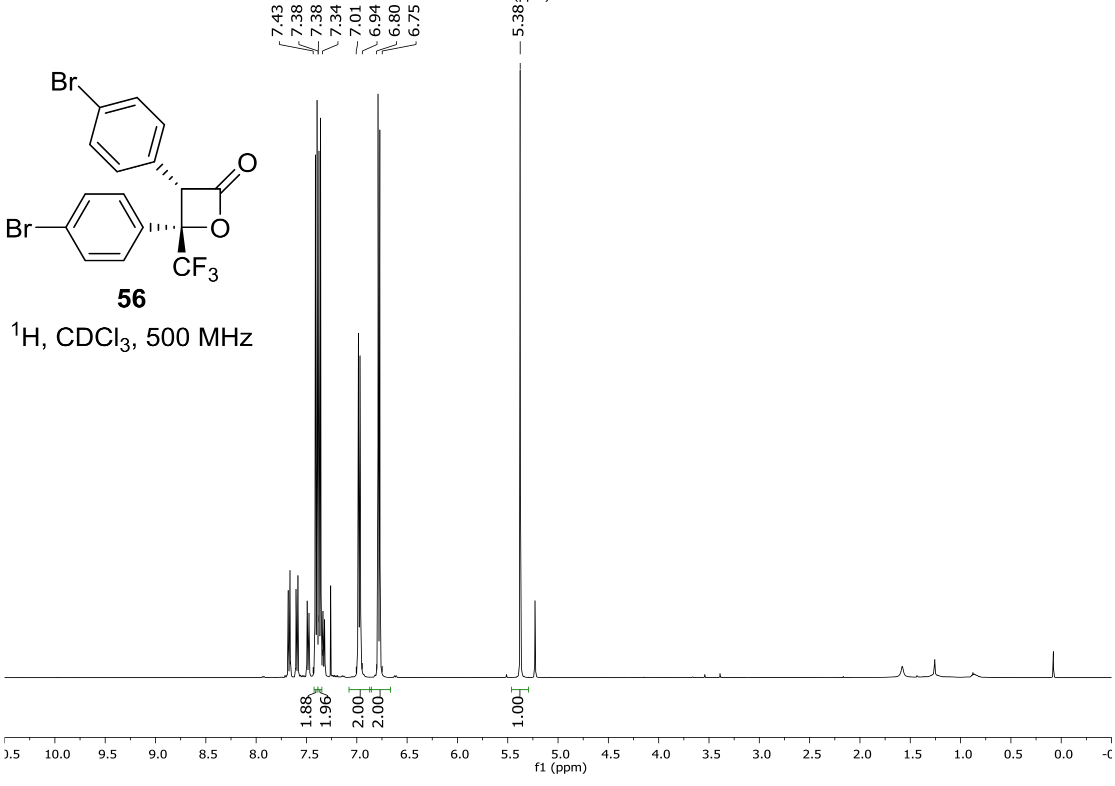
7.43
7.38
7.38
7.34
7.01
6.94
6.80
6.75
5.38
Br
O
Br
O
CF3
56
1H, CDCl3, 500 MHz
1.88
1.96
2.00
2.00
1.00
f1 (ppm)

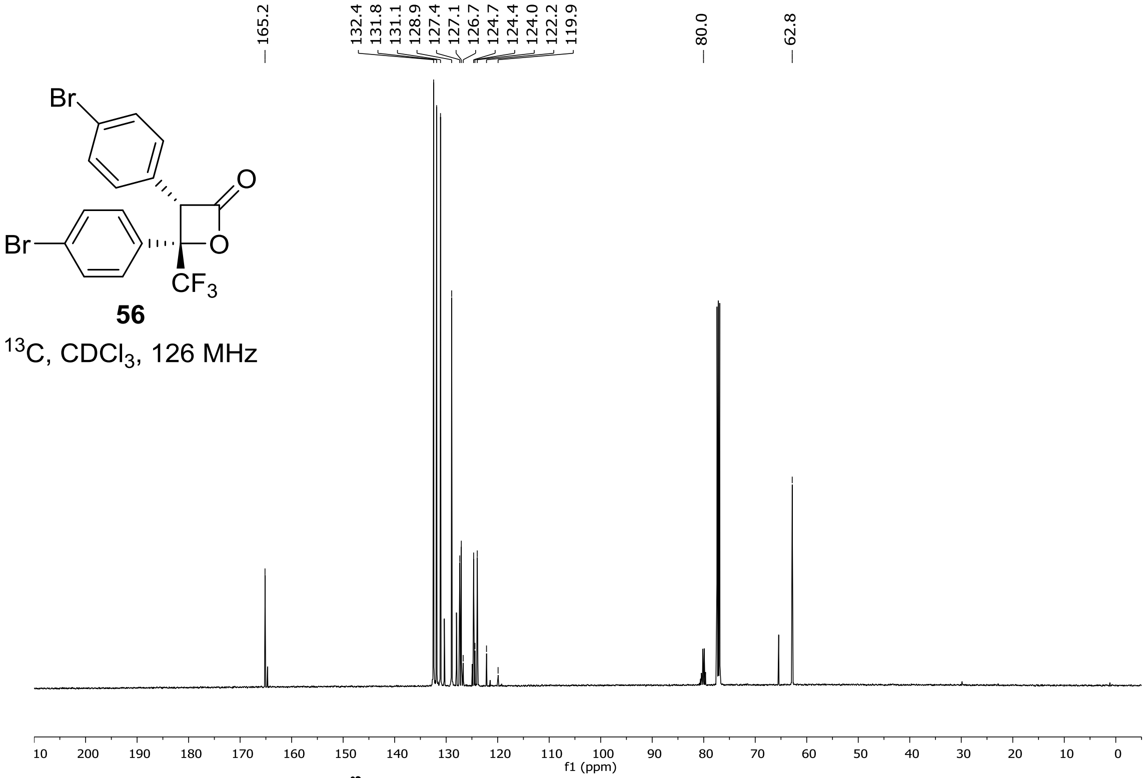
165.2
132.4
131.8
131.1
128.9
127.4
127.1
126.7
124.7
124.4
124.0
122.2
119.9
80.0
62.8
Br
O
Br
O
CF3
56
13C, CDCl3, 126 MHz
f1 (ppm)

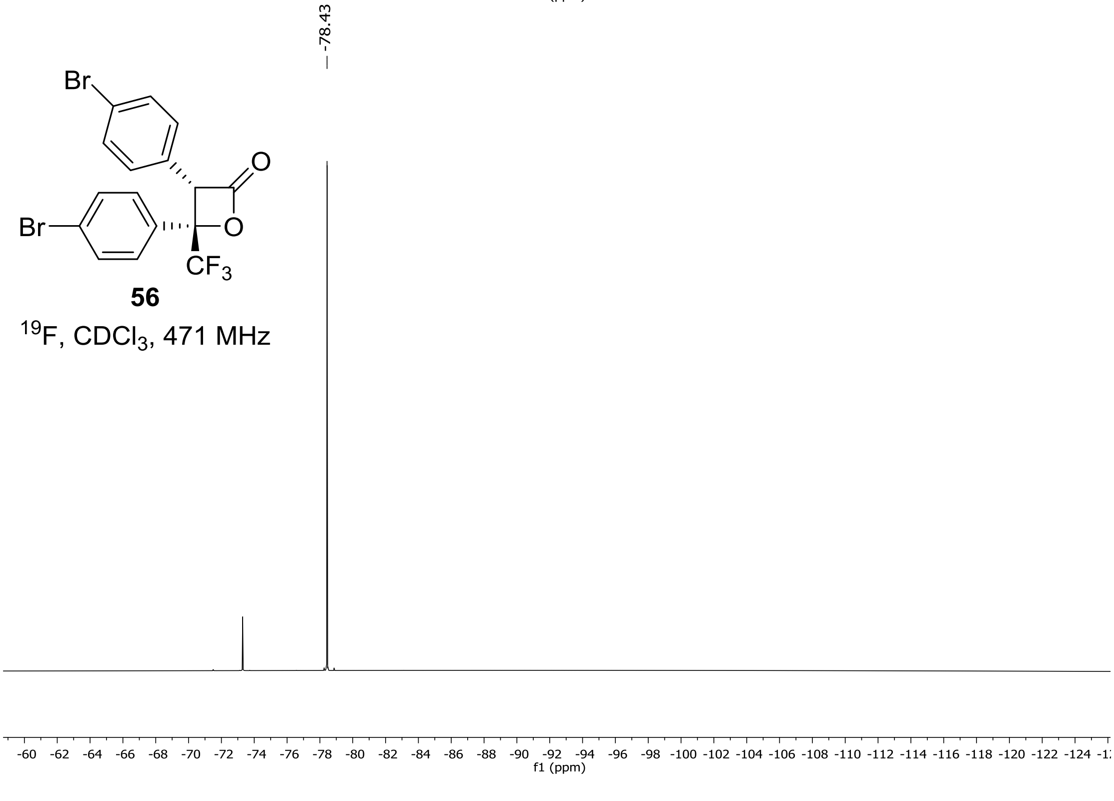
-78.43
Br
O
Br
O
CF3
56
19F, CDCl3, 471 MHz
f1 (ppm)

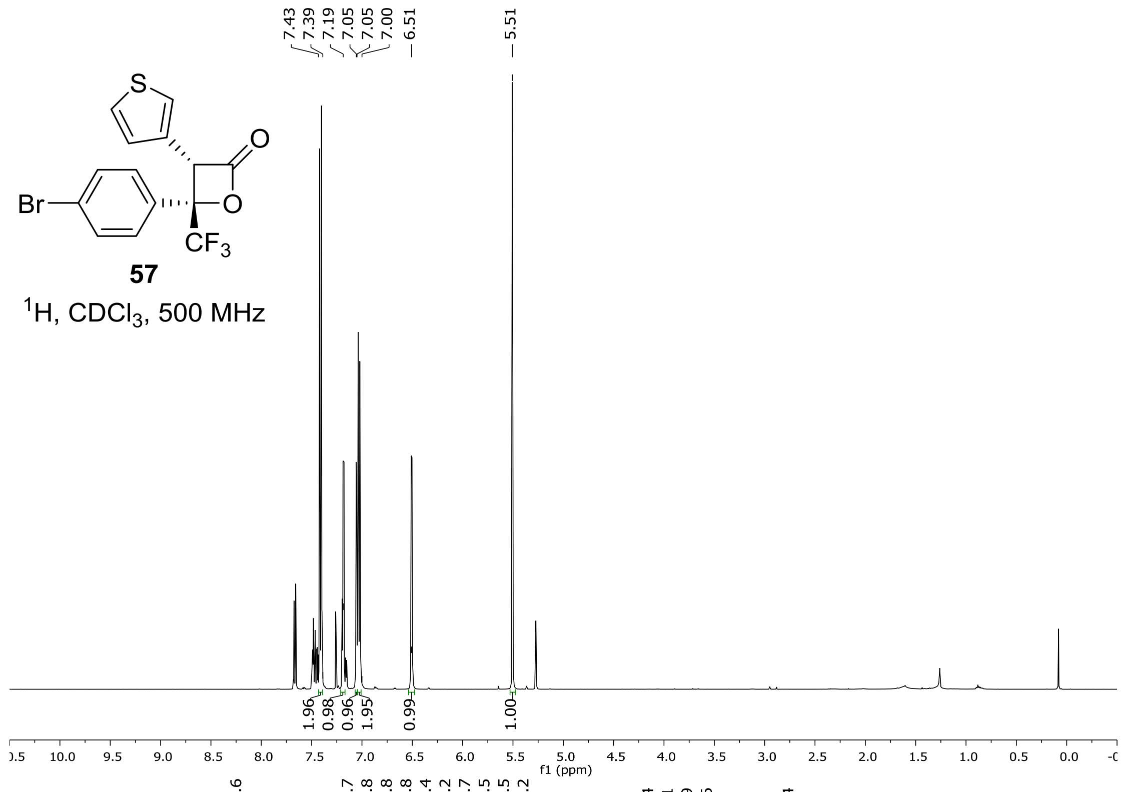
57
^{1}H, $CDCl_3$, 500 MHz
7.43
7.39
7.19
7.05
7.05
7.00
6.51
5.51
1.96
0.98
0.96
1.95
0.99
1.00
f1 (ppm)

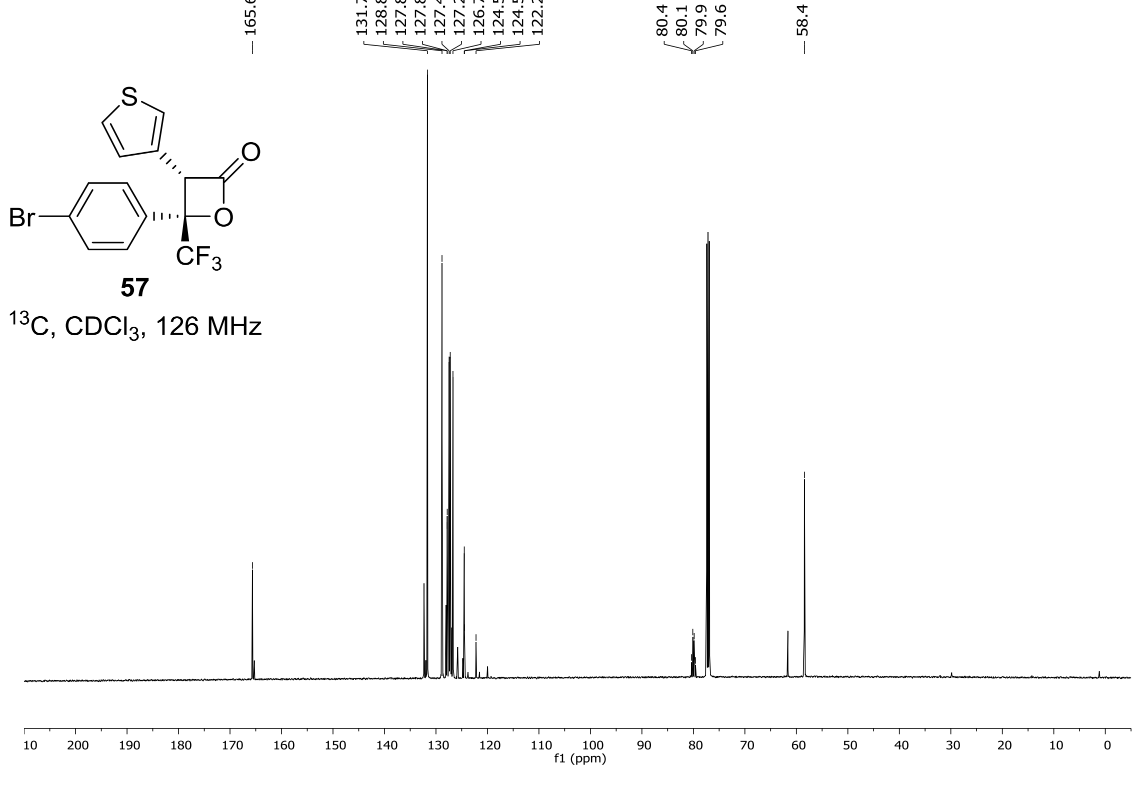
57
^{13}C, $CDCl_3$, 126 MHz
165.6
131.7
128.8
127.8
127.8
127.4
127.2
126.7
124.5
124.5
122.2
80.4
80.1
79.9
79.6
58.4
f1 (ppm)

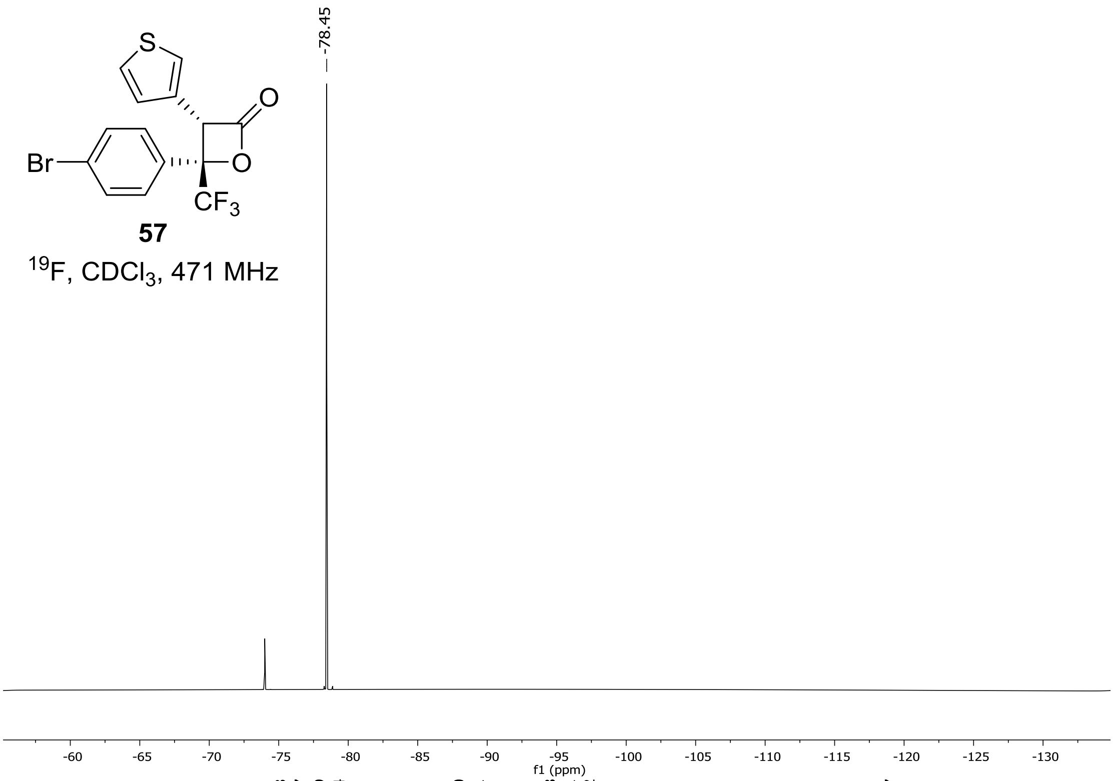

57

^{19}F, $CDCl_3$, 471 MHz

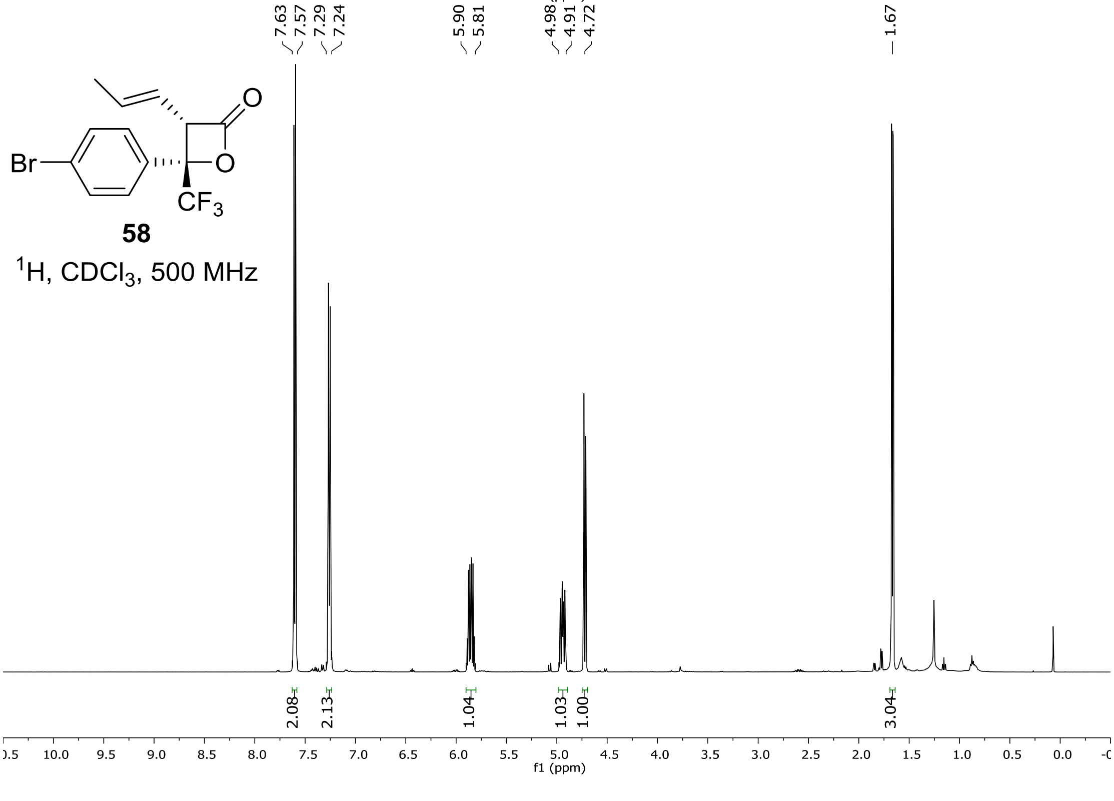

58

^{1}H, $CDCl_3$, 500 MHz

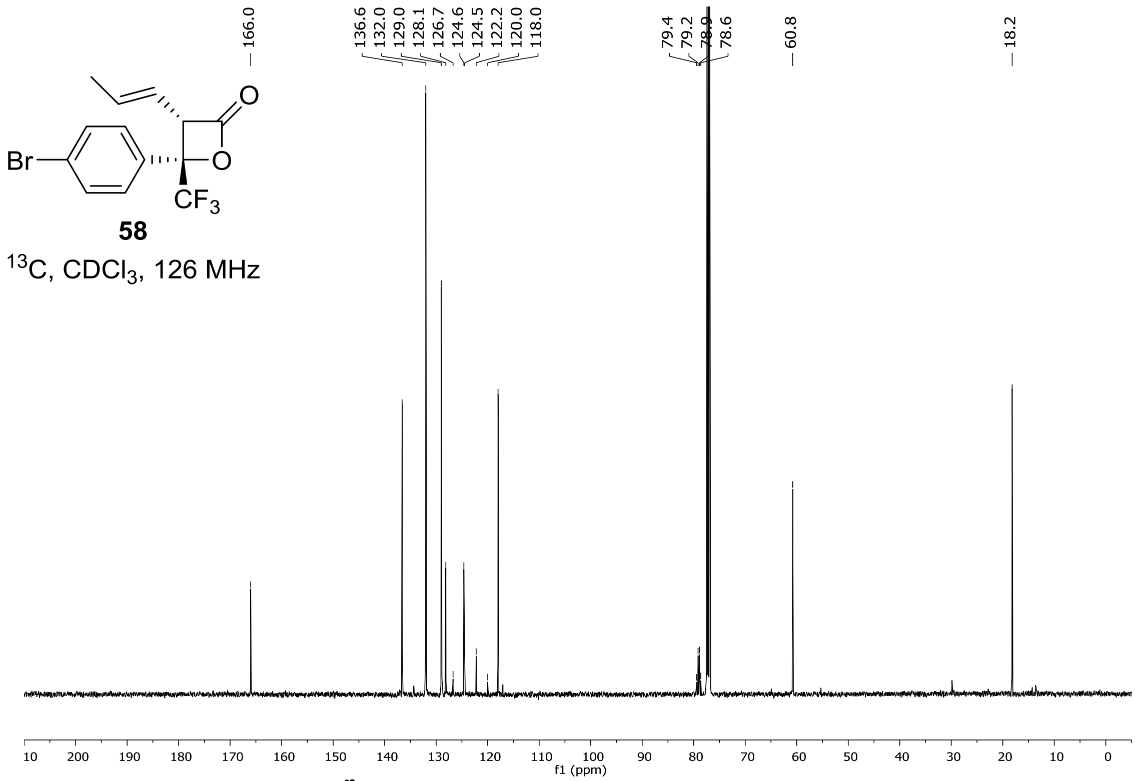
166.0
136.6
132.0
129.0
128.1
126.7
124.6
124.5
122.2
120.0
118.0
79.4
79.2
78.6
60.8
18.2
Br
O
O
CF3
58
13C, CDCl3, 126 MHz
f1 (ppm)

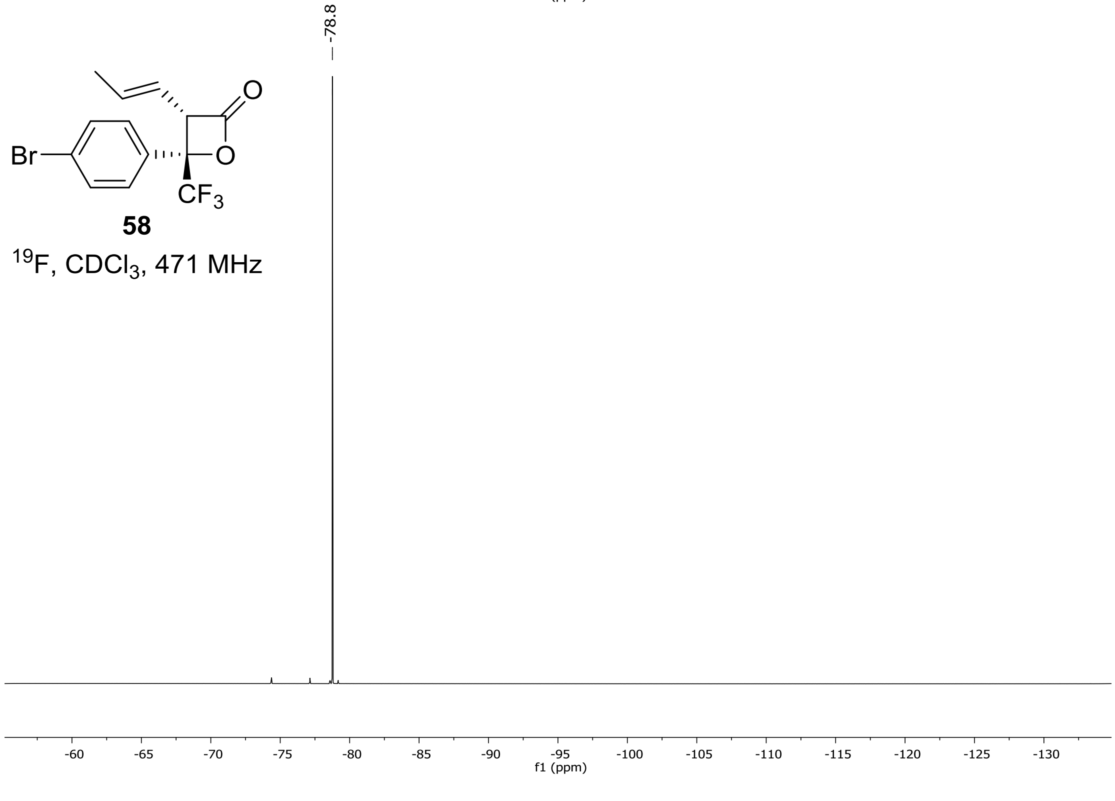
-78.8
Br
O
O
CF3
58
19F, CDCl3, 471 MHz
f1 (ppm)

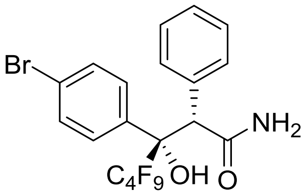

59

^{1}H, $CDCl_3$, 500 MHz

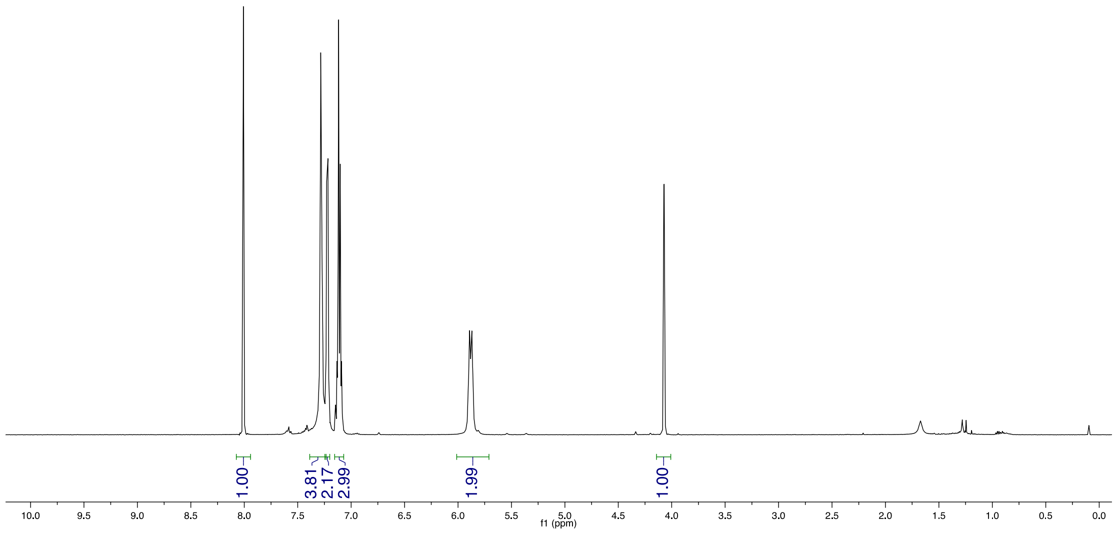

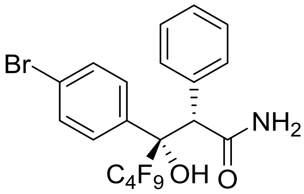

59

^{13}C, $CDCl_3$, 126 MHz

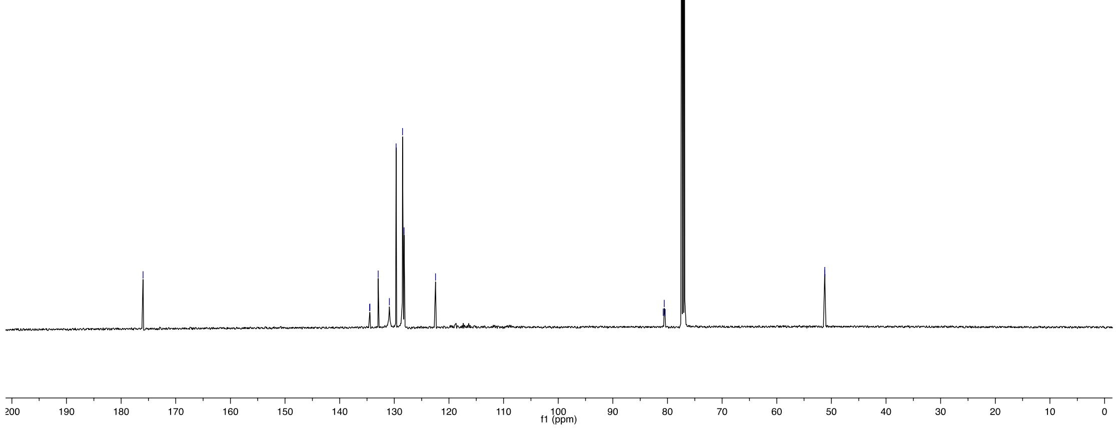

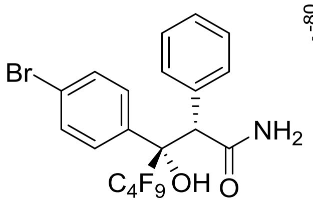

59

^{19}F, $CDCl_3$, 471 MHz

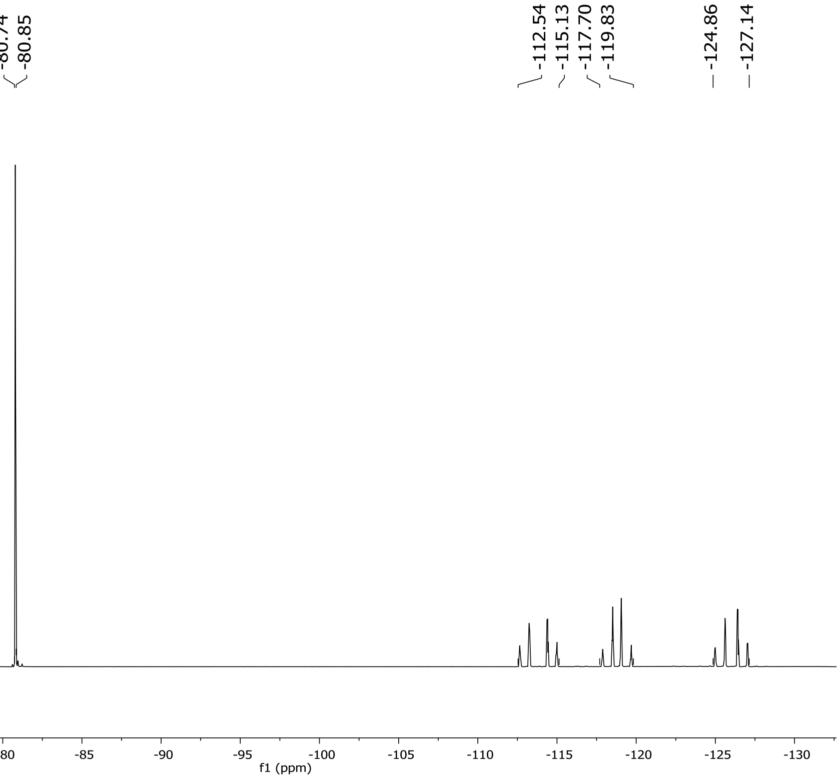

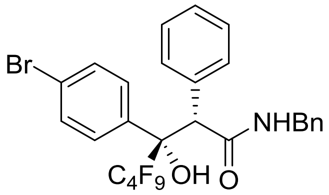

60

^{1}H, $CDCl_3$, 500 MHz

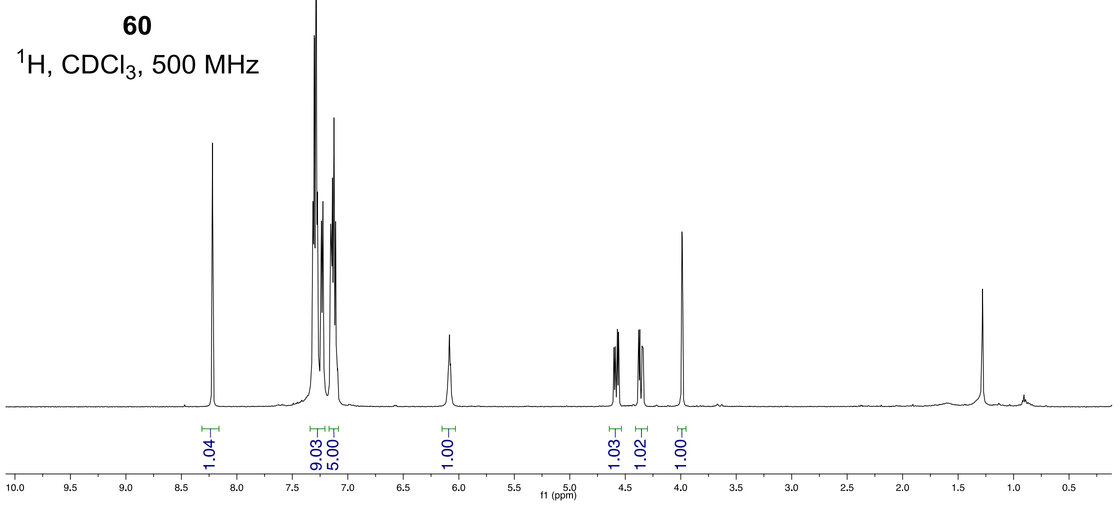

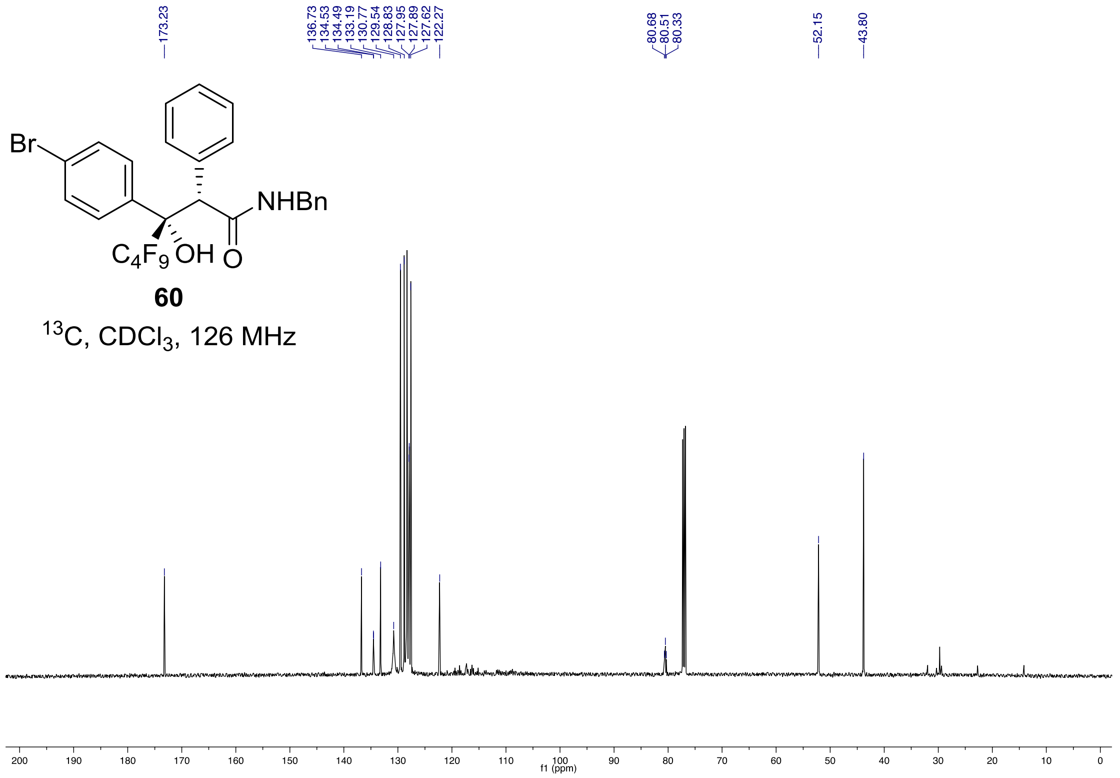

60

^{13}C, $CDCl_3$, 126 MHz

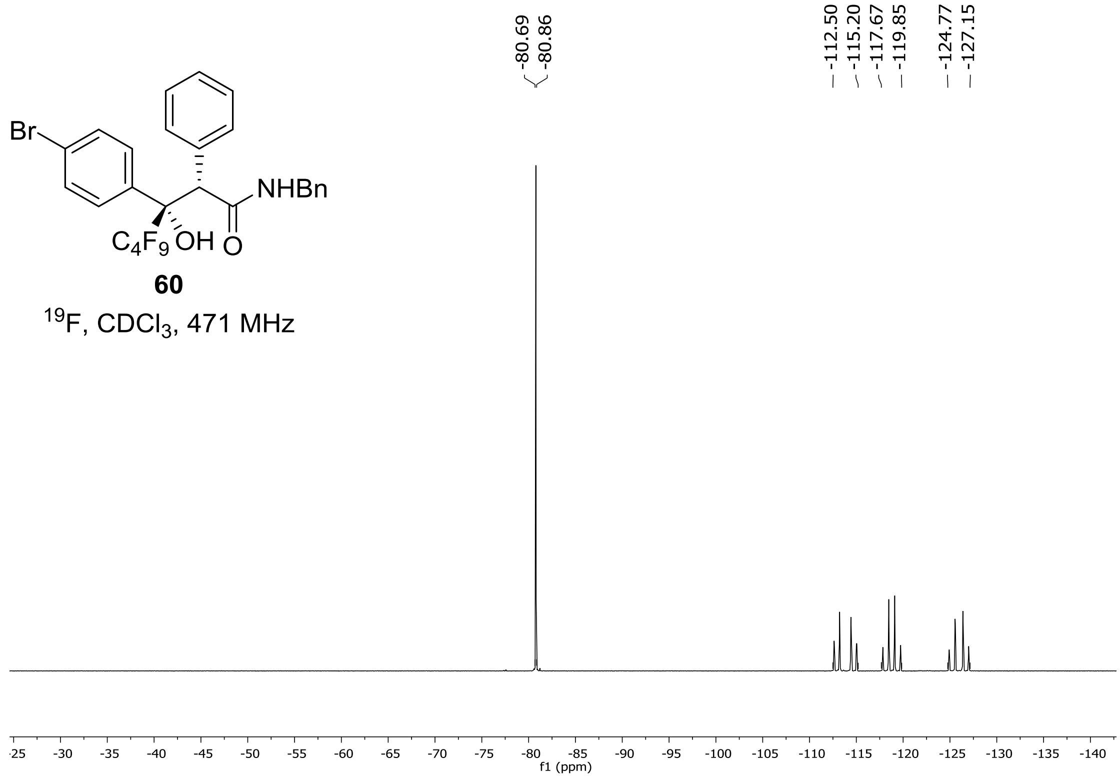

60

^{19}F, $CDCl_3$, 471 MHz

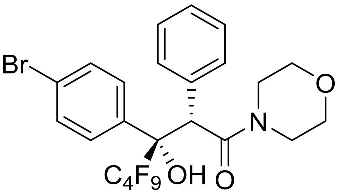

61

^1H, $CDCl_3$, 500 MHz

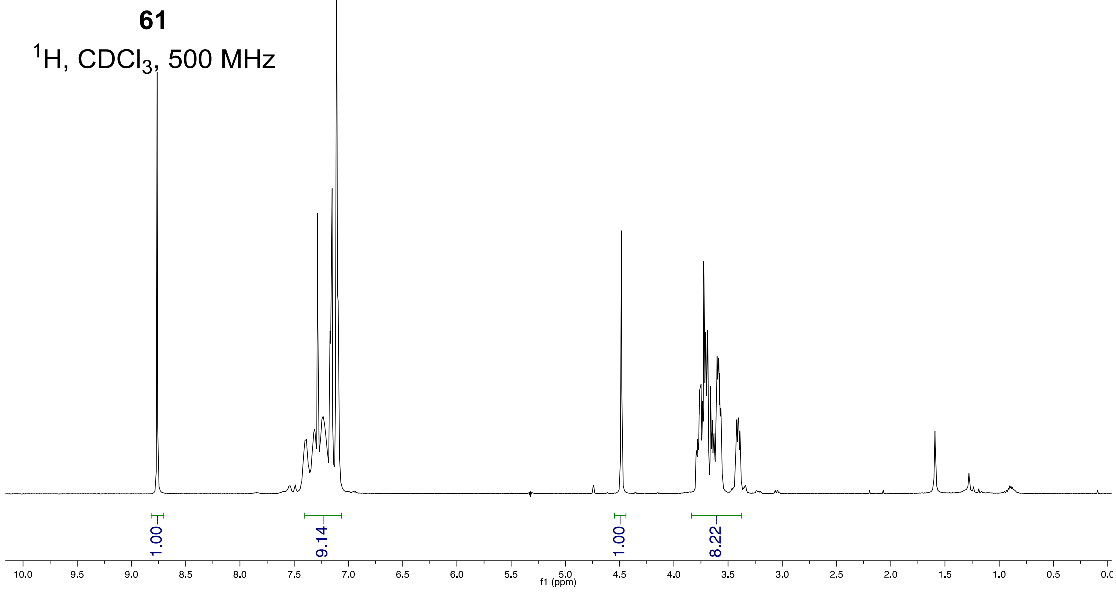

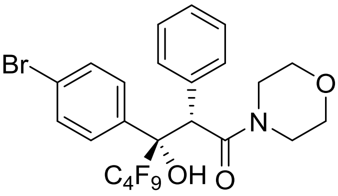

61

^{13}C, $CDCl_3$, 126 MHz

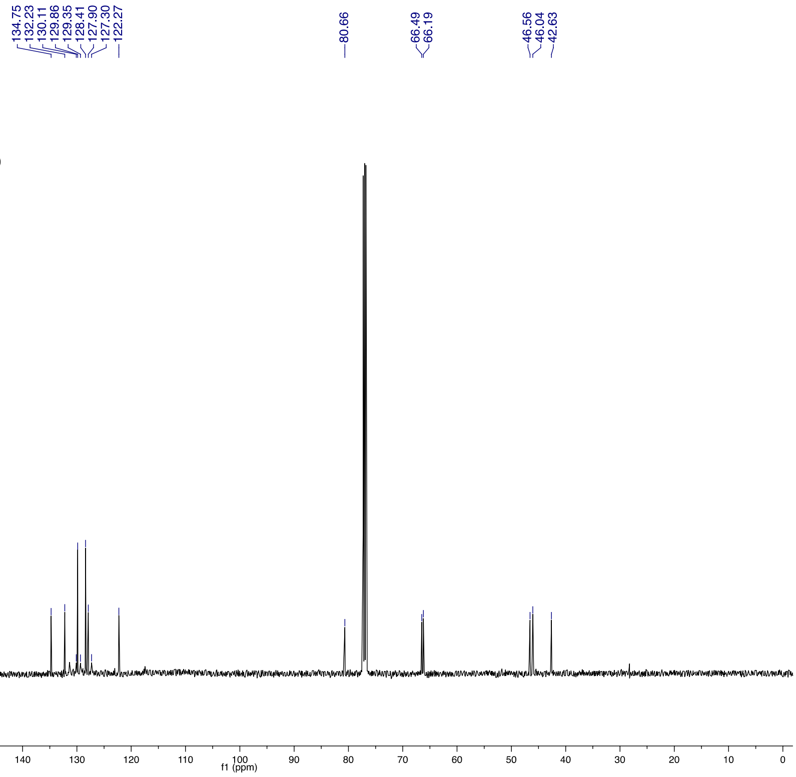

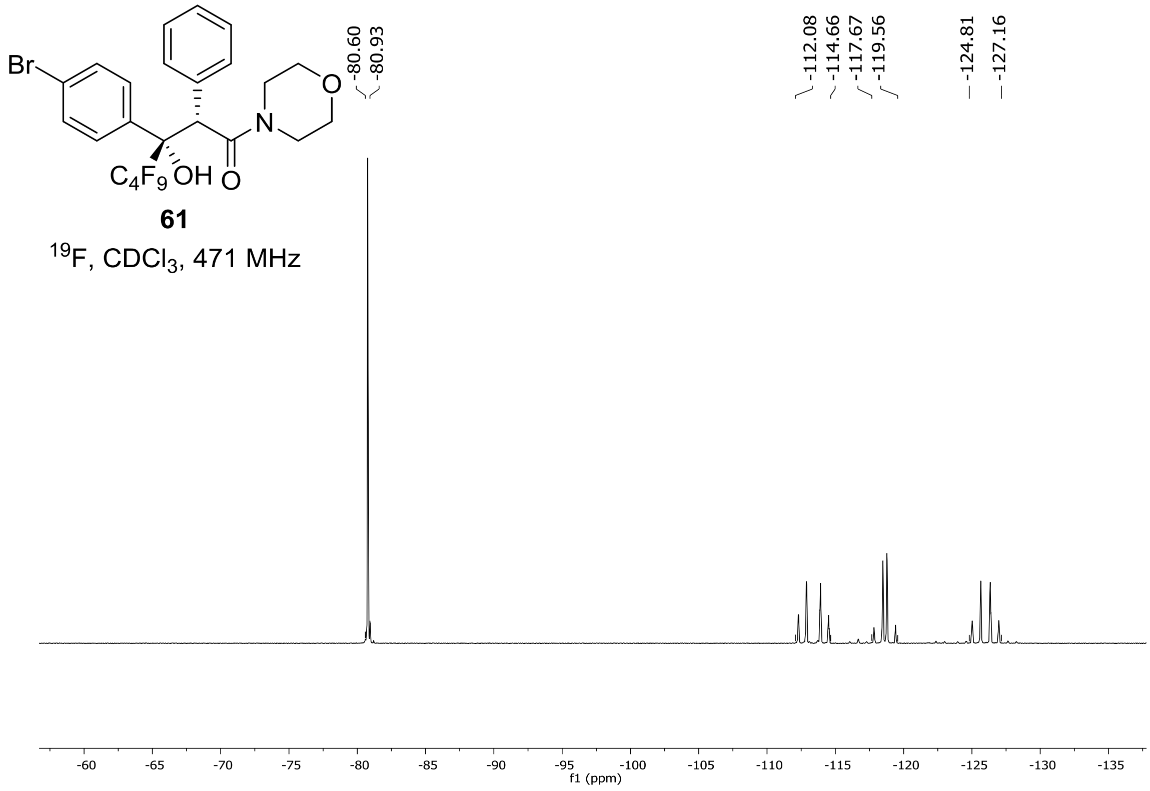

Br
C_4F_9 OH
O
N
O
61
^{19}F, CDCl$_3$, 471 MHz
-80.60
-80.93
-112.08
-114.66
-117.67
-119.56
-124.81
-127.16
f1 (ppm)

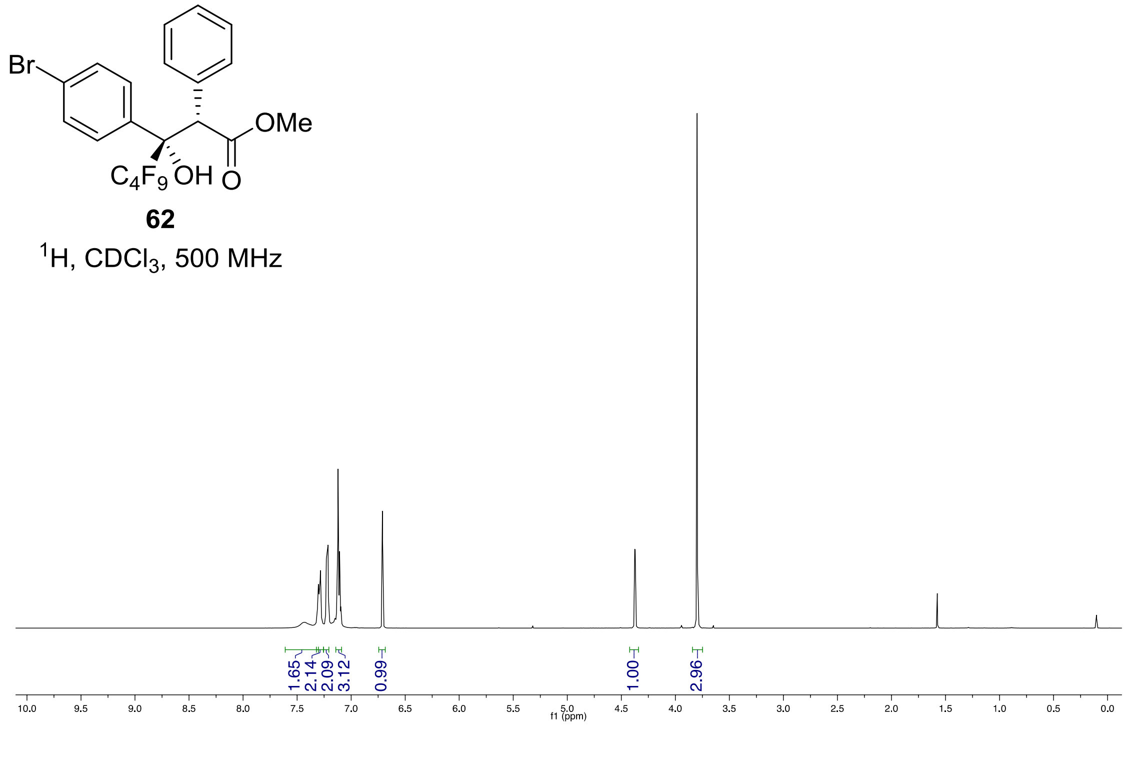

Br
OMe
C_4F_9 OH
O
62
^{1}H, CDCl$_3$, 500 MHz
1.65
2.14
2.09
3.12
0.99
1.00
2.96
f1 (ppm)

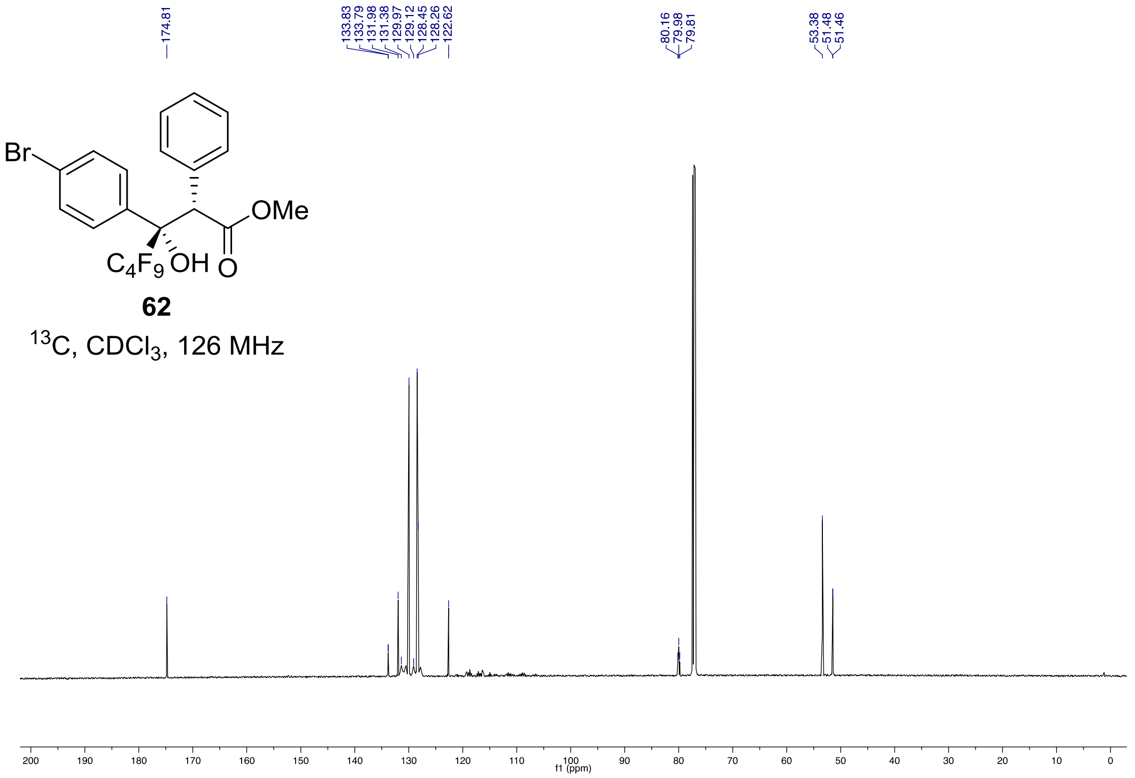
174.81
133.83
133.79
131.98
131.38
129.97
129.12
128.45
128.26
122.62
80.16
79.98
79.81
53.38
51.48
51.46
Br
OMe
C4F9 OH O
62
13C, CDCl3, 126 MHz
f1 (ppm)

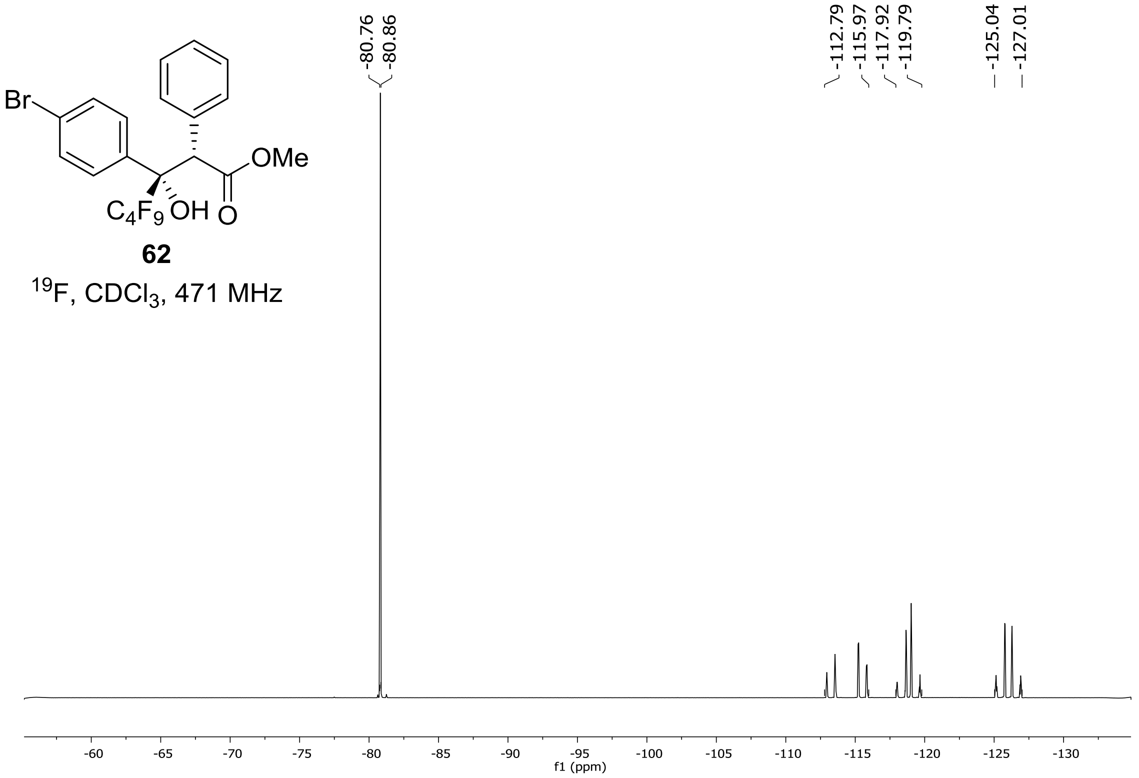
-80.76
-80.86
-112.79
-115.97
-117.92
-119.79
-125.04
-127.01
Br
OMe
C4F9 OH O
62
19F, CDCl3, 471 MHz
f1 (ppm)

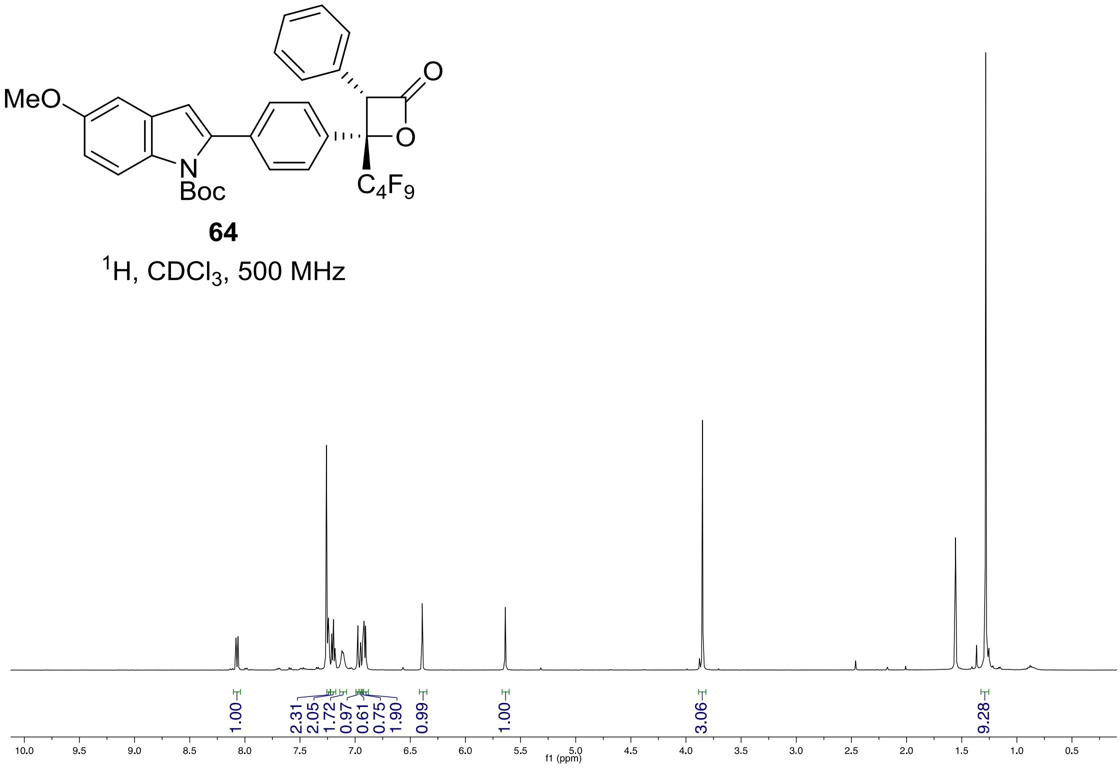

64

^1H, $CDCl_3$, 500 MHz

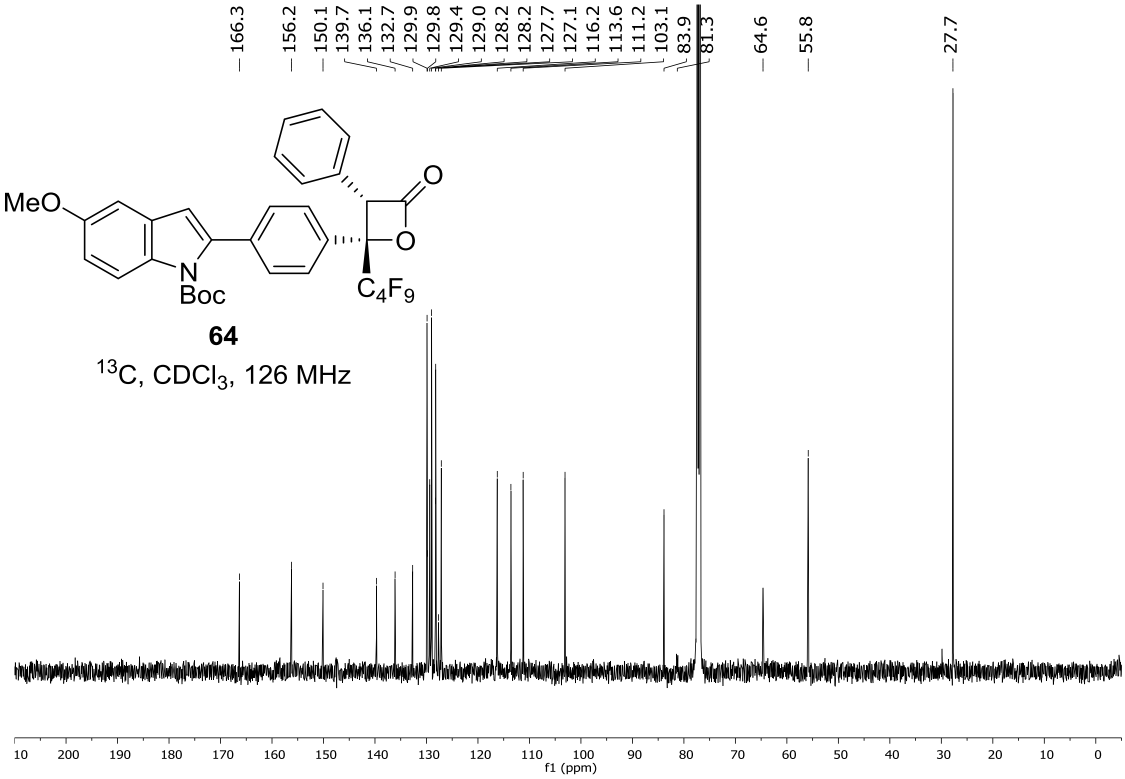

64

^{13}C, $CDCl_3$, 126 MHz

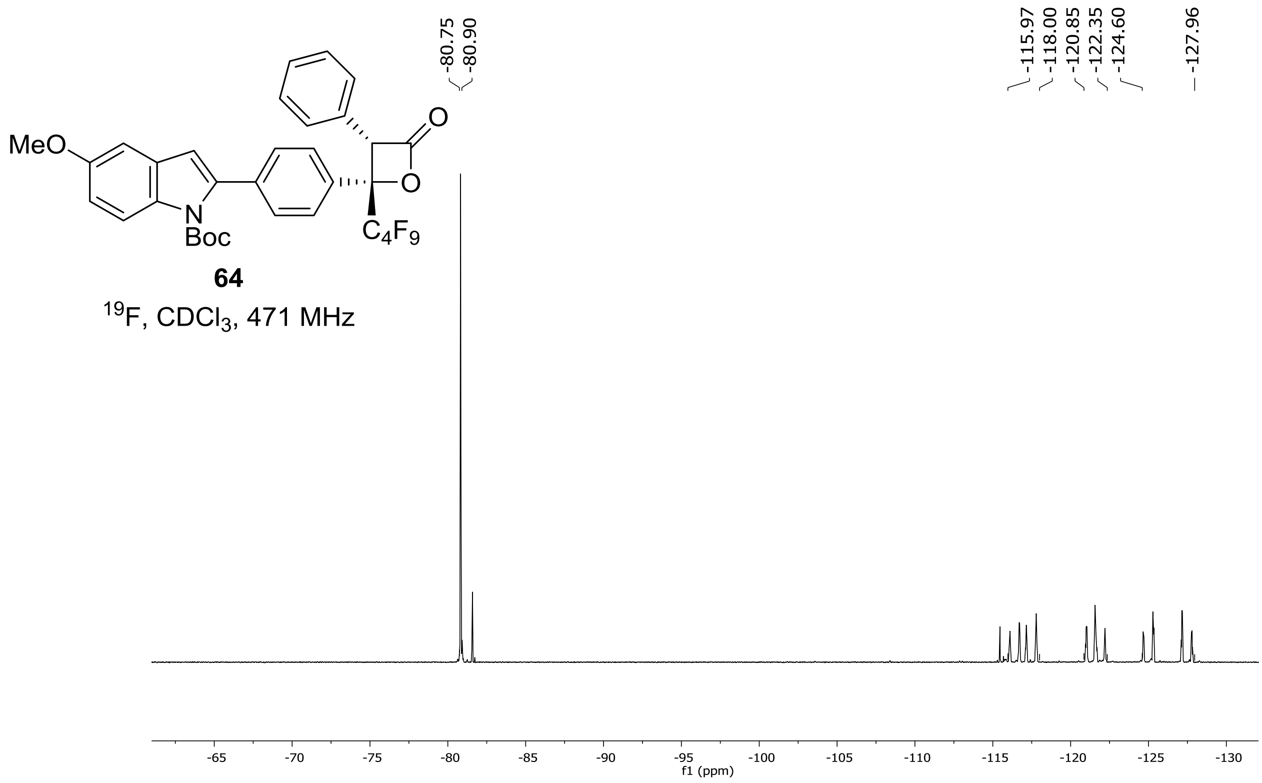

MeO
N
Boc
O
O
C_4F_9
64
^{19}F, $CDCl_3$, 471 MHz
-80.75
-80.90
-115.97
-118.00
-120.85
-122.35
-124.60
-127.96
f1 (ppm)

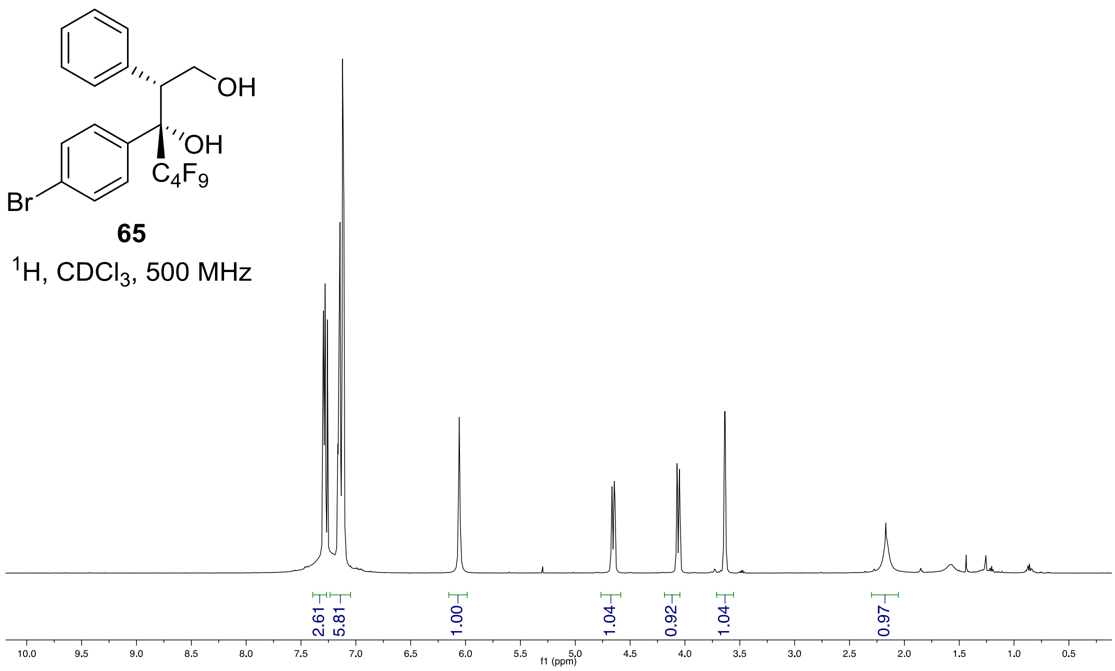

OH
OH
C_4F_9
Br
65
^{1}H, $CDCl_3$, 500 MHz
2.61
5.81
1.00
1.04
0.92
1.04
0.97
f1 (ppm)

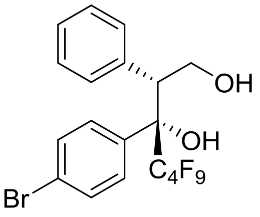

65

^{13}C, $CDCl_3$, 126 MHz

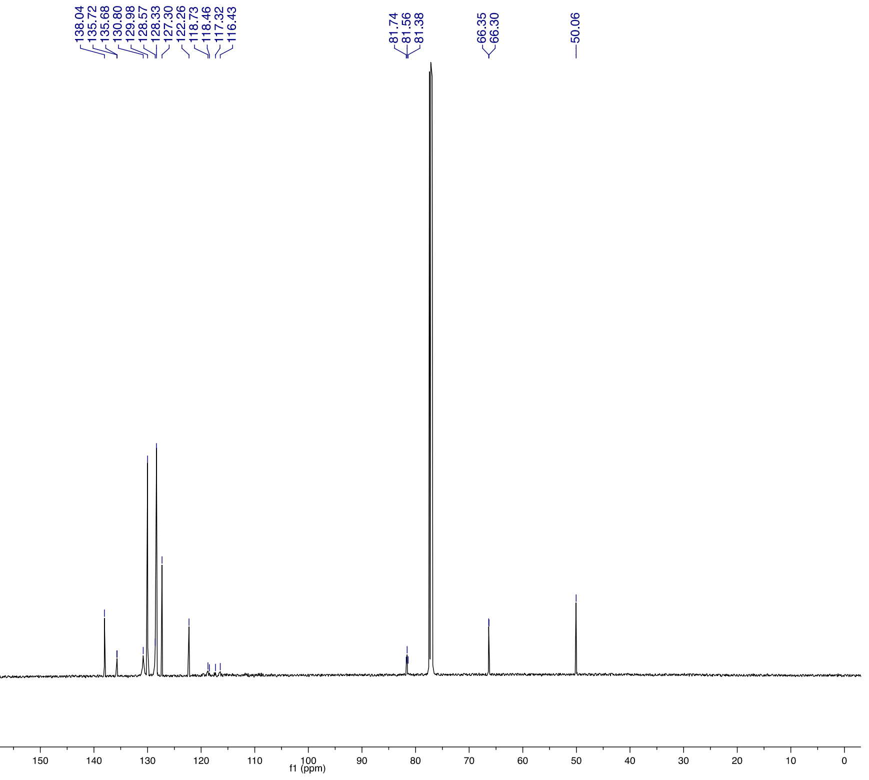

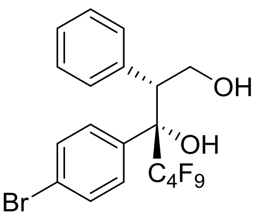

65

^{19}F, $CDCl_3$, 471 MHz

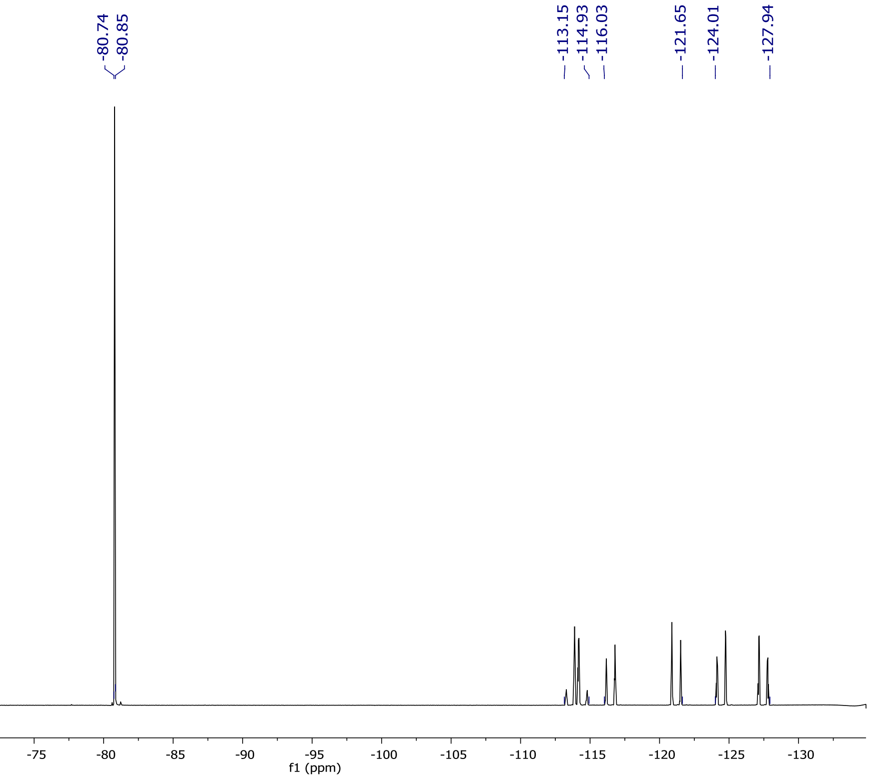

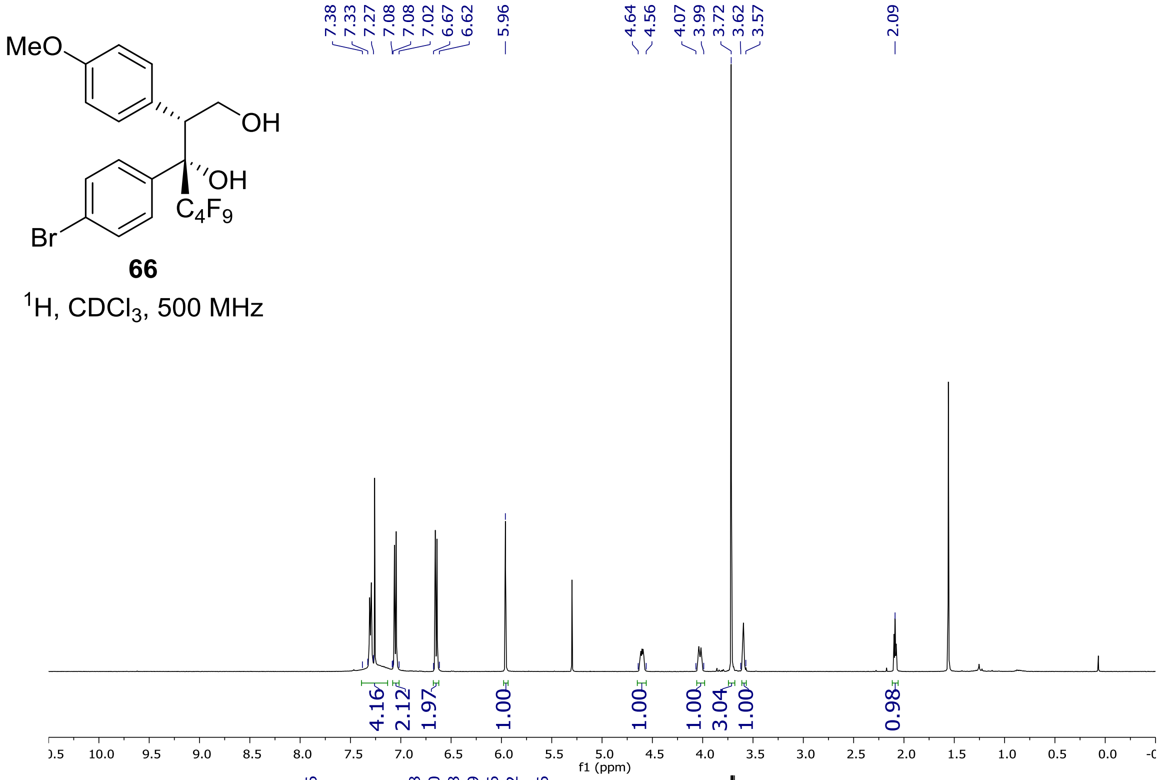

MeO
OH
OH
C_4F_9
Br
66
^{1}H, CDCl$_3$, 500 MHz
7.38 7.33 7.27 7.08 7.08 7.02 6.67 6.62 5.96 4.64 4.56 4.07 3.99 3.72 3.62 3.57 2.09
4.16 2.12 1.97 1.00 1.00 1.00 3.04 1.00 0.98
f1 (ppm)

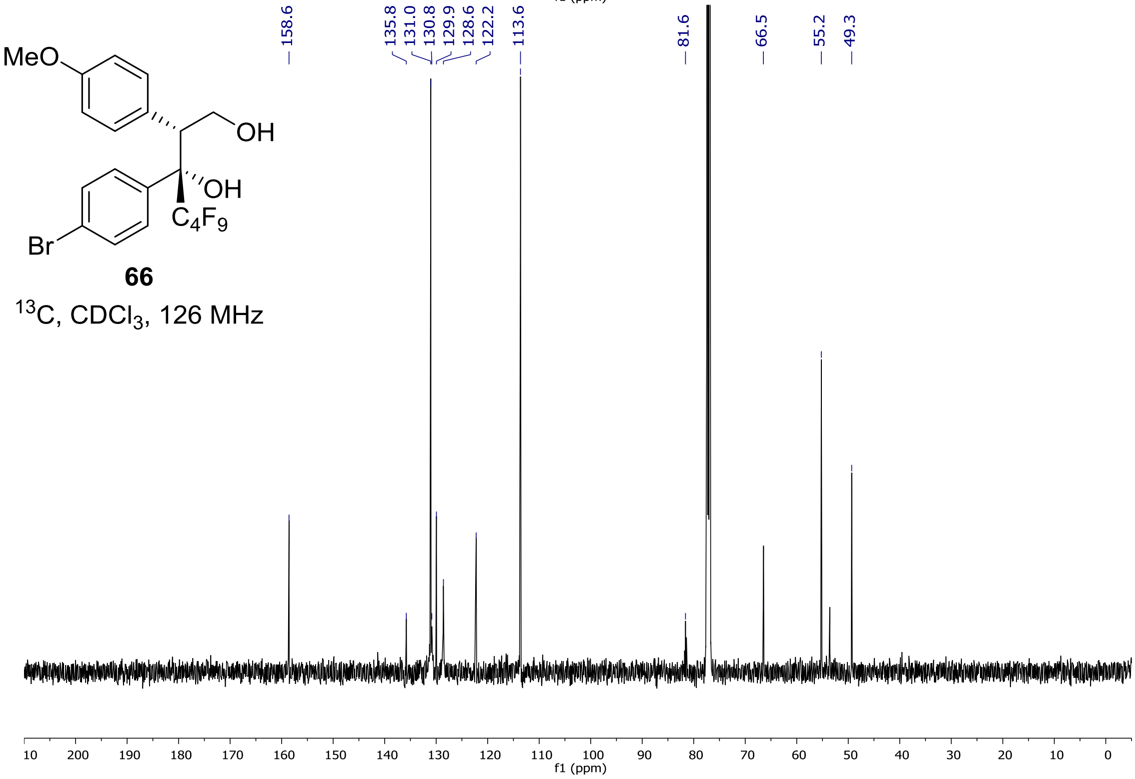

MeO
OH
OH
C_4F_9
Br
66
^{13}C, CDCl$_3$, 126 MHz
158.6 135.8 131.0 130.8 129.9 128.6 122.2 113.6 81.6 66.5 55.2 49.3
f1 (ppm)

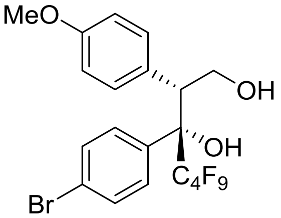

66

^{19}F, $CDCl_3$, 471 MHz

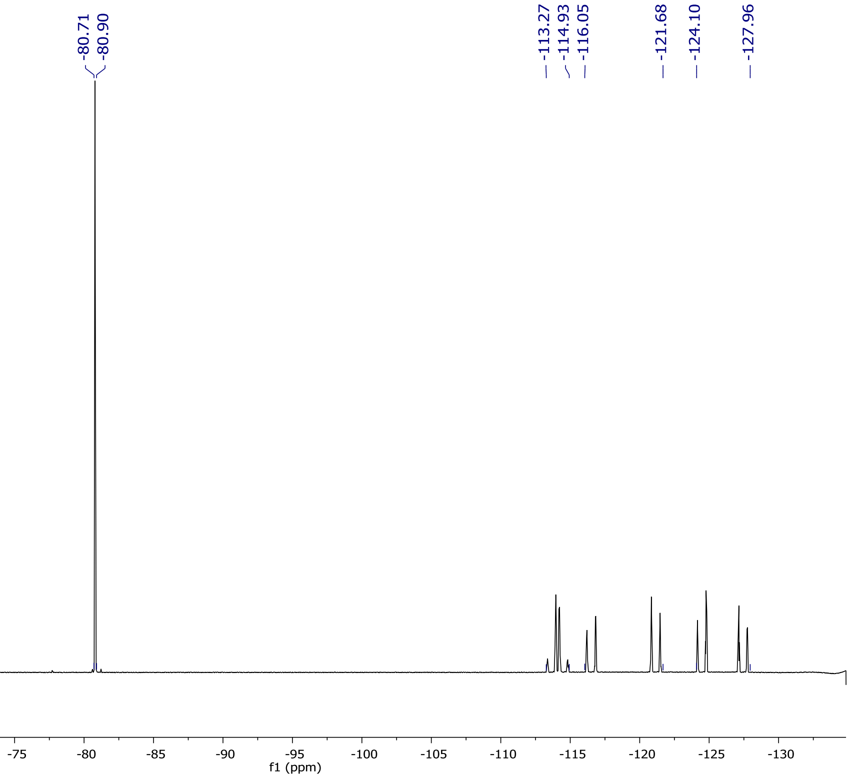

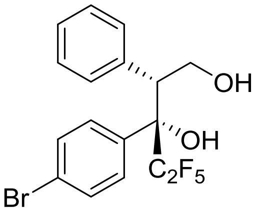

67

^{1}H, $CDCl_3$, 500 MHz

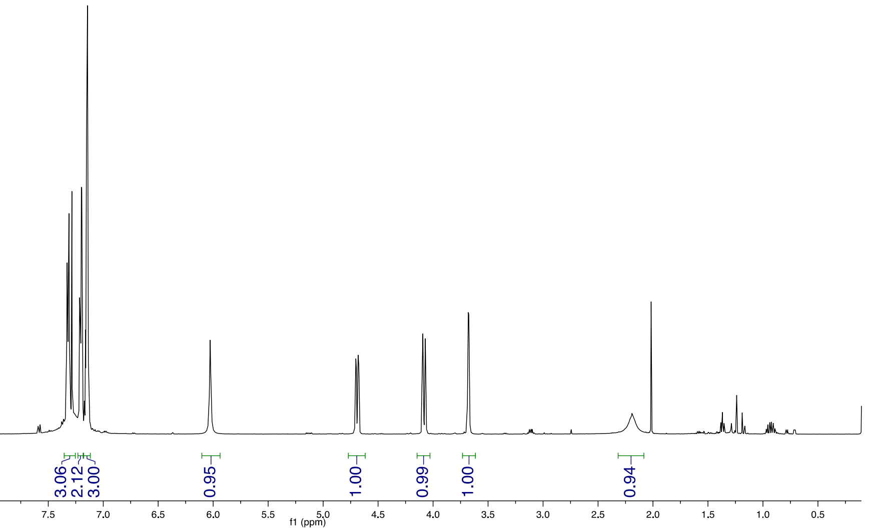

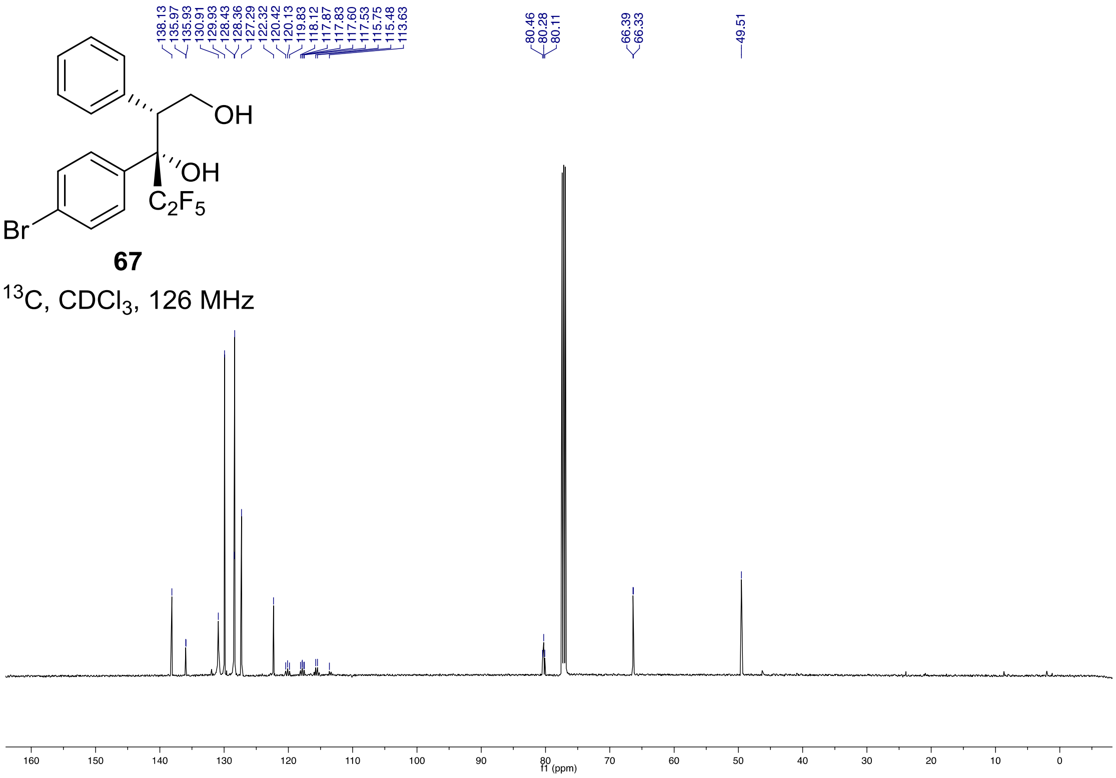

67

^{13}C, $CDCl_3$, 126 MHz

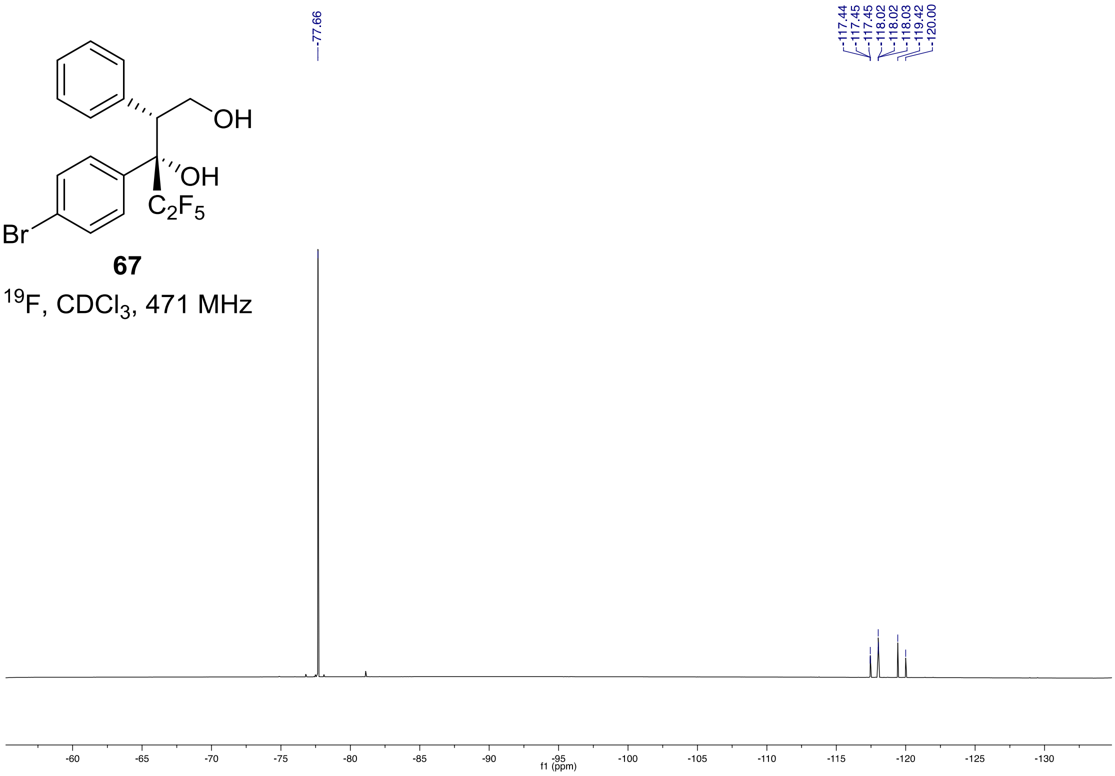

67

^{19}F, $CDCl_3$, 471 MHz

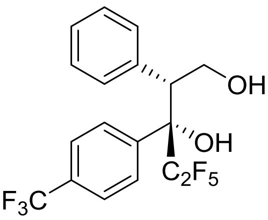

68

^1H, $CDCl_3$, 500 MHz

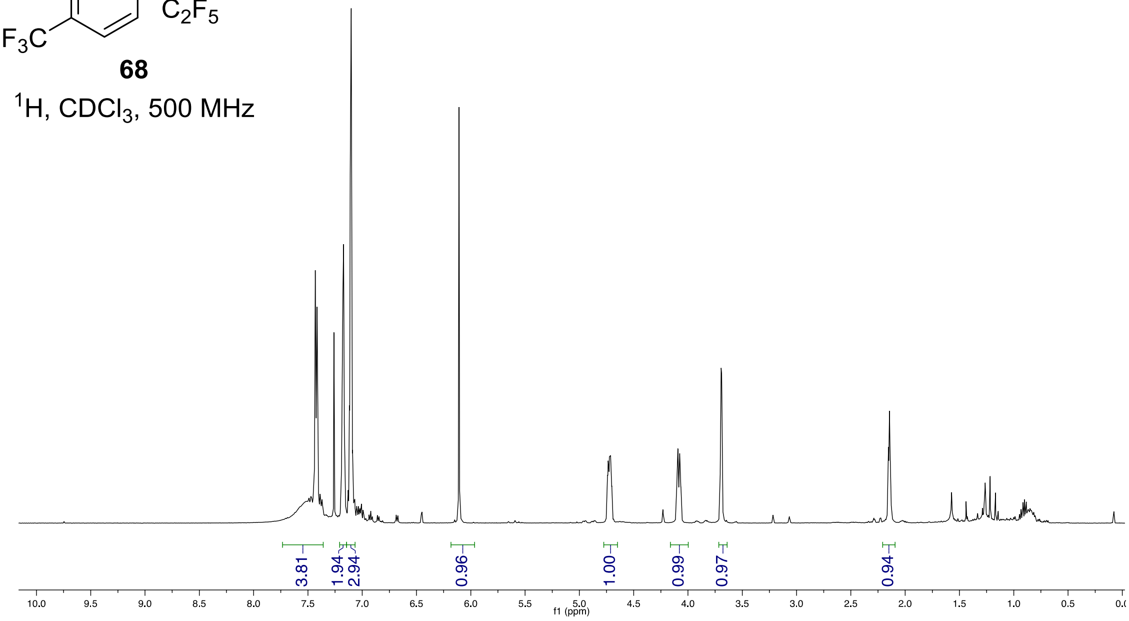

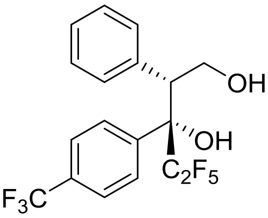

68

^{13}C, $CDCl_3$, 126 MHz

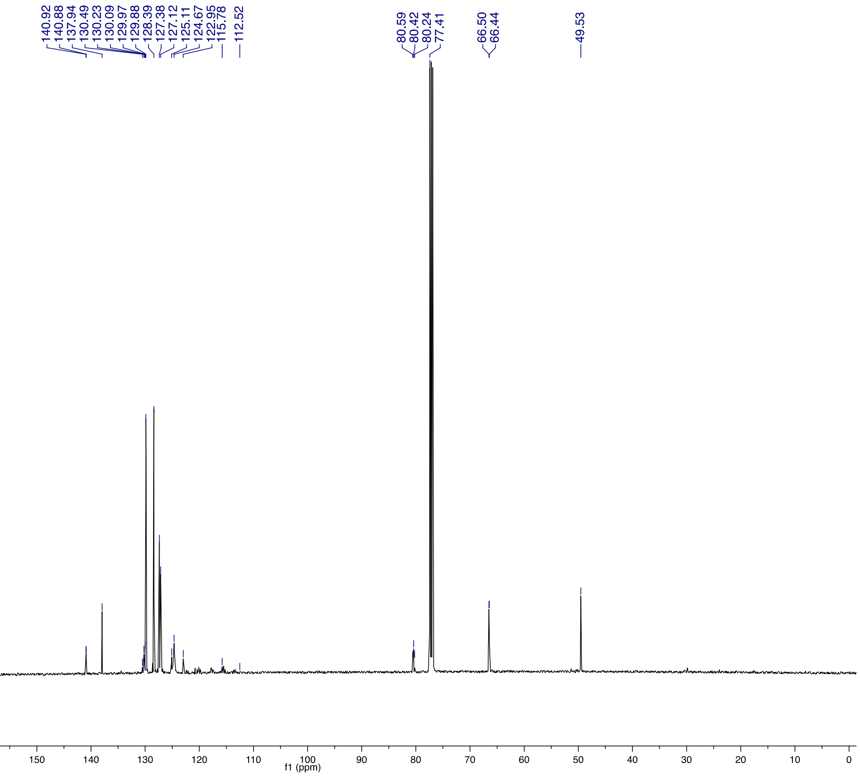

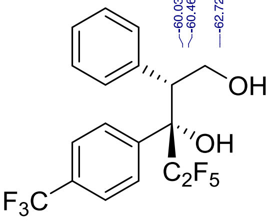

68

^{19}F, $CDCl_3$, 471 MHz

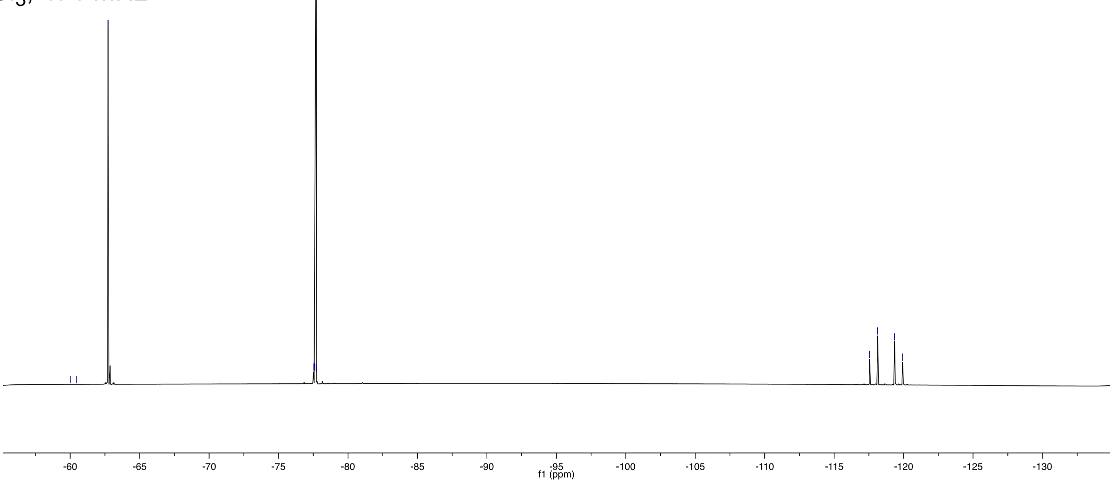

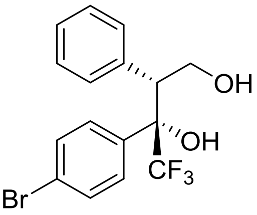

69

^1H, $CDCl_3$, 500 MHz

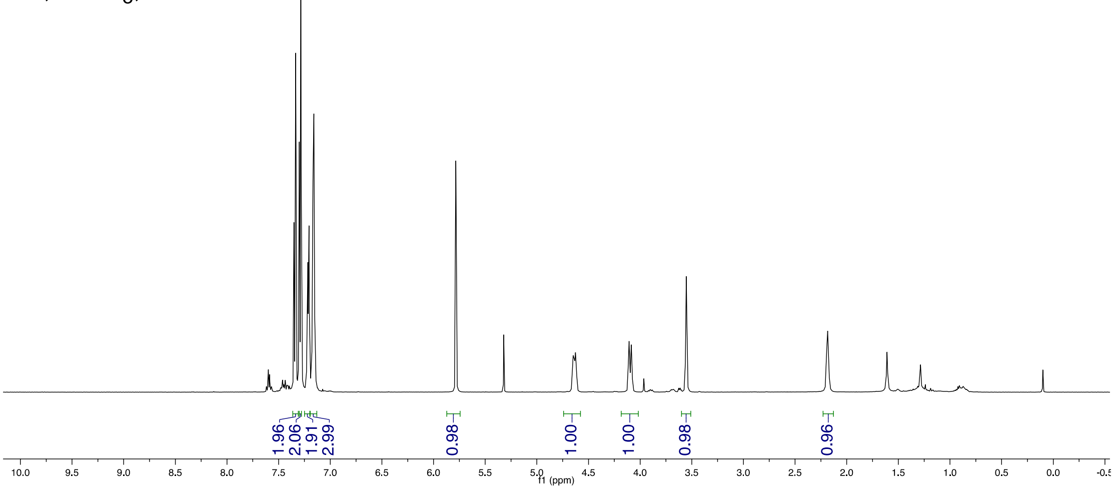

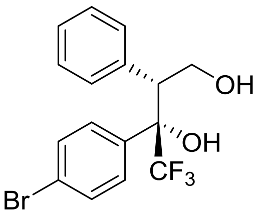

69

^{13}C, $CDCl_3$, 126 MHz

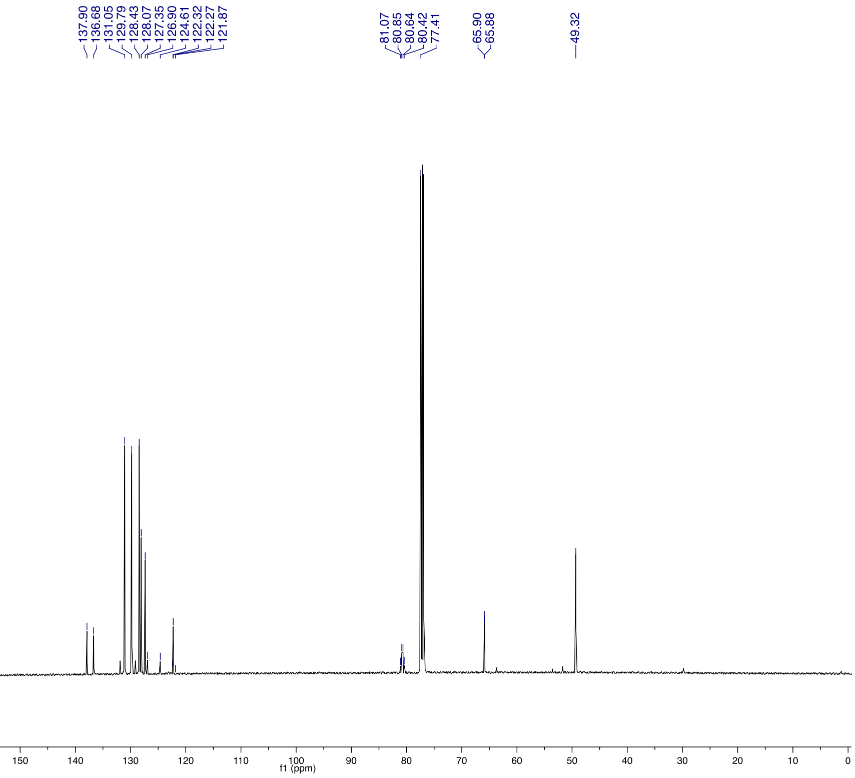

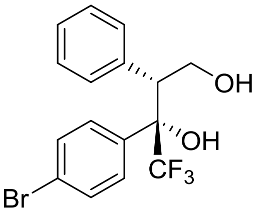

69

^{19}F, $CDCl_3$, 471 MHz

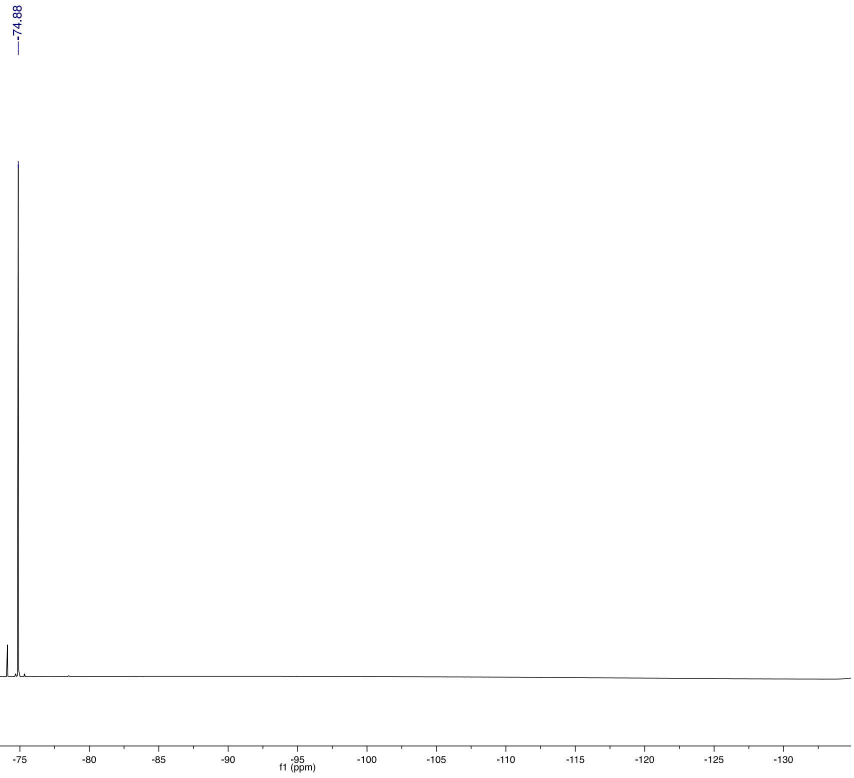

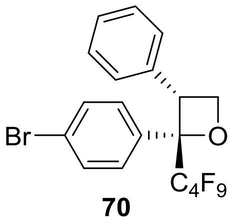

70

^1H, $CDCl_3$, 500 MHz

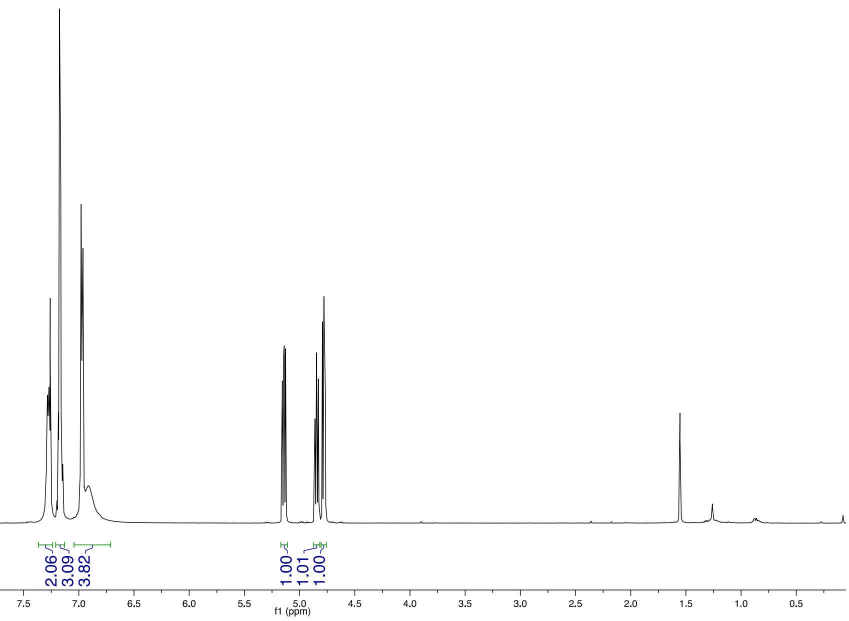

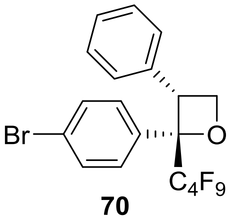

70

^{13}C, $CDCl_3$, 126 MHz

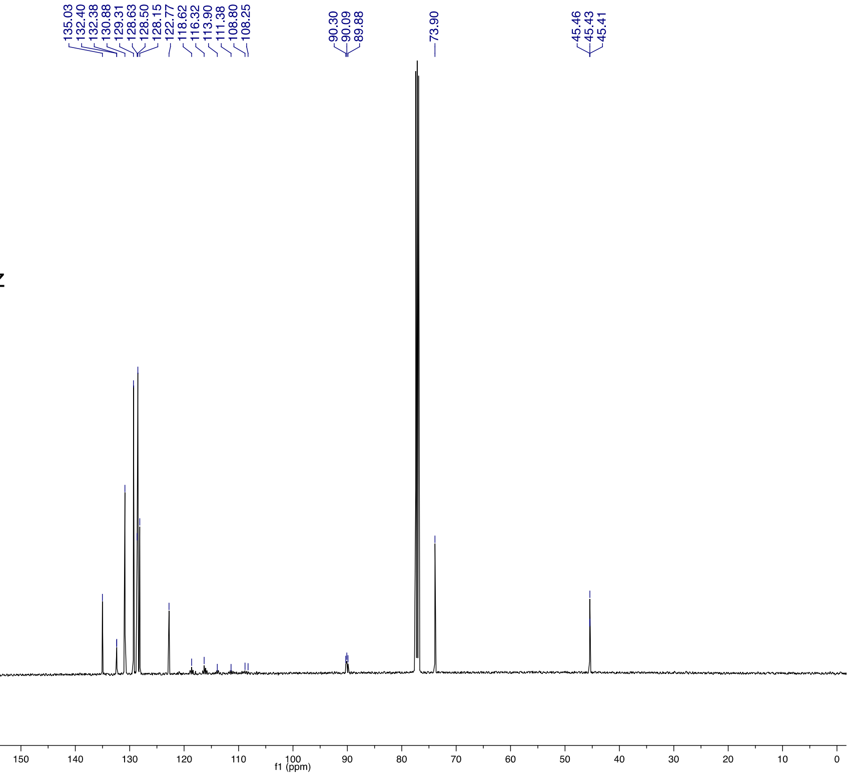

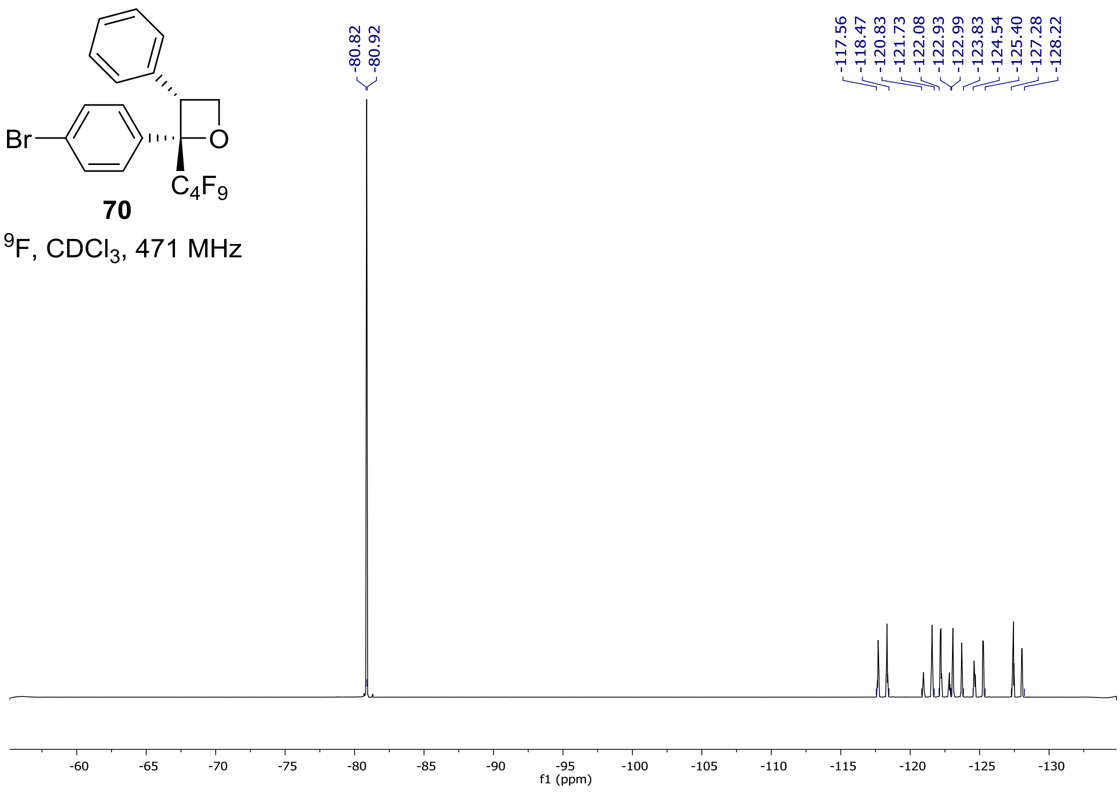

Br
O
C_4F_9
70
^{19}F, $CDCl_3$, 471 MHz
-80.82
-80.92
-117.56
-118.47
-120.83
-121.73
-122.08
-122.93
-122.99
-123.83
-124.54
-125.40
-127.28
-128.22
f1 (ppm)

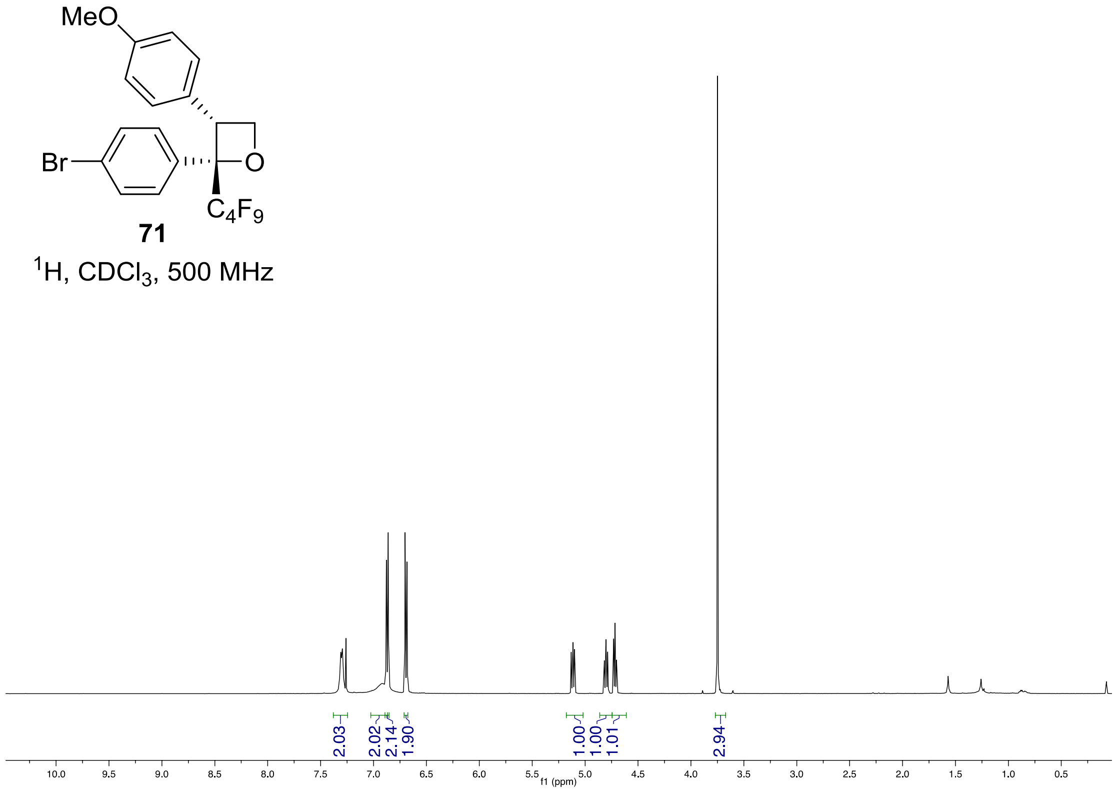

MeO
Br
O
C_4F_9
71
^{1}H, $CDCl_3$, 500 MHz
2.03
2.02
2.14
1.90
1.00
1.00
1.01
2.94
f1 (ppm)

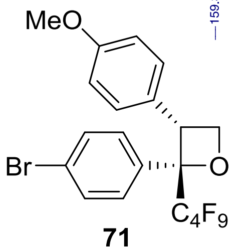

71

^{13}C, $CDCl_3$, 500 MHz

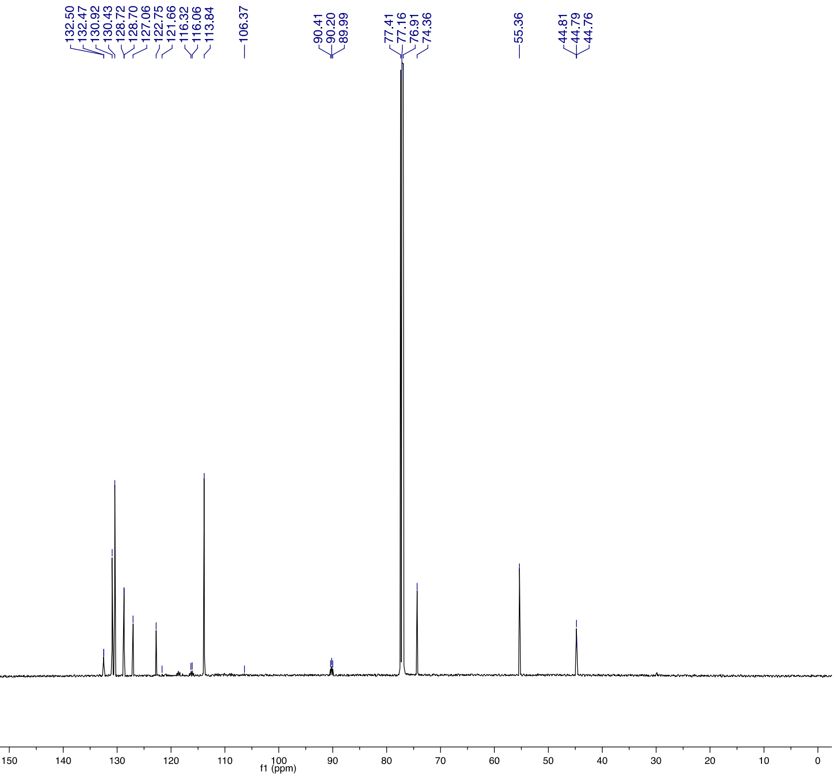

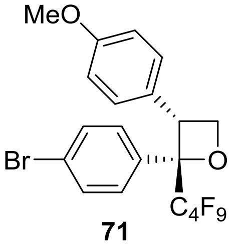

71

^{19}F, $CDCl_3$, 471 MHz

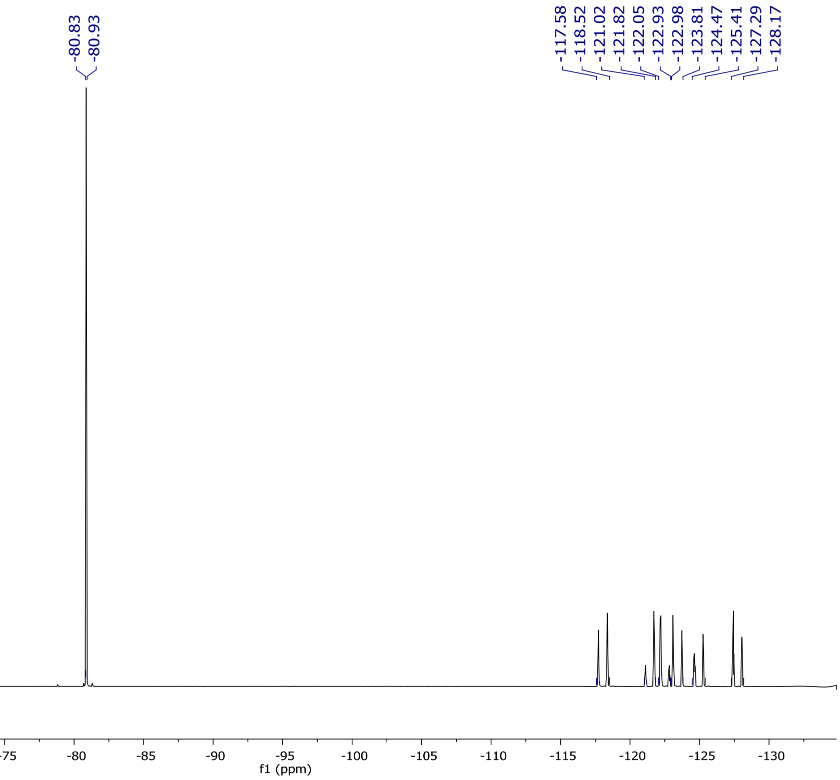

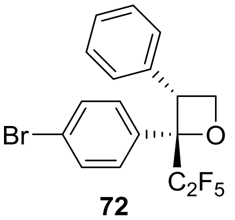

72

^1H, $CDCl_3$, 500 MHz

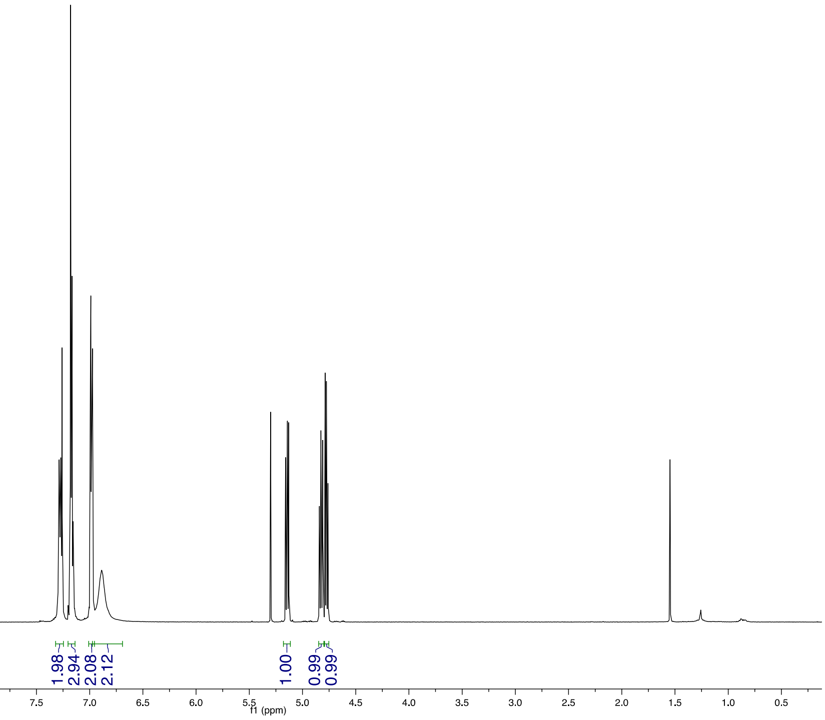

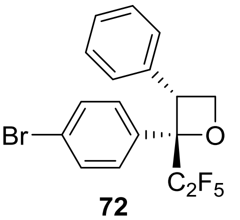

72

^{13}C, $CDCl_3$, 126 MHz

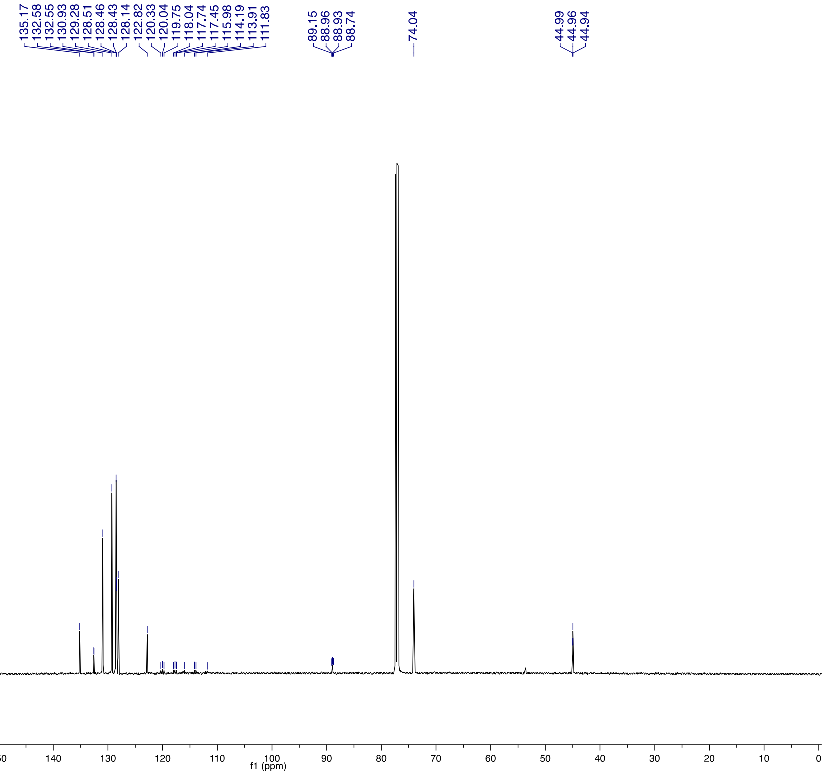

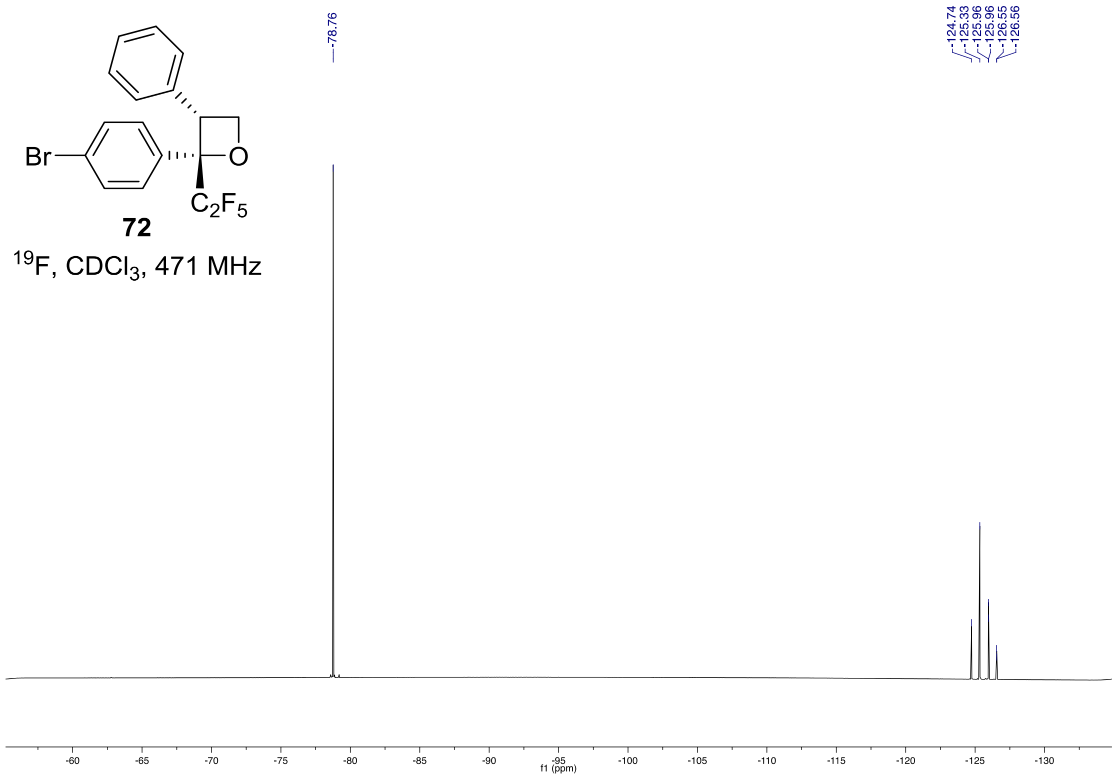
Br
O
C2F5
72
19F, CDCl3, 471 MHz
-78.76
-124.74
-125.33
-125.96
-125.96
-125.96
-126.55
-126.56
f1 (ppm)

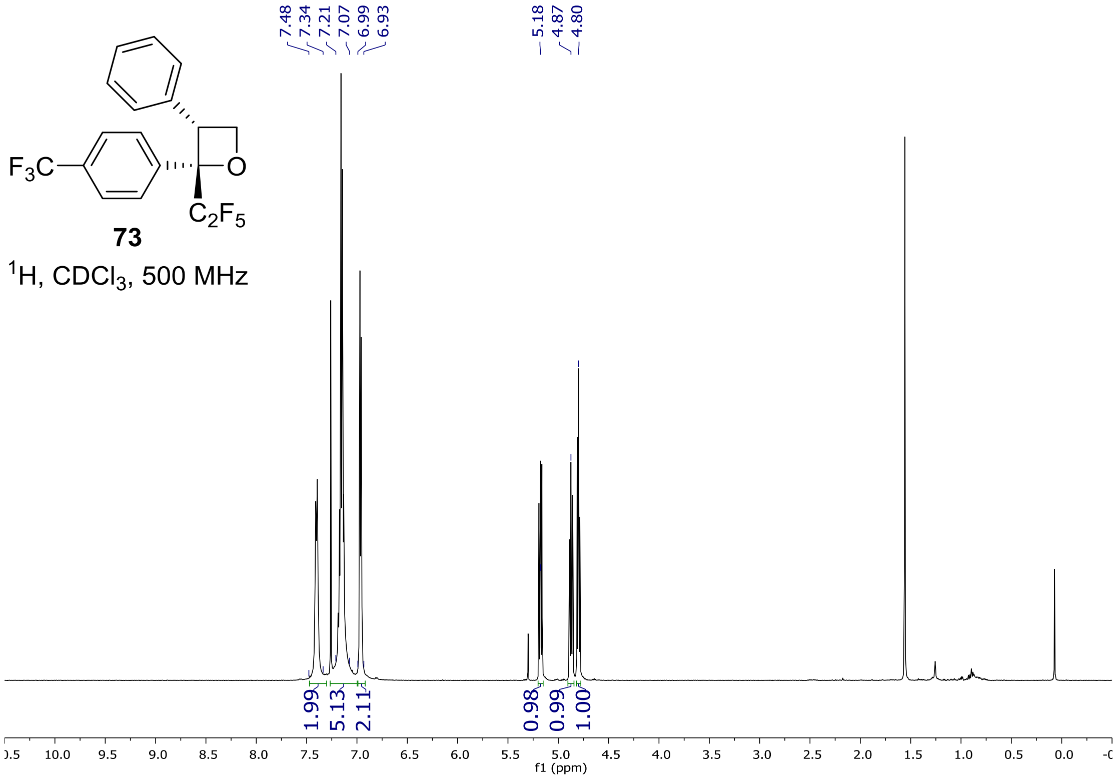
F3C
O
C2F5
73
1H, CDCl3, 500 MHz
7.48
7.34
7.21
7.07
6.99
6.93
5.18
4.87
4.80
1.99
5.13
2.11
0.98
0.99
1.00
f1 (ppm)

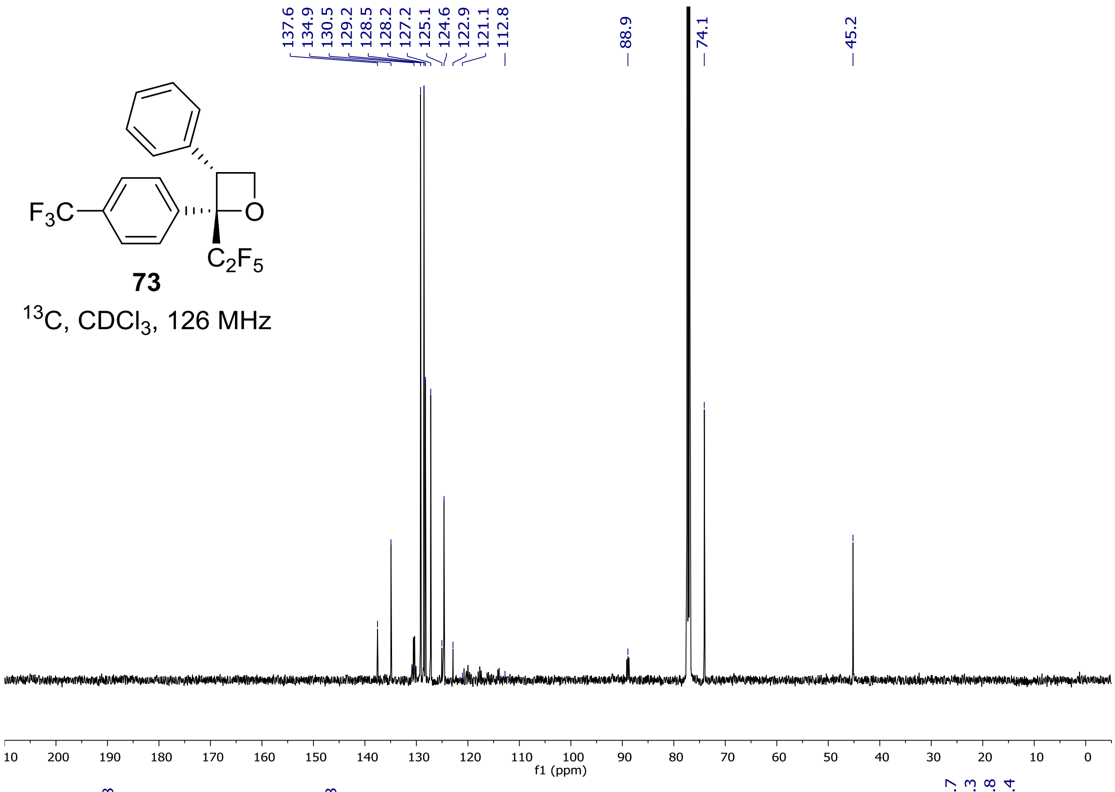
137.6
134.9
130.5
129.2
128.5
128.2
127.2
125.1
124.6
122.9
121.1
112.8
88.9
74.1
45.2
F3C
O
C2F5
73
13C, CDCl3, 126 MHz
f1 (ppm)

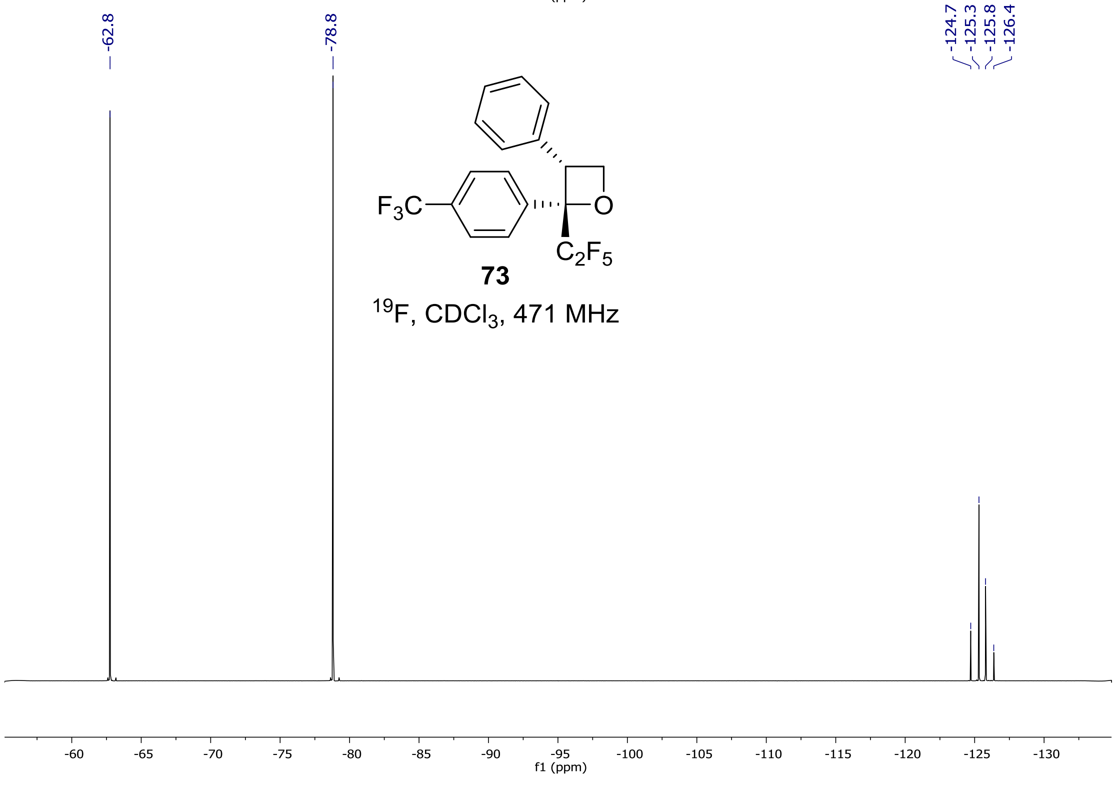
-62.8
-78.8
-124.7
-125.3
-125.8
-126.4
F3C
O
C2F5
73
19F, CDCl3, 471 MHz
f1 (ppm)

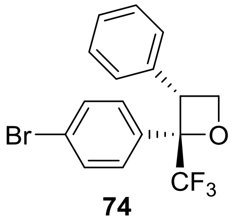

74

^{1}H, $CDCl_3$, 500 MHz

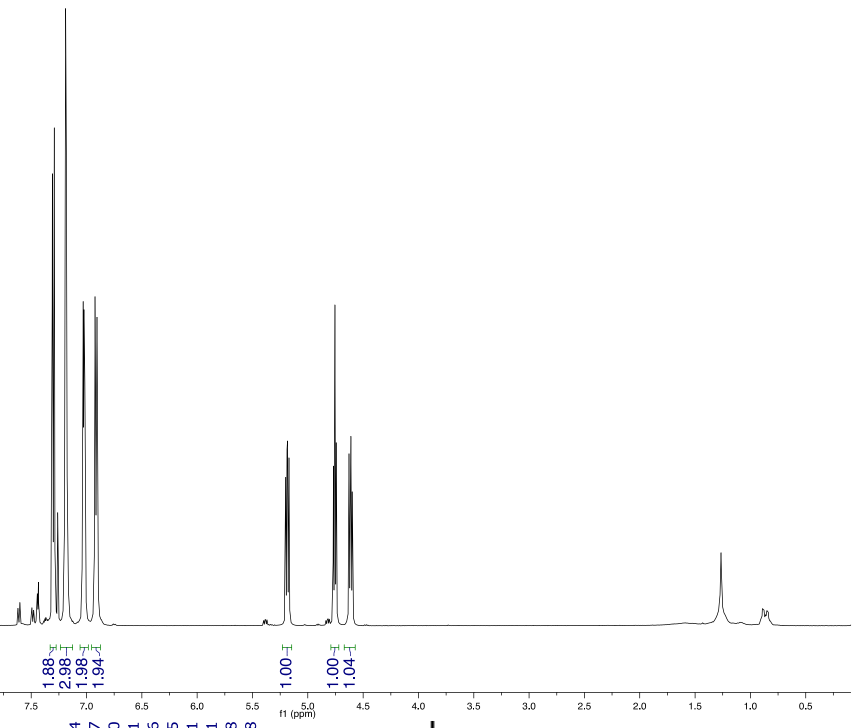

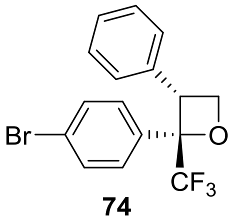

74

^{13}C, $CDCl_3$, 126 MHz

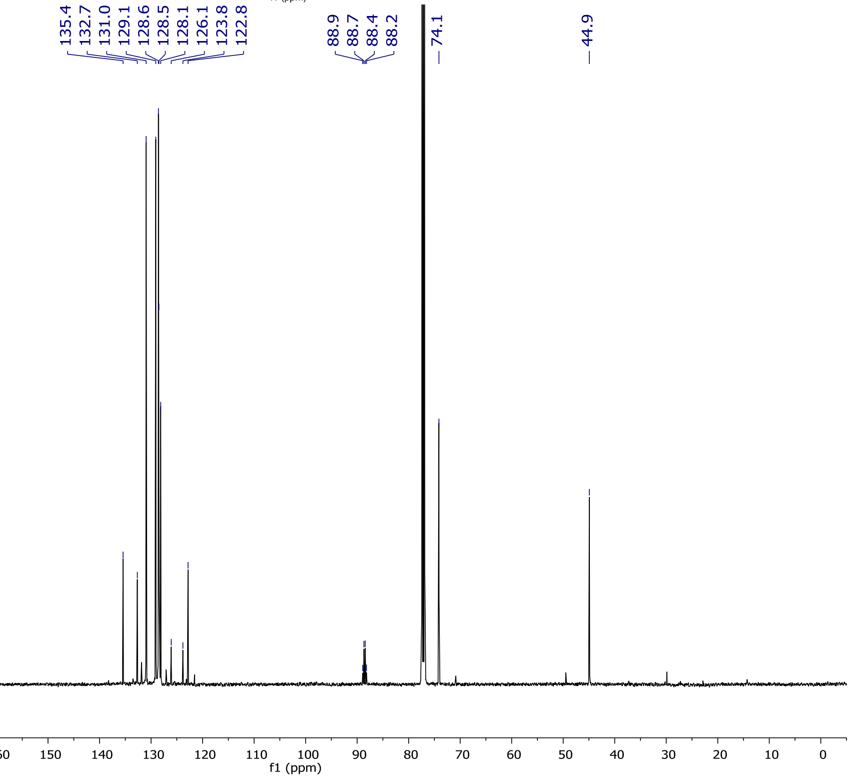

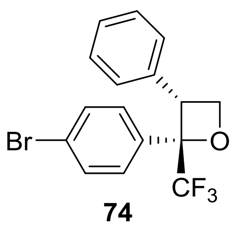

74

^{19}F, $CDCl_3$, 471 MHz

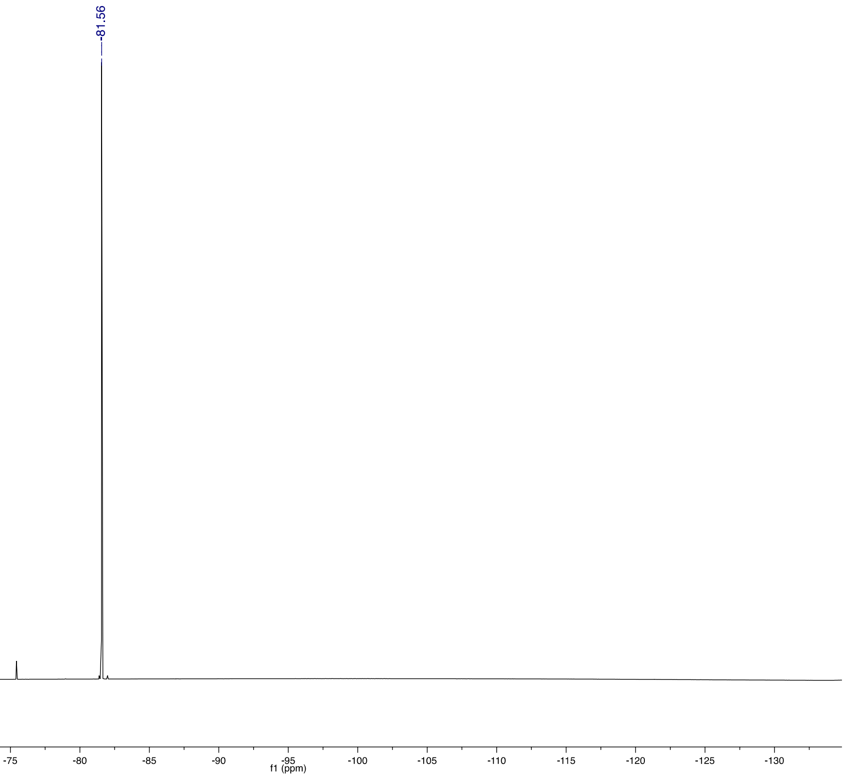

HPLC Spectra

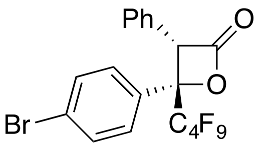

Chiralcel OD-H (97:3 hexane:IPA, flow rate 1.5 mLmin^{-1}, 211 nm, 40 °C) t_R(3*R*,4*R*): 2.8 min, t_R(3*S*,4*S*): 3.4 min, 97:3 er.

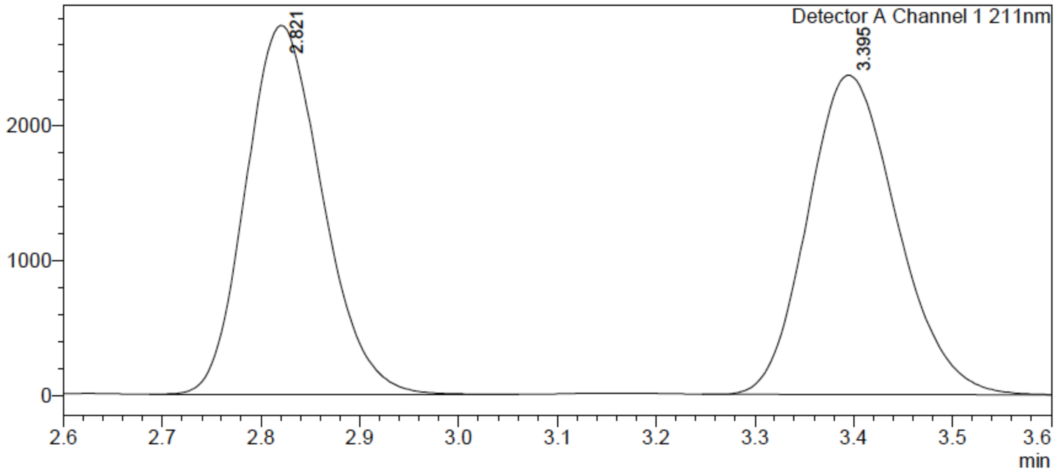

<Peak Table>

Detector A Channel 1 211nm

Peak#	Ret. Time	Area%
1	2.821	49.831
2	3.395	50.169
Total		100.000

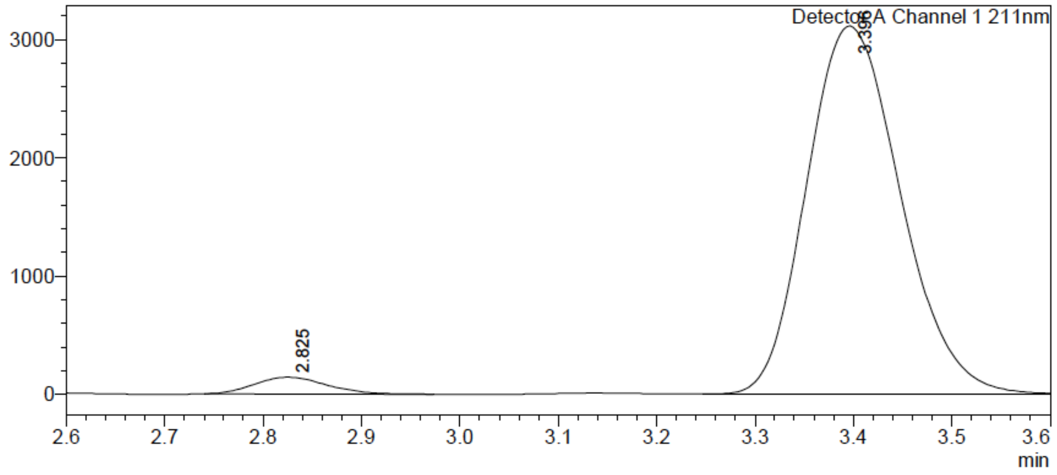

<Peak Table>

Detector A Channel 1 211nm

Peak#	Ret. Time	Area%
1	2.825	3.402
2	3.396	96.598
Total		100.000

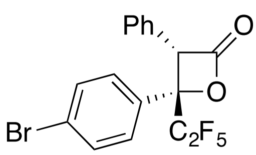

Chiralcel OD-H (97:3 Hexane:IPA, flow rate 1.5 mLmin^{-1}, 211 nm, 40 °C) t_R(3*R*,4*R*): 3.0 min, t_R(3*S*,4*S*): 3.8 min, 97:3 er.

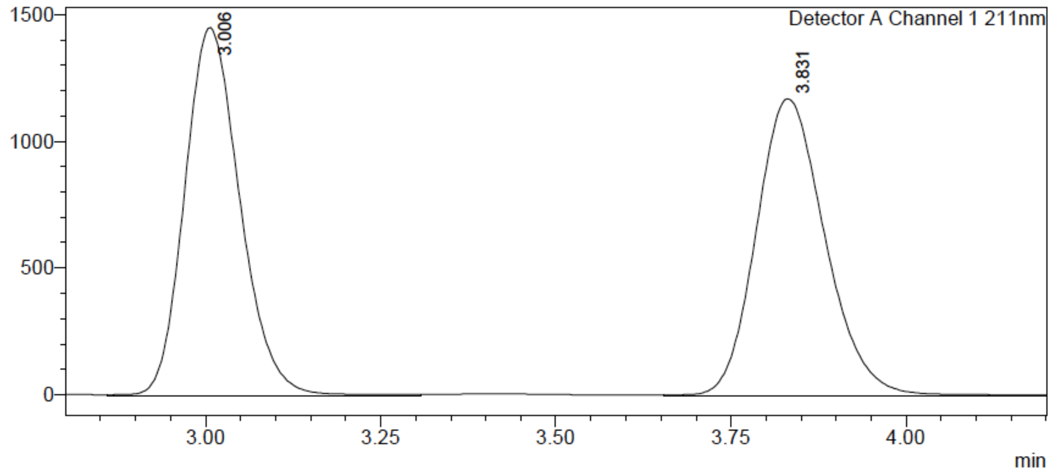

<Peak Table>

Detector A Channel 1 211nm

Peak#	Ret. Time	Area%
1	3.006	49.349
2	3.831	50.651
Total		100.000

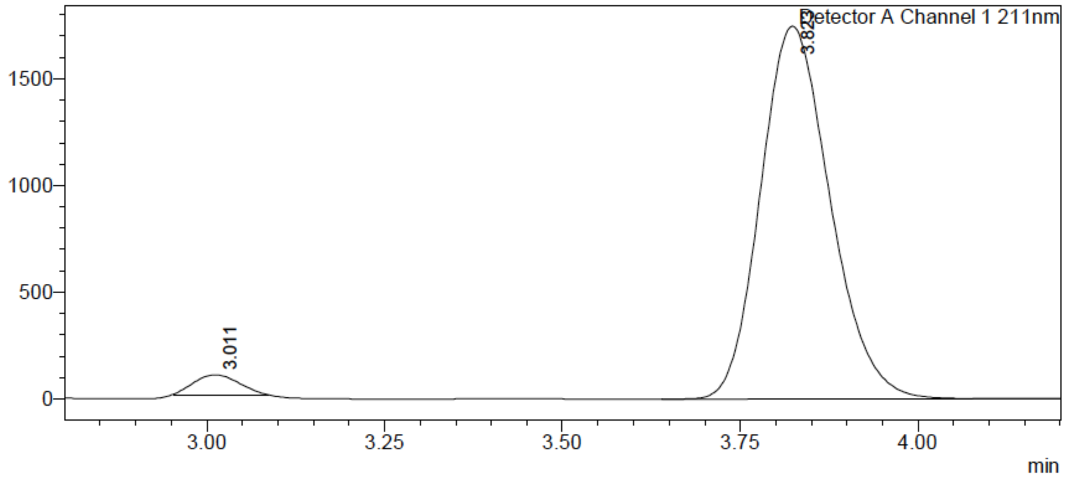

<Peak Table>

Detector A Channel 1 211nm

Peak#	Ret. Time	Area%
1	3.011	3.282
2	3.823	96.718
Total		100.000

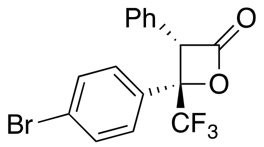

Chiralcel OD-H (97:3 Hexane:IPA, flow rate 1.5 mLmin^{-1}, 211 nm, 40 °C) t_R(3*R*,4*R*): 3.2 min, t_R(3*S*,4*S*): 4.1 min, 95:5 er;

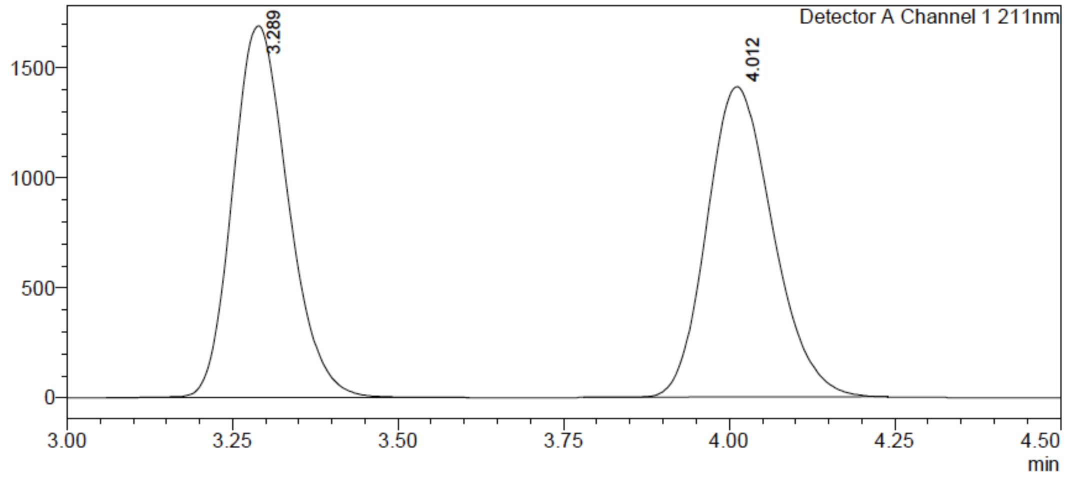

<Peak Table>

Detector A Channel 1 211nm

Peak#	Ret. Time	Area%
1	3.289	50.096
2	4.012	49.904
Total		100.000

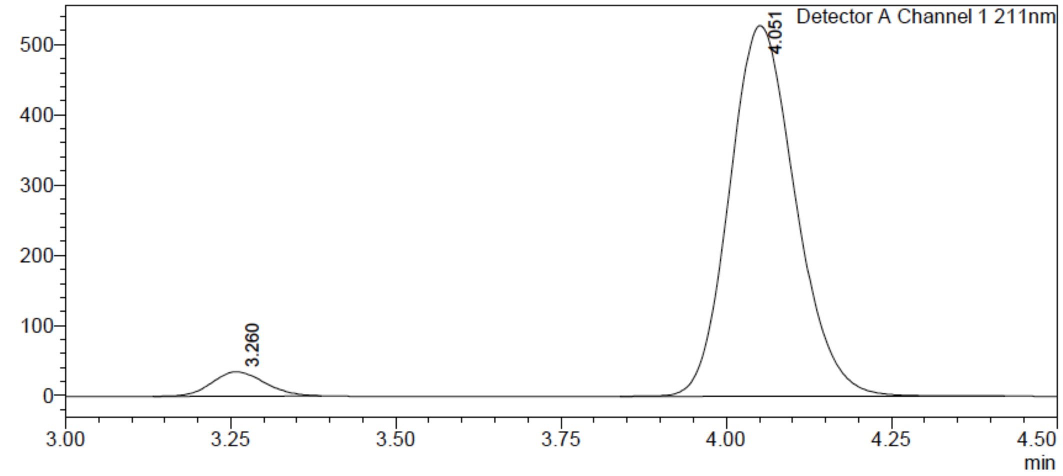

<Peak Table>

Detector A Channel 1 211nm

Peak#	Ret. Time	Area%
1	3.260	4.940
2	4.051	95.060
Total		100.000

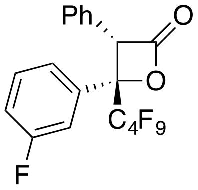

Chiralpak IC (99.9:0.1 Hexane:IPA, flow rate 1.0 mLmin^{-1}, 211 nm, 30 °C)

t_R(3*R*,4*R*): 4.4 min, t_R(3*S*,4*S*): 5.3 min, 96:4 er.

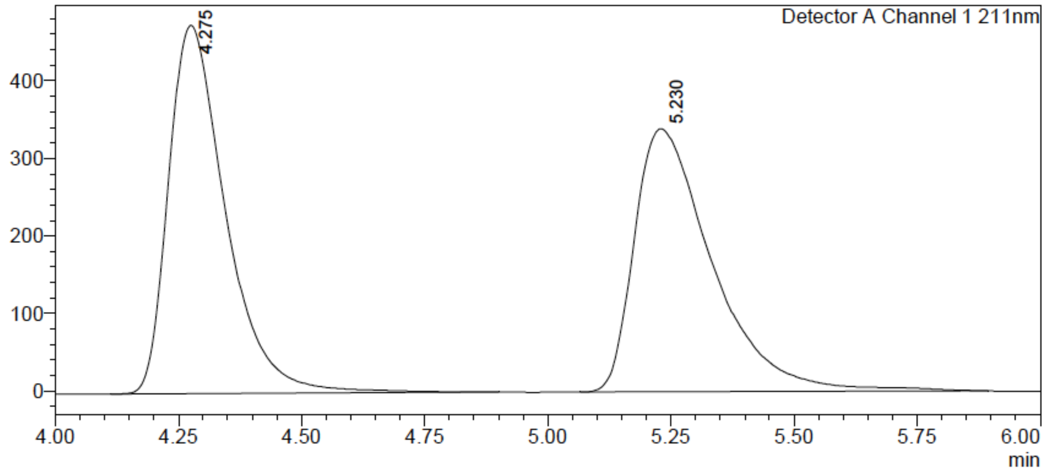

<Peak Table>

Detector A Channel 1 211nm

Peak#	Ret. Time	Area%
1	4.275	50.575
2	5.230	49.425
Total		100.000

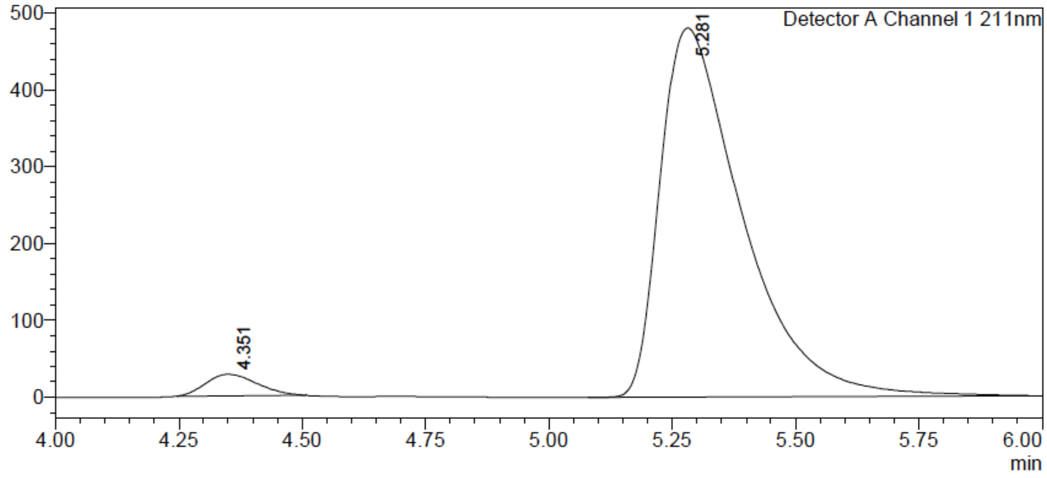

<Peak Table>

Detector A Channel 1 211nm

Peak#	Ret. Time	Area%
1	4.351	3.515
2	5.281	96.485
Total		100.000

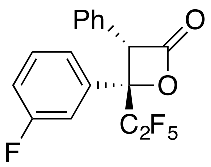

Chiralcel OD-H (99:1 Hexane:IPA, flow rate 1.5 mLmin^{-1}, 211 nm, 40 °C)
t_R(3*R*,4*R*): 3.5 min, t_R(3*S*,4*S*): 5.0 min, 95:5 er.

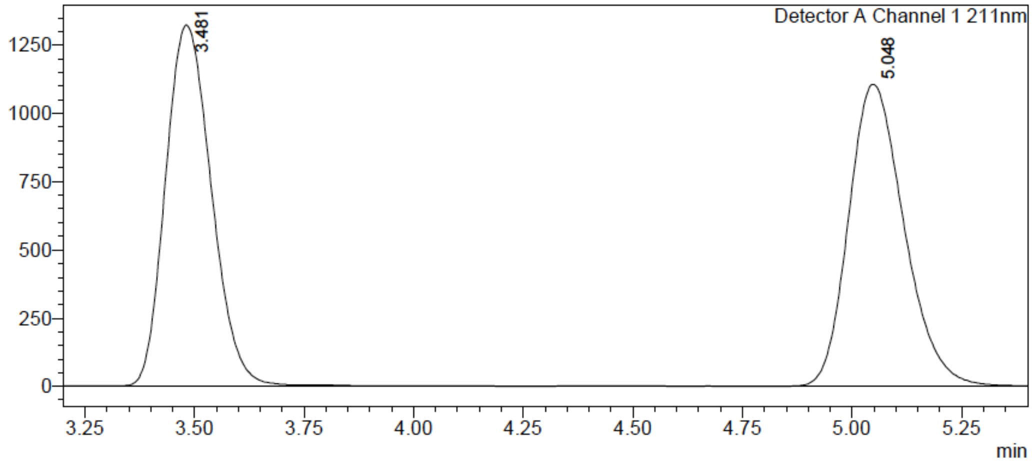

<Peak Table>

Detector A Channel 1 211nm

Peak#	Ret. Time	Area%
1	3.481	49.887
2	5.048	50.113
Total		100.000

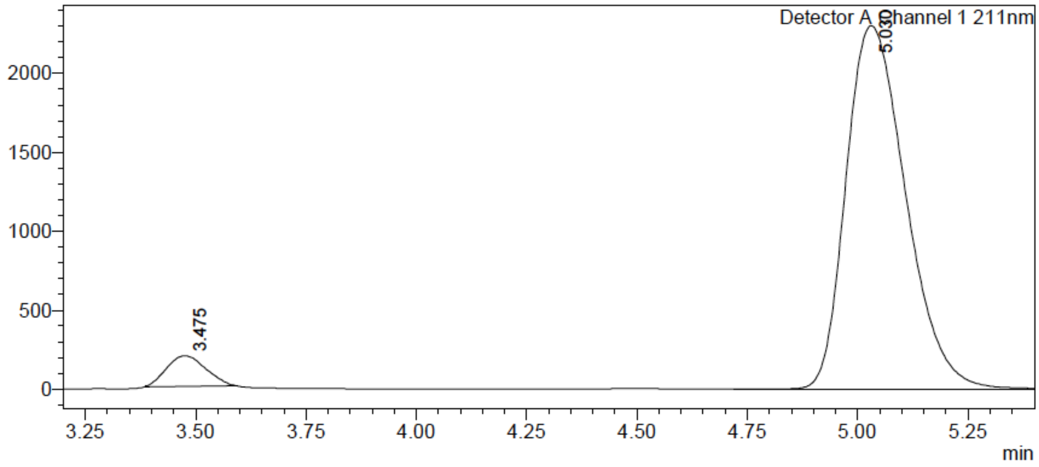

<Peak Table>

Detector A Channel 1 211nm

Peak#	Ret. Time	Area%
1	3.475	5.226
2	5.030	94.774
Total		100.000

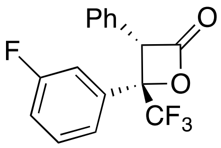

Chiralcel OD-H (97:3 Hexane:IPA, flow rate 1.5 mLmin^{-1}, 211 nm, 40 °C)

t_R(3*R*,4*R*): 3.2 min, t_R(3*S*,4*S*): 3.9 min, 97:3 er.

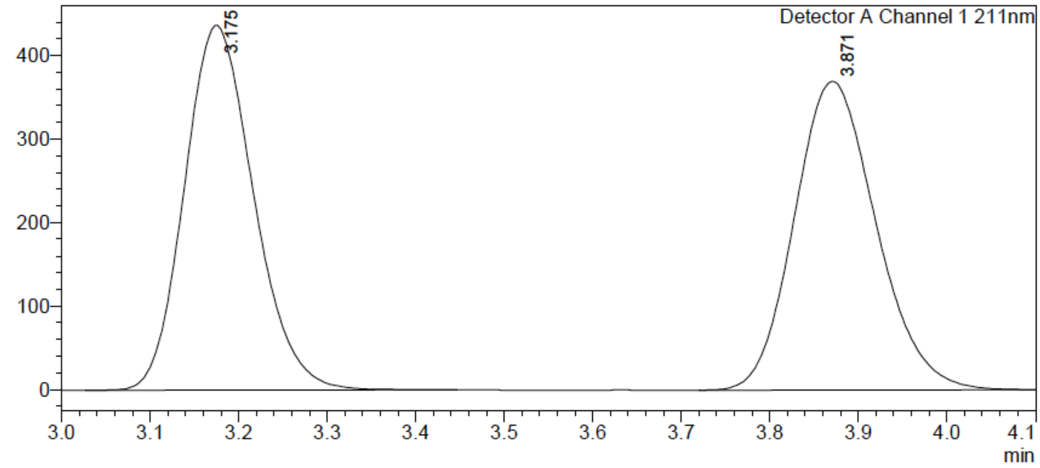

<Peak Table>

Detector A Channel 1 211nm

Peak#	Ret. Time	Area%
1	3.175	49.965
2	3.871	50.035
Total		100.000

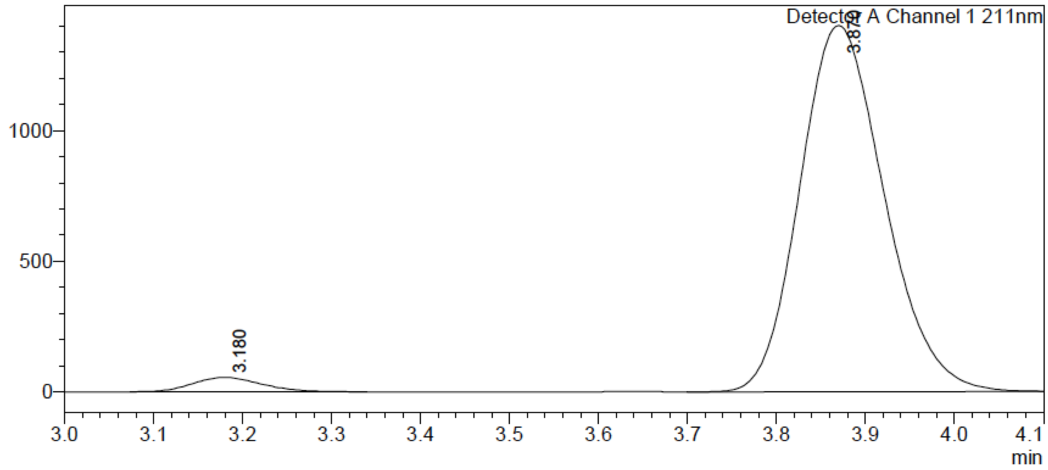

<Peak Table>

Detector A Channel 1 211nm

Peak#	Ret. Time	Area%
1	3.180	3.138
2	3.870	96.862
Total		100.000

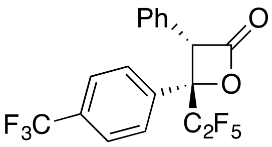

Chiralcel OD-H (97:3 Hexane:IPA, flow rate 1.5 mLmin^{-1}, 211 nm, 40 °C) t_R(3*R*,4*R*): 2.8 min, t_R(3*S*,4*S*): 3.8 min, 94:6 er.

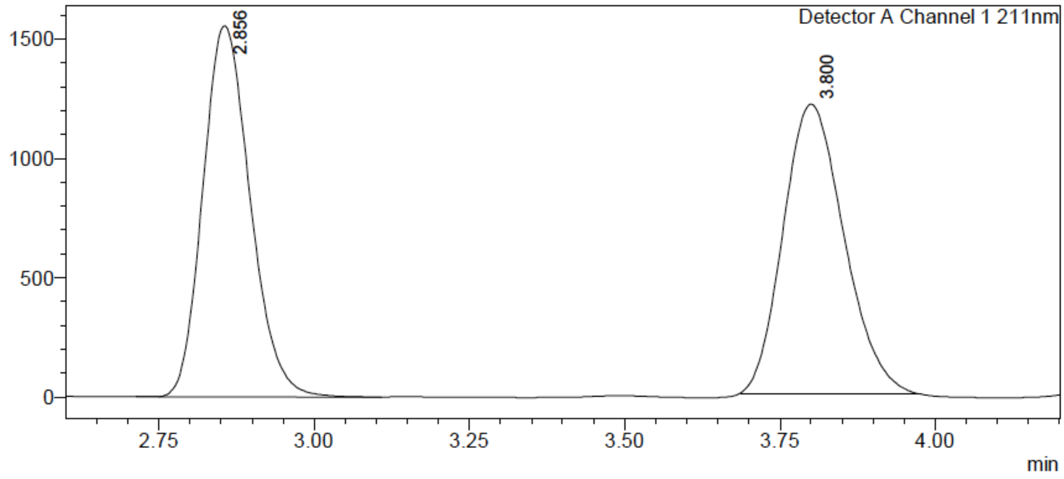

<Peak Table>

Detector A Channel 1 211nm

Peak#	Ret. Time	Area%
1	2.856	49.930
2	3.800	50.070
Total		100.000

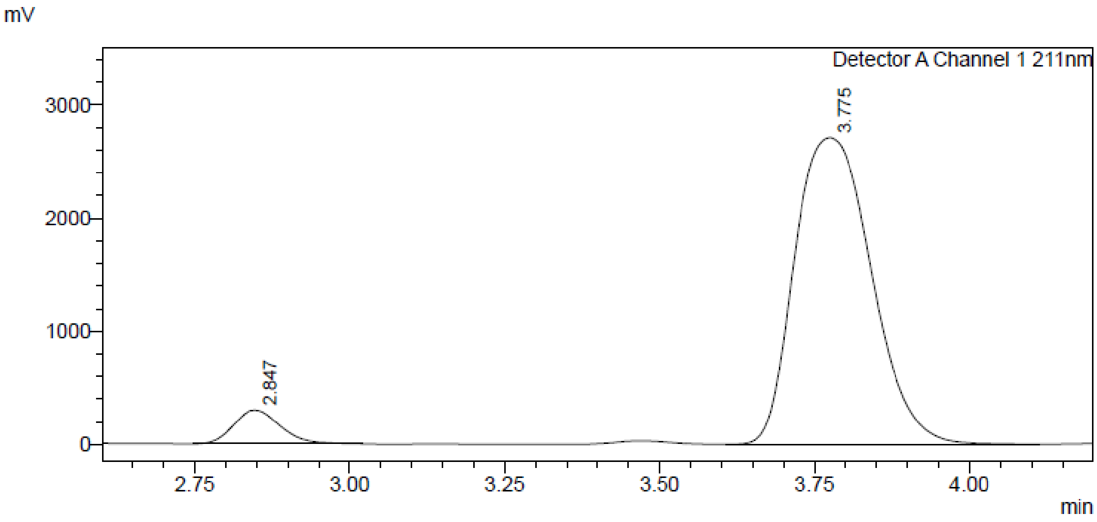

<Peak Table>

Detector A Channel 1 211nm

Peak#	Ret. Time	Area%
1	2.847	6.331
2	3.775	93.669
Total		100.000

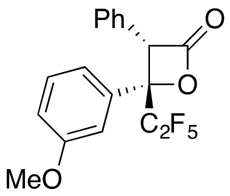

Chiralcel OD-H (97:3 Hexane:IPA, flow rate 1.5 mLmin^{-1}, 211 nm, 40 °C)

t_R(3*R*,4*R*): 3.1 min, t_R(3*S*,4*S*): 3.7 min, 88:12 er.

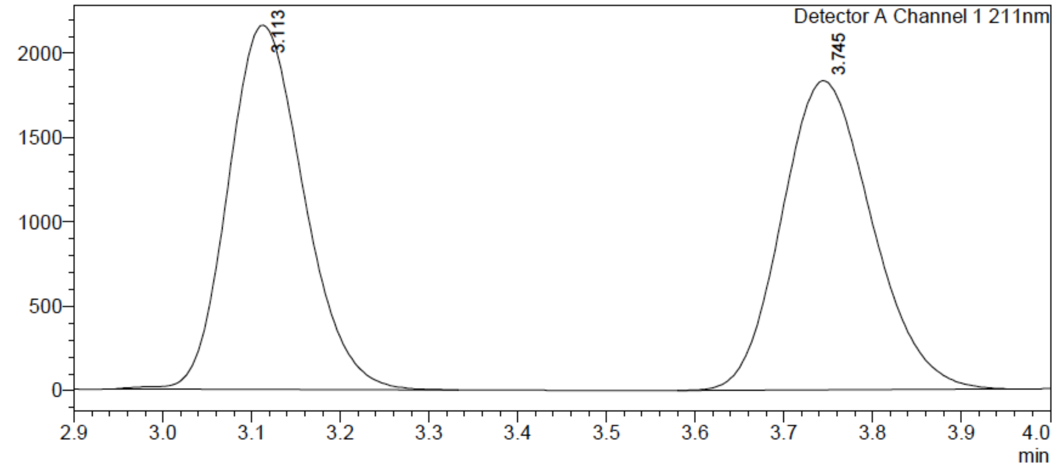

<Peak Table>

Detector A Channel 1 211nm

Peak#	Ret. Time	Area%
1	3.113	49.726
2	3.745	50.274
Total		100.000

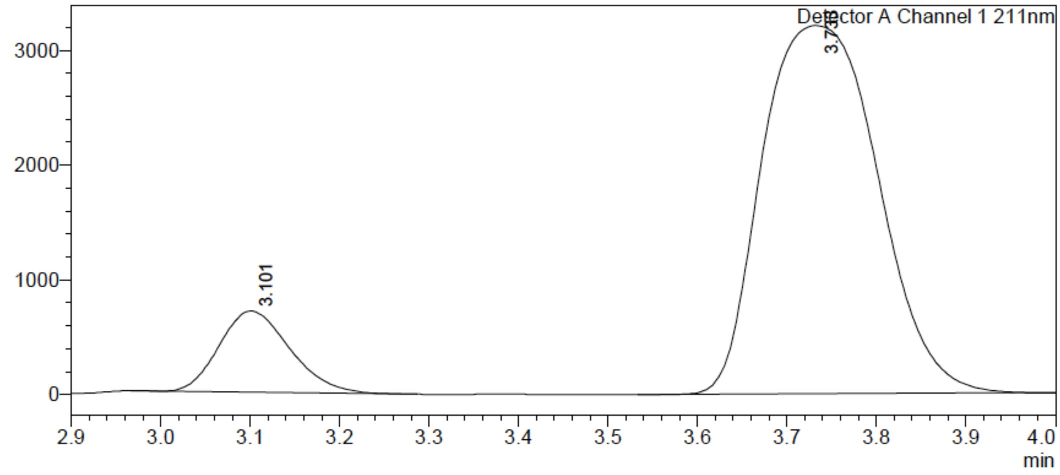

<Peak Table>

Detector A Channel 1 211nm

Peak#	Ret. Time	Area%
1	3.101	11.886
2	3.733	88.114
Total		100.000

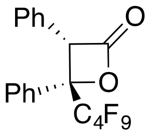

Chiralpak IC (99.9:0.1 Hexane:IPA, flow rate 1 mLmin^{-1}, 211 nm, 30 °C) t_R(3*R*,4*R*): 3.3 min, t_R(3*S*,4*S*): 3.9 min, 98:2 er.

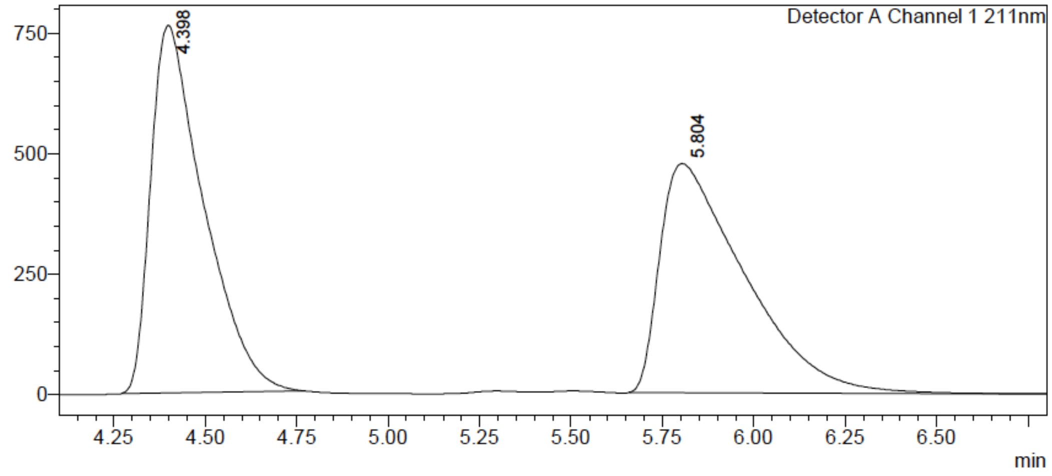

<Peak Table>

Detector A Channel 1 211nm

Peak#	Ret. Time	Area%
1	4.398	50.103
2	5.804	49.897
Total		100.000

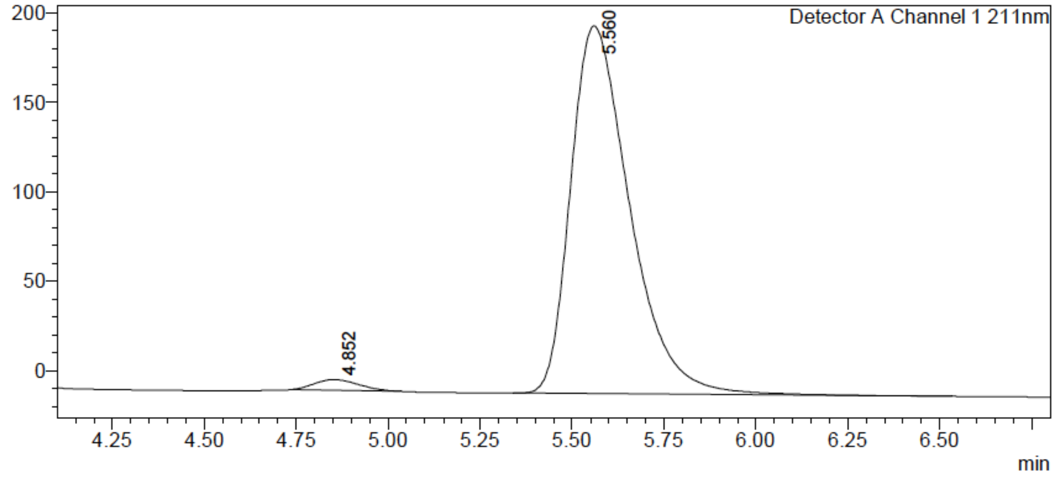

<Peak Table>

Detector A Channel 1 211nm

Peak#	Ret. Time	Area%
1	4.852	2.114
2	5.560	97.886
Total		100.000

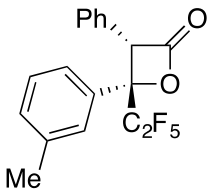

Chiral HPLC analysis, Chiralcel OD-H (99.5:0.5 Hexane : IPA, flow rate 1.5 mLmin^{-1}, 211 nm, 40ºC) t_R(3*R*,4*R*): 3.5 min, t_R(3*S*,4*S*): 4.9 min, 92:8 er.

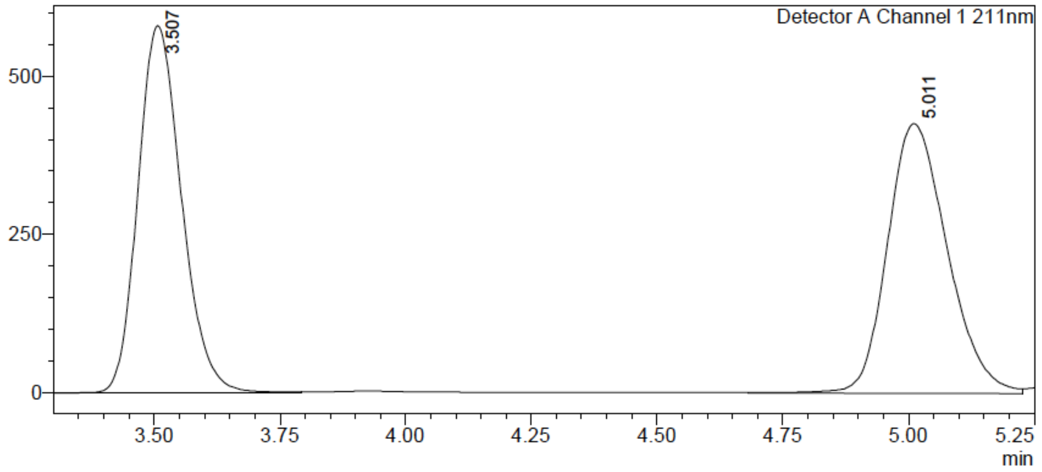

<Peak Table>

Detector A Channel 1 211nm

Peak#	Ret. Time	Area%
1	3.507	49.874
2	5.011	50.126
Total		100.000

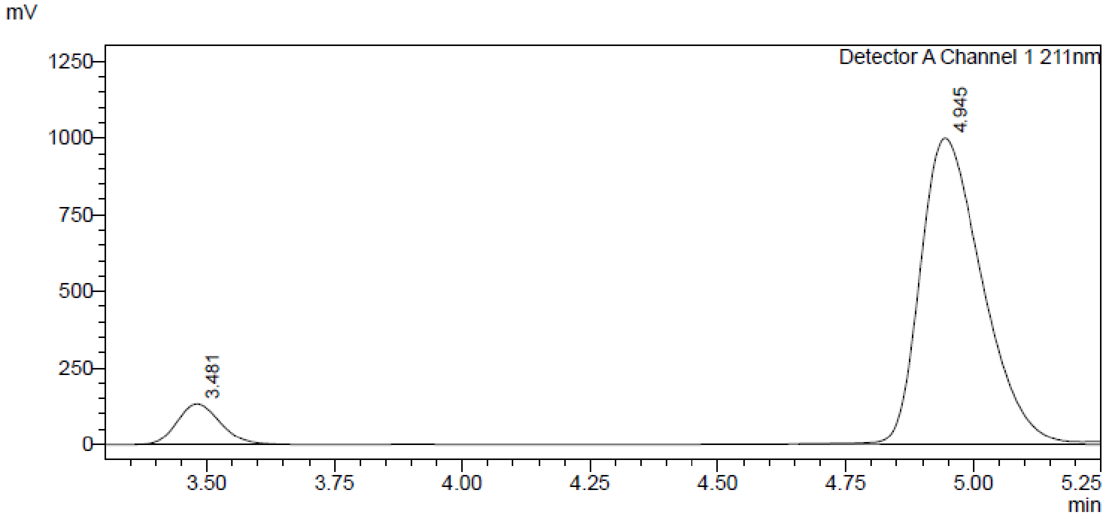

<Peak Table>

Detector A Channel 1 211nm

Peak#	Ret. Time	Area%
1	3.481	8.392
2	4.945	91.608
Total		100.000

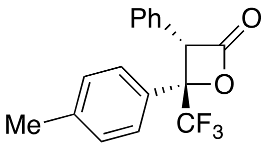

Chiralcel OD-H (97:3 Hexane:IPA, flow rate 1.5 mLmin^{-1}, 211 nm, 40 °C) t_R(3*R*,4*R*): 3.0 min, t_R(3*S*,4*S*): 3.4 min, 99:1 er.

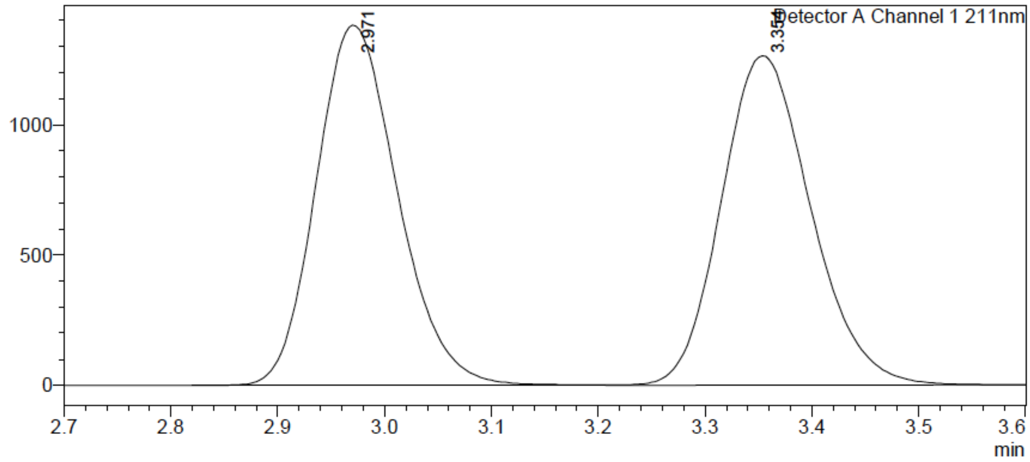

<Peak Table>

Detector A Channel 1 211nm

Peak#	Ret. Time	Area%
1	2.971	49.825
2	3.354	50.175
Total		100.000

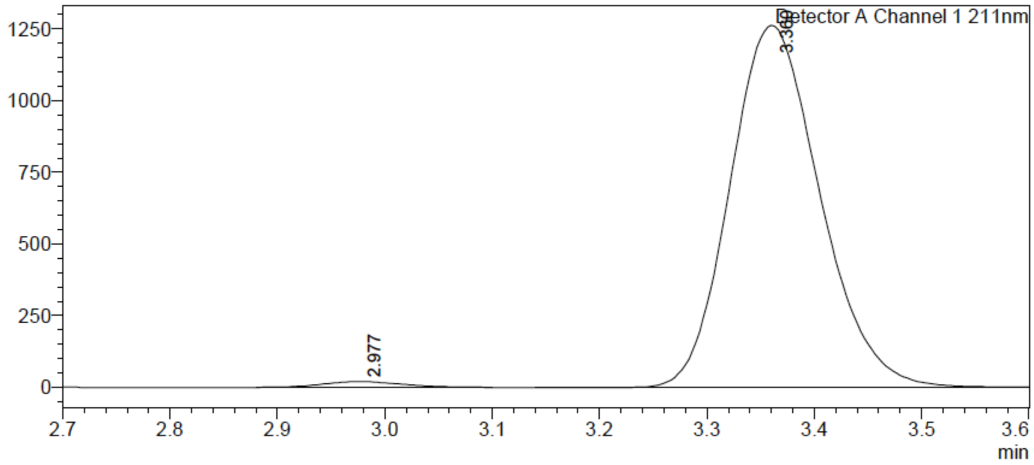

<Peak Table>

Detector A Channel 1 211nm

Peak#	Ret. Time	Area%
1	2.977	1.351
2	3.360	98.649
Total		100.000

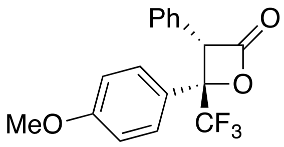

Chiralcel OD-H (97:3 Hexane:IPA, flow rate 1.5 mLmin^{-1}, 211 nm, 40 °C) t_R(3*R*,4*R*): 3.6 min, t_R(3*S*,4*S*): 4.5 min, 99:1 er .

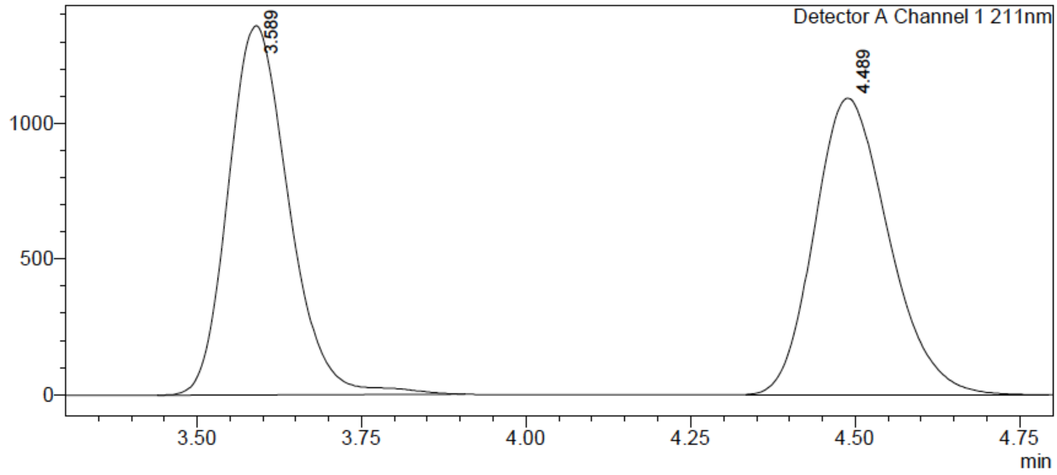

<Peak Table>

Detector A Channel 1 211nm

Peak#	Ret. Time	Area%
1	3.589	50.347
2	4.489	49.653
Total		100.000

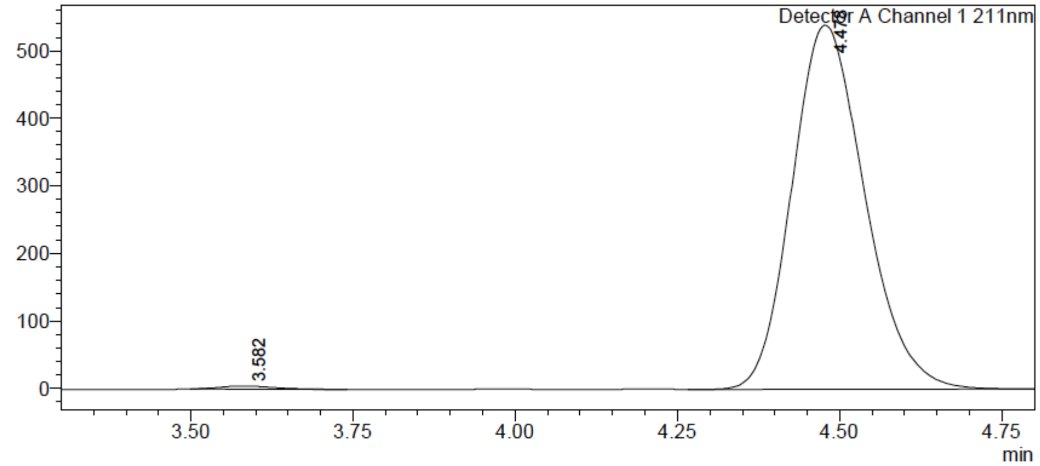

<Peak Table>

Detector A Channel 1 211nm

Peak#	Ret. Time	Area%
1	3.582	0.630
2	4.478	99.370
Total		100.000

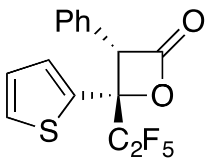

Chiralcel OD-H (99.9:0.1 Hexane:IPA, flow rate 1.5 mLmin^{-1}, 211 nm, 40 °C) t_R(3*R*,4*S*): 5.5 min, t_R(3*S*,4*R*): 11.9 min, 87:13 er.

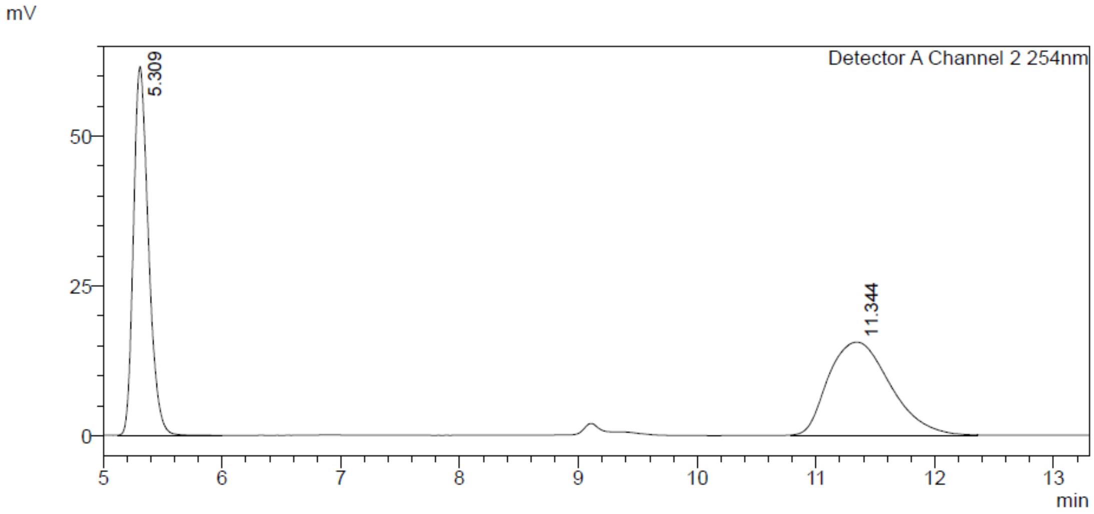

<Peak Table>

Detector A Channel 2 254nm

Peak#	Ret. Time	Area%
1	5.309	49.057
2	11.344	50.943
Total		100.000

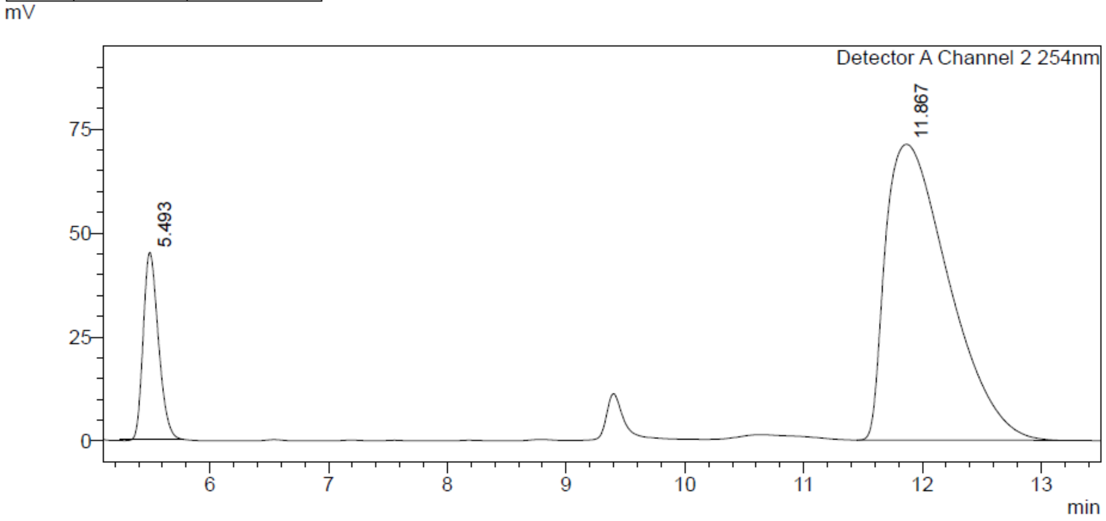

<Peak Table>

Detector A Channel 2 254nm

Peak#	Ret. Time	Area%
1	5.493	13.055
2	11.867	86.945
Total		100.000

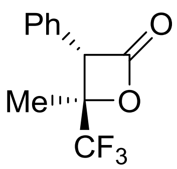

Chiralcel OD-H (97:3 Hexane:IPA, flow rate 1.5 mLmin^{-1}, 254 nm, 40 °C)

t_R(3*R*,4*S*): 2.9 min, t_R(3*S*,4*R*): 3.3 min, 93:7 er.

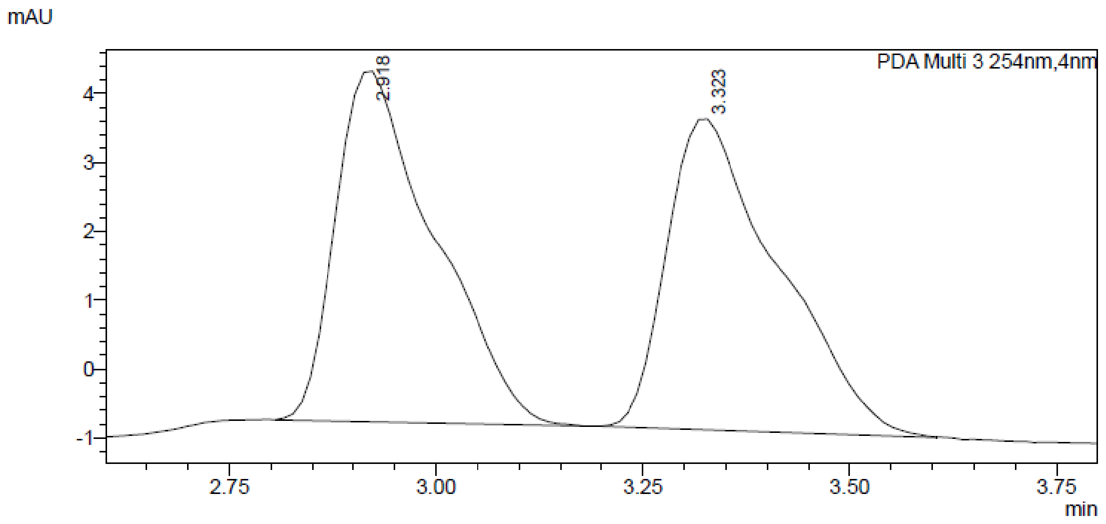

<Peak Table>

PDA Ch3 254nm

Peak#	Ret. Time	Area%
1	2.918	50.044
2	3.323	49.956
Total		100.000

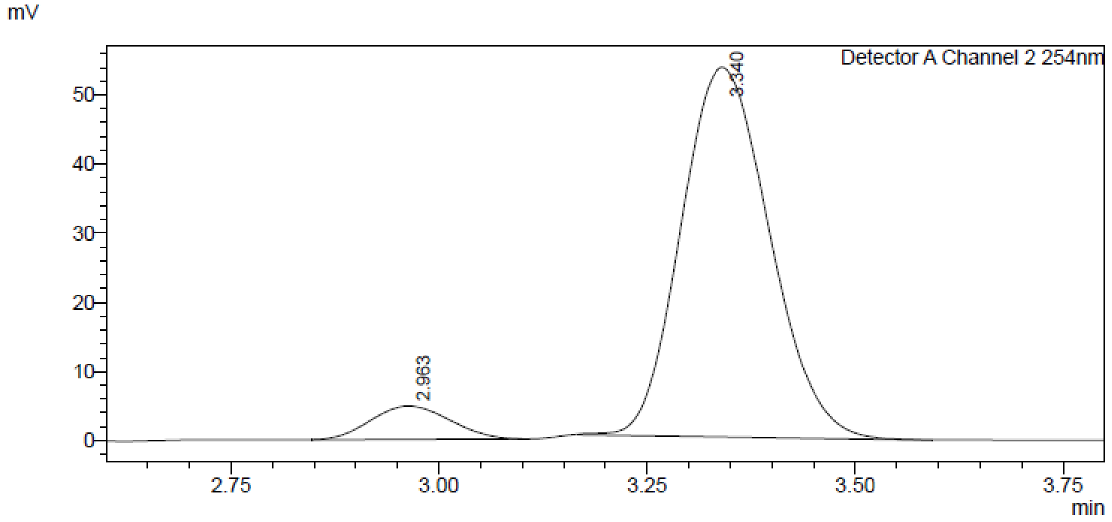

<Peak Table>

Detector A Channel 2 254nm

Peak#	Ret. Time	Area%
1	2.963	7.483
2	3.340	92.517
Total		100.000

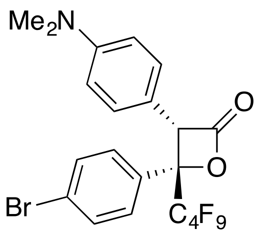

Chiralcel OD-H (97:3 Hexane:IPA, flow rate 1.5 mLmin^{-1}, 211 nm, 40 °C) t_R(3*R*,4*R*): 3.8 min, t_R(3*S*,4*S*): 4.6 min, 97:3 er.

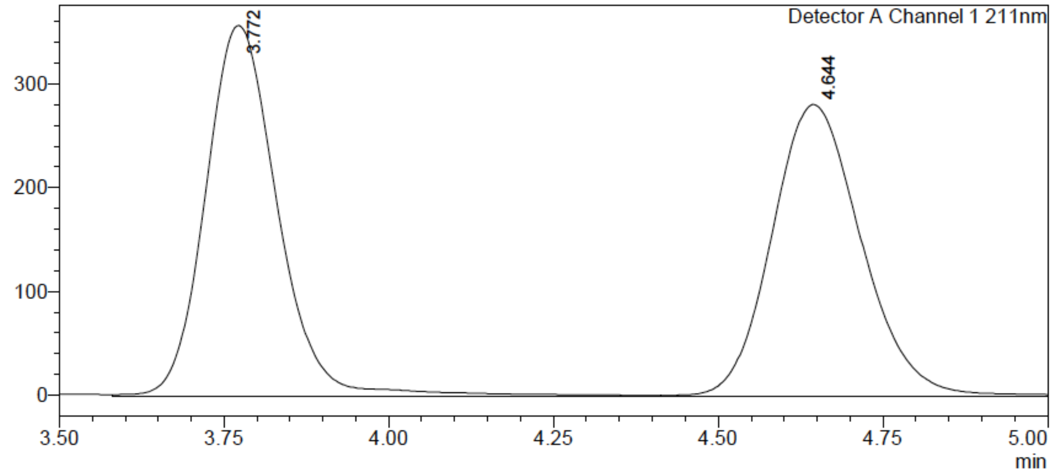

<Peak Table>

Detector A Channel 1 211nm

Peak#	Ret. Time	Area%
1	3.772	50.674
2	4.644	49.326
Total		100.000

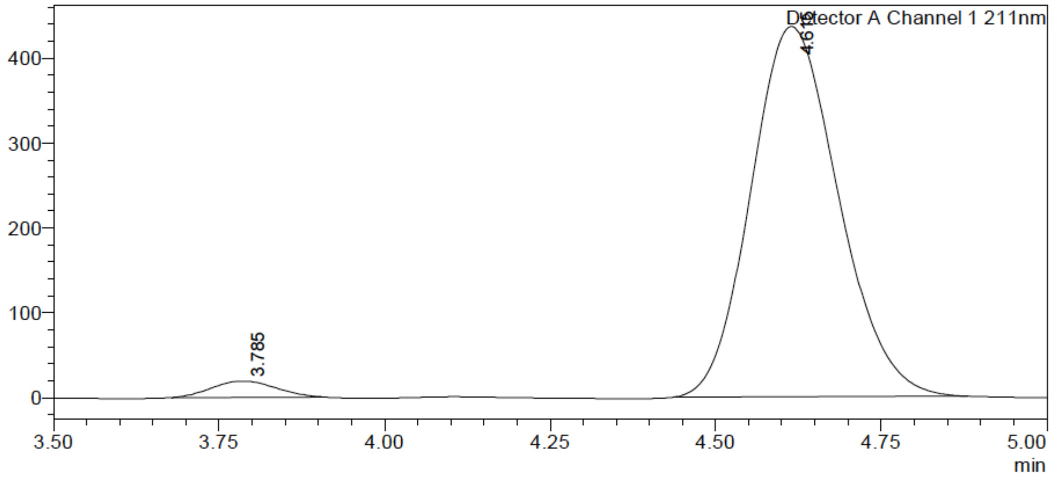

<Peak Table>

Detector A Channel 1 211nm

Peak#	Ret. Time	Area%
1	3.785	3.078
2	4.615	96.922
Total		100.000

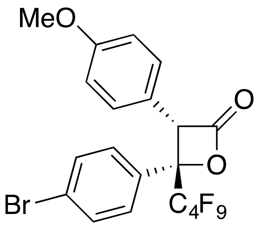

Chiralcel OD-H (97:3 hexane:IPA, flow rate 1.5 mLmin^{-1}, 211 nm, 40 °C), t_R(3*R*,4*R*): 3.3 min, t_R(3*S*,4*S*): 3.8 min, 97:3 er.

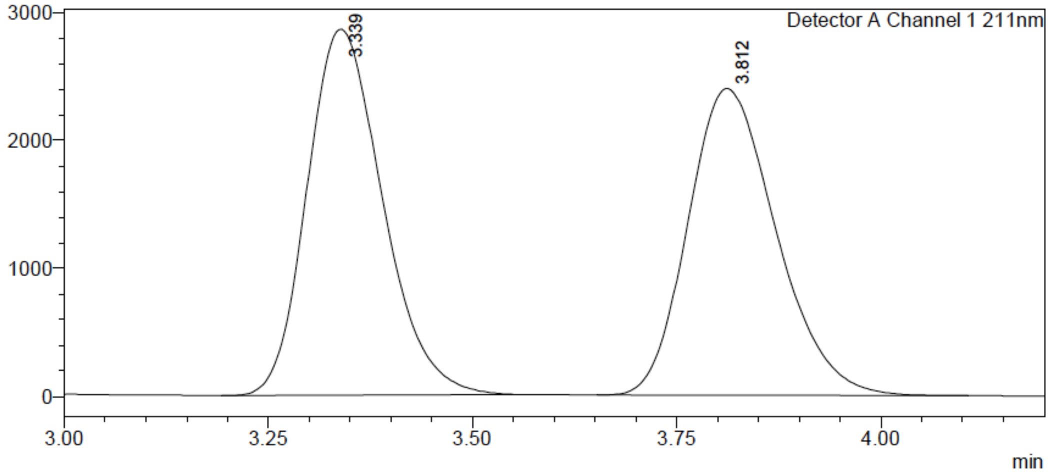

<Peak Table>

Detector A Channel 1 211nm

Peak#	Ret. Time	Area%
1	3.339	50.577
2	3.812	49.423
Total		100.000

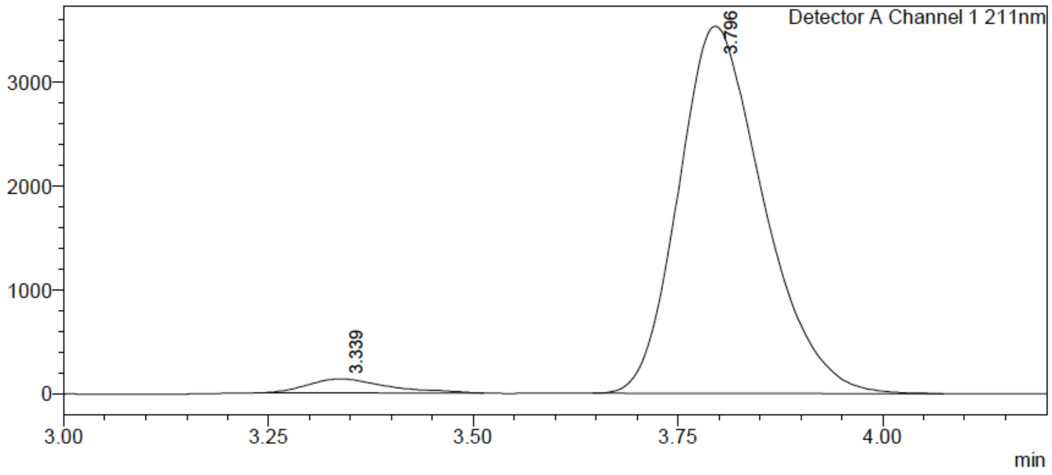

<Peak Table>

Detector A Channel 1 211nm

Peak#	Ret. Time	Area%
1	3.339	3.427
2	3.796	96.573
Total		100.000

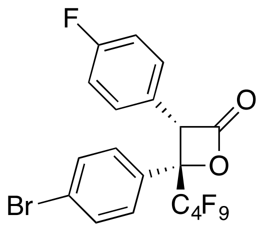

Chiralcel OD-H (97:3 hexane:IPA, flow rate 1.5 mLmin^{-1}, 211 nm, 40 °C), t_R(3*R*,4*R*): 2.8 min, t_R(3*S*,4*S*): 3.1 min, 96:4 er.

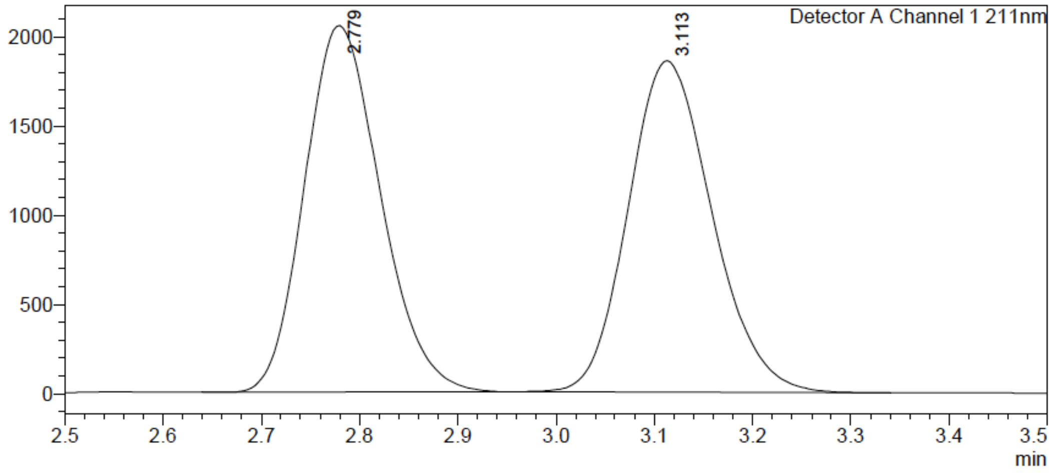

<Peak Table>

Detector A Channel 1 211nm

Peak#	Ret. Time	Area%
1	2.779	49.972
2	3.113	50.028
Total		100.000

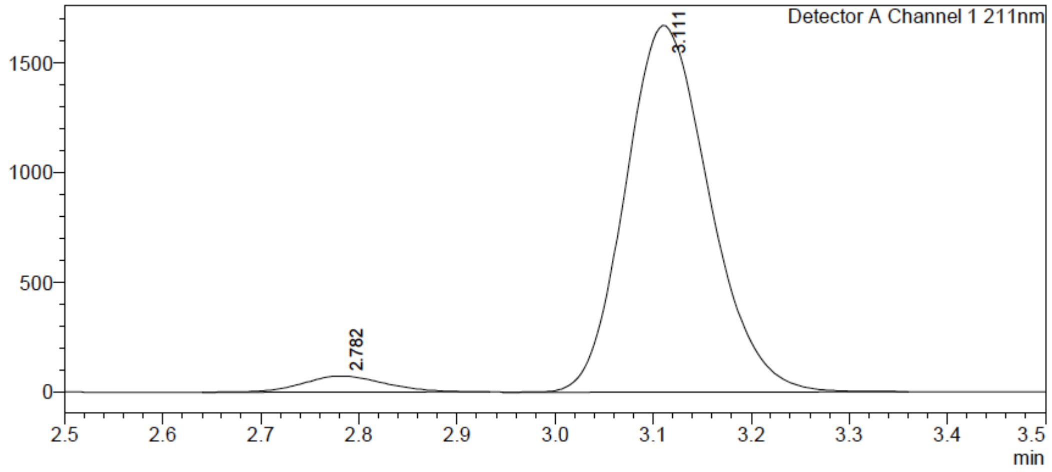

<Peak Table>

Detector A Channel 1 211nm

Peak#	Ret. Time	Area%
1	2.782	4.124
2	3.111	95.876
Total		100.000

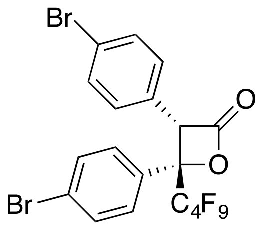

Chiralpak AD-H (99:1 hexane:IPA, flow rate 1.5 mLmin^{-1}, 211 nm, 40 °C) t_R(3*S*,4*S*): 3.6 min, t_R(3*R*,4*R*): 4.1 min, 95:5 er.

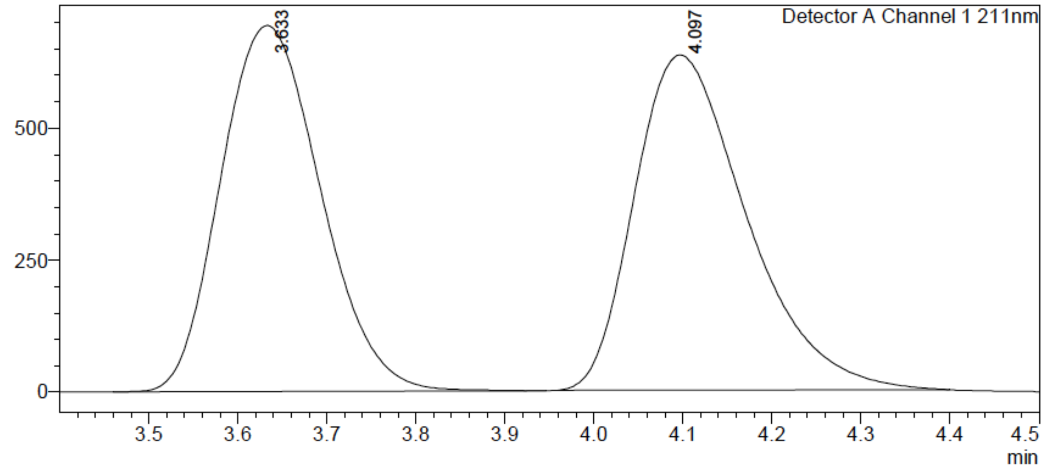

<Peak Table>

Detector A Channel 1 211nm

Peak#	Ret. Time	Area%
1	3.633	49.312
2	4.097	50.688
Total		100.000

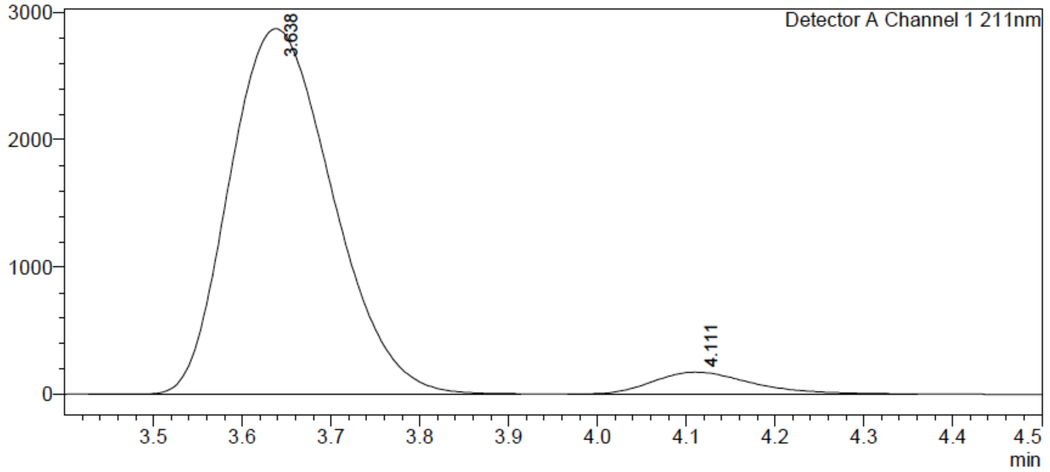

<Peak Table>

Detector A Channel 1 211nm

Peak#	Ret. Time	Area%
1	3.638	94.558
2	4.111	5.442
Total		100.000

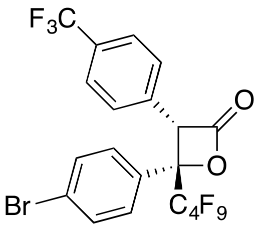

Chiralpak AD-H (99:1 hexane:IPA, flow rate 1.5 mLmin^{-1}, 211 nm, 40 °C), t_R(3*S*,4*S*): 3.2 min, t_R(3*R*,4*R*): 4.0 min, 95:5 er .

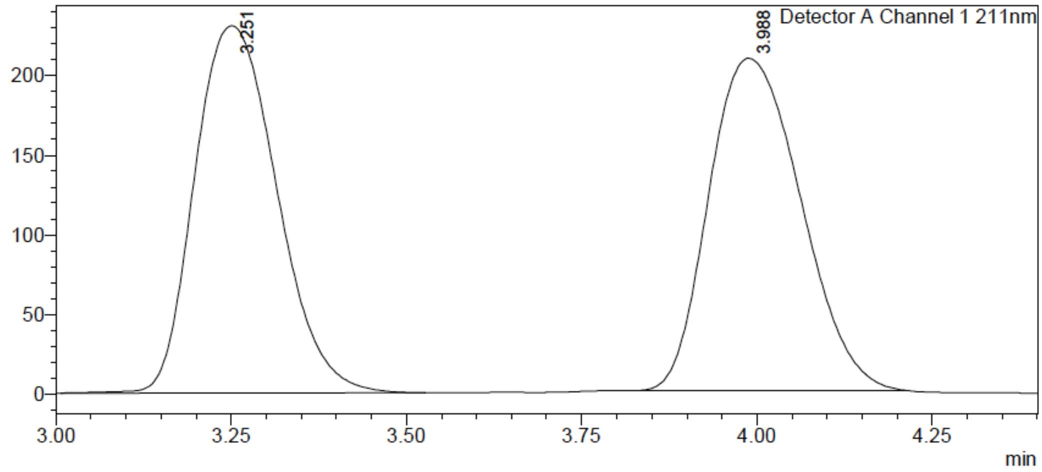

<Peak Table>

Detector A Channel 1 211nm

Peak#	Ret. Time	Area%
1	3.251	49.757
2	3.988	50.243
Total		100.000

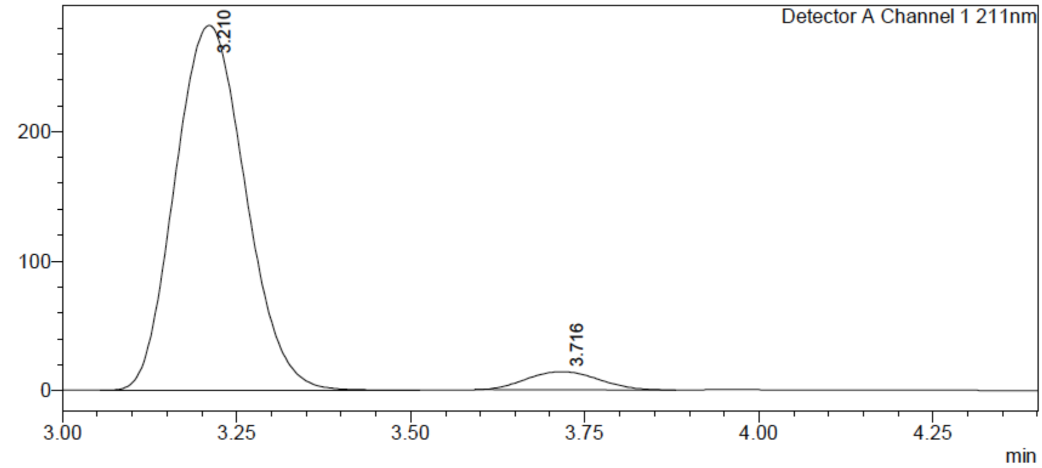

<Peak Table>

Detector A Channel 1 211nm

Peak#	Ret. Time	Area%
1	3.210	95.113
2	3.716	4.887
Total		100.000

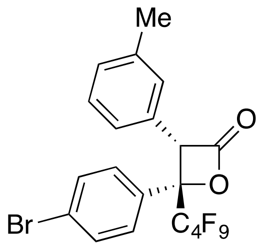

Chiralcel OD-H (97:3 hexane:IPA, flow rate 1.5 mLmin^{-1}, 211 nm, 40 °C), t_R(3*R*,4*R*): 2.6 min, t_R(3*S*,4*S*): 3.3 min, 97:3 er.

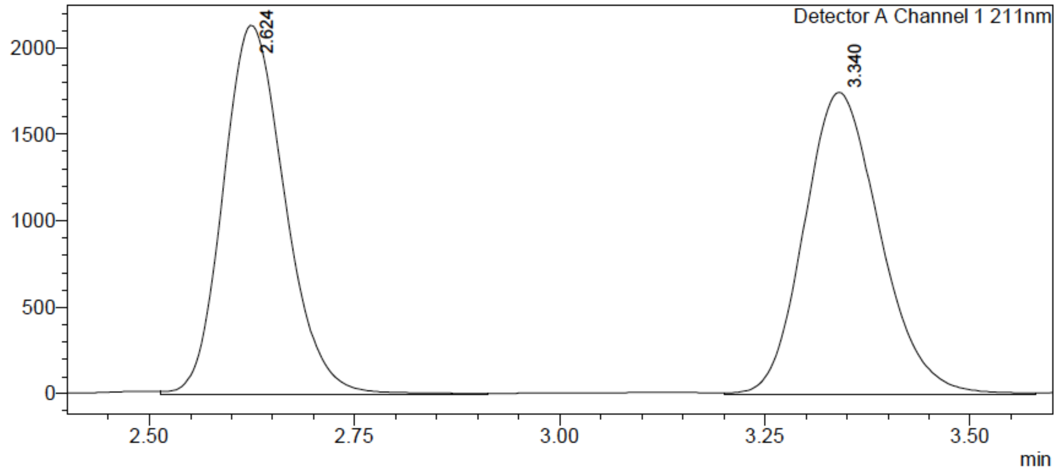

<Peak Table>

Detector A Channel 1 211nm

Peak#	Ret. Time	Area%
1	2.624	49.876
2	3.340	50.124
Total		100.000

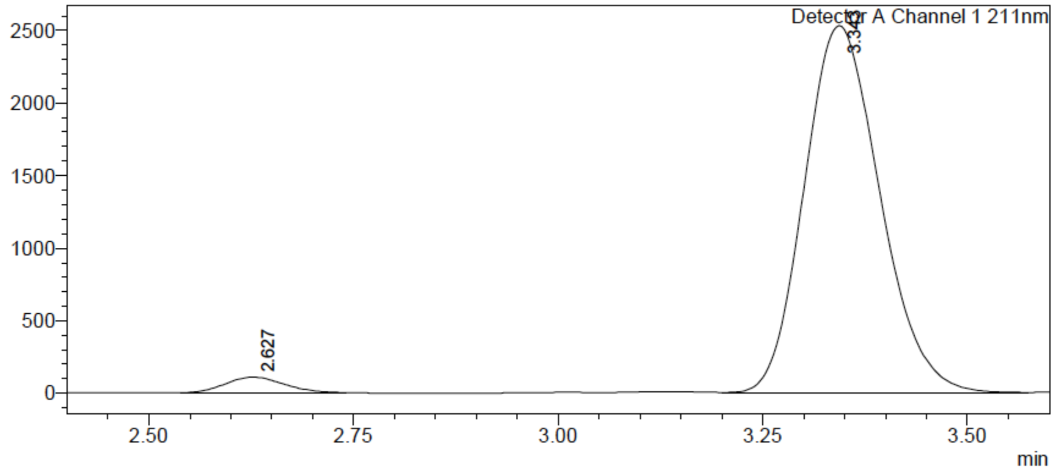

<Peak Table>

Detector A Channel 1 211nm

Peak#	Ret. Time	Area%
1	2.627	3.144
2	3.343	96.856
Total		100.000

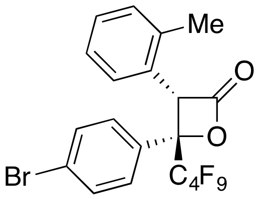

Chiralcel OD-H (97:3 hexane:IPA, flow rate 1.5 mLmin^{-1}, 211 nm, 40 °C), t_R(3*R*,4*R*): 2.8 min, t_R(3*S*,4*S*): 3.8 min, 99:1 er.

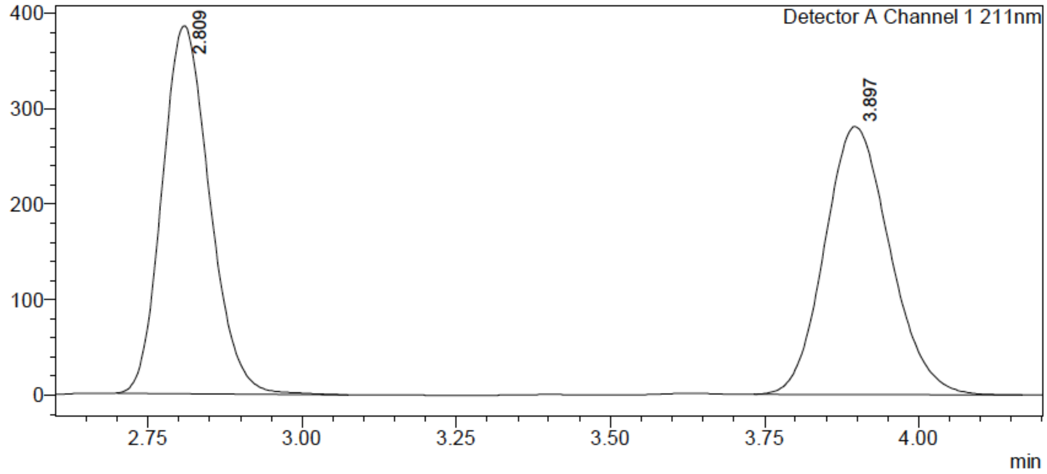

<Peak Table>

Detector A Channel 1 211nm

Peak#	Ret. Time	Area%
1	2.809	49.993
2	3.897	50.007
Total		100.000

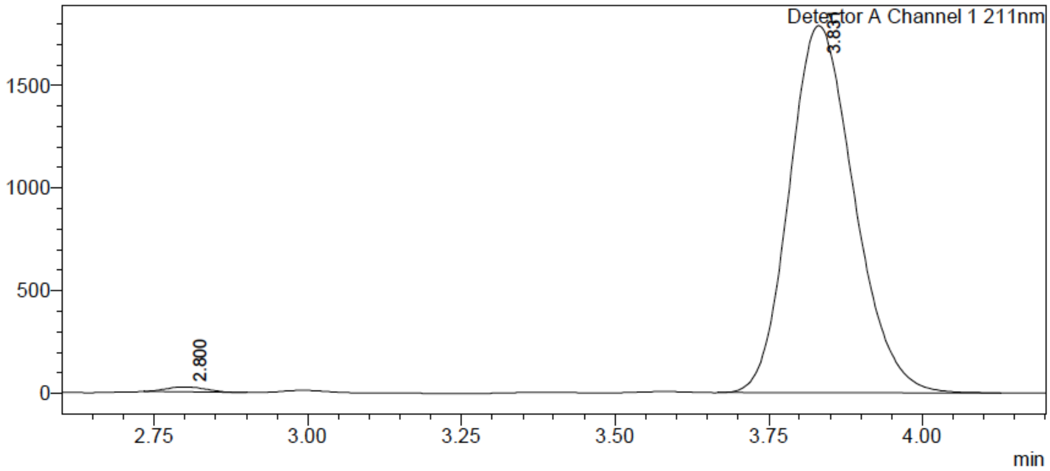

<Peak Table>

Detector A Channel 1 211nm

Peak#	Ret. Time	Area%
1	2.800	0.846
2	3.831	99.154
Total		100.000

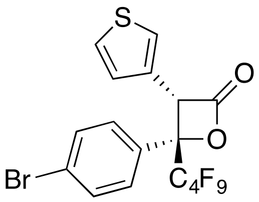

Chiralcel OD-H (97:3 hexane:IPA, flow rate 1.5 mLmin^{-1}, 211 nm, 40 °C), t_R(3*R*,4*R*): 3.1 min, t_R(3*S*,4*S*): 3.8 min, 94:6 er.

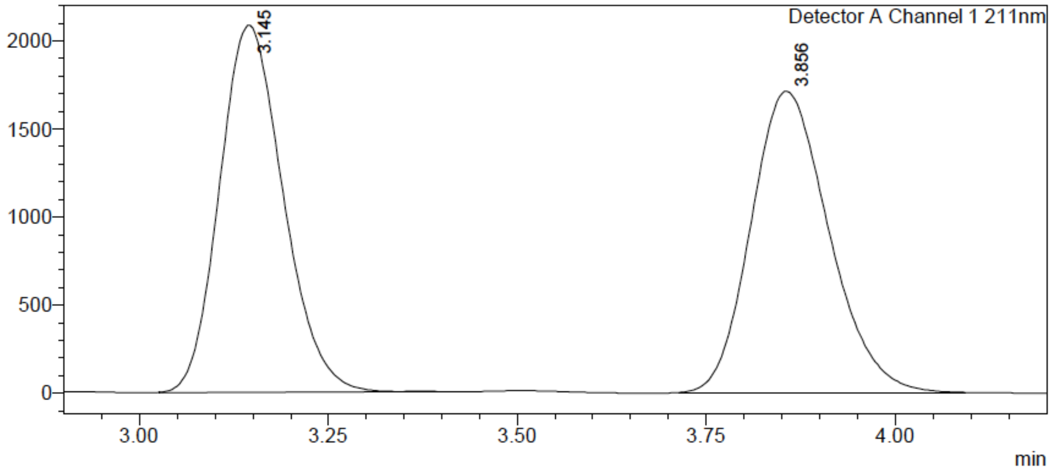

<Peak Table>

Detector A Channel 1 211nm

Peak#	Ret. Time	Area%
1	3.145	49.893
2	3.856	50.107
Total		100.000

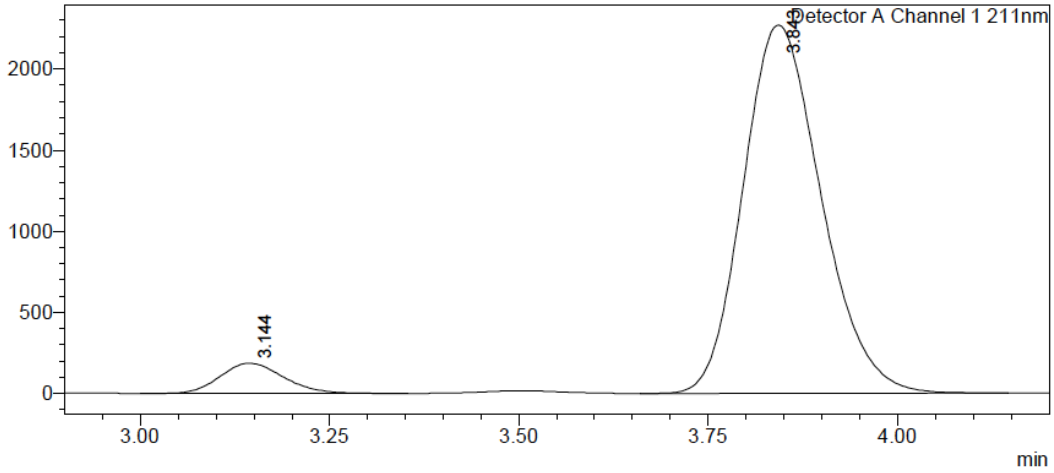

<Peak Table>

Detector A Channel 1 211nm

Peak#	Ret. Time	Area%
1	3.144	6.253
2	3.843	93.747
Total		100.000

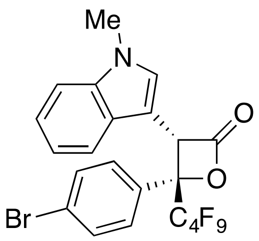

Chiralpak AS-H (99:1 hexane:IPA, flow rate 1.5 mLmin^{-1}, 211 nm, 40 °C), t_R(3*R*,4*R*): 3.9 min, t_R(3*S*,4*S*): 5.2 min, 99:1 er.

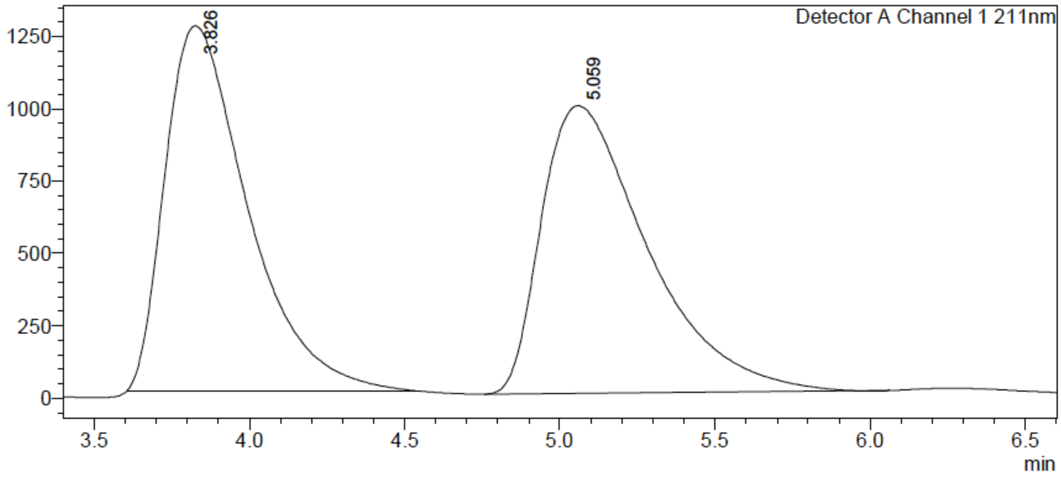

<Peak Table>

Detector A Channel 1 211nm

Peak#	Ret. Time	Area%
1	3.826	49.744
2	5.059	50.256
Total		100.000

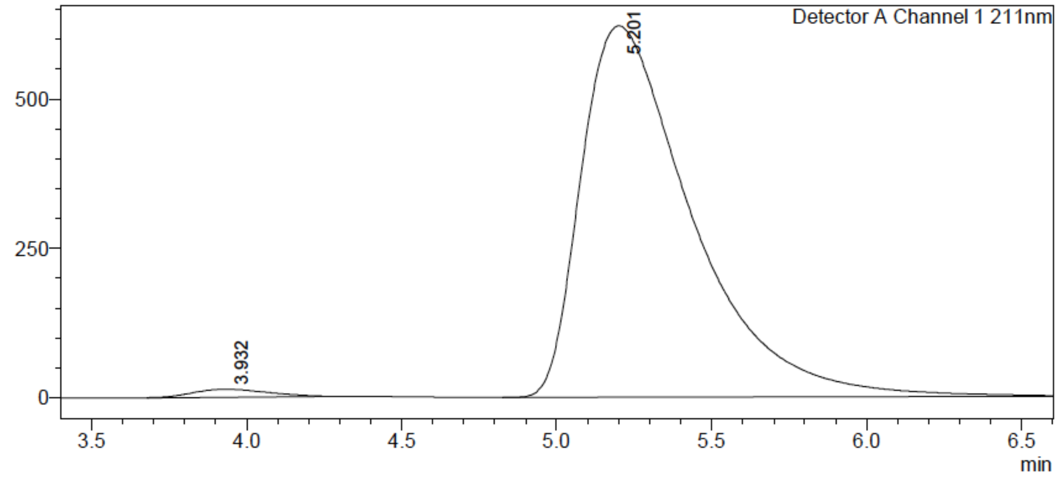

<Peak Table>

Detector A Channel 1 211nm

Peak#	Ret. Time	Area%
1	3.932	1.450
2	5.201	98.550
Total		100.000

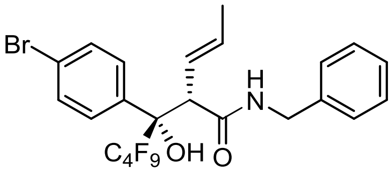

Chiralpak AD-H (98:2 hexane:IPA, flow rate 1.5 mLmin^{-1}, 254 nm, 40 °C), t_R(3*R*,4*R*): 9.9 min, t_R(3*S*,4*S*): 13.2 min, 86:14 er.

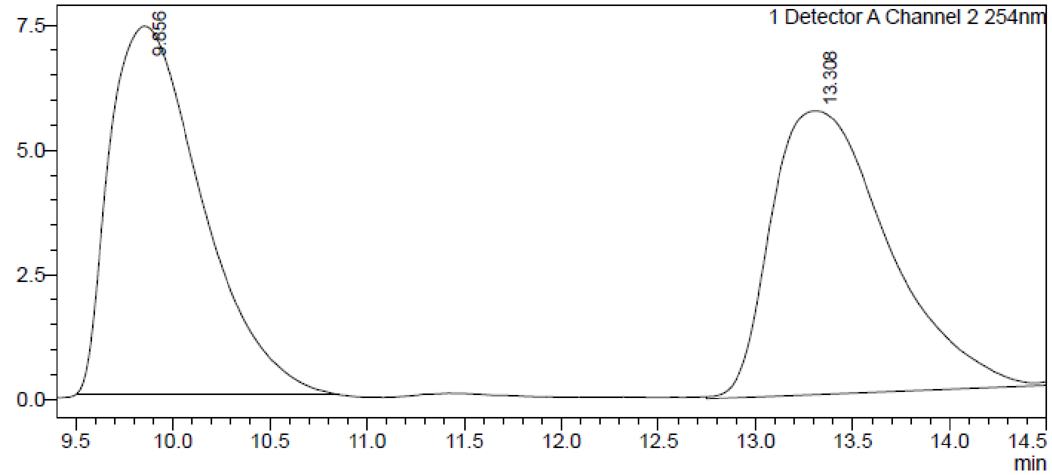

<Peak Table>

Detector A Channel 2 254nm

Peak#	Ret. Time	Area%
1	9.856	49.961
2	13.308	50.039
Total		100.000

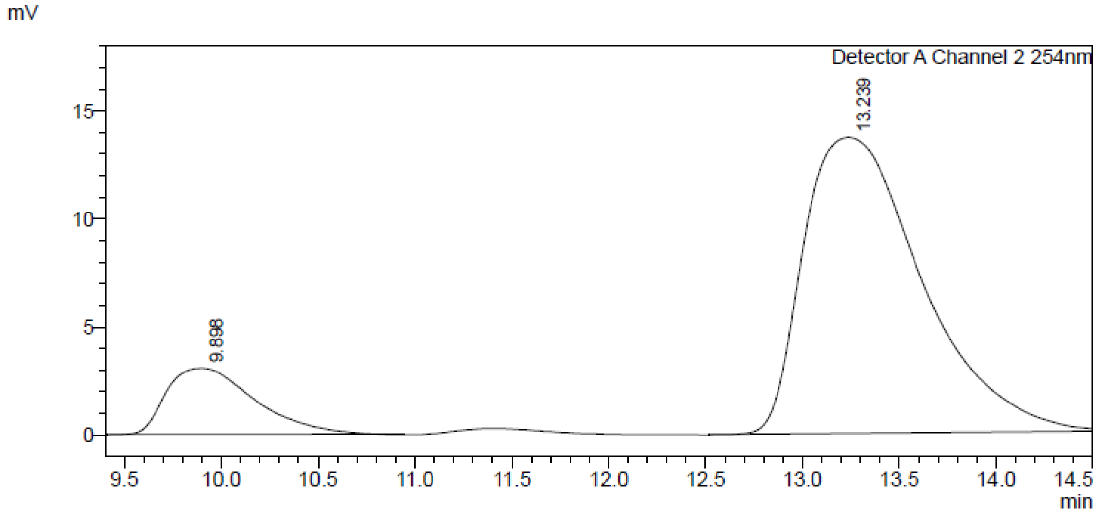

<Peak Table>

Detector A Channel 2 254nm

Peak#	Ret. Time	Area%
1	9.898	14.204
2	13.239	85.796
Total		100.000

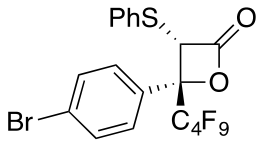

Chiralcel OD-H (99.5:0.5 Hexane:IPA, flow rate 1.5 mLmin^{-1}, 211 nm, 40 °C) t_R(3*R*,4*R*): 3.9 min, t_R(3*S*,4*S*): 4.4 min, 83:17 er.

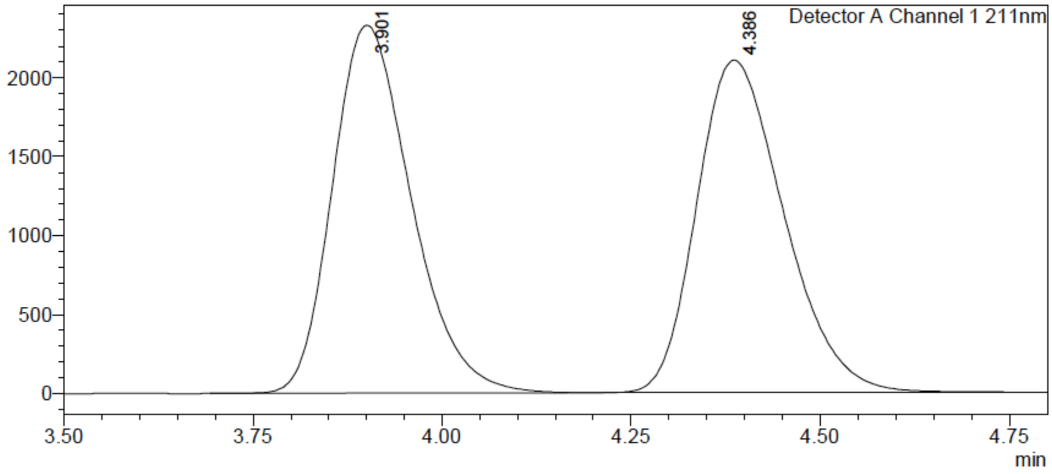

<Peak Table>

Detector A Channel 1 211nm

Peak#	Ret. Time	Area%
1	3.901	49.910
2	4.386	50.090
Total		100.000

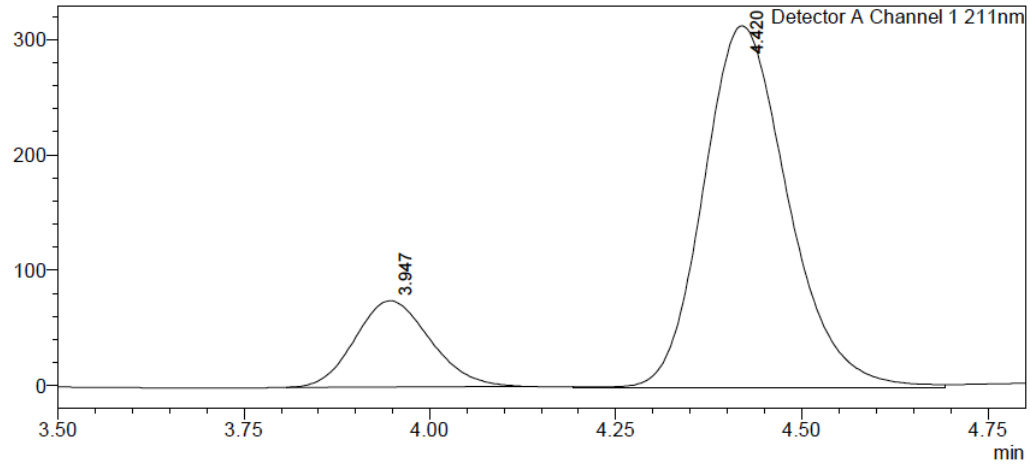

<Peak Table>

Detector A Channel 1 211nm

Peak#	Ret. Time	Area%
1	3.947	17.494
2	4.420	82.506
Total		100.000

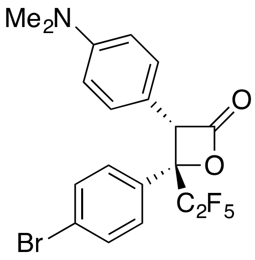

Chiralcel OD-H (97:3 Hexane:IPA, flow rate 1.5 mLmin^{-1}, 211 nm, 40 °C) t_R(3*R*,4*R*): 4.0 min, t_R(3*S*,4*S*): 5.3 min, 96:4 er.

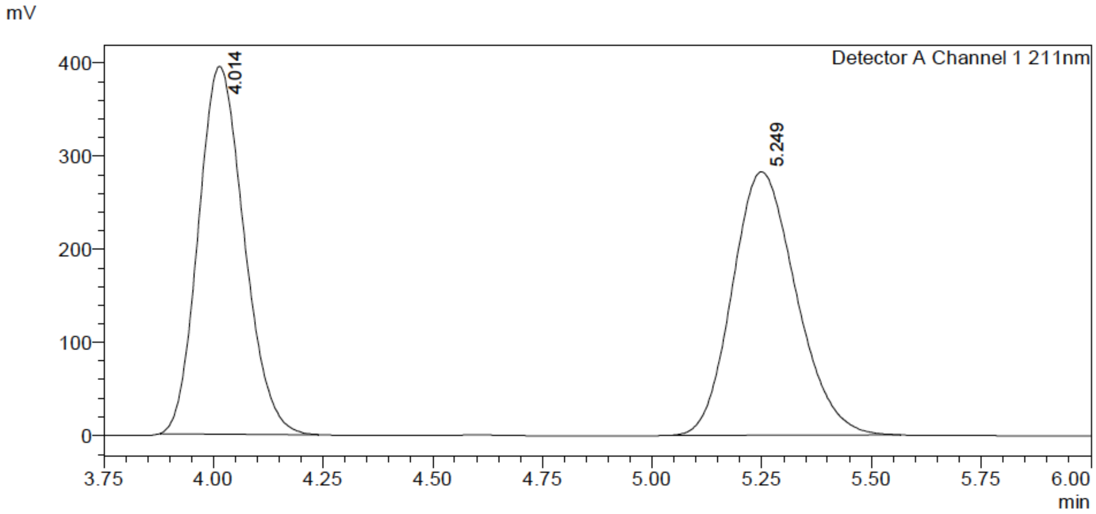

<Peak Table>

Detector A Channel 1 211nm

Peak#	Ret. Time	Area%
1	4.014	49.935
2	5.249	50.065
Total		100.000

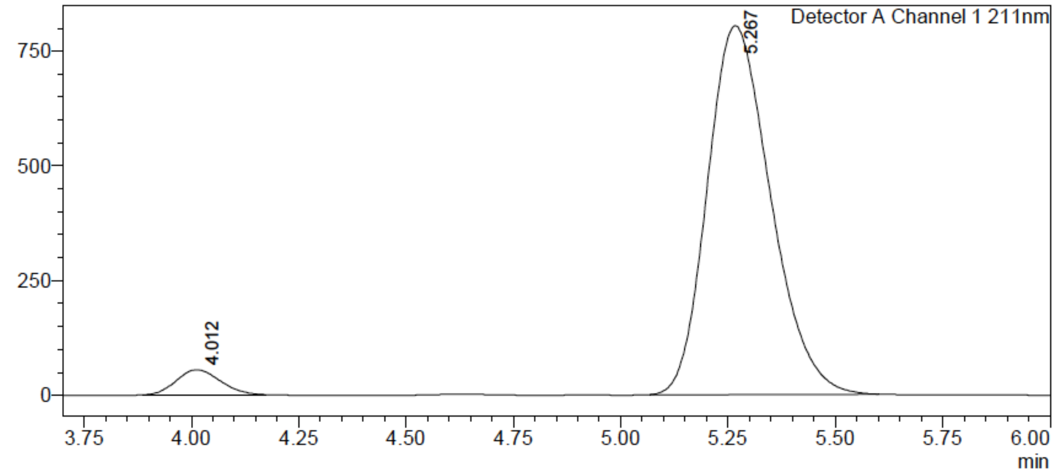

<Peak Table>

Detector A Channel 1 211nm

Peak#	Ret. Time	Area%
1	4.012	4.500
2	5.267	95.500
Total		100.000

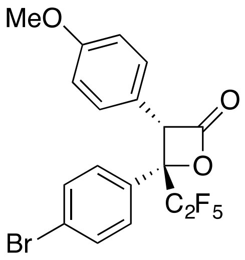

Chiralcel OD-H (97:3 Hexane : IPA, flow rate 1.5 mLmin^{-1}, 211 nm, 40 °C) t_R(3*R*,4*R*): 3.6 min, t_R(3*S*,4*S*): 4.4 min, 96:4 er.

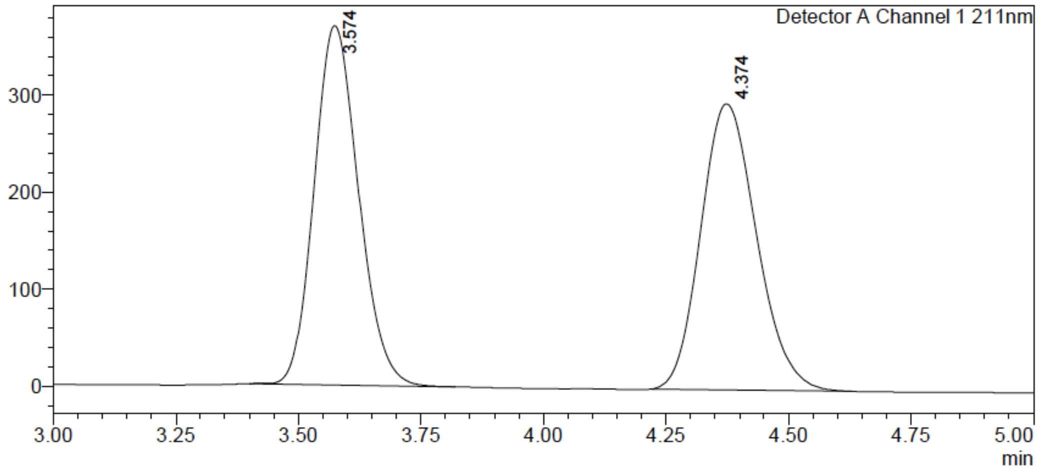

<Peak Table>

Detector A Channel 1 211nm

Peak#	Ret. Time	Area%
1	3.574	49.999
2	4.374	50.001
Total		100.000

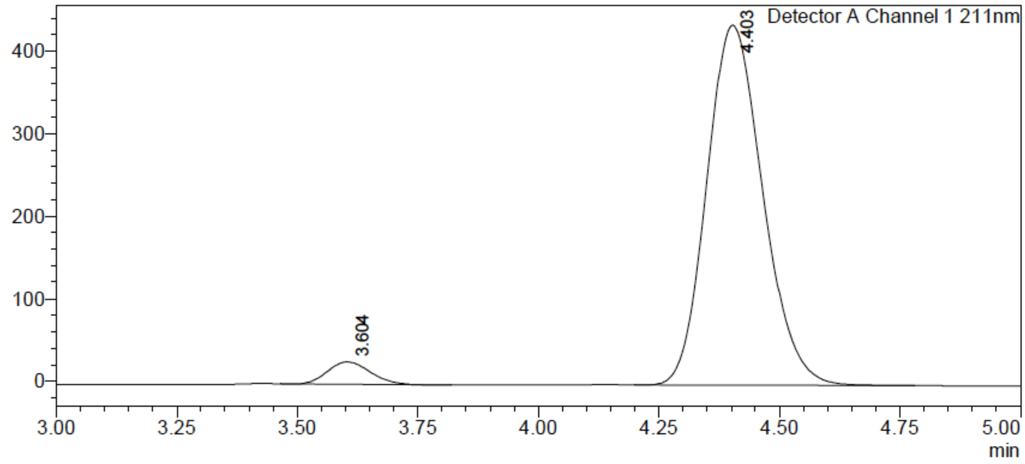

<Peak Table>

Detector A Channel 1 211nm

Peak#	Ret. Time	Area%
1	3.604	4.491
2	4.403	95.509
Total		100.000

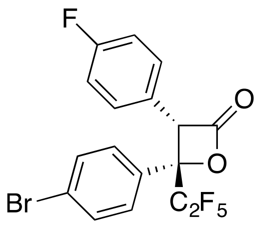

Chiralcel OD-H (97:3 Hexane:IPA, flow rate 1.5 mLmin^{-1}, 211 nm, 40 °C) t_R(3*R*,4*R*): 3.0 min, t_R(3*S*,4*S*): 3.5 min, 95:5 er.

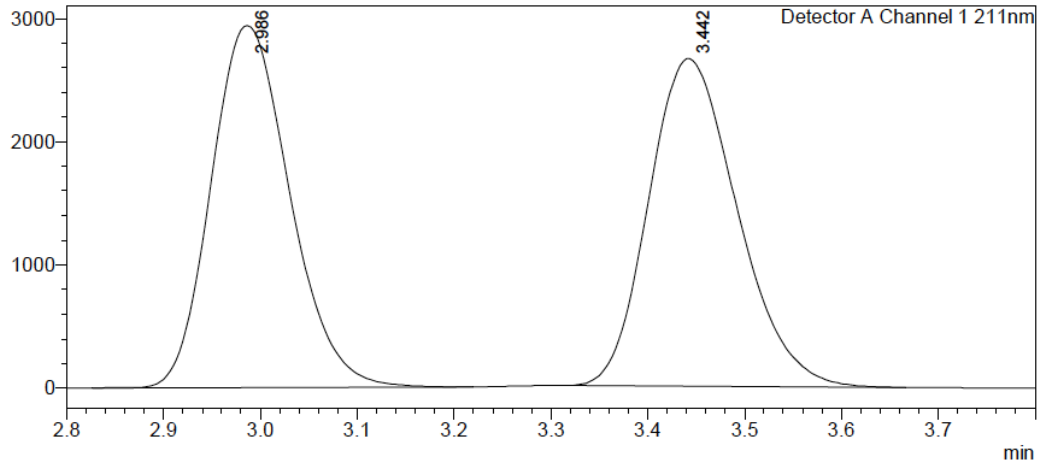

<Peak Table>

Detector A Channel 1 211nm

Peak#	Ret. Time	Area%
1	2.986	49.474
2	3.442	50.526
Total		100.000

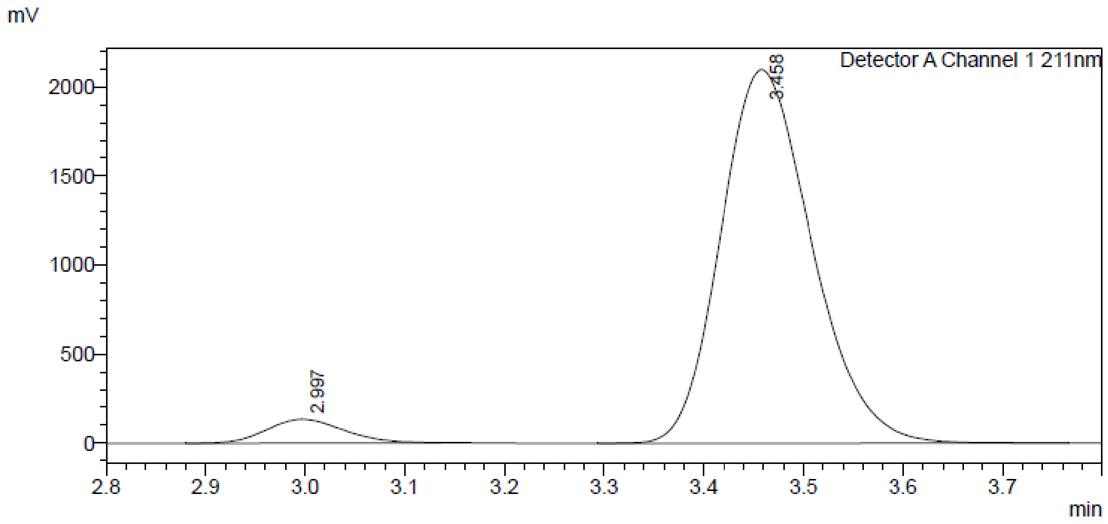

<Peak Table>

Detector A Channel 1 211nm

Peak#	Ret. Time	Area%
1	2.997	5.059
2	3.458	94.941
Total		100.000

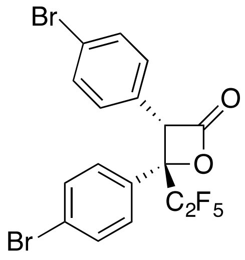

Chiralcel OD-H (97:3 Hexane:IPA, flow rate 1.5 mLmin^{-1}, 211 nm, 40 °C) t_R(3*R*,4*R*): 3.6 min, t_R(3*S*,4*S*): 4.1 min, 95:5 er.

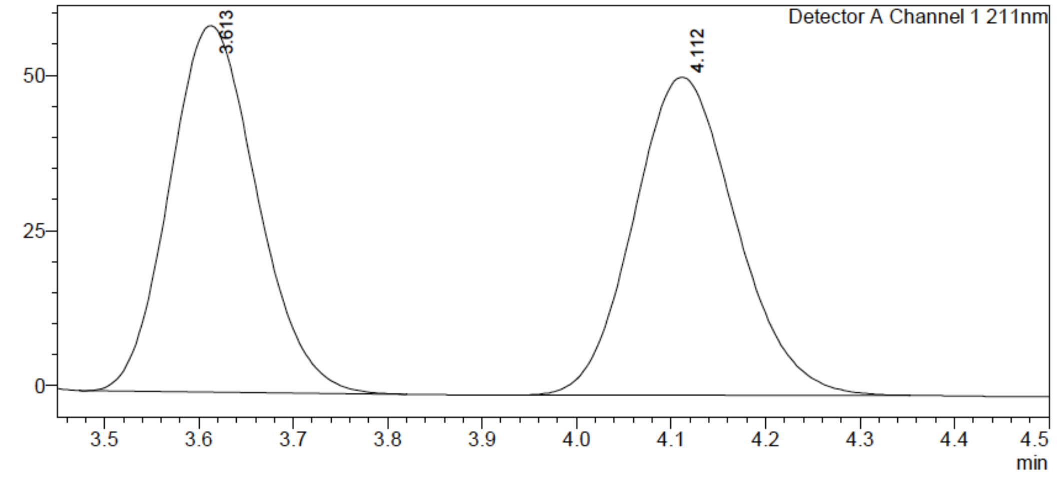

<Peak Table>

Detector A Channel 1 211nm

Peak#	Ret. Time	Area%
1	3.613	49.614
2	4.112	50.386
Total		100.000

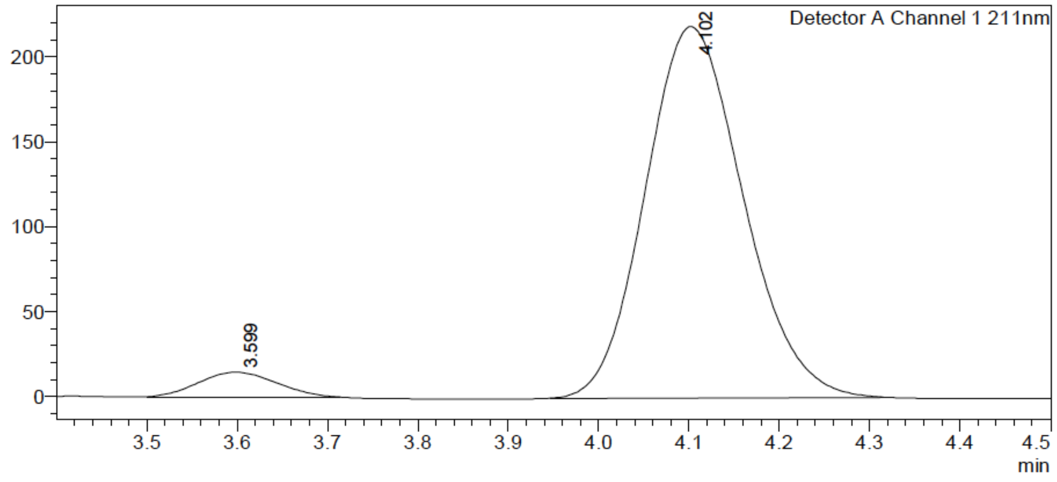

<Peak Table>

Detector A Channel 1 211nm

Peak#	Ret. Time	Area%
1	3.599	5.004
2	4.102	94.996
Total		100.000

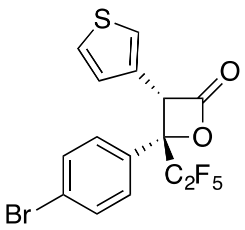

Chiralcel OD-H (97:3 Hexane:IPA, flow rate 1.5 mLmin^{-1}, 211 nm, 40 °C) t_R(3*R*,4*R*): 3.4 min, t_R(3*S*,4*S*): 4.6 min, 93:7 er.

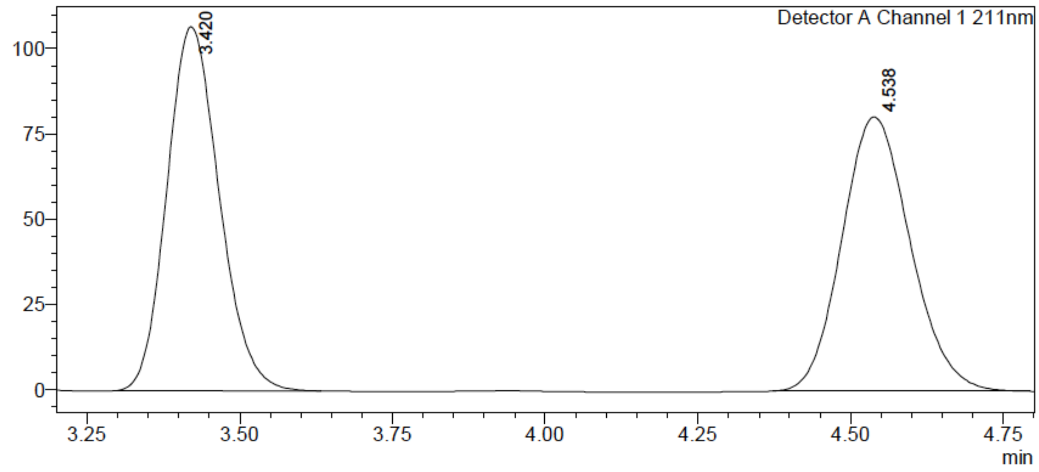

<Peak Table>

Detector A Channel 1 211nm

Peak#	Ret. Time	Area%
1	3.420	50.431
2	4.538	49.569
Total		100.000

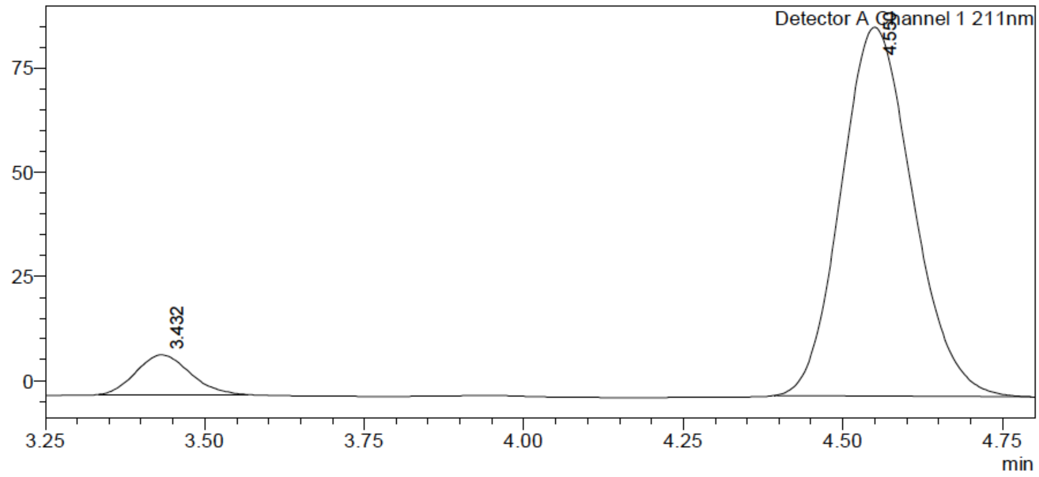

<Peak Table>

Detector A Channel 1 211nm

Peak#	Ret. Time	Area%
1	3.432	7.431
2	4.550	92.569
Total		100.000

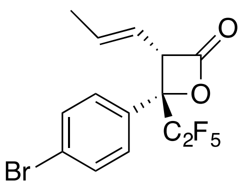

Chiralpak AD-H (99:1 Hexane:IPA, flow rate 1.5 mLmin^{-1}, 211 nm, 40 °C) t_R(3*S*,4*S*): 2.9 min, t_R(3*R*,4*R*): 3.3 min, 83:17 er.

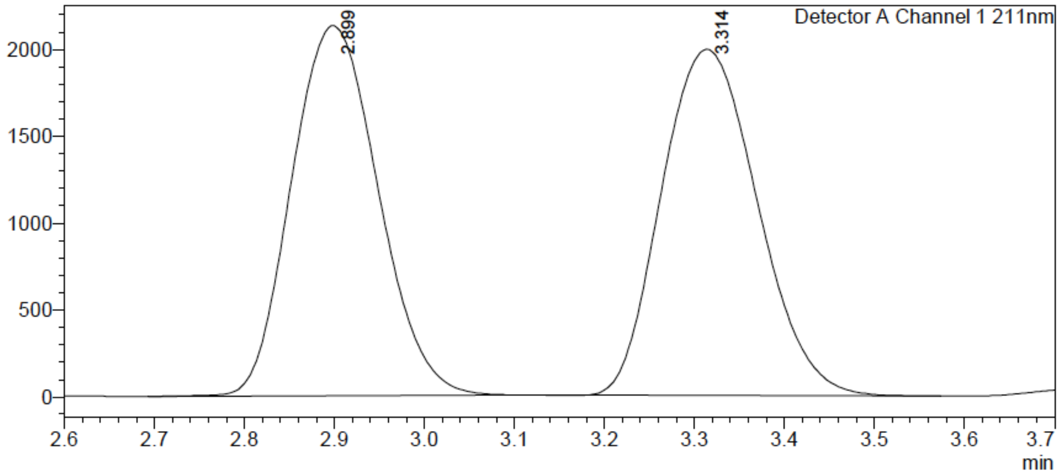

<Peak Table>

Detector A Channel 1 211nm

Peak#	Ret. Time	Area%
1	2.899	49.123
2	3.314	50.877
Total		100.000

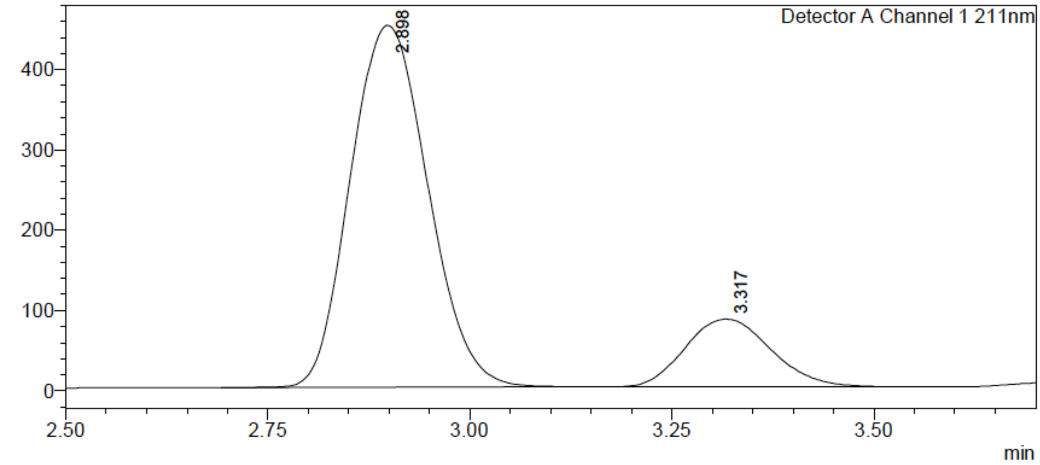

<Peak Table>

Detector A Channel 1 211nm

Peak#	Ret. Time	Area%
1	2.898	82.879
2	3.317	17.121
Total		100.000

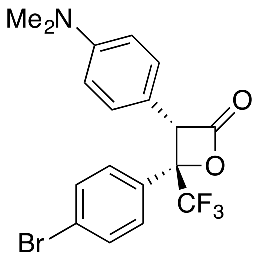

Chiralcel OD-H (97:3 Hexane:IPA, flow rate 1.5 mLmin^{-1}, 211 nm, 40 °C) t_R(3*R*,4*R*): 4.7 min, t_R(3*S*,4*S*): 6.2 min, 95:5 er.

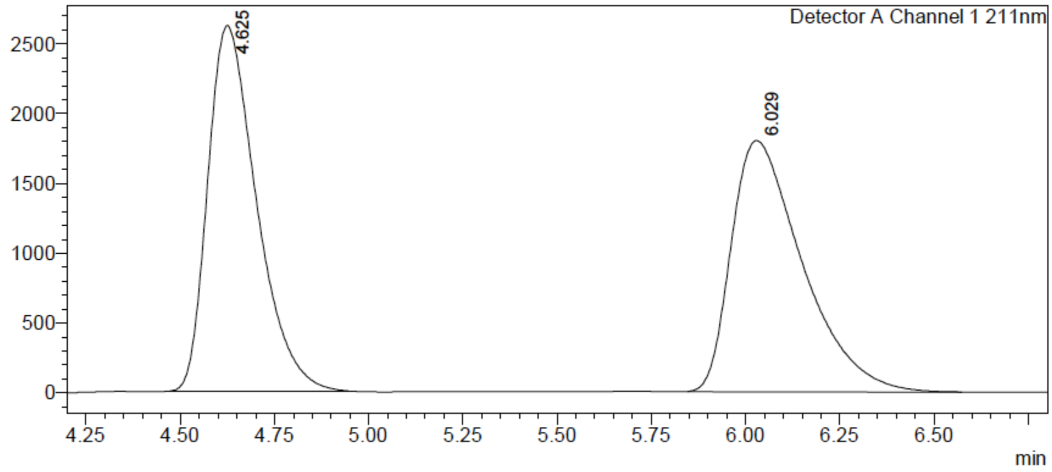

<Peak Table>

Detector A Channel 1 211nm

Peak#	Ret. Time	Area%
1	4.625	50.048
2	6.029	49.952
Total		100.000

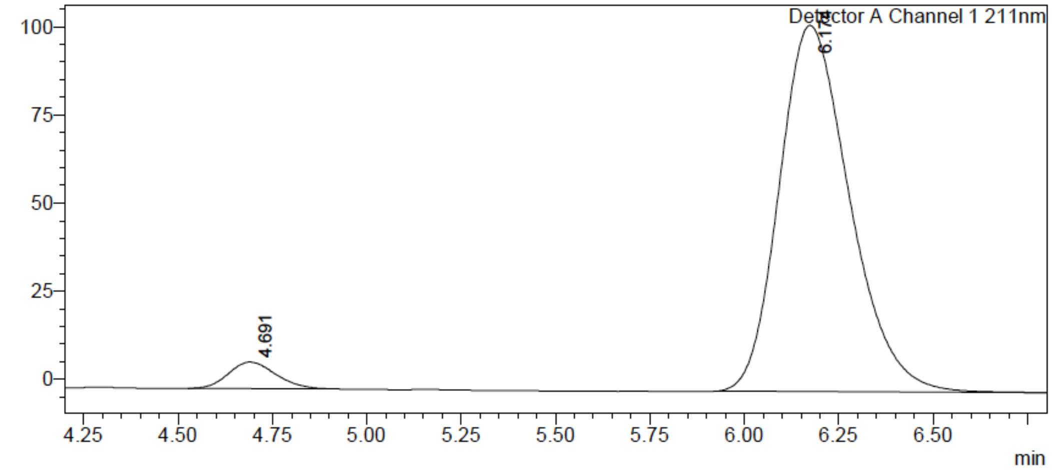

<Peak Table>

Detector A Channel 1 211nm

Peak#	Ret. Time	Area%
1	4.691	4.627
2	6.174	95.373
Total		100.000

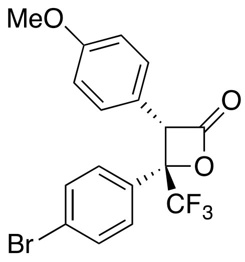

Chiralcel OD-H (97:3 Hexane:IPA, flow rate 1.5 mLmin^{-1}, 211 nm, 40 °C) t_R(3*R*,4*R*): 4.1 min, t_R(3*S*,4*S*): 4.5 min, 92:8 er.

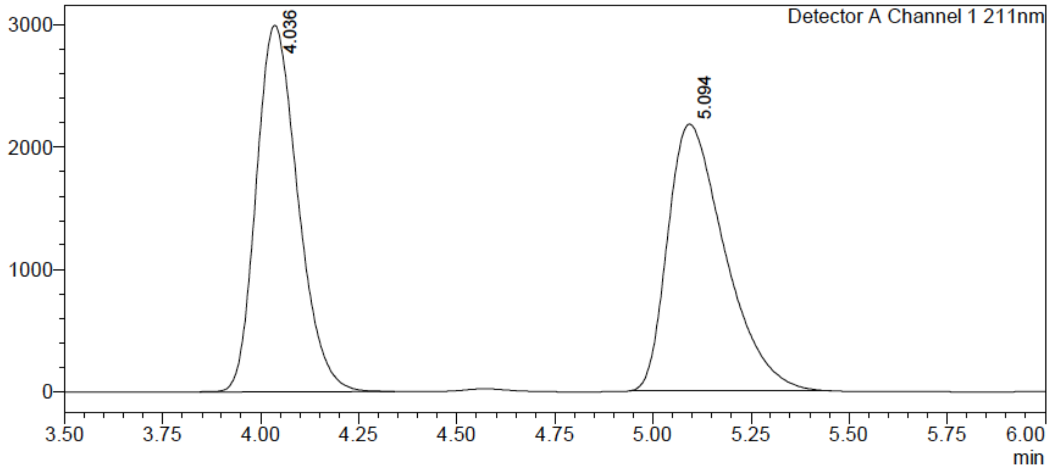

<Peak Table>

Detector A Channel 1 211nm

Peak#	Ret. Time	Area%
1	4.036	49.896
2	5.094	50.104
Total		100.000

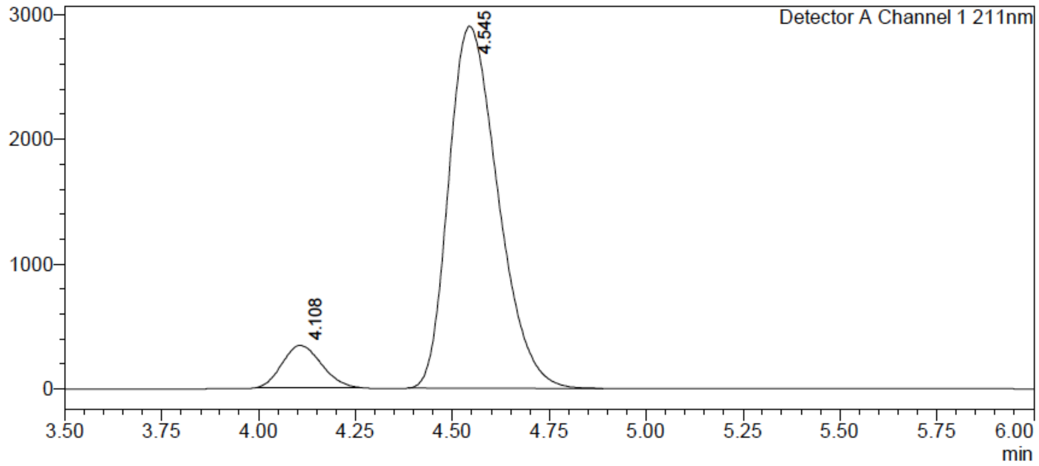

<Peak Table>

Detector A Channel 1 211nm

Peak#	Ret. Time	Area%
1	4.108	8.491
2	4.545	91.509
Total		100.000

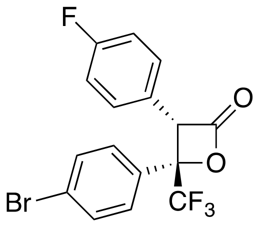

Chiralcel OD-H (97:3 Hexane:IPA, flow rate 1.5 mLmin^{-1}, 211 nm, 40 °C) t_R(3*R*,4*R*): 3.3 min, t_R(3*S*,4*S*): 3.9 min, 96:4 er.

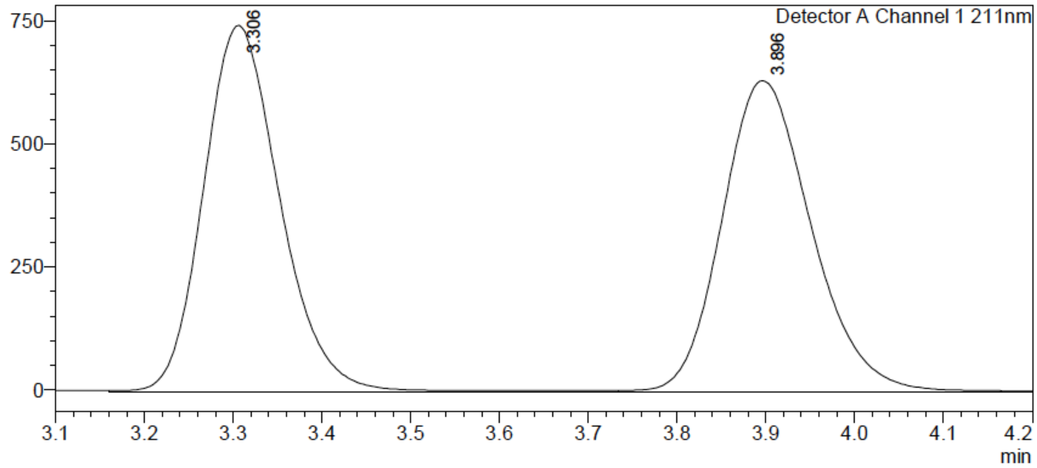

<Peak Table>

Detector A Channel 1 211nm

Peak#	Ret. Time	Area%
1	3.306	50.075
2	3.896	49.925
Total		100.000

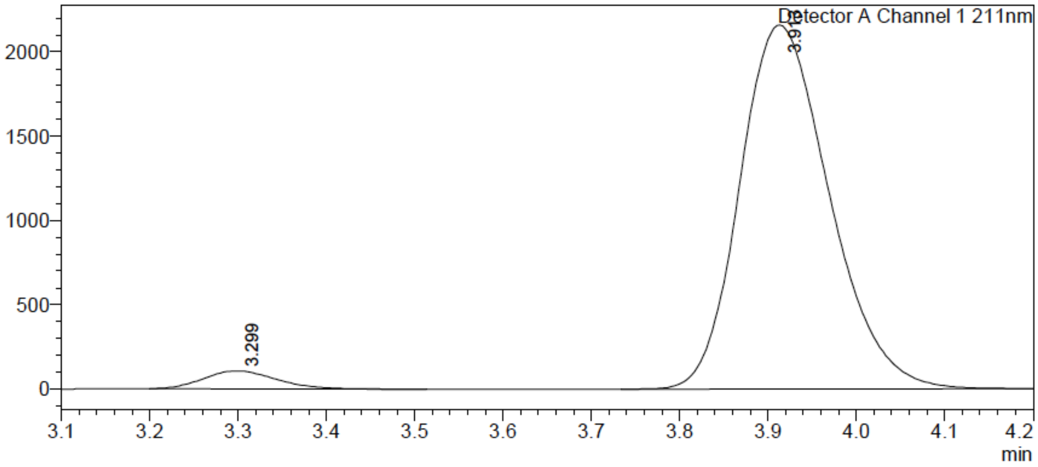

<Peak Table>

Detector A Channel 1 211nm

Peak#	Ret. Time	Area%
1	3.299	3.835
2	3.913	96.165
Total		100.000

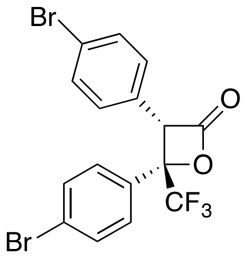

Chiralcel OD-H (97:3 Hexane:IPA, flow rate 1.5 mLmin^{-1}, 211 nm, 40 °C) t_R(3*R*,4*R*): 4.1 min, t_R(3*S*,4*S*): 4.5 min, 91:9 er.

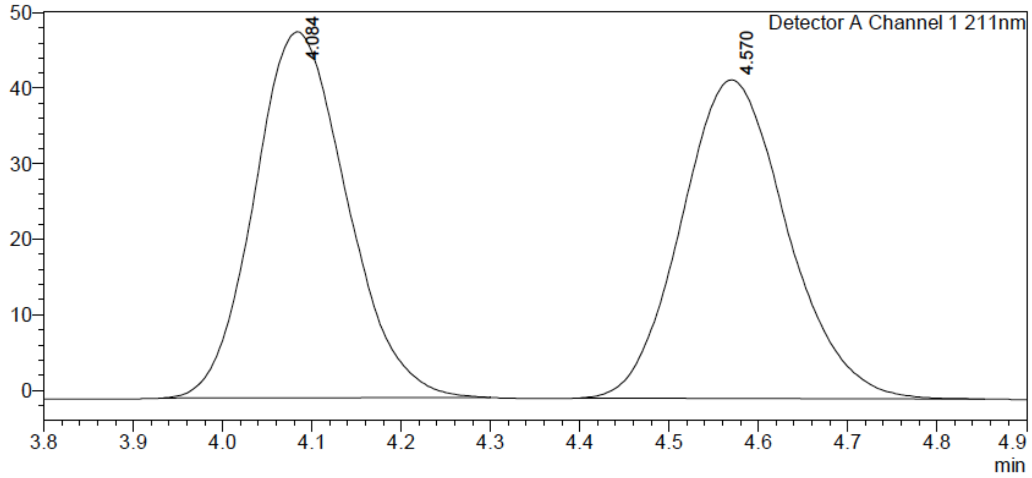

<Peak Table>

Detector A Channel 1 211nm

Peak#	Ret. Time	Area%
1	4.084	50.160
2	4.570	49.840
Total		100.000

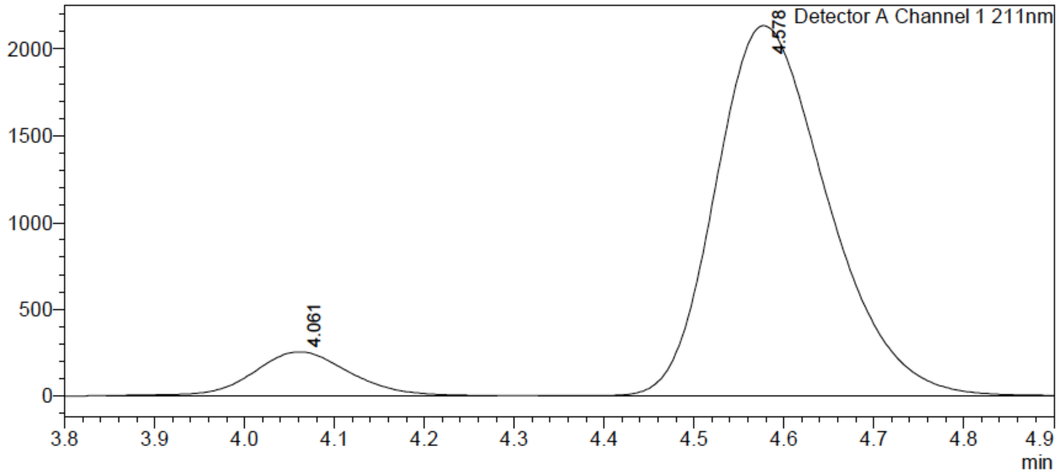

<Peak Table>

Detector A Channel 1 211nm

Peak#	Ret. Time	Area%
1	4.061	9.202
2	4.578	90.798
Total		100.000

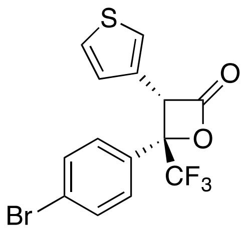

Chiralcel OD-H (97:3 Hexane:IPA, flow rate 1.5 mLmin^{-1}, 211 nm, 40 °C) t_R(3*R*,4*R*): 3.9 min, t_R(3*S*,4*S*): 4.7 min, 95:5 er.

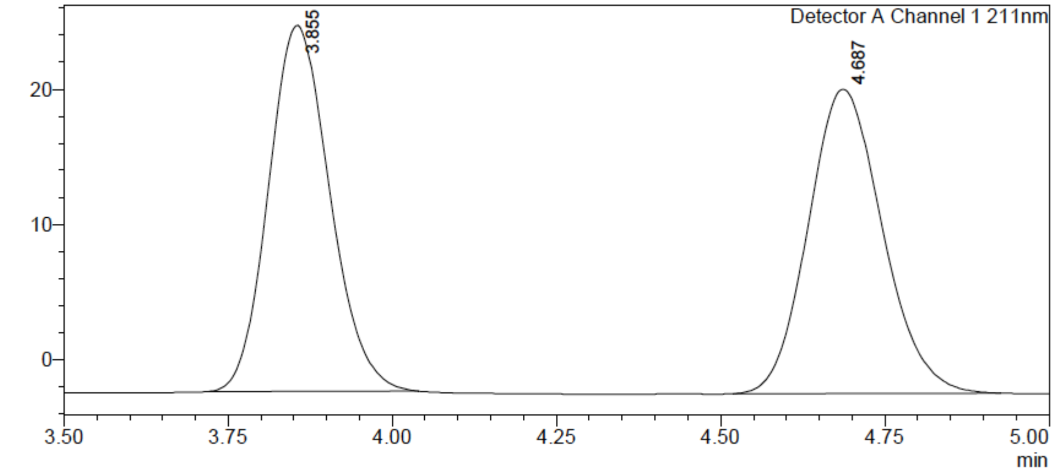

<Peak Table>

Detector A Channel 1 211nm

Peak#	Ret. Time	Area%
1	3.855	49.869
2	4.687	50.131
Total		100.000

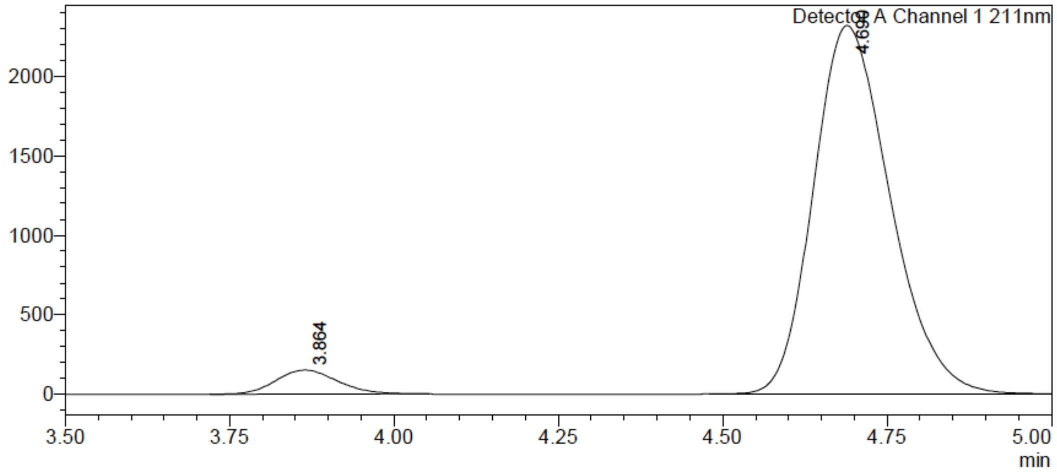

<Peak Table>

Detector A Channel 1 211nm

Peak#	Ret. Time	Area%
1	3.864	5.012
2	4.690	94.988
Total		100.000

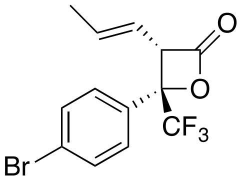

Chiralpak AD-H (99:1 Hexane:IPA, flow rate 1.5 mLmin^{-1}, 211 nm, 40 °C) t_R(3*S*,4*S*): 3.2 min, t_R(3*R*,4*R*): 3.5 min, 98:2 er.

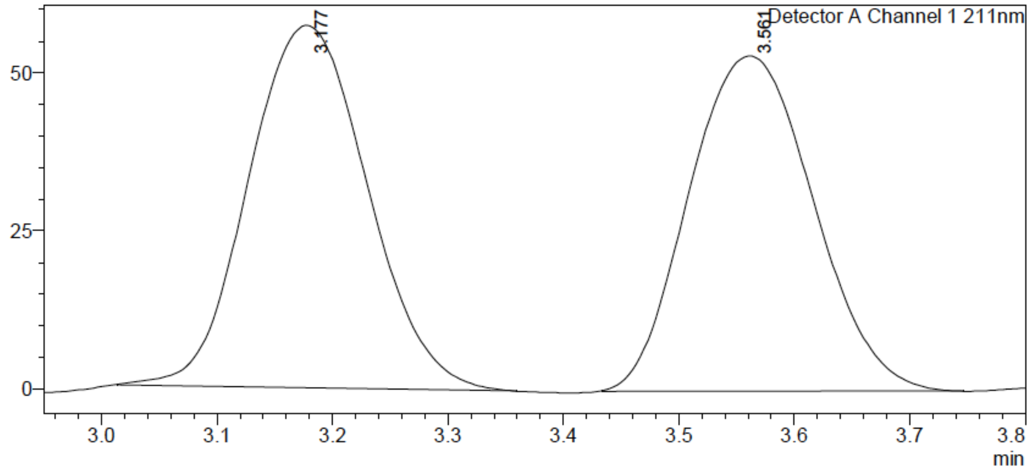

<Peak Table>

Detector A Channel 1 211nm

Peak#	Ret. Time	Area%
1	3.177	50.832
2	3.561	49.168
Total		100.000

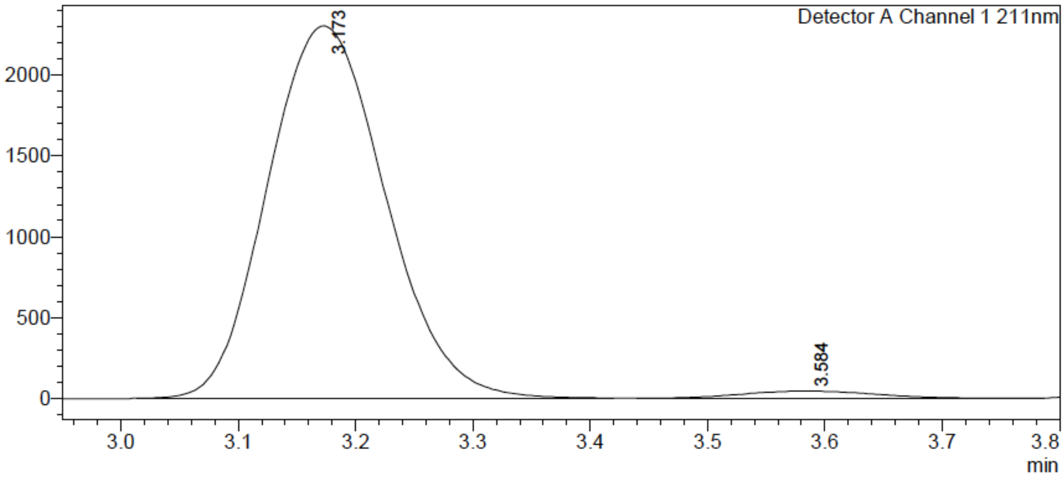

<Peak Table>

Detector A Channel 1 211nm

Peak#	Ret. Time	Area%
1	3.173	97.934
2	3.584	2.066
Total		100.000

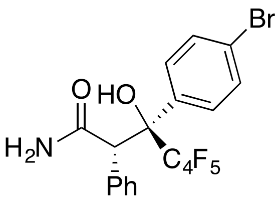

Chiralcel OD-H (95:5 hexane:IPA, flow rate 1.5 mLmin^{-1}, 211 nm, 40 °C) t_R(2*R*,3*R*): 11.3 min, t_R(2*S*,3*S*): 22.2 min, 97:3 er.

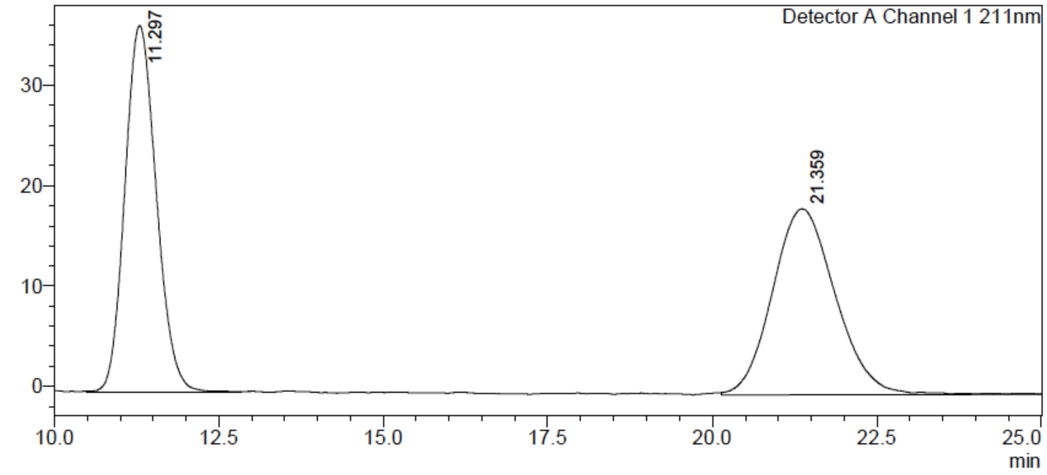

<Peak Table>

Detector A Channel 1 211nm

Peak#	Ret. Time	Area%
1	11.297	49.856
2	21.359	50.144
Total		100.000

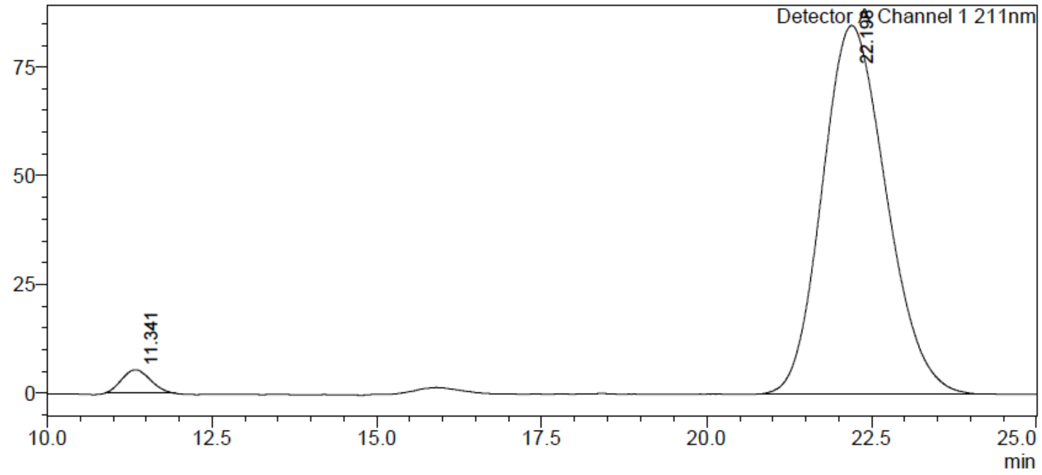

<Peak Table>

Detector A Channel 1 211nm

Peak#	Ret. Time	Area%
1	11.341	2.768
2	22.198	97.232
Total		100.000

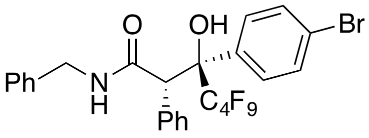

Chiralpak AD-H (97:3 hexane:IPA, flow rate 1.5 mLmin^{-1}, 211 nm, 40 °C) t_R(2*R*,3*R*): 7.3 min, t_R(2*S*,3*S*): 19.4 min, 94:6 er.

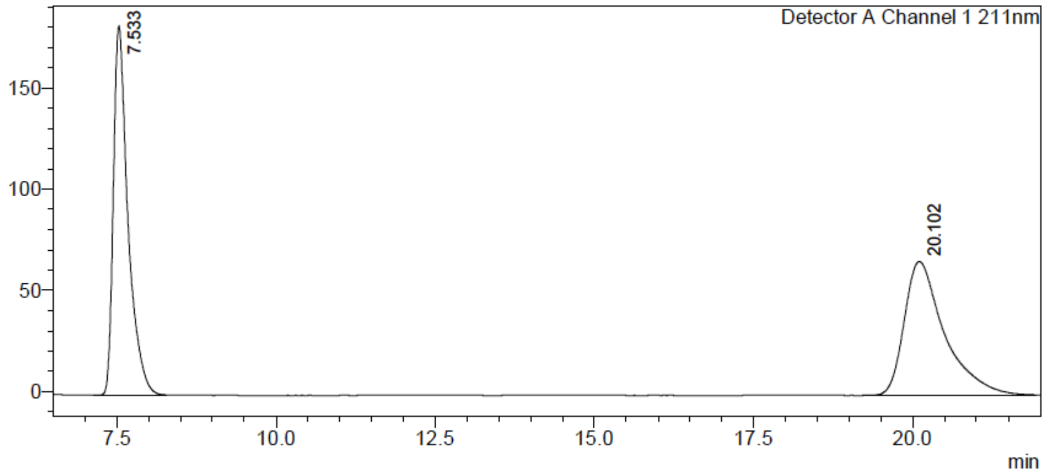

<Peak Table>

Detector A Channel 1 211nm

Peak#	Ret. Time	Area%
1	7.533	50.150
2	20.102	49.850
Total		100.000

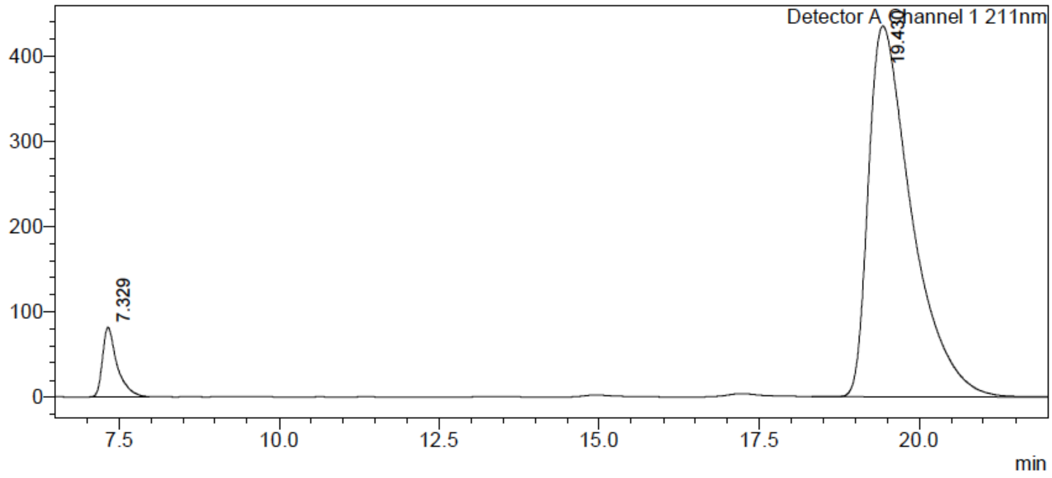

<Peak Table>

Detector A Channel 1 211nm

Peak#	Ret. Time	Area%
1	7.329	5.912
2	19.430	94.088
Total		100.000

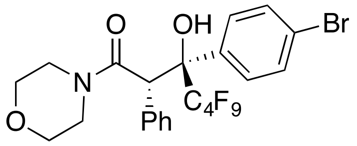

Chiralpak AD-H (97:3 hexane:IPA, flow rate 1.5 mLmin^{-1}, 211 nm, 40 °C) t_R(2*R*,3*R*): 4.6 min, t_R(2*S*,3*S*): 5.2 min, 98:2 er.

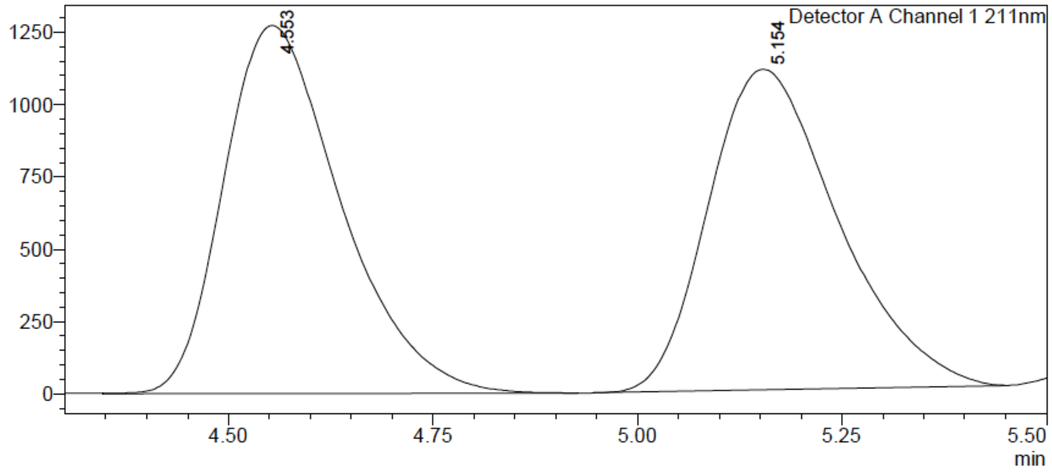

<Peak Table>

Detector A Channel 1 211nm

Peak#	Ret. Time	Area%
1	4.553	50.857
2	5.154	49.143
Total		100.000

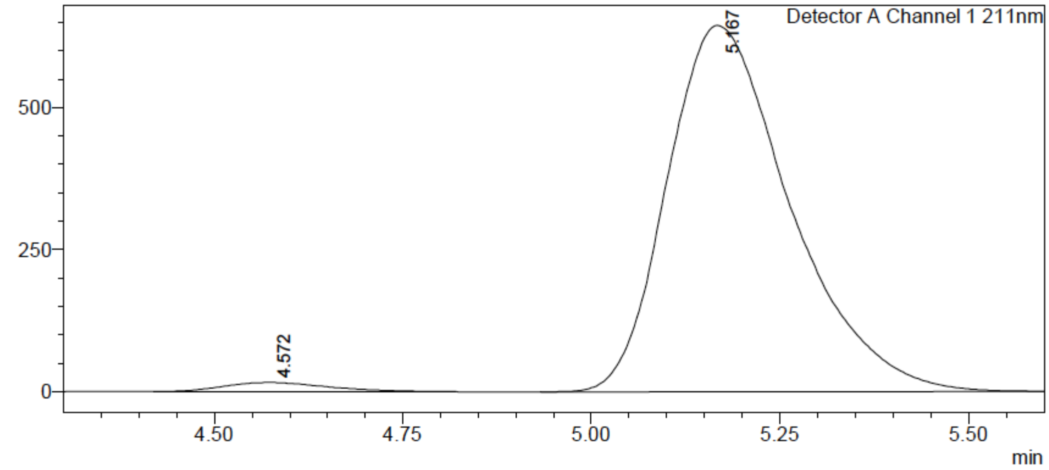

<Peak Table>

Detector A Channel 1 211nm

Peak#	Ret. Time	Area%
1	4.572	2.064
2	5.167	97.936
Total		100.000

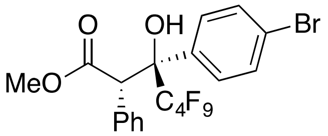

Chiralpak AD-H (99:1 hexane:IPA, flow rate 1.5 mLmin^{-1}, 211 nm, 40 °C) t_R(2*S*,3*S*): 3.2 min, t_R(2*R*,3*R*): 3.8 min, 98:2 er.

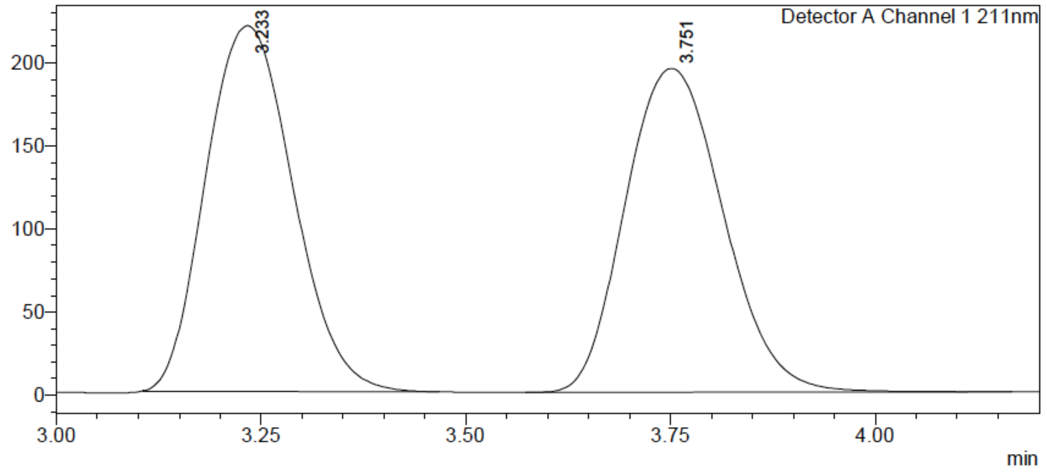

<Peak Table>

Detector A Channel 1 211nm

Peak#	Ret. Time	Area%
1	3.233	50.176
2	3.751	49.824
Total		100.000

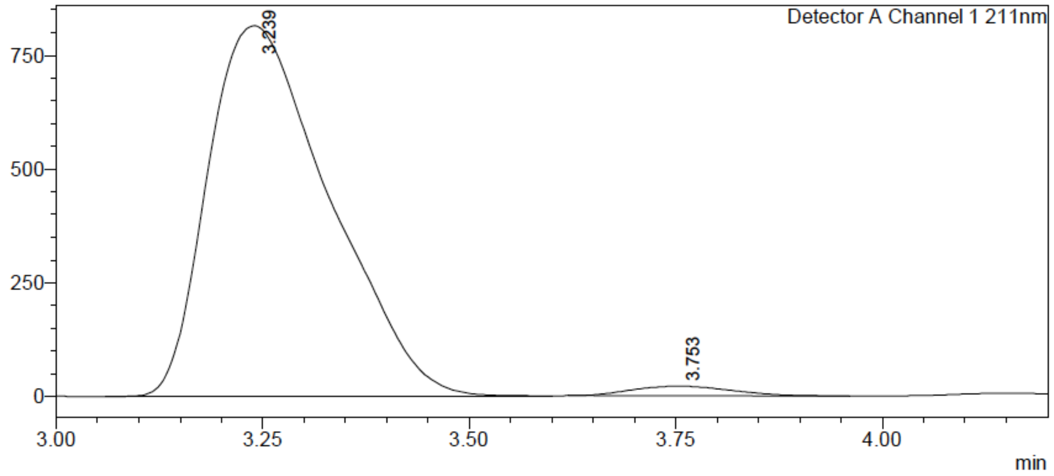

<Peak Table>

Detector A Channel 1 211nm

Peak#	Ret. Time	Area%
1	3.239	97.901
2	3.753	2.099
Total		100.000

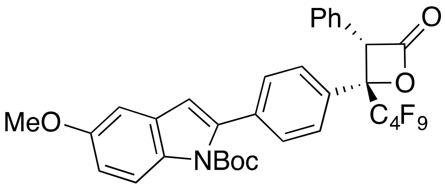

Chiralcel OD-H (97:3 Hexane:IPA, flow rate 1.5 mLmin^{-1}, 211 nm, 40 °C) t_R(2*R*,3*R*): 3.7 min, t_R(2*S*,3*S*): 4.8 min, 97:3 er.

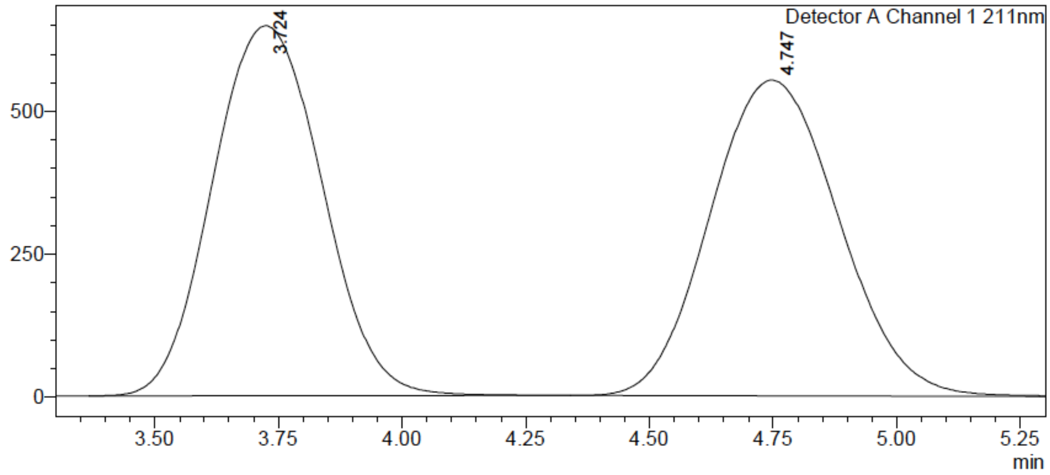

<Peak Table>

Detector A Channel 1 211nm

Peak#	Ret. Time	Area%
1	3.724	49.924
2	4.747	50.076
Total		100.000

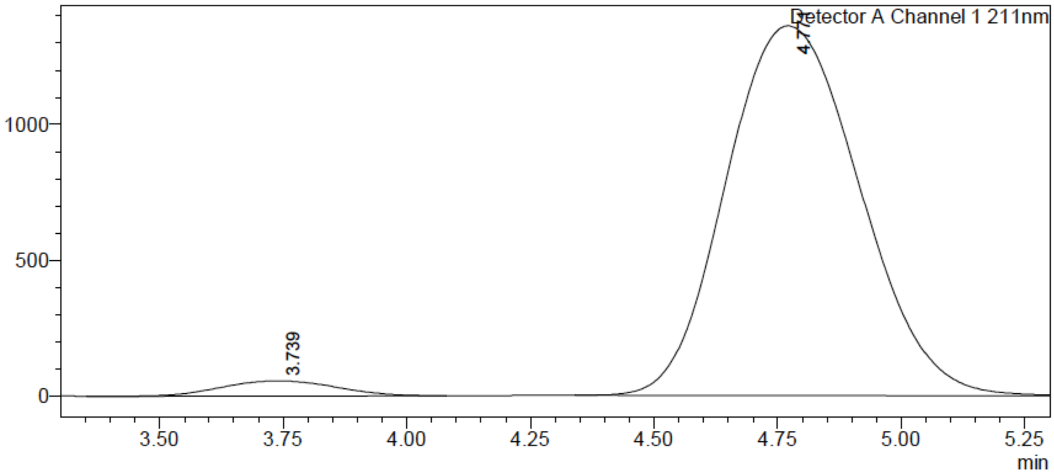

<Peak Table>

Detector A Channel 1 211nm

Peak#	Ret. Time	Area%
1	3.739	3.349
2	4.771	96.651
Total		100.000

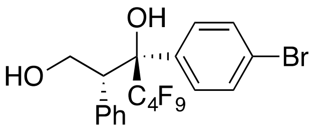

Chiralcel OD-H (97:3 Hexane:IPA, flow rate 1.5 mLmin^{-1}, 211 nm, 40 °C) t_R(2*S*,3*R*): 16.7 min, t_R(2*R*,3*S*): 26.5 min, 97:3 er.

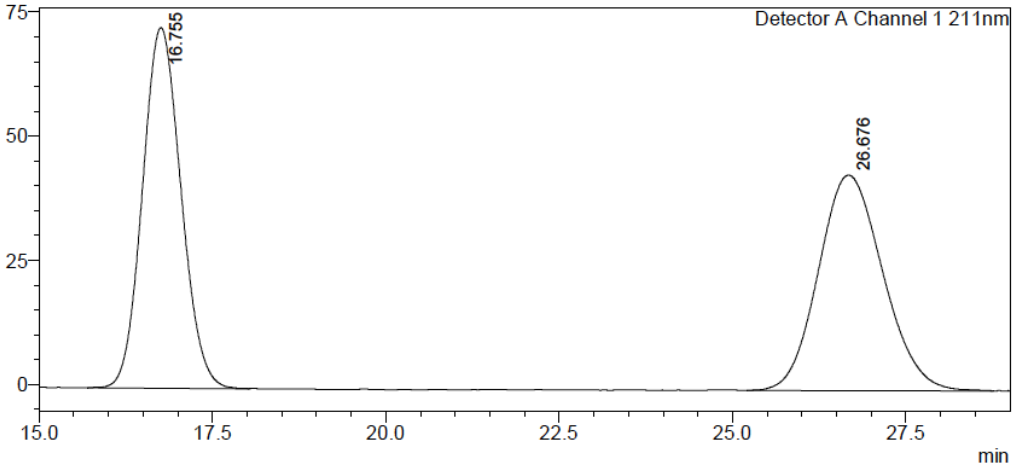

<Peak Table>

Detector A Channel 1 211nm

Peak#	Ret. Time	Area%
1	16.755	50.002
2	26.676	49.998
Total		100.000

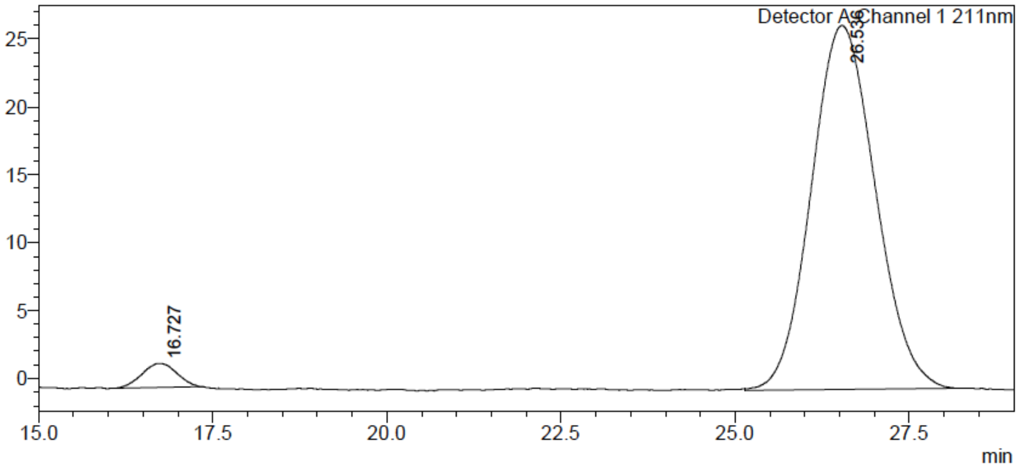

<Peak Table>

Detector A Channel 1 211nm

Peak#	Ret. Time	Area%
1	16.727	3.449
2	26.536	96.551
Total		100.000

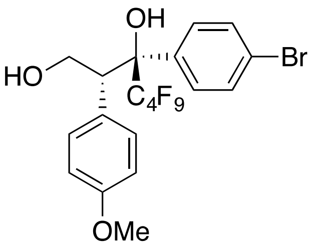

Chiralcel OD-H (97:3 Hexane:IPA, flow rate 1.5 mLmin^{-1}, 211 nm, 40 °C) t_R(2*R*,3*S*): 8.3 min, t_R(2*S*,3*R*): 10.8 min, 97:3 er.

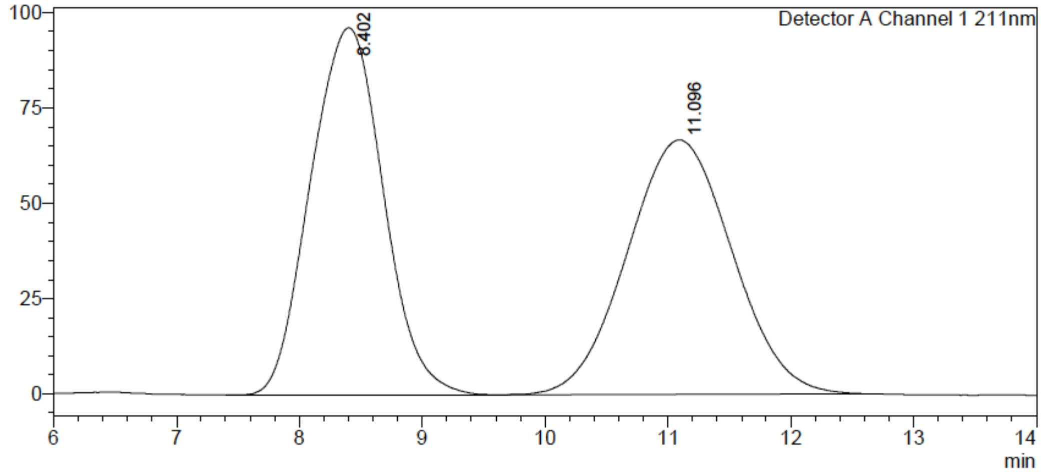

<Peak Table>

Detector A Channel 1 211nm

Peak#	Ret. Time	Area%
1	8.402	50.027
2	11.096	49.973
Total		100.000

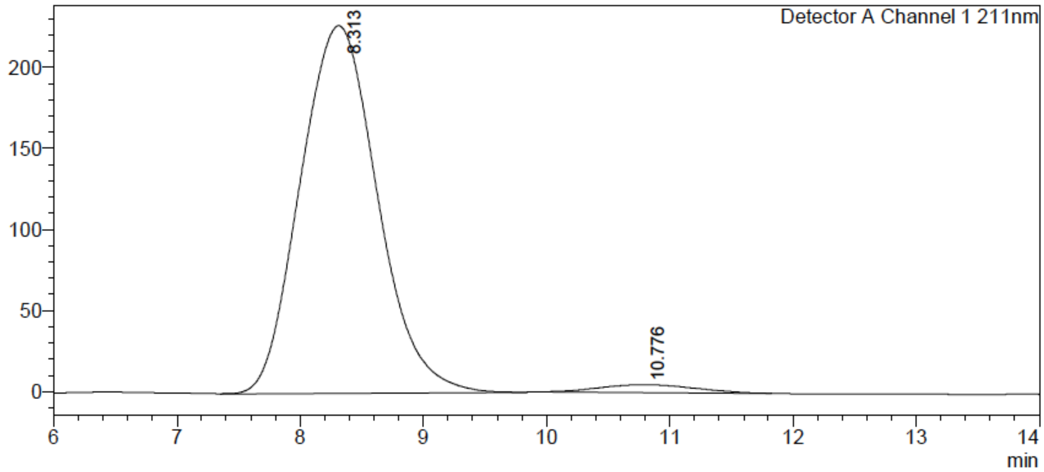

<Peak Table>

Detector A Channel 1 211nm

Peak#	Ret. Time	Area%
1	8.313	97.345
2	10.776	2.655
Total		100.000

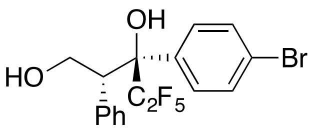

Chiralcel OD-H (97:3 Hexane:IPA, flow rate 1.5 mLmin^{-1}, 211 nm, 40 °C) t_R(2*S*,3*R*): 20.9 min, t_R(2*R*,3*S*): 30.7 min, 94:6 er.

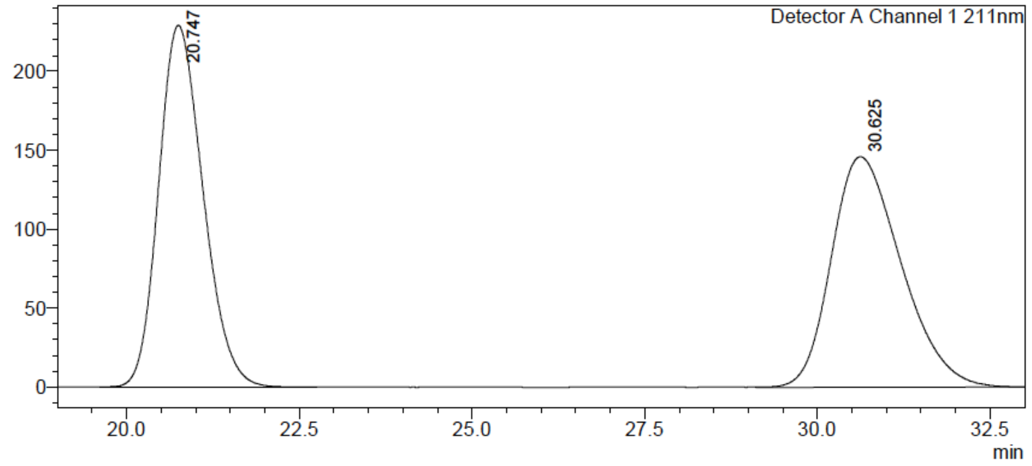

<Peak Table>

Detector A Channel 1 211nm

Peak#	Ret. Time	Area%
1	20.747	50.036
2	30.625	49.964
Total		100.000

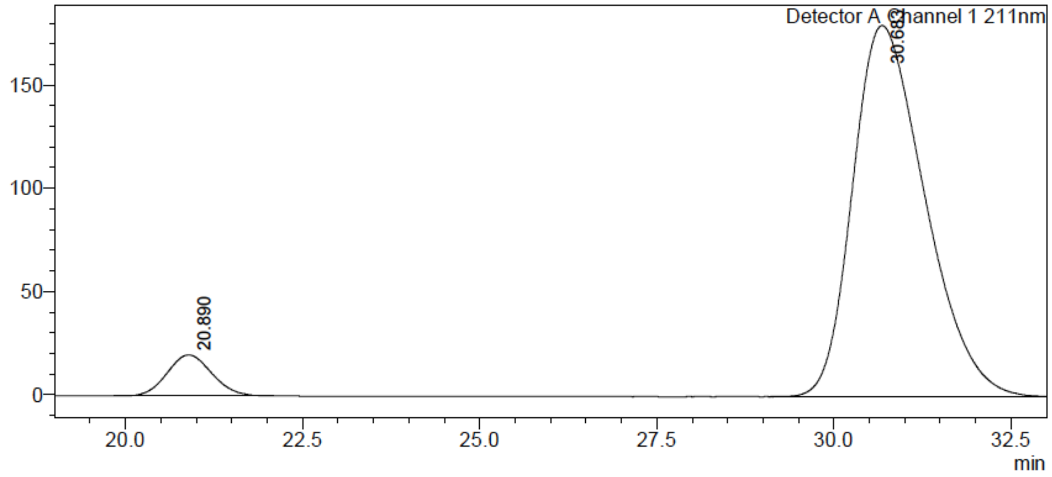

<Peak Table>

Detector A Channel 1 211nm

Peak#	Ret. Time	Area%
1	20.890	6.443
2	30.683	93.557
Total		100.000

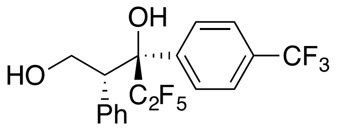

Chiralpak IC (99:1 Hexane:IPA, flow rate 1.5 mLmin^{-1}, 211 nm, 40 °C) t_R(2*S*,3*R*): 3.9 min, t_R(2*R*,3*S*): 4.7 min, 95:5 er.

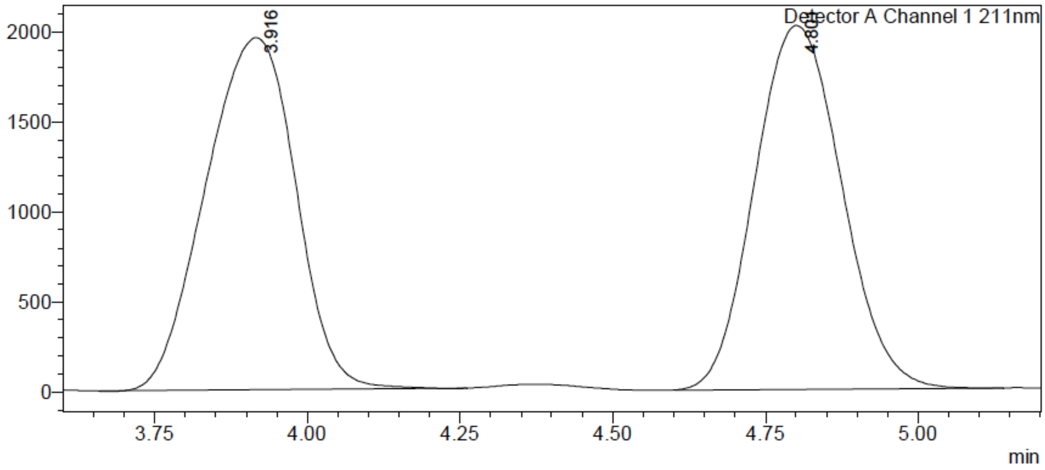

<Peak Table>

Detector A Channel 1 211nm

Peak#	Ret. Time	Area%
1	3.916	50.084
2	4.801	49.916
Total		100.000

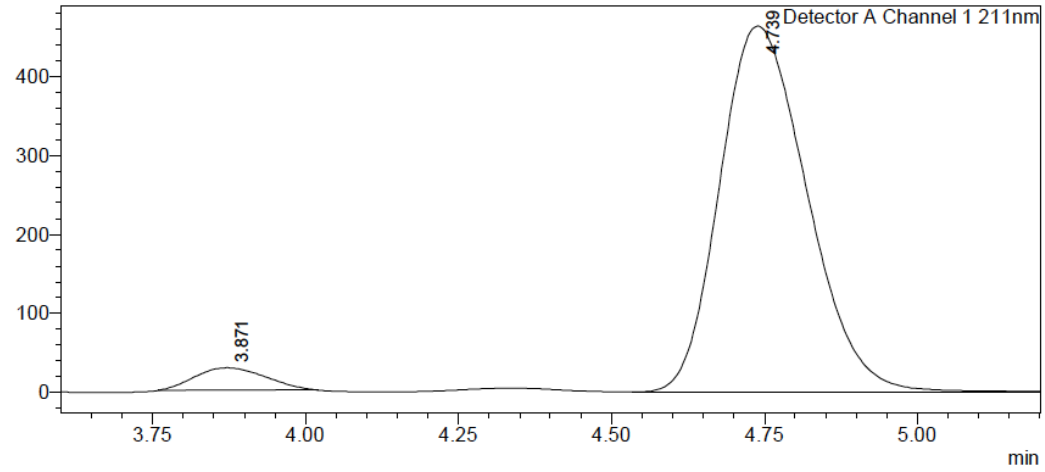

<Peak Table>

Detector A Channel 1 211nm

Peak#	Ret. Time	Area%
1	3.871	4.733
2	4.739	95.267
Total		100.000

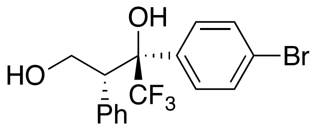

Chiralpak IC (97:3 Hexane:IPA, flow rate 1.5 mLmin^{-1}, 211 nm, 40 °C) t_R(2*S*,3*R*): 4.7 min, t_R(2*R*,3*S*): 5.8 min, 94:6 er.

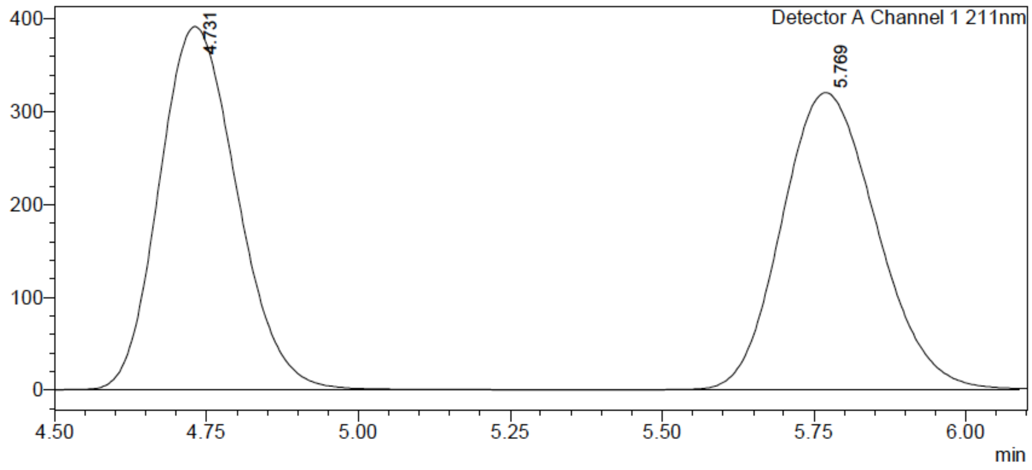

<Peak Table>

Detector A Channel 1 211nm

Peak#	Ret. Time	Area%
1	4.731	50.108
2	5.769	49.892
Total		100.000

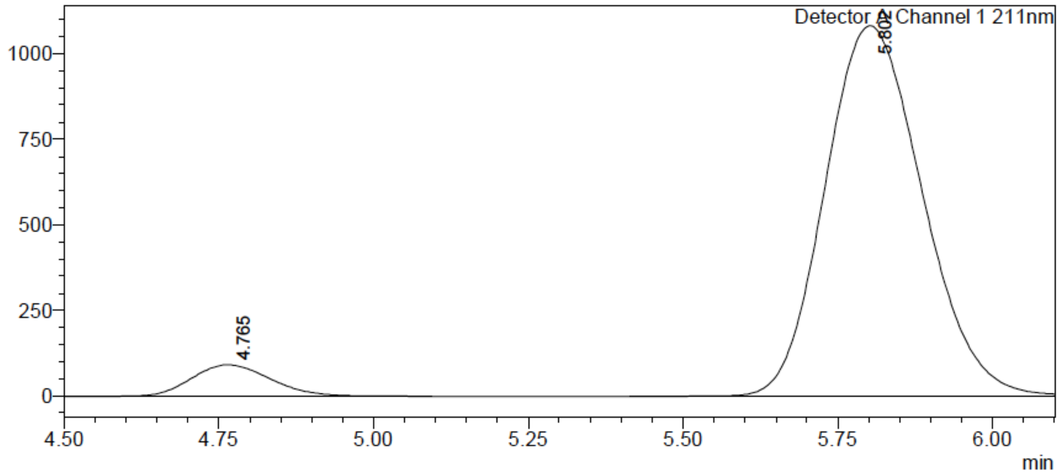

<Peak Table>

Detector A Channel 1 211nm

Peak#	Ret. Time	Area%
1	4.765	6.430
2	5.802	93.570
Total		100.000

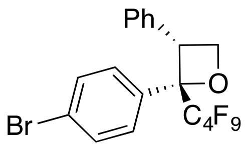

Chiralcel OD-H (97:3 Hexane:IPA, flow rate 1.5 mLmin^{-1}, 211 nm, 40 °C) t_R(2*S*,3*R*): 3.5 min, t_R(2*R*,3*S*): 6.3 min, 97:3 er.

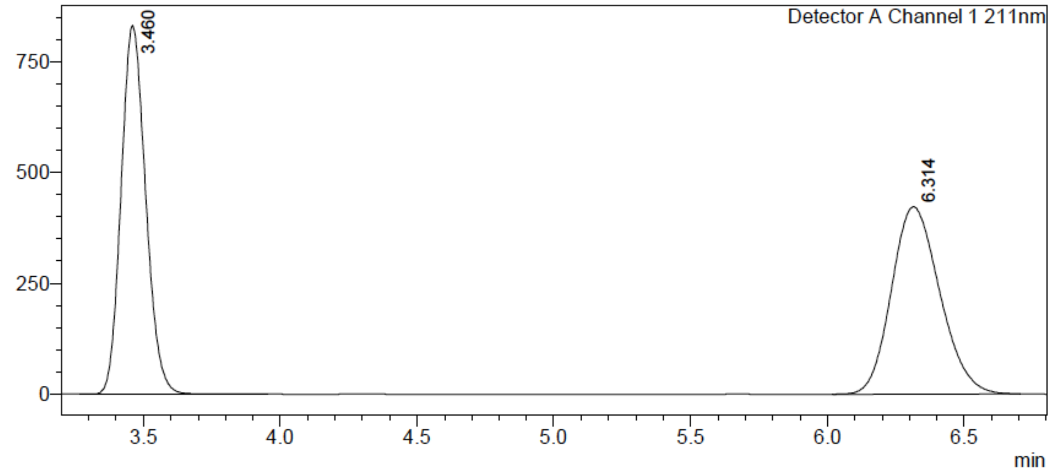

<Peak Table>

Detector A Channel 1 211nm

Peak#	Ret. Time	Area%
1	3.460	49.982
2	6.314	50.018
Total		100.000

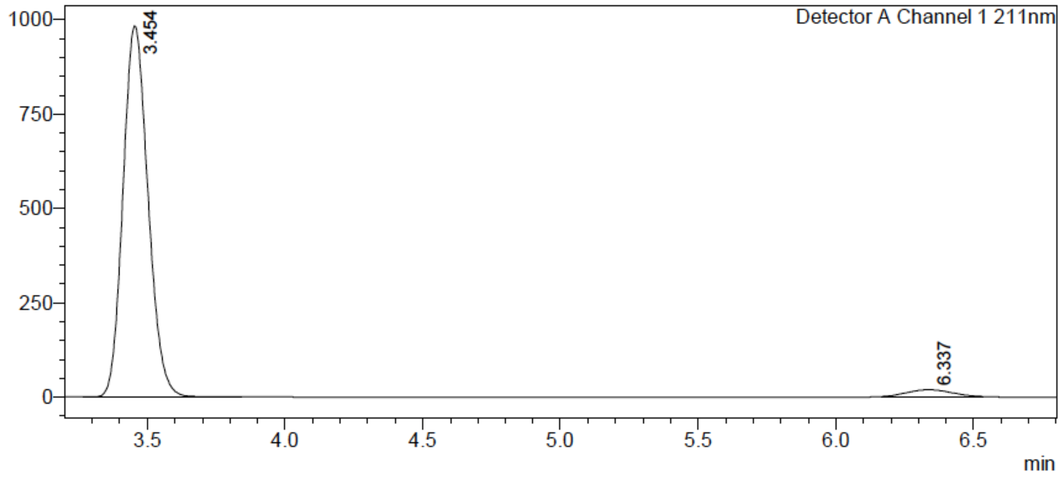

<Peak Table>

Detector A Channel 1 211nm

Peak#	Ret. Time	Area%
1	3.454	96.886
2	6.337	3.114
Total		100.000

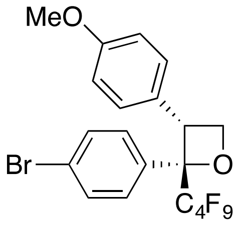

Chiralcel OD-H (97:3 Hexane:IPA, flow rate 1.5 mLmin^{-1}, 211 nm, 40 °C) t_R(2*S*,3*R*): 3.7 min, t_R(2*R*,3*S*): 5.1 min, 97:3 er.

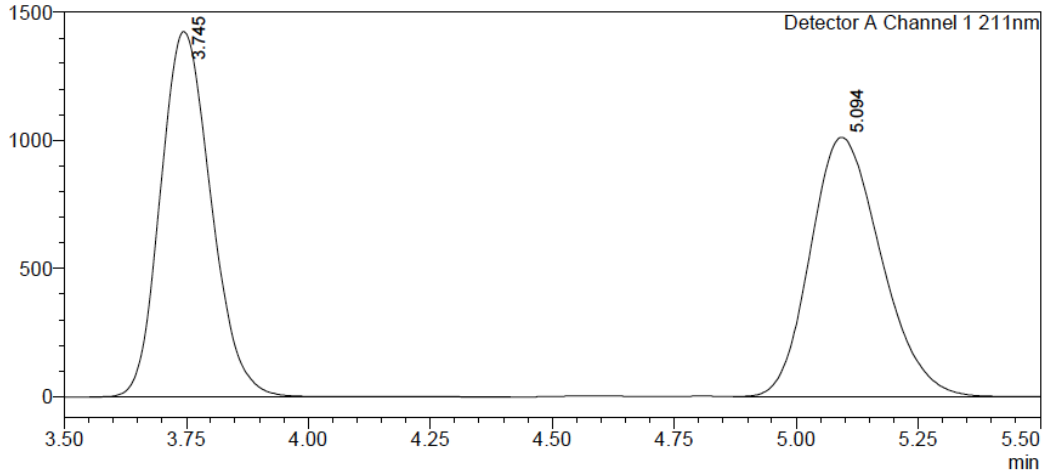

<Peak Table>

Detector A Channel 1 211nm

Peak#	Ret. Time	Area%
1	3.745	50.011
2	5.094	49.989
Total		100.000

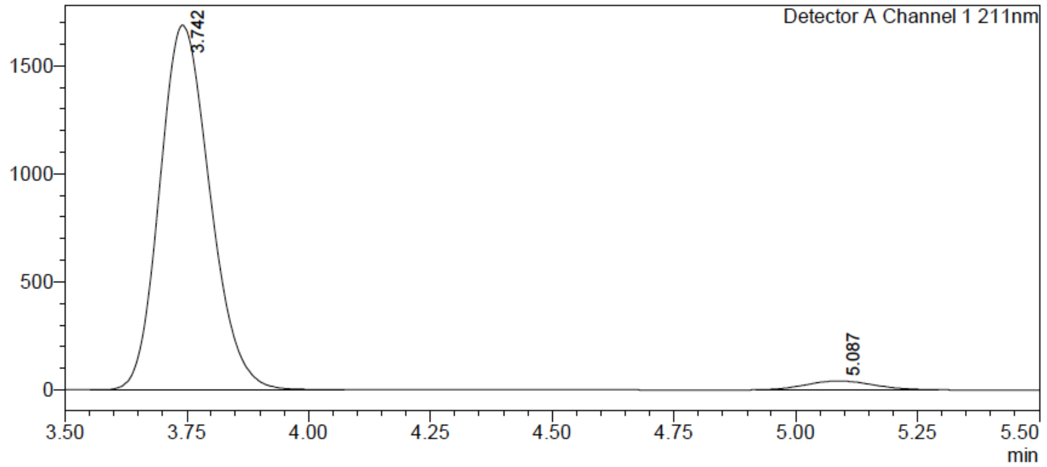

<Peak Table>

Detector A Channel 1 211nm

Peak#	Ret. Time	Area%
1	3.742	96.863
2	5.087	3.137
Total		100.000

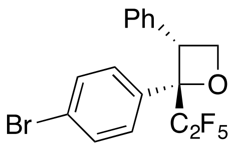

Chiralcel OD-H (97:3 Hexane:IPA, flow rate 1.5 mLmin^{-1}, 211 nm, 40 °C)
t_R(2*S*,3*R*): 4.2 min, t_R(2*R*,3*S*): 6.2 min, 94:6 er.

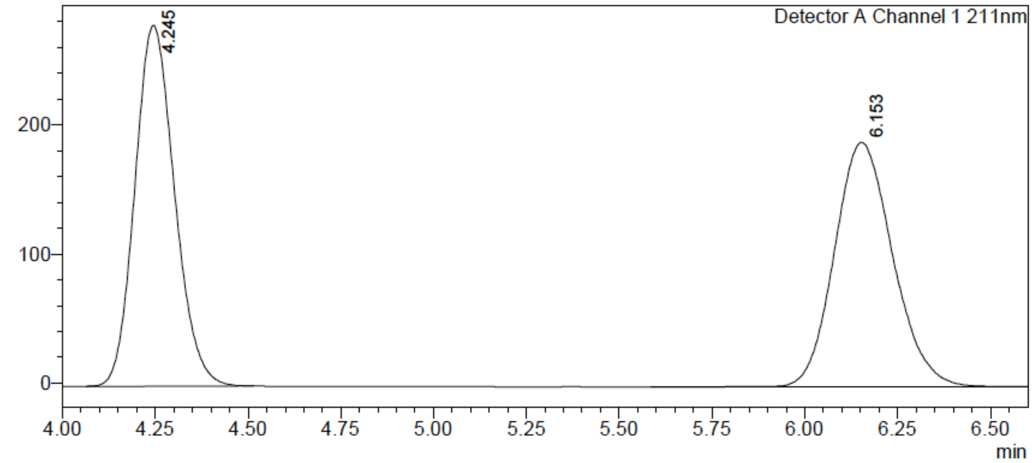

<Peak Table>

Detector A Channel 1 211nm

Peak#	Ret. Time	Area%
1	4.245	50.109
2	6.153	49.891
Total		100.000

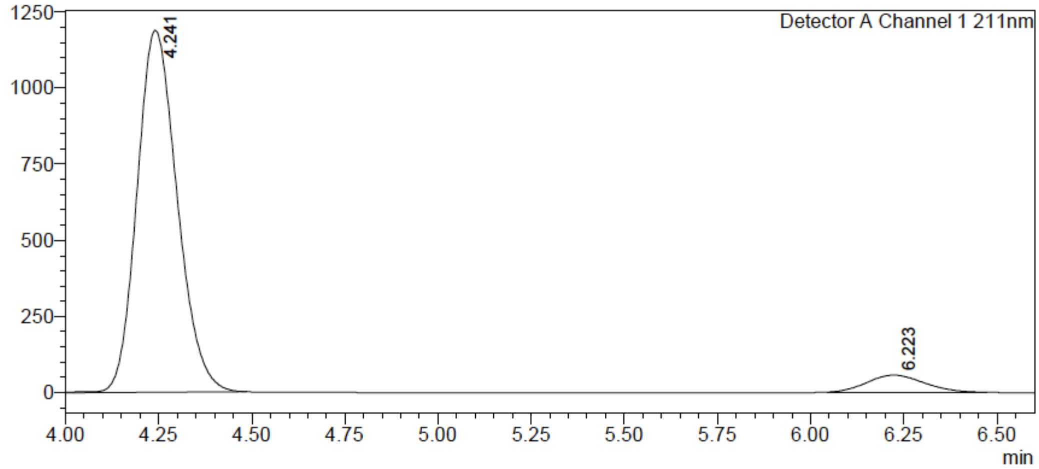

<Peak Table>

Detector A Channel 1 211nm

Peak#	Ret. Time	Area%
1	4.241	93.734
2	6.223	6.266
Total		100.000

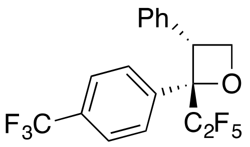

Chiralcel OD-H (97:3 Hexane:IPA, flow rate 1.5 mLmin^{-1}, 211 nm, 40 °C) t_R(2*S*,3*R*): 4.2 min, t_R(2*R*,3*S*): 6.0 min, 95:5 er.

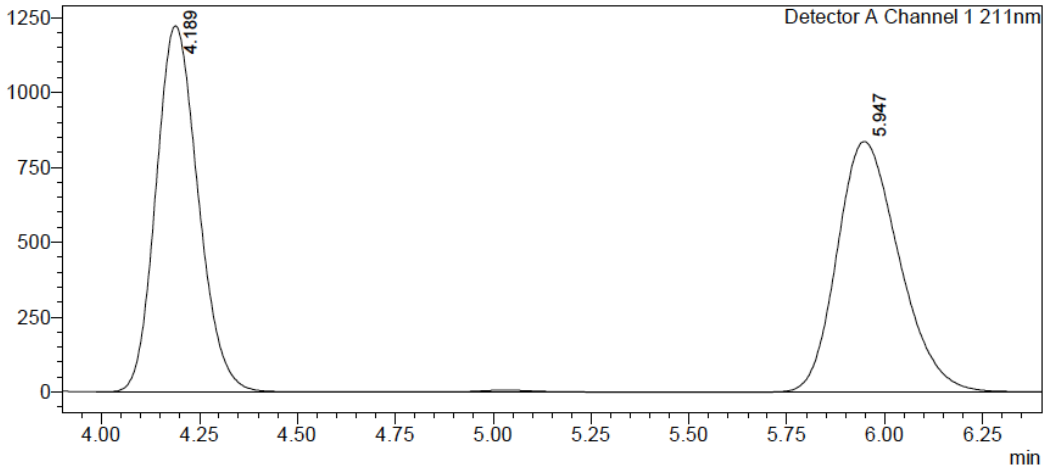

<Peak Table>

Detector A Channel 1 211nm

Peak#	Ret. Time	Area%
1	4.189	49.911
2	5.947	50.089
Total		100.000

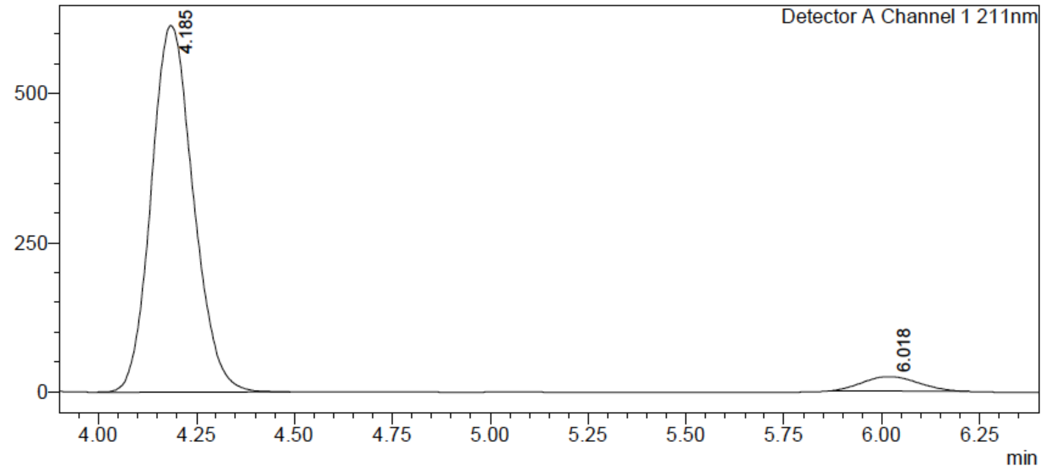

<Peak Table>

Detector A Channel 1 211nm

Peak#	Ret. Time	Area%
1	4.185	94.935
2	6.018	5.065
Total		100.000

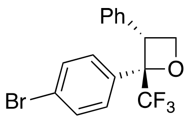

Chiralcel OD-H (97:3 Hexane:IPA, flow rate 1.5 mLmin^{-1}, 211 nm, 40 °C)

t_R(2*S*,3*R*): 4.1 min, t_R(2*R*,3*S*): 6.7 min, 94:6 er.

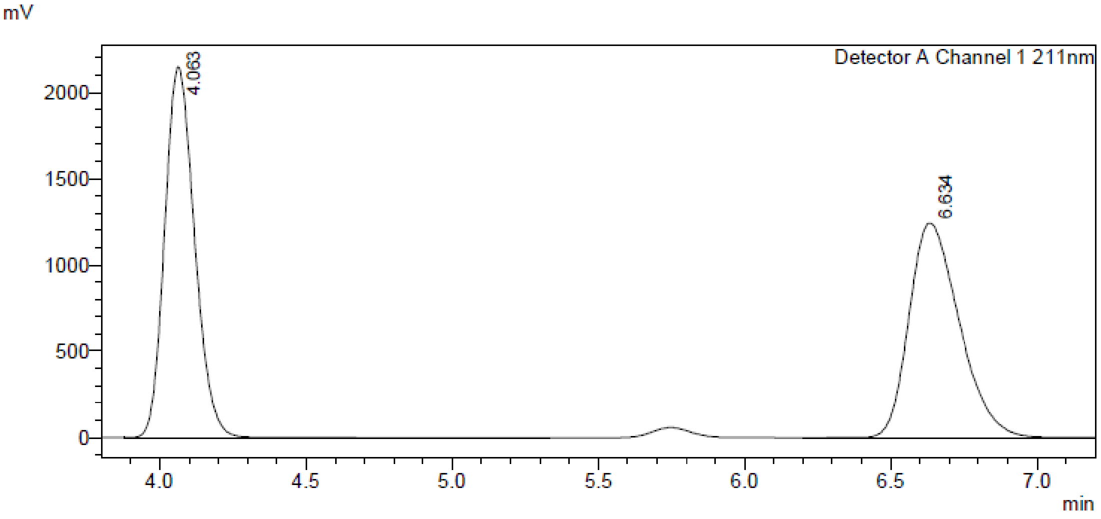

<Peak Table>

Detector A Channel 1 211nm

Peak#	Ret. Time	Area%
1	4.063	50.609
2	6.634	49.391
Total		100.000

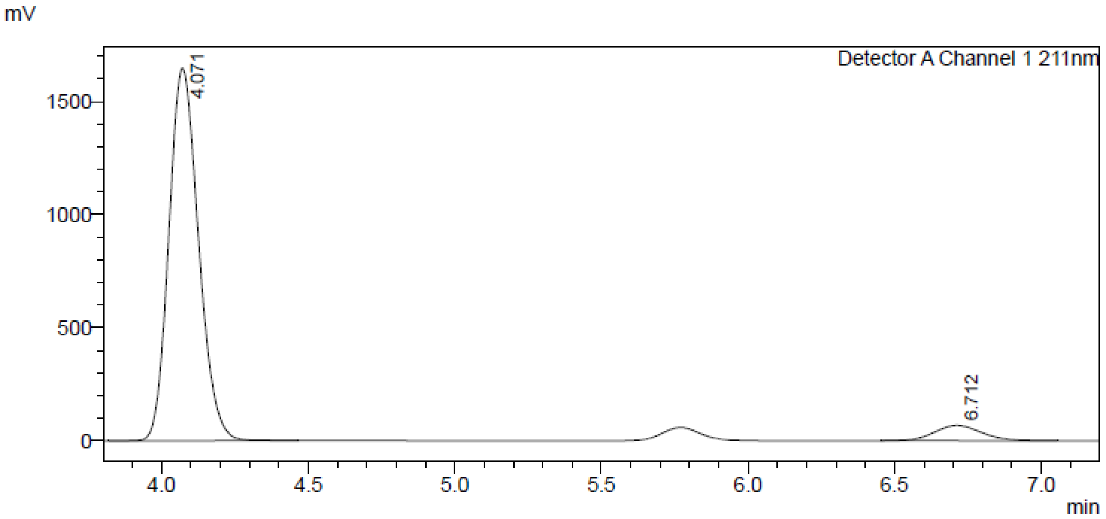

<Peak Table>

Detector A Channel 1 211nm

Peak#	Ret. Time	Area%
1	4.071	93.550
2	6.712	6.450
Total		100.000

References

1. S. R. Smith, C. Fallan, J. E. Taylor, R. McLennan, D. S. B. Daniels, L. C. Morrill, A. M. Z. Slawin and A. D. Smith, *Chem. Eur. J.*, 2015, **21**, 10530–10536.
2. L. C. Morrill, L. A. Ledingham, J.-P. Couturier, J. Bickel, A. D. Harper, C. Fallan and A. D. Smith, *Org. Biomol. Chem.*, 2014, **12**, 624–636.
3. D. G. Stark, L. C. Morrill, D. B. Cordes, A. M. Z. Slawin, T. J. C. O'Riordan and A. D. Smith, *Chem. Asian J.*, 2016, **11**, 395–400.
4. A. T. Davies, A. M. Z. Slawin and A. D. Smith, *Chem. Eur. J.*, 2015, **21**, 18944–18948.
5. Y. Zhao and D. G. Truhlar, *Theor. Chem. Acc.*, 2008, **120**, 215–241.
6. (*a*) R. Ditchfield, W. J. Hehre and J. A. Pople, *J. Chem. Phys.*, 1971, **54**, 724–728; (*b*) W. J. Hehre, R. Ditchfield and J. A. Pople, *J. Chem. Phys.*, 1972, **56**, 2257–2261; (*c*) P. C. Hariharan and J. A. Pople, *Theor. Chem. Acc.*, 1973, **28**, 213–222; (*d*) P. C. Hariharan and J. A. Pople, *Mol. Phys.*, 1974, **27**, 209–214; (*e*) M. S. Gordon, *Chem. Phys. Lett.*, 1980, **76**, 163–168; (*f*) M. M. Francl, W. J. Pietro, W. J. Hehre, J. S. Binkley, D. J. DeFrees, J. A. Pople and M. S. Gordon, *J. Chem. Phys.*, 1982, **77**, 3654–3665; (*g*) R. C. Binning Jr. and L. A. Curtiss, *J. Comp. Chem.*, 1990, **11**, 1206–1216; (*h*) J.–P. Blaudeau, M. P. McGrath, L. A. Curtiss and L. Radom, *J. Chem. Phys.*, 1997, **107**, 5016–5021; (*i*) V. A. Rassolov, J. A. Pople, M. A. Ratner and T. L. Windus, *J. Chem. Phys.*, 1998, **109**, 1223–1229; (*j*) V. A. Rassolov, M. A. Ratner, J. A. Pople, P. C. Redfern and L. A. Curtiss, *J. Comp. Chem.*, 2001, **22**, 976–984.
7. S. Miertuš, E. Scrocco and J. Tomasi, *Chem. Phys.*, 1981, **55**, 117–129.
8. (*a*) J. P. Perdew, K. Burke and M. Ernzerhof, *Phys. Rev. Lett.*, 1996, **77**, 3865–3868; (*b*) J. P. Perdew, K. Burke and M. Ernzerhof, *Phys. Rev. Lett.*, 1997, **78**, 1396.
9. CYLview, 1.0b; Legault, C. Y., Université de Sherbrooke, 2009 (http://www.cylview.org)
